# Asymmetric Synthesis of Quaternary Hydantoins via a Palladium-Catalyzed Aza-Heck Cyclization

**DOI:** 10.1021/jacs.5c16022

**Published:** 2025-11-14

**Authors:** Ellie A. Meck, Humair M. Omer, Montana J. Edwards, Temidayo D. Idowu, Mariah L. Murray, Katerina M. Korch, Donald A. Watson

**Affiliations:** Department of Chemistry and Biochemistry, University of Delaware, Newark, Delaware 19716, United States

## Abstract

An asymmetric, palladium-catalyzed aza-Heck cyclization to prepare enantioenriched 5,5-disubstituted hydantoins (quaternary hydantoins), which are medicinally important compounds but are classically challenging to prepare, is reported. The required substrates can be prepared readily from α,β-unsaturated amides in a single operation, and aza-Heck cyclization provides a wide range of topologically complex, highly substituted, pharmaceutically relevant hydantoins. The capacity of this method to impact the synthesis of drug substances is demonstrated by the first asymmetric synthesis of the anticonvulsant (*R*)-mephenytoin.

## Graphical abstract

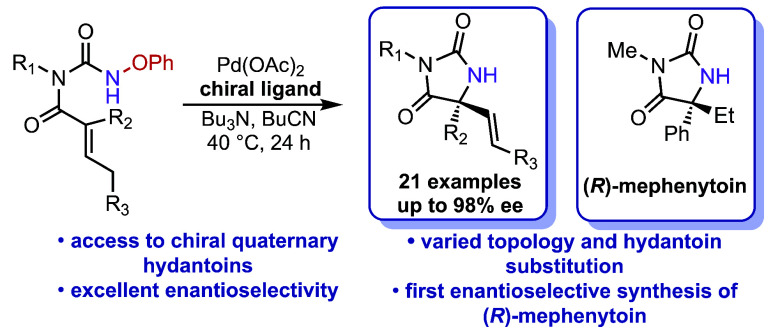

Hydantoins are an important class of N-heterocycles that are widely used as pharmaceutical agents, chiral auxiliaries, and asymmetric catalysts (Figure 1A).^1^ Many hydantoins, including a large number bioactive hydantoins, contain a fully substituted C5-stereogenic center (quaternary hydantoins) and often contain one or more unsubstituted nitrogen atoms. Although classical methods to prepare achiral or racemic hydantoins are known,^2^ asymmetric methods to synthesize enantioenriched quaternary hydantoins remain underdeveloped. Enantioenriched quaternary hydantoins can been prepared from α,α-amino acids^3^ or quaternary α,α-amino nitriles;^4^ however access to these enantioenriched starting materials is often step-intensive and methods to prepare them are frequently limited in scope (Figure 1B).^5^ Additionally, chiral quaternary hydantoins have been accessed via manipulation of a tertiary hydantoin core, using asymmetric Michael reactions.^6^ However, these routes tend to require high step counts and often lead to highly specific C-5 substituents on the hydantoin core.

**Figure 1:**
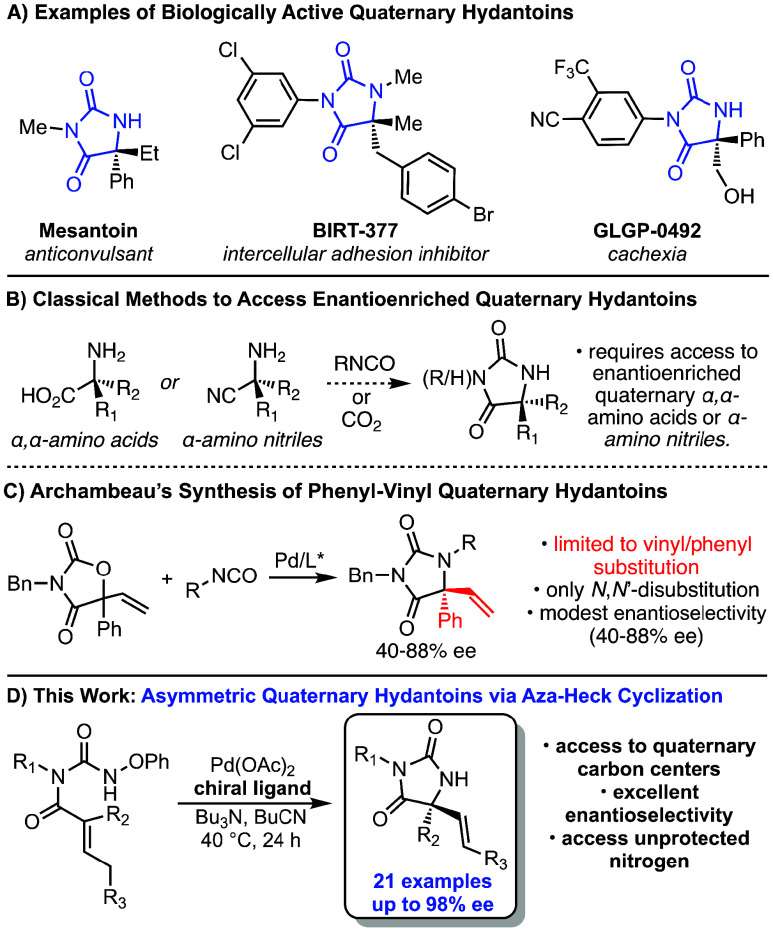
Importance of hydantoins and recent syntheses of chiral quaternary hydantoins.

An alternative and highly attractive approach is the development of reactions that directly form the hydantoin core while establishing the C5-stereocenter. For example, Gong and co-workers developed an asymmetric copper-catalyzed synthesis of hydantoins using ^*t*^Bu-diaziridinone.^7^ Likewise, Foley’s group accessed asymmetric hydantoins using a chiral phosphoric acid-catalyzed Biltz reaction.^8^ However, these methods have only been demonstrated to deliver hydantoins with trisubstituted C5 centers and cannot access chiral quaternary hydantoins. More recently, Archambeau reported a multicomponent catalytic route to hydantoins with quaternary C5 centers and demonstrated some asymmetric examples with good to modest ee (Figure 1C).^9^ However, the report was limited to products where C5 was substituted by a phenyl group and a terminal vinyl group. In addition, this method only produced products with substitution at both nitrogen centers.^10^ Thus, the ability to access chiral quaternary hydantoins with a broad range of substituent patterns and topologies from readily accessible starting materials remains a significant challenge.

We hypothesized that highly substituted hydantoins with diverse substitutions could be accessed using an aza-Heck strategy employing a chiral ligand. In particular, we thought that the formation of fully substituted stereocenters would be possible because of the high degree of substitution tolerated in other aza-Heck reactions developed by us and others to deliver a range of heterocycles, including pyrroles,^11^ lactams,^12^ imidazolinones,^13^ and indolines.^14^ Further, we have also shown that aza-Heck cyclizations readily access heterocycles with N–H functionality, suggesting that access to unprotected or differentially protected quaternary hydantoins might be possible.^12a,13^ However, despite optimism for an asymmetric variant, we recognized that the identification of a chiral ligand would likely present a significant challenge. Although enantioselective carbo-Heck reactions are now mainstream, few aza-Heck reactions enable control of absolute stereochemistry.^15^ Those that are known fall into two groups: those that can access chiral (dihydro)­pyrrolidine and piperidine (Figure 2a) and those that access chiral isoindolinones (Figure 2b). Herein, we report the first asymmetric synthesis of quaternary hydantoins bearing an unsubstituted nitrogen, starting from achiral and acyclic materials. These products can be achieved in high yields and enantioselectivities and are versatile reactions to access chiral quaternary hydantoins with varied topology in a highly straightforward way.

**Figure 2:**
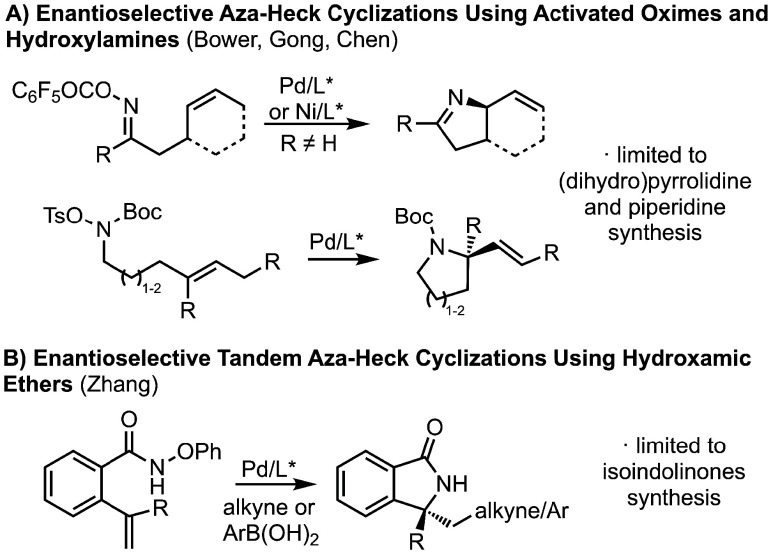
Known Asymmetric Aza-Heck Cyclizations.

Our initial studies investigated the cyclization of unprotected phenoxyamide **1** (R = H) to access fully unprotected hydantoin **2** (eq 1). However, under a variety of catalytic conditions, the desired cyclization product was not observed. We speculate that this is likely due to an unfavorable equilibrium between the extended *s-trans* and folded *s-cis* conformations, the latter of which is required for cyclization. Indeed, ground state calculations for these two conformations of the starting material reveal that **1-** *s-cis* (R = H) is higher in energy by 5.7 kcal/mol compared to the *s-trans* conformer.^16^ We further postulated that the addition of a substituent to the pendant nitrogen would destabilize the extended conformation and favor cyclization.^17^ Calculations also supported this hypothesis; the introduction of *N-*methyl substitution lowers the energy difference between extended and folded conformations to only 0.6 kcal/mol. Harnessing the benefit of *N*-substitution, and with an eye toward later deprotection, we decided to investigate *para*-methoxy benzyl (PMB) substituted starting materials.^18^

1

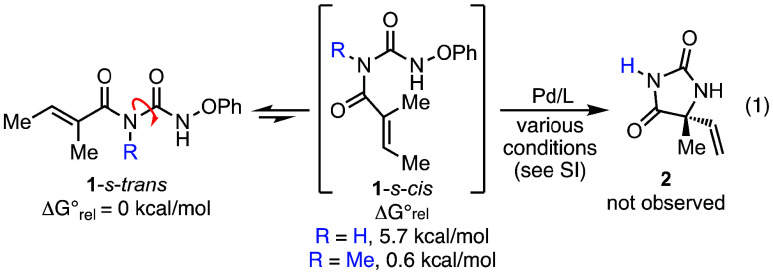




Access to the *N*-substituted aza-Heck starting materials was readily achieved using the route shown in Scheme 1. Deprotonation of α,β-unsaturated secondary amides, such as **3**, with lithium bis­(trimethylsilyl)­amide (LiHMDS), followed by treatment with triphosgene resulted in a trichlorocarbamate intermediate, which can be either isolated or carried through *in situ*,^19^ and subsequent treatment with phenoxyamine hydrochloride and Et_3_N affords phenoxyamide substrates, such as **4**, in high yield.

**Scheme 1:**
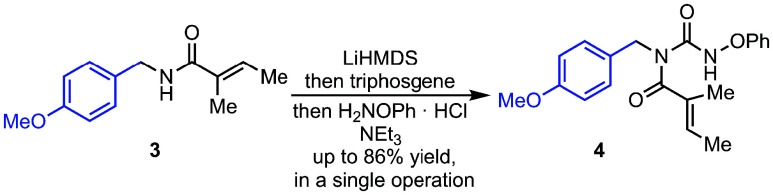
Access to Aza-Heck Substrates

Using **4** as a model system, we then investigated the cyclization to form hydantoin product **5** under palladium-catalyzed conditions. A variety of reaction parameters were studied utilizing high-throughput experimentation (HTE); Pd­(OAc)_2_, acetonitrile, and *n*Bu_3_N were found to be a suitable precatalyst, solvent, and base, respectively.^16^ Phosphoramidite and closely related ligands also proved to be superior. Control experiments proved that a metal catalyst is required for cyclization and base is required to induce enantioselectivity.^16^

A more focused screen of 48 chiral phosphoramidite and phosphite ligands was then performed (selected examples are shown in Scheme 2). Commercially available ligand **L1** [derived from (*S*)-Binol and (*S*)-1-aminoindane] was found to be uniquely effective among those screened, delivering quaternary hydantoin **5** in 57% assay yield and 67% ee. Further optimization studies showed that BuCN was superior to MeCN in terms of enantioselectivity (Table 1, entries 1 and 2). At lower temperatures, both improved yield and selectivity were observed (entry 3). Optimal conditions were found at 40 °C with 5 mol % catalyst loading (Table 1, entries 3–5). Interestingly, the diastereomeric ligand to **L1**, derived from *S*-Binol and R-1-aminoindane (not shown) provided much lower yield and ee, suggesting an important role of the indane stereocenter in enantioinduction.^16^

**Scheme 2:**
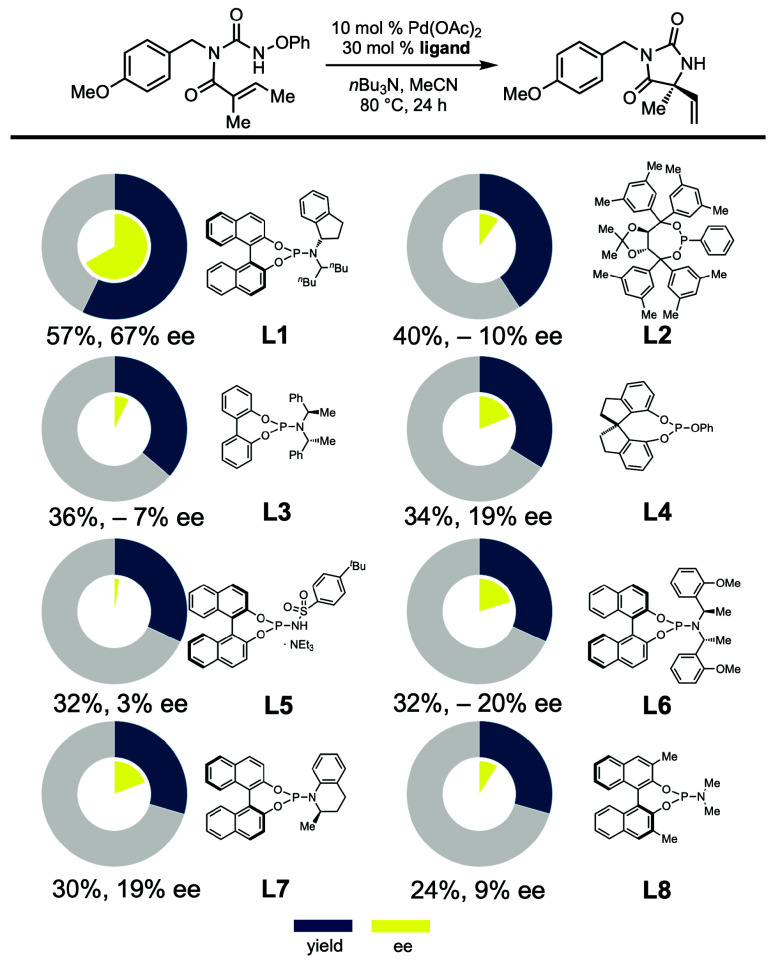
Selected Examples from High-Throughput Experimentation Screen

**Table 1:**
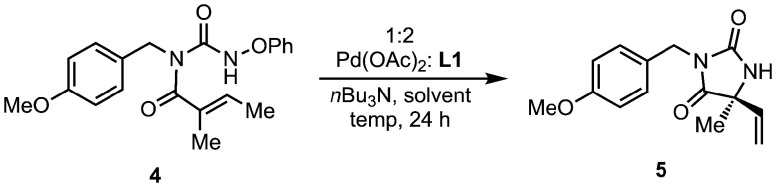
Reaction Optimization

Entry	Pd (mol %)	Solvent	Temp (°C)	Yield **5** ^a^ (%)	ee^b^ (%)
**1**	10	MeCN	80	77	61
**2**	10	BuCN	80	72	77
**3**	10	BuCN	25	97	90
**4**	5	BuCN	25	85	98
**5**	5	BuCN	40	94	98

a 0.1 mmol scale; yield determined by ^1^H NMR analysis with 1,3,5-trimethoxybenzene as an internal standard. Reactions were run at a 0.1 mmol scale in a nitrogen filled glovebox.

b ee of crude product determined by SFC-MS analysis.^20^

These optimized conditions allowed for the synthesis of a wide range of quaternary hydantoins in high yields with outstanding enantioselectivities (Scheme 3). The model hydantoin **5** was isolated in 87% yield and 98% ee on a 0.5 mmol scale. On gram scale, similar yield and enantioselectivity were observed. In general, broad functional group tolerance was observed, including heterocycles, esters, chloride, and fluoride groups (**6–9**). The brominated starting material successfully cyclized to give product **10**; however, the yield was significantly reduced. In this case, byproducts consistent with competitive Ar–Br bond activation were also observed. Notably, the method could also prepare spirocyclic products, which are of significant interest in pharmaceutical production.^21^ For example, hydantoins **11–13** were synthesized in high yields and excellent ee’s, showing that starting materials containing 5–7-membered rings were tolerated. Spirocyclic products bearing additional heterocycles, such as hydantoin **14**, could also be prepared in high yield and ee. Products bearing different substitutions on the α position of the alkene were also tolerated; starting materials bearing both electron-rich aryl (**15**) and methyl silyl ethers (**16**) provided good yields and excellent ee’s upon cyclization. Internal disubstituted alkenes (trans) were also excellent substrates, providing a single *trans*-alkene product in high yield and ee (**17**). Consistent with a Heck-like pathway, tetrasubstituted alkenes proved to be more challenging in these cyclizations.^22^ At 40 °C, using the standard conditions, low yields were observed, but by increasing the temperature to 75 °C product **18** was formed in high yield, albeit with somewhat lower ee. In contrast, product **19**, the starting material of which contains a more complex tetrasubstituted alkene, could be obtained with higher ee but more modest yield. These results illustrate that highly substituted alkenes can participate in this cyclization.

**Scheme 3:**
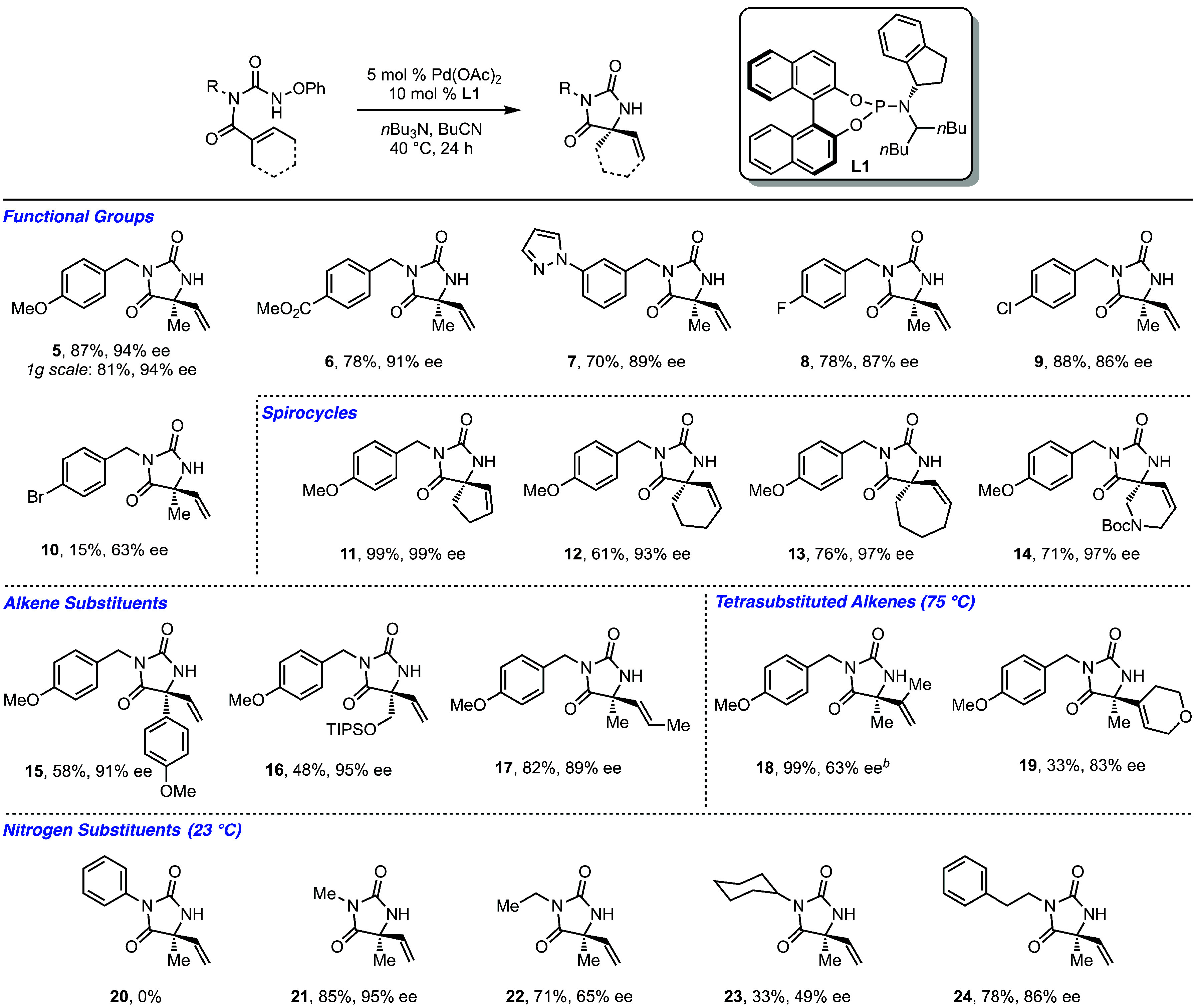
Scope of Benzyl Substituted Quaternary Hydantoins^a^

a Isolated yields, 0.5 mmol scale. Reactions were run with 0.25 M BuCN and 5 equiv of Bu_3_N relative to the electrophile.
b Yield determined by ^1^H NMR analysis using 1,3,5-trimethoxybenzene as the internal standard, 0.1 mmol scale.

We next examined the role of the substituent on the pendant nitrogen. As mentioned previously, when this nitrogen is unsubstituted, no cyclization is observed (eq 1). Additionally, when the substrate contains an *N*-Ph substituent, no cyclization is observed (**20**, Scheme 3). Unlike for the N–H substrate, we believe this to be electronic in nature; similar ground state calculations for the N–Ph substrate show that the *s*-*cis* conformation is energetically accessible.^16^ In contrast, other alkyl substituents on the pendant nitrogen are tolerated, and in these cases superior results were observed at rt compared to at 40 °C.^16^ Methyl-substituted hydantoin **21** was formed in high yield with excellent ee. However, larger alkyl chains, such as Et and Cy, led to lower yield and enantioselectivity (**22** and **23**). Interestingly, examination of the substrate bearing 2-phenethyl at the nitrogen resulted in dramatic recovery of both the yield and ee in the product (**24**). Collectively, these results suggest that the substituent on the pendant nitrogen is intimately involved in the cyclization step and suggests a role for the aromatic group on the nitrogen side chain in the enantio-determining step of the cyclization.^23^

As designed, removal of the PMB-group is readily achieved, allowing access to doubly unprotected hydantoins. Treatment of hydantoin **5** with ceric ammonium nitrate delivered unprotected hydantoin **25** in a high yield (eq 2). This deprotection allowed for assignment of the absolute stereochemistry of the aza-Heck cyclization, with the product corresponding to the known (*S*)-stereoisomer.^16^

2

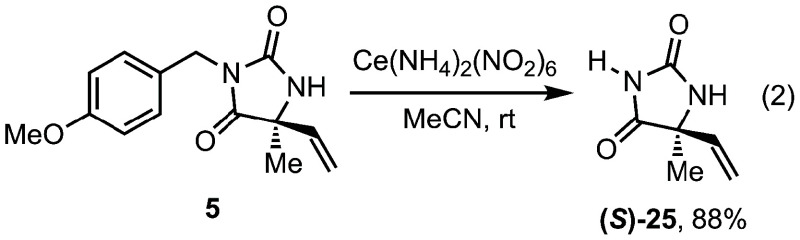




Of note, in several of the cyclizations shown in Scheme 3, a small amount of cyclized side-product bearing an intact N–OPh bond was observed. The presence of this byproduct is consistent with a competitive aza-Wacker cyclization.^24^ For example, in the cyclization of model starting material **4**, in addition to major product **5**, 5% of product **26** was observed (Scheme 4). However, in contrast to hydantoin **5**, product **26** was found to be racemic by using chiral SFC analysis. This was true in all cases examined. This is highly significant, as the lack of enantioenrichment of **26** precludes it being an intermediate in route to enantioenriched **5** (via N–OPh bond reduction) and is further evidence for the aza-Heck cyclization being distinct from aza-Wacker pathways.^12a^ Furthermore, the amount of aza-Wacker product increases with the amount of Pd­(OAc)_2_ used in the reaction, suggesting that its formation may be involved in the pathway for initial reduction of the Pd­(II) precatalyst.^25^ Finally, we do note that in control experiments, small amounts of **26** were found to convert to **5** under the aza-Heck conditions;^16^ this observation likely explains why lower ee’s are observed with higher catalyst loadings (see Table 1, entries 3 and 4).

**Scheme 4:**
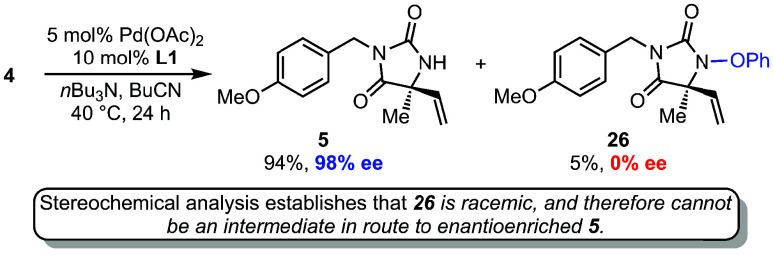
Observation of Aza-Wacker Byproduct Excludes Aza-Wacker Mechanism

Based on these results, we favor a Heck-like pathway wherein oxidative addition into the N–O bond, followed by suprafacial migratory insertion and syn β-hydride elimination, leads to the observed product (Figure 3). Due to the complexity and conformational flexibility of the preferred ligand, detailed computational studies will be required to understand stereoinduction in the migratory insertion; these studies are currently ongoing.

**Figure 3:**
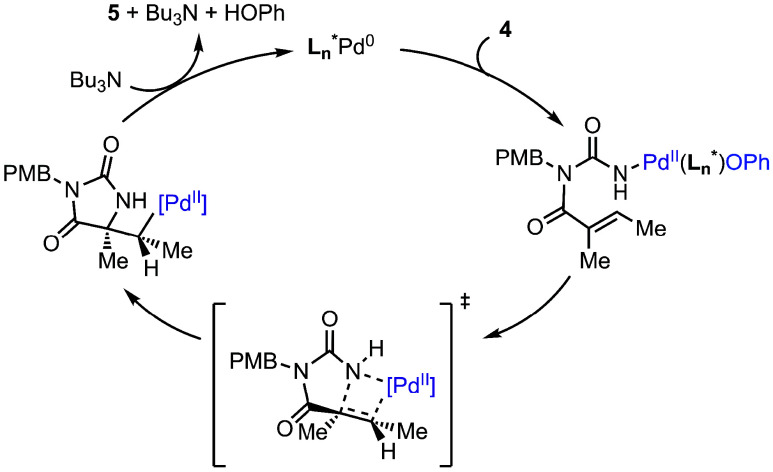
Proposed aza-Heck mechanism.

Finally, to demonstrate the utility of this method, we have established the first enantioselective synthesis of mephenytoin, a compound that has been previously used clinically to treat seizure disorders.^26^ The only prior asymmetric synthesis employed a chiral-auxiliary approach.^27^ Using our standard conditions, substrate **28** (prepared in a single operation from amide **27** as described above) underwent aza-Heck cyclization to access quaternary enantioenriched hydantoin intermediate **29** in 43% yield and 70% ee (Scheme 5). However, using the novel (*S*)-spinol-(*S*)-1-amino-indane-derived ligand **L9**, product **29** could be obtained in 69% yield with outstanding enantioselectivity (91% ee). It is notable that the (*S*)-1-amino-indane fragment of the ligand again proved critical in the enantioselectivity of this cyclization. Reduction of the alkene via hydrogenation then afforded (*R*)-mephenytoin **30** in near quantitative yield, with the optical rotation of the final product matching the reported data. This stereochemical outcome matches that observed in eq 2, giving further confidence in the assigned absolute stereochemistry of the cyclizations.

**Scheme 5:**
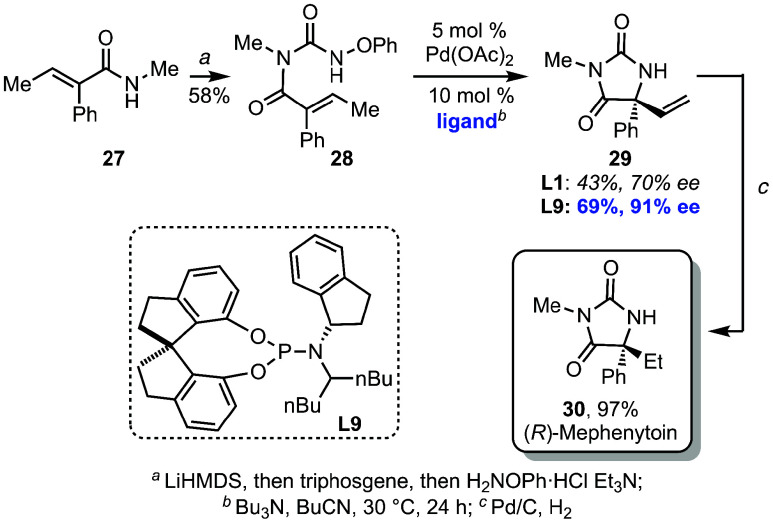
First Enantioselective Synthesis of (*R*)-Mephenytoin

In conclusion, we have developed an asymmetric palladium-catalyzed synthesis of quaternary hydantoins using an aza-Heck approach. We have created a rapid route that converts simple unsaturated amides into the required substrates and have shown that the cyclization proceeds with high enantioselectivity and outstanding functional group tolerance and delivers a wide range of topologically complex hydantoins that can also be readily deprotected. Analysis of side products from the reaction further supports an operable aza-Heck pathway. Finally, we have shown that this method is directly applicable to drug-substances, demonstrating the first asymmetric synthesis of the anticonvulsant (*R*)-mephenytoin.

## Supplementary Material





## Notes

Additional details from optimization studies, detailed experimental procedures and spectral data (PDF)
FID files (ZIP)

## ◆

School of Pharmacy, BRAC University, Dhaka 1212, Bangladesh
