# Supplementary material for: Asymmetric Synthesis of Quaternary Hydantoins via a Palladium-Catalyzed Aza-Heck Cyclization
Source: J Am Chem Soc. 2025 Nov 14;147(49):44692–8. doi: 10.1021/jacs.5c16022 (PMC12703750; doi:10.1021/jacs.5c16022)
Supplement: Supplementary file 1 [file ja5c16022_si_001.pdf]

# Asymmetric Synthesis of Quaternary Hydantoins via a Palladium-Catalyzed Aza-Heck Cyclization

Ellie A. Meck, Humair M. Omer, Montana J. Edwards, Temidayo D. Idowu, Mariah L. Murray, Katerina M. Korch, and Donald A. Watson

Department of Chemistry and Biochemistry, University of Delaware,
Newark, Delaware 19716, United States

## Supporting Information


| Index | Page |
|---|---|
| 1. General Experimental Details | S2 |
| 2. Instrumentation and Chromatography | S2 |
| 3. Ground-State Calculations | S3 |
| 4. High Throughput Experimentation and Optimization | S8 |
| 5. Preparation of Esters and Carboxylic Acids | S20 |
| 6. Preparation of *N*-Phenoxy Amide Substrates | S25 |
| 7. Asymmetric Cyclization to Prepare Hydantoins | S51 |
| 8. Observation and Analysis of Wacker By-product | S60 |
| 9. PMB Deprotection of Hydantoin Product **5** | S62 |
| 10. Synthesis of (*R*)-Mephenytoin **30** | S63 |
| 11. Synthesis of Unprotected Substrate | S65 |
| 12. Preparation of N–Me Substrate and Cyclization | S66 |
| 13. Characterization and Analysis of Trichloromethyl Carbamates | S68 |
| 14. References | S70 |
| 15. Spectra | S72 |

## 1. General Experimental Details

Tetrahydrofuran (THF), dimethyl formamide (DMF), dichloromethane ($CH_2Cl_2$), toluene, 1,4-dioxane, ethyl acetate (EtOAc), and acetonitrile (MeCN) were dried on alumina according to published procedures.[1] Butyronitrile was purchased from VWR, distilled from calcium hydride (40 °C, 30 mtorr), and stored at room temperature in a nitrogen-filled Straus flask. Benzonitrile was purchased from Sigma-Aldrich, distilled from calcium hydride (80 °C, 10 mtorr), and stored at room temperature in a nitrogen-filled Straus flask. Propionitrile was purchased from Sigma-Aldrich, distilled from calcium hydride (100 °C), and stored at room temperature in a nitrogen-filled Straus flask. Pentanenitrile was purchased from Sigma-Aldrich, distilled over calcium hydride (50 °C, 30 mtorr), and stored at room temperature in a nitrogen-filled Straus flask. Cyclopentylmethylether (CPME) was purchased from Sigma-Aldrich, distilled from sodium metal and benzophenone (50 °C, 30 mtorr), and stored at room temperature in a nitrogen-filled Straus flask. Anhydrous dimethylacetamide (DMA) was purchased from Sigma-Aldrich and used as received. Tributylamine was purchased from Sigma-Aldrich, distilled from calcium hydride (50 °C, 50 mtorr), and stored at room temperature in a nitrogen-filled Straus flask. Triethylamine was purchased from Sigma-Aldrich, distilled from calcium hydride (105 °C), and stored at room temperature in a nitrogen-filled Straus flask. All solvents used for purification were purchased from Fisher as A.C.S. grade and used as received. Palladium acetate was purchased from Strem and stored in a nitrogen-filled glovebox at rt. (*E*)-*N*-methyl-2-methyl-2-butenamide,[2] (*E*)-*N*-cyclohexyl-2-methylbut-2-enamide,[3] (*E*)-2-hydroxymethyl-but-2-enoic acid ethyl ester,[4] cyclohept-1-enecarboxylic acid,[5] 2,3-dimethyl-2-butenoic acid,[6] and 2-phenyl-2-butenal[7] were synthesized according to literature procedures. Ligands **L1** and **L9** were prepared using a known literature procedure.[8] ***All operations involving triphosgene, including rotary evaporations, were conducted used inside of a chemical fume hood.*** All other reagents and chemicals were purchased in the highest available analytical purity from commercial suppliers and sparged with nitrogen for 15 minutes prior to use. Vials used in the glovebox were dried in a gravity oven at 140 °C for a minimum of 12 h, transferred into the glovebox hot, and then stored at room temperature in the glovebox prior to use. All other glassware was flame-dried under vacuum prior to use. “Double manifold” refers to a standard Schlenk-line gas manifold equipped with nitrogen and vacuum (ca. 100 mtorr). All optimization reactions (0.1 mmol scale) were run in a nitrogen-filled glovebox in 1-dram vials sealed with a PTFE-lined cap and heated using an aluminum block on a magnetic stir plate. All other reactions were performed using standard Schlenk technique and heated with stirring in temperature-controlled oil baths.

## 2. Instrumentation and Chromatography.

All $^{1}H$, $^{13}C$, and $^{19}F$ NMR spectra were acquired on either a 400 MHz Bruker AVANCE NMR spectrometer equipped with a QNP Cryoprobe, a 600 MHz Bruker NEO NMR spectrometer with a QNP Cryoprobe, or a 600 MHz Bruker AVANCE NMR spectrometer equipped with a Bruker SMART probe. $^{13}C$ NMR were recorded using Attached Proton Test (ATP) phase pulse sequence; carbons with odd number of protons are phased down and those with an even number of protons are phased up. All samples were analyzed in the indicated deutero-solvent and were recorded at ambient temperatures unless otherwise stated. All chemical shifts are reported in ppm. $^{1}H$ NMR spectra were calibrated using the residual protio-signal in deutero-solvents as a standard. $^{13}C$ NMR spectra were calibrated using the deutero-solvent as a standard. IR spectra were recorded on a Nicolet Magma-IR 560 FT-IR spectrometer as thin films on KBr plates. High resolution MS data was acquired at the University of Delaware Mass Spectral Facility on a Thermo Q-Exactive Orbitrap using electrospray ionization (ESI). Unless otherwise noted, column chromatography was performed either by hand or on a Isolera 3 Biotage unit with 40-63 μm silica gel; the eluent is reported in parentheses. Analytical thin-layer chromatography (TLC) was performed on precoated glass plates and visualized by ultraviolet light (UV) or by staining with potassium permanganate ($KMnO_4$), ceric ammonium molybdate (CAM), vanillin, or iodine ($I_2$). Enantiomeric excesses were determined by supercritical fluid chromatography (SFC) on a Waters Acquity UPC2. Optical rotations were recorded on a JASCO P-2000 Polarimeter.

## 3. Ground-State Calculations

Density functional theory (DFT) calculations were carried out using the Gaussian 16 software package.[9] 3D images of the optimized geometries were generated using CYLview.[10] Geometry optimizations and vibrational frequency calculations were done in the gas phase using the B3LYP functional[11] with a 6-31g(d) basis set and GD3 empirical dispersion correction. All optimized geometries have zero imaginary frequencies. Single point energy calculations were carried out on the B3LYP optimized geometries using the M062X[12] functional and a 6-311+G(d,p) basis set with the SMD[13] solvation model and acetonitrile as the solvent. Thermal corrections to the Gibbs free energies and enthalpies were applied at 298.15 K using the harmonic oscillator approximation.

### Relative Energies of Substrate 1A (N-H) and S1 (N-Me)

(i)

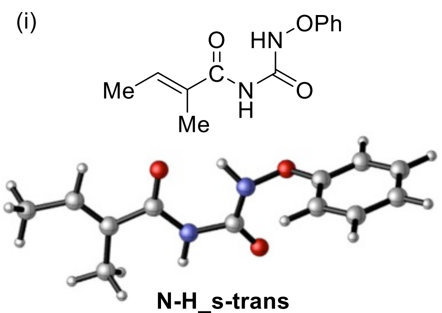


**N-H_s-trans**
ΔG = 0.0 kcal/mol
ΔH = 0.0 kcal/mol

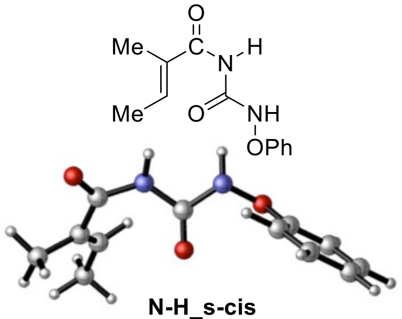


**N-H_s-cis**
ΔG = 5.7 kcal/mol
ΔH = 6.6 kcal/mol

(ii)

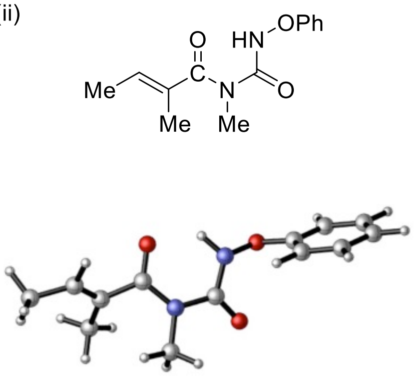


**N-Me_s-trans**
ΔG = 0.0 kcal/mol
ΔH = 0.0 kcal/mol

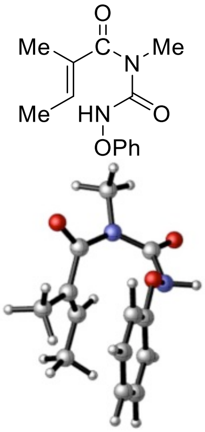


**N-Me_s-cis**
ΔG = 0.6 kcal/mol
ΔH = 0.1 kcal/mol

**Cartesian Coordinates**
**N-H_s-*trans***
B3LYP electronic energy: -800.8562238 a.u.
B3LYP enthalpy: -800.58971 a.u.
B3LYP free energy: -800.654106 a.u.
M062X SCF energy in solution: -800.7675621 a.u.
M062X enthalpy in solution: -800.5010483 a.u.
M062X free energy in solution: -800.5654443 a.u.
Cartesian Coordinates

| ATOM | X | Y | Z |
|---|---|---|---|
| C | 2.33219700 | -0.21747700 | -0.13882200 |
| O | 1.92507500 | -1.34281600 | -0.43269400 |
| C | 3.77263500 | 0.07701900 | 0.16658100 |
| C | 4.61112500 | -0.97295700 | 0.08703000 |
| H | 4.15914600 | -1.92360500 | -0.18983700 |
| C | 4.17685900 | 1.48584200 | 0.53378200 |
| H | 3.97276800 | 2.19313700 | -0.28178200 |
| H | 3.64196300 | 1.84101700 | 1.42462300 |
| H | 5.24345700 | 1.55612200 | 0.75362600 |
| C | 6.08681100 | -1.00563200 | 0.32962700 |
| H | 6.32149300 | -1.72540300 | 1.12534500 |
| H | 6.60783200 | -1.36087400 | -0.56968000 |
| H | 6.51322400 | -0.03911800 | 0.60904300 |
| N | 1.47228400 | 0.86789900 | -0.06655500 |
| H | 1.84607800 | 1.79127800 | 0.10600900 |
| C | 0.08859700 | 0.94111500 | -0.37796700 |
| O | -0.45762500 | 2.02180700 | -0.46493500 |
| N | -0.53641700 | -0.28511200 | -0.46432600 |
| H | 0.04430400 | -1.09123400 | -0.69749200 |
| O | -1.73932900 | -0.26960900 | -1.17304700 |
| C | -2.87312900 | -0.25915100 | -0.37124400 |
| C | -4.07785000 | -0.24983200 | -1.07845500 |
| C | -2.85570100 | -0.26246900 | 1.02182100 |
| C | -5.28070000 | -0.24673800 | -0.37598400 |
| H | -4.05116900 | -0.24205900 | -2.16346600 |
| C | -4.07179100 | -0.26004700 | 1.71038600 |
| H | -1.91131700 | -0.26804200 | 1.55164500 |
| C | -5.28550500 | -0.25245300 | 1.02237300 |
| H | -6.21805700 | -0.23855200 | -0.92571100 |
| H | -4.06204500 | -0.26186600 | 2.79719400 |
| H | -6.22500900 | -0.24869100 | 1.56729600 |

**N-H_s-*cis***
B3LYP electronic energy: -800.8406758 a.u.
B3LYP enthalpy: -800.5744 a.u.
B3LYP free energy: -800.64025 a.u.
M062X SCF energy in solution: -800.7567619 a.u.
M062X enthalpy in solution: -800.4904861 a.u.
M062X free energy in solution: -800.5563361 a.u.
Cartesian Coordinates

| ATOM | X | Y | Z |
|---|---|---|---|
| C | 2.73353600 | -1.13936500 | -0.25721700 |
| O | 3.18333200 | -2.27499300 | -0.25030500 |
| C | 3.44476300 | 0.00086100 | 0.37597500 |
| C | 3.29306000 | 1.24656900 | -0.10797400 |
| H | 2.59431200 | 1.39227000 | -0.92714800 |
| C | 4.39461500 | -0.41996800 | 1.46994600 |
| H | 3.84784700 | -0.90031400 | 2.29036500 |
| H | 5.10442700 | -1.16151000 | 1.08951600 |
| H | 4.95212500 | 0.42339200 | 1.88112400 |
| C | 3.96886900 | 2.50072100 | 0.35131100 |
| H | 4.49141000 | 2.98526200 | -0.48424600 |
| H | 3.21144000 | 3.21433800 | 0.70332700 |
| H | 4.68705400 | 2.34117900 | 1.15843800 |
| N | 1.51695000 | -0.92916200 | -0.96703600 |
| C | 0.43241700 | -0.12036100 | -0.59086500 |
| O | 0.41572200 | 0.71105400 | 0.28490700 |
| N | -0.70771900 | -0.47498200 | -1.35298500 |
| H | -0.53032200 | -0.66286700 | -2.33619700 |
| O | -1.71424900 | 0.50120400 | -1.31076300 |
| C | -2.75782800 | 0.19956800 | -0.44469800 |
| C | -3.77525800 | 1.15638000 | -0.41909800 |
| C | -2.82324000 | -0.94395400 | 0.34827000 |
| C | -4.87565600 | 0.95879800 | 0.41182700 |
| H | -3.68572300 | 2.03780000 | -1.04608100 |
| C | -3.93527200 | -1.12629400 | 1.17511100 |
| H | -2.02424600 | -1.67389900 | 0.31778700 |
| C | -4.96301200 | -0.18374800 | 1.21320100 |
| H | -5.66717800 | 1.70296400 | 0.43255400 |
| H | -3.98950700 | -2.01661400 | 1.79588700 |
| H | -5.82141900 | -0.33430000 | 1.86119500 |
| H | 1.26475500 | -1.79205200 | -1.44182600 |

**N-Me_s-*trans***
B3LYP electronic energy: -840.1574292 a.u.
B3LYP enthalpy: -839.861448 a.u.
B3LYP free energy: -839.928823 a.u.
M062X SCF energy in solution: -840.0542896 a.u.
M062X enthalpy in solution: -839.7583084 a.u.
M062X free energy in solution: -839.8256834 a.u.
Cartesian Coordinates

| ATOM | X | Y | Z |
|---|---|---|---|
| C | -2.18559700 | -0.37106500 | 0.20685100 |
| O | -1.71974400 | -1.42872500 | 0.63404100 |
| C | -3.67348400 | -0.16950300 | 0.14099600 |
| C | -4.38850300 | -1.17405000 | -0.39114000 |
| H | -3.84069000 | -2.04180200 | -0.75487500 |
| C | -4.27923500 | 1.07605900 | 0.76277000 |
| H | -3.54274900 | 1.61737600 | 1.36370700 |
| H | -4.67405200 | 1.77629100 | 0.01707600 |
| H | -5.10486400 | 0.80405200 | 1.42817400 |
| C | -5.87869300 | -1.22839800 | -0.53796500 |
| H | -6.15693300 | -1.36094100 | -1.59205100 |
| H | -6.28436000 | -2.09505000 | 0.00153200 |
| H | -6.37898400 | -0.32986600 | -0.16696700 |
| N | -1.37610200 | 0.68382600 | -0.21674300 |
| C | 0.01674200 | 0.81845800 | 0.09415600 |
| O | 0.57573000 | 1.88875700 | -0.04475300 |
| N | 0.65756200 | -0.34965500 | 0.44902200 |
| H | 0.08800300 | -1.11843700 | 0.80314500 |
| O | 1.82828500 | -0.16062500 | 1.18904800 |
| C | 2.99519000 | -0.24315800 | 0.44241600 |
| C | 4.16731300 | -0.08095600 | 1.18515900 |
| C | 3.03989600 | -0.47042700 | -0.93150200 |
| C | 5.39956600 | -0.15109500 | 0.53929500 |
| H | 4.09284100 | 0.09943200 | 2.25286200 |
| C | 4.28446300 | -0.53929900 | -1.56300600 |
| H | 2.12028300 | -0.59164100 | -1.49013400 |
| C | 5.46627900 | -0.38143400 | -0.83829200 |
| H | 6.31124400 | -0.02426600 | 1.11698700 |
| H | 4.32267600 | -0.71590400 | -2.63477000 |
| H | 6.42846700 | -0.43462000 | -1.33931800 |
| C | -1.91506400 | 1.81419400 | -0.98922900 |
| H | -2.80580700 | 1.48583200 | -1.52468600 |
| H | -2.15496800 | 2.66790600 | -0.35011200 |
| H | -1.15775900 | 2.13121900 | -1.70627500 |

**N-Me_s-*cis***
B3LYP electronic energy: -840.1591695 a.u.
B3LYP enthalpy: -839.863094 a.u.
B3LYP free energy: -839.929685 a.u.
M062X SCF energy in solution: -840.0542203 a.u.
M062X enthalpy in solution: -839.7581448 a.u.
M062X free energy in solution: -839.8247358 a.u.
Cartesian Coordinates

| ATOM | X | Y | Z |
|---|---|---|---|
| C | 1.53758000 | 0.68633700 | -0.97536700 |
| O | 1.78088900 | 0.89158800 | -2.15340600 |
| C | 0.46961400 | 1.44083600 | -0.26133100 |
| C | 0.61298900 | 1.74666000 | 1.04044500 |
| H | 1.48922100 | 1.37842500 | 1.56956700 |
| C | -0.65040000 | 1.95234400 | -1.13930900 |
| H | -0.49492500 | 3.00588100 | -1.40152200 |
| H | -0.69208600 | 1.38780200 | -2.07201600 |
| H | -1.61875800 | 1.86059800 | -0.63934400 |
| C | -0.31969700 | 2.58274300 | 1.86206800 |
| H | -0.62144400 | 2.03569600 | 2.76545500 |
| H | 0.18223200 | 3.49791200 | 2.20505500 |
| H | -1.22268900 | 2.87133800 | 1.31956700 |
| N | 2.39667400 | -0.17926400 | -0.23952400 |
| C | 2.05313100 | -1.06548700 | 0.77653300 |
| O | 2.86915500 | -1.58152900 | 1.52453200 |
| N | 0.69232800 | -1.37859700 | 0.96926900 |
| H | 0.64470100 | -2.26692500 | 1.46580400 |
| O | -0.06062900 | -1.49284700 | -0.22544600 |
| C | -1.37650700 | -1.07208000 | -0.09762200 |
| C | -1.93175800 | -0.56583000 | 1.07603800 |
| C | -2.13441700 | -1.18896300 | -1.26522400 |
| C | -3.26685500 | -0.15242400 | 1.06485100 |
| H | -1.32184900 | -0.47425300 | 1.96551400 |
| C | -3.46466100 | -0.77571500 | -1.25738400 |
| H | -1.66580800 | -1.58644800 | -2.15987900 |
| C | -4.03795600 | -0.25172600 | -0.09430100 |
| H | -3.70105100 | 0.25193700 | 1.97532100 |
| H | -4.05316800 | -0.85982100 | -2.16671500 |
| H | -5.07447800 | 0.07206800 | -0.09245700 |
| C | 3.76280500 | -0.30581500 | -0.77650100 |
| H | 4.39028900 | -0.75596800 | -0.00982600 |
| H | 3.76161900 | -0.92222100 | -1.68023300 |
| H | 4.13666200 | 0.68602800 | -1.03532900 |

## 4. High-Throughput Experimentation and Optimization

### 4.1 Palladium, Ligand, and Solvent Screen with Substrate 4

Vials containing pre-plated ligand (1 μmol, 15 mol% for monodentate ligands and 30 mol% for bidentate ligands) were added to a 96-well reaction block and equipped with stirbars. $[Pd(allyl)Cl]_2$ (0.2 mg, 0.5 μmol, 5 mol%) in MeCN (20 μL) [from a stock solution of $[Pd(allyl)Cl]_2$ (4.6 mg, 12.6 μmol) in MeCN (0.5 mL)], $PdCl_2(COD)$ (0.3 mg, 1 μmol, 10 mol%) in MeCN (20 μL) [from a stock solution of $PdCl_2(COD)$ (7.1 mg, 24.8 μmol) in MeCN (0.5 mL)], $PdCl_2(CH_3CN)_2$ (0.3 mg, 1 μmol, 10 mol%) in MeCN (20 μL) [from a stock solution of $PdCl_2(CH_3CN)_2$ (6.5 mg, 25.0 μmol) in MeCN (0.5 mL)], $PdI_2$ (0.4 mg, 1 μmol, 10 mol%) in MeCN (20 μL) [from a stock solution of $PdI_2$ (9.0 mg, 24.9 μmol) in MeCN (0.5 mL)], $[(cinnamyl)PdCl]_2$ (0.3 mg, 0.5 μmol, 5 mol%) in MeCN (20 μL) [from a stock solution of $[(cinnamyl)PdCl]_2$ (6.5 mg, 12.5 μmol) in MeCN (0.5 mL)], $Pd_2(dba)_3CHCl_3$ (0.5 mg, 0.5 μmol, 10 mol%) in MeCN (20 μL) [from a stock solution of $Pd_2(dba)_3CHCl_3$ (12.9 mg, 12.5 μmol) in MeCN (0.5 mL)], $Pd(OAc)_2$ (0.2 mg, 1 μmol, 10 mol%) in MeCN (20 μL) [from a stock solution of $Pd(OAc)_2$ (5.6 mg, 24.9 μmol) in MeCN (0.5 mL)], and $Pd(TFA)_2$ (0.3 mg, 1 μmol, 10 mol%) in MeCN (20 μL) [from a stock solution of $Pd(TFA)_2$ (8.3 mg, 24.9 mmol) in MeCN (0.5 mL)] were added to individual rows of vials using a micropipettor. Substrate **4** (3.5 mg, 10 μmol, 1 equiv) in MeCN (40 μL) [from a stock solution of substrate **4** (425.3 mg, 1.2 mmol) in MeCN (4.8 mL)] and $n$Bu$_3$N (11.9 μL, 50 μmol, 5 equiv) in MeCN (40 μL) [from a stock solution of $n$Bu$_3$N (1.425 mL, 6.0 mmol) in MeCN (4.8 mL)] were added to each of the vials using a multichannel micropipettor. The reaction block was sealed and stirred at 500 rpm at 80 °C for 24 h. The reaction block was then removed from the glovebox, cooled to rt, and opened to air. MeCN (400 μL) was added to each vial and the reaction mixtures were allowed to stir for 10 minutes. An aliquot of each crude reaction solution (20 μL) was transferred to an SFC analysis collection plate and further diluted with MeCN (480 μL). An aliquot of the diluted reaction solution (20 μL) was transferred to another SFC analysis collection plate and further diluted with MeCN (480 μL). The reactions were analyzed by SFC and integrated against a calibration curve to determine the yield of product and ee formed in each reaction. Reaction conditions for each well and the resulting yields are provided in tabular and graphical format in Table **S1** and Figure **S1**, respectively.

**Table S1.** Ligands, CAS numbers, yields, and ee's from catalyst and ligand HTE screen.

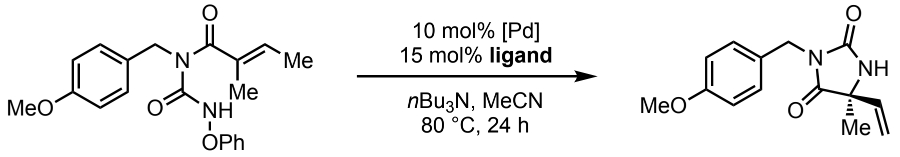


| Entry | Palladium Catalyst | Ligand | Ligand CAS # | Yield (%) | ee (%)[a] |
|---|---|---|---|---|---|
| 1 | $[Pd(allyl)Cl]_2$ | (*R*)-MeO-BIPHEP | 133545-16-1 | 9.6 | 23.3 |
| 2 | $[Pd(allyl)Cl]_2$ | iPr-BPE | 136705-63-0 | 2.7 | 11.2 |
| 3 | $[Pd(allyl)Cl]_2$ | (*S*)-PipPhos | 284472-79-3 | 31.8 | 9.5 |
| 4 | $[Pd(allyl)Cl]_2$ | (*R*)-(-)-4-ethoxydinaphtho[2,1-d:1',2'-f][1,3,2]dioxaphosphepine | 557089-86-8 | 91.7 | 36.6 |
| 5 | $[Pd(allyl)Cl]_2$ | (*R*,*R*)-*N*-Pinap | 828927-97-5 | 12.0 | 30.5 |
| 6 | $[Pd(allyl)Cl]_2$ | (*S*)-4-*tert*-butyl-2-[2-(diphenylphosphino)phenyl]-2-oxazoline | 148461-16-9 | 13.3 | 39.7 |
| 7 | $[Pd(allyl)Cl]_2$ | (*R*)-(−)-1,1′-binaphthyl-2,2′-diyl hydrogenphosphate | 39648-67-4 | 8.1 | 2.3 |
| 8 | $[Pd(allyl)Cl]_2$ | (*R*)-ShiP | 656233-53-3 | 26.6 | 18.8 |

| | | | | | |
|---|---|---|---|---|---|
| 9 | $[Pd(allyl)Cl]_2$ | (*S*)-(+)-(3,5-dioxa-4-phosphacyclohepta[2,1-a;3,4-a']dinaphthalen-4-yl)-5*H*-dibenz[b,f]azepine | 942939-38-0 | 21.2 | 14.6 |
| 10 | $[Pd(allyl)Cl]_2$ | (*S*)-NMDPP | 43077-29-8 | 41.7 | 0.7 |
| 11 | $[Pd(allyl)Cl]_2$ | (3a*R*,8a*R*)-(-)-4,4,8,8-tetrakis(3,5-diethylphenyl)tetrahydro-2,2-dimethyl-6-phenyl-1,3-dioxolo[4,5-e]dioxaphosphepin | 1187446-93-0 | 20.8 | 8.2 |
| 12 | $Pd(COD)Cl_2$ | none | | 8.8 | 0.3 |
| 13 | $Pd(COD)Cl_2$ | (*R*)-MeO-BIPHEP | 133545-16-1 | 4.2 | 51.0 |
| 14 | $Pd(COD)Cl_2$ | iPr-BPE | 136705-63-0 | 0 | 0.0 |
| 15 | $Pd(COD)Cl_2$ | (*S*)-PipPhos | 284472-79-3 | 21.6 | 16.0 |
| 16 | $Pd(COD)Cl_2$ | (*R*)-(-)-4-ethoxydinaphtho[2,1-d:1',2'-f][1,3,2]dioxaphosphepine | 557089-86-8 | 29.5 | 21.3 |
| 17 | $Pd(COD)Cl_2$ | (*R*,*R*)-*N*-Pinap | 828927-97-5 | 8.4 | 38.3 |
| 18 | $Pd(COD)Cl_2$ | (*S*)-4-*tert*-butyl-2-[2-(diphenylphosphino)phenyl]-2-oxazoline | 148461-16-9 | 3.9 | 66.4 |
| 19 | $Pd(COD)Cl_2$ | (*R*)-(−)-1,1′-binaphthyl-2,2′-diyl hydrogenphosphate | 39648-67-4 | 15.9 | 1.2 |
| 20 | $Pd(COD)Cl_2$ | (R)-ShiP | 656233-53-3 | 14.3 | 15.1 |
| 21 | $Pd(COD)Cl_2$ | (*S*)-(+)-(3,5-dioxa-4-phosphacyclohepta[2,1-a;3,4-a']dinaphthalen-4-yl)-5*H*-dibenz[b,f]azepine | 942939-38-0 | 24.0 | 20.3 |
| 22 | $Pd(COD)Cl_2$ | (*S*)-NMDPP | 43077-29-8 | 35.7 | 6.9 |
| 23 | $Pd(COD)Cl_2$ | (3a*R*,8a*R*)-(-)-4,4,8,8-tetrakis(3,5-diethylphenyl)tetrahydro-2,2-dimethyl-6-phenyl-1,3-dioxolo[4,5-e]dioxaphosphepin | 1187446-93-0 | 22.6 | 10.3 |
| 24 | $Pd(COD)Cl_2$ | none | | 14.2 | 1.5 |
| 25 | $PdCl_2(MeCN)_2$ | (R)-MeO-BIPHEP | 133545-16-1 | 27.8 | 24.1 |
| 26 | $PdCl_2(MeCN)_2$ | iPr-BPE | 136705-63-0 | 0.0 | 0.0 |
| 27 | $PdCl_2(MeCN)_2$ | (*S*)-PipPhos | 284472-79-3 | 29.8 | 19.5 |
| 28 | $PdCl_2(MeCN)_2$ | (*R*)-(-)-4-ethoxydinaphtho[2,1-d:1',2'-f][1,3,2]dioxaphosphepine | 557089-86-8 | 2.8 | 43.2 |
| 29 | $PdCl_2(MeCN)_2$ | (*R*,*R*)-*N*-Pinap | 828927-97-5 | 9.6 | 53.4 |
| 30 | $PdCl_2(MeCN)_2$ | (*S*)-4-*tert*-Butyl-2-[2-(diphenylphosphino)phenyl]-2-oxazoline | 148461-16-9 | 4.2 | 68.1 |
| 31 | $PdCl_2(MeCN)_2$ | (R)-(−)-1,1′-binaphthyl-2,2′-diyl hydrogenphosphate | 39648-67-4 | 14.9 | 0.4 |
| 32 | $PdCl_2(MeCN)_2$ | (*R*)-ShiP | 656233-53-3 | 13.0 | 1.9 |

| | | | | | |
|---|---|---|---|---|---|
| 33 | $PdCl_2(MeCN)_2$ | (*S*)-(+)-(3,5-dioxa-4-phosphacyclohepta[2,1-a;3,4-a']dinaphthalen-4-yl)-5*H*-dibenz[b,f]azepine | 942939-38-0 | 32.9 | 25.8 |
| 34 | $PdCl_2(MeCN)_2$ | (*S*)-NMDPP | 43077-29-8 | 30.8 | 5.5 |
| 35 | $PdCl_2(MeCN)_2$ | (3a*R*,8a*R*)-(-)-4,4,8,8-tetrakis(3,5-diethylphenyl)tetrahydro-2,2-dimethyl-6-phenyl-1,3-dioxolo[4,5-e]dioxaphosphepin | 1187446-93-0 | 19.3 | 46.9 |
| 36 | $PdCl_2(MeCN)_2$ | none | | 15.3 | 1.3 |
| 37 | $PdI_2$ | (*R*)-MeO-BIPHEP | 133545-16-1 | 0.0 | 0.0 |
| 38 | $PdI_2$ | iPr-BPE | 136705-63-0 | 0.0 | 0.0 |
| 39 | $PdI_2$ | (*S*)-PipPhos | 284472-79-3 | 0.0 | 0.0 |
| 40 | $PdI_2$ | (*R*)-(-)-4-ethoxydinaphtho[2,1-d:1',2'-f][1,3,2]dioxaphosphepine | 557089-86-8 | 0.0 | 0.0 |
| 41 | $PdI_2$ | (*R*,R*)-N*-Pinap | 828927-97-5 | 0.0 | 0.0 |
| 42 | $PdI_2$ | (*S*)-4-*tert*-butyl-2-[2-(diphenylphosphino)phenyl]-2-oxazoline | 148461-16-9 | 0.0 | 0.0 |
| 43 | $PdI_2$ | (*R*)-(−)-1,1′-binaphthyl-2,2′-diyl hydrogenphosphate | 39648-67-4 | 1.1 | 0.6 |
| 44 | $PdI_2$ | (*R*)-ShiP | 656233-53-3 | 1.6 | 34.3 |
| 45 | $PdI_2$ | (*S*)-(+)-(3,5-dioxa-4-phosphacyclohepta[2,1-a;3,4-a']dinaphthalen-4-yl)-5*H*-dibenz[b,f]azepine | 942939-38-0 | 0.0 | 0.0 |
| 46 | $PdI_2$ | (*S*)-NMDPP | 43077-29-8 | 1.2 | 2.2 |
| 47 | $PdI_2$ | (3a*R*,8a*R*)-(-)-4,4,8,8-tetrakis(3,5-diethylphenyl)tetrahydro-2,2-dimethyl-6-phenyl-1,3-dioxolo[4,5-e]dioxaphosphepin | 1187446-93-0 | 2.2 | 12.7 |
| 48 | $PdI_2$ | none | | 0 | 0 |
| 49 | $[(cinnamyl)PdCl]_2$ | (*R*)-MeO-BIPHEP | 133545-16-1 | 2.7 | 71.2 |
| 50 | $[(cinnamyl)PdCl]_2$ | iPr-BPE | 136705-63-0 | 0.0 | 0.0 |
| 51 | $[(cinnamyl)PdCl]_2$ | (*S*)-PipPhos | 284472-79-3 | 67.4 | 23.0 |
| 52 | $[(cinnamyl)PdCl]_2$ | (*R*)-(-)-4-ethoxydinaphtho[2,1-d:1',2'-f][1,3,2]dioxaphosphepine | 557089-86-8 | 78.1 | 25.3 |
| 53 | $[(cinnamyl)PdCl]_2$ | (*R*,*R*)-*N*-Pinap | 828927-97-5 | 7.3 | 49.0 |
| 54 | $[(cinnamyl)PdCl]_2$ | (*S*)-4-*tert*-butyl-2-[2-(diphenylphosphino)phenyl]-2-oxazoline | 148461-16-9 | 7.0 | 63.3 |
| 55 | $[(cinnamyl)PdCl]_2$ | (*R*)-(−)-1,1′-binaphthyl-2,2′-diyl hydrogenphosphate | 39648-67-4 | 17.0 | 0.4 |
| 56 | $[(cinnamyl)PdCl]_2$ | (*R*)-ShiP | 656233-53-3 | 9.2 | 2.9 |

| | | | | | |
|---|---|---|---|---|---|
| 57 | $[(cinnamyl)PdCl]_2$ | (*S*)-(+)-(3,5-dioxa-4-phosphacyclohepta[2,1-a;3,4-a']dinaphthalen-4-yl)-5*H*-dibenz[b,f]azepine | 942939-38-0 | 4.4 | 65.5 |
| 58 | $[(cinnamyl)PdCl]_2$ | (*S*)-NMDPP | 43077-29-8 | 33.3 | 8.7 |
| 59 | $[(cinnamyl)PdCl]_2$ | (3a*R*,8a*R*)-(-)-4,4,8,8-tetrakis(3,5-diethylphenyl)tetrahydro-2,2-dimethyl-6-phenyl-1,3-dioxolo[4,5-e]dioxaphosphepin | 1187446-93-0 | 29.3 | 1.8 |
| 60 | $[(cinnamyl)PdCl]_2$ | none | | 17.4 | 1.9 |
| 61 | $Pd_2dba_3{\cdot}CHCl_3$ | (*R*)-MeO-BIPHEP | 133545-16-1 | 2.7 | 23.6 |
| 62 | $Pd_2dba_3{\cdot}CHCl_3$ | iPr-BPE | 136705-63-0 | 0.0 | 0.0 |
| 63 | $Pd_2dba_3{\cdot}CHCl_3$ | (*S*)-PipPhos | 284472-79-3 | 25.8 | 21.1 |
| 64 | $Pd_2dba_3{\cdot}CHCl_3$ | (*R*)-(-)-4-ethoxydinaphtho[2,1-d:1',2'-f][1,3,2]dioxaphosphepine | 557089-86-8 | 47.7 | 19.7 |
| 65 | $Pd_2dba_3{\cdot}CHCl_3$ | (*R*,*R*)-*N*-Pinap | 828927-97-5 | 10.4 | 20.2 |
| 66 | $Pd_2dba_3{\cdot}CHCl_3$ | (*S*)-4-*tert*-butyl-2-[2-(diphenylphosphino)phenyl]-2-oxazoline | 148461-16-9 | 8.0 | 35.8 |
| 67 | $Pd_2dba_3{\cdot}CHCl_3$ | (*R*)-(−)-1,1′-binaphthyl-2,2′-diyl hydrogenphosphate | 39648-67-4 | 19.2 | 3.1 |
| 68 | $Pd_2dba_3{\cdot}CHCl_3$ | (*R*)-ShiP | 656233-53-3 | 21.1 | 1.6 |
| 69 | $Pd_2dba_3{\cdot}CHCl_3$ | (*S*)-(+)-(3,5-dioxa-4-phosphacyclohepta[2,1-a;3,4-a']dinaphthalen-4-yl)-5*H*-dibenz[b,f]azepine | 942939-38-0 | 0.0 | 0.0 |
| 70 | $Pd_2dba_3{\cdot}CHCl_3$ | (*S*)-NMDPP | 43077-29-8 | 63.9 | 6.3 |
| 71 | $Pd_2dba_3{\cdot}CHCl_3$ | (3a*R*,8a*R*)-(-)-4,4,8,8-tetrakis(3,5-diethylphenyl)tetrahydro-2,2-dimethyl-6-phenyl-1,3-dioxolo[4,5-e]dioxaphosphepin | 1187446-93-0 | 39.4 | 5.9 |
| 72 | $Pd_2dba_3{\cdot}CHCl_3$ | none | | 53.4 | 1.5 |
| 73 | $Pd(OAc)_2$ | (*R*)-MeO-BIPHEP | 133545-16-1 | 26.8 | 8.9 |
| 74 | $Pd(OAc)_2$ | iPr-BPE | 136705-63-0 | 31.8 | 10.9 |
| 75 | $Pd(OAc)_2$ | (*S*)-PipPhos | 284472-79-3 | 90.6 | 13.7 |
| 76 | $Pd(OAc)_2$ | (*R*)-(-)-4-ethoxydinaphtho[2,1-d:1',2'-f][1,3,2]dioxaphosphepine | 557089-86-8 | 90.7 | 34.6 |
| 77 | $Pd(OAc)_2$ | (*R*,*R*)-*N*-Pinap | 828927-97-5 | 58.4 | 6.3 |
| 78 | $Pd(OAc)_2$ | (*S*)-4-*tert*-butyl-2-[2-(diphenylphosphino)phenyl]-2-oxazoline | 148461-16-9 | 28.2 | 3.1 |
| 79 | $Pd(OAc)_2$ | (*R*)-(−)-1,1′-binaphthyl-2,2′-diyl hydrogenphosphate | 39648-67-4 | 73.4 | 0.3 |
| 80 | $Pd(OAc)_2$ | (*R*)-ShiP | 656233-53-3 | 81.3 | 9.1 |

| | | | | | |
|---|---|---|---|---|---|
| 81 | $Pd(OAc)_2$ | (*S*)-(+)-(3,5-dioxa-4-phosphacyclohepta[2,1-a;3,4-a']dinaphthalen-4-yl)-5*H*-dibenz[b,f]azepine | 942939-38-0 | 88.4 | 22.9 |
| 82 | $Pd(OAc)_2$ | (*S*)-NMDPP | 43077-29-8 | 70.0 | 3.2 |
| 83 | $Pd(OAc)_2$ | (3a*R*,8a*R*)-(-)-4,4,8,8-tetrakis(3,5-diethylphenyl)tetrahydro-2,2-dimethyl-6-phenyl-1,3-dioxolo[4,5-e]dioxaphosphepin | 1187446-93-0 | 60.1 | 0.9 |
| 84 | $Pd(OAc)_2$ | none | | 67.9 | 1.0 |
| 85 | $Pd(TFA)_2$ | (*R*)-MeO-BIPHEP | 133545-16-1 | 17.9 | 47.3 |
| 86 | $Pd(TFA)_2$ | iPr-BPE | 136705-63-0 | 4.7 | 7.2 |
| 87 | $Pd(TFA)_2$ | (*S*)-PipPhos | 284472-79-3 | 72.4 | 15.2 |
| 88 | $Pd(TFA)_2$ | (*R*)-(-)-4-ethoxydinaphtho[2,1-d:1',2'-f][1,3,2]dioxaphosphepine | 557089-86-8 | 99.9 | 37.7 |
| 89 | $Pd(TFA)_2$ | (*R*,*R*)-*N*-Pinap | 828927-97-5 | 24.0 | 4.4 |
| 90 | $Pd(TFA)_2$ | (*S*)-4-*tert*-butyl-2-[2-(diphenylphosphino)phenyl]-2-oxazoline | 148461-16-9 | 20.0 | 10.8 |
| 91 | $Pd(TFA)_2$ | (*R*)-(−)-1,1′-binaphthyl-2,2′-diyl hydrogenphosphate | 39648-67-4 | 44.0 | 1.0 |
| 92 | $Pd(TFA)_2$ | (*R*)-ShiP | 656233-53-3 | 46.8 | 17.0 |
| 93 | $Pd(TFA)_2$ | (*S*)-(+)-(3,5-dioxa-4-phosphacyclohepta[2,1-a;3,4-a']dinaphthalen-4-yl)-5*H*-dibenz[b,f]azepine | 942939-38-0 | 76.9 | 32.5 |
| 94 | $Pd(TFA)_2$ | (*S*)-NMDPP | 43077-29-8 | 46.6 | 7.9 |
| 95 | $Pd(TFA)_2$ | (3a*R*,8a*R*)-(-)-4,4,8,8-tetrakis(3,5-diethylphenyl)tetrahydro-2,2-dimethyl-6-phenyl-1,3-dioxolo[4,5-e]dioxaphosphepin | 1187446-93-0 | 50.3 | 4.2 |
| 96 | $Pd(TFA)_2$ | none | | 40.1 | 1.2 |

[a] The small, non-zero, values observed for enantioenrichment in entries 12, 24, 36, 60, 72, 84, and 96 are consequences of the SFC-MS integration method.

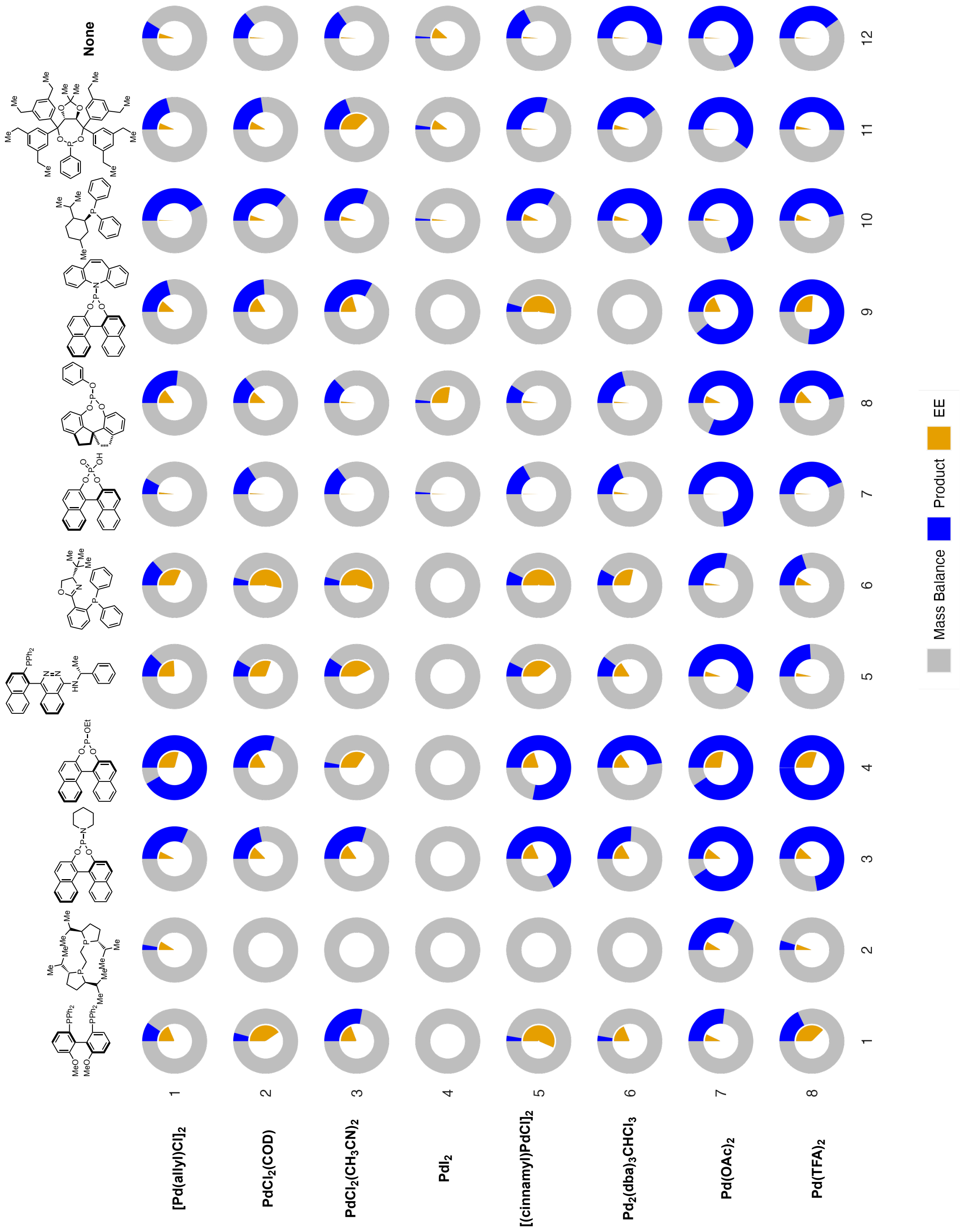


**Figure S1.** Yields and ee's for general HTE plate screen.

## 4.2 Phosphoramidite and monodentate phosphine ligand screen with substrate 4.

Vials containing pre-plated ligand (1 µmol, 15 mol %) were added to a 48-well reaction block and equipped with stirbars. $Pd(OAc_2)$ (0.32 mg, 10 mol%) in MeCN (67 µL) [from a stock solution of $Pd(OAc)_2$ (11.2 mg, 0.5 mmol) in MeCN (5 mL)], substrate **4** ( 3.5 mg, 10 µmol, 1 equiv) and $n$Bu$_3$N (11.9 µL, 50 µmol, 5 equiv) in MeCN (67 µL) [from a stock solution of substrate **4** (177 mg, 0.5 mmol) and $n$Bu$_3$N (595 uL, 2.5 mmol, 5 equiv) in MeCN (5 mL)], were added to each of the vials using a multichannel micropipettor. The reaction block was sealed and stirred at 650 rpm at 80 °C for 24 h. The reaction block was then removed from the glovebox, cooled to rt, and opened to air. MeCN (250 µL) was added to each vial. An aliquot of each crude reaction solution (100 µL) was transferred to an SFC analysis collection plate, further diluted with EtOAc (500 µL), sealed with aluminum sealing tape, and centrifuged for 20 min. The reactions were analyzed by SFC and integrated against a calibration curve to determine the yield and ee of product formed in each reaction. Reaction conditions for each well and the resulting yields are provided in tabular and graphical format in Table **S2** and Figure **S2**, respectively.

**Table S2.** Ligands, CAS numbers, yields, and ee’s from phosphoramidite HTE screen.

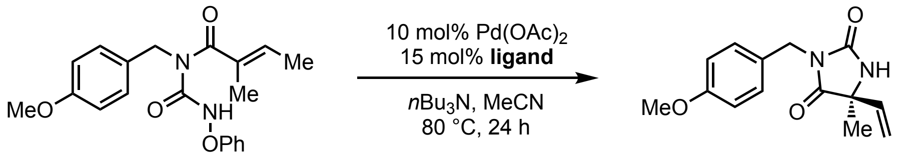


4 5

| Entry | Ligand | Ligand CAS # | Yield (%) | ee (%) |
|---|---|---|---|---|
| 1 | (S)-PipPhos | 284472-79-3 | 19.5 | – 2.0 |
| 2 | (S)-(+)-(3,5-dioxa-4-phospha-cyclohepta[2,1-a;3,4-a’]dinaphthalen-4-yl)bis[(1S)-1-phenylethyl]amine | 380230-02-4 | 15.7 | 30.2 |
| 3 | (S)-(+)-(8,9,10,11,12,13,14,15-octahydro-3,5-dioxa-4-phospha-cyclohepta[2,1-a;3,4-a’] dinaphthalen-4-yl)dimethylamine | 389130-06-7 | 22.9 | – 21.3 |
| 4 | (S)-MonoPhos | 185449-80-3 | 24.4 | 8.8 |
| 5 | (S)-MorfPhos | 185449-81-4 | 24.9 | 15.8 |
| 6 | (S)-SiPhos | 443965-14-8 | 22.4 | 30.0 |
| 7 | (S)-SiPhos-PE | 500997-69-3 | 21.0 | 32.2 |
| 8 | (S)-(+)-(2,6-dimethyl-3,5-dioxa-4-phosphacyclohepta[2,1-a;3,4-a’]dinaphthalen-4-yl)dimethylamine | 185449-86-9 | 29.4 | 8.9 |
| 9 | (R)-1,1’-binapthyl-2,2’-diylethyl phosphite | 557089-86-8 | 19.7 | – 13.8 |
| 10 | (R)-(−)-1,1′-binaphthyl-2,2′-diyl hydrogenphosphate | 39648-67-4 | 17.3 | 4.5 |
| 11 | BIPOL-A1(R) | 500103-26-4 | 36.2 | – 7.3 |
| 12 | (2R)-1-(11bS)-(dinaphtho[2,1-d:1',2'-f][1,3,2]dioxaphosphepin-4-yl)-2-methyl-1,2,3,4-tetrahydroquinoline | 1186392-32-4 | 29.5 | 19.1 |
| 13 | (R)-MOP | 145964-33-6 | 11.0 | 5.0 |
| 14 | (R)-ShiP | 656233-53-3 | 33.9 | 18.9 |
| 15 | (11bR)-N,N-bis[(R)-(-)-1-(2-methoxyphenyl)ethyl]dinaphtho[2,1- | 736158-72-8 | 31.8 | – 20.3 |

| | | | | |
|---|---|---|---|---|
| | d:1',2'-f][1,3,2]dioxaphosphepin-4-amine | | | |
| 16 | (3aR,8aR)-(-)-4,4,8,8-tetrakis(3,5-dimethylphenyl)tetrahydro-2,2-dimethyl-6-phenyl-1,3-dioxolo[4,5-e]dioxaphosphepin | 1019840-96-0 | 40.7 | – 9.7 |
| 17 | (11bS)-N-((R)-2,3-dihydro-1H-inden-1-yl)-N-(di-n-butylmethyl)dinaphtho[2,1-d:1',2'-f][1,3,2]dioxaphosphepin-4-amine | 2019254-27-2 | 57.2 | 66.8 |
| 18 | (S)-(+)-(3,5-dioxa-4-phosphacyclohepta[2,1-a;3,4-a']dinaphthalen-4-yl)-5H-dibenz[b,f]azepine | 942939-38-0 | 23.1 | – 29.2 |
| 19 | (3aR,8aR)-(-)-4,4,8,8-tetrakis(3,5-diethylphenyl)tetrahydro-2,2-dimethyl-6-phenyl-1,3-dioxolo[4,5-e]dioxaphosphepin | 1187446-93-0 | 16.4 | – 8.3 |
| 20 | (S)-(+)-(3,5-dioxa-4-phospha-cyclohepta[2,1-a;3,4-a']dinaphthalen-4-yl)bis[(1R)-1-phenylethyl]amine | 415918-91-1 | 22.8 | 13.8 |
| 21 | (11bS)-N,N-bis[(R)-1-phenylethyl]-dinaphtho[2,1-d:1',2'-f][1,3,2]dioxaphosphepin-4-amine | 497883-22-4 | 26.6 | 36.9 |
| 22 | (11aS)-(-)-10,11,12,13-tetrahydro-5-(1,1-dimethylethyl)diindeno[7,1-de,1',7'-fg)[1.3.2] dioxaphosphocin | 912457-08-0 | 15.3 | – 8.8 |
| 23 | 4-butyl-N-[(11bR)-dinaphtho[2,1-d:1',2'-f][1,3,2]dioxaphosphepin-4-yl]benzenesulfonamide triethylamine adduct | 1150592-91-8 | 31.9 | – 2.6 |
| 24 | (3aR,8aR)-(-)-4,4,8,8-tetrakis(3,5-di-isopropylphenyl)tetrahydro-2,2-dimethyl-6-phenyl-1,3-dioxolo[4,5-e]dioxaphosphepin | 1361146-90-8 | 15.9 | 23.4 |
| 25 | (S)-(+)-(3,5-dioxa-4-phospha-cyclohepta[2,1-a;3,4-a']dinaphthalen-4-yl)benzyl(methyl)amine | 490023-37-5 | 21.4 | 32.5 |
| 26 | (S)-NMDPP | 43077-29-8 | 12.4 | – 17.7 |
| 27 | (S)-4-tert-butyl-2-[2-(diphenylphosphino)phenyl]-2-oxazoline | 148461-16-9 | 26.3 | – 29.7 |
| 28 | (R,R)-N-Pinap | 828927-97-5 | 12.3 | – 15.2 |

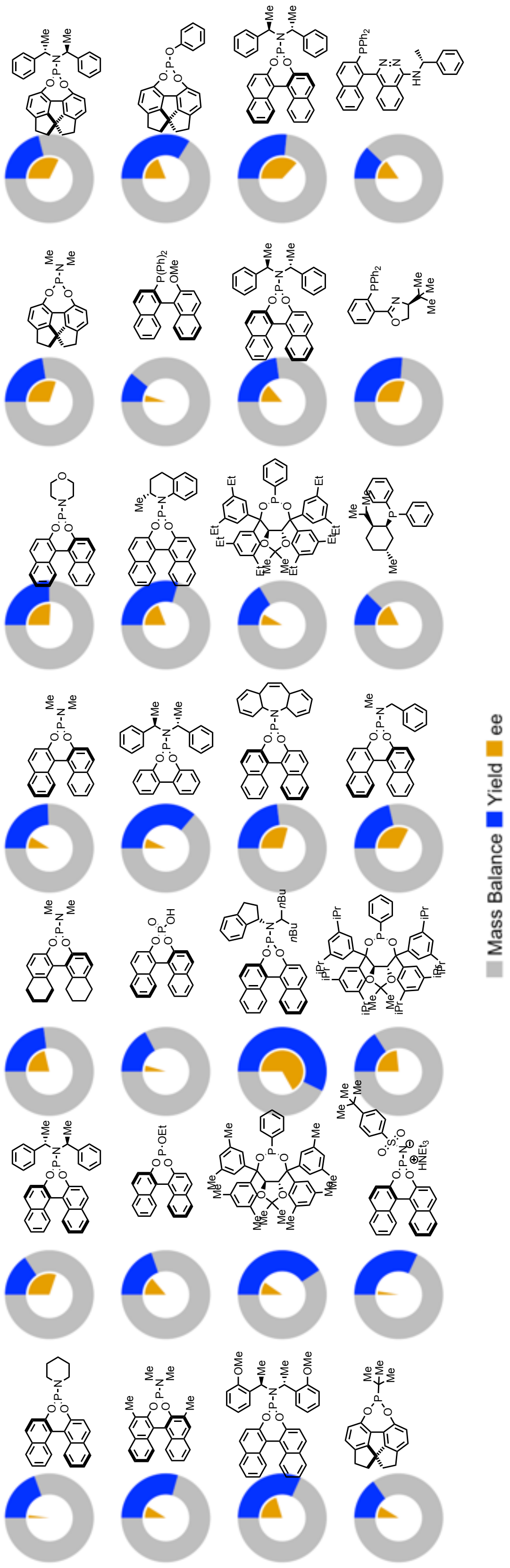


**Figure S2.** Yields and ee's for phosphoramidite ligand HTE screen.

## 4.3 Additional Optimization Details

General Procedure for Optimization of the Asymmetric Cyclization: In a $N_2$-filled glovebox, $Pd(OAc)_2$ (5-10 mol%), (11bS)-N,N-bis[(R)-1-phenylethyl]-dinaphtho[2,1-d:1',2'-f][1,3,2]dioxaphosphepin-4-amine (**L1**) (20-30 mol%), substrate **4** (1 equiv) were added to an oven-dried 1-dram vial containing an 8 mm Teflon-coated stirbar. Anhydrous solvent (0.25 M) was added, the vial was capped, and the mixture was stirred at the indicated temperature for 24 h inside the glovebox in a preheated aluminum-block set atop a stirplate. The reaction vial was then removed from the glovebox, cooled to rt, and concentrated *in vacuo* to give the crude reaction residue. Yields were obtained by $^1H$ NMR against an internal standard (1,3,5-trimethoxybenzene), and ee values were achieved by chiral SFC-MS analysis.

**Table S3. Evaluation of Bases*[a]***

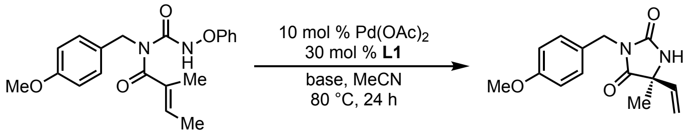


4 5

| Entry | Base | Yield (%) | ee (%) |
|---|---|---|---|
| 1 | $Bu_3N$ | 77 | 61 |
| 2 | $iPr_2NEt$ | 71 | 49 |
| 3 | 2,2,6,6-tetramethylpiperdine | 80 | 58 |
| 4 | *N*-Me-2,2,6,6-tetramethylpiperdine | 58 | 47 |
| 5 | $Cy_2NMe$ | 89 | 63 |
| 6 | $Cy_2NEt$ | 71 | 56 |

*[a]* Conditions: substrate **4** (0.1 mmol), $Pd(OAc)_2$ (10 mol %), **L1** (30 mol %), and base (5 equiv) in MeCN (2 mL) at 80 °C for 24 h. Yield was determined by $^1H$ NMR using 1,3,5-trimethoxybenzene as an internal standard; %ee was determined using SFC-MS analysis.

**Table S4. Evaluation of Solvents[a]**

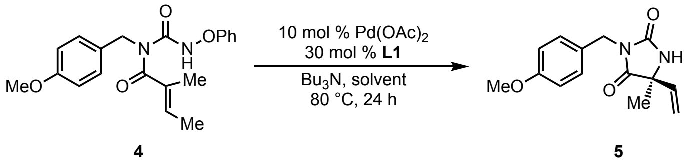

| Entry | Solvent | Yield (%) | ee (%) |
|---|---|---|---|
| 1 | toluene | 72 | 65 |
| 2 | THF | 62 | 67 |
| 3 | 1,4-dioxane | 58 | 63 |
| 4 | CPME | 75 | 72 |
| 5 | DMA | 60 | 63 |
| 6 | DMF | 73 | 67 |
| 7 | EtOAc | 95 | 71 |
| 8 | MeCN | 77 | 61 |
| 9 | benzonitrile | 74 | 69 |
| 10 | propionitrile | 68 | 80 |
| 11 | butyronitrile | 72 | 77 |
| 12 | valeronitrile | 60 | 82 |

[a] Conditions: substrate **4** (0.1 mmol), $Pd(OAc)_2$ (10 mol %), **L1** (30 mol %), and $Bu_3N$ (5 equiv) in solvent (2 mL) at 80 °C for 24 h. Yield was determined by $^1H$ NMR using 1,3,5-trimethoxybenzene as an internal standard; %ee was determined using SFC-MS analysis.

**Table S5. Evaluation of Temperature[a]**

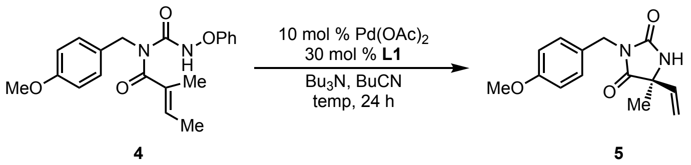


| Entry | Temp (°C) | Yield (%) | ee (%) |
|---|---|---|---|
| 1 | 100 | 47 | 70 |
| 2 | 90 | 70 | 74 |
| 3 | 80 | 72 | 77 |
| 4 | 70 | 87 | 82 |
| 5 | 60 | 85 | 78 |
| 6 | 50 | 81 | 83 |
| 7 | 40 | 86 | 84 |
| 8 | 30 | 85 | 87 |
| 9 | 23 | 97 | 90 |
| 10 | 10 | 63 | 93 |
| 11 | 0 | 34 | 90 |
| 12 | – 30 | 16 | 79 |

[a] Conditions: substrate **4** (0.1 mmol), $Pd(OAc)_2$ (10 mol %), **L1** (30 mol %), and $Bu_3N$ (5 equiv) in BuCN (2 mL) at the designated temperature for 24 h. Yield was determined by $^1H$ NMR using 1,3,5-trimethoxybenzene as an internal standard; %ee was determined using SFC-MS analysis.

## Table S6. Control Experiments[a]

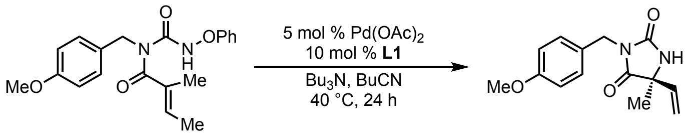


**4** **5**

| Entry | Catalyst | Ligand | Base | Yield (%) | ee (%) |
|---|---|---|---|---|---|
| 1 | $Pd(OAc)_2$ | L1 | $Bu_3N$ | 87 | 98 |
| 2 | $Pd(OAc)_2$ | – | $Bu_3N$ | 10 | 0 |
| 3 | – | – | $Bu_3N$ | 0 | 0 |
| 4 | $Pd(OAc)_2$ | L1 | – | 34 | 48 |

[a] Conditions: substrate **4** (0.1 mmol), $Pd(OAc)_2$ (10 mol %), **L1** (30 mol %), and $Bu_3N$ (5 equiv) in BuCN (2 mL) at the designated temperature for 24 h. Yield was determined by $^1H$ NMR using 1,3,5-trimethoxybenzene as an internal standard; %ee was determined using SFC-MS analysis.

## 4.4 Experiments Using the (S,R) Diastereomer of L1

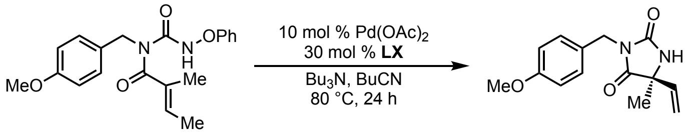


**4** **5**

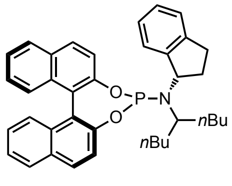


**L1**
79%, 70% ee

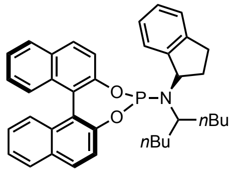


**L10**
40%, 28% ee

## 5. Preparation of Esters and Carboxylic Acids

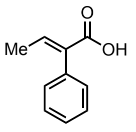


**S1**
Chemical Formula: $C_{10}H_{10}O_2$
Exact Mass: 162.0681
Molecular Weight: 162.1880

(**S1**) **S1** was prepared according to a modified literature procedure.[14] To a 500 mL round bottom flask equipped with a magnetic stirbar was added 2-phenyl-2-butenal (2.2 g, 15 mmol, 1 equiv), acetonitrile (150 mL, 0.1 M), $NaH_2PO_4$ (1.9 g, 21 mmol, 2.6 equiv), 30% aqueous hydrogen peroxide (1.3 mL, 16.5 mmol, 1.1 equiv), and water (150 mL, 0.1 M), before cooling to 0 °C in a ice water bath. $NaClO_2$ in water (1.9 g in 30 mL of water) was then added dropwise over 1 h at 0 °C. The reactions was allowed to warm to rt and maintained. After 16 h, the reaction was quenched with saturated aqueous sodium sulfite until no unreacted hydrogen peroxide was detected by a peroxide test strip. The solution was then acidified with 10% HCl until pH = 1 (as measured by pH paper). The reaction was then extracted with ethyl acetate (3 x 100 mL). The combined organic layers were then dried over $Mg_2SO_4$, filtered, and concentrated *in vacuo* to afford **S1** as a yellow solid (0.50 g, 21% yield) which was used without purification.

**S1** is a known compound. The observed spectra are in accord with published data.[15] Partial characterization is shown below.

$^{1}$H NMR (600 MHz, $CDCl_3$) δ 7.38 (appt ddd, $J$ = 7.5, 6.3, 1.3 Hz, 2H), 7.33 (appt dt, $J$ = 6.6, 2.3 Hz, 1H), 7.31 (d, $J$ = 7.2 Hz, 1H), 7.23–7.17 (m, 2H), 1.78 (d, $J$ = 7.2 Hz, 3H).

$^{13}$C NMR (151 MHz, $CDCl_3$) δ 171.3, 142.9, 134.6, 134.1, 129.9, 128.3, 127.8, 15.9.

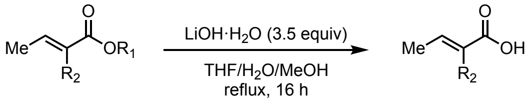


**General Procedure A:** To a round bottom flask equipped with a magnet stirbar was added the ester (1 equiv), $LiOH{\cdot}H_2O$ (3.5 equiv), and THF/$H_2O$/MeOH (4:1:1, 0.17 M total). The flask was then connected to a reflux condenser and heated at reflux overnight under air. The reaction was cooled to room temperature, quenched with 1 M HCl until pH = 1 (as measured by pH paper) and extracted with ethyl acetate (3 x 100 mL). The combined organic layers were then washed with $H_2O$ (100 mL) and brine (100 mL), dried with $Mg_2SO_4$, filtered, and concentrated *in vacuo*. The resulting carboxylic acid was used directly in the next step without purification unless otherwise stated. Spectral data for the crude carboxylic acids are provided for reference.

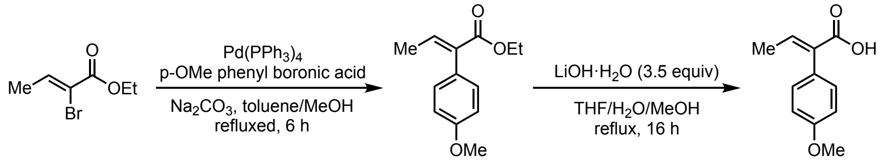


**S2**
Chemical Formula: $C_{13}H_{16}O_3$
Exact Mass: 220.1099
Molecular Weight: 220.2680

**S3**
Chemical Formula: $C_{11}H_{12}O_3$
Exact Mass: 192.0786
Molecular Weight: 192.2140

(**S2**) **S2** was prepared using a modified literature procedure.[16] An oven dried or flame dried three-neck round bottom flask, equipped with a magnetic stirbar and rubber septa, was connect to a double manifold hot via rubber hose and needle and cooled to rt under vacuum. Once cooled, the flask was placed under $N_2$. $Pd(PPh_3)_4$ (0.10 g, 10 mol%, 0.025 equiv) was added quickly by removing a septum. Upon replacement of the septum, the flask was evacuated and backfilled with $N_2$ three times. Anhydrous toluene (10 mL, 0.2 M) was then added via syringe, followed by aqueous $Na_2CO_3$ (2 M in water, 3.5 mL) via syringe. Ethyl-2-bromobut-2-enoate (6.3 g, 33 mmol, 1 equiv) was then added, followed by 4-methoxyphenyl boronic acid (5.3 g, 35 mmol, 1.1 equiv) in EtOH (3.5 mL, 0.5 M) before placing the flask in a preheated oil bath at 80 °C. After 6 h, the reaction was cooled to rt and poured into saturated aqueous $NH_4Cl$ solution (100 mL).

The aqueous layer was extracted with ethyl acetate (3 x 100 mL) and the combined organic layers were dried with $Mg_2SO_4$, filtered, and concentrated *in vacuo*. The crude material was partially purified via flash silica column chromatography (10:90 acetone:hexanes) to afford **S2** (1.2 g) and directly used in the next step. $^1$H and $^{13}$C NMR are provided for identification purposes only.

$^1$H NMR (600 MHz, $CDCl_3$, for identification purposes only) δ 7.25 (d, *J* = 8.8 Hz, 2H), 6.84 (d, *J* = 9.1 Hz, 2H), 6.18 (q, *J* = 7.2 Hz, 1H), 4.30 (q, *J* = 7.1 Hz, 2H), 3.80 (s, 3H), 2.01 (d, *J* = 7.0 Hz, 3H), 1.32 (td, *J* = 7.1, 0.9 Hz, 3H).

$^{13}$C NMR (151 MHz, $CDCl_3$, for identification purposes only) δ 167.6, 159.2, 139.4, 135.3, 131.1, 128.4, 113.8, 60.8, 55.4, 16.0, 14.4.

(**S3**) According to **General Procedure A**, **S2** (2.2 g, 10 mmol, 1 equiv), $LiOH\cdot H_2O$ (1.5 g, 35 mmol, 3.5 equiv) and THF/$H_2O$/MeOH (70 mL, 4:1:1, 0.17 M total) were added to a 250 mL round bottom flask and refluxed overnight (ca. 16 h) to afford **S3** as a yellow solid in 80% purity (1.3 g). **S3** was used without further purification. $^1$H and $^{13}$C NMR are provided for identification purposes only.

$^1$H NMR (400 MHz, $CDCl_3$, for identification purposes only) δ 7.16–7.09 (m, 2H), 6.94–6.89 (m, 2H), 6.87-6.84 (m, 1H), 3.82 (s, 3H), 1.79 (d, *J* = 7.2 Hz, 3H).

$^{13}$C NMR (101 MHz, $CDCl_3$, for identification purposes only) δ 172.1, 159.1, 142.6, 133.7, 131.2, 114.1, 113.7, 55.4, 16.0.

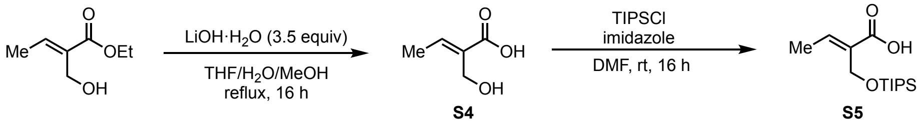


**S4**
Chemical Formula: $C_5H_8O_3$
Exact Mass: 116.0473
Molecular Weight: 116.1160

**S5**
Chemical Formula: $C_{14}H_{28}O_3Si$
Exact Mass: 272.1808
Molecular Weight: 272.4600

(**S4**) According to **General Procedure A**, (*E*)-2-hydroxymethyl-but-2-enoic acid ethyl ester, (4.12 g, 29 mmol, 1 equiv), LiOH·$H_2O$ (4.20 g, 100 mmol, 3.5 equiv) and THF/$H_2O$/MeOH (207 mL, 4:1:1, 0.17 M total) were added to a 250 mL round bottom flask and refluxed overnight (ca. 16 h) to afford **S4** as a yellow solid (1.3 g). **S4** was used without any further purification. $^1$H and $^{13}$C NMR are provided for identification purposes only.

**S4** is a known compound. The observed spectra are in accord with published data.[17] Partial characterization is provided below.

$^1$H NMR (600 MHz, $CDCl_3$, for identification purposes only) δ 7.14 (q, *J* = 7.2 Hz, 1H), 4.38 (s, 2H), 1.95 (d, *J* = 7.3 Hz, 3H).

$^{13}$C NMR (151 MHz, $CDCl_3$, for identification purposes only) δ 172.3, 143.8, 131.2, 56.8, 14.6.

(**S5**) An oven dried or flame dried 100 mL round bottom flask equipped with a magnetic stirbar and a rubber septum was connected to a double manifold hot via rubber hose and needle and cooled to rt under vacuum. Once cooled, the flask was placed under $N_2$, and **S4** (12 mmol, 1.35 g, 1.0 equiv) and imidazole (35 mmol, 4.30 g, 3.0 equiv) were added by quickly removing the septum. After replacing the septum, the flask was evacuated and backfilled with $N_2$ three times, and anhydrous DMF (24 mL, 0.5 M) was added. Chlorotriisopropylsilane (18 mmol, 3.80 mL, 1.5 equiv) was then added dropwise via syringe at rt. After 24 h, the reaction was quenched with saturated aqueous sodium bicarbonate solution (30 mL), and the layers were separtated. The aqueous layer was then extracted with diethyl ether (3 x 30 mL). The combined organic layers were washed with brine (50 mL), dried over $Mg_2SO_4$, filtered, and concentrated *in vacuo* to afford **S5** as a light-yellow oil (9.4 g). The crude material was used directly in the next step without isolation from the excess chlorotriisopropylsilane. $^1$H NMR is provided for identification purposes only.

$^1$H NMR (600 MHz, $CDCl_3$, for identification purposes only) δ 7.08 (q, *J* = 7.6 Hz, 1H), 4.55 (s, 2H), 1.21 – 1.11 (m, 3H), 1.11 – 1.06 (m, 18H).

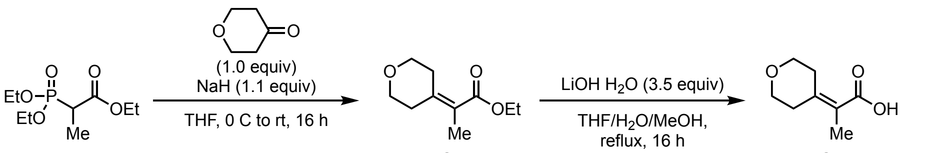


**S6**
Chemical Formula: $C_{10}H_{16}O_3$
Exact Mass: 184.1099
Molecular Weight: 184.2350

**S7**
Chemical Formula: $C_8H_{12}O_3$
Exact Mass: 156.0786
Molecular Weight: 156.1810

(**S6**) An oven dried or flame dried 500 mL round bottom flask, equipped with a magnetic stirbar and rubber septum, was connected to a double manifold via rubber hose and needle and cooled to rt under vacuum. Once cooled, the flask was placed under $N_2$ and NaH (60% suspension in mineral oil, 0.5 g, 11 mmol, 1.1 equiv) was added by quickly removing the septum. After replacing the septum, the flask was evacuated and backfilled with $N_2$ three times. Anhydrous THF (40 mL, 0.25 M) was then added via syringe and the flask was cooled to 0 °C using an ice water bath. Ethyl 2-diethoxyphosphorylpropionate (2.6 mL, 12 mmol, 1.2 equiv) was then added dropwise via syringe, the ice water bath was removed, the reaction was warmed to rt, and stirred for 30 minutes at rt. The reaction was then cooled to 0 °C using an ice water bath, before adding tetrahydro-4*H*-pyran-4-one (0.9 mL, 12 mmol, 1.0 equiv) dropwise via syringe, followed by allowing the reaction to warm to rt. After 16 h, the reaction mixture was concentrated *in vacuo.* The crude material was purified via flash silica column chromatography (0:100 to 15:85 acetone:hexanes) to afford **S6** as a colorless oil (1.5 g, 74% yield).

**S6** is a known compound. The acquired spectra are in accord with published data.[18] Partial characterization is shown below.

$^1$H NMR (600 MHz, $CDCl_3$) δ 4.19 (dq, *J* = 7.0, 0.9 Hz, 2H), 3.73 (t, *J* = 5.3 Hz, 2H), 3.69 (t, *J* = 4.9 Hz, 2H), 2.66 (t, *J* = 6.0 Hz, 2H), 2.37 (t, *J* = 5.5 Hz, 2H), 1.87 (s, 3H), 1.29 (dt, *J* = 7.1, 1.0 Hz, 3H).

$^{13}$C NMR (151 MHz, $CDCl_3$) δ 169.8, 143.0, 121.8, 68.8, 68.3, 60.4, 32.8, 31.9, 15.1, 14.4.

(**S7**) According to **General Procedure A**, **S6** (1.5 g, 8.1 mmol, 1 equiv), $LiOH{\cdot}H_2O$ (1.47 g, 28.4 mmol, 3.5 equiv) and THF/$H_2O$/MeOH (72 mL, 4:1:1, 0.17 M total) were added to a 250 mL round bottom flask and refluxed overnight (ca. 16 h). The reaction was worked up according to General Procedure A, before purification using flash silica column chromatography (0:100 to 50:50 acetone:hexanes) to afford **S7** as a white solid (1.3 g, 70% purity). This partially purified compound was used directly in the next step. $^1$H and $^{13}$C NMR are provided for identification purposes only.

$^1$H NMR (600 MHz, $CDCl_3$, for identification purposes only) δ 3.76 (t, *J* = 5.5 Hz, 2H), 3.72 (t, *J* = 5.5 Hz, 2H), 2.84 (dt, *J* = 4.9, 1.1 Hz, 2H), 2.43 (t, *J* = 5.5 Hz, 2H), 1.92 (s, 3H).

$^{13}$C NMR (151 MHz, $CDCl_3$, for identification purposes only) δ 147.9, 133.9, 120.5, 68.8, 68.3, 33.0, 32.6, 15.1.

## 6. Preparation of N-Phenoxy Amide Substrates

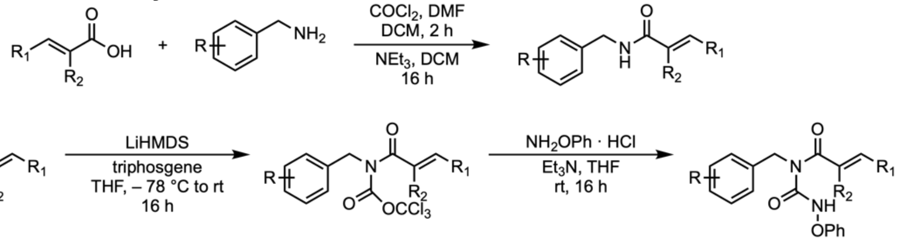


**General Procedure B:** A dried round bottom flask, equipped with a magnetic stirbar and a rubber septum, was attached hot to a double manifold via a rubber hose and needle and cooled under vacuum to rt. Once cooled, the flask was back filled with $N_2$, the septum was quickly removed, carboxylic acid (1 equiv) was added, and the septum was replaced. The reaction flask was then evacuated under vacuum and backfilled with $N_2$ three times. Anhydrous $CH_2Cl_2$ (0.5 M) and anhydrous dimethylformamide (0.05 equiv) were added sequentially via syringe, and the flask was cooled to 0 °C using an ice water bath. Next, oxalyl chloride (1.2 equiv) was added dropwise via syringe (Caution: gas evolution). The reaction mixture was maintained at 0 °C for 15 min, the ice water bath was removed, the reaction was warmed to rt, and stirred at rt. After 2 h and while still connected to the manifold, the flask was evacuated to remove the excess oxalyl chloride and $CH_2Cl_2$ to give a solid. The flask was refilled with $N_2$ and anhydrous $CH_2Cl_2$ (0.5 M) was added via syringe to redissolve the solid.

IA separate dried round bottom flask, equipped with a magnetic stirbar and a rubber septum, attached to a double manifold hot via rubber hose and needle and cooled under vacuum to rt. Once cooled, the flask was evacuated and refilled with $N_2$ three times. Anhydrous $CH_2Cl_2$ (0.5M), anhydrous $NEt_3$ (1.5 equiv), and amine (1.1 equiv) were added sequentially via syringe.

The solution of acid chloride, formed initially, was then added to the amine solution in the second flask via cannula at rt (Caution: extreme bubbling and exothermic). The resulting solution was stirred at rt. After 16 h, the reaction was quenched with saturated aqueous $NH_4Cl$ (50 mL), transferred to a separatory funnel, and the layers were separated. The organic layer was then washed with brine (3 X 50 mL), dried over $Mg_2SO_4$, filtered, and concentrated *in vacuo* to afford the desired amide. Unless otherwise specified, the product was analytically pure (> 95% by $^1$H NMR) and no further purification was needed.

**General Procedure C:** A dried round bottom flask, equipped with a magnetic stirbar and a rubber septum, was connected hot to a double manifold via a rubber hose and needle, and cooled under vacuum to rt. Once cooled, the flask was backfilled with $N_2$, the septum was quickly removed, the synthesized amide (1 equiv) was added, and the septum was replaced. The flask was evacuated and backfilled with $N_2$ four times. Anhydrous THF (0.2 M) was then added via syringe, and the flask was cooled to – 78 °C in a dry ice/acetone bath. Once cooled, LiHMDS (1 M in THF, 1.1 equiv) was added via syringe dropwise, and the mixture was stirred at – 78 °C for 5 min.

In a separate dried round bottom flask, equipped with a magnetic stirbar and rubber septum, was added triphosgene in a chemical fume hood by quickly removing the septum. **(Warning: Caution is required when utilizing triphosgene: weigh and use only in a fume hood)** After replacing the septum, the flask was connected to a double manifold via a rubber hose and needle and filled with $N_2$. Anhydrous THF (1 M) was then added. The resulting solution was then added via syringe dropwise to the lithium amide solution at –78 °C that was prepared in the first step**.** The dryice/acetone bath was removed, the reaction was warmed to rt, and stirred until TLC showed complete conversion to the two carbamate rotamers (usually overnight). The sealed flask was then transferred to a chemical fume hood containing a rotary evaporator and Teflon vacuum pump, and the septum was removed, celite was added, and the solution was concentrated *in vacuo* onto the celite using the rotary evaporator in the hood. **(Note: the pump should also be housed within the fume hood to ensure that any released phosgene is safely removed).** The resulting carbamate was then purified via flash silica column chromatography to give the corresponding trichloro-carbamate. Note: In most cases, the carbamates were produced as a kinetic mixture of two conformers. In all cases investigated, these conformers thermally equilibrated to a single product (***see section 12 for details***); however, the mixture can be carried forward into the next step before reaching equilibrium, as treatment with phenoxyamine and triethylamine lead to a single product. In some cases, full characterization data was collected on the mixture of kinetic trichloro-carbamate products. In other cases,

$^{1}H$ and $^{13}C$ NMR spectra of the initial kinetic mixture are provided for identification purposes only, and the carbamate conformers was carried forward without complete characterization.

A dried round bottom flask, equipped with a magnetic stirbar and a rubber septum, was connected to a double manifold via a rubber hose and needle hot and cooled to rt under vacuum. Once cooled, the flask was backfilled with $N_2$, the septum was quickly removed, *N*-phenoxyamine hydrochloride (2 equiv) was added, and the septum was replaced. The flask was evacuated and backfilled with $N_2$ four times. Anhydrous THF (0.2 M) was then added via syringe. After 5 min., anhydrous $Et_3N$ (3 equiv) was added dropwise via syringe and the reaction was maintained for 1 h.

A separate oven dried round bottom flask, equipped with a magnetic stirbar and a septum, was connected to a double manifold via a rubber hose and needle, and cooled to rt under vacuum. Once cooled, the flask was backfilled with $N_2$, the septum was removed, and the trichloro-carbamate from the previous step was added. After replacing the septum, the flask was evacuated and backfilled with $N_2$ four times. Anhydrous THF (0.5 M) was then added via syringe. The resulting solution was added dropwise to the previously prepared solution of *N*-phenoxyamine hydrochloride and $Et_3N$ via syringe. The resulting reaction was maintained at rt overnight, after which the rubber septum was removed, approximately 1 g of celite was added, and the reaction was concentrated *in vacuo.* Purification via flash silica column chromatography then gave the phenoxyamide substrate.

**General Procedure D (telescoped procedure):** A dried round bottom flask, equipped with a magnetic stirbar and rubber septum, was connected to a double manifold via rubber hose and needle hot, and cooled to rt under vacuum. Once cooled, the flask was backfilled with $N_2$, the septum was quickly removed, the synthesized amide (1 equiv) was added, and the septa was replaced. The flask was then evacuated and backfilled with $N_2$ four times. Anhydrous THF (0.2 M) was then added via syringe, and the flask was cooled to – 78 °C in a dry ice/acetone bath. Once cooled, LiHMDS (1 M in THF, 1.1 equiv) was added dropwise via syringe, and the mixture was stirred at – 78 °C for 5 min.

In a separate round bottom flask equipped with a magnetic stirbar and septum was added triphosgene in a chemical fume hood. **(Warning: Caution is required when utilizing triphosgene: weigh and use only in a fume hood).** The septum was replaced and the flask was connected to a double manifold and filled with $N_2$. Anhydrous THF (1 M) was then added via syringe at rt. The resulting solution was added dropwise to the previously prepared amide solution via syringe at – 78 °C (Caution: gas evolution). The reaction was then the dry ice/acetone bath was removed, the reaction was warmed to rt, and maintained overnight.

A dried round bottom flask, equipped with a magnetic stirbar and a rubber septum, was connected to a double manifold via rubber hose and needle hot and cooled to rt under vacuum. Once cooled, the flask was backfilled with $N_2$, the septum was removed, *N*-phenoxyamine hydrochloride (2 equiv) was added, and the septum was replaced.The flask was then evacuated and backfilled with $N_2$ four times. Anhydrous THF (0.2 M) was then added via syringe. After 5 min., anhydrous $Et_3N$ (3 equiv) was added dropwise via syringe. After 1 h, the trichloro-carbamate formed in the prior step was added dropwise to this flask via syringe. (**Caution: Possible evolution of phosgene left as a byproduct of the trichloro-carbamate formation)** After 16 h, the rubber septum was removed, approximately 1 g of celite was added, and the reaction was concentrated *in vacuo*. Purification via flash silica column chromatography then afforded the phenoxyamide substrate.

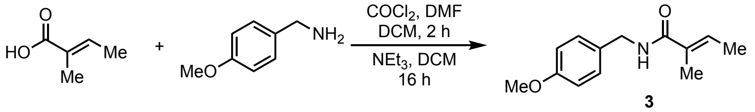


3
Chemical Formula: $C_{13}H_{17}NO_2$
Exact Mass: 219.1259
Molecular Weight: 219.2840

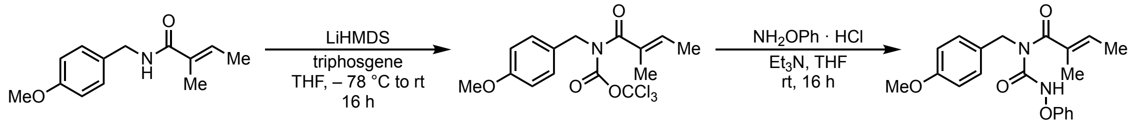


3
Chemical Formula: $C_{13}H_{17}NO_2$
Exact Mass: 219.1259
Molecular Weight: 219.2840

S8
Chemical Formula: $C_{15}H_{16}Cl_3NO_4$
Exact Mass: 379.0145
Molecular Weight: 380.6460

4
Chemical Formula: $C_{20}H_{22}N_2O_4$
Exact Mass: 354.1580
Molecular Weight: 354.4060

(**3**) According to **General Procedure B**, tiglic acid (1.10 g, 20 mmol, 1 equiv), DMF (0.08 mL, 1 mmol, 0.05 equiv), oxalyl chloride (2.18 mL, 24 mmol, 1.2 equiv), and $CH_2Cl_2$ (40 mL, 0.5 M) were added to a 250 mL round bottom flask and reacted at rt for 2 h. 4-Methoxybenzyl amine (2.9 mL, 22 mmol, 1.1 equiv) and $Et_3N$ (4.18 mL, 30 mmol, 1.5 equiv) in $CH_2Cl_2$ (40 mL, 0.5 M) were then added to afford, after reacting at rt for 16 h, **3** as a yellow solid (4.2 g, 95% yield) which was used without further purification.

**3** is a known compound. The acquired spectra are in accord with published data.[19] Partial characterization is shown below.

$^1$H NMR (600 MHz, $CDCl_3$) δ 7.22 (d, *J* = 8.5 Hz, 2H), 6.86 (d, *J* = 10.1 Hz, 2H), 6.44 (q, *J* = 7.6 Hz, 1H), 5.92 (br s, 1H), 4.42 (d, *J* = 7.2 Hz, 2H), 3.79 (s, 3H), 1.84 (s, 3H), 1.74 (d, *J* = 8.1 Hz, 3H).

$^{13}$C NMR (151 MHz, $CDCl_3$) δ 169.3, 159.2, 131.8, 131.0, 130.7, 129.4, 114.2, 55.5, 43.5, 14.0, 12.6.

(**S8**) According to **General Procedure C**, **3** (1.1 g, 5 mmol, 1 equiv), LiHMDS (5.5 mL, 5.5 mmol, 1.1 equiv), triphosgene (1.5 g, 5 mmol, 1 equiv) and THF (25 mL, 0.2 M) were added to a 250 mL round bottom flask. After 16 h, the reaction was worked up according to general procedure C and purified using flash silica gel chromatography (0:100 to 15:85 ethyl acetate:hexanes) to provide **S8** as a 46:54 mixture of conformers that are kinetically stable at rt (1.2 g).

**S8** as a mixture of kinetically stable conformers (1.4 g, 3.6 mmol, 1 equiv), phenoxyamine hydrochloride (1.1 g, 7.2 mmol, 2 equiv), $Et_3N$ (1.5 mL, 10.8 mmol, 3 equiv) and THF (36 mL, 0.2 M) were then added to a 100 mL round bottom flask. After 16 h at rt, the reaction was worked up according to general procedure C and purified using flash silica gel chromatography (0:100 to 15:85 ethyl acetate:hexanes) to afford **4** as a light tan solid (0.46 g, 48% yield).

Data for **S8** (Tabulated as a mixture of two conformers where $^1$H and $^{13}$C NMR spectra are provided for identification purposes only):

$^1$H NMR (400 MHz, $CDCl_3$. Note: Spectrum shows mixture of kinetically stable conformers at rt, please see spectrum for details. Diagnostic peaks given.) δ 7.36–7.31 (m), 6.18 (dq, *J* = 1.3, 7.1 Hz), 6.00 (dq, *J* = 1.3, 5.2 Hz), 4.87 (s), 4.82 (s),1.91-1.87 (m), 1.86-1.85 (m).

$^{13}$C NMR (101 MHz, $CDCl_3$. Note: Spectrum shows mixture of kinetically stable conformers at rt, please see spectrum for details. Useful diagnostic peaks given.) δ 176.3, 159.5, 139.9, 137.9, 130.3, 129.2, 114.3, 59.1, 55.4, 51.9, 50.8, 49.5, 23.5, 14.5, 11.8.

Data for **4**:

$^1$H NMR (600 MHz, $CDCl_3$) δ 11.37 (s, 1H), 7.32–7.27 (m, 2H), 7.14–7.10 (m, 2H), 7.08–7.05 (m, 2H), 7.03 (tt, *J* = 7.4, 1.0 Hz, 1H), 6.85 (dt, *J* = 8.7, 3.1 Hz, 2H), 5.81–5.74 (dq, *J* = 7.0, 1.4 Hz, 1H), 4.96 (s, 2H), 3.79 (s, 3H), 1.74 (t, *J* = 1.3 Hz, 3H), 1.72 (dt, *J* = 6.8, 1.1 Hz, 3H).

$^{13}$C NMR (151 MHz, $CDCl_3$) δ 176.9, 159.7, 159.2, 156.3, 132.2, 129.6, 129.6, 129.5, 128.8, 123.0, 114.2, 113.4, 55.4, 49.4, 13.9, 13.5.

FTIR (cm$^{-1}$) 3238 (br), 2935, 2836, 1725, 1662, 1612, 1592, 1514, 1489, 1457.

HRMS (ESI) m/z, calculated for $[C_{20}H_{23}N_2O_4]^+$ ($[M+H]^+$): 355.1658, found: 355.1648.

MP = 70–73 °C.

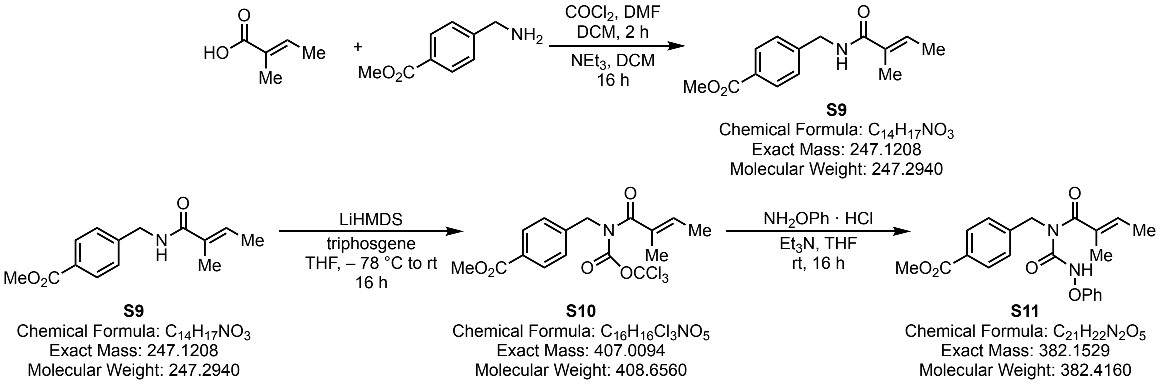


(**S9**) According to **General Procedure B**, tiglic acid (2.0 g, 20 mmol, 1 equiv), DMF (0.08 mL, 1 mmol, 0.05 equiv), oxalyl chloride (2.18 mL, 24 mmol, 1.2 equiv), and $CH_2Cl_2$ (40 mL, 0.5 M) were added to a 250 mL round bottom flask. After 2 h at rt, 4-methylester benzyl amine hydrochloride (3.6 g, 22 mmol, 1.1 equiv), $Et_3N$ (7.0 mL, 50 mmol, 1.5 equiv), and $CH_2Cl_2$ (40 mL, 0.5 M) were added. After 16 h at rt, **S9** was afforded as a dark yellow solid (4.8g, 95% yield) and used without further purification.

$^{1}$H NMR (600 MHz, $CDCl_3$) δ 8.00 (d, $J$ = 8.4 Hz, 2H), 7.35 (d, $J$ = 8.6 Hz, 2H), 6.49 (qq, $J$ = 6.9, 1.5 Hz, 1H), 6.05 (br s, 1H), 4.56 (d, $J$ = 5.9 Hz, 2H), 3.91 (s, 3H), 1.87 (quint, $J$ = 1.2 Hz, 3H), 1.77 (dq, $J$ = 6.9, 1.2 Hz, 3H).

$^{13}$C NMR (151 MHz, $CDCl_3$) δ 169.4, 167.0, 144.0, 131.5, 130.2, 129.5, 127.8, 52.3, 45.9, 43.5, 14.1, 12.6.

FTIR (cm$^{-1}$) 3305 (br), 2950, 1721, 1662, 1613, 1626, 1279, 1110, 1019, 750.

HRMS (ESI) m/z, calculated for $[C_{14}H_{18}NO_3]^+$ ($[M+H]^+$): 248.1287, found: 248.1289.

MP = 104–107 °C.

(**S11**) According to **General Procedure C, S9** (1.2 g, 5 mmol, 1 equiv), LiHMDS (5.5 mL, 5.5 mmol, 1.1 equiv), triphosgene (1.5 g, 5 mmol, 1 equiv) and THF (25 mL, 0.2 M) were added to a 250 mL round bottom flask. After 16 h at rt, the reaction was worked up according to procedure C and purified using flash silica gel chromatography (0:100 to 15:85 ethyl acetate:hexanes) to provide **S10** as a 36:64 mixture of conformers that are kinetically stable at rt (1.14 g).

**S10** as a mixture of kinetically stable conformers (2.7 mmol, 1.1 g, 2.7 mmol, 1 equiv), phenoxyamine hydrochloride (0.8 g, 5.4 mmol, 2 equiv), $Et_3N$ (1.1 mL, 8.1 mmol, 3 equiv), and THF (14 mL, 0.2 M) were then added to a 50 mL round bottom flask. After 16 h at rt, the reaction was worked up according to procedure C and purified using flash silica gel chromatography (0:100 to 15:85 ethyl acetate:hexanes) to afford **S11** as a light tan solid (0.46 g, 48% yield).

Data for **S10** (Tabulated as a mixture of two conformers where $^{1}$H and $^{13}$C NMR spectra are provided for identification purposes):

$^1H$ NMR (600 MHz, $CDCl_3$, Note: Spectrum shows mixture of kinetically stable conformers at rt, please see spectrum for details. Useful diagnostic peaks given.) δ 7.45 (dd, *J* = 8.4, 1.6 Hz), 7.40 (dd, *J* = 8.1, 1.3 Hz), 6.24 (qd, *J* = 7.0, 2.3 Hz), 6.06 (qt, *J* = 6.9, 1.3 Hz), 4.98 (s), 1.92-1.90 (m), 1.88-1.86 (m).

$^{13}C$ NMR (151 MHz, $CDCl_3$, Note: Spectrum shows mixture of kinetically stable conformers at rt, please see spectrum for details. Useful diagnostic peaks given.) δ 173.4, 166.7, 149.0, 147.7, 140.5, 137.8, 134.3, 130.3, 129.9, 52.2, 51.7, 49.2, 14.6, 14.1, 13.0, 12.8.

Data for **S11**:

$^1H$ NMR (600 MHz, $CDCl_3$) δ 11.35 (s, 1H), 7.95–7.91 (m, 2H), 7.26–7.21 (m, 2H), 7.19–7.16 (m, 2H), 7.03–7.00 (m, 2H), 6.98 (tt, *J* = 7.3, 1.1 Hz, 1H), 5.66 (qq, *J* = 6.8, 1.5 Hz, 1H), 5.00 (s, 2H), 3.85 (s, 3H), 1.66 (quint, *J* = 1.2 Hz, 3H), 1.61 (dt, *J* = 6.9, 1.2 Hz, 3H).

$^{13}C$ NMR (151 MHz, $CDCl_3$) δ 176.7, 166.8, 159.6, 156.1, 142.7, 131.9, 130.2, 129.8, 129.6, 129.5, 127.0, 123.2, 113.4, 52.3, 49.7, 13.9, 13.5.

FTIR ($cm^{-1}$) 3249 (br), 2952, 1722, 1664, 1489, 1281, 1019.

HRMS (ESI) m/z, calculated for $[C_{21}H_{23}N_2O_5]^+$ ($[M+H]^+$): 383.1607, found: 383.1613.

MP = 81–83 °C.

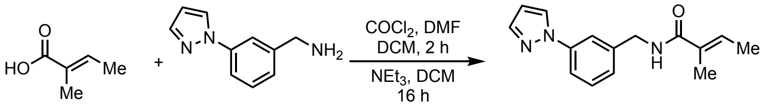


**S12**
Chemical Formula: $C_{15}H_{17}N_3O$
Exact Mass: 255.1372
Molecular Weight: 255.3210

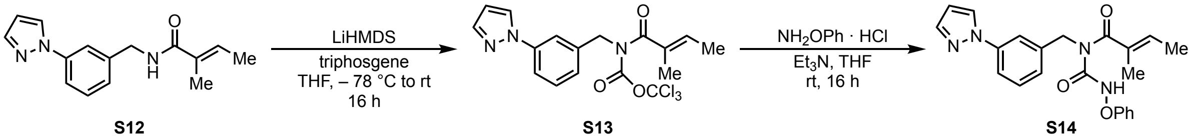


**S12**
Chemical Formula: $C_{15}H_{17}N_3O$
Exact Mass: 255.1372
Molecular Weight: 255.3210

**S13**
Chemical Formula: $C_{17}H_{16}Cl_3N_3O_3$
Exact Mass: 415.0257
Molecular Weight: 416.6830

**S14**
Chemical Formula: $C_{22}H_{22}N_4O_3$
Exact Mass: 390.1692
Molecular Weight: 390.4430

(**S12**) According to **General Procedure B**, tiglic acid (1.0 g, 10 mmol, 1 equiv), DMF (0.04 mL, 0.5 mmol, 0.05 equiv), oxalyl chloride (1.1 mL, 12 mmol, 1.2 equiv), and $CH_2Cl_2$ (20 mL, 0.2 M) were added to a 250 mL round bottom flask. After 2 h at rt, [3-(1H-pyrazol-3-yl)phenyl]methylanamine (1.7 mL, 11 mmol, 1.1 equiv), $Et_3N$ (2.1 mL, 15 mmol, 1.5 equiv), and $CH_2Cl_2$ (20 mL, 0.2 M) were added. After 16 h at rt, **S12** was afforded as a thick orange oil (2.30 g) and used without further purification. $^1H$ and $^{13}C$ NMR are provided for identification purposes only.

$^1H$ NMR (600 MHz, $CDCl_3$) δ 7.93 (d, *J* = 2.4 Hz, 1H), 7.73 (d, *J* = 1.8 Hz, 1H), 7.67 (t, *J* = 2.2 Hz, 1H), 7.59 (dd, *J* = 8.1, 1.3 Hz, 1H), 7.42 (t, *J* = 7.9 Hz, 1H), 7.25 (d, *J* = 7.6 Hz, 1H), 6.50–6.47 (m, 2H), 6.04 (br s, 1H), 4.58 (d, *J* = 5.9 Hz, 2H), 1.87 (t, *J* = 1.3 Hz, 3H), 1.76 (dd, *J* = 7.0, 1.2 Hz, 3H).

$^{13}C$ NMR (151 MHz, $CDCl_3$) δ 169.4, 141.2, 140.6, 140.5, 131.7, 131.4, 130.0, 127.1, 126.1, 118.9, 118.4, 107.9, 43.7, 14.1, 12.6.

(**S14**) According to **General Procedure C**, **S12** (1.3 g, 5 mmol, 1 equiv), LiHMDS (5.5 mL, 5.5 mmol, 1.1 equiv), triphosgene (1.5 g, 5 mmol, 1 equiv), and THF (25 mL, 0.2 M) were added to a 250 mL round bottom flask. After 16 h at rt, the reaction was worked up according to general procedure C and purified using flash silica gel chromatography (0:100 to 15:85 acetone:hexanes) to provide **S13** as a 46:54 mixture of conformers that are kinetically stable at rt (1.1 g).

**S13** as a mixture of kinetically stable conformers (1.0 g, 2.5 mmol, 1 equiv), phenoxyamine hydrochloride (0.73 g, 5 mmol, 2 equiv), $Et_3N$ (1.0 mL, 7.5 mmol, 3 equiv), and THF (12.5 mL, 0.2 M) were added to a 100 mL round bottom flask. After 16 h at rt, the reaction was worked up according to general procedure C and purified via flash silica gel chromatography (0:100 to 15:85 acetone:hexanes) to afford **S14** as a light tan solid (0.69 g, 71% yield).

Data for **S13** (Tabulated as a mixture of two conformers where $^1H$ and $^{13}C$ NMR spectra are provided for identification purposes):

$^1H$ NMR (400 MHz, $CDCl_3$, Note: Spectrum shows mixture of kinetically stable conformers at rt, please see spectrum for details. Useful diagnostic peaks given.**)** δ 7.95–7.89 (m), 6.50–6.44 (m), 6.30 (qq, *J* = 7.0, 1.4 Hz), 6.10 (qq, *J* = 7.0, 1.5 Hz), 4.99 (s), 4.93 (s), 1.92–1.90 (m), 1.89–1.87 (m).

$^{13}C$ NMR (151 MHz, $CDCl_3$, Note: Spectrum shows mixture of kinetically stable conformers at rt, please see spectrum for details. Useful diagnostic peaks given.**)** δ 173.6, 149.1, 147.9, 141.4, 140.5, 138.1, 134.5, 130.0, 127.0, 119.1, 107.9, 52.0, 49.2, 14.7, 13.1.

Data for **S14**:

$^1H$ NMR (600 MHz, $CDCl_3$) δ 11.32 (s, 1H), 7.77 (s, 1H), 7.60 (s, 1H), 7.50–7.43 (m, 2H), 7.31–7.25 (m, 1H), 7.15 (t, *J* = 8.1 Hz, 2H), 6.98 (d, *J* = 7.8 Hz, 1H), 6.96 (d, *J* = 8.1 Hz, 2H), 6.90 (t, *J* = 7.3 Hz, 1H), 6.34 (s, 1H), 5.68 (q, *J* = 6.9 Hz, 1H), 4.95 (s, 2H), 1.66 (s, 3H), 1.56 (d, *J* = 6.9 Hz, 3H).

$^{13}C$ NMR (151 MHz, $CDCl_3$) δ 176.5, 159.6, 156.1, 141.3, 140.5, 139.2, 131.9, 130.0, 129.5, 126.9, 124.9, 123.0, 118.1, 117.9, 113.3, 111.3, 107.8, 49.5, 13.9, 13.4.

FTIR ($cm^{-1}$) 3242 (br), 2923, 1724, 1661, 1449, 1201, 1151, 752.

HRMS (ESI) m/z, calculated for $[C_{22}H_{23}N_4O_3]^+$ ($[M+H]^+$): 391.1770, found: 391.1763.

MP = 95–99 °C.

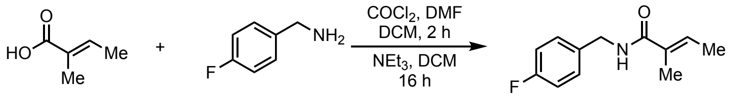


**S15**
Chemical Formula: $C_{12}H_{14}FNO$
Exact Mass: 207.1059
Molecular Weight: 207.2484

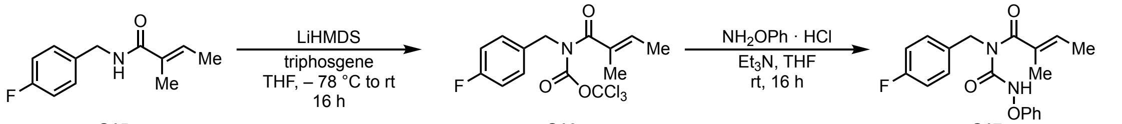


**S15**
Chemical Formula: $C_{12}H_{14}FNO$
Exact Mass: 207.1059
Molecular Weight: 207.2484

**S16**
Chemical Formula: $C_{14}H_{13}Cl_3FNO_3$
Exact Mass: 366.9945
Molecular Weight: 368.6104

**S17**
Chemical Formula: $C_{19}H_{19}FN_2O_3$
Exact Mass: 342.1380
Molecular Weight: 342.3704

(**S15**)**:** According to **General Procedure B**, tiglic acid (2.0 g, 20 mmol, 1 equiv), DMF (0.08 mL, 1 mmol, 0.05 equiv), oxalyl chloride (2.05 mL, 24 mmol, 1.2 equiv) and $CH_2Cl_2$ (40 mL, 0.5 M) were added to a 250 mL round bottom flask. After 2 h at rt, 4-fluorobenzyl amine (2.5 mL, 22 mmol, 1.1 equiv), $Et_3N$ (30 mmol, 4.18 mL) and $CH_2Cl_2$ (40 mL, 0.5 M) were added. After 16 h, **S15** was afforded as a dark yellow solid (4.10 g) and used without further purification. $^1H$ and $^{13}C$ NMR are provided for identification purposes only.

$^1H$ NMR (600 MHz, $CDCl_3$) δ 7.29–7.22 (m, 2H), 7.02–6.97 (m, 2H), 6.45 (qq, *J* = 7.0, 1.4 Hz, 1H), 6.06 (br s, 1H), 4.45 (d, *J* = 5.8 Hz, 2H), 1.85–1.83 (m, 3H), 1.74 (dq, *J* = 7.0, 1.1 Hz, 3H).

$^{13}C$ NMR (151 MHz, $CDCl_3$) δ 169.3, 163.1, 161.4, 131.7, 131.2, 129.6 (d, *J* = 8.0 Hz), 115.6 (d, *J* = 21.6 Hz), 43.1, 14.0, 12.5.

(**S17**) According to **General Procedure C**, **S15** (1.66 g, 8 mmol, 1 equiv), LiHMDS (8.8 mL, 8.8 mmol, 1.1 equiv), triphosgene (2.4 g, 8 mmol, 1 equiv) and THF (40 mL, 0.2 M) were added to a 250 mL round bottom flask. After 16 h at rt, the reaction was worked up according to general procedure C and purified using flash silica gel chromatography (0 100 to 10:90 ethyl acetate:hexanes) to provide **S16** as a 50:50 mixture of conformers that are kinetically stable at rt (1.6 g).

**S16** as a mixture of kinetically stable conformers (1.76 g, 5 mmol, 1 equiv), phenoxyamine hydrochloride (1.5 g, 10 mmol, 2 equiv), $Et_3N$ (2.1 mL, 15 mmol, 3 equiv), and THF (25 mL, 0.2 M) were then added to a 100 mL round bottom flask. After 16 h at rt, the reaction was worked up according to general procedure C and purified using flash silica gel chromatography (0:100 to 20:80 ethyl acetate:hexanes) to afford **S17** as a tan solid (1.07 g, 62% yield).

Data for **S16** (Tabulated as a mixture of two conformers where $^{1}H$ and $^{13}C$ NMR spectra are provided for identification purposes):

$^{1}H$ NMR (600 MHz, $CDCl_3$, Note: Spectrum shows mixture of kinetically stable conformers at rt, please see spectrum for details. Useful diagnostic peaks given.) δ 7.40–7.38 (m), 7.34–7.32 (m), 7.03–7.01 (m), 6.20 (q, *J* = 7.1 Hz), 6.01 (q, *J* = 7.0 Hz), 4.89 (s), 4.84 (s), 1.89 (s), 1.86 (s).

$^{13}C$ NMR (151 MHz, $CDCl_3$, Note: Spectrum shows mixture of kinetically stable conformers at rt, please see spectrum for details. Useful diagnostic peaks given.)) δ 173.6, 163.5, 148.9, 137.9, 134.6, 131.9, 130.7, 120.3, 115.8, 107.3, 51.6, 48.9, 14.7, 12.8.

Data for **S17**:

$^{1}H$ NMR (600 MHz, $CDCl_3$) δ 11.34 (s, 1H), 7.30 (t, *J* = 7.1 Hz, 2H), 7.19–7.15 (m, 2H), 7.05 (dd, *J* = 14.9, 7.7 Hz, 3H), 7.01 (t, *J* = 8.6 Hz, 2H), 5.77 (qt, *J* = 6.8, 1.7 Hz, 1H), 4.98 (s, 2H), 1.76 (s, 3H), 1.72 (dq, *J* = 6.8, 1.1 Hz, 3H).

$^{13}C$ NMR (151 MHz, CDCl3) δ 176.7, 163.1, 161.5, 159.7, 156.2, 133.3 (d, *J* = 3.3 Hz), 132.1, 129.9 (d, J = 8.2 Hz), 123.1, 115.8, 115.6, 113.4, 49.3, 13.9, 13.6.

$^{19}F$ NMR (565 MHz, $CDCl_3$) δ –114.61 (s).

FTIR ($cm^{-1}$) 3234 (br), 1724, 1661, 1591, 1510, 1223,1158, 752, 689, 495.

HRMS (ESI) m/z, calculated for $[C_{19}H_{20}FN_2O_3]^+$ ($[M+H]^+$): 343.1458, found: 343.1453.

MP = 89–93 °C.

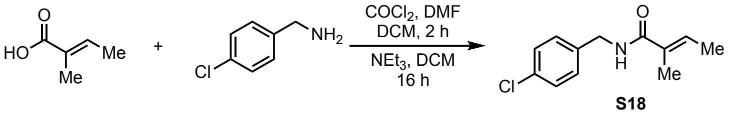

**S18**
Chemical Formula: $C_{12}H_{14}ClNO$
Exact Mass: 223.0764
Molecular Weight: 223.7000

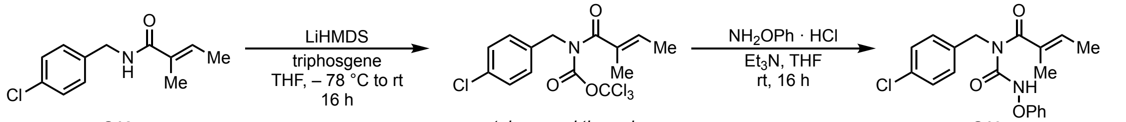

**S18**
Chemical Formula: $C_{12}H_{14}ClNO$
Exact Mass: 223.0764
Molecular Weight: 223.7000

*telescoped through without purification*

**S19**
Chemical Formula: $C_{19}H_{19}ClN_2O_3$
Exact Mass: 358.1084
Molecular Weight: 358.8220

(**S18**) According to **General Procedure B**, tiglic acid (2.0 g, 20 mmol, 1 equiv), DMF (0.08 mL, 1 mmol, 0.05 equiv), oxalyl chloride (2.18 mL, 24 mmol, 1.2 equiv), and $CH_2Cl_2$ (40 mL, 0.5 M) were added to a 250 mL round bottom flask. After 2 h at rt, 4-chlorobenzylamine (2.67 mL, 22 mmol, 1.1 equiv), $Et_3N$ (4.18 mL, 30 mmol, 1.5 equiv), and $CH_2Cl_2$ (40 mL, 0.5 M) were added. After 16 h at rt, **S18** was afforded as a dark yellow solid (3.7 g, 84% yield) and used without further purification.

$^1$H NMR (600 MHz, $CDCl_3$) δ 7.28–7.26 (m, 1H), 7.25 (d, $J$ = 2.3 Hz, 1H), 7.03–6.99 (m, 2H), 6.46 (qq, $J$ = 6.9, 1.5 Hz, 1H), 5.97 (br s, 1H), 4.46 (d, $J$ = 5.8 Hz, 2H), 1.87–1.85 (m, 3H), 1.77–1.75 (m, 3H).

$^{13}$C NMR (151 MHz, $CDCl_3$) δ 169.2, 163.0, 161.4, 134.4, 131.6, 131.1, 129.4, 43.2, 43.1, 14.0.

FTIR (cm$^{-1}$) 3297 (br), 3047, 2933, 1662, 1612, 1532, 1505, 834, 686, 573.

HRMS (ESI) m/z, calculated for $[C_{12}H_{15}ClNO]^+$ ($[M+H]^+$): 224.0842, found: 224.0834.

MP = 69–73 °C.

(**S19**) According to **General Procedure D**, **S18** (5 mmol, 1.12g), LiHMDS (5.5 mmol, 5.5 mL), triphosgene (5 mmol, 1.48g), and THF (37 mL) were added to a 250 mL round bottom flask. After 16 h at rt, phenoxyamine hydrochloride (4 mmol, 0.58 g) and $Et_3N$ (6 mmol, 0.84 mL) in THF (10 mL) were added. After 16 h at rt, the reaction was worked up according to general procedure D and purified using flash silica gel chromatography (0:100 to 15:85 ethyl acetate:hexanes) to afford **S19** as a light tan powder (0.34 g, 19% yield over two steps).

$^1$H NMR (600 MHz, $CDCl_3$) δ 11.35 (s, 1H), 7.34–7.27 (m, 4H), 7.17–7.10 (m, 2H), 7.09–7.01 (m, 3H), 5.77 (qq, $J$ = 6.8, 1.5 Hz, 1H), 4.98 (s, 2H), 1.76 (quint, $J$ = 1.2 Hz, 3H), 1.71 (dq, $J$ = 6.8, 1.2 Hz, 3H).

$^{13}$C NMR (151 MHz, $CDCl_3$) δ 176.6, 159.6, 156.1, 136.0, 133.6, 132.0, 129.9, 129.6, 129.0, 128.7, 123.2, 113.4, 49.3, 13.9, 13.6.

FTIR (cm$^{-1}$) 3240 (br), 1724, 1662, 1592, 1490, 1195, 752.

HRMS (ESI) m/z, calculated for $[C_{19}H_{20}ClN_2O_3]^+$ ($[M+H]^+$): 359.1163, found: 359.1155.

MP = 101–105 °C.

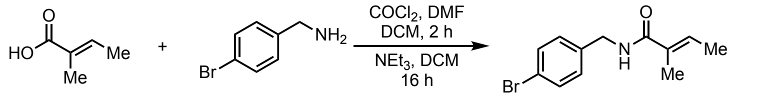


**S20**
Chemical Formula: $C_{12}H_{14}BrNO$
Exact Mass: 267.0259
Molecular Weight: 268.1540

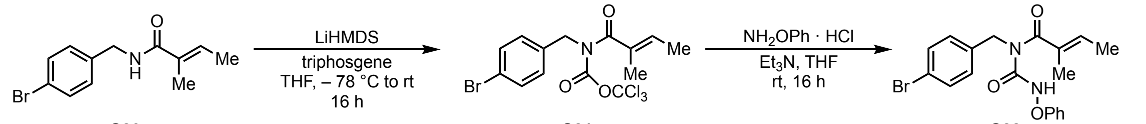


**S20**
Chemical Formula: $C_{12}H_{14}BrNO$
Exact Mass: 267.0259
Molecular Weight: 268.1540

**S21**
Chemical Formula: $C_{14}H_{13}BrCl_3NO_3$
Exact Mass: 426.9144
Molecular Weight: 429.5160

**S22**
Chemical Formula: $C_{19}H_{19}BrN_2O_3$
Exact Mass: 402.0579
Molecular Weight: 403.2760

(**S20**) According to **General Procedure B**, tiglic acid (4.0 g, 40 mmol, 1 equiv), DMF (0.15 mL, 2 mmol, 0.05 equiv), oxalyl chloride (4.12 mL, 48 mmol, 1.2 equiv), and $CH_2Cl_2$ (80 mL, 0.5 M) were added to a 250 mL round bottom flask at rt. After 2 h, 4-bromobenzylamine (5.6 mL, 44 mmol, 1.1 equiv), $Et_3N$ (8.36 mL, 60 mmol, 1.5 equiv) and $CH_2Cl_2$ (80 mL, 0.5 M) were added at rt. After 16 h **S20** was afforded as a dark yellow solid (10.4 g, 97% yield) and used without further purification.

$^1$H NMR (600 MHz, $CDCl_3$) δ 7.45 (d, $J$ = 8.4 Hz, 2H), 7.17 (d, $J$ = 8.3 Hz, 2H), 6.47 (qq, $J$ = 7.0, 1.4 Hz, 1H), 5.98 (br s, 1H), 4.45 (d, $J$ = 5.9 Hz, 2H), 1.86 (m, 3H), 1.76 (dq, $J$ = 7.0, 1.2 Hz, 3H).

$^{13}$C NMR (151 MHz, CDCl3) δ 169.3, 137.8, 131.9, 131.7, 131.4, 129.6, 121.5, 43.3, 14.1, 12.6.

FTIR (cm$^{-1}$) 3299 (br), 1653, 1616, 1532, 1229, 1012, 668.

HRMS (ESI) m/z, calculated for $[C_{12}H_{15}BrNO]^+$ ($[M+H]^+$): 268.0337, found: 268.0333.

MP = 76–79 °C.

(**S22**) According to **General Procedure C**, **S20** (10 mmol, 2.7 g), LiHMDS (11 mmol, 11 mL), triphosgene (10 mmol, 3.0 g), and THF (50 mL) were added to a 200 mL round bottom flask at rt. After 16 h, the reaction was worked up according to general procedure C and purified using flash silica gel chromatography (0:100 to 10:90 ethyl acetate:hexanes) to provide **S21** as a 43:57 mixture of conformers that are kinetically stable at rt (2.9 g).

**S21** (5 mmol, 2.15 g), as a mixture of kinetically stable conformers, phenoxyamine hydrochloride (10 mmol, 1.5 g), $Et_3N$ (15 mmol, 2.1 mL), and THF (25 mL) were added to a 100 mL round bottom flask at rt. After 16 h, the reaction was worked up according to general procedure C and purified using flash silica gel chromatography (5:95 to 10:90 ethyl acetate:hexanes) to afford **S22** as a reddish-white solid (1.12 g, 56% yield).

Data for **S21** (Tabulated as a mixture of two conformers where $^1$H and $^{13}$C NMR spectra are provided for identification purposes):

$^1$H NMR (600 MHz, $CDCl_3$, Note: Spectrum shows mixture of kinetically stable conformers at rt, please see spectrum for details. Useful diagnostic peaks given.) δ 6.22 (qq, $J$ = 7.0, 1.4 Hz), 6.03 (qq, $J$ = 6.9, 1.4 Hz), 4.87 (s), 4.82 (s).

$^{13}$C NMR (151 MHz, $CDCl_3$, Note: Spectrum shows mixture of kinetically stable conformers at rt, please see spectrum for details. Useful diagnostic peaks given.) δ 173.6, 137.9, 134.3, 130.4, 122.5, 51.6, 49.0, 14.7, 13.1.

Data for **S22**:

$^{1}$H NMR (600 MHz, CDCl3) δ 11.34 (s, 1H), 7.46–7.44 (m, 2H), 7.30 (ddt, *J* = 7.2, 4.3, 2.3 Hz, 2H), 7.06 (dddd, *J* = 11.3, 8.4, 6.1, 1.5 Hz, 5H), 5.77 (qq, *J* = 6.9, 1.5 Hz, 1H), 4.96 (s, 2H), 1.77–1.75 (m, 3H), 1.71 (dq, *J* = 6.8, 1.2 Hz, 3H).

$^{13}$C NMR (151 MHz, CDCl3) δ 176.6, 159.6, 156.1, 136.6, 132.1, 132.0, 129.9, 129.6, 129.0, 123.2, 121.7, 113.4, 49.4, 14.0, 13.6.

FTIR (cm$^{-1}$) 3238 (br), 1723, 1662, 1591, 1488, 1457, 1348, 1150, 752, 688.

HRMS (ESI) m/z, calculated for $[C_{19}H_{20}BrN_2O_3]^+$ ($[M+H]^+$): 403.0657, found: 403.0656.

MP = 107–109 °C.

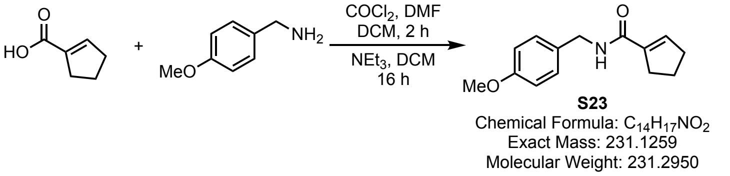


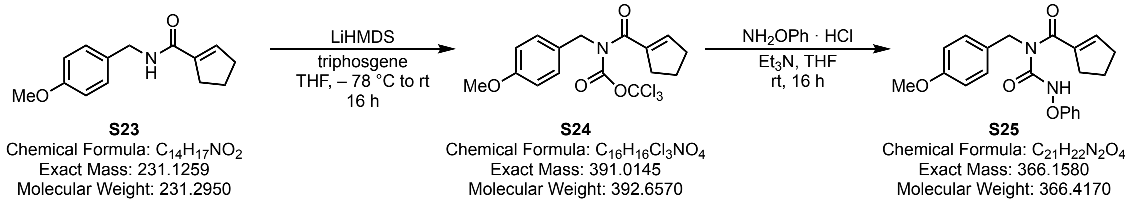


(**S23**) According to **General Procedure B**, 1-cyclopentene-1-carboxylic acid (2.2 g, 20 mmol, 1 equiv), DMF (0.08 mL, 1 mmol, 0.05 equiv), oxalyl chloride (2.18 mL, 24 mmol, 1.2 equiv), and $CH_2Cl_2$ (40 mL, 0.5 M) were added to a 250 mL round bottom flask at rt. After 2 h, 4-methoxybenzyl amine (2.9 mL, 22 mmol, 1.1 equiv), $Et_3N$ (4.18 mL, 30 mmol, 1.5 equiv), and $CH_2Cl_2$ (40 mL, 0.5 M) were added at rt. After 16 h, **S23** was obtained as a tan solid and used without further purification (4.5 g, 95% yield).

$^{1}$H NMR (600 MHz, $CDCl_3$) δ 7.23 (d, *J* = 8.7 Hz, 2H), 6.87 (d, *J* = 8.7 Hz, 2H), 6.55 (quint, *J* = 2.0 Hz, 1H), 5.82 (br s, 1H), 4.44 (d, *J* = 5.7 Hz, 2H), 3.80 (s, 3H), 2.57–2.51 (m, 2H), 2.48 (tq, *J* = 7.6, 2.6 Hz, 2H), 2.01–1.94 (m, 2H).

$^{13}$C NMR (151 MHz, $CDCl_3$) δ 165.4, 159.2, 139.2, 138.5, 130.7, 129.5, 114.3, 55.5, 43.1, 33.3, 31.7, 23.5.

FTIR (cm$^{-1}$) 3307 (br), 2961, 1642, 1603, 1532, 1252, 1029, 814.

HRMS (ESI) m/z, calculated for $[C_{14}H_{18}NO_2]^+$ ($[M+H]^+$): 232.1338, found: 232.1334.

MP = 104–106 °C.

(**S25**) According to **General Procedure C**, **S23** (0.93 g, 4 mmol, 1 equiv), LiHMDS (4.4 mL, 4.4 mmol, 1.1 equiv), triphosgene (1.19 mL, 4 mmol, 1 equiv), and THF (20 mL, 0.2 M) were added to a 250 mL round bottom flask at rt. After 16 h, the reaction was worked up according to general procedure C and purified using flash silica gel chromatography (0:100 to 15:85 ethyl acetate:hexanes) to provide **S24** as a 50:50 mixture of conformers that are kinetically stable at rt (0.8 g).

**S24** (0.80 g, 2.1 mmol, 1 equiv), as a mixture of kinetically stable conformers, phenoxyamine hydrochloride (0.61 g, 4.2 mmol, 2 equiv), $Et_3N$ (0.88 mL, 6.3 mmol, 3 equiv) and THF (11 mL, 0.2 M) were added to a 250 mL round bottom flask was added at rt. After 16 h, the reaction was worked up according to general procedure C and purified using flash silica gel chromatography (0:100 to 15:85 acetone:hexanes) to afford **S25** as a light tan powder (0.54 g, 70% yield).

Data for **S24** (Tabulated as a mixture of two conformers where $^1$H and $^{13}$C NMR spectra are provided for identification purposes):

$^1$H NMR (600 MHz, $CDCl_3$, Note: Spectrum shows mixture of kinetically stable conformers at rt, please see spectrum for details. Useful diagnostic peaks given.) δ 6.39 (tt, $J$ = 4.5, 1.9 Hz), 6.25 (tt, $J$ = 4.5, 1.9 Hz), 4.89 (s), 4.82 (s), 2.65 (ddt, $J$ = 10.1, 6.8, 2.3 Hz), 2.60 (ddt, $J$ = 10.1, 6.8, 2.3 Hz).

$^{13}$C NMR (151 MHz, $CDCl_3$, Note: Spectrum shows mixture of kinetically stable conformers at rt, please see spectrum for details. Useful diagnostic peaks given.) δ 169.6, 159.5, 149.0, 147.9, 145.0, 141.5, 140.9, 140.8, 128.4, 127.9, 114.2, 55.4, 51.7, 49.1, 33.8, 33.6, 32.3, 31.8, 23.2, 23.2.

Data for **S25**:

$^1$H NMR (600 MHz, $CDCl_3$) δ 11.55 (s, 1H), 7.32–7.28 (m, 2H), 7.13–7.10 (m, 2H), 7.10–7.06 (m, 2H), 7.03 (tt, $J$ = 7.3, 1.0 Hz, 1H), 6.86 (d, $J$ = 2.0 Hz, 2H), 6.02–6.00 (m, 1H), 5.02 (s, 2H), 3.80 (s, 3H), 2.52 (ddt, $J$ = 9.5, 5.2, 2.4 Hz, 2H), 2.50–2.48 (m, 2H), 1.92 (quint, $J$ = 7.6 Hz, 2H).

$^{13}$C NMR (151 MHz, $CDCl_3$) δ 173.4, 159.7, 159.1, 156.0, 138.2, 136.2, 129.7, 129.6, 128.3, 123.0, 114.2, 113.4, 55.4, 49.0, 34.6, 33.8, 22.9.

FTIR (cm$^{-1}$) 3243 (br), 2955, 2837, 1724, 1657, 1513, 1248, 1179.

HRMS (ESI) m/z, calculated for $[C_{21}H_{23}N_2O_4]^+$ ($[M+H]^+$): 367.1658, found: 367.1648.

MP = 84–87 °C.

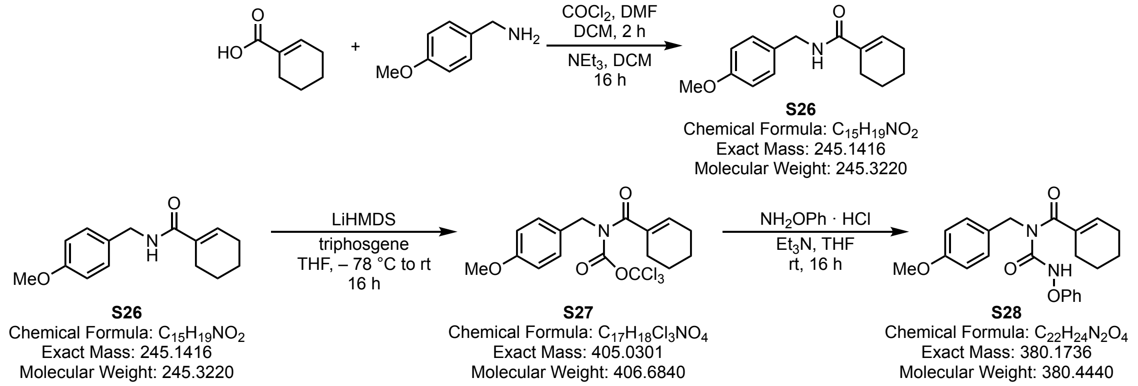


(**S26**) According to **General Procedure B**, 1-cyclohexene-1-carboxylic acid (3.2 g, 20 mmol, 1 equiv), DMF (0.08 mL, 1 mmol, 0.05 equiv), oxalyl chloride (2.18 mL, 24 mmol, 1.2 equiv), and $CH_2Cl_2$ (40 mL, 0.5 M) were added to a 250 mL round bottom flask at rt. After 2 h, 4-methoxybenzyl amine (2.9 mL, 22 mmol, 1.1 equiv), $Et_3N$ (4.18 mL, 30 mmol, 1.5 equiv), and $CH_2Cl_2$ (40 mL, 0.5 M) were added at rt. After 16 h **S26** was afforded as a white solid and was used without purification (4.9 g, 99% yield).

**S26** is a known compound. The acquired spectra is in accord with published data.[20] Partial characterization is shown below.

$^1$H NMR (600 MHz, $CDCl_3$) δ 7.22 (d, $J$ = 8.7 Hz, 2H), 6.86 (d, $J$ = 8.7 Hz, 2H), 6.65 (quint, $J$ = 3.9 Hz, 1H), 5.86 (br s, 1H), 4.43 (d, $J$ = 5.6 Hz, 2H), 3.80 (s, 3H), 2.23 (tq, $J$ = 6.0, 2.3 Hz, 2H), 2.15 (tq, $J$ = 6.1, 2.7 Hz, 2H), 1.71–1.64 (m, 2H), 1.63–1.55 (m, 2H).

$^{13}$C NMR (151 MHz, $CDCl_3$) δ 168.5, 159.2, 133.9, 133.2, 130.8, 129.4, 114.4, 55.5, 43.3, 25.5, 24.5, 22.3, 21.7.

FTIR ($cm^{-1}$) 3317 (br), 2931, 2857, 1659, 1615, 1512, 1247, 1175, 1034, 822.

MP = 57–60 °C.

(**S28**) According to **General Procedure C**, **S26** (3.3 g, 13.5 mmol, 1 equiv), LiHMDS (14.9 mL, 14.9 mmol, 1.1 equiv), triphosgene (4.0 g, 13.5 mmol, 1 equiv), and THF (68 mL, 0.2 M) were added to a 250 mL round bottom flask at rt. After 16 h, the reaction was worked up according to general procedure C and purified using flash silica gel chromatography (0:100 to 15:85 ethyl acetate:hexanes) to provide **S27** as a 46:54 mixture of conformers that are kinetically stable at rt (4.0 g).

**S27** (4.0 g, 9.8 mmol, 1 equiv), as a mixture of kinetically stable conformers, phenoxyamine hydrochloride (2.9 g, 19.6 mmol, 2 equiv), $Et_3N$ (4.1 mL, 29.4 mmol, 3 equiv), and THF (50 mL, 0.2 M) were added to a 200 mL round bottom flask at rt. After 16 h, the reaction was worked up according to general procedure C and purified using flash silica gel chromatography (0:100 to 15:85 acetone:hexanes) to afford **S28** as a light tan solid (1.8 g, 47% yield).

Data for **S27** (Tabulated as a mixture of two conformers where $^1H$ and $^{13}C$ NMR spectra are provided for identification purposes):

$^1H$ NMR (600 MHz, $CDCl_3$, Note: Spectrum shows mixture of kinetically stable conformers at rt, please see spectrum for details. Useful diagnostic peaks given.) δ 6.34 (dq, *J* = 4.0, 2.0 Hz), 6.15 (dq, *J* = 3.9, 2.0 Hz), 4.86 (s), 4.81 (s), 2.32 (tt, *J* = 6.3, 2.2 Hz), 2.27 (tt, *J* = 6.4, 2.1 Hz), 2.17–2.13 (m), 2.13 – 2.11 (m).

$^{13}C$ NMR (151 MHz, $CDCl_3$, Note: Spectrum shows mixture of kinetically stable conformers at rt, please see spectrum for details. Useful diagnostic peaks given.) δ 173.2, 168.8, 159.2, 142.3, 140.4, 136.0, 134.9, 133.2, 129.4, 127.8, 114.3, 55.4, 51.9, 43.5, 26.1, 24.5, 22.2, 21.7, 21.4.

Data for **S28:**

$^1H$ NMR (600 MHz, $CDCl_3$) δ 11.43 (s, 1H), 7.32–7.28 (m, 2H), 7.15–7.11 (m, 2H), 7.10–7.06 (m, 2H), 7.04 (tt, *J* = 7.3, 1.1 Hz, 1H), 6.85 (dt, *J* = 8.9, 3.1 Hz, 2H), 5.92 (tt, *J* = 3.8, 1.8 Hz, 1H), 5.00 (s, 2H), 3.80 (s, 3H), 2.15–2.10 (m, 2H), 2.05–2.02 (m, 2H), 1.67–1.63 (m, 2H), 1.63–1.59 (m, 2H).

$^{13}C$ NMR (151 MHz, $CDCl_3$) δ 176.3, 159.7, 159.2, 156.4, 134.4, 131.1, 129.8, 129.6, 128.8, 123.0, 114.2, 113.4, 55.5, 49.4, 25.6, 24.8, 21.9, 21.4.

FTIR ($cm^{-1}$) 3244 (br), 2934, 2859, 1722, 1660, 1513, 1248, 1187, 1032, 752, 689.

HRMS (ESI) m/z, calculated for $[C_{22}H_{25}N_2O_4]^+$ ($[M+H]^+$): 381.1815, found: 381.1807.

MP = 83–87 °C.

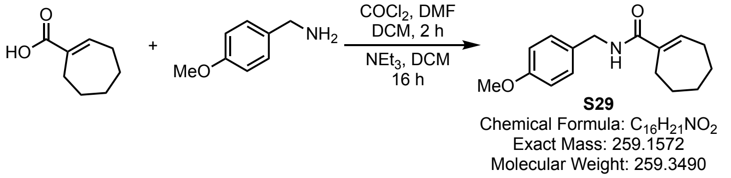


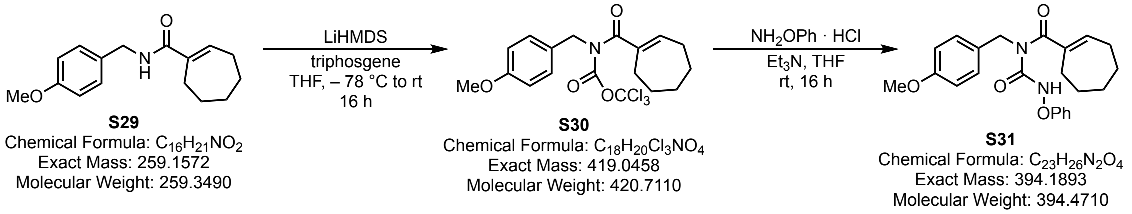


(**S29**) According to **General Procedure B**, cyclo-1-heptene carboxylic acid (1.12 g, 8 mmol, 1 equiv), DMF (0.03mL, 0.04 mmol, 0.05 equiv), oxalyl chloride (0.82 mL, 9.6 mmol, 1.2 equiv), and $CH_2Cl_2$ (16 mL, 0.5 M) were added to a 250 mL round bottom flask at rt. After 2 h, 4-methoxybenzyl amine (1.14 mL, 8.8 mmol, 1.1 equiv), $Et_3N$ (1.67 mL, 12 mmol, 1.5 equiv), and $CH_2Cl_2$ (16 mL, 0.5 M) were added at rt. After 16 h, the crude material was purified via flash silica gel chromatography (20:80 ethyl acetate:hexanes) to afford **S29** as a thick dark brown oil (2.06 g, 99% yield).

$^1$H NMR (600 MHz, $CDCl_3$) *δ* 7.22 (d, *J* = 8.2 Hz, 2H), 6.86 (d, *J* = 8.2 Hz, 2H), 6.54 (t, *J* = 6.5 Hz, 1H), 5.91 (br s, 1H), 4.40 (d, *J* = 5.6 Hz, 2H), 3.79 (s, 3H), 2.49–2.44 (m, 2H), 2.23 (q, *J* = 6.2 Hz, 2H), 1.76 (quint, *J* = 6.0 Hz, 2H), 1.54 (dquint, *J* = 20.5, 5.9 Hz, 4H).

$^{13}$C NMR (600 MHz, $CDCl_3$) δ 170.3, 159.2, 141.3, 136.5, 130.8, 129.4, 114.2, 55.4, 43.5, 32.1, 28.66, 28.62, 26.6, 26.2.

FTIR (cm$^{-1}$) 3280 (br), 2923, 2849, 1653, 1612, 1513, 1441, 1300, 1248, 1176, 1033, 818.

HRMS (ESI) m/z, calculated for $[C_{16}H_{22}NO_2]^+$ ($[M+H]^+$): 260.1651, found: 260.1645.

(**S31**) According to **General Procedure C**, **S29** (1.3 g, 5 mmol, 1 equiv), LiHMDS (5.5 mL, 5.5 mmol, 1.1 equiv), triphosgene (1.5 g, 5 mmol, 1 equiv), and THF (25 mL, 0.2 M) were added to a 100 mL round bottom flask at rt. After 16 h, the reaction was worked up according to general procedure C and purified using flash silica gel chromatography (0:100 to 10:90 ethyl acetate:hexanes) to provide **S30** as a 31:69 mixture of conformers that are kinetically stable at rt (1.2 g).

**S30** (0.8 g, 1.9 mmol, 1 equiv), as a mixture of kinetically stable conformers, phenoxyamine hydrochloride (0.55 g, 3.8 mmol, 2 equiv), $Et_3N$ (0.8 mL, 5.7 mmol, 3 equiv) , andTHF (9.5 mL, 0.2 M) were added to a 50 mL round bottom flask at rt. After 16 h, the reaction was worked up according to general procedure C and purified using flash silica gel chromatography (40:60 diethyl ether:hexanes) to give **S31** as a thick colorless oil (0.42 g, 57% yield).

Data for **S30** (Tabulated as a mixture of two conformers where $^1$H and $^{13}$C NMR spectra are provided for identification purposes):

$^1$H NMR (600 MHz, $CDCl_3$, Note: Spectrum shows mixture of kinetically stable conformers at rt, please see spectrum for details. Useful diagnostic peaks given.) δ 7.38–7.30 (m), 6.91–6.79 (m), 6.46 (t, *J* = 6.6 Hz), 6.37 (t, *J* = 6.5 Hz), 4.88 (s), 4.80 (s).

$^{13}$C NMR (151 MHz, $CDCl_3$, Note: Spectrum shows mixture of kinetically stable conformers at rt, please see spectrum for details. Useful diagnostic peaks given.) δ 174.1, 159.6, 148.0, 144.4, 141.6, 130.4, 114.1, 55.4, 51.9, 49.5, 31.9, 29.3, 29.0, 26.3, 25.6.

Data for **S31**:

$^{1}$H NMR (600 MHz, CDCl$_3$) δ 11.36 (s, 1H), 7.28 (dd, *J* = 8.8, 7.3 Hz, 2H), 7.13 (d, *J* = 8.7 Hz, 2H), 7.07–7.00 (m, 3H), 6.84 (dt, *J* = 5.4, 3.0 Hz, 2H), 6.13 (t, *J* = 6.3 Hz, 1H), 4.97 (s, 2H), 3.80 (s, 3H), 2.30–2.26 (m, 2H), 2.24 (q, *J* = 6.1 Hz, 2H), 1.78–1.75 (m, 2H), 1.59–1.55 (m, 4H).

$^{13}$C NMR (151 MHz, CDCl$_3$) δ 177.2, 159.7, 159.1, 156.3, 139.9, 136.9, 129.6, 129.6, 128.7, 123.0, 114.2, 113.4, 55.5, 49.4, 31.8, 30.8, 28.9, 26.9, 26.1.

FTIR (cm$^{-1}$) 3235 (br), 2925, 1722, 1657, 1513, 1445, 1248, 1181, 752.

HRMS (ESI) m/z, calculated for $[C_{23}H_{27}N_2O_4]^+$ ($[M+H]^+$): 395.1971, found: 395.1964.

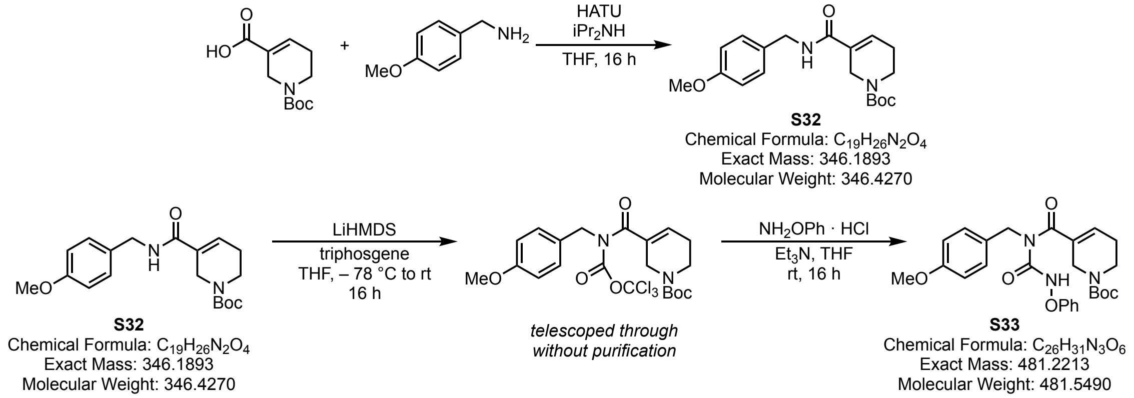


(**S32**) According to literature procedure,[21] **S32** was prepared as a light tan solid (2.9 g, 79% yield). Broad peaks are due to amide bond rotation, therefore, spectra are reported at 60 °C where the peaks coalesce.

$^{1}$H NMR (400 MHz, DMSO at 60 °C) δ 7.16 (d, *J* = 8.8 Hz, 2H), 6.86 (d, *J* = 8.8 Hz, 2H), 6.33 (br s, 1H), 5.76 (br s, 1H), 4.20 (d, *J* = 6.7 Hz, 2H), 3.73 (s, 3H), 3.41 (s, 1H), 2.93–2.79 (m, 1H), 2.71 (s, 2H), 1.98 (d, *J* = 14.7 Hz, 1H), 1.74 (td, *J* = 14.0, 5.7 Hz, 1H), 1.43 (s, 9H).

$^{13}$C NMR (101 MHz, DMSO at 60 °C) δ 167.8, 158.1, 153.0, 131.0, 128.2, 113.5, 79.2, 60.0, 58.3, 54.9, 41.3, 38.0, 28.6, 27.8, 24.2.

FTIR (cm$^{-1}$) 3357 (br), 2976, 1697, 1696, 1513, 1248, 1162, 1033.

HRMS (ESI) m/z, calculated for $[C_{19}H_{27}N_2O_4]^+$ ($[M+H]^+$): 347.1971, found: 347.1965.

MP = 134–136 °C.

(**S33**) According to **General Procedure D**, **S32** (1.7 g, 5 mmol, 1 equiv), LiHMDS (5.5 mL, 5.5 mmol, 1.1 equiv), triphosgene (1.48 g, 5 mmol, 1 equiv), and THF (25 mL, 0.2 M) were added to a 250 mL round bottom flask at rt. After 16 h, phenoxyamine hydrochloride (1.45 g, 10 mmol, 2 equiv), Et$_3$N (2.1 mL, 15 mmol, 3 equiv), and THF (25 mL, 0.2 M) were added at rt. After 16 h, the reaction was worked up according to general procedure D and purified using flash silica gel chromatography (0:100 to 15:85 acetone:hexanes) to afford **S33** as a tan flakey solid (1.3 g, 55% yield).

$^{1}$H NMR (600 MHz, DMSO) δ 12.03 (s, 1H), 7.26 (d, *J* = 8.7 Hz, 2H), 7.22 (t, *J* = 8.1 Hz, 2H), 7.01 (t, *J* = 7.2 Hz, 1H), 6.92 (d, *J* = 8.5 Hz, 2H), 6.76 (d, *J* = 8.1 Hz, 2H), 6.30 (dt, *J* = 4.5, 2.3 Hz, 1H), 4.70 (s, 2H), 4.14–4.06 (m, 2H), 3.75 (s, 3H), 2.52–2.50 (m, 2H), 2.17 (dt, *J* = 6.8, 3.2 Hz, 2H), 1.39 (s, 9H).

$^{13}$C NMR (101 MHz, DMSO) δ 169.1, 159.1, 158.7, 155.2, 153.8, 133.1, 130.3, 129.5, 129.4, 128.9, 128.5, 122.7, 113.8, 113.0, 79.1, 55.1, 47.8, 30.7, 28.0, 24.5.

FTIR (cm$^{-1}$) 3217 (br), 2974, 1695, 1668, 1613, 1513, 1247, 1162, 1031, 753.

HRMS (ESI) m/z, calculated for $[C_{26}H_{32}N_3O_6]^+$ ($[M+H]^+$): 482.2291, found: 482.2284.

MP = 57–60 °C.

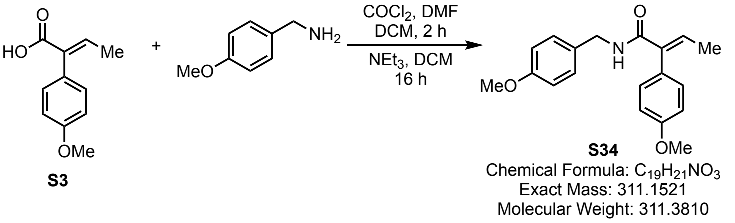


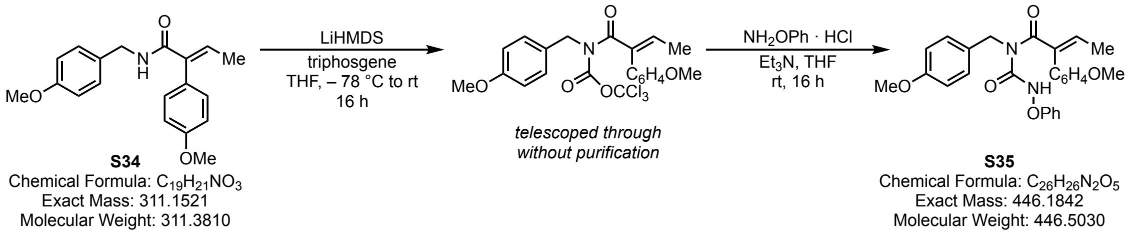


(**S34**) According to **General Procedure B**, **S3** (3.8 g, 20 mmol, 1 equiv), DMF (0.08 mL, 1 mmol, 0.05 equiv), oxalyl chloride (2.18 mL, 24 mmol, 1.2 equiv), and $CH_2Cl_2$ (40 mL, 0.5 M) were added to a 250 mL round bottom flask at rt. After 2 h, 4-methoxybenzyl amine (2.9 mL, 22 mmol, 1.1 equiv), $Et_3N$ (4.18 mL, 30 mmol, 1.5 equiv), and $CH_2Cl_2$ (40 mL, 0.5 M) were added at rt. After 16 h, the crude material was purified via flash silica chromatography (10:90 to 30:70 acetone:hexanes) to afford **S34** as a yellow solid (3.4 g, 55% yield).

$^1$H NMR (600 MHz, $CDCl_3$) δ 7.14 (d, *J* = 7.0 Hz, 1H), 7.13–7.11 (m, 2H), 7.10 (d, *J* = 2.2 Hz, 1H), 7.09 (d, *J* = 2.2 Hz, 1H), 6.92 (d, *J* = 8.7 Hz, 2H), 6.82 (d, *J* = 8.7 Hz, 2H), 5.64 (br s, 1H), 4.40 (d, *J* = 5.8 Hz, 2H), 3.82 (s, 3H), 3.77 (s, 3H), 1.65 (d, *J* = 7.2 Hz, 3H).

$^{13}$C NMR (151 MHz, $CDCl_3$) δ 167.1, 159.4, 159.0, 136.5, 136.3, 131.2, 131.1, 130.7, 128.9, 127.5, 114.5, 114.1, 55.4, 43.4, 15.3.

FTIR (cm$^{-1}$) 3291 (br), 2835, 1661, 1512, 1463, 1176, 832.

HRMS (ESI) m/z, calculated for $[C_{19}H_{22}NO_3]^+$ ($[M+H]^+$): 312.1600, found: 312.1598.

MP = 111–113 °C.

(**S35**) According to **General Procedure D**, **S34** (1.6 g, 5 mmol, 1 equiv), LiHMDS (5.5 mL, 5.5 mmol, 1.1 equiv), triphosgene (1.48 g, 5 mmol, 1 equiv), and THF (25 mL, 0.2 M) were added to a 250 mL round bottom flask at rt. After 16 h, phenoxyamine hydrochloride (1.45 g, 10 mmol, 2 equiv), $Et_3N$ (2.1 mL, 15 mmol, 3 equiv), and THF (25 mL, 0.2 M) were added at rt. After 16 h, the reaction was worked up according to general procedure D and purified using flash silica gel chromatography (0:100 to 15:85 acetone:hexanes) to afford **S35** as a dark red solid (0.34 g, 36% yield).

$^1$H NMR (600 MHz, $CDCl_3$) δ 11.57 (s, 1H), 7.32–7.27 (m, 2H), 7.25–7.22 (m, 2H), 7.06 (dd, *J* = 8.9, 1.1 Hz, 4H), 7.03 (tt, *J* = 7.3, 1.1 Hz, 1H), 6.94 (dt, *J* = 8.7, 2.9 Hz, 2H), 6.81 (dt, *J* = 8.4, 3.3 Hz, 2H), 5.87 (q, *J* = 7.1 Hz, 1H), 4.77 (s, 2H), 3.84 (s, 3H), 3.79 (s, 3H), 1.80 (d, *J* = 7.1 Hz, 3H).

$^{13}$C NMR (151 MHz, $CDCl_3$) δ 175.3, 159.7, 159.1, 156.1, 137.2, 134.9, 130.5, 130.0, 129.6, 129.6, 128.5, 125.7, 123.0, 114.5, 114.0, 113.4, 55.5, 55.4, 48.5, 14.8.

FTIR (cm$^{-1}$) 3252 (br), 2956, 2836, 1724, 1512, 1249, 1031, 831.

HRMS (ESI) m/z, calculated for $[C_{26}H_{27}N_2O_5]^+$ ($[M+H]^+$): 447.1920, found: 447.1928.

MP = 104–106 °C.

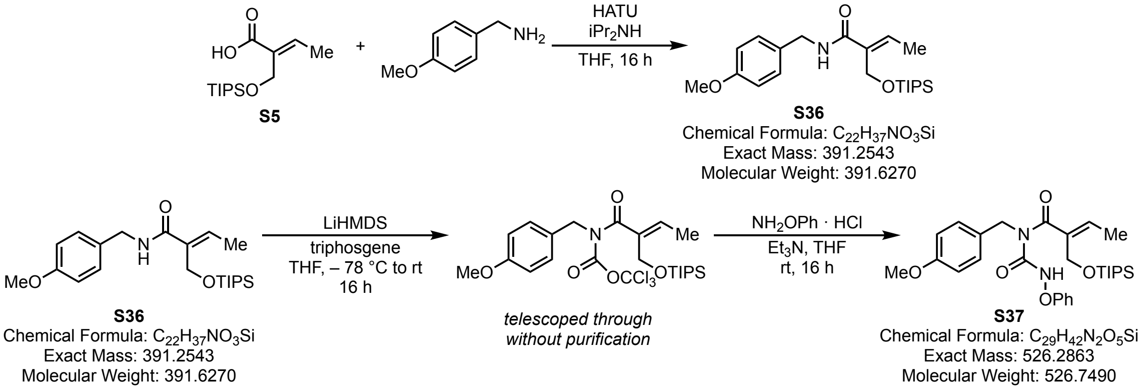


(**S36**) According to literature procedure,[21] **S36** was prepared as a light yellow oil in 85% purity (1.4 g). $^1$H and $^{13}$C NMR are provided for identification purposes only.

$^1$H NMR (600 MHz, $CDCl_3$) δ 7.61 (br s, 1H), 7.24–7.19 (m, 2H), 6.91–6.86 (m, 1H), 6.83 (dq, *J* = 9.5, 2.6 Hz, 2H), 4.47 (s, 2H), 4.43 (d, *J* = 5.5 Hz, 2H), 3.79 (s, 3H), 1.81 (d, *J* = 7.3 Hz, 3H), 1.08–1.01 (m, 3H), 0.99–0.96 (m, 18H).

$^{13}$C NMR (151 MHz, $CDCl_3$) δ 167.9, 159.1, 135.4, 132.8, 130.8, 129.5, 114.1, 67.2, 58.5, 55.5, 43.4, 18.0, 11.8.

(**S37**) According to **General Procedure D**, **S36** (1.2 g, 3.1 mmol, 1 equiv), LiHMDS (3.4 mL, 3.4 mmol, 1.1 equiv), triphosgene (0.9 g, 3.1 mmol, 1 equiv), and THF (12 mL, 0.2 M) were added to a 250 mL round bottom flask at rt. After 16 h, phenoxyamine hydrochloride (0.4 g, 6.2 mmol, 2 equiv), $Et_3N$ (0.8 mL, 9.3 mmol, 3 equiv), and THF (12 mL, 0.2 M) were added at rt. After 16 h, the reaction was worked up according to general procedure D and purified using flash silica gel chromatography (5:95 acetone:hexanes) to afford **S37** as a dark red oil (0.28 g, 25% yield).

$^1$H NMR (600 MHz, $CDCl_3$) δ 10.65 (s, 1H), 7.35–7.30 (m, 2H), 7.21–7.16 (m, 2H), 6.97 (appt tt, *J* = 7.3, 1.1 Hz, 1H), 6.87–6.83 (m, 2H), 6.75–6.71 (m, 2H), 5.87 (q, *J* = 7.1 Hz, 1H), 4.93 (s, 2H), 4.61 (s, 2H), 3.82 (s, 3H), 1.78 (d, *J* = 7.0 Hz, 3H), 1.08–1.03 (m, 3H), 1.00 (d, *J* = 4.9 Hz, 18H).

$^{13}$C NMR (151 MHz, $CDCl_3$) δ 171.5, 159.5, 159.4, 156.1, 136.3, 130.4, 129.8, 129.3, 129.3, 122.9, 114.0, 113.1, 59.5, 55.5, 48.0, 18.1, 13.6, 11.9.

FTIR ($cm^{-1}$) 3201 (br), 2945, 2866, 1736, 1673, 1513, 1249, 1030, 688.

HRMS (ESI) m/z, calculated for $[C_{29}H_{42}N_2O_5Si]^+$: 527.2942, found: 527.2937.

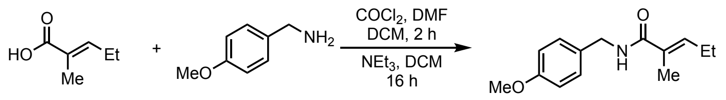

**S38**
Chemical Formula: $C_{14}H_{19}NO_2$
Exact Mass: 233.1416
Molecular Weight: 233.3110

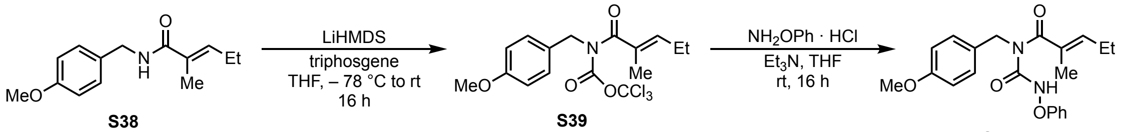

**S38**
Chemical Formula: $C_{14}H_{19}NO_2$
Exact Mass: 233.1416
Molecular Weight: 233.3110

**S39**
Chemical Formula: $C_{16}H_{18}Cl_3NO_4$
Exact Mass: 393.0301
Molecular Weight: 394.6730

**S40**
Chemical Formula: $C_{21}H_{24}N_2O_4$
Exact Mass: 368.1736
Molecular Weight: 368.4330

(**S38**) According to **General Procedure B**, (E)-2-methyl-2-pentenoic acid (2.2 g, 20 mmol, 1 equiv), DMF (0.08 mL, 1 mmol, 0.05 equiv), oxalyl chloride (2.18 mL, 24 mmol, 1.2 equiv), and $CH_2Cl_2$ (40 mL, 0.5 M) were added to a 250 mL round bottom flask at rt. After 2 h, 4-methoxybenzyl amine (2.9 mL, 22 mmol, 1.1 equiv), $Et_3N$ (4.18 mL, 30 mmol, 1.5 equiv), and $CH_2Cl_2$ (40 mL, 0.5 M) were added at rt. After 16 h **S38** was afforded as a white solid and was used without purification (4.3 g). Partial characterization appears below for identification purposes.

$^1$H NMR (600 MHz, $CDCl_3$) δ 7.25 – 7.21 (m, 2H), 6.90 – 6.85 (m, 2H), 6.33 (td, *J* = 7.3, 1.5 Hz, 1H), 5.91 (s, 1H), 4.43 (d, *J* = 5.5 Hz, 2H), 3.80 (s, 3H), 2.15 (quint, *J* = 7.5 Hz, 2H), 1.84 (s, 3H), 1.02 (t, *J* = 7.5 Hz, 3H).

$^{13}$C NMR (151 MHz, $CDCl_3$) δ 169.4, 159.2, 137.9, 130.7, 130.4, 129.4, 114.3, 55.5, 43.5, 21.8, 13.5, 12.7.

(**S40**) According to **General Procedure C**, **S38** (2.1 g, 9 mmol, 1 equiv), LiHMDS (9.9 mL, 9.9 mmol, 1.1 equiv), triphosgene (2.6 g, 9 mmol, 1 equiv), and THF (36 mL, 0.2 M) were added to a 100 mL round bottom flask at rt. After 16 h, the reaction was worked up according to general procedure C and purified using flash silica gel chromatography (0:100 to 15:85 ethyl acetate:hexanes) to provide **S39** as a 47:53 mixture of conformers that are kinetically stable at rt (2.1 g).

**S39** (2.1 g, 5.3 mmol, 1 equiv), as a mixture of kinetically stable conformers, phenoxyamine hydrochloride (1.5 g, 10.6 mmol, 2 equiv), $Et_3N$ (2.2 mL, 15.9 mmol, 3 equiv), and THF (27 mL, 0.2 M) were added to a 50 mL round bottom flask at rt. After 16 h, the reaction was worked up according to general procedure C and purified using flash silica gel chromatography (0:100 to 15:85 ethyl acetate:hexanes) to give **S40** as a light tan solid (0.90 g, 47% yield).

Data for **S39** (Tabulated as a mixture of two conformers where $^1$H and $^{13}$C NMR spectra are provided for identification purposes):

$^1$H NMR (600 MHz, $CDCl_3$, Note: Spectrum shows mixture of kinetically stable conformers at rt, please see spectrum for details. Useful diagnostic peaks given.) 7.35 – 7.31 (m), 6.04 (td, *J* = 7.4, 1.5 Hz), 5.89 (td, *J* = 7.2, 1.5 Hz), 4.87 (s), 4.81 (s), 1.88 (s), 1.84 (s), 0.98 (t, *J* = 7.6 Hz), 0.94 (t, *J* = 7.6 Hz).

$^{13}$C NMR (151 MHz, $CDCl_3$, Note: Spectrum shows mixture of kinetically stable conformers at rt, please see spectrum for details. Useful diagnostic peaks given.) δ 174.0, 159.5, 148.5, 147.7, 144.6, 140.7, 133.0, 130.3, 128.2, 127.6, 126.3, 121.1, 114.0, 77.0, 55.3, 51.8, 49.1, 22.1, 21.7, 13.1, 12.7.

Data for **S40**:

$^1$H NMR (600 MHz, $CDCl_3$) δ 11.38 (s, 1H), 7.32 – 7.28 (m, 2H), 7.14 – 7.09 (m, 2H), 7.09 – 7.01 (m, 3H), 6.87 – 6.83 (m, 2H), 5.69 (tq, $J$ = 7.3, 1.6 Hz, 1H), 4.98 (s, 2H), 3.79 (s, 3H), 2.12 (quint, $J$ = 7.4 Hz, 2H), 1.72 (s, 3H), 1.00 (t, $J$ = 7.5 Hz, 3H).

$^{13}$C NMR (151 MHz, $CDCl_3$) δ 176.9, 159.7, 159.2, 156.3, 136.3, 130.7, 129.6, 128.8, 123.0, 114.2, 113.4, 77.4, 55.5, 49.4, 21.3, 14.0, 13.1.

FTIR (cm$^{-1}$) 3243 (br), 2965, 2361, 1772, 1700, 1249, 1033, 776.

MP = 86–89 °C.

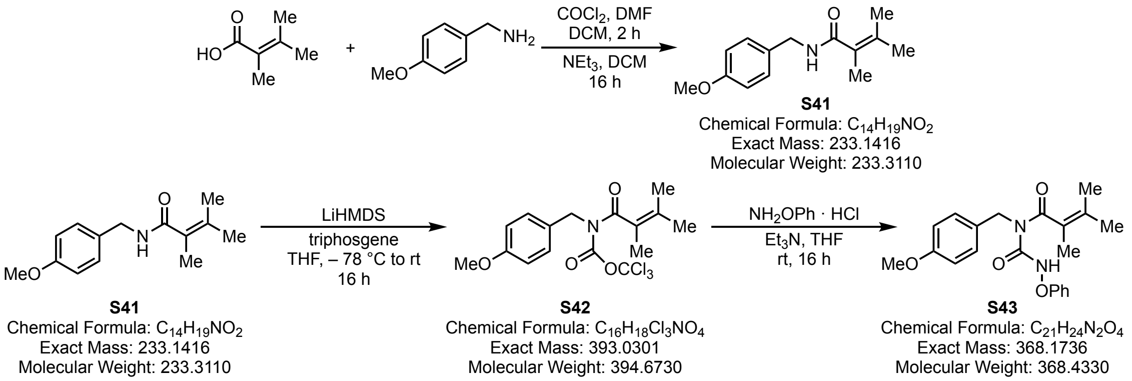


(**S41**) According to **General Procedure B**, 2,3-dimethyl-2-butenoic acid (2.5 g, 22 mmol, 1 equiv), DMF (0.17 mL, 2.2 mmol, 0.05 equiv), oxalyl chloride (2.12 mL, 24.2 mmol, 1.2 equiv), and $CH_2Cl_2$ (40 mL, 0.5 M) were added to a 250 mL round bottom flask at rt. After 2 h, 4-methoxybenzyl amine (3.5 mL, 26.4 mmol, 1.1 equiv), $Et_3N$ (4.6 mL, 33 mmol, 1.1 equiv), and $CH_2Cl_2$ (40 mL, 0.5 M) were added. After 16 h, the crude material was purified via flash silica gel chromatography (10:90 to 30:70 acetone:hexanes) to afford **S41** as a tan solid (1.5 g, 29% yield).

$^1$H NMR (600 MHz, $CDCl_3$) δ 7.23 (d, $J$ = 8.7 Hz, 2H), 6.87 (d, $J$ = 8.7 Hz, 2H), 5.60 (br s, 1H), 4.43 (d, $J$ = 5.7 Hz, 2H), 3.80 (s, 3H), 1.84 (s, 3H), 1.78 (s, 3H), 1.68 (s, 3H).

$^{13}$C NMR (101 MHz, $CDCl_3$) δ 172.4, 159.2, 132.6, 130.6, 127.2, 114.3, 55.4, 43.2, 31.7, 22.8, 20.2, 16.3.

FTIR (cm$^{-1}$) 3277 (br), 2916, 1627, 1512, 1246, 1034, 818.

HRMS (ESI) m/z, calculated for $[C_{14}H_{20}NO_2]^+$ ($[M+H]^+$): 234.1494, found: 234.1489.

MP = 57–59 °C.

(**S43**) According to **General Procedure C**, **S41** (1.4 g, 6 mmol, 1 equiv), LiHMDS (6.6 mL, 6.6 mmol, 1.1 equiv), triphosgene (1.8 g, 6 mmol, 1 equiv), and THF (30 mL, 0.2 M) were added to a 250 mL round bottom flask at rt. After 16 h, the reaction was worked up according to general procedure C and purified using flash silica gel chromatography (0:100 to 15:85 acetone:hexanes) to provide **S42** as a 43:57 mixture of conformers that are kinetically stable at rt (1.4 g).

**S42** (1.4 g, 3.5 mmol, 1 equiv), as a mixture of kinetically stable conformers, phenoxyamine hydrochloride (1.0 g, 7 mmol, 3 equiv), $Et_3N$ (1.5 mL, 10.5 mmol, 3 equiv), and THF (18 mL, 0.2 M) were then added to a 100 mL round bottom flask at rt. After 16 h, the reaction was worked up according to general procedure C and purified using flash silica gel chromatography (0:100 to 15:85 acetone:hexanes) to give **S43** as a tan powder (0.85 g, 66% yield).

Data for **S42** (Tabulated as a mixture of two conformers where $^1$H and $^{13}$C NMR spectra are provided for identification purposes):

$^{1}$H NMR (600 MHz, $CDCl_3$, Note: Spectrum shows mixture of kinetically stable conformers at rt, please see spectrum for details. Useful diagnostic peaks given.) δ 7.38–7.36 (m), 7.31–7.28 (m), 5.02 (s), 4.89 (s), 1.82–1.84 (m), 1.80–1.78 (m), 1.72–1.70 (m), 1.63–1.61 (m).

$^{13}$C NMR (151 MHz, $CDCl_3$ Note: Spectrum shows mixture of kinetically stable conformers at rt, please see spectrum for details. Useful diagnostic peaks given.) δ 173.4, 159.5, 149.4, 147.4, 136.8, 134.6, 130.5, 129.8, 128.4, 126.5, 114.1, 77.4, 76.9, 55.4, 50.4, 47.8, 22.0, 20.7, 16.1.

Data for **S43**:

$^{1}$H NMR (400 MHz, $CDCl_3$) δ 11.76 (s, 1H), 7.35–7.28 (m, 2H), 7.15–7.08 (m, 4H), 7.05 (tt, *J* = 7.2, 1.1 Hz, 1H), 6.83 (dt, *J* = 8.6, 3.0 Hz, 2H), 4.87 (s, 2H), 3.79 (s, 3H), 1.71 (s, 3H), 1.69 (s, 3H), 1.47 (appt s, 3H).

$^{13}$C NMR (101 MHz, $CDCl_3$) δ 177.4, 159.8, 159.2, 156.2, 133.3, 129.6, 129.5, 129.0, 125.4, 123.1, 114.0, 113.4, 55.4, 48.4, 22.2, 19.4, 16.1.

FTIR ($cm^{-1}$) 3239 (br), 2935, 1721, 1653, 1513, 1457, 1248, 1186, 1105, 1032, 753, 688.

HRMS (ESI) m/z, calculated for $[C_{21}H_{25}N_2O_4]^+$ ($[M+H]^+$): 369.1815, found: 369.1806.

MP = 99–101 °C.

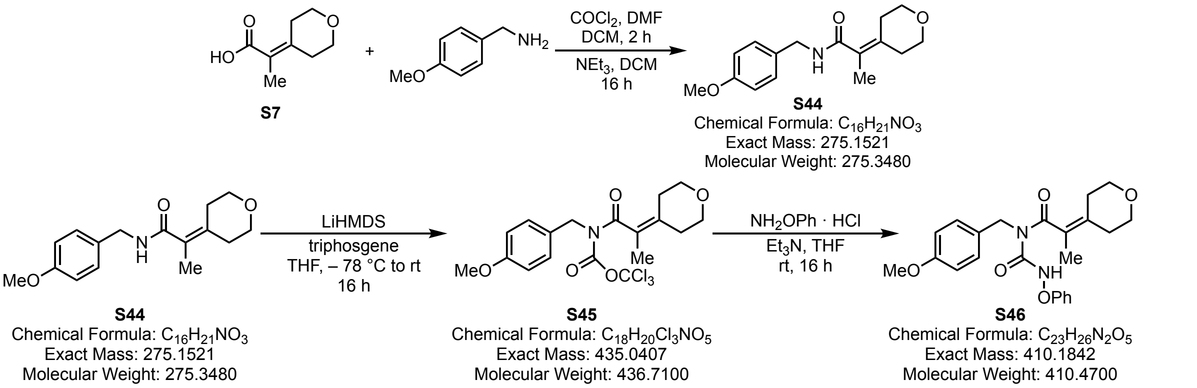


(**S44**) According to **General Procedure B**, **S7** (3.1 g, 20 mmol, 1 equiv), DMF (0.08 mL, 1 mmol, 0.05 equiv), oxalyl chloride (2.18 mL, 24 mmol, 1.2 equiv), and $CH_2Cl_2$ (40 mL, 0.2 M) were added to a 250 mL round bottom flask at rt. After 2 h, 4-methoxybenzyl amine (2.9 mL, 22 mmol, 1.1 equiv), $Et_3N$ (4.18 mL, 30 mmol, 1.5 equiv), and $CH_2Cl_2$ (40 mL, 0.2 M) were added. After 16 h, the crude material was purified via flash silica gel chromatography (30:70 acetone:hexanes) to afford **S44** as a white solid (2.31 g, 42% yield).

$^{1}$H NMR (400 MHz, $CDCl_3$) δ 7.22 (dt, *J* = 8.7, 2.4 Hz, 2H), 6.87 (dt, *J* = 8.8, 3.0 Hz, 2H), 5.61 (br s, 1H), 4.43 (d, *J* = 5.8 Hz, 2H), 3.80 (s, 3H), 3.69 (dt, *J* = 13.9, 5.5 Hz, 2H), 3.66 (t, *J* = 5.5 Hz, 2H), 2.38 (t, *J* = 6.1 Hz, 2H), 2.29 (t, *J* = 5.2 Hz, 2H), 1.87 (s, 3H).

$^{13}$C NMR (151 MHz, $CDCl_3$) δ 171.6, 159.3, 134.6, 130.3, 129.4, 126.1, 114.3, 68.9, 68.3, 55.5, 43.2, 32.8, 30.5, 15.5.

FTIR ($cm^{-1}$) 3276 (br), 2960, 2851, 1623, 1514, 1440, 1299, 1249.

HRMS (ESI) m/z, calculated for $[C_{16}H_{22}NO_3]^+$: 276.1600, found: 276.1595.

MP = 135–137 ºC

(**S46**) According to **General Procedure C**, **S44** (1.4 g, 5 mmol, 1 equiv), LiHMDS (5.5 mL, 5.5 mmol, 1.1 equiv), triphosgene (1.5 g, 5 mmol, 1 equiv), and THF (25 mL, 0.2 M) were added to a 200 mL round bottom flask at rt. After 16 h, the reaction was worked up according to general procedure C and purified using flash silica gel chromatography (0:100 to 15:85 acetone:hexanes) to provide **S45** as a 43:57 mixture of conformers that are kinetically stable at rt (1.03 g).

**S45** (0.96 g, 2.2 mmol, 1 equiv), as a mixture of kinetically stable conformers, phenoxyamine hydrochloride (0.64 g, 4.4 mmol, 2 equiv), $Et_3N$ (0.92 mL, 6.6 mmol, 3 equiv), and THF (11 mL, 0.2 M) were added to a 50 mL round bottom flask at rt. After 16 h, the reaction was worked up according to general procedure C and purified using flash silica gel chromatography (10:90 acetone:hexanes) to afford **S46** as a tan solid (0.60 g, 67% yield).

Data for **S45**:

$^1$H NMR (600 MHz, $CDCl_3$, Note: Spectrum shows mixture of kinetically stable conformers at rt, please see spectrum for details. Useful diagnostic peaks given.) δ 5.04 (s), 4.89 (s), 2.63 (dt, *J* = 5.6, 1.2 Hz), 2.40 (t, *J* = 5.5 Hz), 2.32–2.29 (m), 2.20 (t, *J* = 5.2 Hz), 2.11 (t, *J* = 5.6 Hz), 1.91 (s), 1.85 (s), 1.81 (s).

$^{13}$C NMR (151 MHz, $CDCl_3$, Note: Spectrum shows mixture of kinetically stable conformers at rt, please see spectrum for details. Useful diagnostic peaks given.) δ 172.9, 159.6, 153.4, 149.7, 147.3, 137.2, 135.6, 130.5, 129.8, 128.2, 125.4, 125.0, 119.3, 114.2, 113.8, 102.4, 68.5, 68.2, 67.9, 67.7, 55.4, 50.2, 47.8, 35.1, 32.7, 32.4, 31.0, 30.7, 30.4, 15.4,14.9.

Data for **S46**:

$^1$H NMR (600 MHz, $CDCl_3$) δ 11.72 (s, 1H), 7.35–7.29 (m, 2H), 7.14–7.09 (m, 4H), 7.06 (tt, *J* = 7.3, 1.1 Hz, 1H), 6.87–6.82 (m, 2H), 5.08–4.71 (m, 2H), 3.79 (s, 3H), 3.78–3.58 (m, 2H), 3.49 (s, 2H), 2.38–2.19 (m, 2H), 2.09 (s, 1H), 1.85 (d, *J* = 16.4 Hz, 1H), 1.76 (s, 3H).

$^{13}$C NMR (151 MHz, $CDCl_3$) δ 176.6, 159.7, 159.3, 156.0, 135.3, 129.6, 129.3, 128.7, 124.1, 123.2, 114.2, 113.4, 67.9, 67.9, 55.5, 48.4, 32.8, 29.8, 15.1.

FTIR (cm$^{-1}$) 3242 (br), 2959, 2848, 2361, 2337, 1719, 1653, 1612, 1591, 1514, 1488, 1457.

HRMS (ESI) m/z, calculated for $[C_{23}H_{27}N_2O_5]^+$ ($[M+H]^+$): 411.1920, found: 411.1918.

MP = 109–112 °C.

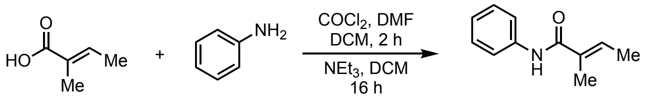


**S47**
Chemical Formula: $C_{11}H_{13}NO$
Exact Mass: 175.0997
Molecular Weight: 175.2310

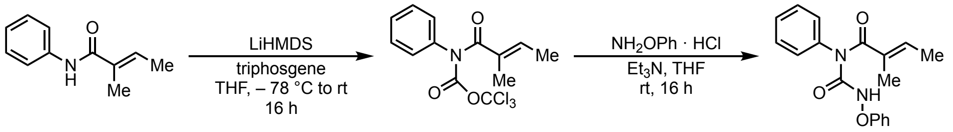


**S47**
Chemical Formula: $C_{11}H_{13}NO$
Exact Mass: 175.0997
Molecular Weight: 175.2310

*telescoped through without purification*

**S48**
Chemical Formula: $C_{18}H_{18}N_2O_3$
Exact Mass: 310.1317
Molecular Weight: 310.3530

(**S47**) According to **General Procedure B**, tiglic acid (2.0 g, 20 mmol, 1 equiv), DMF (0.08 mL, 1 mmol, 0.05 equiv), oxalyl chloride (2.07 mL, 24 mmol, 1.2 equiv), and $CH_2Cl_2$ (40 mL, 0.5 M) were added to a 250 mL round bottom flask at rt. After 2 h, aniline (2.0 mL, 22 mmol, 1.1 equiv), $Et_3N$ (4.18 mL, 30 mmol, 1.5 equiv), and $CH_2Cl_2$ (40 mL, 0.5 M) were added. After 16 h, the crude material was purified via flash silica gel chromatography (10:90 to 40:60 ethyl acetate:hexanes) to afford **S47** as a white solid (2.69 g, 77% yield).

$^1$H NMR (600 MHz, $CDCl_3$) δ 7.55 (d, *J* = 8.6 Hz, 2H), 7.41 (br s, 1H), 7.36–7.30 (m, 2H), 7.14–7.08 (m, 1H), 6.52 (qq, *J* = 6.9, 1.5 Hz, 1H), 1.95 (quint, *J* = 1.2 Hz, 3H), 1.82 (dq, *J* = 6.9, 1.1 Hz, 3H).

$^{13}$C NMR (151 MHz, $CDCl_3$) δ 132.9, 131.2, 129.3, 129.0, 124.2, 119.9, 119.6, 14.1, 12.6.

FTIR (cm$^{-1}$) 3284 (br), 1662, 1636, 1596, 1536, 1490, 1441, 1321, 758.

HRMS (ESI) m/z, calculated for $[C_{11}H_{14}NO]^+$ ($[M+H]^+$): 176.1075, found: 176.1072.

MP = 72–75 °C.

(**S48**) According to **General Procedure D**, **S47** (0.9 g, 5 mmol, 1 equiv), LiHMDS (5.5 mL, 5.5 mmol, 1.1 mmol), triphosgene (1.48 g, 5 mmol, 1 equiv), and THF (25 mL, 0.2 M) were added to a 250 mL round bottom flask at rt. After 16 h, phenoxyamine hydrochloride (1.5 g, 10 mmol, 2 equiv), $Et_3N$ (2.1 mL, 15 mmol, 3 equiv), and THF (25 mL, 0.2 M) were added. After 16 h, the reaction was worked up according to general procedure D and purified using flash silica gel chromatography (0:100 to 15:85 ethyl acetate:hexanes) to afford **S48** as a dark brown solid (0.13 g, 41% yield).

$^1$H NMR (600 MHz, $CDCl_3$) δ 11.41 (s, 1H), 7.39–7.35 (m, 2H), 7.35–7.32 (m, 1H), 7.32–7.29 (m, 2H), 7.21–7.18 (m, 2H), 7.16–7.13 (m, 2H), 7.04 (tt, *J* = 7.4, 1.1 Hz, 1H), 5.94 (qq, *J* = 6.9, 1.3 Hz, 1H), 1.51 (dq, *J* = 6.8, 1.1 Hz, 3H), 1.50 (quint, *J* = 1.1 Hz, 3H).

$^{13}$C NMR (151 MHz, $CDCl_3$) δ 175.5, 159.7, 155.7, 137.7, 133.7, 132.4, 129.5, 129.4, 128.9, 128.4, 123.0, 113.6, 13.7, 13.5.

FTIR (cm$^{-1}$) 3246 (br), 3036, 2923, 1733, 1667, 1262, 1152, 891, 735.

HRMS (ESI) m/z, calculated for $[C_{18}H_{19}N_2O_3]^+$ ($[M+H]^+$): 311.1396, found: 311.1387.

MP = 109–112 °C.

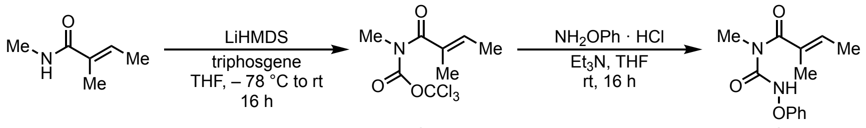

S49
Chemical Formula: $C_8H_{10}Cl_3NO_3$
Exact Mass: 272.9726
Molecular Weight: 274.5220

S50
Chemical Formula: $C_{13}H_{16}N_2O_3$
Exact Mass: 248.1161
Molecular Weight: 248.2820

(**S50**) According to **General Procedure C**, (*E*)-*N*-methyl-2-methyl-2-butenamide (0.8 g, 7 mmol, 1 equiv), LiHMDS (7.7 mL, 7.7 mmol, 1.1 equiv), triphosgene (2.1 g, 7 mmol, 1 equiv), and THF (25 mL, 0.2 M) were added to a 200 mL round bottom flask at rt. After 16 h, the reaction was worked up according to general procedure C and purified using flash silica gel chromatography (0:100 to 15:85 acetone:hexanes) to provide **S49** as a 45:55 mixture of conformers that are kinetically stable at rt (0.85 g).

**S49** (0.85 g, 3.1 mmol, 1 equiv), as a mixture of kinetically stable conformers, phenoxyamine hydrochloride (0.9 g, 6.2 mmol, 2 equiv), $Et_3N$ (1.3 mL, 9.3 mmol, 3 equiv), and THF (16 mL, 0.2 M) were added to a 100 mL round bottom flask at rt. After 16 h, the reaction was worked up according to general procedure C and purified using flash silica gel chromatography (0:100 to 15:85 ethyl acetate:hexanes) to afford **S50** as a colorless oil (0.42 g, 55% yield).

Data for **S49** (Tabulated as a mixture of two conformers where $^1$H and $^{13}$C NMR spectra are provided for identification purposes):

$^1$H NMR (600 MHz, $CDCl_3$, Note: Spectrum shows mixture of kinetically stable conformers at rt, please see spectrum for details. Useful diagnostic peaks given.) δ 6.39 (dq, *J* = 7.0, 1.4 Hz), 6.17 (dq, *J* = 7.0, 1.5 Hz), 3.29 (s), 3.24 (s), 1.92–1.90 (m), 1.88–1.86 (m), 1.83–1.80 (m), 1.80–1.77 (m).

$^{13}$C NMR (151 MHz, $CDCl_3$, Note: Spectrum shows mixture of kinetically stable conformers at rt, please see spectrum for details. Useful diagnostic peaks given.) δ 174.0, 137.6, 134.2, 130.3, 120.8, 116.6, 36.0, 33.1, 14.6, 13.1.

Data for **S50**:

$^1$H NMR (600 MHz, $CDCl_3$) δ 11.51 (s, 1H), 7.33–7.28 (m, 2H), 7.13–7.09 (m, 2H), 7.04 (tt, *J* = 7.4, 1.1 Hz, 1H), 5.93 (dq, *J* = 6.9, 1.6 Hz, 1H), 3.27 (s, 3H), 1.92–1.89 (m, 3H), 1.79–1.77 (m, 3H).

$^{13}$C NMR (151 MHz, $CDCl_3$) δ 176.4, 159.8, 156.3, 132.2, 130.4, 129.6, 123.0, 113.5, 34.7, 13.7, 13.6.

FTIR (cm$^{-1}$) 3253 (br), 2971, 2249, 1725, 1664, 1205, 755.

HRMS (ESI) m/z, calculated for $[C_{13}H_{17}N_2O_3]^+$ ($[M+H]^+$): 249.1239, found: 249.1233.

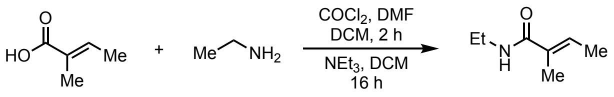


**S51**
Chemical Formula: $C_7H_{13}NO$
Exact Mass: 127.0997
Molecular Weight: 127.1870

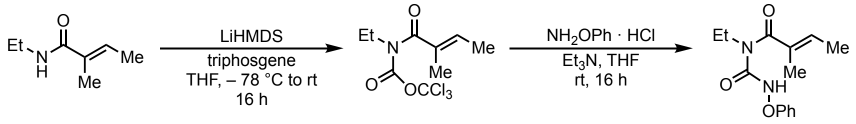


**S51**
Chemical Formula: $C_7H_{13}NO$
Exact Mass: 127.0997
Molecular Weight: 127.1870

**S52**
Chemical Formula: $C_9H_{12}Cl_3NO_3$
Exact Mass: 286.9883
Molecular Weight: 288.5490

**S53**
Chemical Formula: $C_{14}H_{18}N_2O_3$
Exact Mass: 262.1317
Molecular Weight: 262.3090

(**S51**) According to **General Procedure B**, tiglic acid (2.0 g, 20 mmol, 1 equiv), DMF (0.08 mL, 1 mmol, 0.05 equiv), oxalyl chloride (2.18 mL, 24 mmol, 1.2 equiv), and $CH_2Cl_2$ (40 mL, 0.5 M) were added to a 250 mL round bottom flask at rt. After 2 h, $Et_3N$ (4.18 mL, 30 mmol, 1.5 equiv), and $CH_2Cl_2$ (40 mL, 0.5 M) were added. An oven dried needle was then directly attached to a lecture bottle of ethyl amine gas, the needle was placed directly through the septum into the reaction solution. A vent needle was then placed in the septum, and the ethyl amine gas was bubbled through the reaction solution for 15 min. The vent needle was removed, followed by the ethyl amine needle, and the reaction was maintained at rt. After 16 h, the crude material was purified via flash silica gel chromatography (15:85 acetone:hexanes) to afford **S51** as a light green oil with 85% purity (1.2 g). Partial characterization appears below for identification purposes.

$^1H$ NMR (600 MHz, $CDCl_3$) δ 6.40 (qd, $J$ = 6.9, 1.5 Hz, 1H), 5.72 (br s, 1H), 3.33 (quint, $J$ = 13.6, 11 Hz, 2H), 1.82 (s, 3H), 1.73 (d, $J$ = 6.8 Hz, 3H), 1.15 (t, $J$ = 7.3 Hz, 3H).

$^{13}C$ NMR (151 MHz, $CDCl_3$) δ 169.4, 132.0, 130.4, 34.6, 15.0, 14.0, 12.5.

(**S53**) According to **General Procedure C**, **S51** (1.2 g, 9.4 mmol, 1 equiv), LiHMDS (10.4 mL, 10.4 mmol, 1.1 equiv), triphosgene (2.8 g, 9.4 mmol, 1 equiv), and THF (47 mL, 0.2 M) were added to a 200 mL round bottom flask at rt. After 16 h, the reaction was worked up according to general procedure C and purified using flash silica gel chromatography (0:100 to 15:85 acetone:hexanes) to provide **S52** as a 50:50 mixture of conformers that are kinetically stable at rt (1.7 g).

**S52** (1.7 g, 5.9 mmol, 1 equiv), as a mixture of kinetically stable conformers, phenoxyamine hydrochloride (1.7 g, 11.8 mmol, 2 equiv), $Et_3N$ (2.5 mL, 17.7 mmol, 3 equiv), and THF (30 mL, 0.2 M) were added to a 100 mL round bottom flask at rt. After 16 h, the reaction was worked up according to general procedure C and purified using flash silica gel chromatography (0:100 to 15:85 ethyl acetate:hexanes) to afford **S53** as a dark red oil (1.0 g, 70% yield).

Data for **S52** (Tabulated as a mixture of two conformers where $^1H$ and $^{13}C$ NMR spectra are provided for identification purposes):

$^1H$ NMR (600 MHz, $CDCl_3$, Note: Spectrum shows mixture of kinetically stable conformers at rt, please see spectrum for details. Useful diagnostic peaks given.) δ 6.38–6.35 (m), 6.20–6.08 (m), 1.92–1.89 (m), 1.88–1.86 (m), 1.83–1.81 (m), 1.80–1.77 (m).

$^{13}C$ NMR (151 MHz, $CDCl_3$, Note: Spectrum shows mixture of kinetically stable conformers at rt, please see spectrum for details. Useful diagnostic peaks given.) δ 173.8, 137.4, 134.2, 133.7, 116.8, 44.5, 43.9, 41.8, 40.7, 17.6, 14.6, 13.9, 13.1.

Data for **S53**:

$^1H$ NMR (600 MHz, $CDCl_3$) δ 11.43 (s, 1H), 7.31–7.29 (m, 2H), 7.11–7.09 (m, 2H), 7.04–7.02 (tt, $J$ = 7.4, 1.2 Hz, 1H), 5.85 (dq, $J$ = 8.5, 1.6 Hz, 1H), 3.80 (dq, $J$ = 7.0, 2.0 Hz, 2H), 1.92–1.89 (m, 3H), 1.79–1.76 (m, 3H), 1.24–1.21 (m, 3H).

$^{13}C$ NMR (151 MHz, $CDCl_3$) δ 176.7, 159.8, 155.8, 132.2, 129.5, 128.1, 123.0, 113.4, 42.1, 15.2, 14.1, 13.4.

FTIR ($cm^{-1}$) 3338 (br), 2972, 2249, 1727, 1662, 1412, 1202, 756.

HRMS (ESI) m/z, calculated for $[C_{14}H_{19}N_2O_3]^+$ ($[M+H]^+$): 263.1396, found: 263.1391.

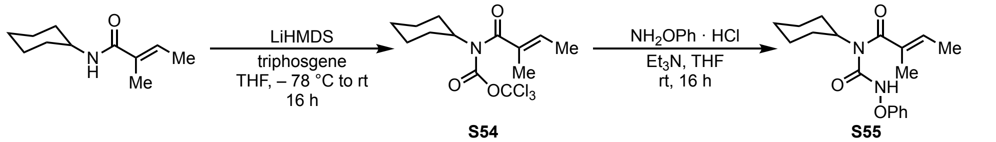


**S54**
Chemical Formula: $C_{13}H_{18}Cl_3NO_3$
Exact Mass: 341.0352
Molecular Weight: 342.6410

**S55**
Chemical Formula: $C_{18}H_{24}N_2O_3$
Exact Mass: 316.1787
Molecular Weight: 316.4010

(**S55**) According to **General Procedure C**, (*E*)-*N*-cyclohexyl-2-methylbut-2-enamide (1.8 g, 10 mmol, 1 equiv), LiHMDS (11 mL, 11 mmol, 1.1 equiv), triphosgene (2.9 g, 10 mmol, 1 equiv), and THF (50 mL, 0.2 M) were added to a 200 mL round bottom flask at rt. After 16 h, the reaction was worked up according to General Procedure C and purified using flash silica gel chromatography (0:100 to 15:85 acetone:hexanes) to provide **S54** as a 33:66 mixture of conformers that are kinetically stable at rt (0.88 g).

**S54** (0.88 g, 2.6 mmol, 1 equiv), as a mixture of kinetically stable conformers, phenoxyamine hydrochloride (0.76 g, 5.2 mmol, 2 equiv), $Et_3N$ (1.1 mL, 7.8 mmol, 3 equiv), and THF (10 mL, 0.2 M) were added to a 100 mL round bottom flask at rt. After 16 h, the reaction was worked up according to General Procedure C and purified using flash silica gel chromatography (0:100 to 15:85 acetone:hexanes) to afford **S55** as a dark red solid (0.55 g, 67% yield).

Data for **S54** (Tabulated as a mixture of two conformers where $^1H$ and $^{13}C$ NMR spectra are provided for identification purposes):

$^1H$ NMR (600 MHz, $CDCl_3$, Note: Spectrum shows mixture of kinetically stable conformers at rt, please see spectrum for details. Useful diagnostic peaks given.) δ 6.72–6.64 (m), 6.37–6.28 (m), 1.94–1.85 (m), 1.85–1.78 (m), 1.71–1.60 (m), 1.35–1.25 (m), 1.22–1.06 (m).

$^{13}C$ NMR (151 MHz, $CDCl_3$, Note: Spectrum shows mixture of kinetically stable conformers at rt, please see spectrum for details. Useful diagnostic peaks given.) δ 173.8, 147.1, 146.2, 141.9, 136.3, 135.9, 61.1, 58.7, 30.6, 26.2, 25.2, 15.2, 14.4, 12.7.

Data for **S55**:

$^1H$ NMR (600 MHz, $CDCl_3$) δ 10.22 (s, 1H), 7.33–7.27 (m, 2H), 7.09–7.04 (m, 3H), 5.99 (qq, *J* = 6.8, 1.5 Hz, 1H), 3.86 (tt, *J* = 12.0, 3.6 Hz, 1H), 2.22 (qd, *J* = 12.3, 3.5 Hz, 2H), 1.91 (s, 3H), 1.85–1.70 (m, 7H), 1.64–1.57 (m, 1H), 1.27–1.10 (m, 3H).

$^{13}C$ NMR (151 MHz, $CDCl_3$) δ 175.8, 159.7, 156.5, 133.4, 129.6, 129.4, 123.0, 113.3, 60.3, 30.7, 26.7, 25.2, 13.8, 13.7.

FTIR ($cm^{-1}$) 3174 (br), 2933, 1707, 1661, 1490, 1261, 752.

HRMS (ESI) m/z, calculated for $[C_{18}H_{25}N_2O_3]^+$ ($[M+H]^+$): 317.1865, found: 317.1865.

MP = 103–105 °C.

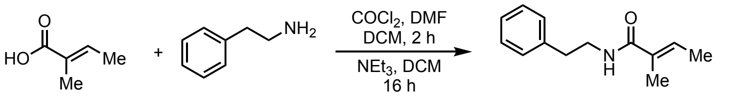


**S56**
Chemical Formula: $C_{13}H_{17}NO$
Exact Mass: 203.1310
Molecular Weight: 203.2850

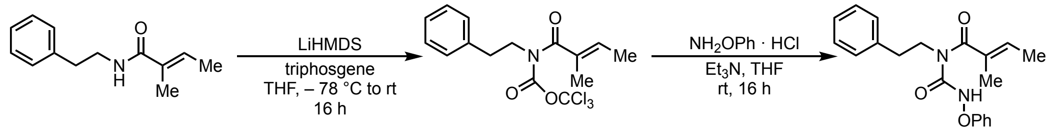


| **S56** | **S57** | **S58** |
|---|---|---|
| Chemical Formula: $C_{13}H_{17}NO$ | Chemical Formula: $C_{15}H_{16}Cl_3NO_3$ | Chemical Formula: $C_{20}H_{22}N_2O_3$ |
| Exact Mass: 203.1310 | Exact Mass: 363.0196 | Exact Mass: 338.1630 |
| Molecular Weight: 203.2850 | Molecular Weight: 364.6470 | Molecular Weight: 338.4070 |

(**S56**) According to **General Procedure B**, tiglic acid (20 mmol, 2.0 g, 1 equiv), DMF (1 mmol, 0.08 mL, 0.05 equiv), oxalyl chloride (24 mmol, 2.18 mL, 1.2 equiv), and $CH_2Cl_2$ (40 mL) were added to a 250 mL round bottom flask at rt. After 2 h, 2-phenylethan-1-amine (22 mmol, 2.8 mL), $Et_3N$ (30 mmol, 4.18 mL), and $CH_2Cl_2$ (40 mL) were added. After 16 h, **S56** was afforded as a dark brown oil (3.79 g, 93%) and used without further purification.

**S56** is a known compound. Acquired spectra are in accord with published data.[22] Partial characterization is shown below.

$^1$H NMR (600 MHz, $CDCl_3$) δ 7.32 (t, *J* = 7.3 Hz, 2H), 7.24 (t, *J* = 7.2 Hz, 1H), 7.21 (dd, *J* = 8.3, 1.6 Hz, 2H), 6.37 (qd, *J* = 7.0, 1.5 Hz, 1H), 5.68 (br s, 1H), 3.58 (q, *J* = 7.0 Hz, 2H), 2.85 (t, *J* = 7.0 Hz, 2H), 1.78 (s, 3H), 1.73 (dd, *J* = 7.0, 1.2 Hz, 3H).

$^{13}$C NMR (151 MHz, $CDCl_3$) δ 169.5, 139.2, 131.9, 130.7, 128.9, 128.8, 126.6, 40.9, 35.8, 14.0, 12.4.

(**S58**) According to **General Procedure C**, **S56** (1.0 g, 5 mmol, 1 equiv), LiHMDS (5.5 mL, 5.5 mmol, 1.1 equiv), triphosgene (1.5 g, 5 mmol, 1 equiv), and THF (37 mL) were added to a 200 mL round bottom flask at rt. After 16 h, the reaction was worked up according to General Procedure C and purified using flash silica gel chromatography (0:100 to 15:85 acetone:hexanes) to provide **S57** as a 45:55 mixture of conformers that are kinetically stable at rt (0.91 g).

**S57** (0.91g, 2.5 mmol, 1 equiv), as a mixture of kinetically stable conformers, phenoxyamine hydrochloride (1.46 g, 10.0 mmol, 2 equiv), $Et_3N$ (2.1 mL, 15 mmol, 3 equiv), and THF (25 mL, 0.2 M) were added to a 100 mL round bottom flask at rt. After 16 h, the reaction was worked up according to General Procedure C and purified using flash silica gel chromatography (0:100 to 15:85 acetone:hexanes) to afford **S58** as a dark red solid (0.71 g, 84% yield).

Data for **S57** (Tabulated as a mixture of two conformers where $^1$H and $^{13}$C NMR spectra are provided for identification purposes):

$^1$H NMR (600 MHz, $CDCl_3$, Note: Spectrum shows mixture of kinetically stable conformers at rt, please see spectrum for details. Useful diagnostic peaks given.) δ 6.26 (qq, *J* = 8.9, 1.4 Hz), 6.01 (qq, *J* = 7.0, 1.5 Hz), 1.88–1.86 (m), 1.80–1.77 (m), 1.76–1.73 (m).

$^{13}$C NMR (151 MHz, $CDCl_3$, Note: Spectrum shows mixture of kinetically stable conformers at rt, please see spectrum for details. Useful diagnostic peaks given.) δ 173.8, 148.8, 147.7, 137.6, 137.5, 134.4, 133.8, 129.1, 128.8, 127.0, 50.3, 47.6, 35.0, 14.7, 14.1, 13.1.

Data for **S58**:

$^1$H NMR (600 MHz, $CDCl_3$) δ 11.44 (s, 1H), 7.34–7.29 (m, 4H), 7.23 (appt tt, *J* = 6.7, 1.4 Hz, 1H), 7.15–7.11 (m, 4H), 7.06 (appt tt, *J* = 7.3, 1.1 Hz, 1H), 5.64 (qq, *J* = 6.8, 1.6 Hz, 1H), 3.98 (t, *J* = 7.4 Hz, 2H), 2.92 (t, *J* = 7.1 Hz, 2H) 1.74–1.72 (m, 6H).

$^{13}C$ NMR (151 MHz, $CDCl_3$) δ 176.7, 159.8, 155.9, 138.2, 132.1, 129.6, 129.2, 129.0, 128.9, 126.9, 123.1, 113.4, 48.7, 35.9, 13.9, 13.5.

FTIR ($cm^{-1}$) 3260 (br), 2926, 1725, 1661, 1489, 1199, 1075, 752.

HRMS (ESI) m/z, calculated for $[C_{20}H_{23}N_2O_3]^+$: 339.1709, found: 339.1700.

MP = 43–45 ºC.

## 7. Asymmetric Cyclization to Prepare Hydantoin Products

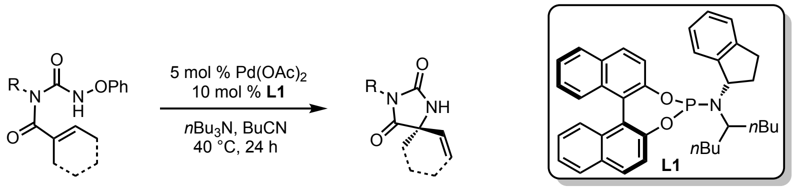


**General Procedure E:** A dried 10 mL or 20 mL Schlenk tube, equipped with a magnetic stirbar, and a teflon cap was attached to a double manifold via rubber hose and cooled under vacuum to rt. Once cooled, the Teflon cap was removed, $Pd(OAc)_2$ (5 mol%), **L1** (10 mol%), and phenoxyamide (1 equiv) were added sequentially to the tube, and the Teflon cap was replaced. The Schlenk tube was evacuated and backfilled with $N_2$ three times. The teflon cap was then replaced with a rubber septum, and anhydrous butyronitrile (0.25 M) and anhydrous tributyl amine (5 equiv) were added sequentially via syringe. The rubber septum was replaced with a Teflon cap, the Schlenk tube was sealed, and the reaction was maintained at the indicated temperature. After 24 h, the crude reaction mixture was transferred to a 50 mL round bottom flask using ethyl acetate (10 mL), concentrated onto celite (approximately 0.5 g) *in vacuo*, and directly purified using silica gel column chromatography to afford the hydantoin product.

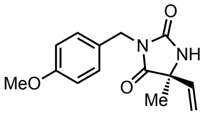

**5**
Chemical Formula: $C_{14}H_{16}N_2O_3$
Exact Mass: 260.1161
Molecular Weight: 260.2930

(**5**) According to **General Procedure E**, $Pd(OAc)_2$ (5.5 mg, 0.025 mmol, 0.05 equiv) **L1** (28.7 mg, 0.05 mmol, 0.1 equiv), **4** (180.0 mg, 0.5 mmol, 1 equiv), $nBu_3N$ (0.6. mL, 2.5 mmol, 5 equiv), and BuCN (2 mL, 0.25 M) were added to a 10 mL Schlenk tube and reacted for 24 h at 40 °C. The reaction was then worked up according to General Procedure E and purified via flash silica column chromatography (10:90 to 30:70 acetone:hexanes) to afford **5** as a yellow solid (113 mg, 87% yield).

94% ee. [CHIRALPAK IC-3, IPA/$CO_2$ = 3/97, v = 2.5 mL/min, λ = 254 nm, m/z = 262, t (major) = 7.97 min, t (minor) = 8.62 min].

$^1$H NMR (600 MHz, $CDCl_3$) δ 7.31–7.27 (m, 2H), 6.85–6.81 (m, 2H), 5.92 (dd, *J* = 17.2, 10.5 Hz, 1H), 5.57 (br s, 1H), 5.33 (d, *J* = 17.1 Hz, 1H), 5.24 (d, *J* = 10.5 Hz, 1H), 4.57 (s, 2H), 3.78 (s, 3H), 1.51 (s, 3H).

$^{13}$C NMR (101 MHz, $CDCl_3$) δ 174.3, 159.1, 156.1, 136.0, 129.7, 128.1, 115.9, 113.8, 62.8, 55.1, 41.5, 23.4.

FTIR (cm$^{-1}$) 3302 (br), 2934, 2837, 1776, 1714, 1514, 1442, 1412, 1249.

HRMS (ESI) m/z, calculated for $[C_{14}H_{17}N_2O_3]^+$ ($[M+H]^+$): 261.1239, found: 261.1234.

Optical rotation: $[\alpha]_1^{21}$= – 21.3° (1.01 mg/mL in $CHCl_3$) at 21 °C

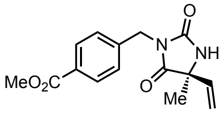

**6**
Chemical Formula: $C_{15}H_{16}N_2O_4$
Exact Mass: 288.1110
Molecular Weight: 288.3030

(**6**) According to **General Procedure E**, $Pd(OAc)_2$ (5.5 mg, 0.025 mmol, 0.05 equiv), **L1** (28.7 mg, 0.05 mmol, 0.1 equiv), **S11** (194 mg, 0.5 mmol, 1 equiv), $nBu_3N$ (0.6. mL, 2.5 mmol, 5 equiv), and BuCN (2 mL, 0.25 M) were added to a 10 mL Schlenk tube and reacted for 24 h at 40 °C. The reactions was worked up according to General Procedure E and purified via flash silica column chromatography (10:90 to 30:70 ethyl acetate:hexanes) to afford **6** as a yellow solid (130 mg, 78% yield).

91% ee. [Chiralpak OJ-3, MeOH/$CO_2$ = 3/97, v = 2.5 mL/min, λ = 254 nm, t (minor) = 7.42 min, t (major) = 8.06 min].

$^1$H NMR (400 MHz, $CDCl_3$) δ 8.03–7.92 (m, 2H), 7.38 (d, *J* = 8.1 Hz, 2H), 6.07 (br s, 1H), 5.93 (dd, *J* = 17.2, 10.5 Hz, 1H), 5.34 (d, *J* = 17.1 Hz, 1H), 5.25 (d, *J* = 10.5 Hz, 1H), 4.68 (s, 2H), 3.90 (s, 3H), 1.53 (s, 3H).

$^{13}$C NMR (101 MHz, $CDCl_3$) δ 171.3, 166.8, 141.0, 136.1, 130.2, 128.1, 116.3, 60.5, 52.2, 42.0, 23.7, 21.2, 14.3.

FTIR (cm$^{-1}$) 3314 (br), 2952, 1777, 1719, 1613, 1439, 1281, 1110.

HRMS (ESI) m/z, calculated for $[C_{15}H_{17}N_2O_4]^+$ ($[M+H]^+$): 289.1189, found: 289.1193.

MP = 82–85 °C

Optical rotation: $[\alpha]_1^{21}$= – 19.4 ° (2 mg/mL in $CHCl_3$) at 21 °C.

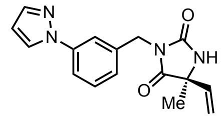


**7**
Chemical Formula: $C_{16}H_{16}N_4O_2$
Exact Mass: 296.1273
Molecular Weight: 296.3300

(**7**) According to **General Procedure E**, $Pd(OAc)_2$ (5.5 mg, 0.025 mmol, 0.05 equiv), **L1** (28.7 mg, 0.05 mmol, 0.1 equiv), **S14** (200 mg, 0.5 mmol, 1 equiv), $nBu_3N$ (0.6. mL, 2.5 mmol, 5 equiv), and BuCN (2 mL, 0.25 M) were added to a 10 mL Schlenk tube and reacted for 24 h at 40 °C. The reactions was worked up according to General Procedure E and purified via flash silica column chromatography (15:85 acetone:hexanes) to afford **7** as a yellow oil (103 mg, 70% yield).

89% ee. [Chiralpak OJ-3, MeCN/$CO_2$ = 15/85, v = 2.5 mL/min, m/z = 297, t (major) = 3.00 min, t (minor) = 3.35 min].

$^1$H NMR (600 MHz, $CDCl_3$) δ 7.90 (d, *J* = 2.4 Hz, 1H), 7.71 (d, *J* = 1.7 Hz, 1H), 7.67 (t, *J* = 1.9 Hz, 1H), 7.64 (dd, *J* = 8.2, 2.3 Hz, 1H), 7.40 (td, *J* = 7.8, 2.1 Hz, 1H), 7.25 (dd, *J* = 6.4, 1.3 Hz, 1H), 6.45 (dd, *J* = 2.5, 1.7 Hz, 1H), 5.94 (ddd, *J* = 17.1, 10.5, 2.2 Hz, 1H), 5.68 (br s, 1 H), 5.36 (dd, *J* = 17.2, 2.4 Hz, 1H), 5.29–5.24 (m, 1H), 4.70 (s, 2H), 2.17 (s, 3H).

$^{13}$C NMR (600 MHz, $CDCl_3$) $\delta$ 174.5, 156.1, 141.2, 140.5, 137.6, 136.2, 130.1, 127.1, 126.3, 119.1, 119.0, 116.4, 107.8, 63.3, 42.1, 23.7.

FTIR (cm$^{-1}$) 3290 (br), 2980, 2932, 1776, 1715, 1440, 1266, 1045, 753.

HRMS (ESI) m/z, calculated for $[C_{16}H_{17}N_4O_2]^+$ ($[M+H]^+$): 297.1352, found: 297.1351.

Optical rotation: $[\alpha]_1^{24}$= – 15.4° (1.0 mg/mL in $CHCl_3$) at 22 °C.

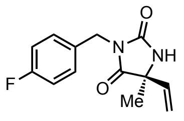


**8**
Chemical Formula: $C_{13}H_{13}FN_2O_2$
Exact Mass: 248.0961
Molecular Weight: 248.2574

(**8**) According to **General Procedure E**, $Pd(OAc)_2$ (5.5 mg, 0.025 mmol, 0.05 equiv), **L1** (28.7 mg, 0.05 mmol, 0.1 equiv), **S17** (170 mg, 0.5 mmol, 1 equiv), $nBu_3N$ (0.6 mL, 2.5 mmol, 5 equiv), and BuCN (2 mL, 0.25 M) were added to a 10 mL Schlenk tube and reacted for 24 h at 40 °C. The reaction was worked up according to general procedure E and purified via flash silica column chromatography (10:90 to 30:70 acetone:hexanes) to afford **8** as a thick colorless oil (97 mg, 78% yield).

87% ee. [Chiralpak OJ-3, MeOH/$CO_2$ = 5/95, v = 2.5 mL/min, λ = 262 nm, t (major) = 5.87 min, t (minor) = 6.57 min].

$^1$H NMR (600 MHz, $CDCl_3$) δ 7.34 (dd, *J* = 8.6, 5.3 Hz, 2H), 6.99 (appt t, *J* = 8.7 Hz, 2H), 5.92 (ddd, *J* = 17.0, 10.4, 1.2 Hz, 1H), 5.42 (br s, 1H), 5.33 (dd, *J* = 17.2, 1.2 Hz, 1H), 5.26 (dd, *J* = 10.5, 1.2 Hz, 1H), 4.60 (s, 2H), 1.52 (s, 3H).

$^{13}$C NMR (151 MHz, CDCl3) δ 174.4, 163.4, 161.8, 155.9, 136.2, 130.4 (d, *J* = 8.4 Hz), 116.4, 115.7 (d, *J* = 21.5 Hz), 63.1, 41.7, 23.7.

$^{19}$F NMR (376 MHz, $CDCl_3$) δ –114.27 (s).

FTIR ($cm^{-1}$) : 3299 (br), 1776, 1714, 1511, 1442, 1223, 1090.

HRMS (ESI) m/z, calculated for $[C_{13}H_{14}FN_2O_2]^+$ ($[M+H]^+$): 249.1039, found: 249.1036.

Optical rotation: $[\alpha]_1^{22}$ = + 10.7° (1 mg/mL in $CHCl_3$) at 22 °C.

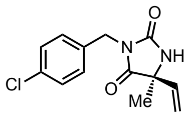

**9**
Chemical Formula: $C_{13}H_{13}ClN_2O_2$
Exact Mass: 264.0666
Molecular Weight: 264.7090

(**9**) According to **General Procedure E**, $Pd(OAc)_2$ (5.5 mg, 0.025 mmol, 0.05 equiv), **L1** (28.7 mg, 0.05 mmol, 0.1 equiv), **S19** (180 mg, 0.5 mmol, 1 equiv), $nBu_3N$ (0.6 mL, 2.5 mmol, 5 equiv), and BuCN (2 mL, 0.25 M) were added to a 10 mL Schlenk tube and reacted for 24 h at 40 °C. The reaction was worked up according to General Procedure E and purified via flash silica column chromatography (10:90 to 30:70 acetone:hexanes) to afford **9** as a brown solid (116 mg, 88%).

86% ee. [Chiralpak OJ-3, $MeOH/CO_2$ = 5/95, v = 2.5 mL/min, m/z = 265, t (major) = 2.75 min, t (minor) = 2.92 min].

$^1H$ NMR (600 MHz, $CDCl_3$) δ 7.36–7.30 (m, 2H), 6.99 (t, *J* = 8.7 Hz, 2H), 5.92 (dd, *J* = 17.2, 10.5 Hz, 1H), 5.66 (s, 1H), 5.33 (d, *J* = 17.2 Hz, 1H), 5.26 (d, *J* = 11.8 Hz, 1H), 4.60 (s, 2H), 1.52 (s, 3H).

$^{13}C$ NMR (151 MHz, $CDCl_3$) δ 174.3, 163.2, 161.6, 136.0, 131.8, 130.2, 116.2, 115.7, 63.0, 41.5, 23.6.

FTIR ($cm^{-1}$) 3295 (br), 2924, 2853, 1774, 1713, 1511, 1223, 1090, 784.

HRMS (ESI) m/z, calculated for $[C_{13}H_{14}ClN_2O_2]^+$ ($[M+H]^+$): 265.0744, found: 265.0742.

MP = 72–76 °C

Optical rotation: $[\alpha]_1^{21}$ = –19.7° (1.19 mg/mL in $CHCl_3$) at 21 °C

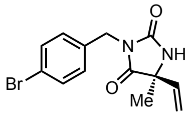

**10**
Chemical Formula: $C_{13}H_{13}BrN_2O_2$
Exact Mass: 308.0160
Molecular Weight: 309.1630

(**10**) According to **General Procedure E**, $Pd(OAc)_2$ (5.5 mg, 0.025 mmol, 0.05 equiv), **L1** (28.7 mg, 0.05 mmol, 0.1 equiv), **S22** (201 mg, 0.5 mmol, 1 equiv), $nBu_3N$ (0.6 mL, 2.5 mmol, 5 equiv), and BuCN (2 mL, 0.25 M) were added to a 10 mL Schlenk tube and reacted for 24 h at 40 °C. The reaction was worked up according to General Procedure E and purified via flash silica column chromatography (0:100 to 20:80 acetone:hexanes) to afford **10** as a white solid (22 mg, 15% yield).

63% ee. [CHIRALCEL OJ-3, $MeOH/CO_2$ = 5/95, v = 2.5 mL/min, m/z = 310, t (major) = 3.72 min, t (minor) = 3.97 min].

$^1H$ NMR (600 MHz, $CDCl_3$) δ 7.46–7.42 (m, 2H), 7.26–7.23 (m, 2H), 5.92 (dd, *J* = 17.2, 10.5 Hz, 1H), 5.34 (d, *J* = 17.2 Hz, 1H), 5.31 (br s, 1H), 5.27 (d, *J* = 10.5 Hz, 1H), 4.58 (s, 2H), 1.53 (s, 3H).

$^{13}C$ NMR (151 MHz, $CDCl_3$) δ 174.3, 155.8, 136.1, 135.0, 132.0, 130.2, 122.2, 116.5, 63.2, 41.8, 23.7.

FTIR ($cm^{-1}$) 3298 (br), 2927, 1776, 1715, 1554, 1488, 1262, 1062, 907, 799.

HRMS (ESI) m/z, calculated for $[C_{13}H_{14}BrN_2O_2]^+$ ($[M+H]^+$): 309.0239, found: 309.0234.

MP = 83–85 °C.

Optical rotation: $[\alpha]_1^{20}$ = – 7.68° (0.95 mg/mL in $CHCl_3$) at 20 °C.

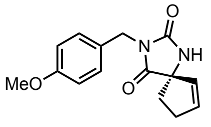

**11**
Chemical Formula: $C_{15}H_{16}N_2O_3$
Exact Mass: 272.1161
Molecular Weight: 272.3040

(**11**) According to **General Procedure E**, $Pd(OAc)_2$ (5.5 mg, 0.025 mmol, 0.05 equiv), **L1** (28.7 mg, 0.05 mmol, 0.1 equiv), **S25** (180 mg, 0.5 mmol, 1 equiv), $nBu_3N$ (0.6 mL, 2.5 mmol, 5 equiv), and BuCN (2 mL, 0.25 M) were added to a 10 mL Schlenk tube and were reacted for 24 h at 40 °C. The reaction was worked up according to General Procedure E and purified via flash silica column chromatography (10:90 to 30:70 acetone :hexanes) to afford **11** as a white solid (130 mg, 99% yield).

99% ee. [Chiralpak IB-3, $MeOH/CO_2$ = 5/95, v = 2 mL/min, λ = 254 nm, t (minor) = 3.84 min, t (major) = 4.14 min].

$^1H$ NMR (600 MHz, $CDCl_3$) δ 7.35–7.32 (m, 2H), 6.86–6.82 (m, 2H), 6.19 (dt, *J* = 5.5, 2.3 Hz, 1H), 5.46 (dt, *J* = 5.5, 2.1 Hz, 1H), 5.36 (br s, 1H), 4.60 (s, 2H), 3.79 (s, 3H), 2.68–2.45 (m, 3H), 1.96 (ddd, *J* = 13.7, 8.1, 5.1 Hz, 1H).

$^{13}C$ NMR (151 MHz, $CDCl_3$) δ 175.8, 159.4, 156.1, 139.2, 130.2, 128.7, 128.5, 114.2, 73.3, 55.4, 42.0, 35.0, 31.9.

FTIR ($cm^{-1}$) 3307 (br), 2936, 2836, 1772, 1712, 1514, 1441, 1247.

HRMS (ESI) m/z, calculated for $[C_{15}H_{17}N_2O_3]^+$ ($[M+H]^+$): 273.1239, found: 273.1233.

MP = 127–130 °C.

Optical rotation: $[\alpha]_1^{22}$= + 70.7 (1.9 mg/mL in $CHCl_3$) at 22 °C.

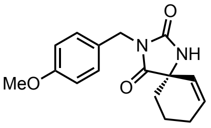

**12**
Chemical Formula: $C_{16}H_{18}N_2O_3$
Exact Mass: 286.1317
Molecular Weight: 286.3310

(**12**) According to **General Procedure E**, $Pd(OAc)_2$ (5.5 mg, 0.025 mmol, 0.05 equiv), **L1** (28.7 mg, 0.05 mmol, 0.1 equiv), **S28** (190 mg, 0.5 mmol, 1 equiv), $nBu_3N$ (0.6 mL, 2.5 mmol, 5 equiv), and BuCN (2 mL, 0.25 M) were added to a 10 mL Schlenk tube and reacted for 24 h at 40 °C. The reaction was worked up according to General Procedure E and purified via flash silica column chromatography (10:90 to 30:70 acetone:hexanes) to afford **12** as a yellow solid (87 mg, 61% yield).

93% ee. [Chiralpak IB-3 $MeOH/CO_2$ = 5/95, v = 2 mL/min, λ = 254 nm, t (minor) = 3.53 min, t (major) = 4.21 min].

$^1H$ NMR (400 MHz, $CDCl_3$) δ 7.32–7.29 (m, 2H), 6.85–6.81 (m, 2H), 6.11 (br s, 1H), 6.11 (dt, *J* = 9.9, 3.7 Hz, 2H), 5.37 (dt, *J* = 10.0, 2.2 Hz, 1H), 4.58 (s, 2H), 3.77 (s, 3H), 2.14–2.00 (m, 4H), 1.79–1.68 (m, 1H), 1.60 (tdd, *J* = 14.0, 6.8, 3.9 Hz, 1H).

$^{13}C$ NMR (101 MHz, $CDCl_3$) δ 175.8, 159.3, 156.6, 134.9, 130.0, 128.6, 123.6, 114.1, 60.8, 55.4, 41.8, 32.6, 24.1, 18.6.

FTIR ($cm^{-1}$) 3279 (br), 2849, 2361, 1733, 1624, 1512, 1247, 1174, 1033.

HRMS (ESI) m/z, calculated for $[C_{16}H_{19}N_2O_3]^+$ ($[M+H]^+$): 287.1396, found: 287.1392.

MP = 118–121 °C.

Optical rotation: $[\alpha]_1^{22}$ + 3.3 (1 mg/mL in $CHCl_3$) at 22 °C.

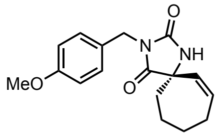

**13**
Chemical Formula: $C_{17}H_{20}N_2O_3$
Exact Mass: 300.1474
Molecular Weight: 300.3580

(**13**) According to **General Procedure E**, $Pd(OAc)_2$ (5.5 mg, 0.025 mmol, 0.05 equiv), **L1** (28.7 mg, 0.05 mmol, 0.1 equiv), **S31** (200 mg, 0.5 mmol, 1 equiv), $nBu_3N$ (20.6 mL, 2.5 mmol, 5 equiv), and BuCN (2 mL, 0.25 M) were added to a 10 mL Schlenk tube and reacted for 24 h at 40 °C. The reactions was worked up according to General Procedure E and purified via flash silica column chromatography (5:95 to 20:80 acetone:hexanes) to afford **13** as a yellow solid (120 mg, 76% yield).

97% ee. [Chiralpak IB-3, $MeOH/CO_2$ = 5/95, v = 2.5 mL/min, t (minor) = 3.23 min, t (major) = 3.63 min].

$^1H$ NMR (600 MHz, $CDCl_3$) δ 7.33–7.30 (m, 2H), 6.84 (dt, *J* = 5.1, 3.0 Hz, 2H), 6.03 (ddd, *J* = 11.7, 6.4, 5.4 Hz, 1H), 5.61 (br s, 1H), 5.41 (d, *J* = 1.6 Hz, 1H), 4.58 (s, 2H), 3.78 (s, 3H), 2.42–2.34 (m, 1H), 2.21–2.13 (m, 1H), 2.07 (ddd, *J* = 13.8, 9.9, 3.9 Hz, 1H), 2.02 (ddt, *J* = 13.1, 8.1, 3.4 Hz, 1H), 1.82–1.74 (m, 2H), 1.74–1.68 (m, 1H), 1.65–1.57 (m, 1H).

$^{13}C$ NMR (151 MHz, $CDCl_3$) δ 175.5, 159.4, 156.2, 137.6, 130.0, 128.5, 114.2, 65.5, 55.4, 41.8, 40.4, 35.9, 28.0, 27.3, 24.0.

FTIR ($cm^{-1}$) 3235 (br), 2932, 1769, 1709, 1513, 1247, 1176, 776.

HRMS (ESI) m/z, calculated for $[C_{17}H_{21}N_2O_3]^+$ ($[M+H]^+$): 301.1552, found: 301.1546.

MP = 112–116 °C.

Optical rotation: $[\alpha]_1^{22}$= + 20.7 (1 mg/mL in $CHCl_3$) at 22 °C.

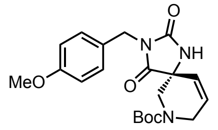

**14**
Chemical Formula: $C_{20}H_{25}N_3O_5$
Exact Mass: 387.1794
Molecular Weight: 387.4360

(**14**) According to **General Procedure E**, $Pd(OAc)_2$ (5.5 mg, 0.025 mmol, 0.05 equiv), **L1** (28.7 mg, 0.05 mmol, 0.1 equiv), **S33** (240 mg, 0.5 mmol, 1 equiv), $nBu_3N$ (0.6 mL, 2.5 mmol, 5 equiv), and BuCN (2 mL, 0.25 M) were added to a 10 mL Schlenk tube and reacted for 24 h at 40 °C. The reaction was worked up according to General Procedure E and purified via flash silica column chromatography (10:90 to 30:70 acetone:hexanes) to afford **14** as a brown solid (140 mg, 71% yield).

97% ee. [Chiralpak IB-3, $MeOH/CO_2$ = 5/95, v = 2.5 mL/min, m/z = 288, t (minor) = 4.25 min, t (major) = 4.81 min].

$^1H$ NMR (600 MHz, $CD_3CN$) δ 7.23 (d, *J* = 8.1 Hz, 2H), 6.91–6.84 (m, 2H), 6.51 (s, 1H), 6.10 (s, 1H), 5.59 (dt, *J* = 10.1, 2.5 Hz, 1H), 4.50 (d, *J* = 1.7 Hz, 2H), 4.09–3.82 (m, 2H), 3.76 (s, 3H), 3.72–3.43 (m, 2H), 1.48–1.32 (m, 9H).

$^{13}C$ NMR (151 MHz, $CD_3CN$) δ 160.1, 156.8, 131.7, 130.8, 129.8, 123.9, 114.9, 80.6, 60.2, 55.8, 48.8, 47.7, 43.7, 42.6, 42.1, 28.4.

FTIR ($cm^{-1}$) 3245 (br), 2925, 2361, 2336, 1716, 1700, 1652, 1245, 1175, 1033.

HRMS (ESI) m/z, calculated for $[C_{20}H_{26}N_3O_5]^+$ ($[M+H]^+$): 388.1873, found: 388.1865.

MP = 134–137 °C.

Optical rotation: $[\alpha]_D^{22}$= + 3.3 (1 mg/mL in $CHCl_3$) at 23 °C.

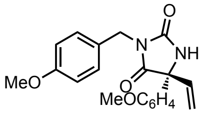

15
Chemical Formula: $C_{20}H_{20}N_2O_4$
Exact Mass: 352.1423
Molecular Weight: 352.3900

(**15**) According to **General Procedure E**, $Pd(OAc)_2$ (5.5 mg, 0.025 mmol, 0.05 equiv), **L1** (28.7 mg, 0.05 mmol, 0.1 equiv), **S35** (220 mg, 0.5 mmol, 1 equiv), $nBu_3N$ (0.6 mL, 2.5 mmol, 5 equiv), and BuCN (2 mL, 0.25 M) were added to a 10 mL Schlenk tube and reacted for 24 h at 40 °C. The reaction was worked up according to General Procedure E and purified via flash silica column chromatography (10:90 to 30:70 acetone:hexanes) to afford **15** as a yellow solid (101 mg, 56% yield).

91% ee. [Chiralpak IB-3, $MeOH/CO_2$ = 10/90, v = 2.5 mL/min, m/z = 353, t (minor) = 2.10 min, t (major) = 2.31 min].

$^1H$ NMR (600 MHz, $CDCl_3$) δ 7.31–7.27 (m, 4H), 6.89–6.86 (m, 2H), 6.83–6.80 (m, 2H), 6.24 (dd, *J* = 17.1, 10.4 Hz, 1H), 5.88 (br s, 1H), 5.42 (d, *J* = 17.2 Hz, 1H), 5.38 (d, *J* = 10.4 Hz, 1H), 4.61 (d, *J* = 2.1 Hz, 2H), 3.79 (s, 3H), 3.78 (s, 3H).

$^{13}C$ NMR (151 MHz, $CDCl_3$) δ 173.0, 160.0, 159.4, 156.3, 135.8, 129.9, 129.4, 128.3, 127.4, 117.0, 114.5, 114.1, 68.1, 55.5, 55.4, 42.1.

FTIR ($cm^{-1}$) 3303 (br), 2930, 1774, 1712, 1611, 1513, 1250, 1033, 832.

HRMS (ESI) m/z, calculated for $[C_{20}H_{21}N_2O_4]^+$ ($[M+H]^+$): 353.1502, found: 353.1507.

MP = 84–86 °C.

Optical rotation: $[\alpha]_1^{22}$= – 19.0 (1 mg/mL in $CHCl_3$) at 22 °C.

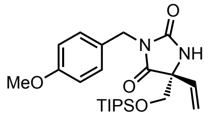

16
Chemical Formula: $C_{23}H_{36}N_2O_4Si$
Exact Mass: 432.2444
Molecular Weight: 432.6360

(**16**) According to **General Procedure E**, $Pd(OAc)_2$ (5.5 mg, 0.025 mmol, 0.05 equiv), **L1** (28.7 mg, 0.05 mmol, 0.1 equiv), **S37** (263 mg, 0.5 mmol, 1 equiv), $nBu_3N$ (0.6 mL, 2.5 mmol, 5 equiv), and BuCN (2 mL, 0.25 M) were added to a 10 mL Schlenk tube and reacted for 24 h at 40 °C. The reaction was worked up according to General Procedure E and purified via flash silica column chromatography (20:80 diethyl ether:hexanes) to afford **16** as a light brown oil (104 mg, 48% yield).

95% ee. [Chiralpak IB-3, $MeOH/CO_2$ = 5/95, v = 2.5 mL/min, λ = 269 nm, m/z = 433, t (minor) = 1.81 min, t (major) = 2.24 min].

$^1H$ NMR (600 MHz, $CDCl_3$) δ 7.33–7.28 (m, 2H), 6.83–6.79 (m, 2H), 5.90 (dd, *J* = 17.2, 10.6 Hz, 1H), 5.41 (d, *J* = 17.2 Hz, 1H), 5.32 (d, *J* = 10.6 Hz, 1H), 5.25 (s, 1H), 4.60–4.51 (m, 2H), 4.05 (d, *J* = 10.0 Hz, 1H), 3.77 (s, 3H), 3.69 (d, *J* = 10.0 Hz, 1H), 1.02–0.94 (m, 21H, all protons from TIPS group overlapping).

$^{13}C$ NMR (151 MHz, $CDCl_3$) δ 172.7, 159.3, 157.0, 132.5, 130.1, 128.4, 118.2, 114.1, 68.7, 66.6, 55.4, 41.9, 17.9, 11.9.

FTIR ($cm^{-1}$) 3287 (br), 2943, 2866, 1716, 1614, 1444, 1249, 1126, 798.

HRMS (ESI) m/z, calculated for $[C_{23}H_{37}N_2O_4Si]^+$: 433.2523, found: 433.2517.

Optical rotation: $[\alpha]_1^{24}$= + 27.3° (1 mg/mL in $CHCl_3$) at 22 °C.

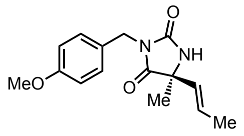

**17**
Chemical Formula: $C_{15}H_{18}N_2O_3$
Exact Mass: 274.1317
Molecular Weight: 274.3200

(**17**) According to **General Procedure E**, $Pd(OAc)_2$ (5.5 mg, 0.025 mmol, 0.05 equiv), **L1** (28.7 mg, 0.05 mmol, 0.1 equiv), **S40** (184 mg, 0.5 mmol, 1 equiv), $nBu_3N$ (0.6 mL, 2.5 mmol, 5 equiv), and BuCN (2 mL, 0.25 M) were added to a 10 mL Schlenk tube and reacted for 24 h at 40 °C. The reaction was worked up according to General Procedure E and purified via flash silica column chromatography (10:90 acetone:hexanes) to afford **17** as a light yellow oil (110 mg, 82% yield).

89% ee. [Chiralpak IB-3, $MeOH/CO_2$ = 4/96, v = 2.0 mL/min, λ = 269 nm, t (major) = 3.07 min, t (minor) = 3.44 min].

$^1H$ NMR (600 MHz, $CDCl_3$) δ 7.31 – 7.28 (m, 2H), 6.85 – 6.82 (m, 2H), 5.74 (dq, *J* = 15.4, 6.5 Hz, 1H), 5.52 (dq, *J* = 15.4, 1.6 Hz, 1H), 5.28 (s, 1H), 4.57 (s, 2H), 3.78 (s, 3H), 1.70 (dd, *J* = 6.5, 1.6 Hz, 3H), 1.48 (s, 3H).

$^{13}C$ NMR (151 MHz, $CDCl_3$) δ 175.1, 159.3, 156.2, 129.9, 129.1, 128.5, 127.7, 114.1, 62.5, 55.4, 41.8, 24.0, 17.7.

FTIR ($cm^{-1}$) 3301 (br), 2935, 1771, 1712, 1514, 1248, 776.

HRMS (ESI) m/z, calculated for $[C_{15}H_{19}N_2O_3]^+$: 275.1396, found: 275.1394.

Optical rotation: $[\alpha]_1^{21}$= – 13.1° (1 mg/mL in $CHCl_3$) at 21 °C.

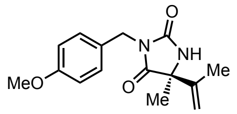

**18**
Chemical Formula: $C_{15}H_{18}N_2O_3$
Exact Mass: 274.1317
Molecular Weight: 274.3200

(**18**) According to **General Procedure E**, $Pd(OAc)_2$ (1.1 mg, 0.005 mmol, 0.05 equiv), **L1** (5.7 mg, 0.01 mmol, 0.1 equiv), **S43** (36.8 mg, 0.1 mmol, 1 equiv), $nBu_3N$ (119 uL, 0.5 mmol, 5 equiv), and BuCN (0.4 mL, 0.25 M) were added to a 10 mL Schlenk tube and reacted for 24 h at 75 °C. The reaction was worked up according to General Procedure E and purified via reverse phase column chromatography using C18 silica gel (50:50 MeOH:water). The isolated product was then purified a second time using flash silica gel chromatography (10:90 acetone:hexanes) to afford **18** as a yellow solid in 80% purity. $^1H$ and $^{13}C$ NMR are provided for identification purposes only.

63% ee. [CHIRALPAK ID-3, $MeOH/CO_2$ = 5/95, v = 2.5 mL/min, λ = 220 nm, m/z = 275, t (major) = 2.05 min, t (minor) = 2.41 min].

$^1H$ NMR (600 MHz, $CDCl_3$) δ 7.31–7.28 (m, 2H), 6.85–6.80 (m, 2H), 5.64 (br s, 1H), 5.12 (s, 1H), 5.02 (appt q, *J* = 1.5 Hz, 1H), 4.61 (s, 2H), 3.78 (s, 3H), 1.73 (s, 3H), 1.55 (s, 3H).

$^{13}C$ NMR (151 MHz, $CDCl_3$) δ 174.6, 159.4, 156.4, 142.1, 130.0, 128.4, 114.3, 114.1, 64.8, 55.4, 41.9, 22.6, 18.7.

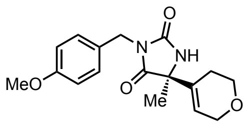

**19**
Chemical Formula: $C_{17}H_{20}N_2O_4$
Exact Mass: 316.1423
Molecular Weight: 316.3570

(**19**) According to **General Procedure E**, $Pd(OAc)_2$ (1.1 mg, 0.005 mmol, 0.05 equiv), **L1** (5.7 mg, 0.01 mmol, 0.1 equiv), **S46** (41.0 mg, 0.1 mmol, 1 equiv), $nBu_3N$ (119 uL, 0.5 mmol, 5 equiv), and BuCN (0.4 mL, 0.25 M) were added to a 10 mL Schlenk tube and reacted for 24 h at 75 °C. The reaction was worked up according to General Procedure E and purified via flash silica column chromatography (15:85 acetone:hexanes) to afford **19** as a white solid in 90% purity (53 mg). $^1H$, $^{13}C$ NMR, and HRMS are provided for identification purposes only.

83% ee. [Chiralpak IB-3, $MeOH/CO_2$ = 5/95, v = 2.5 mL/min, m/z = 317, t (major) = 4.87 min, t (minor) = 5.28 min].

$^{1}H$ NMR (600 MHz, $CD_3CN$) δ 7.03–7.00 (m, 2H), 6.69–6.66 (m, 2H), 6.19 (br s, 1H), 5.67 (sept, $J$ = 1.5, 1H), 4.31 (d, $J$ = 3.0, 2H), 3.89 (qq, $J$ = 17.1, 2.8 Hz, 2H), 3.57 (s, 3H), 3.50 (dt, $J$ = 11.0, 5.8 Hz, 1H), 3.45–3.39 (m, 1H), 2.00–1.94 (m, 1H), 1.69–1.62 (m, 1H), 1.28 (s, 3H).

$^{13}C$ NMR (151 MHz, $CD_3CN$) δ 175.8, 160.2, 156.9, 134.3, 130.0, 130.0, 124.5, 114.9, 67.2, 65.9, 64.6, 55.9, 42.0, 25.1, 21.9.

HRMS (ESI) m/z, calculated for $[C_{17}H_{21}N_2O_4]^+$: 317.1501, found: 317.1493.

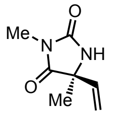


**21**
Chemical Formula: $C_7H_{10}N_2O_2$
Exact Mass: 154.0742
Molecular Weight: 154.1690

(**21**) According to **General Procedure E**, $Pd(OAc)_2$ (5.5 mg, 0.025 mmol, 0.05 equiv), **L1** (28.7 mg, 0.05 mmol, 0.1 equiv), **S50** (124 mg, 0.5 mmol, 1 equiv), $nBu_3N$ (0.6 mL, 2.5 mmol, 5 equiv), and BuCN (2 mL, 0.25 M) were added to a 10 mL Schlenk tube and reacted for 24 h at rt. The reaction was worked up according to General Procedure E and purified via flash silica column chromatography (10:90 to 15:85 acetone:hexanes) to afford **21** as a light yellow solid (65 mg, 85% yield).

95% ee. [Chiralpak OJ-3, $MeOH/CO_2$ = 5/95, v = 2.0 mL/min, t (major) = 1.72 min, t (minor) = 1.91 min].

$^{1}H$ NMR (600 MHz, $CDCl_3$) δ 5.94 (dd, $J$ = 17.2, 10.5 Hz, 1H), 5.95 (br s, 1H), 5.36 (d, $J$ = 17.2 Hz, 1H), 5.27 (d, $J$ = 10.5 Hz, 1H), 3.01 (s, 3H), 1.54 (s, 3H).

$^{13}C$ NMR (151 MHz, $CDCl_3$) δ 174.9, 156.7, 136.3, 116.2, 63.2, 24.9, 23.8.

FTIR ($cm^{-1}$) 3300 (br), 2930, 1757, 1708, 1458, 1163, 780.

HRMS (ESI) m/z, calculated for $[C_7H_{11}N_2O_2]^+$ ($[M+H]^+$): 155.0821, found: 155.0819.

MP = 105-109 °C.

Optical rotation: $[\alpha]_1^{21}$= – 17.9° (1.0 mg/mL in $CHCl_3$) at 21 °C.

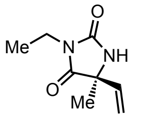


**22**
Chemical Formula: $C_8H_{12}N_2O_2$
Exact Mass: 168.0899
Molecular Weight: 168.1960

(**22**) According to **General Procedure E**, $Pd(OAc)_2$ (5.5 mg, 0.025 mmol, 0.05 equiv), **L1** (28.7 mg, 0.05 mmol, 0.1 equiv), **S53** (131 mg, 0.5 mmol, 1 equiv), $nBu_3N$ (0.6 mL, 2.5 mmol, 5 equiv), and BuCN (2 mL, 0.25 M) were added to a 10 mL Schlenk tube and reacted for 24 h at rt. The reaction was worked up according to General Procedure E and purified via flash silica column chromatography (10:90 acetone:hexanes) to afford **22** as a white solid (60 mg, 71% yield).

65% ee. [Chiralcel OJ-3, $MeOH/CO_2$ = 1/99, v = 2.5 mL/min, m/z = 169, t (major) = 2.49 min, t (minor) = 2.79 min].

$^{1}H$ NMR (600 MHz, $CDCl_3$) δ 5.94 (dd, $J$ = 17.2, 10.5 Hz, 1H), 5.83 (br s, 1H), 5.36 (d, $J$ = 17.2 Hz, 1H), 5.26 (d, $J$ = 10.5 Hz, 1H), 3.54 (q, $J$ = 7.2 Hz, 2H), 1.53 (s, 3H), 1.20 (t, $J$ = 7.2 Hz, 3H).

$^{13}C$ NMR (151 MHz, $CDCl_3$) δ 174.7, 156.5, 136.4, 116.1, 63.0, 33.9, 23.7, 13.3.

FTIR ($cm^{-1}$) 3300 (br), 2980, 1780, 1715, 1453, 1272, 754.

HRMS (ESI) m/z, calculated for $[C_8H_{13}N_2O_2]^+$ ($[M+H]^+$): 169.0977, found: 169.0973.

MP = 76–80 °C.

Optical rotation: $[\alpha]_1^{24}$= – 18.3 (1.0 mg/mL in $CHCl_3$) at 21 °C.

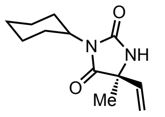

**23**
Chemical Formula: $C_{12}H_{18}N_2O_2$
Exact Mass: 222.1368
Molecular Weight: 222.2880

(**23**) According to **General Procedure E**, $Pd(OAc)_2$ (5.5 mg, 0.025 mmol, 0.05 equiv), **L1** (28.7 mg, 0.05 mmol, 0.1 equiv), **S55** (158 mg, 0.5 mmol, 1 equiv), $nBu_3N$ (0.6 mL, 2.5 mmol, 5 equiv), and BuCN (2 mL, 0.25 M) were added to a 10 mL Schlenk tube and reacted for 24 h at rt. The reaction was worked up according to General Procedure E and purified via flash silica column chromatography (30:70 diethyl ether:hexanes) to afford **23** as a white solid (36 mg, 33% yield).

49% ee. [Chiralcel OJ-3, $MeOH/CO_2$ = 1/99, v = 2.5 mL/min, m/z = 223, t (major) = 3.01 min, t (minor) = 3.45 min].

$^1H$ NMR (600 MHz, $CDCl_3$) δ 5.92 (dd, *J* = 17.2, 10.5 Hz, 1H), 5.33 (d, *J* = 17.2 Hz, 1H), 5.29 (br s, 1H), 5.25 (d, *J* = 10.5 Hz, 1H), 3.86 (tt, *J* = 12.4, 3.9 Hz, 1H), 2.10 (qd, *J* = 12.6, 3.7 Hz, 2H), 1.82 (dd, *J* = 12.4, 4.2 Hz, 2H), 1.70–1.61 (m, 2H), 1.50 (s, 3H), 1.35–1.16 (m, 4H).

$^{13}C$ NMR (151 MHz, $CDCl_3$) δ 174.8, 156.4, 136.6, 116.0, 62.1, 51.6, 29.4, 26.0, 25.1, 23.8.

FTIR ($cm^{-1}$) 3242 (br), 2932, 2855, 1769, 1709, 1423, 1091, 756.

HRMS (ESI) m/z, calculated for $[C_{12}H_{19}N_2O_2]^+$ ($[M+H]^+$): 223.1441, found: 223.1442.

MP = 97–100 °C.

Optical rotation: $[\alpha]_1^{21}$= – 20.5° (1.0 mg/mL in $CHCl_3$) at 21 °C.

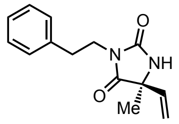

**24**
Chemical Formula: $C_{14}H_{16}N_2O_2$
Exact Mass: 244.1212
Molecular Weight: 244.2940

(**24**) According to **General Procedure E**, $Pd(OAc)_2$ (5.5 mg, 0.025 mmol, 0.05 equiv), **L1** (28.7 mg, 0.05 mmol, 0.1 equiv), **S58** (169 mg, 0.5 mmol, 1 equiv), $nBu_3N$ (0.6 mL, 2.5 mmol, 5 equiv), and BuCN (2 mL, 0.25 M) were added to a 10 mL Schlenk tube and reacted for 24 h at 40 °C. The reaction was worked up according to General Procedure E and purified via flash silica column chromatography (5:95 to 30:70 acetone:hexanes) to afford **24** as a light yellow solid (96 mg, 78% yield).

86% ee. [Chiralpak ID-3, $MeOH/CO_2$ = 3/97, v = 1.0 mL/min, m/z = 245, t (minor) = 18.15 min, t (major) = 20.47].

$^1H$ NMR (600 MHz, $CDCl_3$) δ 7.30–7.27 (m, 3H), 7.22–7.19 (m, 2H), 5.86 (dd, *J* = 17.2, 10.6 Hz, 1H), 5.28 (d, *J* = 17.1 Hz, 1H), 5.23 (d, *J* = 10.5 Hz, 1H), 5.21 (s, 1H), 3.74 (dt, *J* = 7.1, 1.1 Hz, 2H), 2.94 (t, *J* = 7.5 Hz, 2H), 1.44 (s, 3H).

$^{13}C$ NMR (151 MHz, $CDCl_3$) δ 174.6, 156.1, 137.8, 136.2, 129.2, 128.6, 126.8, 116.2, 62.8, 39.8, 33.9, 23.7.

FTIR ($cm^{-1}$) 3283 (br), 2932, 1774, 1678, 1453, 1118, 935, 752, 700.

HRMS (ESI) m/z, calculated for $[C_{14}H_{17}N_2O_2]^+$: 245.1290, found: 245.1288.

MP = 87–89 °C.

Optical rotation: $[\alpha]_1^{23}$= – 17.05° (0.1 mg/mL in $CHCl_3$) at 23 °C.

## 8. Observation and Analysis of Wacker By-product

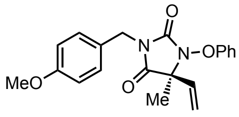


**26**
Chemical Formula: $C_{20}H_{20}N_2O_4$
Exact Mass: 352.1423
Molecular Weight: 352.3900

(**26**) An oven dried 10 mL round bottom flask equipped with a magnetic stirbar and rubber septum was connect to a double manifold via rubber tube and needle adapter and cooled under vacuum. Once cooled to rt, the flask was placed under $N_2$, the septum was quickly removed, $Pd(OAc)_2$ (11 mg, 0.1 mmol, 0.1 equiv), **L1** (57 mg, 0.2 mmol, 0.2 equiv), and **4** (0.35 g, 1 mmol, 1 equiv) were added sequentially. The flask was evacuated and backfilled with $N_2$ three times. Then anhydrous BuCN (4 mL, 0.25 M) was added via syringe. The reaction was placed under a balloon filled with oxygen gas. After addition of a vent needle, oxygen was bubbled through the reaction mixture for 15 minutes. The vent needle was removed, and the reaction was maintained under the oxygen balloon at rt. After 24 h, the oxygen balloon and septum were removed, and the reaction mixture was directly concentrated *in vacuo*. The crude mixture was purified via silica column chromatography (10:90 to 20:80 diethyl ether:hexanes) to afford **26** as a colorless oil (72 mg, 20% yield).

9% ee. [Chiralcel IC-3, MeOH/$CO_2$ = 3/97, v = 2.0 mL/min, t (major) = 3.19 min, t (minor) = 3.50 min].

$^1$H NMR (600 MHz, $CDCl_3$) δ 7.38–7.33 (m, 2H), 7.33–7.27 (m, 2H), 7.15–7.11 (m, 2H), 7.07 (tt, *J* = 7.3, 1.1 Hz, 1H), 6.90–6.84 (m, 2H), 5.91 (dd, *J* = 17.3, 10.8 Hz, 1H), 5.36 (d, *J* = 17.3 Hz, 1H), 5.32 (d, *J* = 10.8 Hz, 1H), 4.66 (d, *J* = 1.9 Hz, 2H), 3.80 (s, 3H), 1.60 (s, 3H).

$^{13}$C NMR (151 MHz, $CDCl_3$) δ 171.4, 159.9, 159.6, 157.7, 133.4, 130.3, 129.6, 127.7, 123.8, 119.5, 114.5, 114.3, 69.6, 55.4, 42.5, 19.9.

FTIR (cm$^{-1}$) 2934, 2837, 1794, 1728, 1514, 1401, 1248, 751.

HRMS (ESI) m/z, calculated for $[C_{20}H_{21}N_2O_4]^+$: 353.1502, found: 353.1510.

## 8.1 Palladium Loading Studies to Observe Wacker Byproduct

In a $N_2$-filled glovebox, $Pd(OAc)_2$ (5-10 mol %), ligand (10-20 mol %), and **4** (1 equiv) were added to an oven-dried 1-dram vial containing an 8 mm Teflon-coated stirbar. Anhydrous BuCN (0.25 M) and $nBu_3N$ (5 equiv) were added, and the vial was capped. The mixture was stirred at 40 °C inside the glovebox in a preheated aluminum-block set atop a stirplate. After 24 h, the reaction vial was removed from the glovebox, cooled to rt, and concentrated *in vacuo*. Yields were obtained by $^1$H NMR of the crude product compared to an internal standard (1,3,5-trimethoxybenzene), and %ee values were determined by chiral SFC-MS analysis.

**Table S7. Evaluation of Palladium Loading and Wacker Byproduct[a]**

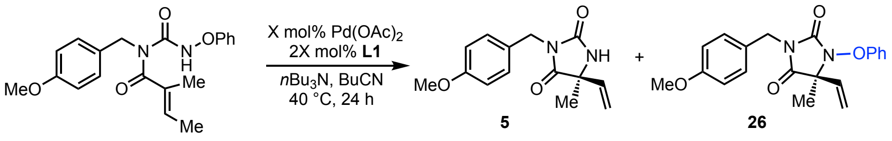


| Entry | Pd (mol %) | L1 (mol %) | Yield 5 (%) | Yield 26 (%) |
|---|---|---|---|---|
| 1 | 5 | 10 | 94 | 5 |
| 2 | 7.5 | 15 | 90 | 8 |
| 3 | 10 | 20 | 88 | 10 |
| 4 | 20 | 40 | 83 | 16 |

[a] Conditions: **4** (0.1 mmol), $Pd(OAc)_2$ (X mol %), **L1** (2X mol %), base (5 equiv) in BuCN (2 mL) at 40 °C for 24 h. Yield was determined by $^1$H NMR of the crude reaction mixture using 1,3,5-trimethoxybenzene as an internal standard. %ee was determined using SFC-MS analysis.

## 8.2 Spiking Experiment with 26

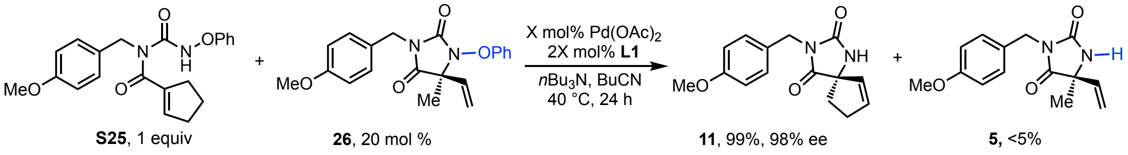


In a $N_2$-filled glovebox, $Pd(OAc)_2$ (1.1 mg, 5 mol %, 0.05 equiv), ligand (5.7 mg, 0.1 mmol, 0.1 equiv), **S25** (36.6 mg, 0.1 mmol, 1 equiv), and **26** (7.0 mg, 20 mol %, 0.2 equiv) were added to an oven-dried 1-dram vial containing an 8 mm Teflon-coated stirbar. Anhydrous BuCN (0.4 mL, 0.25 M) and $nBu_3N$ (119 uL, 0.5 mmol, 5 equiv) were added, and the vial was capped. The mixture was stirred at 40 °C inside the glovebox in a preheated aluminum-block set atop a stirplate. After 24 h, the reaction vial was removed from the glovebox, cooled to rt, and concentrated *in vacuo*. Yields were obtained by $^1$H NMR of the crude product compared to an internal standard (1,3,5-trimethoxybenzene), and %ee values were determined by chiral SFC-MS analysis.

## 9. Deprotection of Hydantoin Product 5

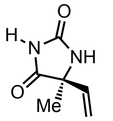

(*S*)-**25**
Chemical Formula: $C_6H_8N_2O_2$
Exact Mass: 140.0586
Molecular Weight: 140.1420

((***S***)-**25**): An oven or flame dried 25 mL round bottom flask equipped with a magnetic stirbar and rubber septum was connect to a double manifold via rubber tube and needle adapter and cooled under vacuum. Once cooled to rt, the flask was placed under $N_2$, the septum was removed, and **5** (87 mg, 0.32 mmol, 1 equiv) was added. The septum was replaced, and the flask was evacuated and backfilled with $N_2$ three times. Anhydrous MeCN (3.0 mL, 0.12 M) was then added via syringe, and the flask was cooled to 0 °C using an ice water bath. In a separate round bottom flask equipped with a stirbar, cerium ammonium nitrate (0.44 g, 0.84 mmol, 2.6 equiv) was dissolved in water (2.0 mL, 0.15 M). The cerium ammonium nitrate and water solution was then added dropwise via syringe, the ice water bath was removed, the reaction was warmed to rt, and the reaction stirred for 24 h. After the reaction was completed, the septum was removed, and the reaction mixture was transferred to a separatory funnel, diluted with brine (1 mL) and extracted with ethyl acetate (3 x 5 mL). The combined organic layers were then washed with brine (10 mL), dried with magnesium sulfate, and concentrated *in vacuo*. The crude material was purified via flash silica column chromatography (15:85 to 25:75 acetone:hexanes) to afford (*S*)-**25** as a white solid (42 mg, 94% yield).

(*S*)-**25** is a known compound. The acquired spectra are in accord with published data. Partial characterization is shown below. The optical rotation data for this compound corresponds with previous reports, allowing the absolute stereochemistry to be determined to be *S*.[23]

$^1$H NMR (600 MHz, $CDCl_3$) δ 8.23 (s, br, 1H), 6.03 (s, br, 1H), 5.95 (dd, *J* = 17.2, 10.5 Hz, 1H), 5.39 (d, *J* = 17.2 Hz, 1H), 5.30 (d, *J* = 10.5 Hz, 1H), 1.57 (s, 3H).

$^{13}$C NMR (600 MHz, $CDCl_3$) δ 175.2, 156.1, 135.9, 116.5, 64.7, 23.7.

Optical rotation: $[\alpha]_1^{22}$ = – 28.1° (1.0 mg/mL, EtOH) at 22 °C.

Optical rotation previously reported: $[\alpha]_1^{27}$ = – 52° (1.0 mg/mL, EtOH) at 27 °C.

## 10. Synthesis of (*R*)-Mephenytoin (29)

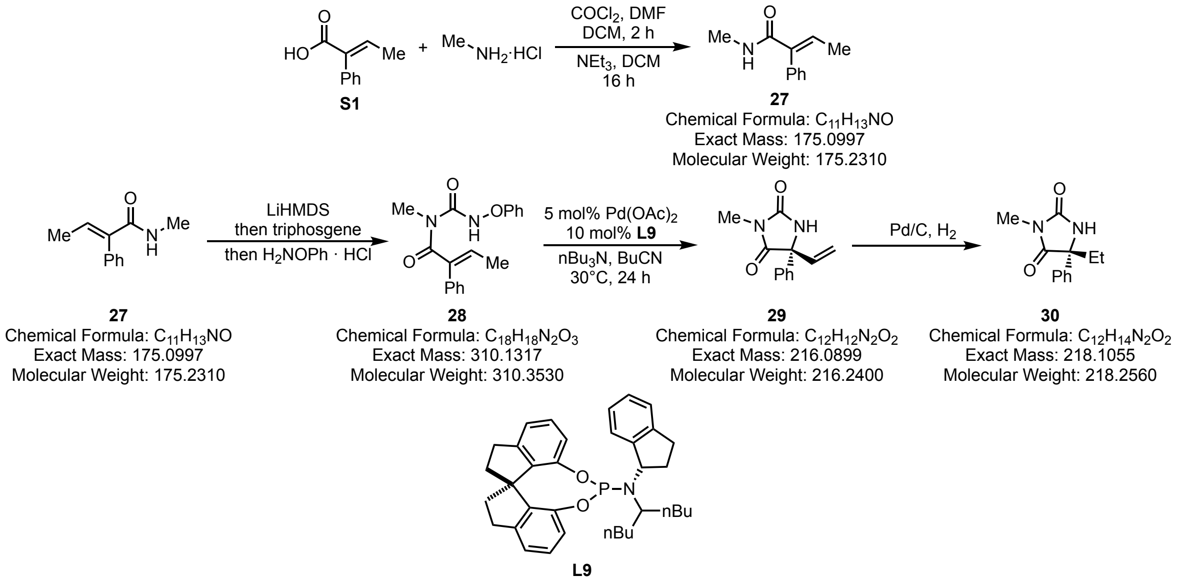


(**27**) According to **General Procedure B**, **S1** (8.5 g, 50 mmol, 1 equiv), DMF (0.2 mL, 2.5 mmol, 0.05 equiv), oxalyl chloride (5.1 mL, 60 mmol, 1.2 equiv), and $CH_2Cl_2$ (100 mL, 0.5 M) were added to a 250 mL round bottom flask and reacted at rt. After 2 h, methylamine hydrochloride (1.8 g, 26.4 mmol, 1.1 equiv), $Et_3N$ (10.5 mL, 75 mmol, 1.1 equiv), and $CH_2Cl_2$ (100 mL, 0.5 M) were added. After 16 h, the crude material was purified via flash silica gel chromatography (10:90 acetone:hexanes) to afford **27** as a yellow solid (1.7 g, 19% yield).

$^1H$ NMR (400 MHz, $CDCl_3$) δ 7.46–7.33 (m, 3H), 7.21–7.16 (m, 2H), 7.12 (q, *J* = 7.1 Hz, 1H), 5.30 (s, 1H), 2.80 (d, *J* = 4.9 Hz, 3H), 1.63 (d, *J* = 7.2 Hz, 3H).

$^{13}C$ NMR (101 MHz, $CDCl_3$) δ 167.5, 136.7, 136.1, 135.9, 130.0, 129.2, 128.2, 26.8, 15.1.

FTIR ($cm^{-1}$) 3441 (br), 3328 (br), 2938, 2361, 1734, 1663, 1519, 702.

HRMS (ESI) m/z, calculated for $[C_{11}H_{14}NO]^+$ ($[M+H]^+$): 176.1075, found: 176.1078.

MP = 70–73 °C.

(**28**) According to **General Procedure D**, **27** (1.5 g, 8.5 mmol, 1 equiv), LiHMDS (9.4 mL, 9.4 mmol, 1.1 equiv), triphosgene (2.5 g, 8.5 mmol, 1 equiv), and THF (43 mL, 0.2 M) were added to a 250 mL round bottom flask and reacted at rt. After 16 h, phenoxyamine hydrochloride (2.4 g, 17 mmol, 2 equiv), $Et_3N$ (3.6 mL, 25.5 mmol, 3 equiv), and THF (43 mL, 0.2 M) were added. After 16 h, the reaction was worked up according to General Procedure D and purified using flash silica gel chromatography (0:100 to 10:90 acetone:hexanes) to afford **S28** as a tan flakey solid (1.5 g, 58% yield).

$^1H$ NMR (600 MHz, $CDCl_3$) δ 11.64 (s, 1H), 7.46–7.41 (m, 2H), 7.37 (tt, *J* = 7.4, 1.3 Hz, 1H), 7.35–7.27 (m, 4H), 7.13 (t, *J* = 1.0 Hz, 1H), 7.11 (dd, *J* = 2.1, 1.0 Hz, 1H), 7.04 (tt, *J* = 7.4, 1.1 Hz, 1H), 6.26 (q, *J* = 7.1 Hz, 1H), 3.08 (s, 3H), 1.93 (d, *J* = 7.1 Hz, 3H).

$^{13}C$ NMR (151 MHz, $CDCl_3$) δ 174.7, 159.8, 156.1, 138.5, 133.4, 131.7, 129.6, 129.0, 128.9, 128.6, 123.1, 113.6, 34.3, 14.9.

FTIR ($cm^{-1}$) 3176 (br), 2959, 2853, 1706, 1662, 1592, 1490, 1450.

HRMS (ESI) m/z, calculated for $[C_{18}H_{19}N_2O_3]^+$ ($[M+H]^+$): 311.1396, found: 311.1398.

MP = 124–127 °C.

(**29**) According to **General Procedure E**, $Pd(OAc)_2$ (0.025 mmol, 5.5 mg), **L9** (0.05 mmol, 26.9 mg), **28** (0.5 mmol, 160 mg), $nBu_3N$ (2.5 mmol, 0.6 mL), and BuCN (2 mL, 0.25 M) were added to a 10 mL Schlenk tube and reacted for 24 h at 30 °C. The reaction was worked up according to General Procedure E and purified via flash column chromatography (10:90 acetone:hexanes) to afford **29** as a light yellow solid (74 mg, 69% yield).

91% ee. [Chiralpak OJ-3, MeOH/$CO_2$ = 5/95, v = 2.5 mL/min, λ = 254 nm, m/z = 217, t (major) = 3.12 min, t (minor) = 3.85 min].

$^1$H NMR (600 MHz, $CDCl_3$) δ 7.49–7.45 (m, 2H), 7.42–7.38 (m, 2H), 7.36 (appt tt, *J* = 7.1, 1.4 Hz, 1H), 6.29 (dd, *J* = 17.1, 10.4 Hz, 1H), 5.70 (br s, 1H), 5.48 (d, *J* = 17.1 Hz, 1H), 5.44 (d, *J* = 10.4 Hz, 1H), 3.05 (s, 3H).

$^{13}$C NMR (151 MHz, $CDCl_3$) δ 172.8, 156.4, 137.1, 135.6, 129.0, 128.8, 125.9, 117.0, 68.5, 25.2.

FTIR (cm$^{-1}$) 3291 (br), 2390, 2381, 1779, 1715, 1450, 1393.

HRMS (ESI) m/z, calculated for $[C_{12}H_{13}N_2O_2]^+$ ($[M+H]^+$): 217.0977, found: 217.0968.

MP = 81–84 °C.

Optical rotation: $[\alpha]_1^{21}$= – 27.7° (1 mg/mL in $CHCl_3$) at 21 °C.

(**30**) In a 10 mL round bottom flask equipped with a magnetic stirbar was added **28** (30 mg, 0.14 mmol, 1 equiv), MeOH (6 mL, 0.1 M) via syringe, and 10% weight Pd/C (1.2 mg, 0.01 mmol, 0.1 equiv) sequentially. The flask was capped with a rubber septum and placed under an $H_2$ filled balloon. A vent needle was added, and the $H_2$ was bubbled through the rection until the balloon was emptied. Once the reaction was complete, the vent needle, balloon, and rubber septum were removed. The reaction mixture was filtered through a pad of celite and washed with MeOH (3 x 10 mL). The filtrate was concentrated *in vacuo* to afford **30** as a white solid (29 mg, 97% yield).

**30** is a known compound. Acquired spectra are in accord with published data. Partial characterization is shown below. The optical rotation data for compound **30** corresponds to previous reports, allowing the absolute stereochemistry to be determined to be *R*.[24]

$^1$H NMR (600 MHz, $CDCl_3$) δ 7.55 (dt, *J* = 7.9, 2.0 Hz, 2H), 7.39 (t, *J* = 7.6 Hz, 2H), 7.33 (t, *J* = 7.4 Hz, 1H), 6.99–6.53 (m, 1H), 3.01 (s, 3H), 2.24 (dq, *J* = 14.5, 6.8 Hz, 1H), 2.12 (dq, *J* = 14.7, 7.4 Hz, 1H), 0.90 (t, *J* = 7.4 Hz, 3H).

$^{13}$C NMR (151 MHz, $CDCl_3$) δ 175.0, 159.4, 137.9, 128.9, 128.6, 125.5, 42.1, 32.2, 24.9, 8.2.

Optical rotation: $[\alpha]_1^{21}$= – 83.8° (1 mg/mL in EtOH) at 21 °C.

Optical rotation previously reported: $[\alpha]_1^{21}$= – 71.4° (1.4 mg/mL in EtOH) at 21 °C.

## 11. Preparation of Unprotected Substrate

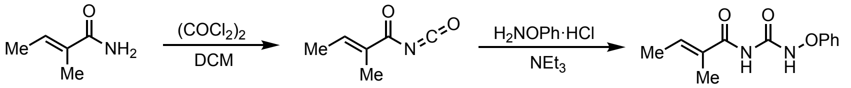


**S59**
Chemical Formula: $C_6H_7NO_2$
Exact Mass: 125.0477
Molecular Weight: 125.1270

**1**-*S*-trans
Chemical Formula: $C_{12}H_{14}N_2O_3$
Exact Mass: 234.1004
Molecular Weight: 234.2550

(**S59**) An oven dried round bottom flask equipped with a reflux condenser, a magnetic stirbar, and a rubber septum connected to a double manifold via rubber tube and needle and cooled under vacuum. Once cooled to rt, the flask was placed under $N_2$, the septum was removed, and (*E*)-2-methyl-2-butenamide (0.3 g, 3 mmol, 1 equiv) was added. The septum was replaced, and the flask was evacuated and backfilled with $N_2$ three times. Anhydrous $CH_2Cl_2$ (24 mL, 0.12 M) was then added via syringe, and the flask was cooled to 0 °C using an ice water bath. Once cooled, oxalyl chloride (0.77 mL, 9 mmol, 3 equiv) was added dropwise via syringe. The reaction flask was then moved to a preheated external oil bath at 55 °C. After stirring overnight, the reaction was removed from the oil bath, cooled to rt, diluted with $Et_2O$ (10 mL), and directly concentrated *in vacuo* to afford **S59** as a red-brown liquid. The crude residue was used directly in the next step without purification. $^1$H NMR was taken for identification purposes.

$^1$H NMR (600 MHz, $CDCl_3$) δ 7.07 (qq, *J* = 7.1, 1.4 Hz, 1H), 1.88 (dq, *J* = 7.1, 1.2 Hz, 3H), 1.83 (quint, *J* = 1.2 Hz, 3H).

(**1-*S*-trans**) **1-*S*-trans** was synthesized using a modified **General Procedure C**. An oven dried round bottom flask equipped with a magnetic stirbar and rubber septum was connected to a double manifold via rubber tube and needle and cooled under vacuum to rt. Once cooled, the flask was placed under $N_2$, the septum was removed and phenoxyamine hydrochloride (6.0 mmol, 874 mg, 2 equiv) was added. The septum was replaced, and the flask was evacuated and backfilled with $N_2$ three times. Anhydrous THF (15 mL, 0.4 M) was added via syringe, followed by $NEt_3$ (1.26 mL, 9.0 mmol, 3 equiv) dropwise via syringe at rt. The reaction was left to stir at rt. After 1 h, **S59** (375 mg, 3.0 mmol, 1 equiv) was added via syringe. After 16 h, the septum was removed, and the reaction was directly concentrated *in vacuo* onto 1 g of celite. Purification using flash silica column chromatography (10:90 to 50:50 ethyl acetate:hexanes) then afforded **1-*S*-trans** as a tan solid (317 mg, 45% yield).

$^1$H NMR (400 MHz, $CDCl_3$) δ 11.18 (s, 1H), 8.33 (s, 1H), 7.36–7.28 (m, 2H), 7.16–7.09 (m, 2H), 7.09–7.02 (m, 1H), 6.68 (qd, *J* = 6.9, 1.5 Hz, 1H), 1.88 (s, 3H), 1.79 (d, *J* = 7.0 Hz, 3H).

$^{13}$C NMR (101 MHz, $CDCl_3$) δ 168.9, 159.7, 154.2, 137.0, 130.3, 129.6, 123.2, 113.3, 14.7, 12.2.

FTIR (cm$^{-1}$) 3289 (br), 2349, 1700, 1665, 1642, 1592, 1491, 1474, 1389.

HRMS (ESI) m/z, calculated for $[C_{12}H_{15}N_2O_3]^+$ ($[M+H]^+$): 235.1083, found: 235.1086.

MP = 109–112 °C.

## 12. Preparation of N–Me Substrate and Cyclization

### 12.1 Synthesis of N–Me Starting Material

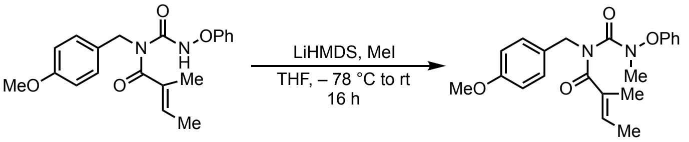


**4**

**S60**
Chemical Formula: $C_{21}H_{24}N_2O_4$
Exact Mass: 368.1736
Molecular Weight: 368.4330

(**S60**) A dried round bottom flask, equipped with a magnetic stirbar and a rubber septum, was connected hot to a double manifold via a rubber hose and needle, and cooled under vacuum to rt. Once cooled, the flask was backfilled with $N_2$, the septum was quickly removed, **4** (1.8 g, 5 mmol, 1 equiv) was added, and the septum was replaced. The flask was evacuated and backfilled with $N_2$ four times. Anhydrous THF (25 mL, 0.2 M) was then added via syringe, and the flask was cooled to – 78 °C in a dry ice/acetone bath. Once cooled, LiHMDS (5.5 mL, 1 M in THF, 5.5 mmol, 1.1 equiv) was added via syringe dropwise, and the mixture was stirred at – 78 °C for 5 min. Methyl iodide (0.4 mL, 6 mmol, 1.2 equiv) was added dropwise via syringe, and the flask was allowed to warm to rt. The reaction stirred at rt for 16 h. The septum was removed and the reaction was directly concentrated *in vacuo* onto 1 g of celite. Purification using flash silica gel chromatography (0:100 to 10:90 acetone:hexanes) afforded **S60** as a colorless oil (0.88 g, 48% yield).

$^1$H NMR (600 MHz, $CDCl_3$) δ 7.20 – 7.16 (m, 2H), 7.13 (td, *J* = 7.2, 2.2 Hz, 2H), 6.98 (t, *J* = 7.4 Hz, 1H), 6.66 – 6.61 (m, 2H), 6.56 (dd, *J* = 9.0, 2.5 Hz, 2H), 6.08 – 6.01 (m, 1H), 4.68 (s, 2H), 3.75 (s, 3H), 3.19 (s, 3H), 1.99 (s, 3H), 1.77 (d, *J* = 6.9 Hz, 3H).

$^{13}$C NMR (151 MHz, $CDCl_3$) δ 172.8, 159.3, 159.1, 157.3, 135.7, 130.9, 130.6, 129.7, 129.6, 123.4, 113.8, 113.2, 55.3, 49.1, 37.2, 14.1, 13.6.

FTIR (cm$^{-1}$) 2950, 1718, 1684, 1418, 1110, 770, 532.

HRMS (ESI) m/z, calculated for $[C_{21}H_{25}N_2O_4]^+$: 369.1815, found: 369.1823.

### 12.2 Cyclization of N–Me Substrate

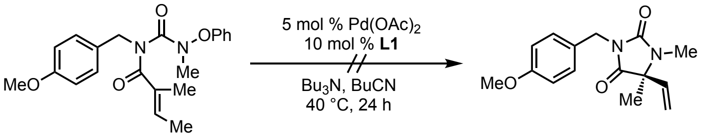


**S60**
Chemical Formula: $C_{21}H_{24}N_2O_4$
Exact Mass: 368.1736
Molecular Weight: 368.4330

0%
>90% SM

In a $N_2$-filled glovebox, $Pd(OAc)_2$ (1.1 mg, 5 mol %, 0.05 equiv), ligand (5.7 mg, 0.1 mmol, 0.1 equiv), and **S60** (36.8 mg, 0.1 mmol, 1 equiv) were added to an oven-dried 1-dram vial containing an 8 mm Teflon-coated stirbar. Anhydrous BuCN (0.4 mL, 0.25 M) and $nBu_3N$ (119 uL, 0.5 mmol, 5 equiv) were added, and the vial was capped. The mixture was stirred at 40 °C inside the glovebox in a preheated aluminum-block set atop a stirplate. After 24 h, the reaction vial was removed from the glovebox, cooled to rt, and concentrated *in vacuo*. Yields were obtained by $^1$H NMR of the crude product compared to an internal standard (1,3,5-trimethoxybenzene). No cyclized product of **S60** was observed by $^1$H NMR and LC-MS.

## 13. Characterization and Analysis of Trichloromethyl Carbamates

In all cases where trichloromethyl carbamates were isolated, they were initially isolated as a kinetic mixture of two stable conformers. These conformers had nearly identical spectra properties, and could be separated from each other in some cases using column chromatography. Despite extensive efforts using 1D and 2D NMR methods (see spectra), we were able to deduce that the products had the same connectivity but we were unable to assign the conformation of each product. However, in all cases studies, we have found that upon heating the two conformers they converge to a single thermodynamically stable conformation. The mixtures also converge when reacted with phenoxyamine to give a single product. In the procedures above, where the trichloromethyl carbamates were isolated, we provided spectral data for the mixture of conformers and carried them forward without complete characterization. Below, however, we provide additional details for the trichloromethyl carbamates (**S8**) arising from amide **4.**

### 13.1 Isolation and Characterization of Trichloromethyl Carbamates

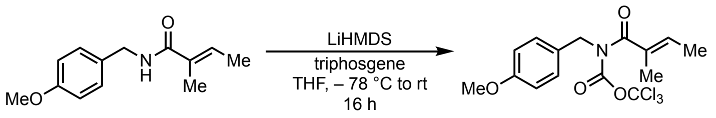


**3** **S8**

(**S8**) According to **General Procedure C**, **3** (1.1 g, 5 mmol, 1 equiv), LiHMDS (5.5 mL, 5.5 mmol, 1.1 equiv), triphosgene (1.5 g, 5 mmol, 1 equiv), and THF (25 mL, 0.2 M) were added to a 250 mL round bottom flask and reacted at rt for 16 h. The reaction was worked up according to general procedure C and purified using preparatory TLC (10:90 ethyl acetate:hexanes) to provide the isolated carbamate conformers as single products.

Data for **S8 conformer 1**:

$^{1}$H NMR (400 MHz, $CDCl_3$) δ 7.33–7.25 (m, 2H), 6.93–6.81 (m, 2H), 6.19 (qq, *J* = 7.0, 1.4 Hz, 1H), 4.87 (s, 2H), 3.79 (s, 3H), 1.85 (quint, *J* = 1.1 Hz, 3H), 1.74 (dq, *J* = 7.0, 1.1 Hz, 3H).

$^{13}$C NMR (151 MHz, $CDCl_3$) δ 173.8, 159.6, 148.8, 137.9, 134.3, 130.5, 130.3, 127.7, 114.2, 55.5, 51.9, 14.7, 12.8.

Data for **S8 conformer 2**:

$^{1}$H NMR (400 MHz, $CDCl_3$) δ 7.38–7.31 (m, 2H), 6.94–6.80 (m, 2H), 6.00 (qq, *J* = 7.0, 1.5 Hz, 1H), 4.82 (s, 2H), 3.79 (s, 3H), 1.89 (quint, *J* = 1.2 Hz, 3H), 1.74 (dquint, *J* = 7.0, 1.1 Hz, 4H).

$^{13}$C NMR (151 MHz, $CDCl_3$) δ 173.5, 159.5, 147.9, 137.9, 134.7, 134.1, 130.4, 128.3, 114.1, 55.4, 49.2, 14.1, 13.1.

### 13.2 Equilibration of Trichloromethyl Carbamate Conformers to a Single Product

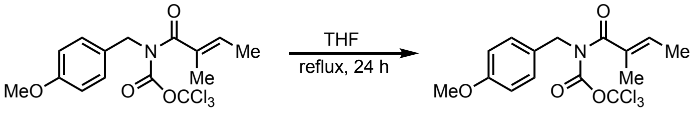


**S8**
*as a 50:50 mixture of conformers*

**S8** as a single conformer

(**S8 as a single conformer**) An oven dried 50 mL round bottom flask equipped with a reflux condenser, magnetic stirbar, and rubber septum was connected to a double manifold via rubber tube and needle and cooled under vacuum. Once cooled to rt, the flask was evacuated and backfilled with $N_2$ three times. Then **S8** as a mixture of conformers (0.88 g, 2.3 mmol, 1 equiv) was added in a solution of anhydrous THF (12 mL) via syringe. The round bottom flask was placed into a preheated oil bath and refluxed for 24 h. The reaction mixture was cooled to rt and directly concentrated *in vacuo* to afford **S8** as a single isomer (0.87 g, 99% yield). $^{1}$H NMR spectra for the mixture of conformers and after thermal conversion to a single conformer are provided below. The top spectrum is the mixture of conformers. The bottom spectrum is **S8** as a single conformer.

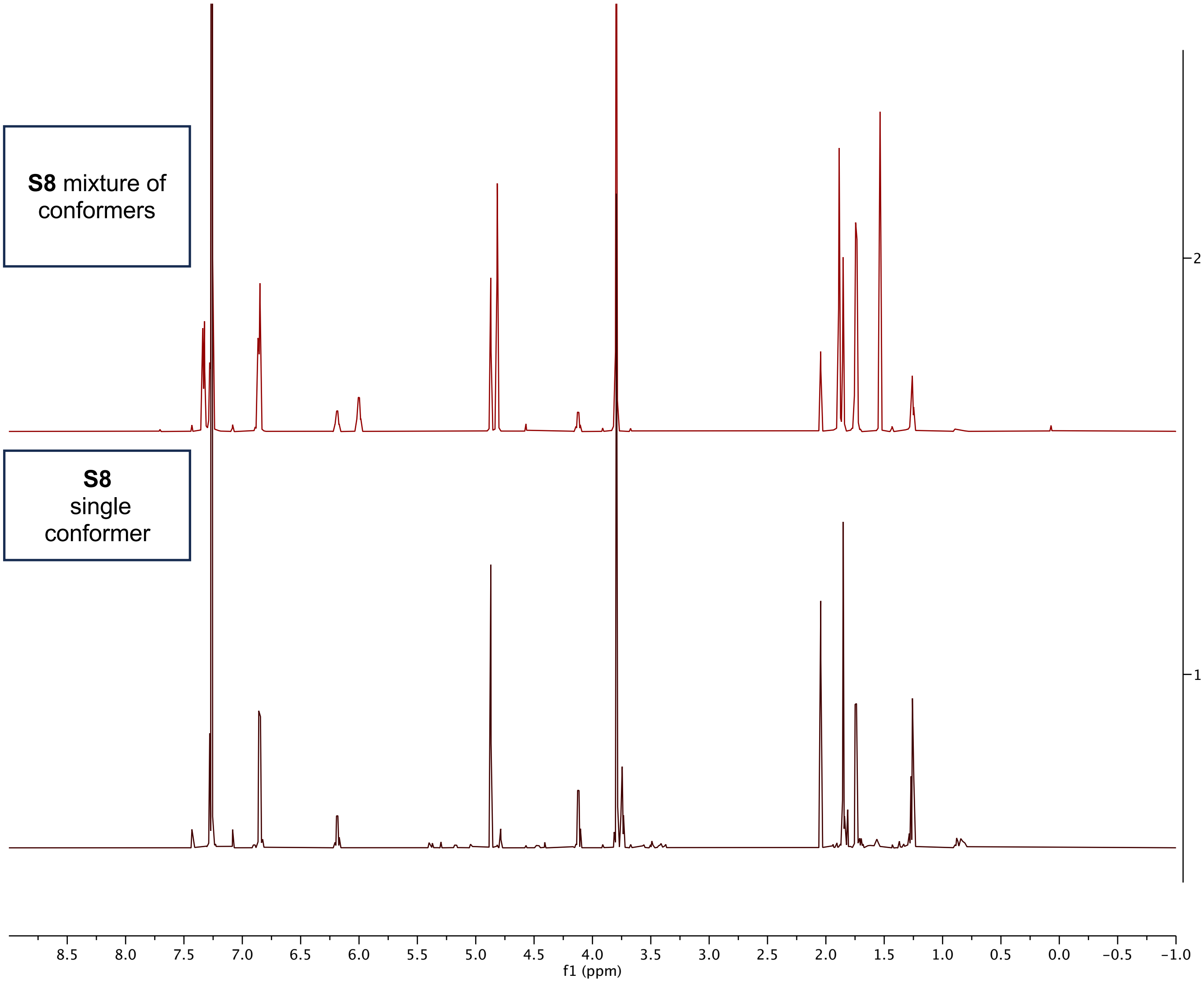

## 14. References

(1) Pangborn, A. B.; Giardello, M. A.; Grubbs, R. H.; Rosen, R. K.; Timmers, F. J. Safe and Convenient Procedure for Solvent Purification. *Organometallics* **1996**, *15*, 1518-1520.
(2) Beak, P.; Kempf, D. J.; Wilson, K. D. The β-Lithiation of α,β-unsaturated amides. *Journal of the American Chemical Society* **1985**, *107*, 4745-4756.
(3) Banik, S. M.; Medley, J. W.; Jacobsen, E. N. Catalytic, Diastereoselective 1,2-Difluorination of Alkenes. *Journal of the American Chemical Society* **2016**, *138*, 5000-5003.
(4) Ramachandran, P. V.; Burghardt, T. E.; Reddy, M. V. R. Preparation of N-protected allylic amines and α-methylene-β-amino acids from vinylalumination/Baylis–Hillman products via tandem SN2′ substitution–Overman rearrangement. *Tetrahedron Letters* **2005**, *46*, 2121-2124.
(5) Nogi, K.; Fujihara, T.; Terao, J.; Tsuji, Y. Cobalt- and Nickel-Catalyzed Carboxylation of Alkenyl and Sterically Hindered Aryl Triflates Utilizing CO2. *The Journal of Organic Chemistry* **2015**, *80*, 11618-11623.
(6) S.A., C. P. 3-Phenyl-4-Hexynoic acid derivatives as GPR40 Agonists. 2019.
(7) AG, S. 2017.
(8) Gao, Z.; Fletcher, S. P. Construction of β to carbonyl stereogenic centres by asymmetric 1,4-addition of alkylzirconocenes to dienones and ynenones. *Chemical Communications* **2018**, *54*, 3601-3604.
(9) Gaussian 16, R. C., Frisch, M. J.; Trucks, G. W.; Schlegel, H. B.; Scuseria, G. E.; Robb, M. A.; Cheeseman, J. R.; Scalmani, G.; Barone, V.; Petersson, G. A.; Nakatsuji, H.; Li, X.; Caricato, M.; Marenich, A. V.; Bloino, J.; Janesko, B. G.; Gomperts, R.; Mennucci, B.; Hratchian, H. P.; Ortiz, J. V.; Izmaylov, A. F.; Sonnenberg, J. L.; Williams-Young, D.; Ding, F.; Lipparini, F.; Egidi, F.; Goings, J.; Peng, B.; Petrone, A.; Henderson, T.; Ranasinghe, D.; Zakrzewski, V. G.; Gao, J.; Rega, N.; Zheng, G.; Liang, W.; Hada, M.; Ehara, M.; Toyota, K.; Fukuda, R.; Hasegawa, J.; Ishida, M.; Nakajima, T.; Honda, Y.; Kitao, O.; Nakai, H.; Vreven, T.; Throssell, K.; Montgomery, J. A., Jr.; Peralta, J. E.; Ogliaro, F.; Bearpark, M. J.; Heyd, J. J.; Brothers, E. N.; Kudin, K. N.; Staroverov, V. N.; Keith, T. A.; Kobayashi, R.; Normand, J.; Raghavachari, K.; Rendell, A. P.; Burant, J. C.; Iyengar, S. S.; Tomasi, J.; Cossi, M.; Millam, J. M.; Klene, M.; Adamo, C.; Cammi, R.; Ochterski, J. W.; Martin, R. L.; Morokuma, K.; Farkas, O.; Foresman, J. B.; Fox, D. J. . *Gaussian, Inc., Wallingford CT* **2016**.
(10) Legault, C. Y. *CYLview, version 1.0b; Universitede Sherbrooke: Quebec, Canada* **2009**.
(11) (a) Becke, A. D. Density-functional thermochemistry. III. The role of exact exchange. *Journal of Chemical Physics* **1993**, *98*, 5648-5652. (b) Lee, C.; Yang, W.; Parr, R. G. Development of the Colle-Salvetti correlation-energy formula into a functional of the electron density. *Physical Review B* **1988**, *37*, 785-789.
(12) (a) Zhao, Y.; Truhlar, D. G. Density Functionals with Broad Applicability in Chemistry. *Accounts of Chemical Research* **2008**, *41*, 157-167. (b) Zhao, Y.; Truhlar, D. G. The M06 suite of density functionals for main group thermochemistry, thermochemical kinetics, noncovalent interactions, excited states, and transition elements: two new functionals and systematic testing of four M06-class functionals and 12 other functionals. *Theoretical Chemistry Accounts* **2008**, *120*, 215-241.
(13) Marenich, A. V.; Cramer, C. J.; Truhlar, D. G. Universal Solvation Model Based on Solute Electron Density and on a Continuum Model of the Solvent Defined by the Bulk Dielectric Constant and Atomic Surface Tensions. *The Journal of Physical Chemistry B* **2009**, *113*, 6378-6396.
(14) Dalcanale, E.; Montanari, F. Selective oxidation of aldehydes to carboxylic acids with sodium chlorite-hydrogen peroxide. *The Journal of Organic Chemistry* **1986**, *51*, 567-569.
(15) Hou, J.; Ee, A.; Feng, W.; Xu, J.-H.; Zhao, Y.; Wu, J. Visible-Light-Driven Alkyne Hydro-/Carbocarboxylation Using CO2 via Iridium/Cobalt Dual Catalysis for Divergent Heterocycle Synthesis. *Journal of the American Chemical Society* **2018**, *140*, 5257-5263.
(16) Sellès, P. Synthesis and Biological Evaluation of Himanimide C and Unnatural Analogues. *Organic Letters* **2005**, *7*, 605-608.
(17) Edwards Jr, J. D.; Matsumoto, T.; Hase, T. Senecio alkaloids. Synthesis of sarracinic acid. *The Journal of Organic Chemistry* **1967**, *32*, 244-246.
(18) Clayman, P. D.; Hyster, T. K. Photoenzymatic Generation of Unstabilized Alkyl Radicals: An Asymmetric Reductive Cyclization. *Journal of the American Chemical Society* **2020**, *142*, 15673-15677.
(19) Fujimoto, H.; Nakayasu, B.; Tobisu, M. Synthesis of γ-Lactams from Acrylamides by Single-Carbon Atom Doping Annulation. *Journal of the American Chemical Society* **2023**, *145*, 19518-19522.
(20) Reeves, D. C.; Rodriguez, S.; Lee, H.; Haddad, N.; Krishnamurthy, D.; Senanayake, C. H. Palladium Catalyzed Alkoxy- and Aminocarbonylation of Vinyl Tosylates. *Organic Letters* **2011**, *13*, 2495-2497.

(21) Guo, Y.; Mao, X.; Xiong, L.; Xia, A.; You, J.; Lin, G.; Wu, C.; Huang, L.; Wang, Y.; Yang, S. Structure-guided discovery of a potent and selective cell-active inhibitor of SETDB1 tudor domain. *Angewandte Chemie International Edition* **2021**, *60*, 8760-8765.
(22) Gualtierotti, J.-B.; Schumacher, X.; Fontaine, P.; Masson, G.; Wang, Q.; Zhu, J. Amidation of Aldehydes and Alcohols through α-Iminonitriles and a Sequential Oxidative Three-Component Strecker Reaction/Thio-Michael Addition/Alumina-Promoted Hydrolysis Process to Access β-Mercaptoamides from Aldehydes, Amines, and Thiols. *Chemistry – A European Journal* **2012**, *18*, 14812-14819.
(23) Achiha, K.; Terashima, T.; Mizuno, H.; Takamura, N.; Kitagawa, T.; Ishikawa, K.; Yamada, S. Studies on optically Active Amino Acids. XVIII. Studies on α-Methyl-α-amino Acids. XIV. Several Optical Properties of α-Methyl-α-amino Acids. *Chemical & Pharmaceutical Bulletin* **1970**, *18*, 61-74.
(24) Atkinson, R. C.; Fernández-Nieto, F.; Mas Roselló, J.; Clayden, J. Pseudoephedrine-Directed Asymmetric α-Arylation of α-Amino Acid Derivatives. *Angewandte Chemie International Edition* **2015**, *54*, 8961-8965.

## 15. Spectral Data

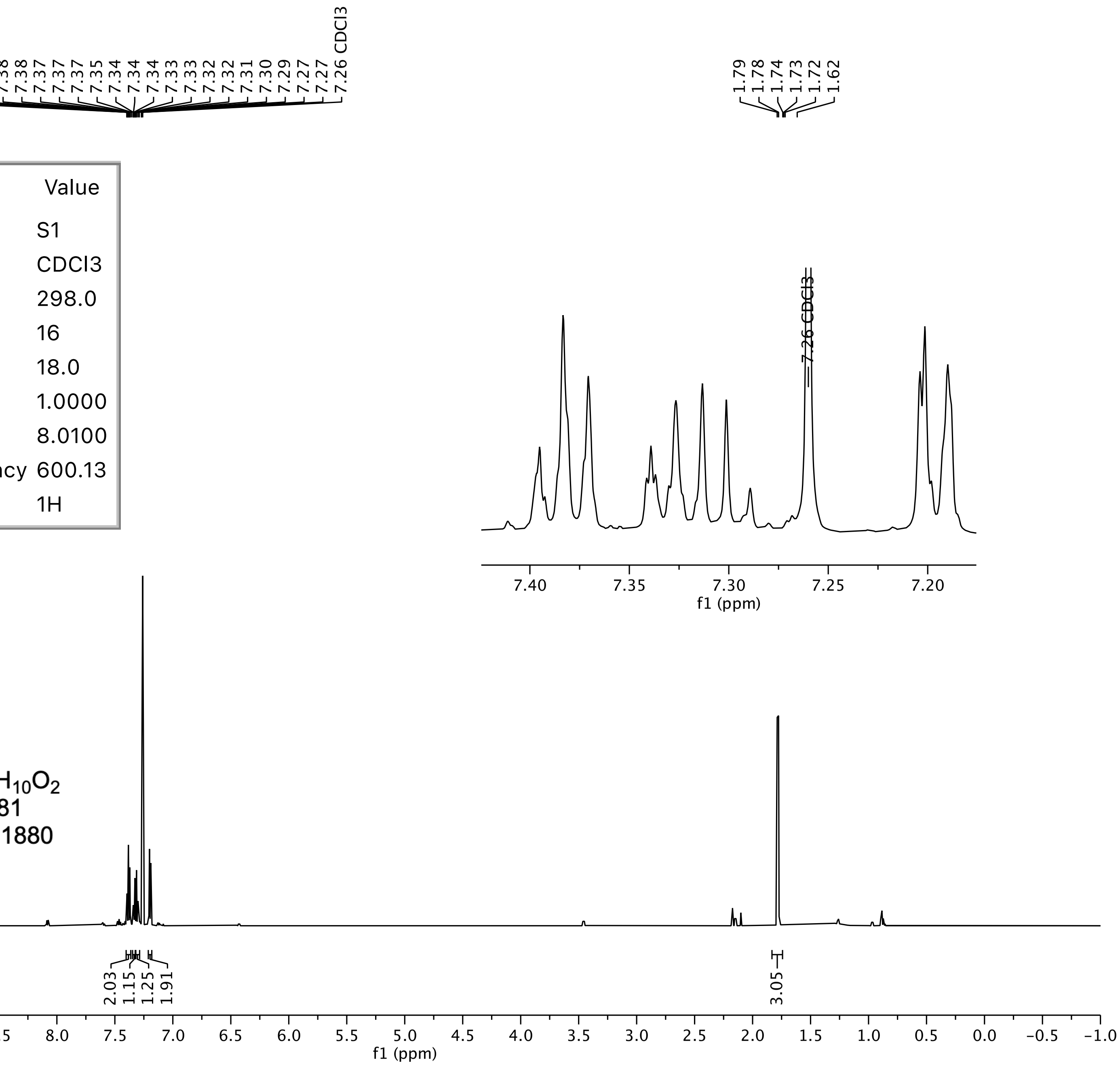


| Parameter | Value |
|---|---|
| 1 Title | S1 |
| 2 Solvent | CDCl3 |
| 3 Temperature | 298.0 |
| 4 Number of Scans | 16 |
| 5 Receiver Gain | 18.0 |
| 6 Relaxation Delay | 1.0000 |
| 7 Pulse Width | 8.0100 |
| 8 Spectrometer Frequency | 600.13 |
| 9 Nucleus | 1H |

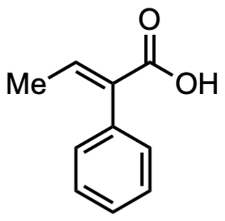


**S1**

Chemical Formula: $C_{10}H_{10}O_2$

Exact Mass: 162.0681

Molecular Weight: 162.1880

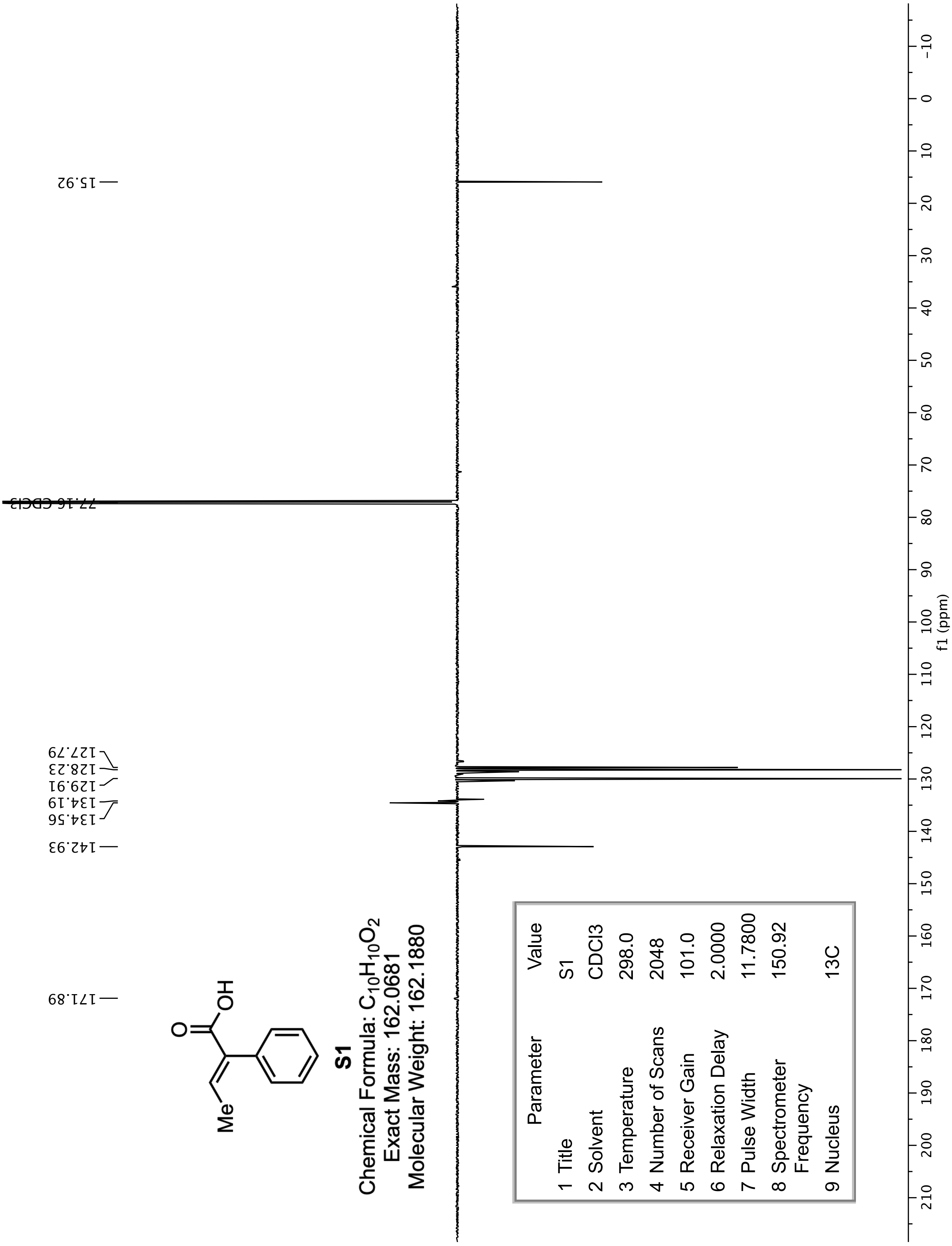


| Parameter | Value |
|---|---|
| 1 Title | S1 |
| 2 Solvent | CDCl3 |
| 3 Temperature | 298.0 |
| 4 Number of Scans | 2048 |
| 5 Receiver Gain | 101.0 |
| 6 Relaxation Delay | 2.0000 |
| 7 Pulse Width | 11.7800 |
| 8 Spectrometer Frequency | 150.92 |
| 9 Nucleus | 13C |

| Parameter | Value |
|---|---|
| 1 Title | S2 for identification purposes only |
| 2 Solvent | CDCl3 |
| 3 Temperature | 300.0 |
| 4 Number of Scans | 16 |
| 5 Receiver Gain | 101.0 |
| 6 Relaxation Delay | 1.0000 |
| 7 Pulse Width | 11.1500 |
| 8 Spectrometer Frequency | 600.32 |
| 9 Nucleus | 1H |

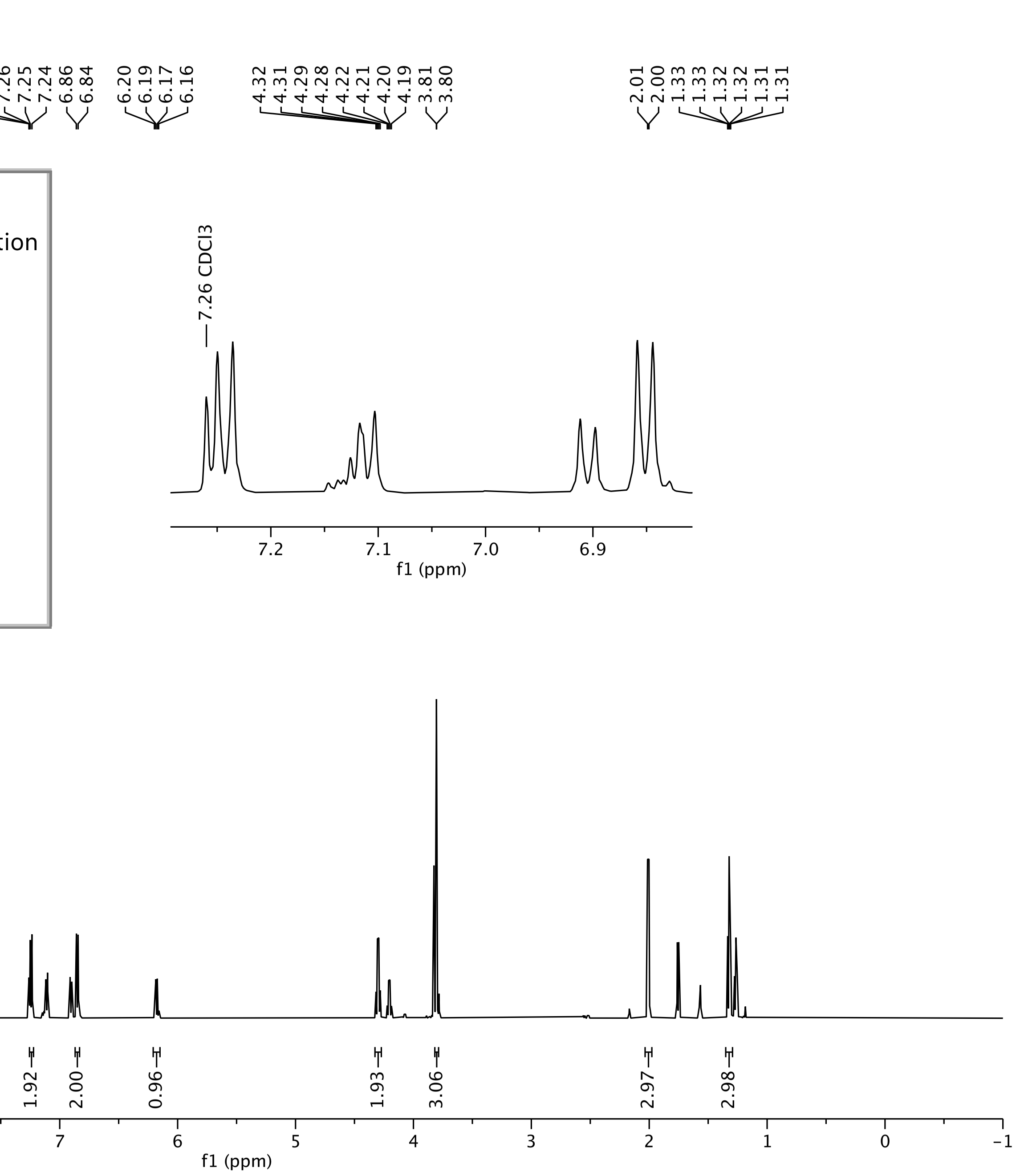


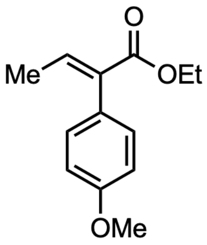


**S2** for identification purposes only
Chemical Formula: $C_{13}H_{16}O_3$
Exact Mass: 220.1099
Molecular Weight: 220.2680

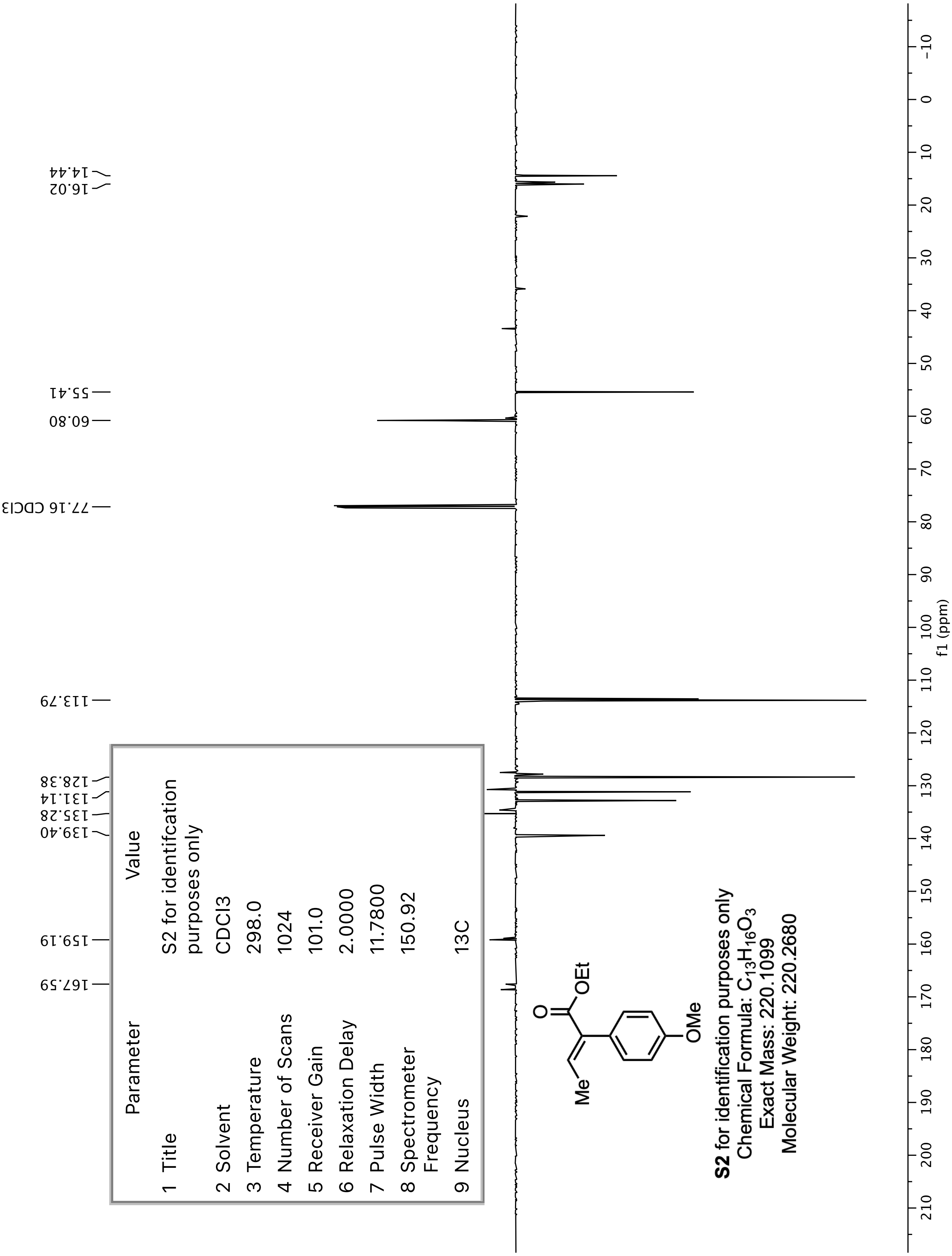
167.59
159.19
139.40
135.28
131.14
128.38
113.79
77.16 CDCl3
60.80
55.41
16.02
14.44
Parameter
Value
1 Title
S2 for identifcation purposes only
2 Solvent
CDCl3
3 Temperature
298.0
4 Number of Scans
1024
5 Receiver Gain
101.0
6 Relaxation Delay
2.0000
7 Pulse Width
11.7800
8 Spectrometer Frequency
150.92
9 Nucleus
13C
Me
O
OEt
OMe
S2 for identification purposes only
Chemical Formula: C13H16O3
Exact Mass: 220.1099
Molecular Weight: 220.2680
f1 (ppm)

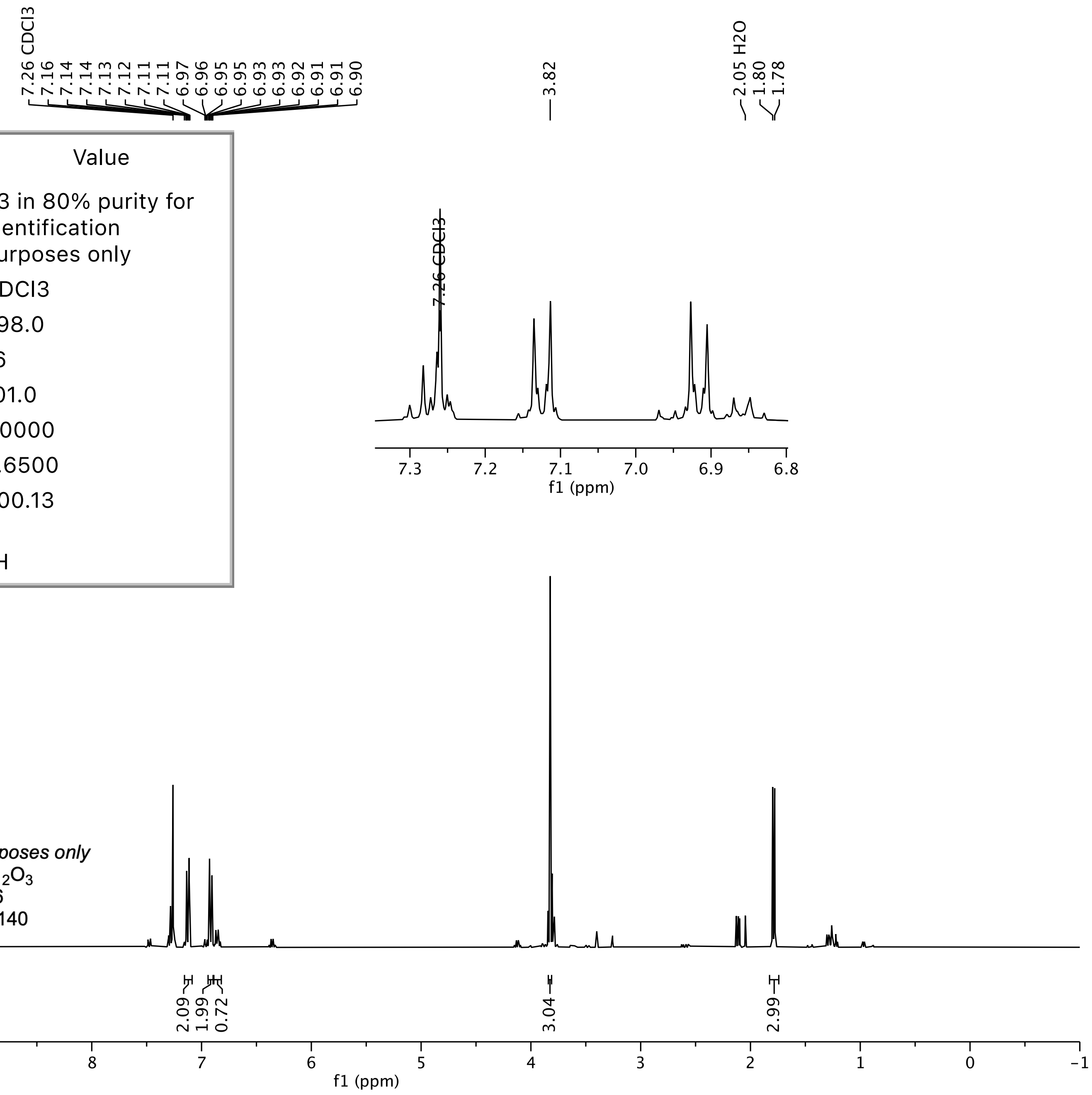


| | Parameter | Value |
|---|---|---|
| 1 | Title | S3 in 80% purity for identification purposes only |
| 2 | Solvent | CDCl3 |
| 3 | Temperature | 298.0 |
| 4 | Number of Scans | 16 |
| 5 | Receiver Gain | 101.0 |
| 6 | Relaxation Delay | 1.0000 |
| 7 | Pulse Width | 8.6500 |
| 8 | Spectrometer Frequency | 400.13 |
| 9 | Nucleus | 1H |

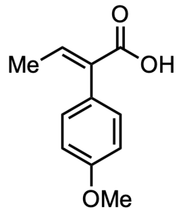


**S3**

*80% purity; for identification purposes only*

Chemical Formula: $C_{11}H_{12}O_3$

Exact Mass: 192.0786

Molecular Weight: 192.2140

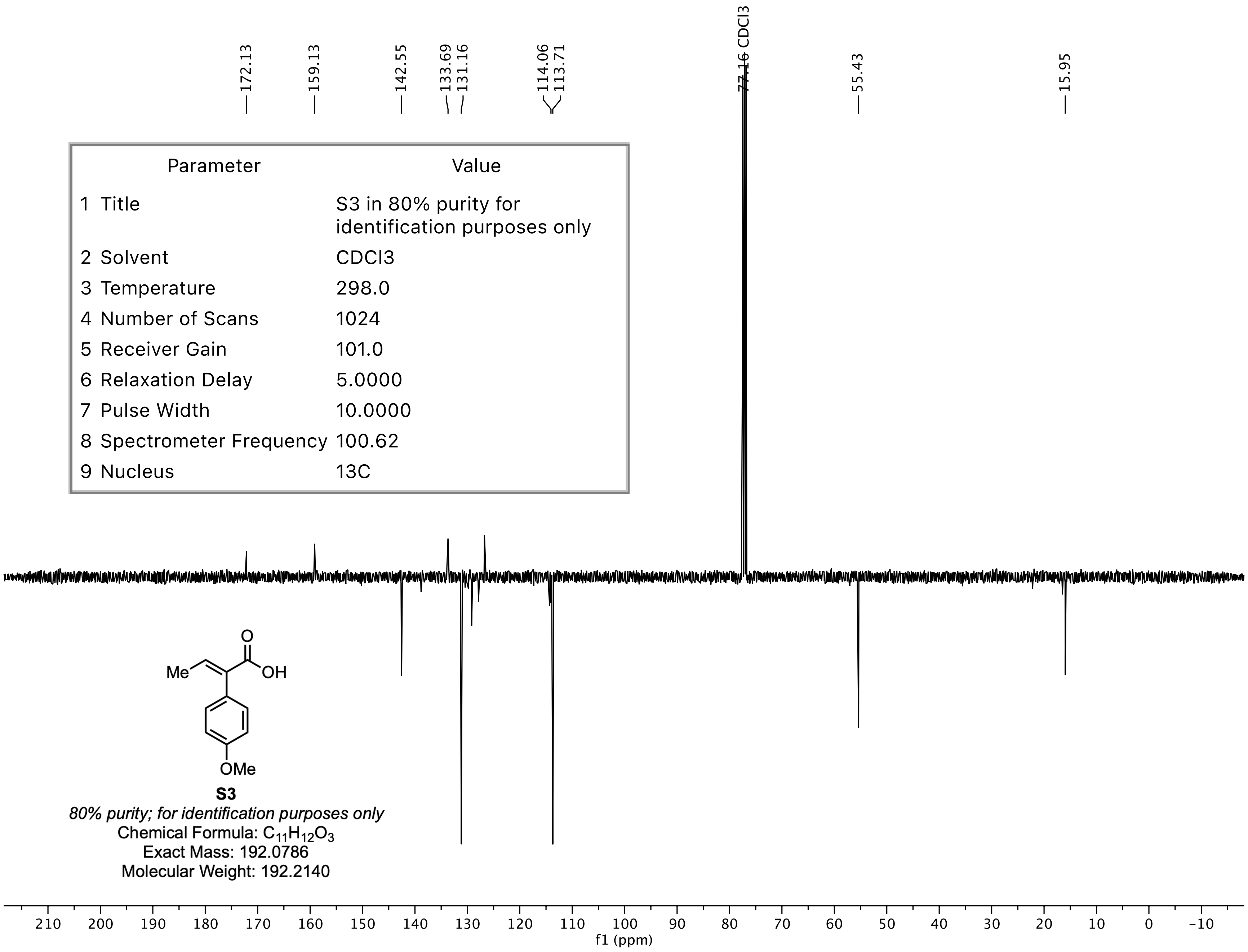


| | Parameter | Value |
|---|---|---|
| 1 | Title | S3 in 80% purity for identification purposes only |
| 2 | Solvent | CDCl3 |
| 3 | Temperature | 298.0 |
| 4 | Number of Scans | 1024 |
| 5 | Receiver Gain | 101.0 |
| 6 | Relaxation Delay | 5.0000 |
| 7 | Pulse Width | 10.0000 |
| 8 | Spectrometer Frequency | 100.62 |
| 9 | Nucleus | 13C |

**S3**

*80% purity; for identification purposes only*

Chemical Formula: $C_{11}H_{12}O_3$

Exact Mass: 192.0786

Molecular Weight: 192.2140

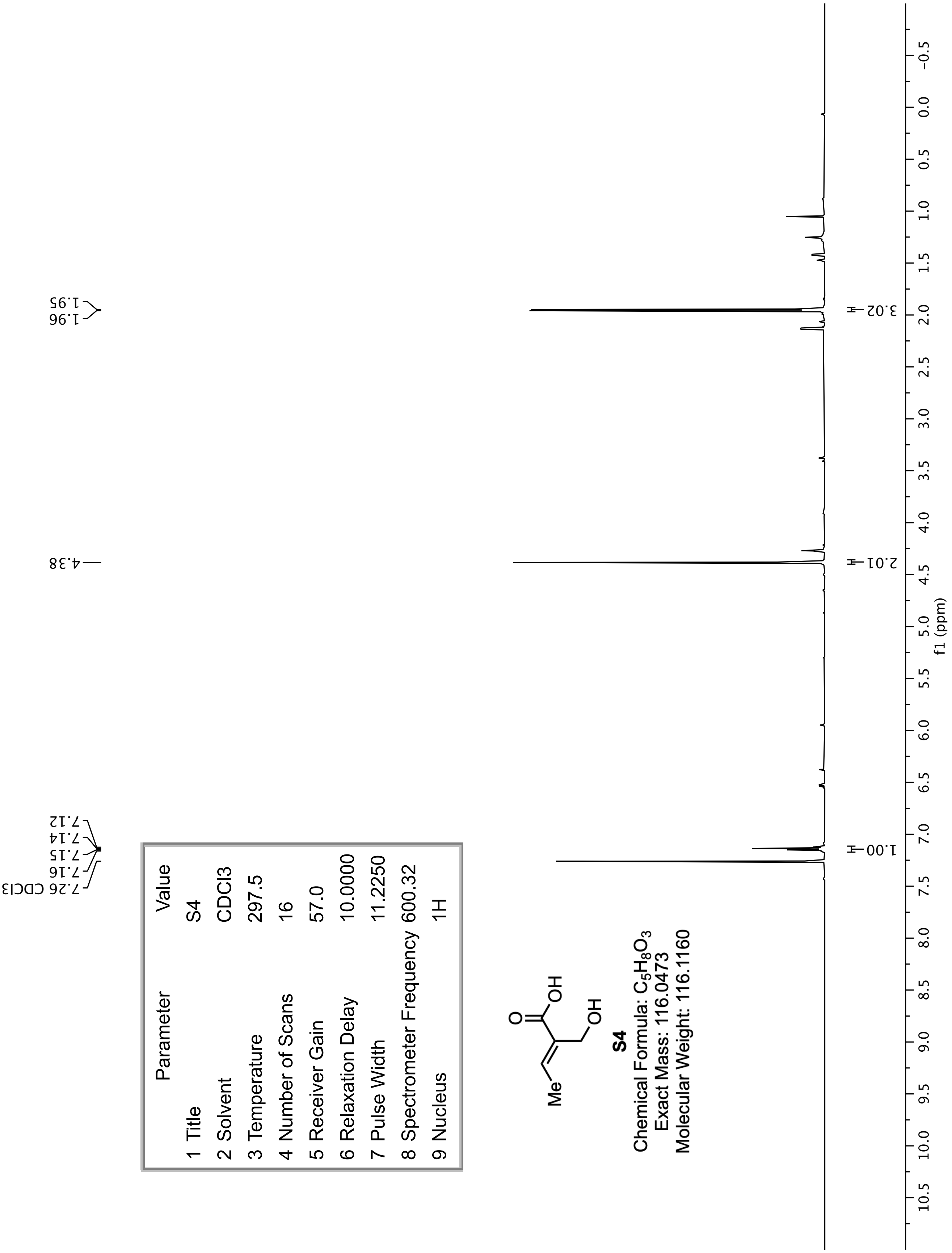


| Parameter | Value |
|---|---|
| 1 Title | S4 |
| 2 Solvent | CDCl3 |
| 3 Temperature | 297.5 |
| 4 Number of Scans | 16 |
| 5 Receiver Gain | 57.0 |
| 6 Relaxation Delay | 10.0000 |
| 7 Pulse Width | 11.2250 |
| 8 Spectrometer Frequency | 600.32 |
| 9 Nucleus | 1H |

**S4**

Chemical Formula: $C_5H_8O_3$
Exact Mass: 116.0473
Molecular Weight: 116.1160

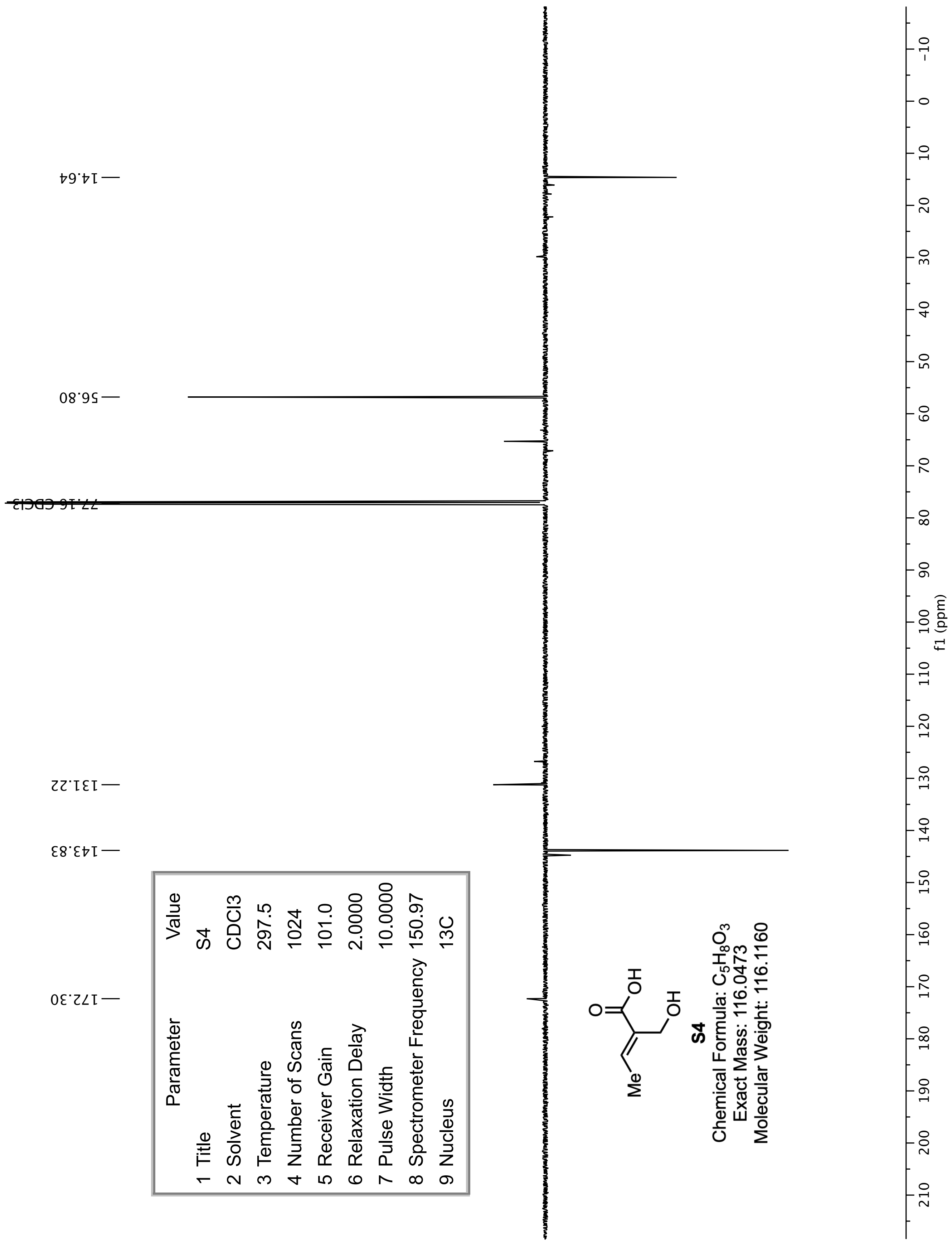


| | Parameter | Value |
|---|---|---|
| 1 | Title | S4 |
| 2 | Solvent | CDCl3 |
| 3 | Temperature | 297.5 |
| 4 | Number of Scans | 1024 |
| 5 | Receiver Gain | 101.0 |
| 6 | Relaxation Delay | 2.0000 |
| 7 | Pulse Width | 10.0000 |
| 8 | Spectrometer Frequency | 150.97 |
| 9 | Nucleus | 13C |

| Parameter | Value |
| --- | --- |
| 1 Title | S5, crude for identification purposes only |
| 2 Solvent | CDCl3 |
| 3 Temperature | 298.0 |
| 4 Number of Scans | 16 |
| 5 Receiver Gain | 18.0 |
| 6 Relaxation Delay | 1.0000 |
| 7 Pulse Width | 8.0700 |
| 8 Spectrometer Frequency | 600.13 |
| 9 Nucleus | 1H |

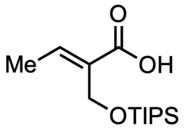


**S5**
*crude; for identification purposes only*
Chemical Formula: $C_{14}H_{28}O_3Si$
Exact Mass: 272.1808
Molecular Weight: 272.4600

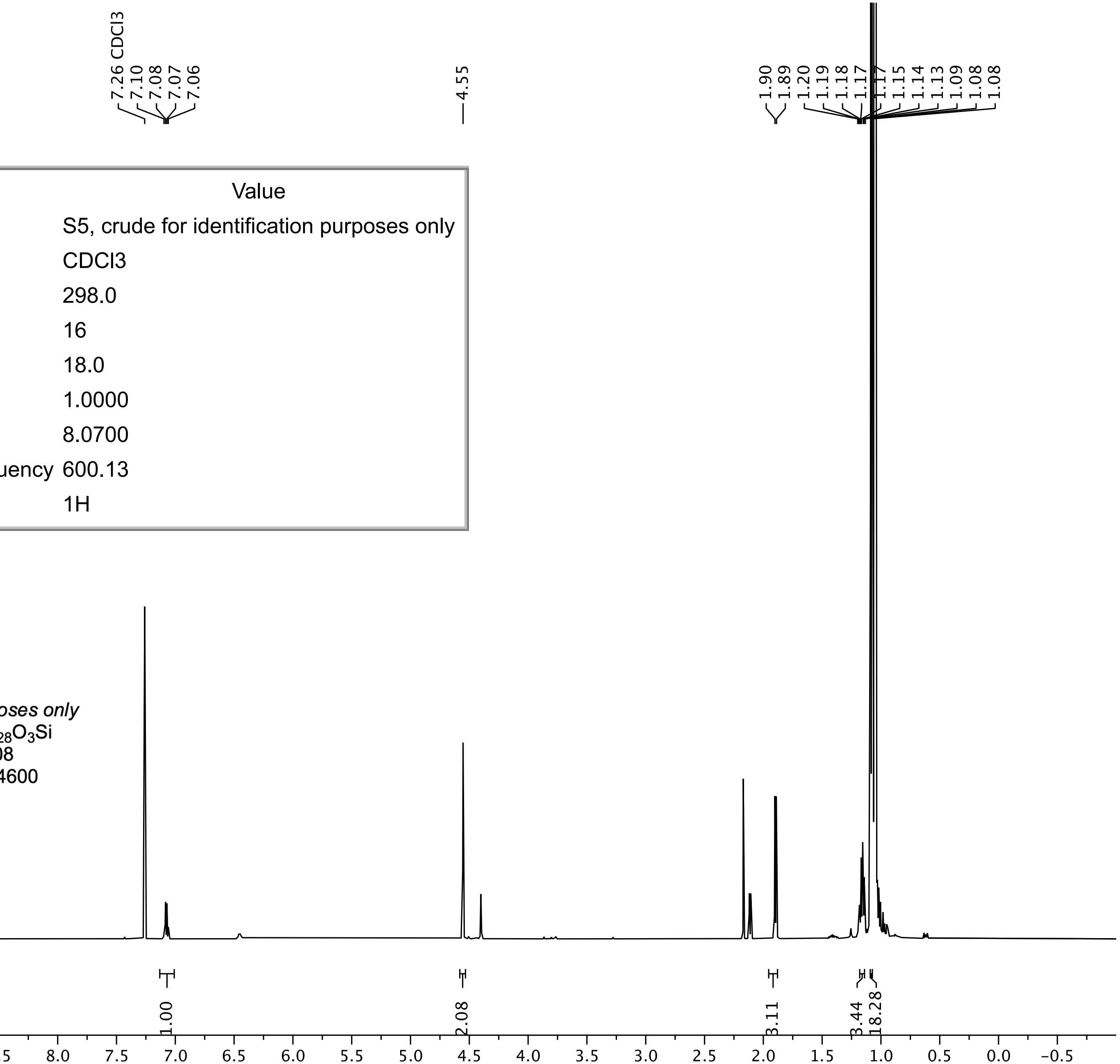

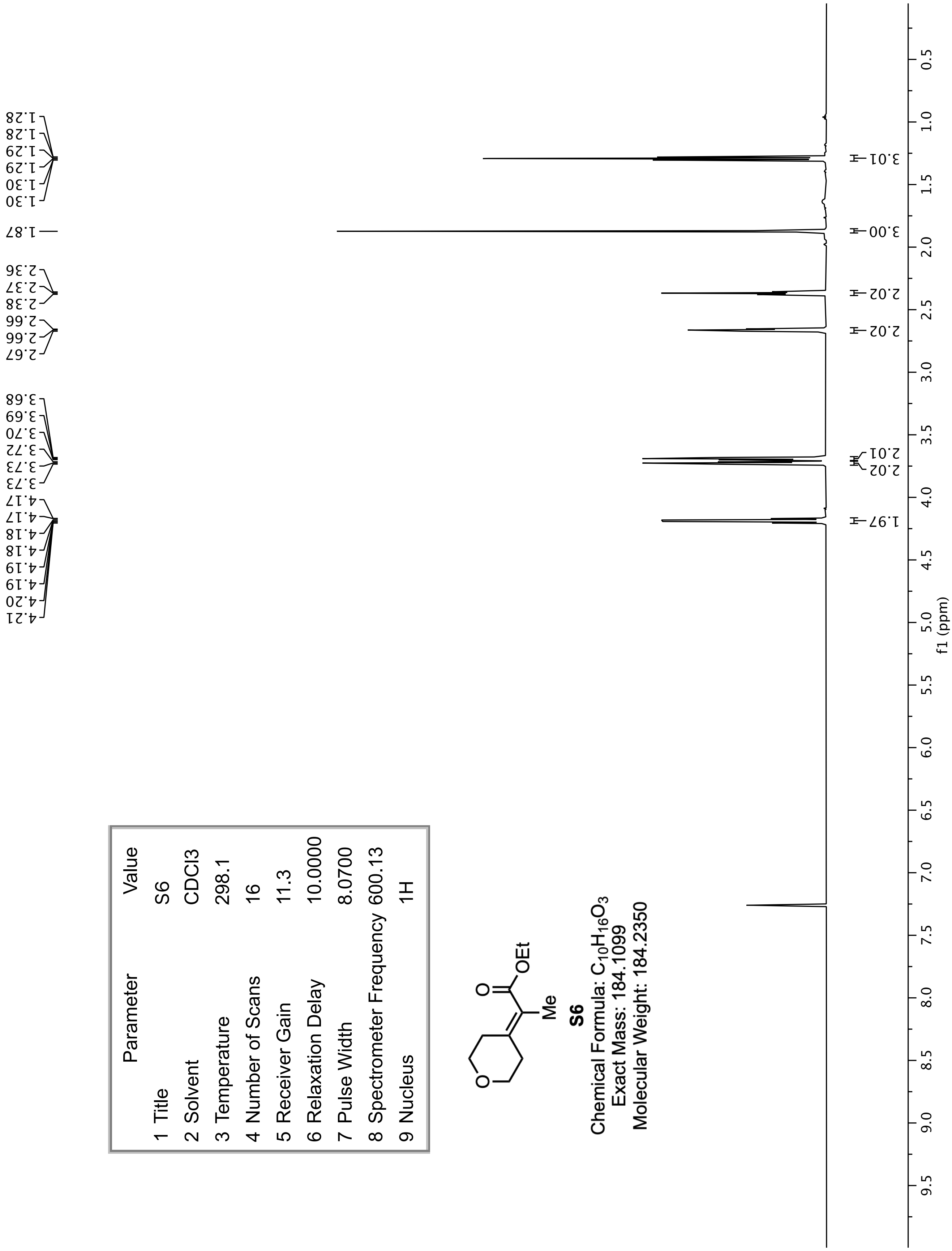


| Parameter | Value |
|---|---|
| 1 Title | S6 |
| 2 Solvent | CDCl3 |
| 3 Temperature | 298.1 |
| 4 Number of Scans | 16 |
| 5 Receiver Gain | 11.3 |
| 6 Relaxation Delay | 10.0000 |
| 7 Pulse Width | 8.0700 |
| 8 Spectrometer Frequency | 600.13 |
| 9 Nucleus | 1H |

**S6**

Chemical Formula: $C_{10}H_{16}O_3$
Exact Mass: 184.1099
Molecular Weight: 184.2350

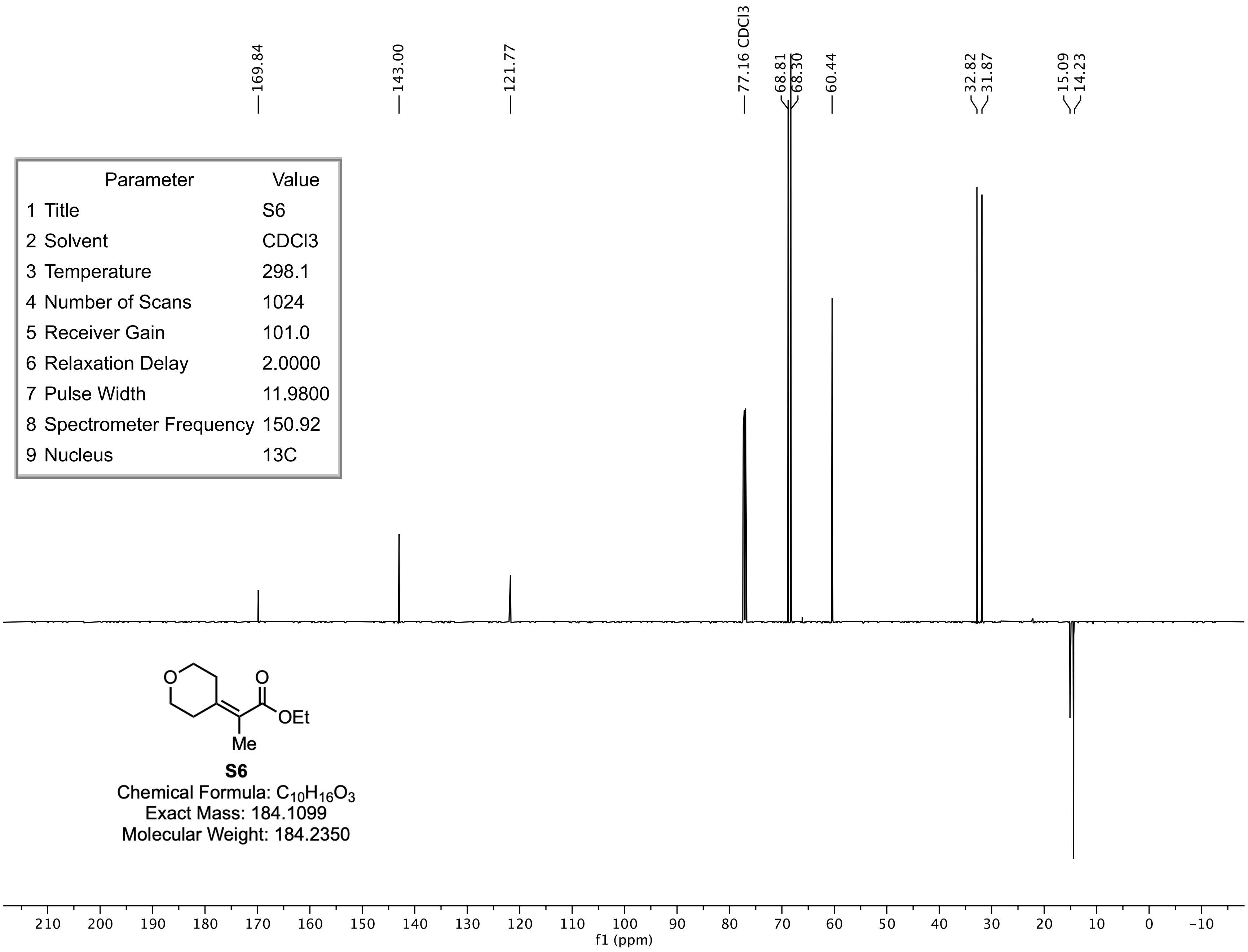


| Parameter | Value |
|---|---|
| 1 Title | S6 |
| 2 Solvent | CDCl3 |
| 3 Temperature | 298.1 |
| 4 Number of Scans | 1024 |
| 5 Receiver Gain | 101.0 |
| 6 Relaxation Delay | 2.0000 |
| 7 Pulse Width | 11.9800 |
| 8 Spectrometer Frequency | 150.92 |
| 9 Nucleus | 13C |

**S6**
Chemical Formula: $C_{10}H_{16}O_3$
Exact Mass: 184.1099
Molecular Weight: 184.2350

| | Parameter | Value |
|---|---|---|
| 1 | Title | S7 in 70% purity, for identification purposes only |
| 2 | Solvent | CDCl3 |
| 3 | Temperature | 298.0 |
| 4 | Number of Scans | 16 |
| 5 | Receiver Gain | 11.3 |
| 6 | Relaxation Delay | 10.0000 |
| 7 | Pulse Width | 8.0700 |
| 8 | Spectrometer Frequency | 600.13 |
| 9 | Nucleus | 1H |

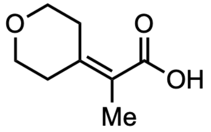


**S7** for identification purposes only
Chemical Formula: $C_8H_{12}O_3$
Exact Mass: 156.0786
Molecular Weight: 156.1810

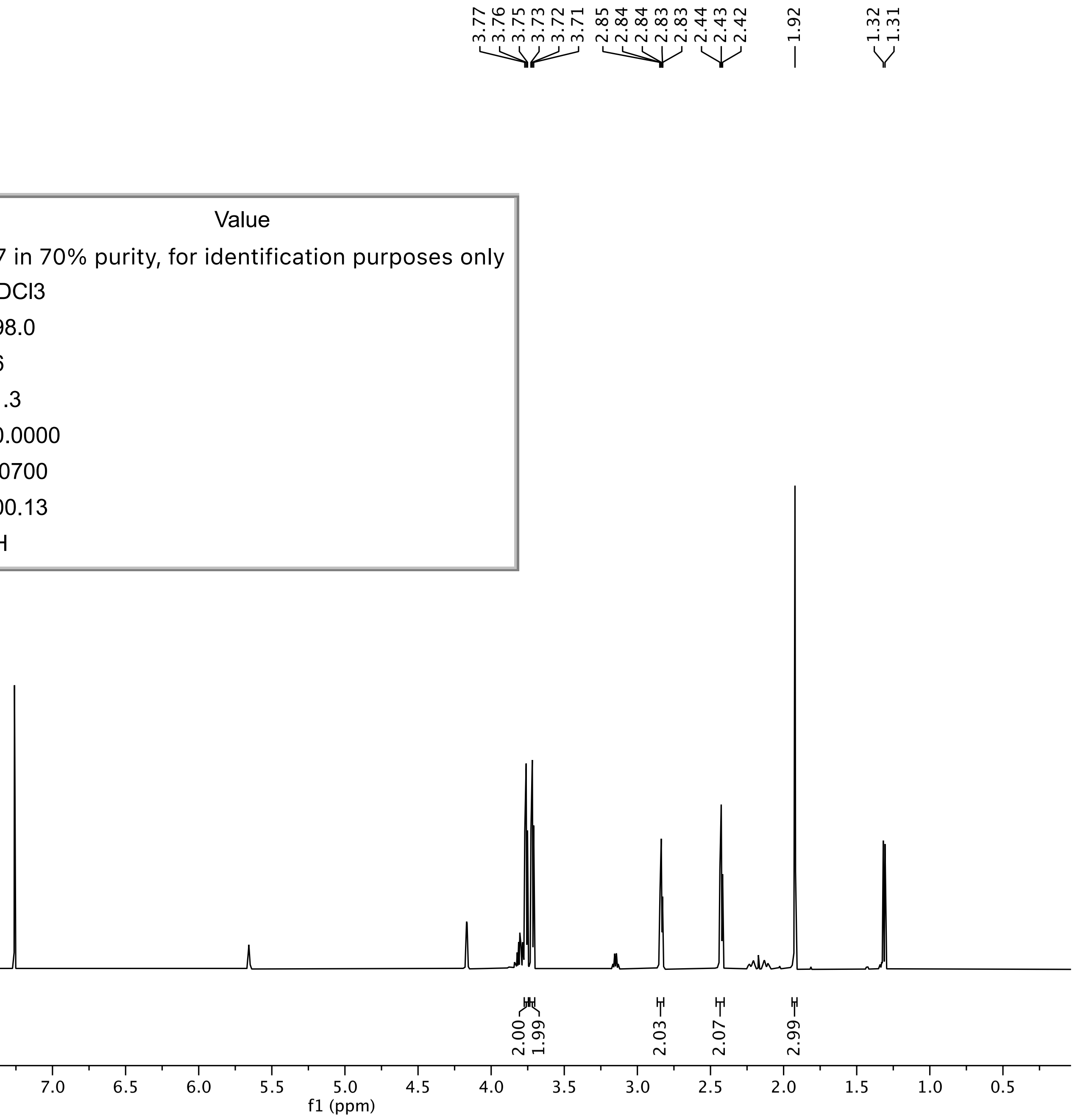

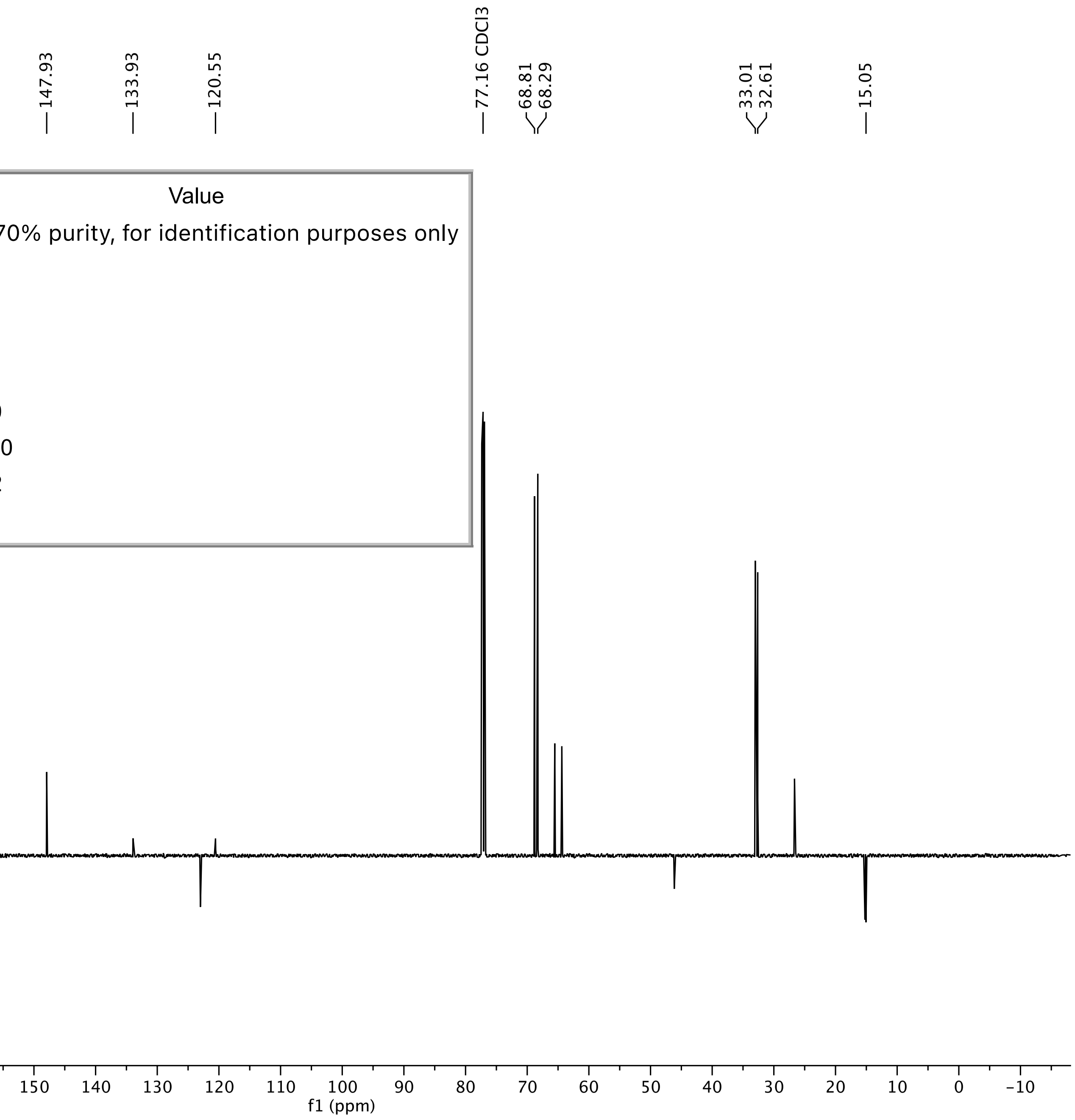


| Parameter | Value |
|---|---|
| 1 Title | S7 in 70% purity, for identification purposes only |
| 2 Solvent | CDCl3 |
| 3 Temperature | 298.0 |
| 4 Number of Scans | 1024 |
| 5 Receiver Gain | 101.0 |
| 6 Relaxation Delay | 2.0000 |
| 7 Pulse Width | 11.9800 |
| 8 Spectrometer Frequency | 150.92 |
| 9 Nucleus | 13C |

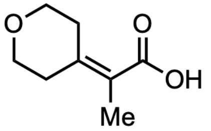


**S7** for identification purposes only
Chemical Formula: $C_8H_{12}O_3$
Exact Mass: 156.0786
Molecular Weight: 156.1810

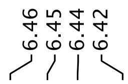


| | Parameter | Value |
|---|---|---|
| 1 | Title | 3 |
| 2 | Solvent | CDCl3 |
| 3 | Temperature | 300.0 |
| 4 | Number of Scans | 16 |
| 5 | Receiver Gain | 57.0 |
| 6 | Relaxation Delay | 1.0000 |
| 7 | Pulse Width | 11.1500 |
| 8 | Spectrometer Frequency | 600.32 |
| 9 | Nucleus | 1H |

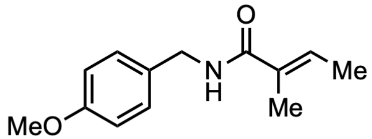


**3**
Chemical Formula: $C_{13}H_{17}NO_2$
Exact Mass: 219.1259
Molecular Weight: 219.2840

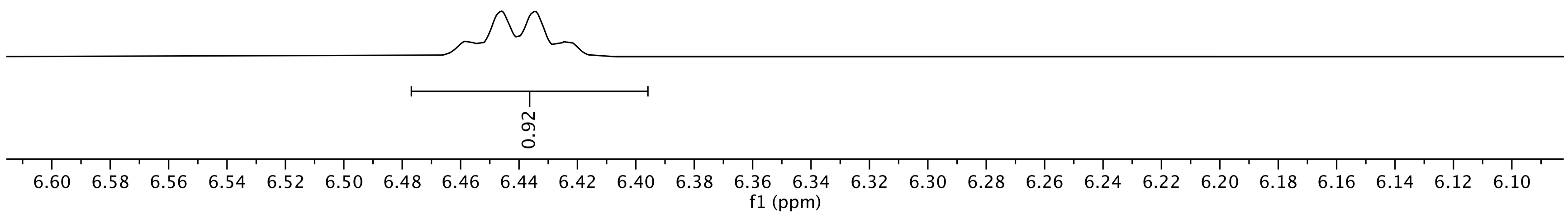

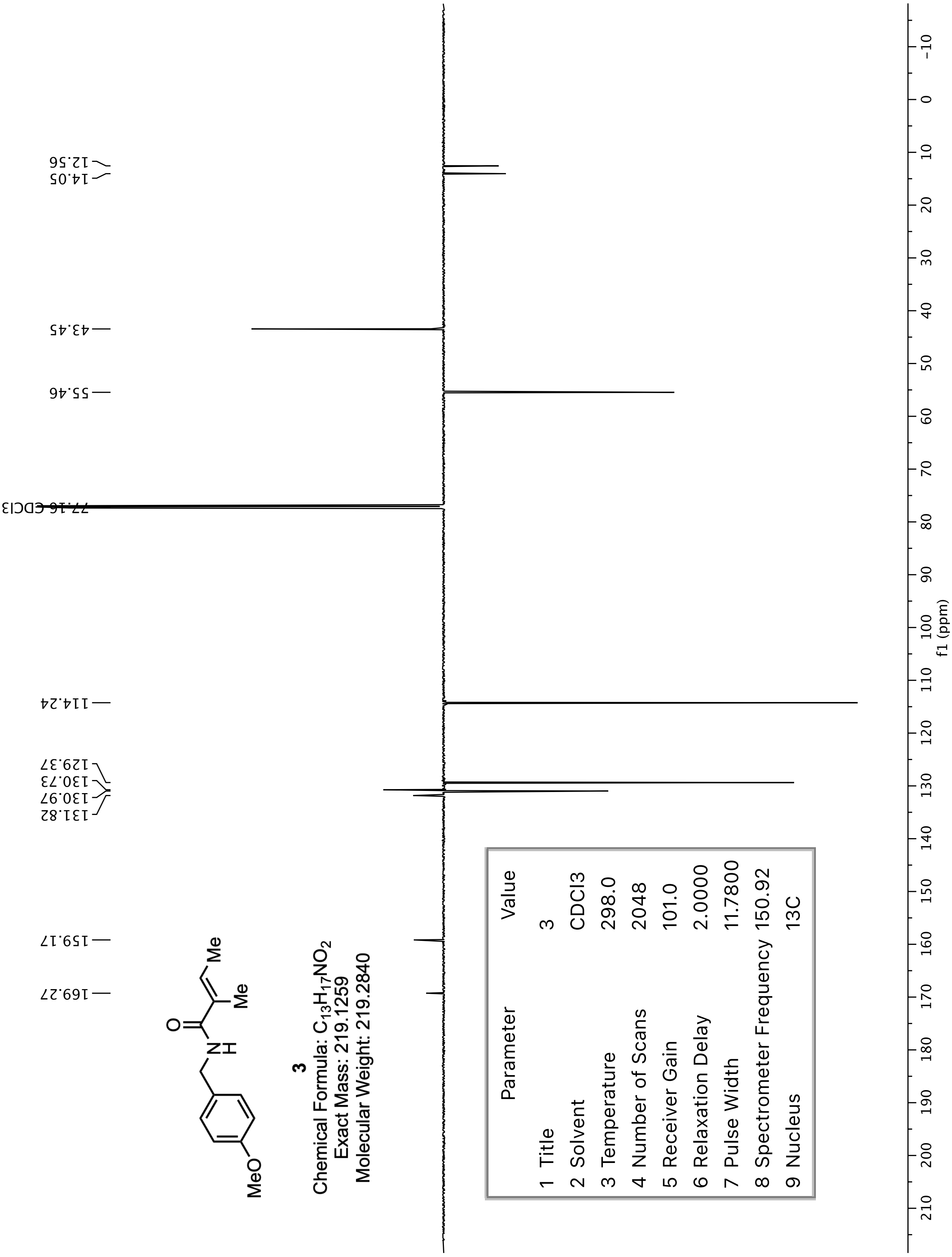


**3**
Chemical Formula: $C_{13}H_{17}NO_2$
Exact Mass: 219.1259
Molecular Weight: 219.2840

| | Parameter | Value |
|---|---|---|
| 1 | Title | 3 |
| 2 | Solvent | CDCl3 |
| 3 | Temperature | 298.0 |
| 4 | Number of Scans | 2048 |
| 5 | Receiver Gain | 101.0 |
| 6 | Relaxation Delay | 2.0000 |
| 7 | Pulse Width | 11.7800 |
| 8 | Spectrometer Frequency | 150.92 |
| 9 | Nucleus | 13C |

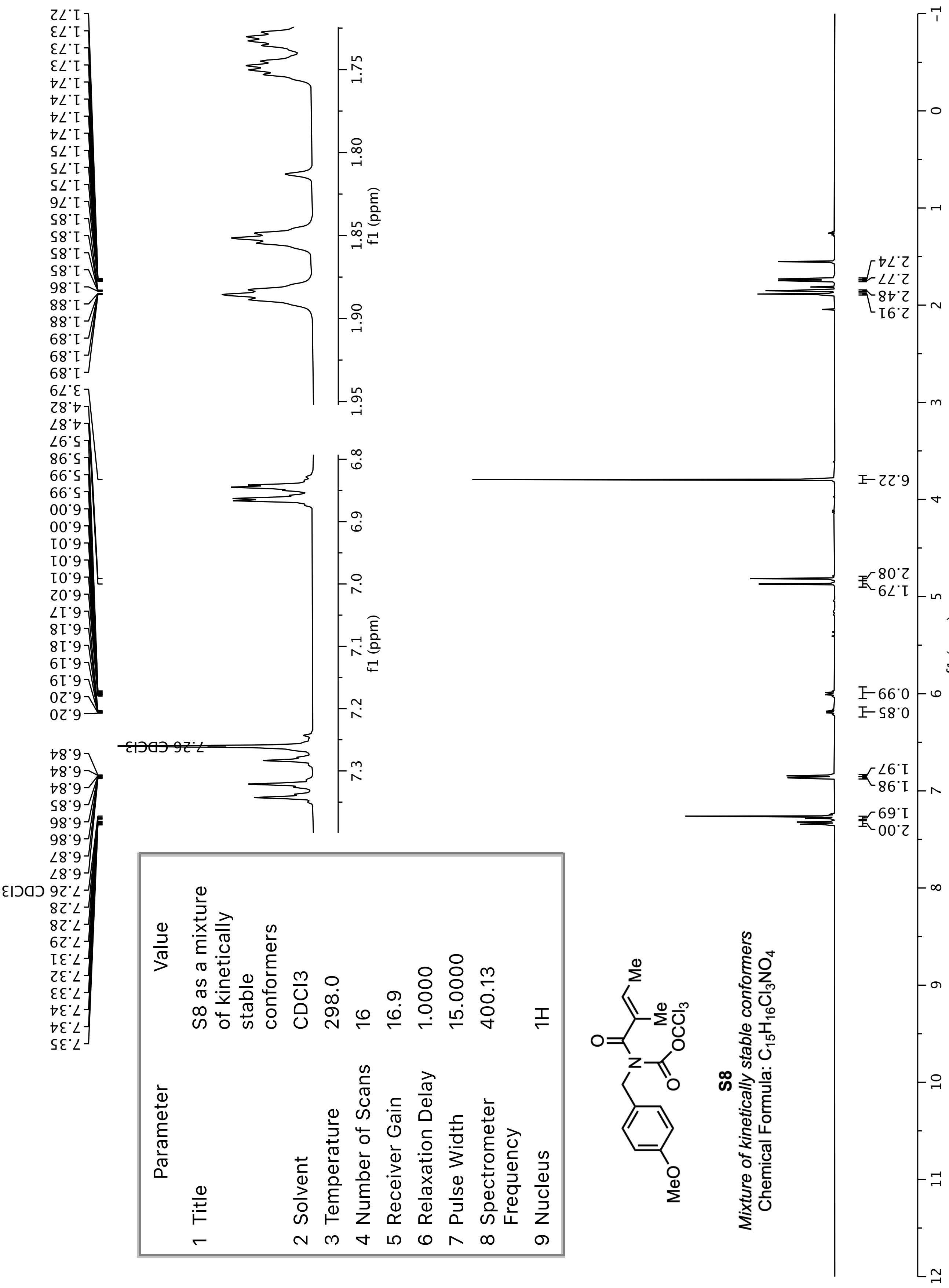


| | Parameter | Value |
|---|---|---|
| 1 | Title | S8 as a mixture of kinetically stable conformers |
| 2 | Solvent | CDCl3 |
| 3 | Temperature | 298.0 |
| 4 | Number of Scans | 16 |
| 5 | Receiver Gain | 16.9 |
| 6 | Relaxation Delay | 1.0000 |
| 7 | Pulse Width | 15.0000 |
| 8 | Spectrometer Frequency | 400.13 |
| 9 | Nucleus | 1H |

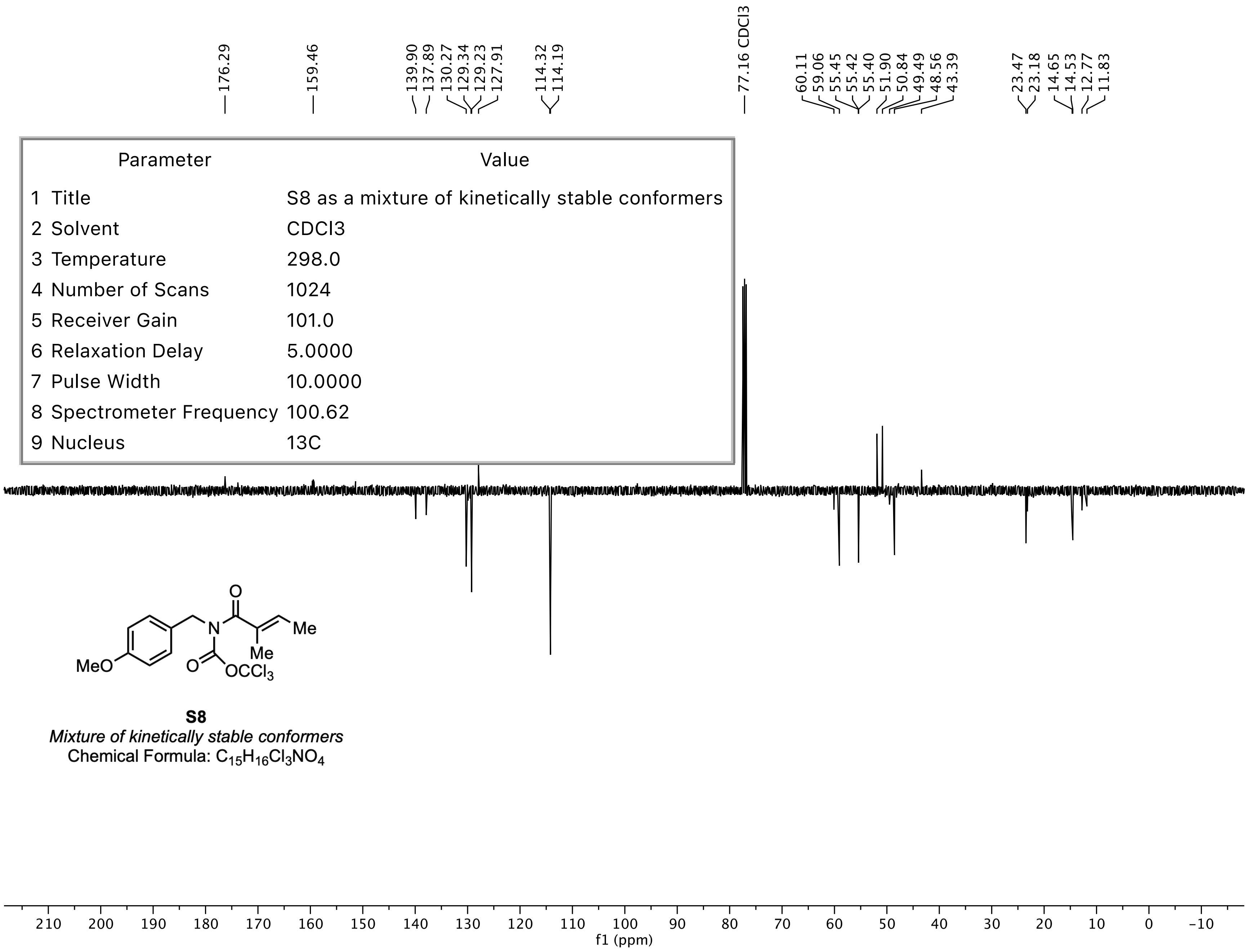


| Parameter | Value |
|---|---|
| 1 Title | S8 as a mixture of kinetically stable conformers |
| 2 Solvent | CDCl3 |
| 3 Temperature | 298.0 |
| 4 Number of Scans | 1024 |
| 5 Receiver Gain | 101.0 |
| 6 Relaxation Delay | 5.0000 |
| 7 Pulse Width | 10.0000 |
| 8 Spectrometer Frequency | 100.62 |
| 9 Nucleus | 13C |



**S8**

*Mixture of kinetically stable conformers*

Chemical Formula: $C_{15}H_{16}Cl_3NO_4$

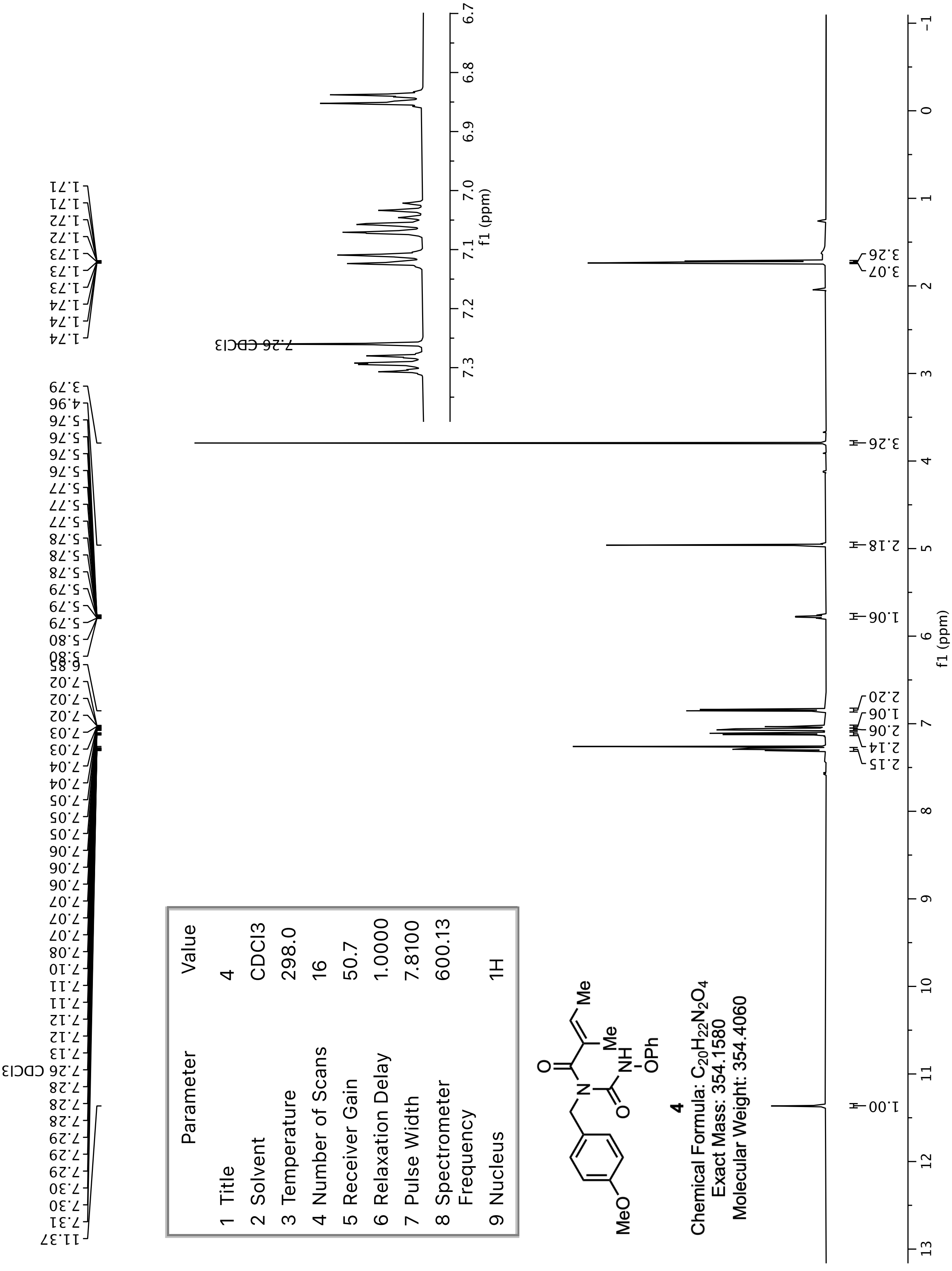


| Parameter | Value |
|---|---|
| 1 Title | 4 |
| 2 Solvent | CDCl3 |
| 3 Temperature | 298.0 |
| 4 Number of Scans | 16 |
| 5 Receiver Gain | 50.7 |
| 6 Relaxation Delay | 1.0000 |
| 7 Pulse Width | 7.8100 |
| 8 Spectrometer Frequency | 600.13 |
| 9 Nucleus | 1H |

**4**

Chemical Formula: $C_{20}H_{22}N_2O_4$
Exact Mass: 354.1580
Molecular Weight: 354.4060

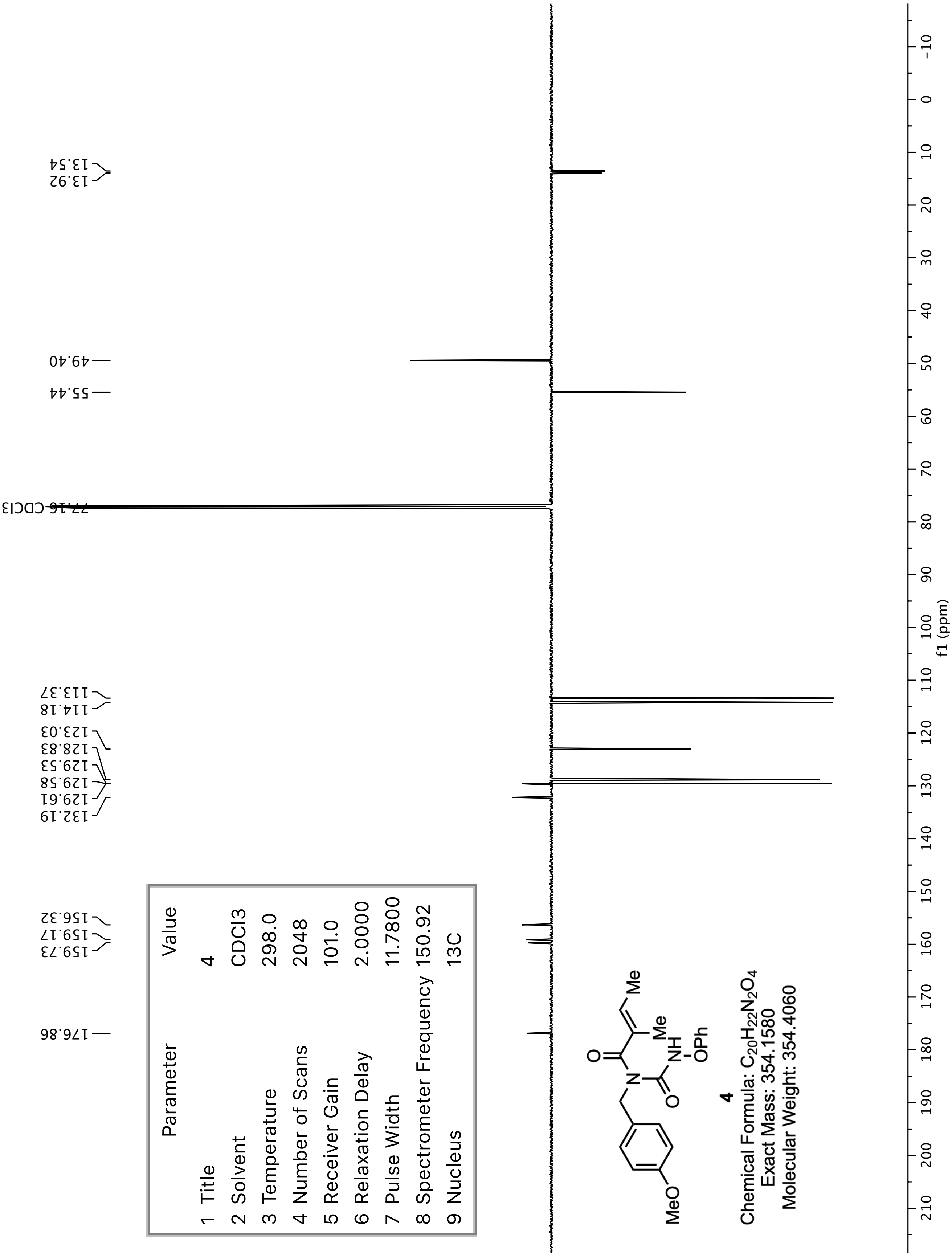


| | Parameter | Value |
|---|---|---|
| 1 | Title | 4 |
| 2 | Solvent | CDCl3 |
| 3 | Temperature | 298.0 |
| 4 | Number of Scans | 2048 |
| 5 | Receiver Gain | 101.0 |
| 6 | Relaxation Delay | 2.0000 |
| 7 | Pulse Width | 11.7800 |
| 8 | Spectrometer Frequency | 150.92 |
| 9 | Nucleus | 13C |

**4**

Chemical Formula: $C_{20}H_{22}N_2O_4$
Exact Mass: 354.1580
Molecular Weight: 354.4060

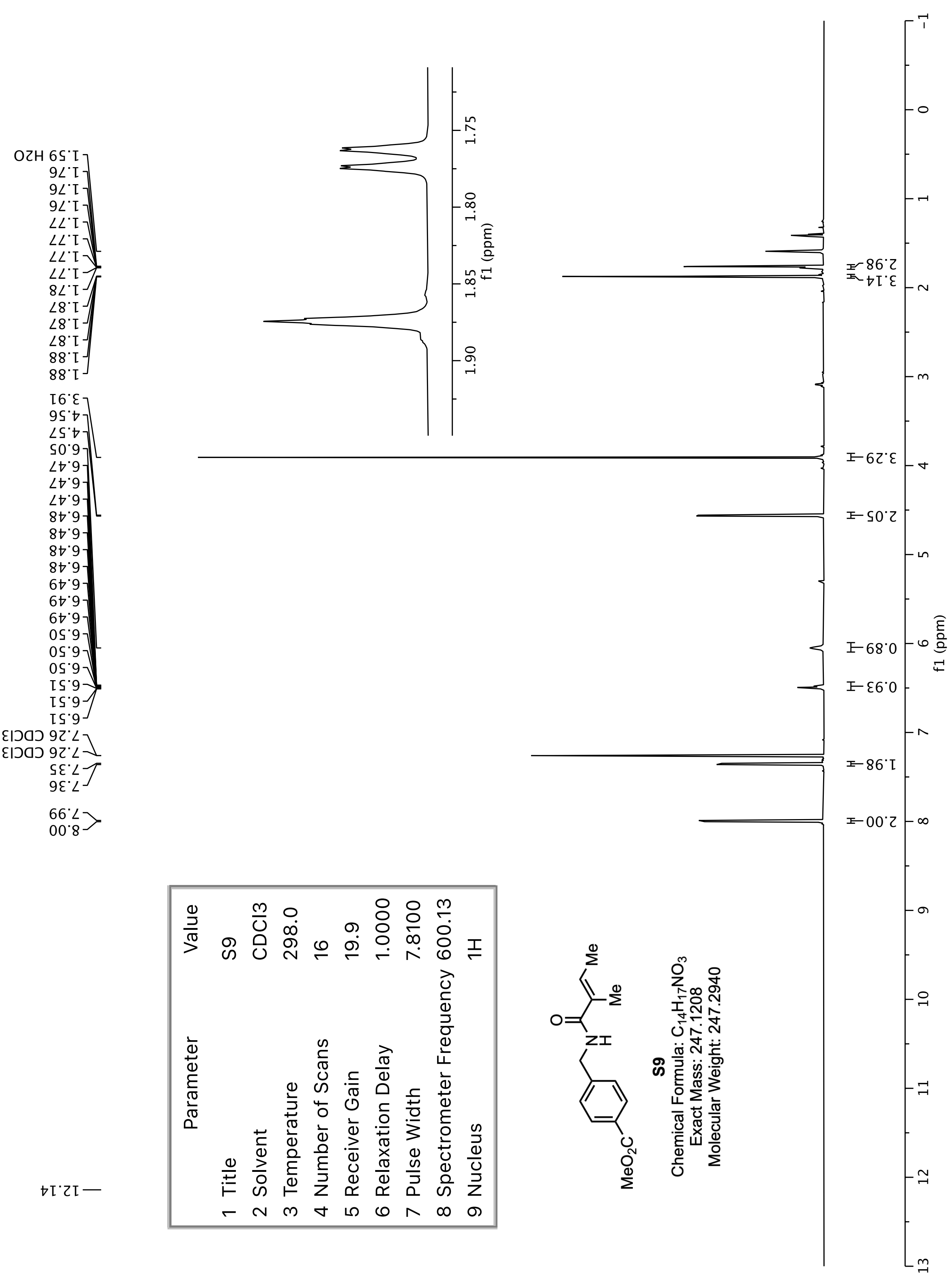


| Parameter | Value |
|---|---|
| 1 Title | S9 |
| 2 Solvent | CDCl3 |
| 3 Temperature | 298.0 |
| 4 Number of Scans | 16 |
| 5 Receiver Gain | 19.9 |
| 6 Relaxation Delay | 1.0000 |
| 7 Pulse Width | 7.8100 |
| 8 Spectrometer Frequency | 600.13 |
| 9 Nucleus | 1H |

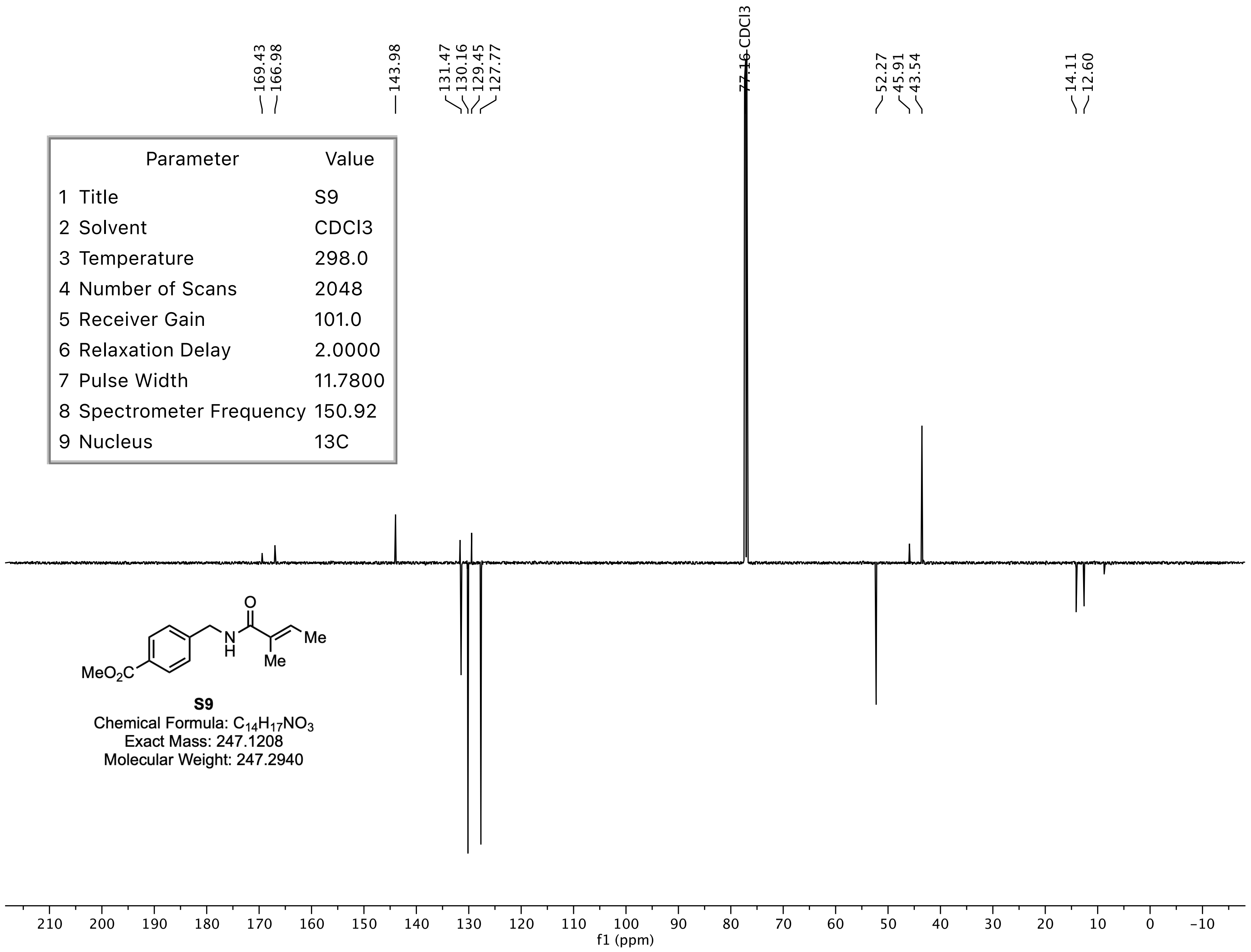


| | Parameter | Value |
|---|---|---|
| 1 | Title | S9 |
| 2 | Solvent | CDCl3 |
| 3 | Temperature | 298.0 |
| 4 | Number of Scans | 2048 |
| 5 | Receiver Gain | 101.0 |
| 6 | Relaxation Delay | 2.0000 |
| 7 | Pulse Width | 11.7800 |
| 8 | Spectrometer Frequency | 150.92 |
| 9 | Nucleus | 13C |

**S9**
Chemical Formula: $C_{14}H_{17}NO_3$
Exact Mass: 247.1208
Molecular Weight: 247.2940

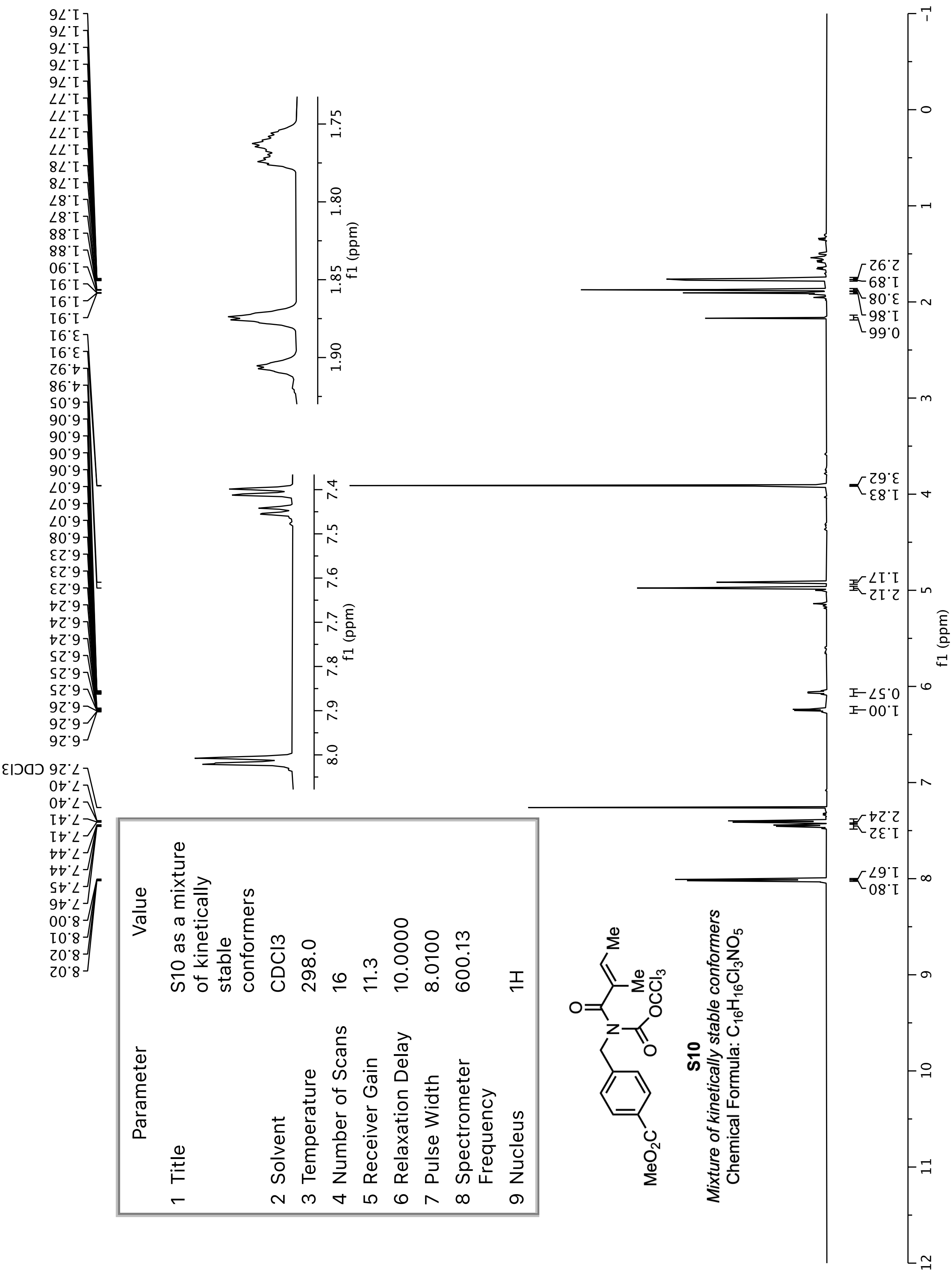


| Parameter | Value |
| --- | --- |
| 1 Title | S10 as a mixture of kinetically stable conformers |
| 2 Solvent | CDCl3 |
| 3 Temperature | 298.0 |
| 4 Number of Scans | 16 |
| 5 Receiver Gain | 11.3 |
| 6 Relaxation Delay | 10.0000 |
| 7 Pulse Width | 8.0100 |
| 8 Spectrometer Frequency | 600.13 |
| 9 Nucleus | 1H |

**S10**

*Mixture of kinetically stable conformers*

Chemical Formula: $C_{16}H_{16}Cl_3NO_5$

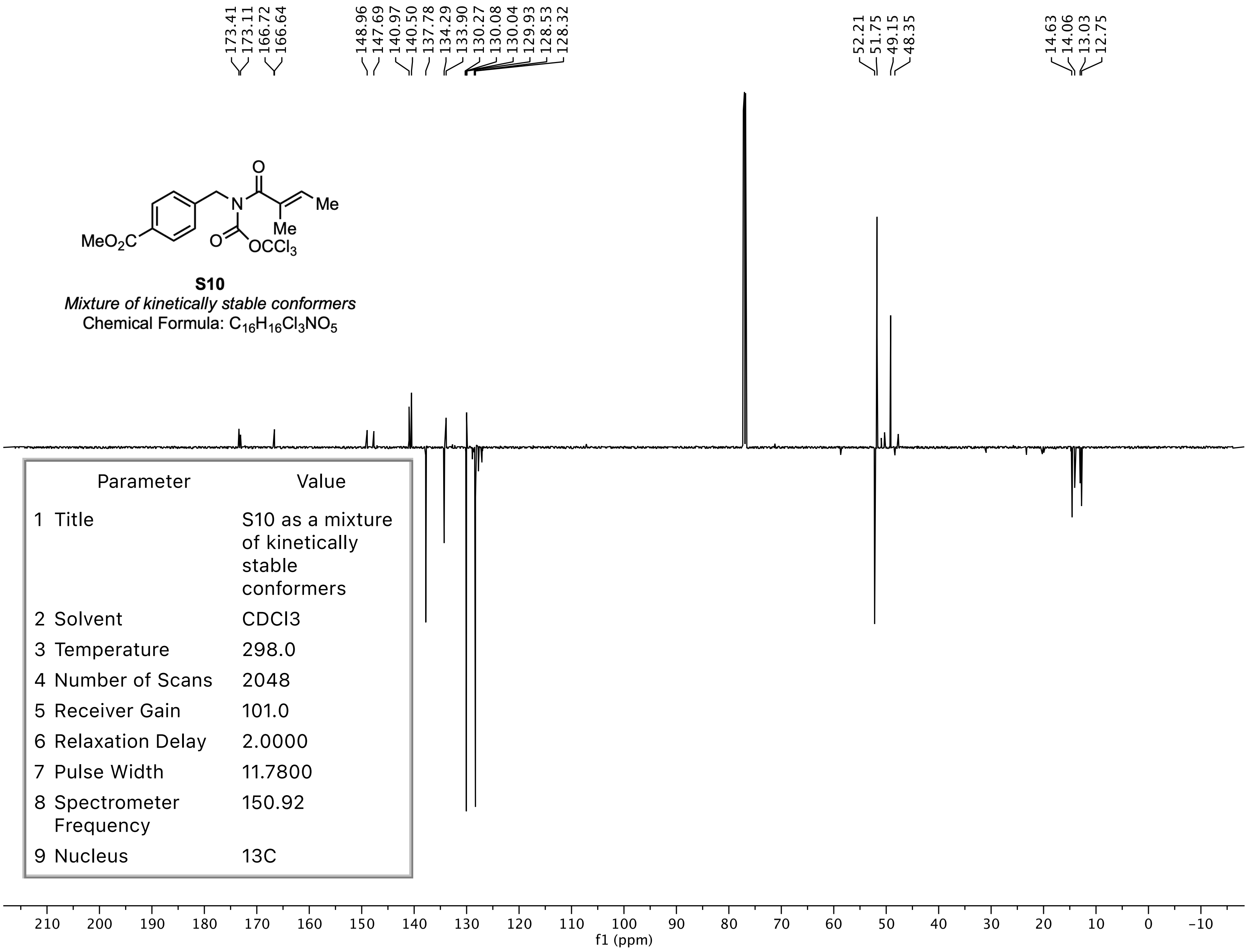


**S10**
*Mixture of kinetically stable conformers*
Chemical Formula: $C_{16}H_{16}Cl_3NO_5$



| | Parameter | Value |
|---|---|---|
| 1 | Title | S10 as a mixture of kinetically stable conformers |
| 2 | Solvent | CDCl3 |
| 3 | Temperature | 298.0 |
| 4 | Number of Scans | 2048 |
| 5 | Receiver Gain | 101.0 |
| 6 | Relaxation Delay | 2.0000 |
| 7 | Pulse Width | 11.7800 |
| 8 | Spectrometer Frequency | 150.92 |
| 9 | Nucleus | 13C |

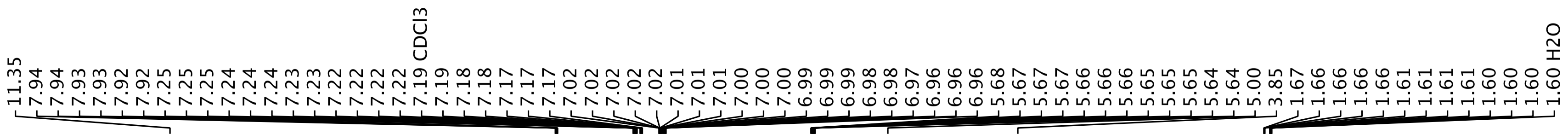


| Parameter | | Value |
|---|---|---|
| 1 | Title | S11 |
| 2 | Solvent | CDCl3 |
| 3 | Temperature | 298.0 |
| 4 | Number of Scans | 16 |
| 5 | Receiver Gain | 11.3 |
| 6 | Relaxation Delay | 10.0000 |
| 7 | Pulse Width | 8.0700 |
| 8 | Spectrometer Frequency | 600.13 |
| 9 | Nucleus | 1H |

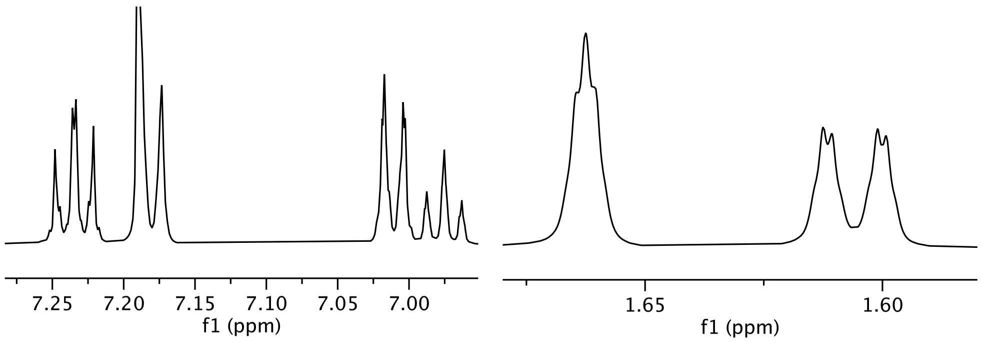


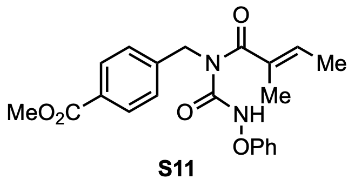


**S11**
Chemical Formula: $C_{21}H_{22}N_2O_5$
Exact Mass: 382.1529
Molecular Weight: 382.4160

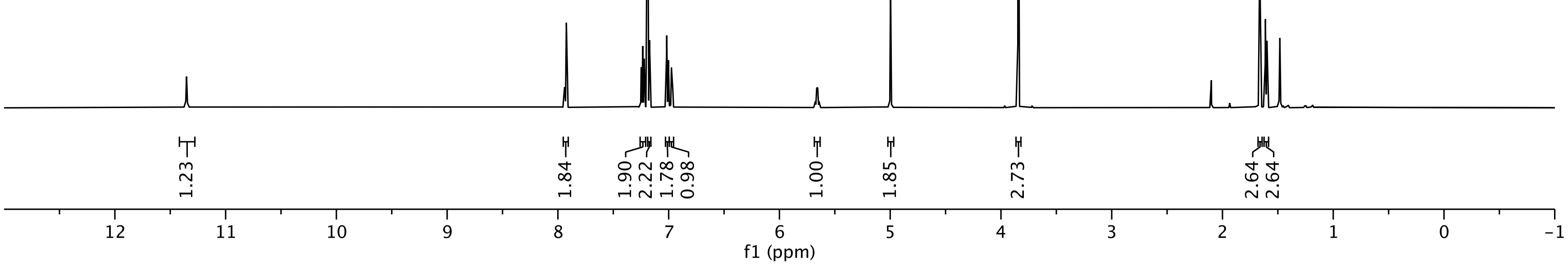

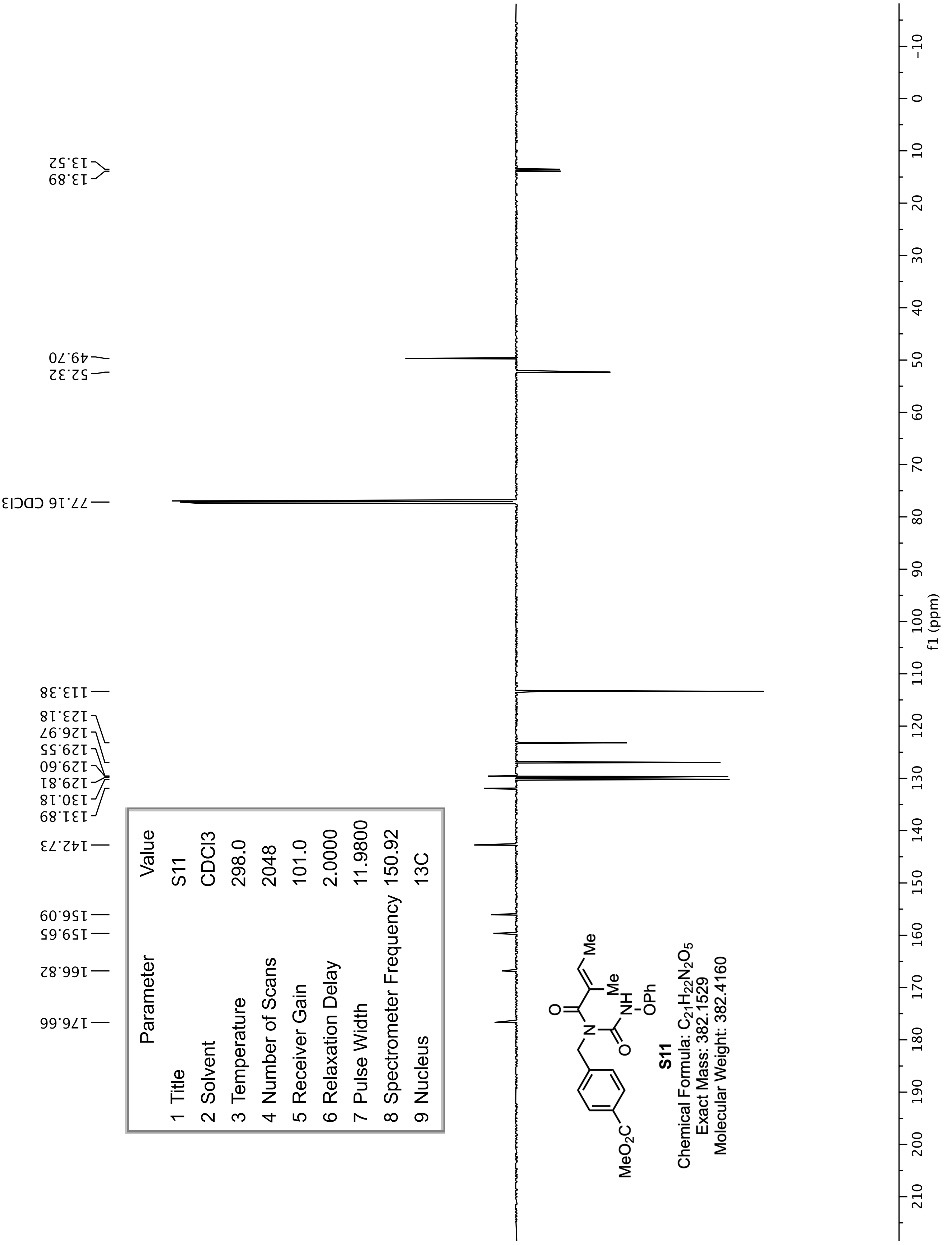


| | Parameter | Value |
|---|---|---|
| 1 | Title | S11 |
| 2 | Solvent | CDCl3 |
| 3 | Temperature | 298.0 |
| 4 | Number of Scans | 2048 |
| 5 | Receiver Gain | 101.0 |
| 6 | Relaxation Delay | 2.0000 |
| 7 | Pulse Width | 11.9800 |
| 8 | Spectrometer Frequency | 150.92 |
| 9 | Nucleus | 13C |

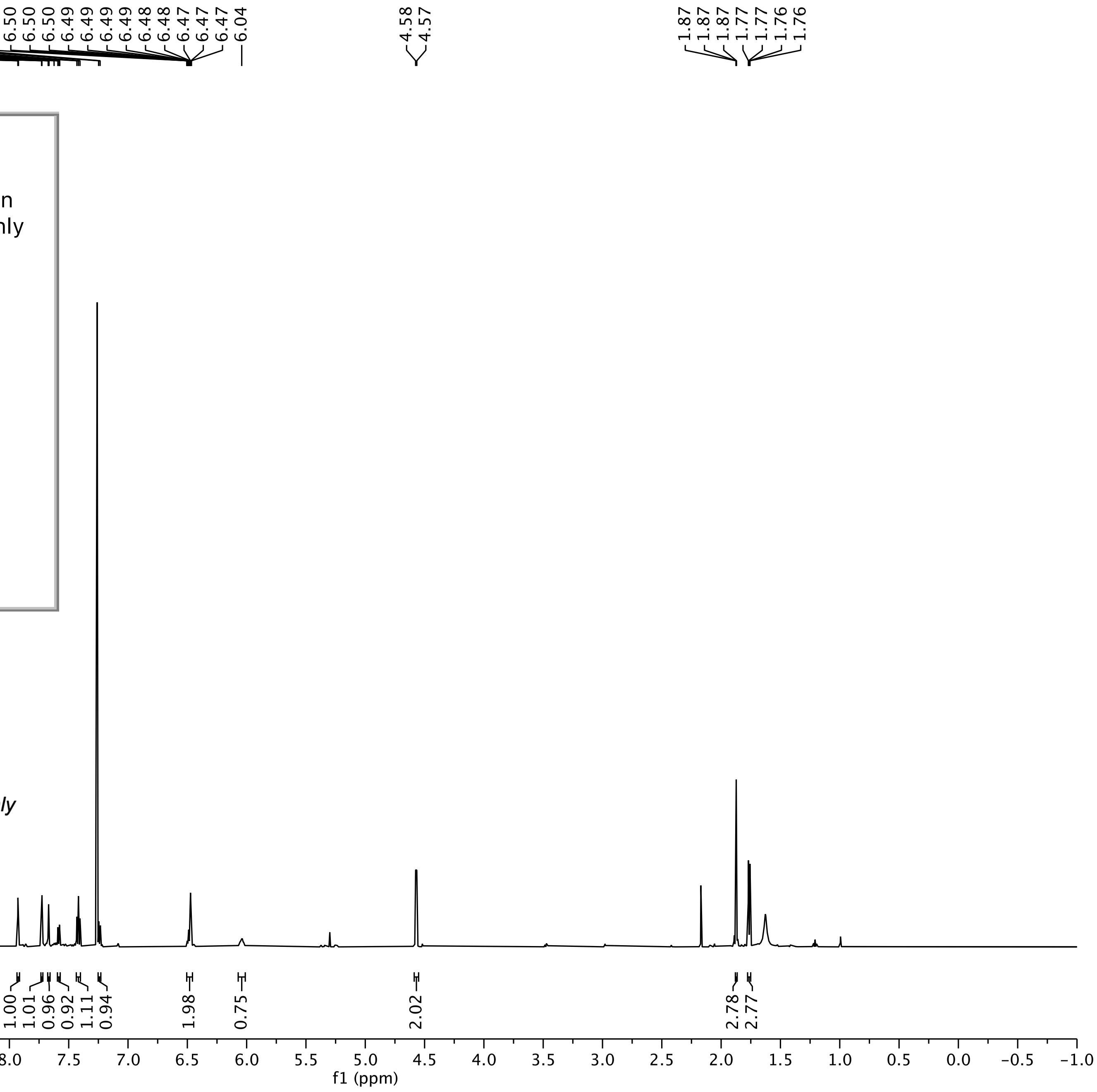


| Parameter | Value |
|---|---|
| 1 Title | S12 for identification purposes only |
| 2 Solvent | CDCl3 |
| 3 Temperature | 298.0 |
| 4 Number of Scans | 16 |
| 5 Receiver Gain | 16.0 |
| 6 Relaxation Delay | 10.0000 |
| 7 Pulse Width | 8.0000 |
| 8 Spectrometer Frequency | 600.13 |
| 9 Nucleus | 1H |

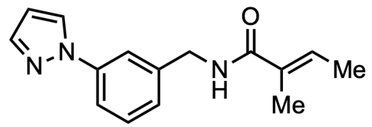


**S12**

*Crude; for identification purposes only*

Chemical Formula: $C_{15}H_{17}N_3O$

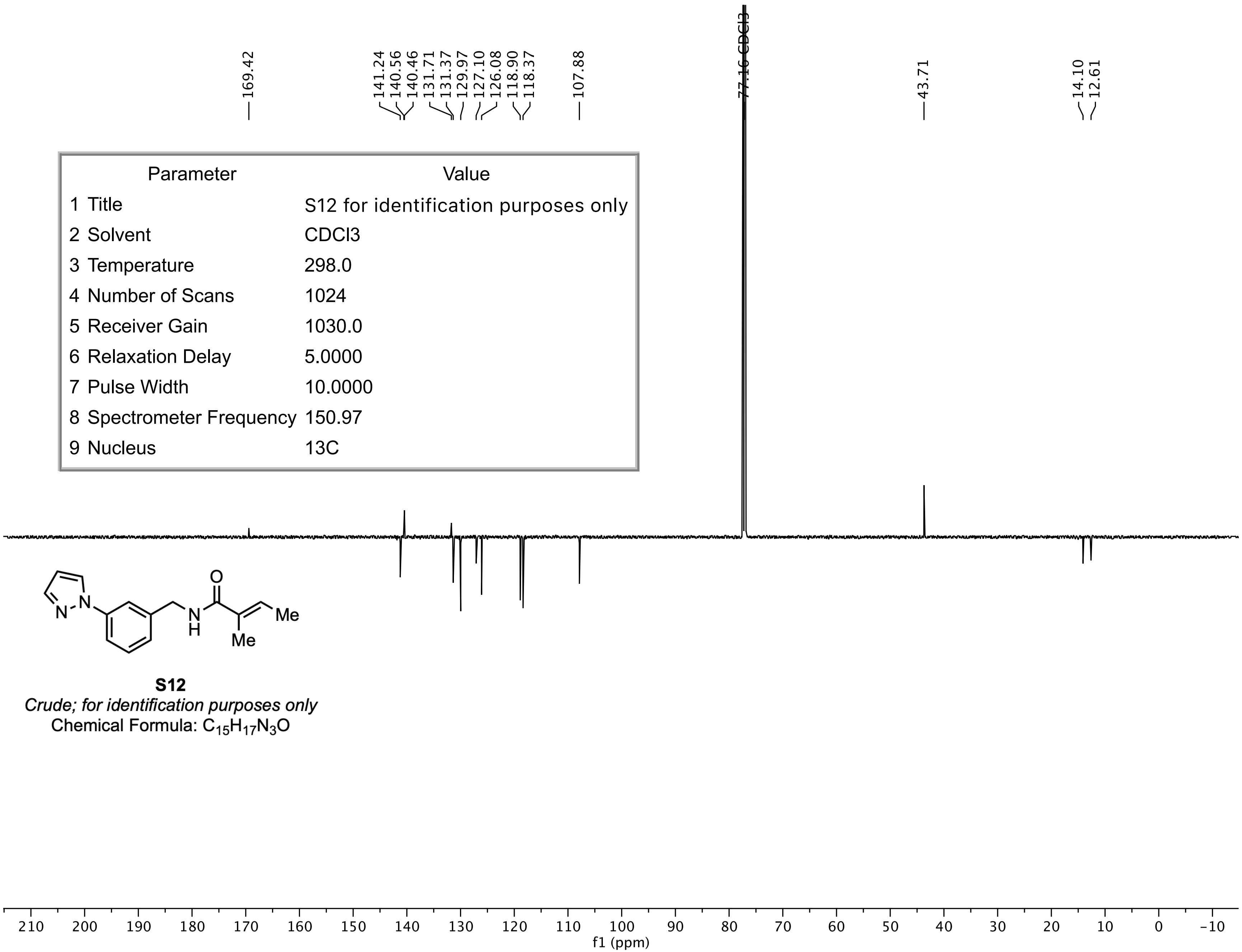


| Parameter | Value |
|---|---|
| 1 Title | S12 for identification purposes only |
| 2 Solvent | CDCl3 |
| 3 Temperature | 298.0 |
| 4 Number of Scans | 1024 |
| 5 Receiver Gain | 1030.0 |
| 6 Relaxation Delay | 5.0000 |
| 7 Pulse Width | 10.0000 |
| 8 Spectrometer Frequency | 150.97 |
| 9 Nucleus | 13C |

**S12**

*Crude; for identification purposes only*

Chemical Formula: $C_{15}H_{17}N_3O$

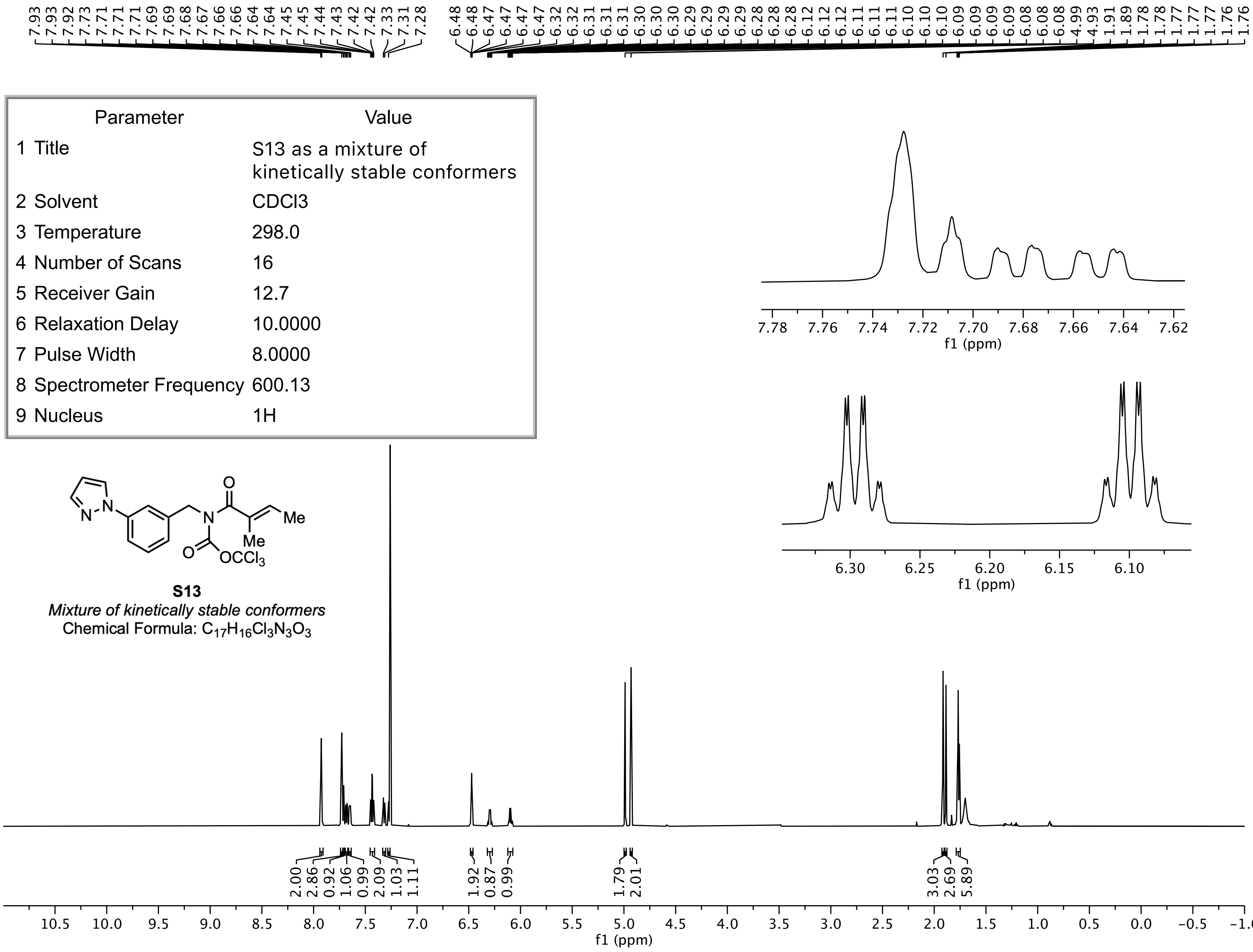


| Parameter | Value |
|---|---|
| 1 Title | S13 as a mixture of kinetically stable conformers |
| 2 Solvent | CDCl3 |
| 3 Temperature | 298.0 |
| 4 Number of Scans | 16 |
| 5 Receiver Gain | 12.7 |
| 6 Relaxation Delay | 10.0000 |
| 7 Pulse Width | 8.0000 |
| 8 Spectrometer Frequency | 600.13 |
| 9 Nucleus | 1H |

**S13**

*Mixture of kinetically stable conformers*

Chemical Formula: $C_{17}H_{16}Cl_3N_3O_3$

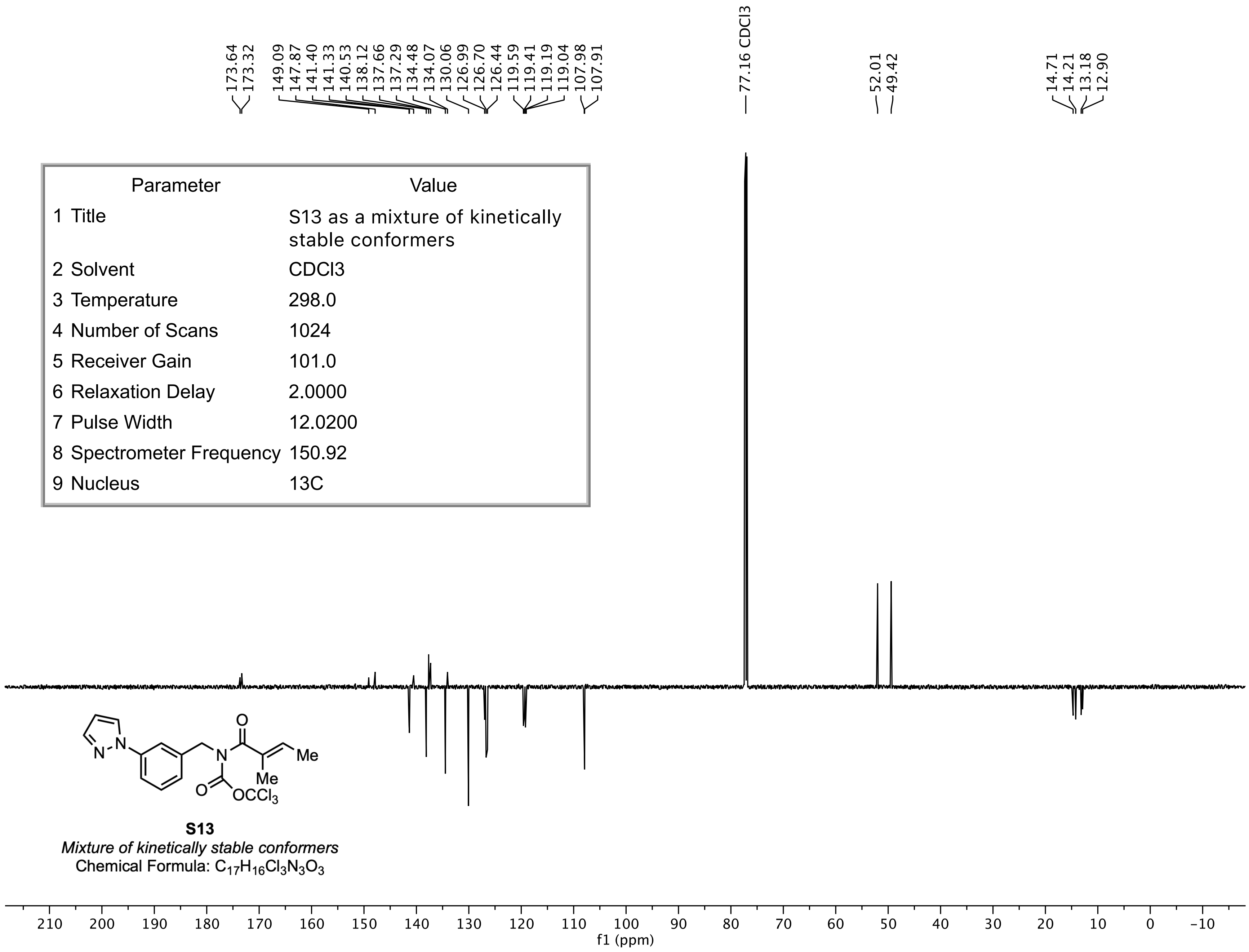


| Parameter | Value |
|---|---|
| 1 Title | S13 as a mixture of kinetically stable conformers |
| 2 Solvent | CDCl3 |
| 3 Temperature | 298.0 |
| 4 Number of Scans | 1024 |
| 5 Receiver Gain | 101.0 |
| 6 Relaxation Delay | 2.0000 |
| 7 Pulse Width | 12.0200 |
| 8 Spectrometer Frequency | 150.92 |
| 9 Nucleus | 13C |

**S13**

*Mixture of kinetically stable conformers*

Chemical Formula: $C_{17}H_{16}Cl_3N_3O_3$

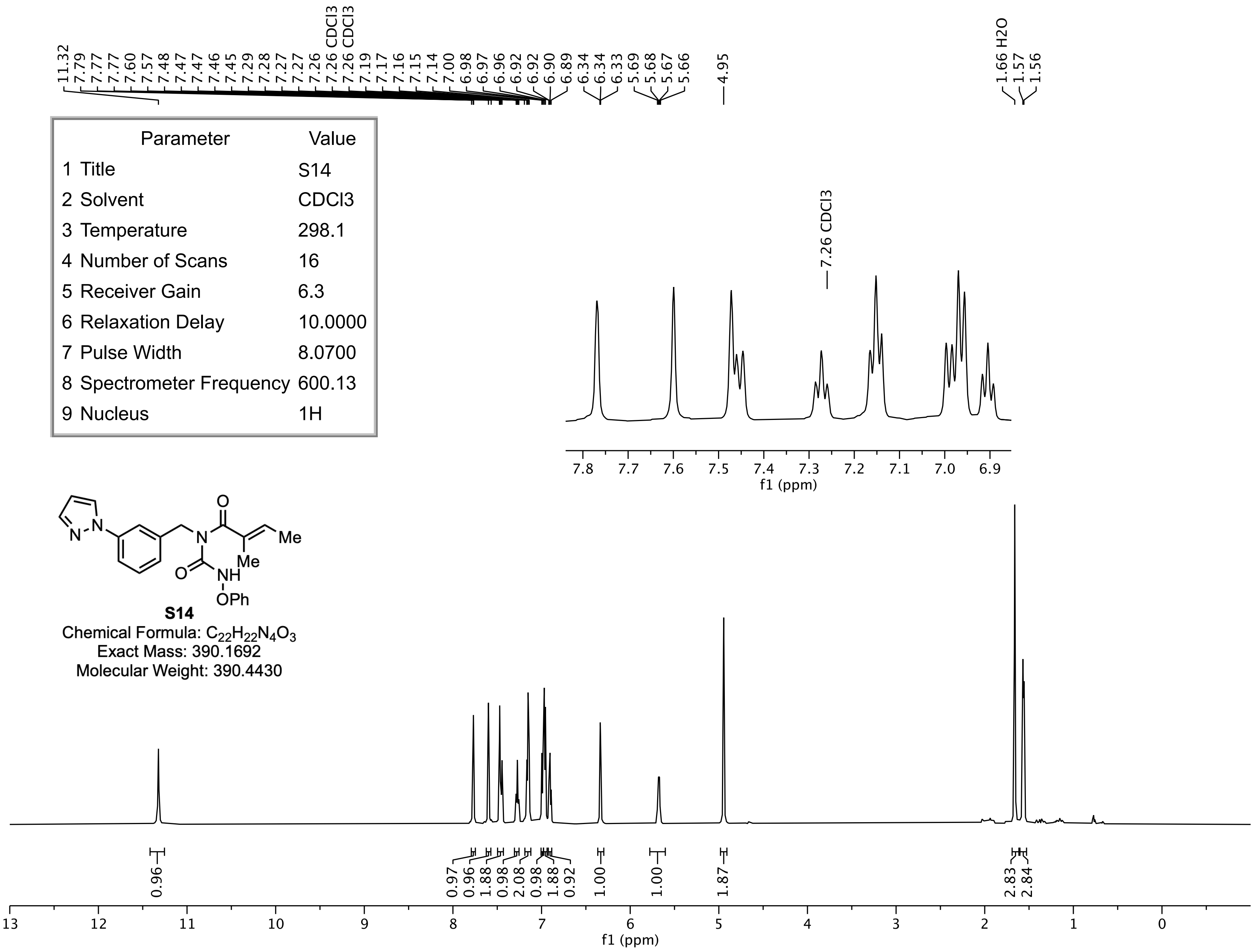


| Parameter | Value |
|---|---|
| 1 Title | S14 |
| 2 Solvent | CDCl3 |
| 3 Temperature | 298.1 |
| 4 Number of Scans | 16 |
| 5 Receiver Gain | 6.3 |
| 6 Relaxation Delay | 10.0000 |
| 7 Pulse Width | 8.0700 |
| 8 Spectrometer Frequency | 600.13 |
| 9 Nucleus | 1H |

**S14**

Chemical Formula: $C_{22}H_{22}N_4O_3$

Exact Mass: 390.1692

Molecular Weight: 390.4430

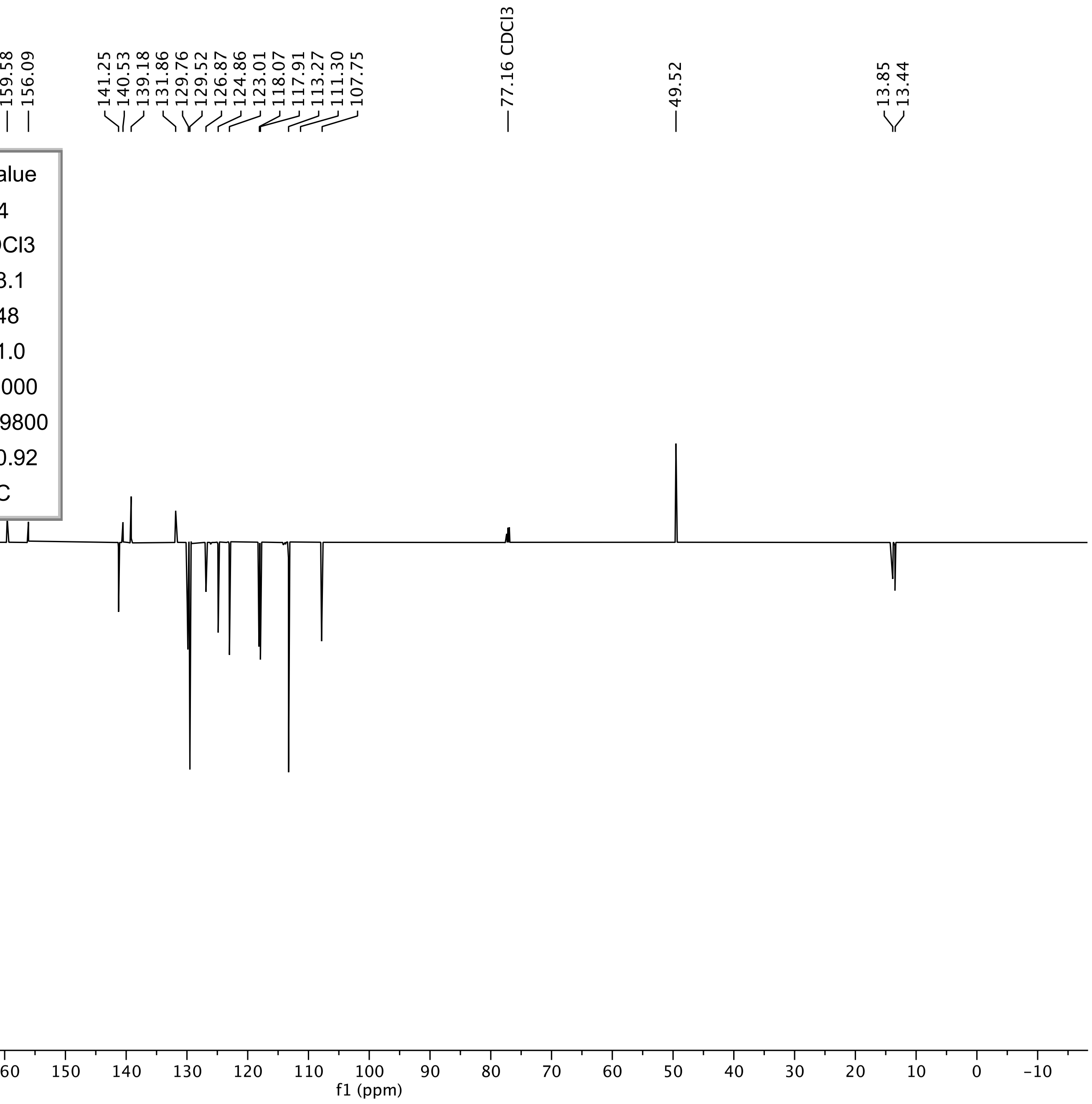


| Parameter | Value |
|---|---|
| 1 Title | S14 |
| 2 Solvent | CDCl3 |
| 3 Temperature | 298.1 |
| 4 Number of Scans | 2048 |
| 5 Receiver Gain | 101.0 |
| 6 Relaxation Delay | 2.0000 |
| 7 Pulse Width | 11.9800 |
| 8 Spectrometer Frequency | 150.92 |
| 9 Nucleus | 13C |

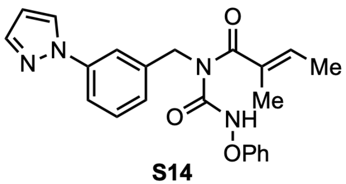


**S14**
Chemical Formula: $C_{22}H_{22}N_4O_3$
Exact Mass: 390.1692
Molecular Weight: 390.4430

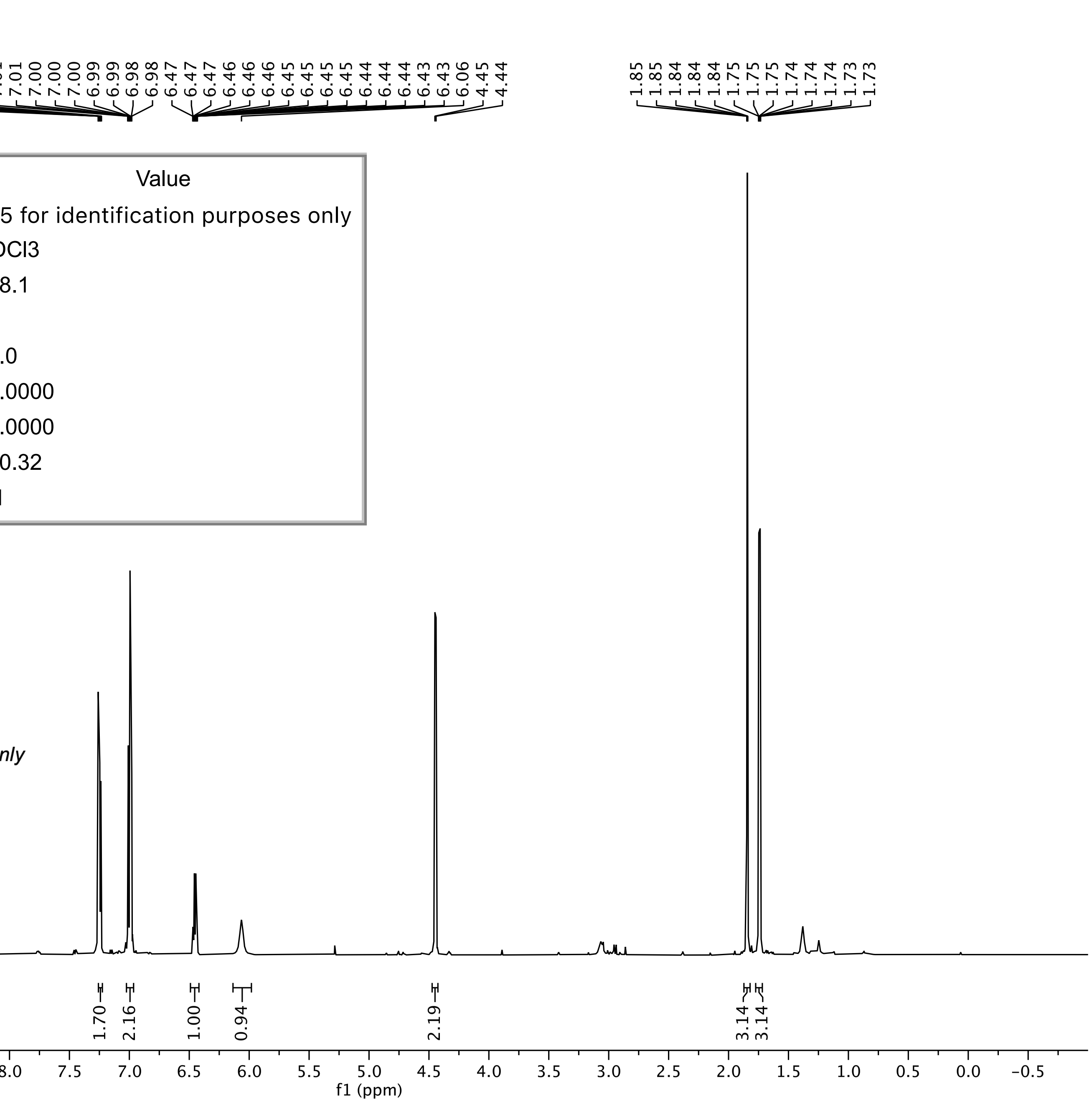


| Parameter | Value |
|---|---|
| 1 Title | S15 for identification purposes only |
| 2 Solvent | CDCl3 |
| 3 Temperature | 298.1 |
| 4 Number of Scans | 64 |
| 5 Receiver Gain | 32.0 |
| 6 Relaxation Delay | 10.0000 |
| 7 Pulse Width | 12.0000 |
| 8 Spectrometer Frequency | 600.32 |
| 9 Nucleus | 1H |

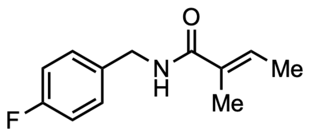


**S15**

*Crude; for identification purposes only*

Chemical Formula: $C_{12}H_{14}FNO$

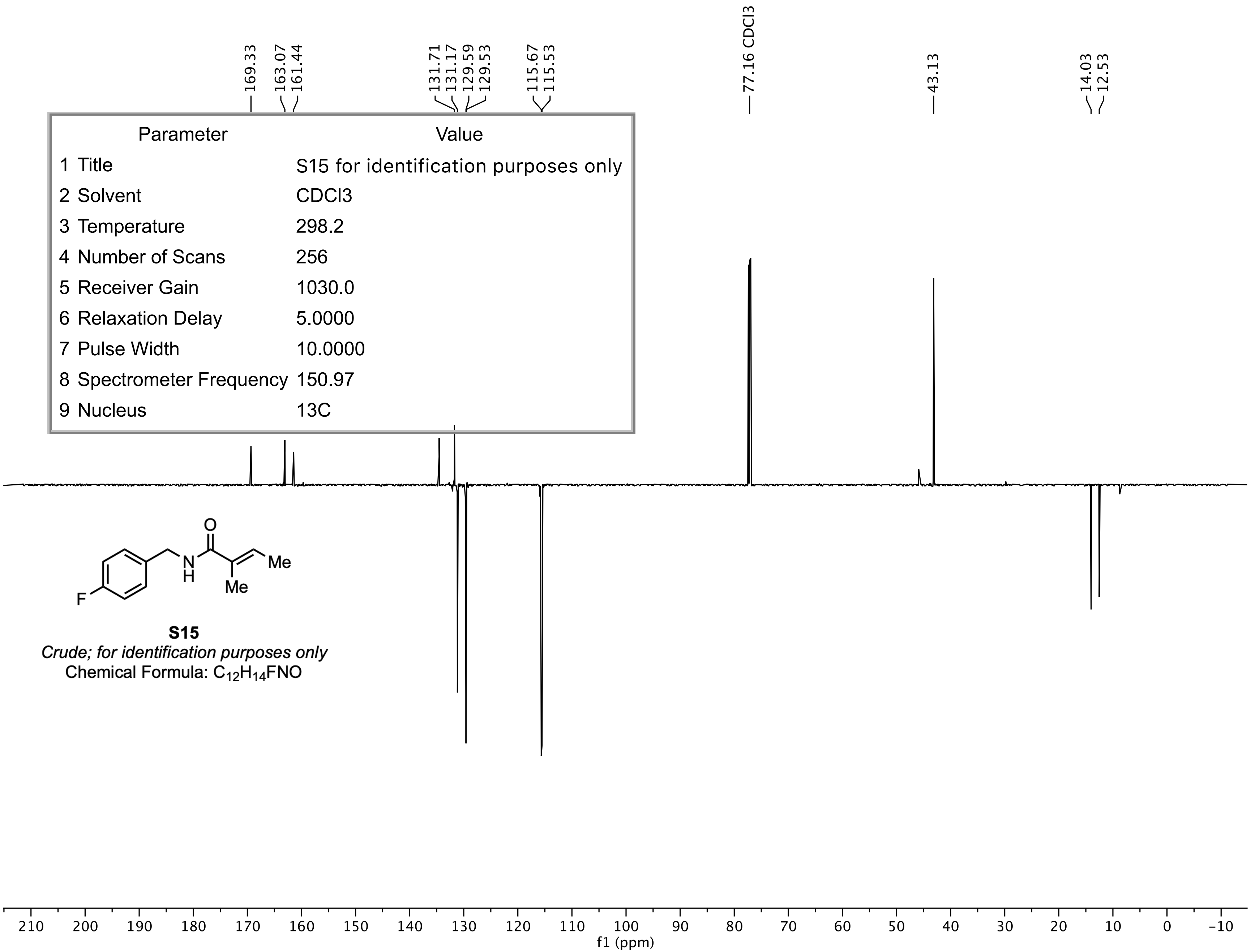


| | Parameter | Value |
|---|---|---|
| 1 | Title | S15 for identification purposes only |
| 2 | Solvent | CDCl3 |
| 3 | Temperature | 298.2 |
| 4 | Number of Scans | 256 |
| 5 | Receiver Gain | 1030.0 |
| 6 | Relaxation Delay | 5.0000 |
| 7 | Pulse Width | 10.0000 |
| 8 | Spectrometer Frequency | 150.97 |
| 9 | Nucleus | 13C |

**S15**

*Crude; for identification purposes only*

Chemical Formula: $C_{12}H_{14}FNO$

| Parameter | Value |
|---|---|
| 1 Title | S16 as a mixture of kinetically stable conformers |
| 2 Solvent | CDCl3 |
| 3 Temperature | 298.0 |
| 4 Number of Scans | 16 |
| 5 Receiver Gain | 32.0 |
| 6 Relaxation Delay | 10.0000 |
| 7 Pulse Width | 12.0000 |
| 8 Spectrometer Frequency | 600.32 |
| 9 Nucleus | 1H |

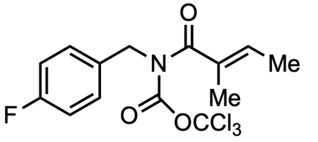


**S16**
*Mixture of kinetically stable conformers*
Chemical Formula: $C_{14}H_{13}Cl_3FNO_3$

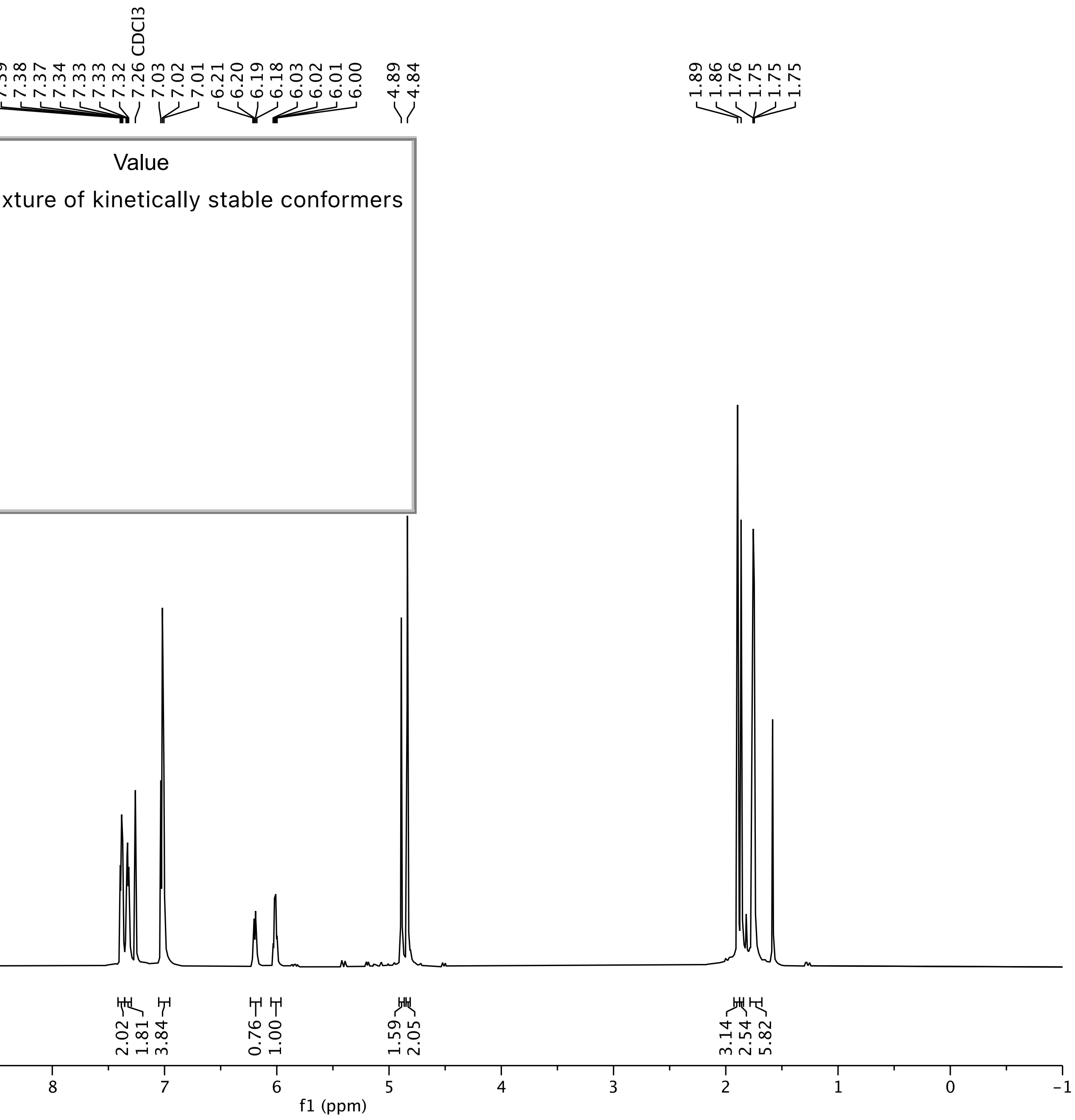

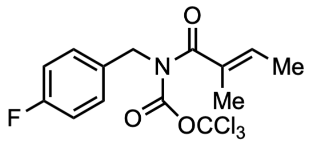

**S16**

*Mixture of kinetically stable conformers*

Chemical Formula: $C_{14}H_{13}Cl_3FNO_3$

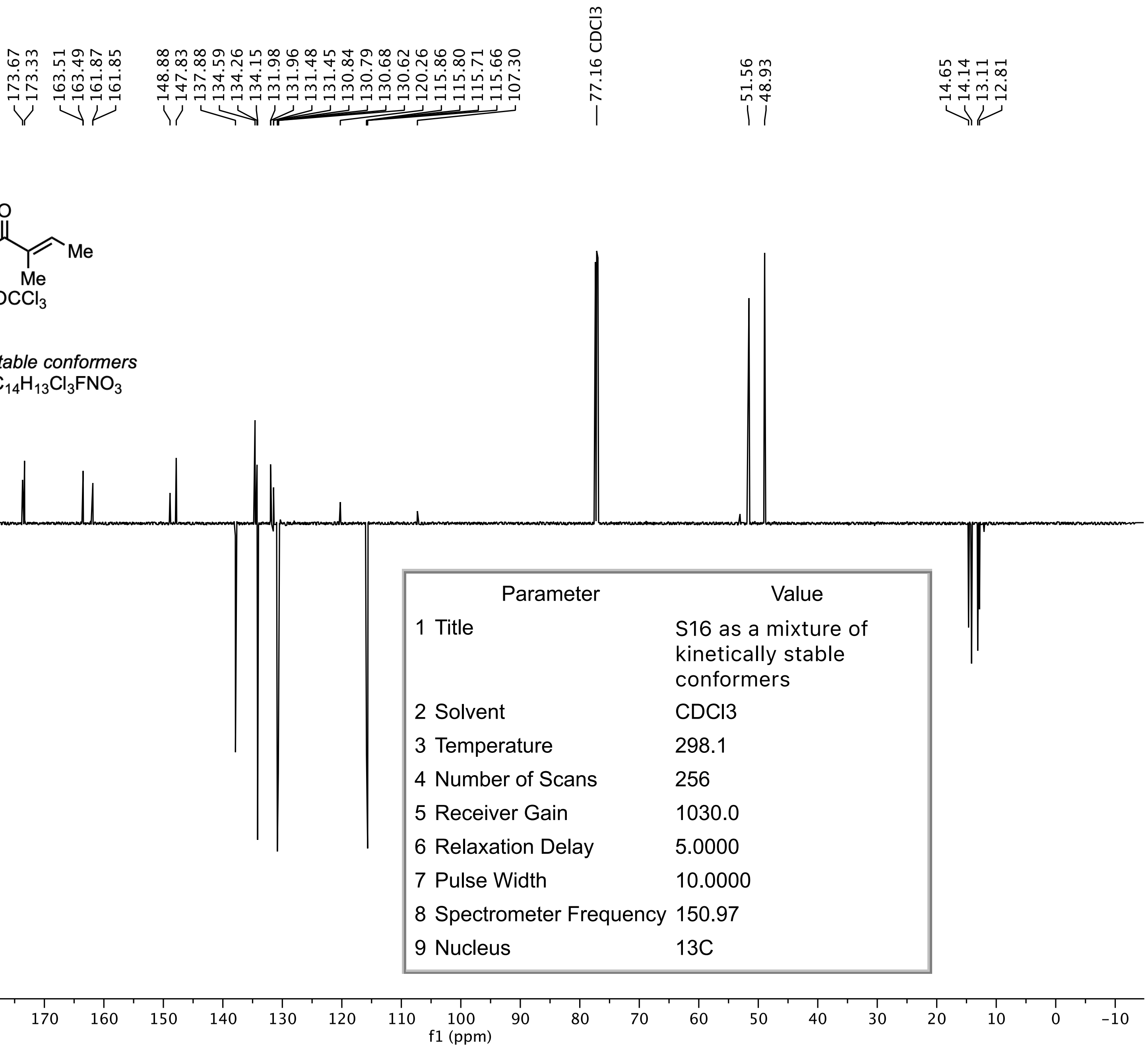


| | Parameter | Value |
|---|---|---|
| 1 | Title | S16 as a mixture of kinetically stable conformers |
| 2 | Solvent | CDCl3 |
| 3 | Temperature | 298.1 |
| 4 | Number of Scans | 256 |
| 5 | Receiver Gain | 1030.0 |
| 6 | Relaxation Delay | 5.0000 |
| 7 | Pulse Width | 10.0000 |
| 8 | Spectrometer Frequency | 150.97 |
| 9 | Nucleus | 13C |

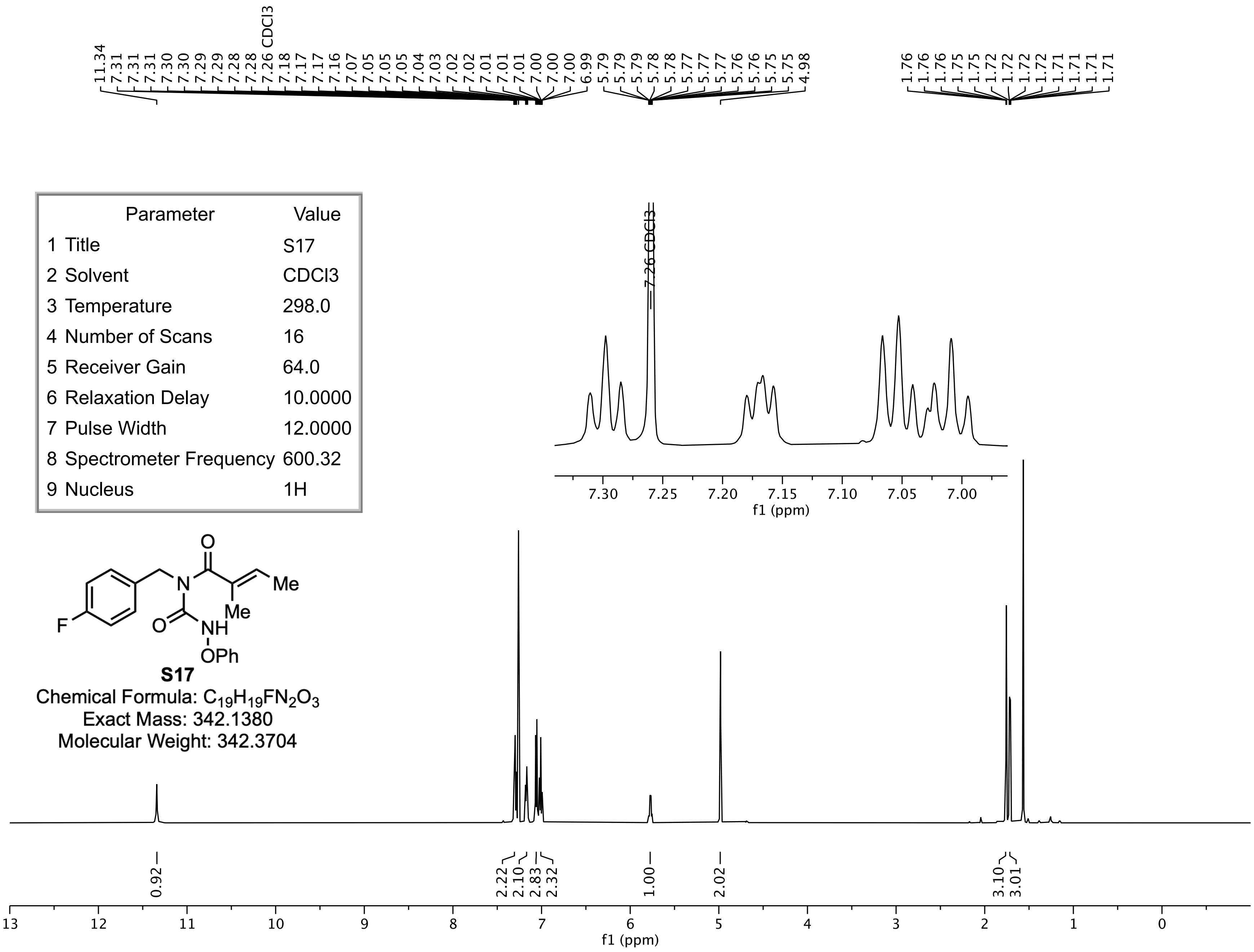


| Parameter | Value |
|---|---|
| 1 Title | S17 |
| 2 Solvent | CDCl3 |
| 3 Temperature | 298.0 |
| 4 Number of Scans | 16 |
| 5 Receiver Gain | 64.0 |
| 6 Relaxation Delay | 10.0000 |
| 7 Pulse Width | 12.0000 |
| 8 Spectrometer Frequency | 600.32 |
| 9 Nucleus | 1H |



**S17**

Chemical Formula: $C_{19}H_{19}FN_2O_3$

Exact Mass: 342.1380

Molecular Weight: 342.3704

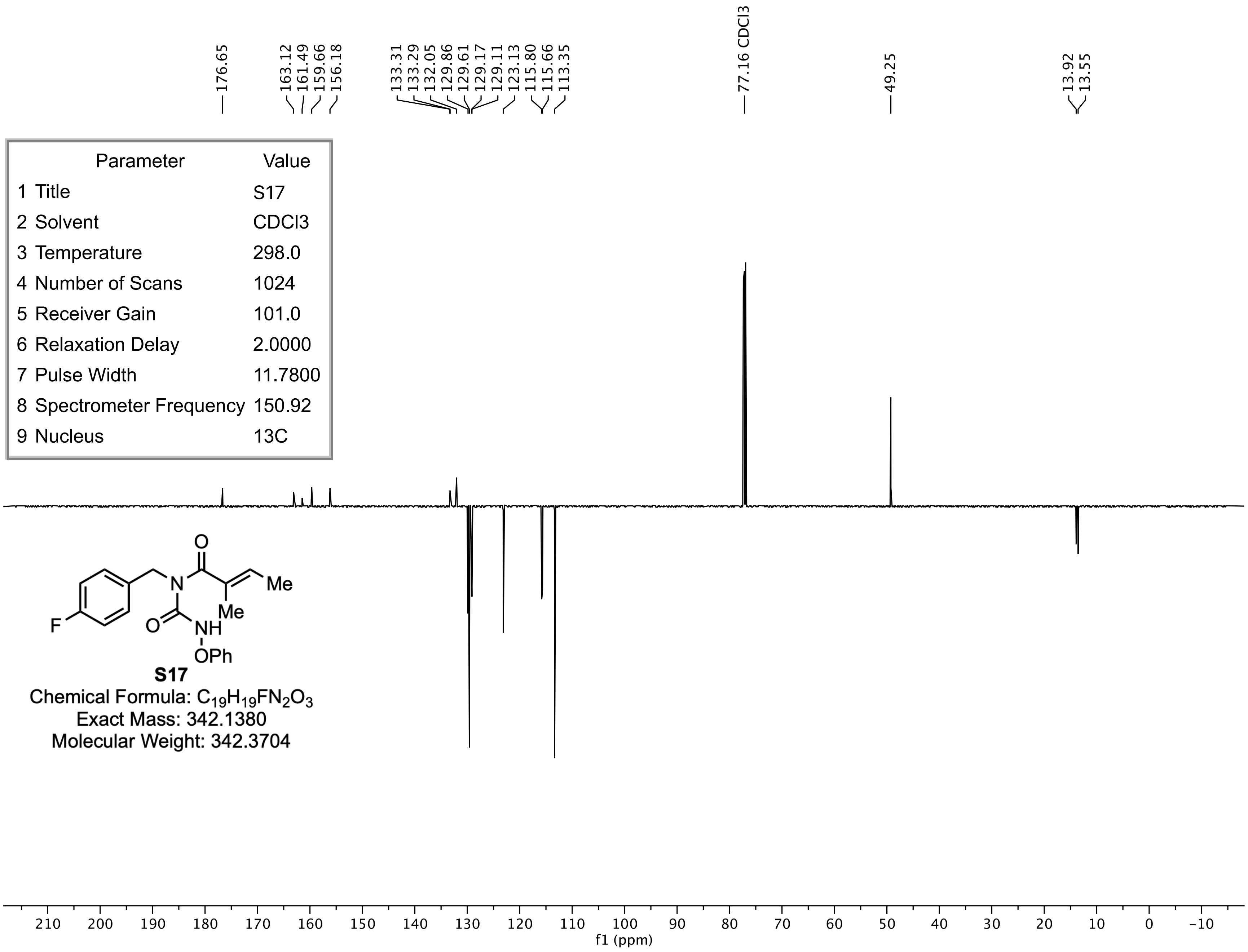


| Parameter | Value |
|---|---|
| 1 Title | S17 |
| 2 Solvent | CDCl3 |
| 3 Temperature | 298.0 |
| 4 Number of Scans | 1024 |
| 5 Receiver Gain | 101.0 |
| 6 Relaxation Delay | 2.0000 |
| 7 Pulse Width | 11.7800 |
| 8 Spectrometer Frequency | 150.92 |
| 9 Nucleus | 13C |

**S17**

Chemical Formula: $C_{19}H_{19}FN_2O_3$

Exact Mass: 342.1380

Molecular Weight: 342.3704

| | Parameter | Value |
|---|---|---|
| 1 | Title | S17 |
| 2 | Solvent | CDCl3 |
| 3 | Temperature | 298.1 |
| 4 | Number of Scans | 16 |
| 5 | Receiver Gain | 101.0 |
| 6 | Relaxation Delay | 1.0000 |
| 7 | Pulse Width | 15.0000 |
| 8 | Spectrometer Frequency | 376.46 |
| 9 | Nucleus | 19F |

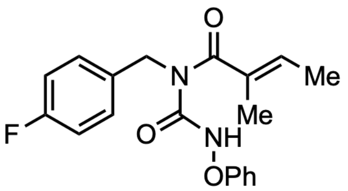


**S17**

Chemical Formula: $C_{19}H_{19}FN_2O_3$
Exact Mass: 342.1380
Molecular Weight: 342.3704

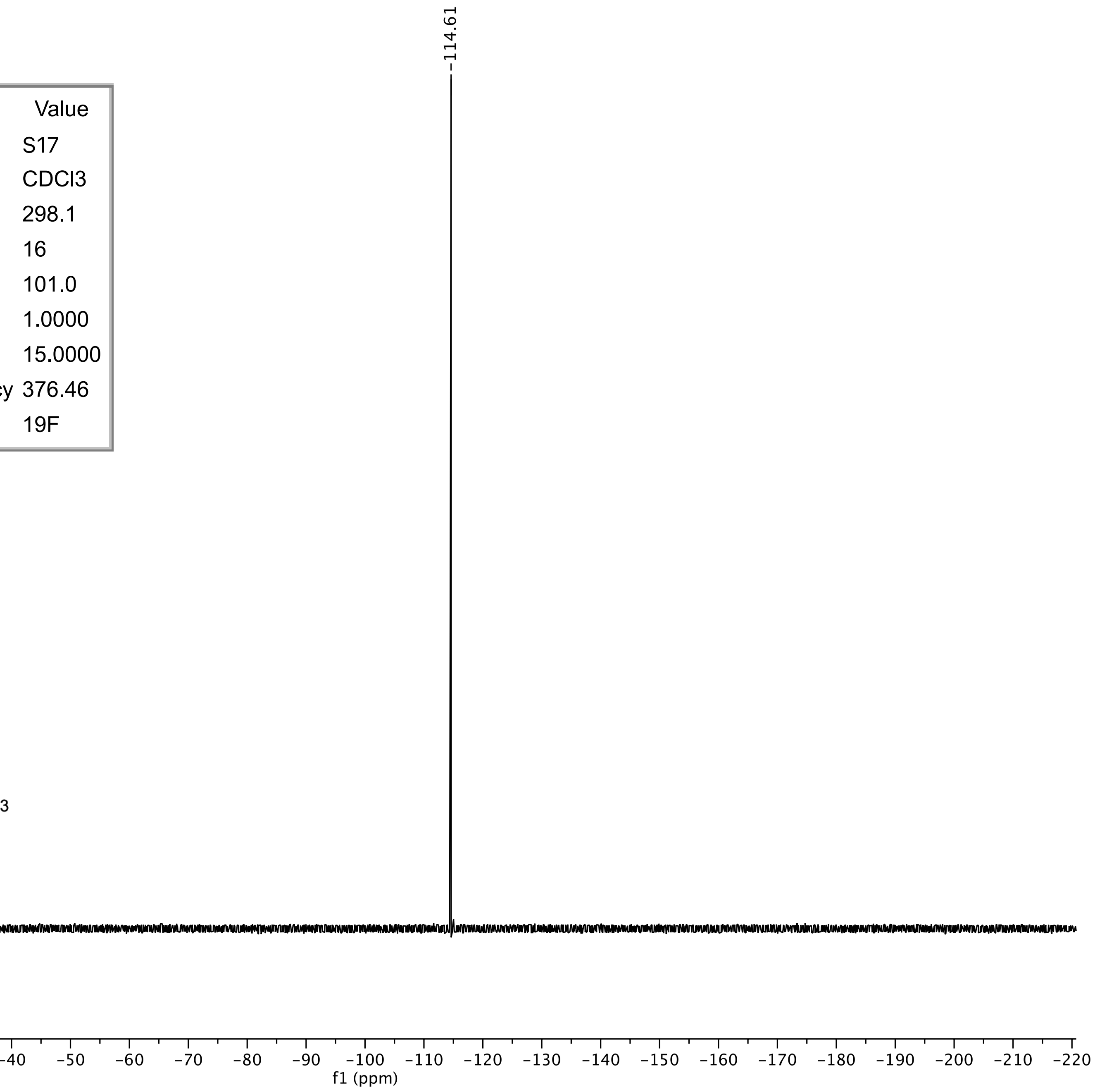

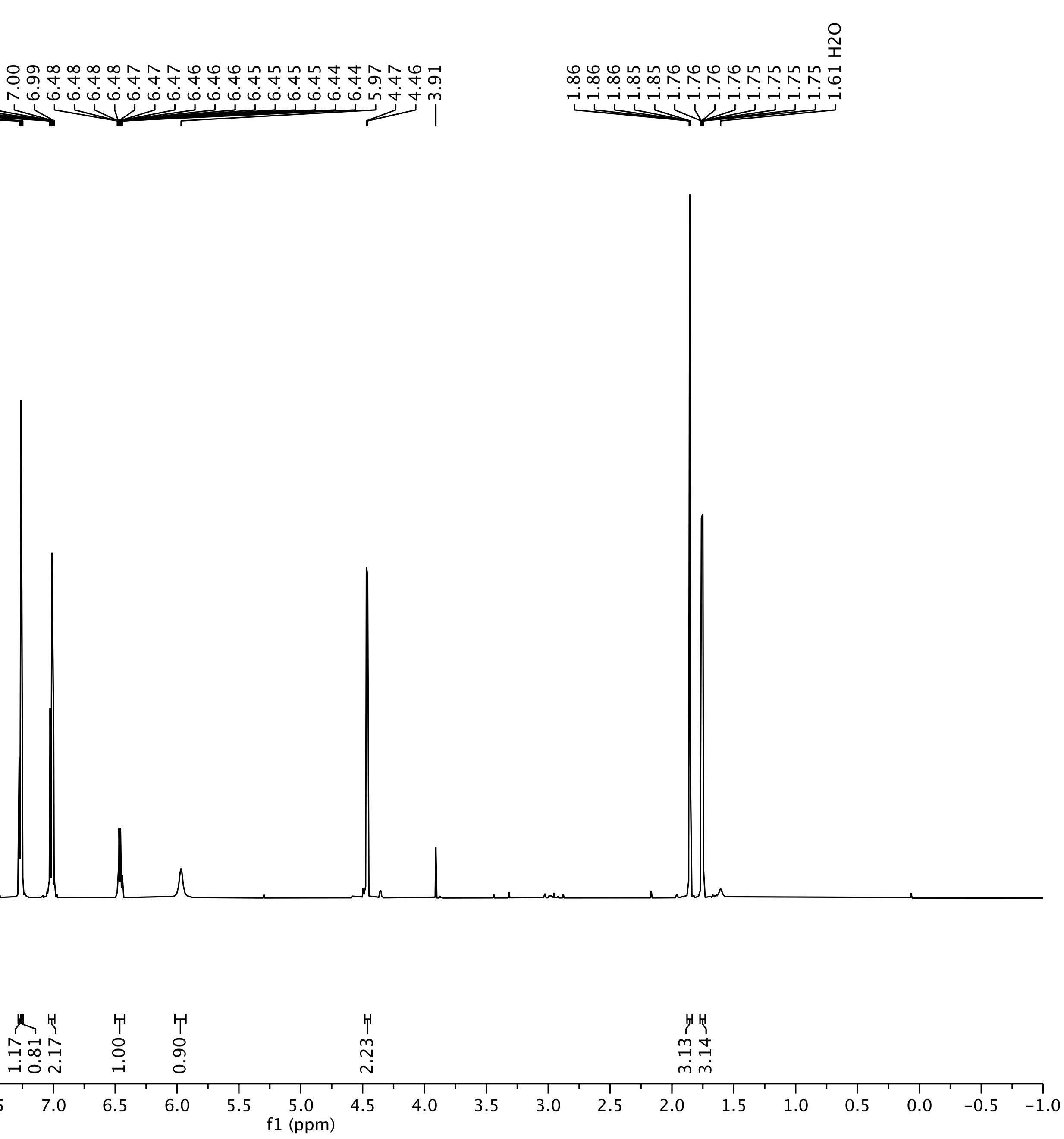


| Parameter | Value |
|---|---|
| 1 Title | S18 |
| 2 Solvent | CDCl3 |
| 3 Temperature | 298.0 |
| 4 Number of Scans | 16 |
| 5 Receiver Gain | 12.7 |
| 6 Relaxation Delay | 10.0000 |
| 7 Pulse Width | 8.0100 |
| 8 Spectrometer Frequency | 600.13 |
| 9 Nucleus | 1H |

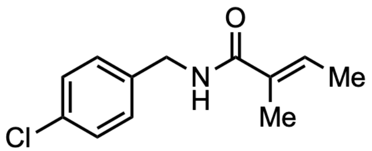


**S18**

Chemical Formula: $C_{12}H_{14}ClNO$

Exact Mass: 223.0764

Molecular Weight: 223.7000

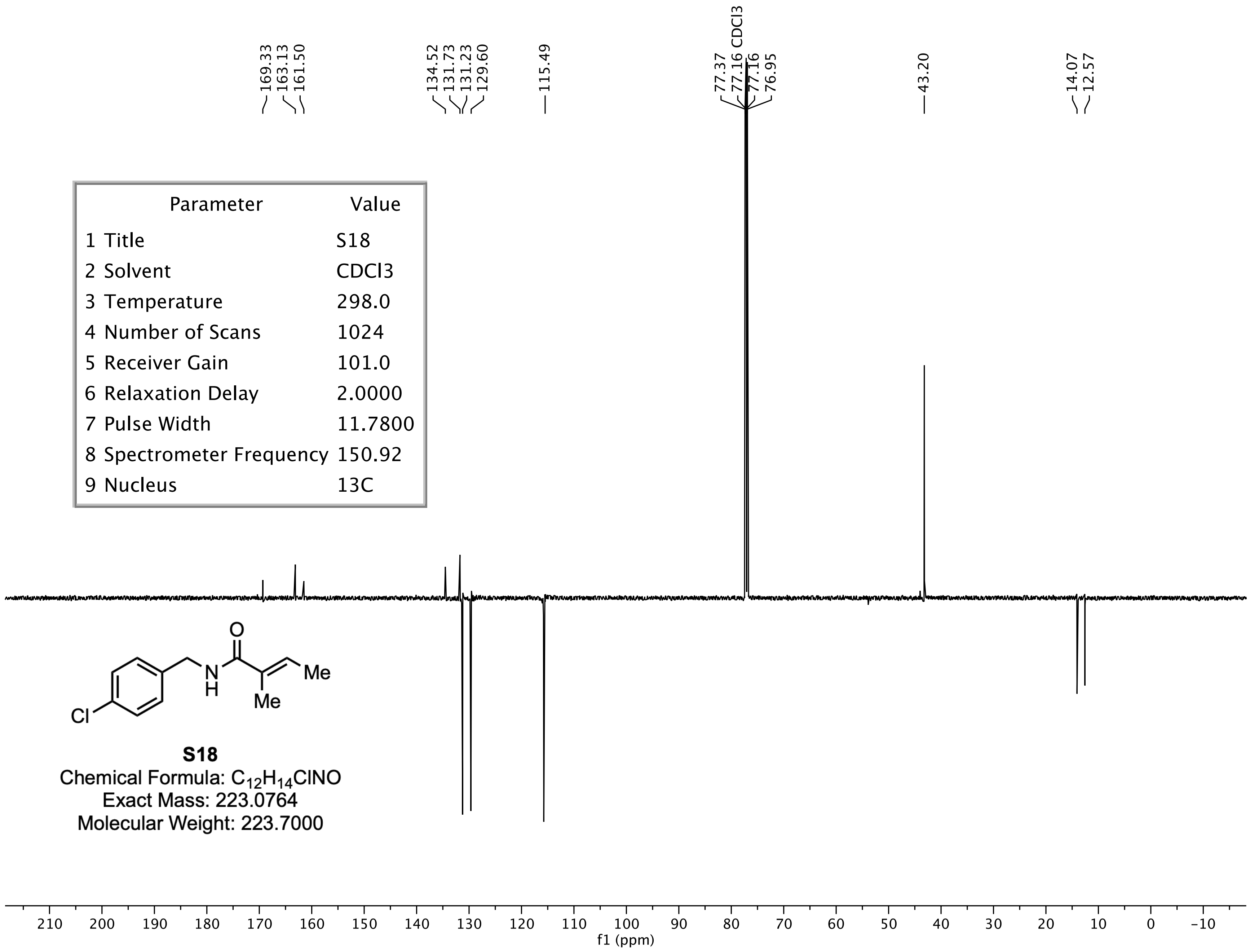


| Parameter | Value |
|---|---|
| 1 Title | S18 |
| 2 Solvent | CDCl3 |
| 3 Temperature | 298.0 |
| 4 Number of Scans | 1024 |
| 5 Receiver Gain | 101.0 |
| 6 Relaxation Delay | 2.0000 |
| 7 Pulse Width | 11.7800 |
| 8 Spectrometer Frequency | 150.92 |
| 9 Nucleus | 13C |

**S18**
Chemical Formula: $C_{12}H_{14}ClNO$
Exact Mass: 223.0764
Molecular Weight: 223.7000

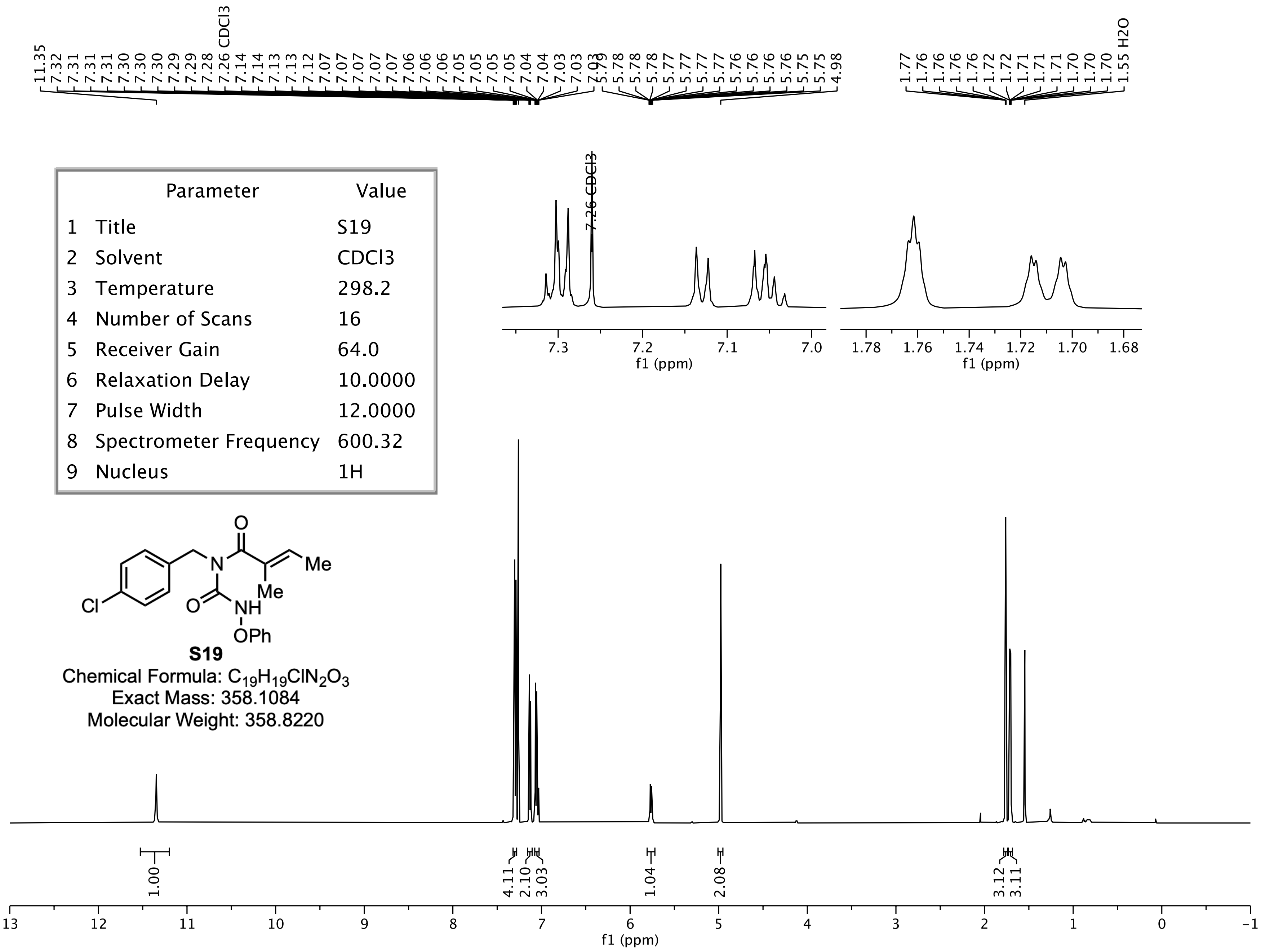


| | Parameter | Value |
|---|---|---|
| 1 | Title | S19 |
| 2 | Solvent | CDCl3 |
| 3 | Temperature | 298.2 |
| 4 | Number of Scans | 16 |
| 5 | Receiver Gain | 64.0 |
| 6 | Relaxation Delay | 10.0000 |
| 7 | Pulse Width | 12.0000 |
| 8 | Spectrometer Frequency | 600.32 |
| 9 | Nucleus | 1H |

**S19**

Chemical Formula: $C_{19}H_{19}ClN_2O_3$
Exact Mass: 358.1084
Molecular Weight: 358.8220

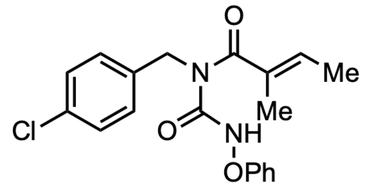


**S19**

Chemical Formula: $C_{19}H_{19}ClN_2O_3$
Exact Mass: 358.1084
Molecular Weight: 358.8220

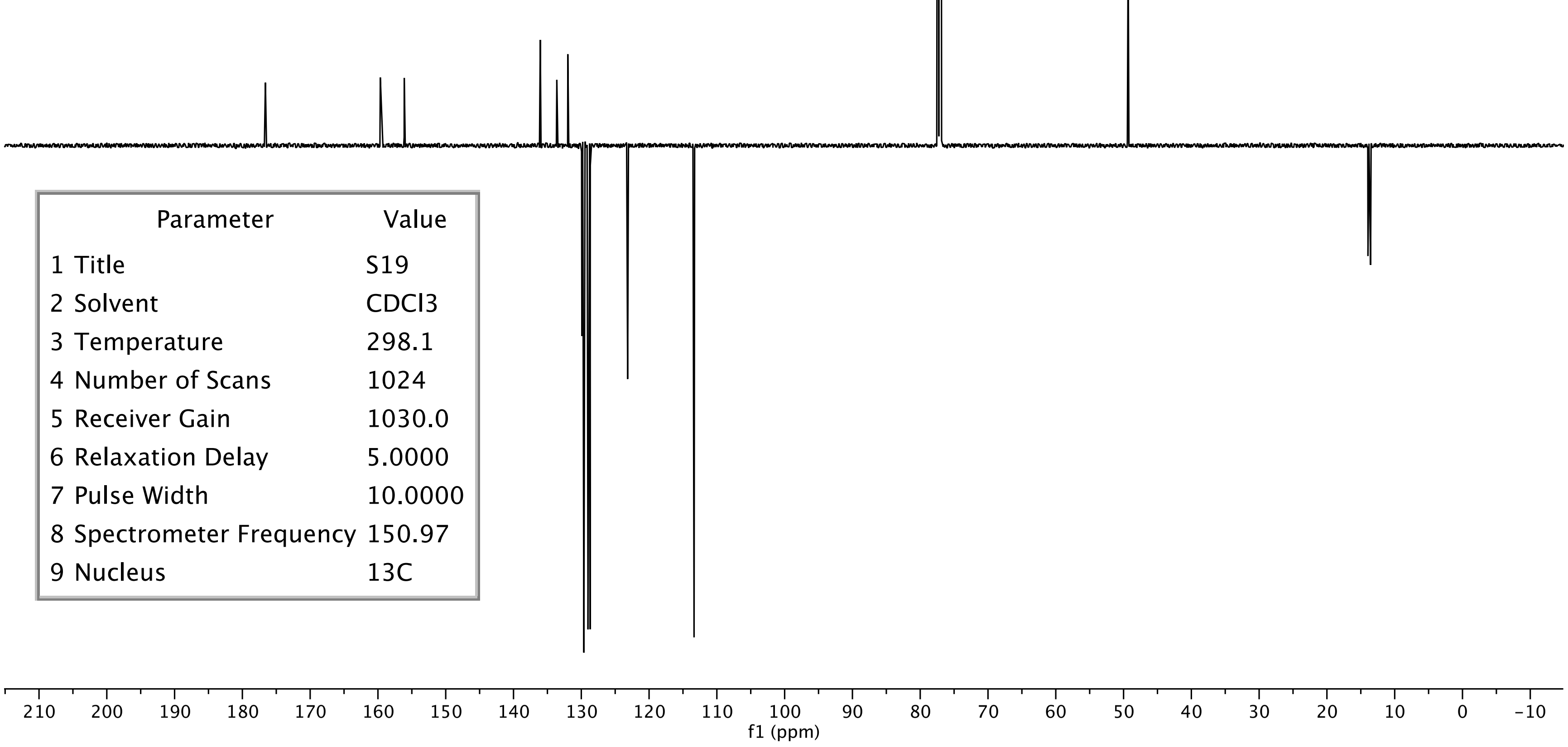


| Parameter | Value |
|---|---|
| 1 Title | S19 |
| 2 Solvent | CDCl3 |
| 3 Temperature | 298.1 |
| 4 Number of Scans | 1024 |
| 5 Receiver Gain | 1030.0 |
| 6 Relaxation Delay | 5.0000 |
| 7 Pulse Width | 10.0000 |
| 8 Spectrometer Frequency | 150.97 |
| 9 Nucleus | 13C |

| Parameter | Value |
|---|---|
| 1 Title | S20 |
| 2 Solvent | CDCl3 |
| 3 Temperature | 298.0 |
| 4 Number of Scans | 16 |
| 5 Receiver Gain | 12.7 |
| 6 Relaxation Delay | 10.0000 |
| 7 Pulse Width | 8.0100 |
| 8 Spectrometer Frequency | 600.13 |
| 9 Nucleus | 1H |

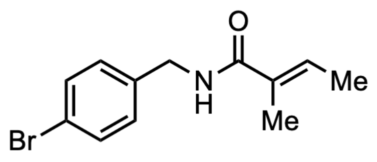


**S20**
Chemical Formula: $C_{12}H_{14}BrNO$
Exact Mass: 267.0259
Molecular Weight: 268.1540

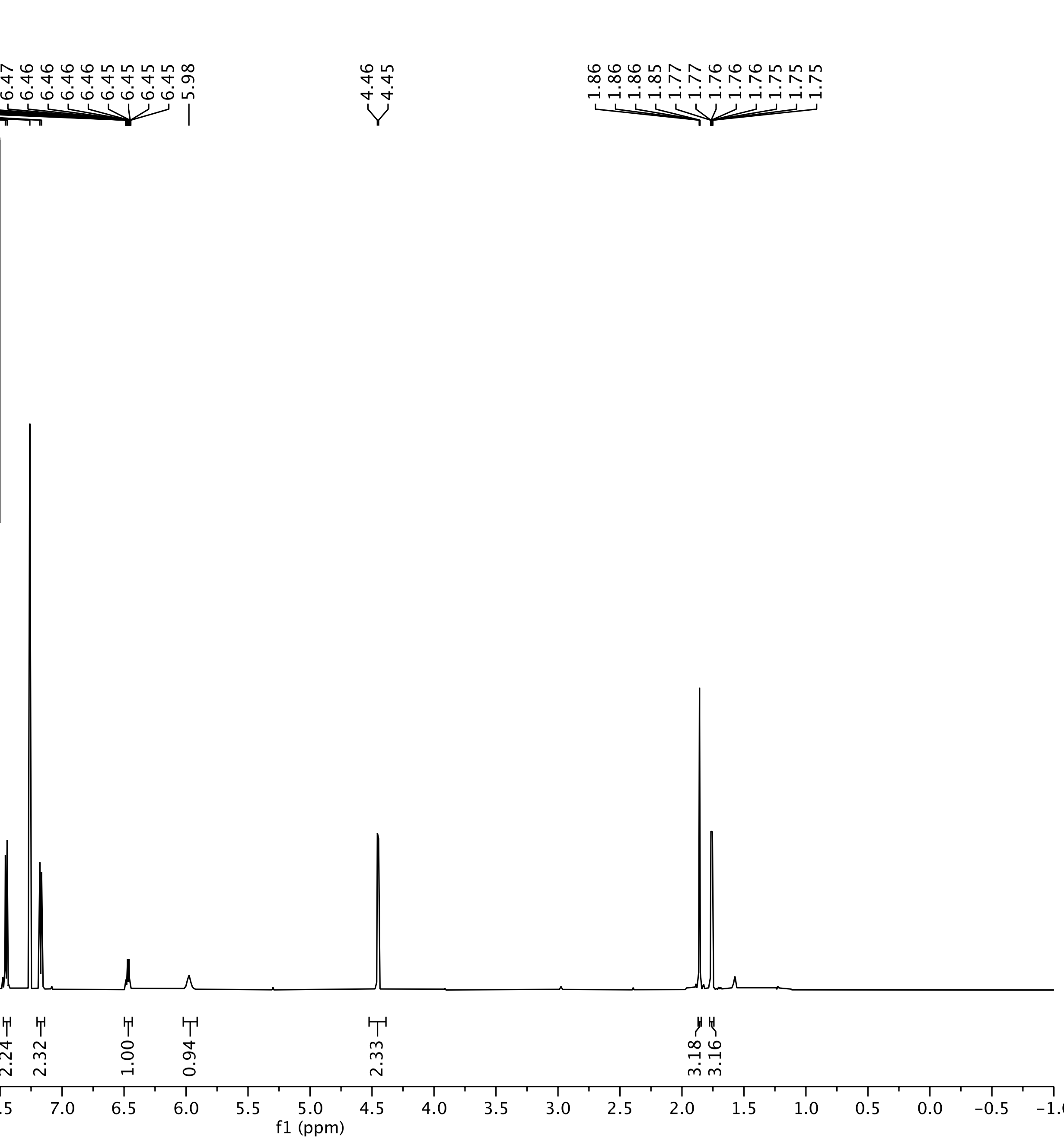

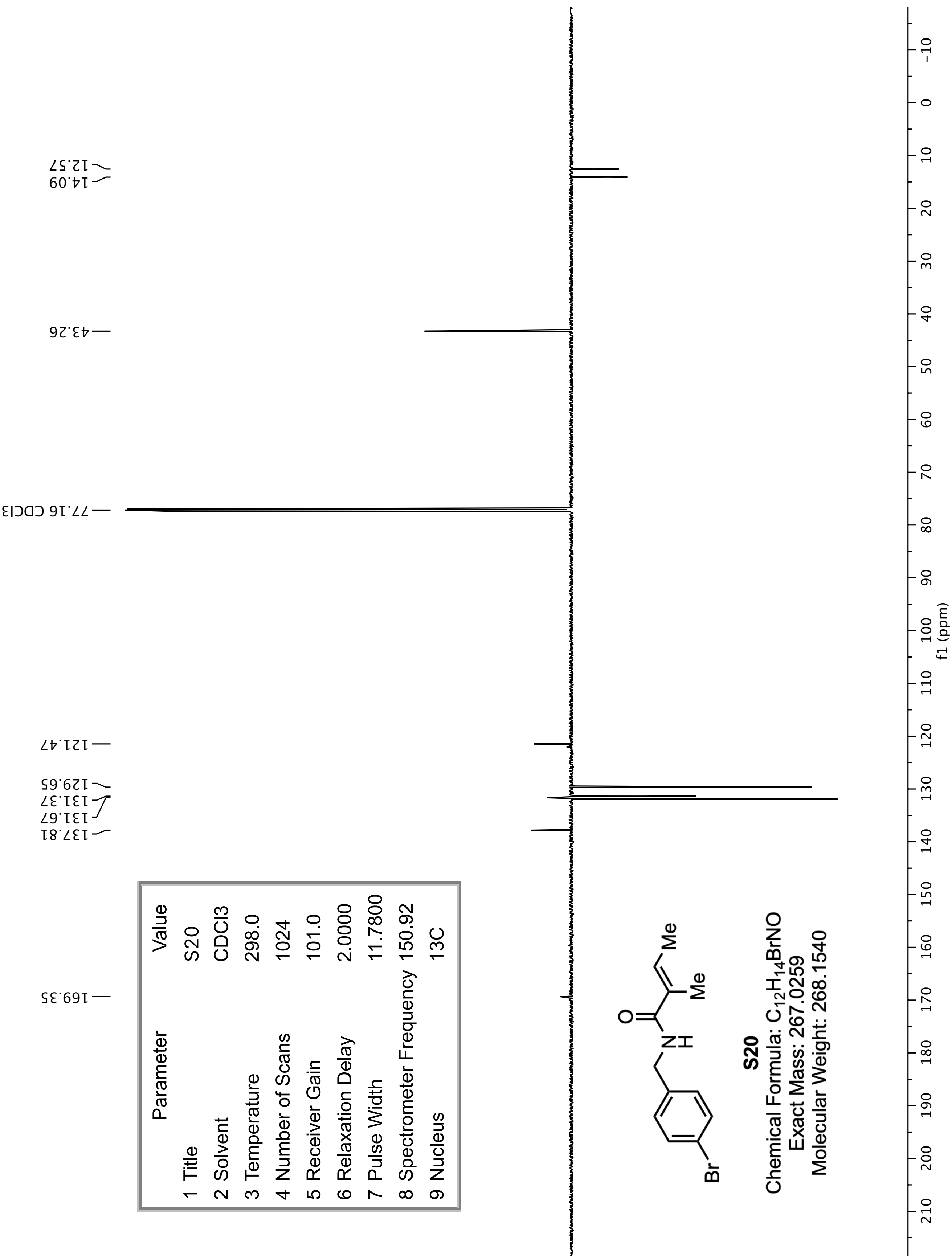


| | Parameter | Value |
| --- | --- | --- |
| 1 | Title | S20 |
| 2 | Solvent | CDCl3 |
| 3 | Temperature | 298.0 |
| 4 | Number of Scans | 1024 |
| 5 | Receiver Gain | 101.0 |
| 6 | Relaxation Delay | 2.0000 |
| 7 | Pulse Width | 11.7800 |
| 8 | Spectrometer Frequency | 150.92 |
| 9 | Nucleus | 13C |

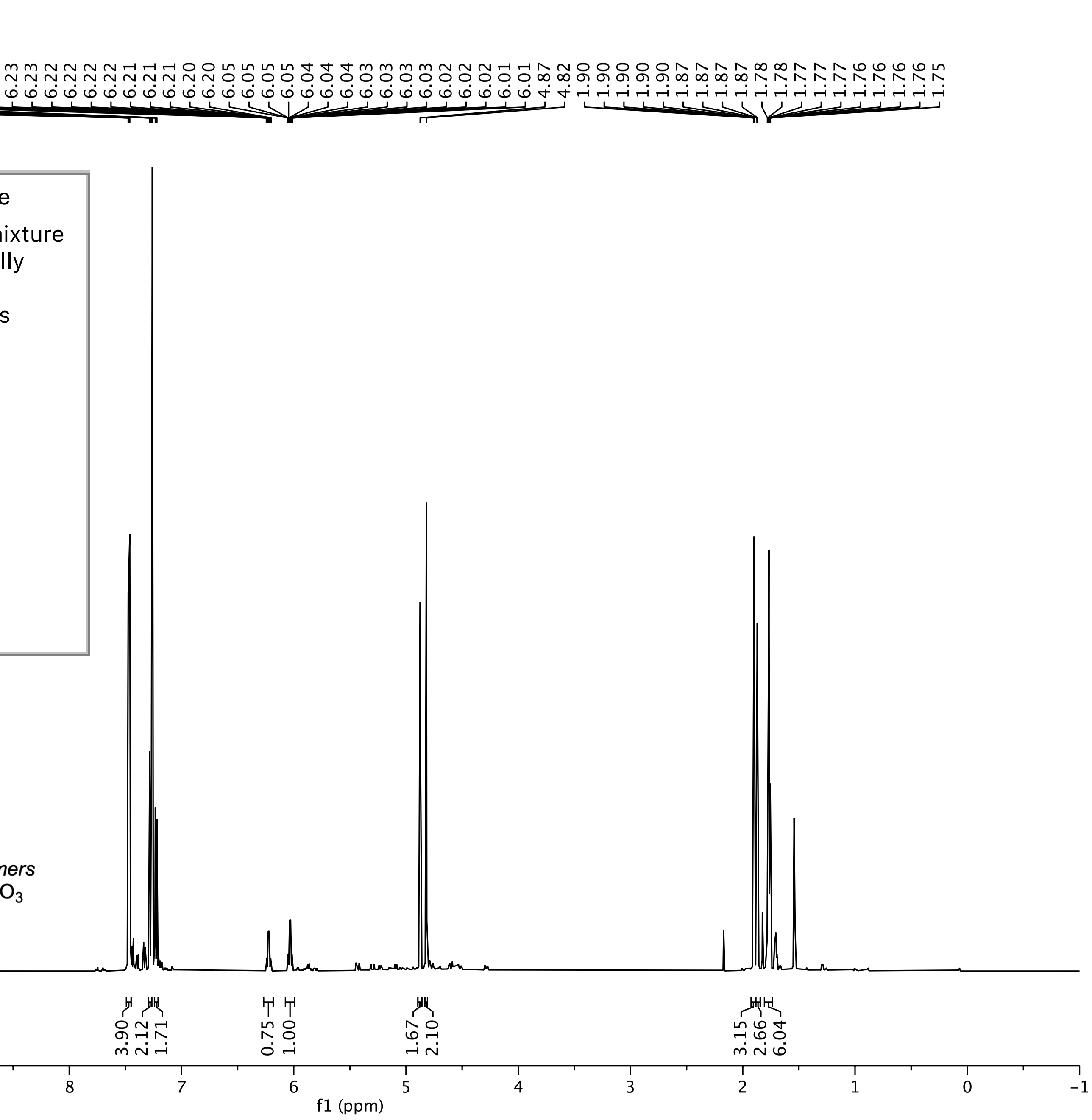


| Parameter | Value |
|---|---|
| 1 Title | S21 as a mixture of kinetically stable conformers |
| 2 Solvent | CDCl3 |
| 3 Temperature | 298.0 |
| 4 Number of Scans | 16 |
| 5 Receiver Gain | 12.7 |
| 6 Relaxation Delay | 10.0000 |
| 7 Pulse Width | 8.0100 |
| 8 Spectrometer Frequency | 600.13 |
| 9 Nucleus | 1H |

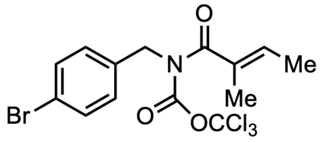


**S21**

*Mixture of kinetically stable conformers*

Chemical Formula: $C_{14}H_{13}BrCl_3NO_3$

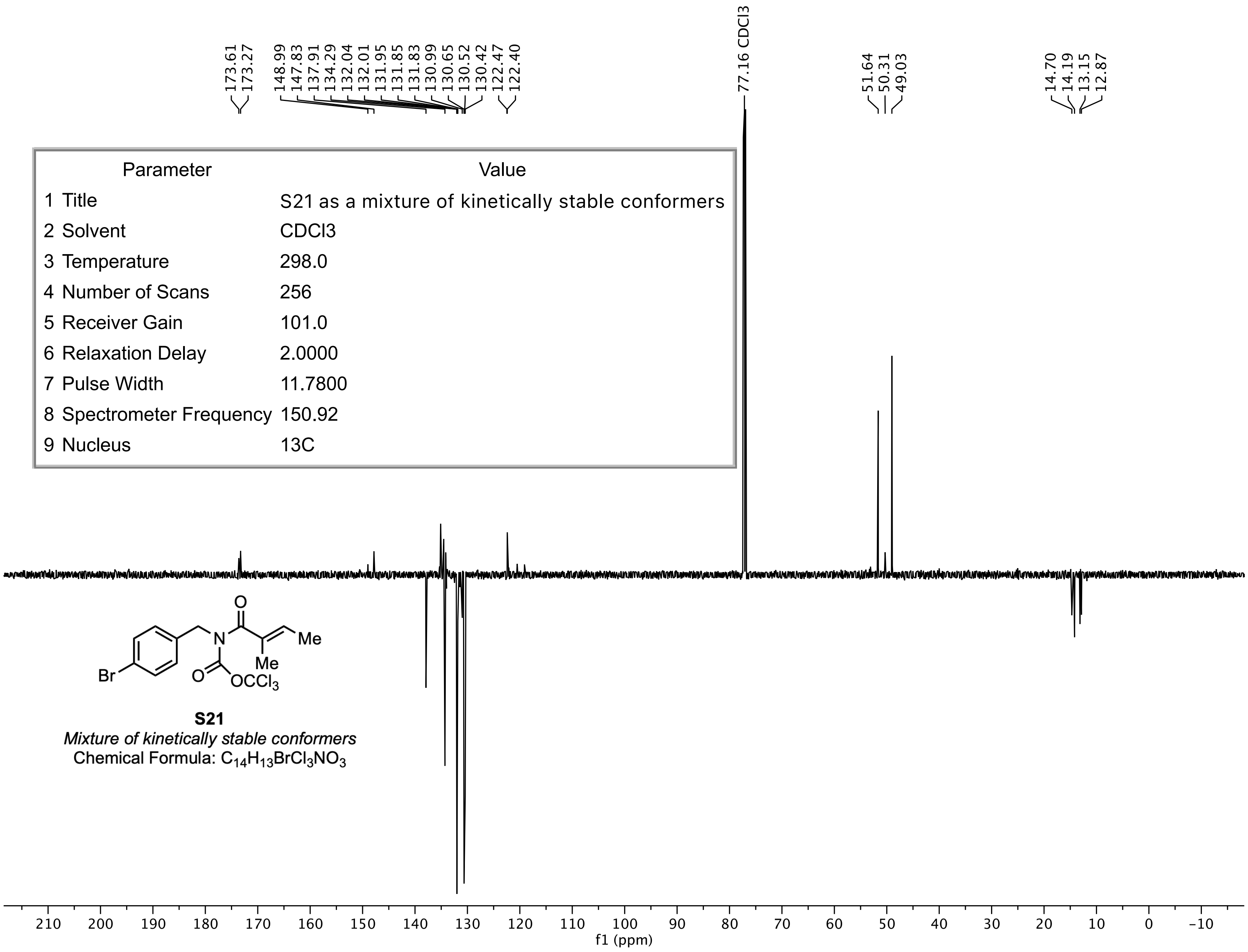


| Parameter | Value |
|---|---|
| 1 Title | S21 as a mixture of kinetically stable conformers |
| 2 Solvent | CDCl3 |
| 3 Temperature | 298.0 |
| 4 Number of Scans | 256 |
| 5 Receiver Gain | 101.0 |
| 6 Relaxation Delay | 2.0000 |
| 7 Pulse Width | 11.7800 |
| 8 Spectrometer Frequency | 150.92 |
| 9 Nucleus | 13C |

**S21**

*Mixture of kinetically stable conformers*

Chemical Formula: $C_{14}H_{13}BrCl_3NO_3$

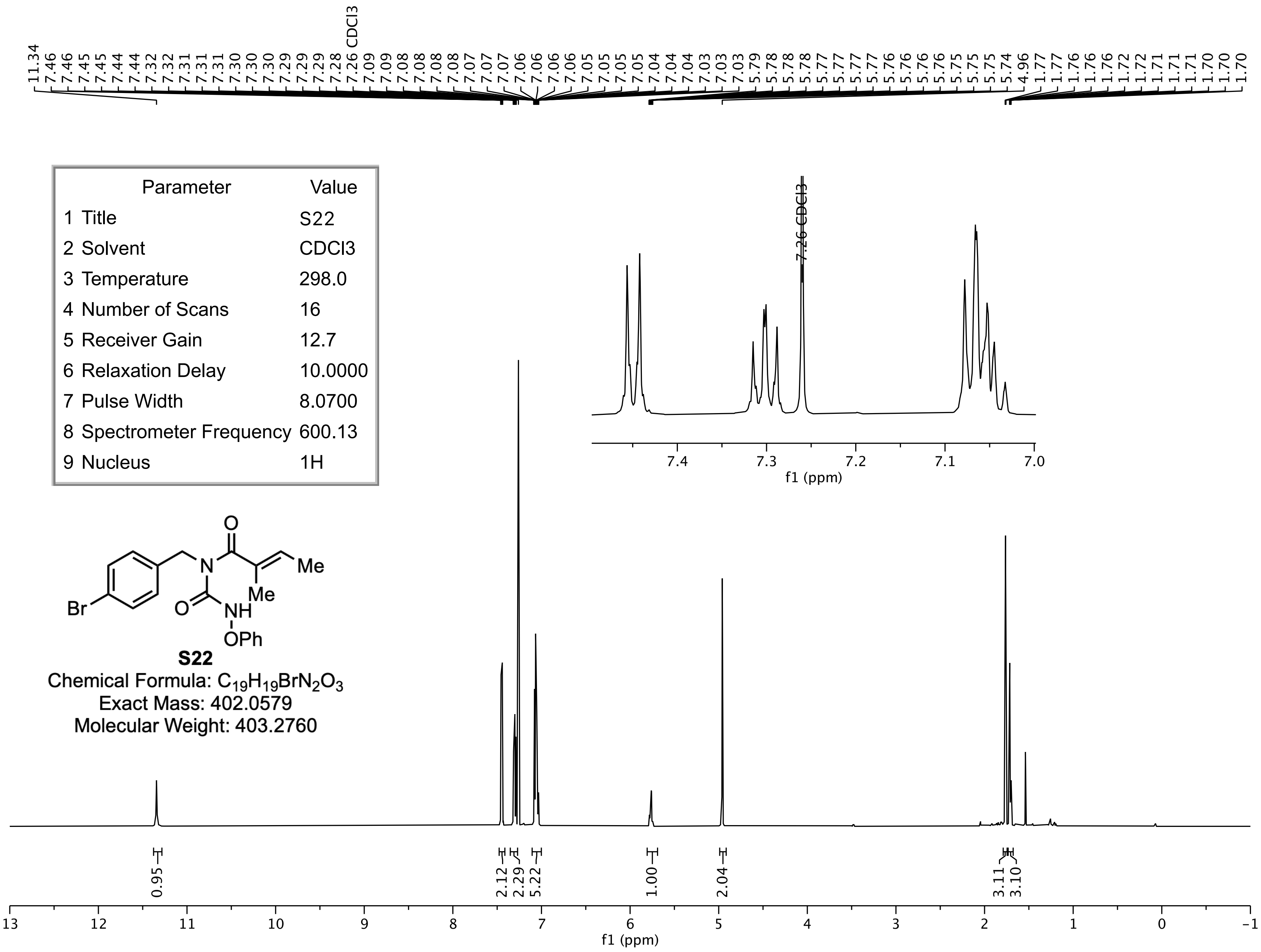


| Parameter | Value |
| --- | --- |
| 1 Title | S22 |
| 2 Solvent | CDCl3 |
| 3 Temperature | 298.0 |
| 4 Number of Scans | 16 |
| 5 Receiver Gain | 12.7 |
| 6 Relaxation Delay | 10.0000 |
| 7 Pulse Width | 8.0700 |
| 8 Spectrometer Frequency | 600.13 |
| 9 Nucleus | 1H |

**S22**

Chemical Formula: $C_{19}H_{19}BrN_2O_3$

Exact Mass: 402.0579

Molecular Weight: 403.2760

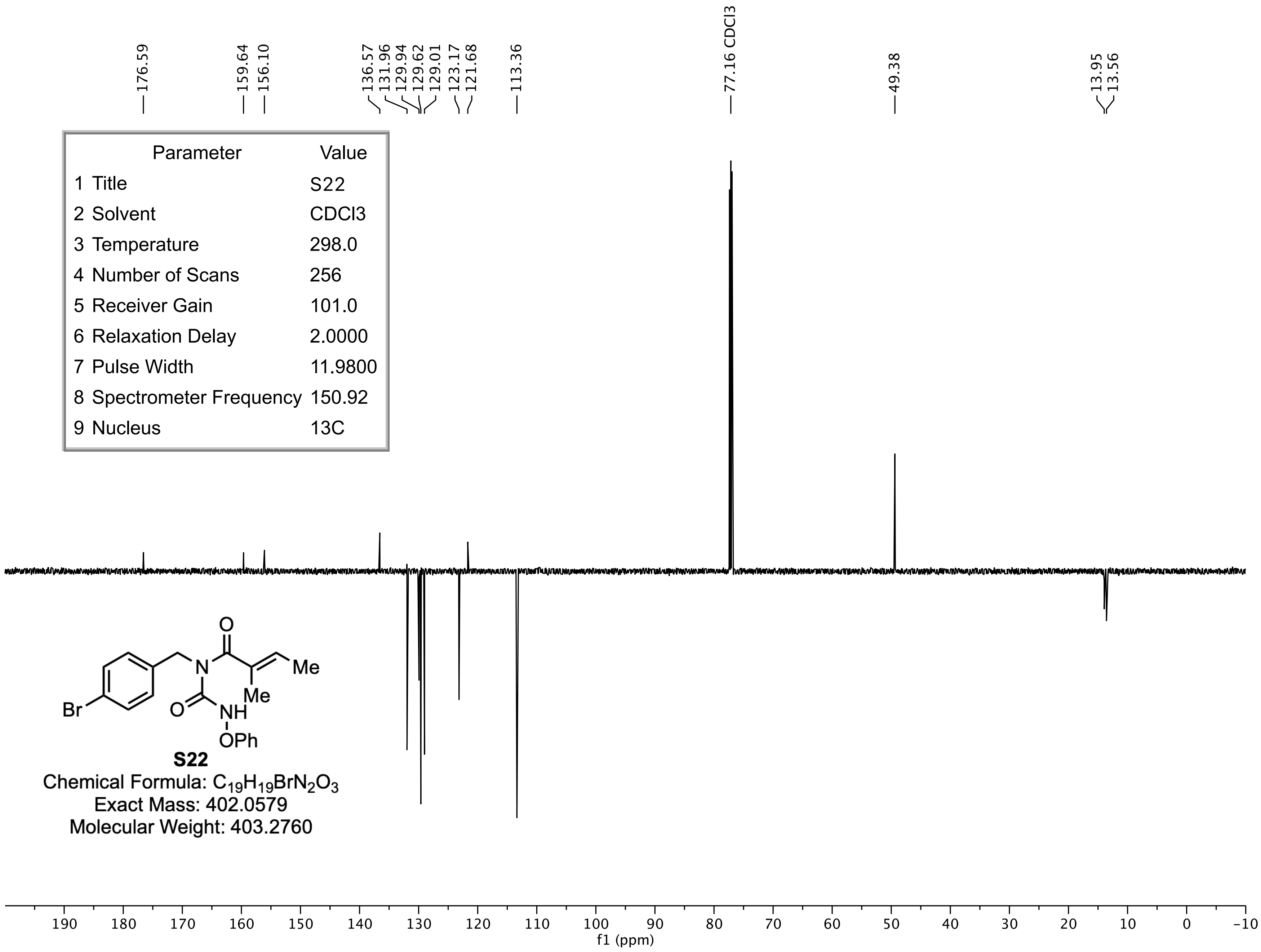


| Parameter | Value |
|---|---|
| 1 Title | S22 |
| 2 Solvent | CDCl3 |
| 3 Temperature | 298.0 |
| 4 Number of Scans | 256 |
| 5 Receiver Gain | 101.0 |
| 6 Relaxation Delay | 2.0000 |
| 7 Pulse Width | 11.9800 |
| 8 Spectrometer Frequency | 150.92 |
| 9 Nucleus | 13C |



**S22**

Chemical Formula: $C_{19}H_{19}BrN_2O_3$

Exact Mass: 402.0579

Molecular Weight: 403.2760

| Parameter | Value |
|---|---|
| 1 Title | S23 |
| 2 Solvent | CDCl3 |
| 3 Temperature | 298.0 |
| 4 Number of Scans | 16 |
| 5 Receiver Gain | 25.0 |
| 6 Relaxation Delay | 1.0000 |
| 7 Pulse Width | 7.8100 |
| 8 Spectrometer Frequency | 600.13 |
| 9 Nucleus | 1H |

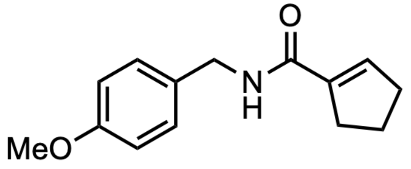


**S23**
Chemical Formula: $C_{14}H_{17}NO_2$
Exact Mass: 231.1259
Molecular Weight: 231.2950

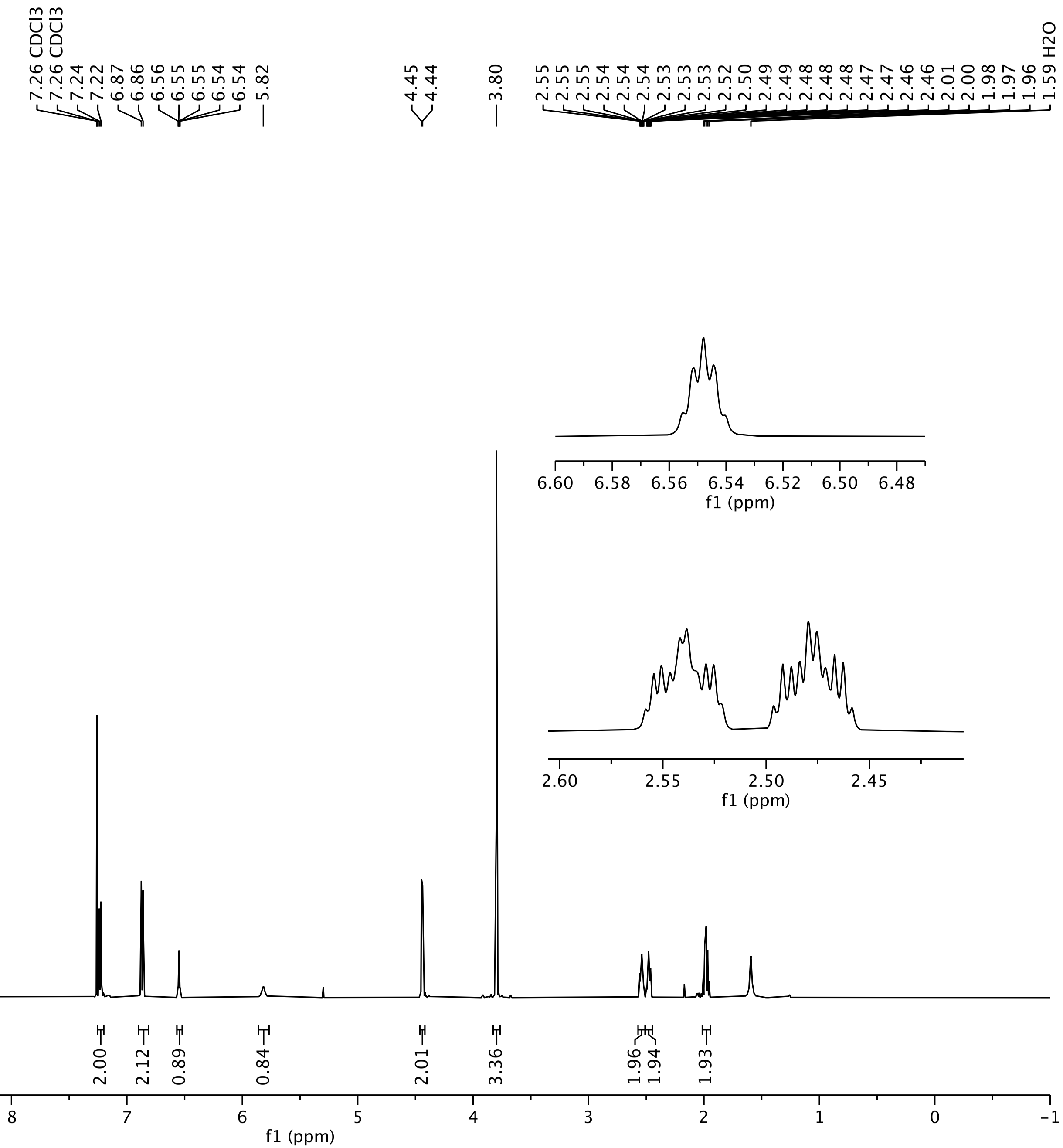

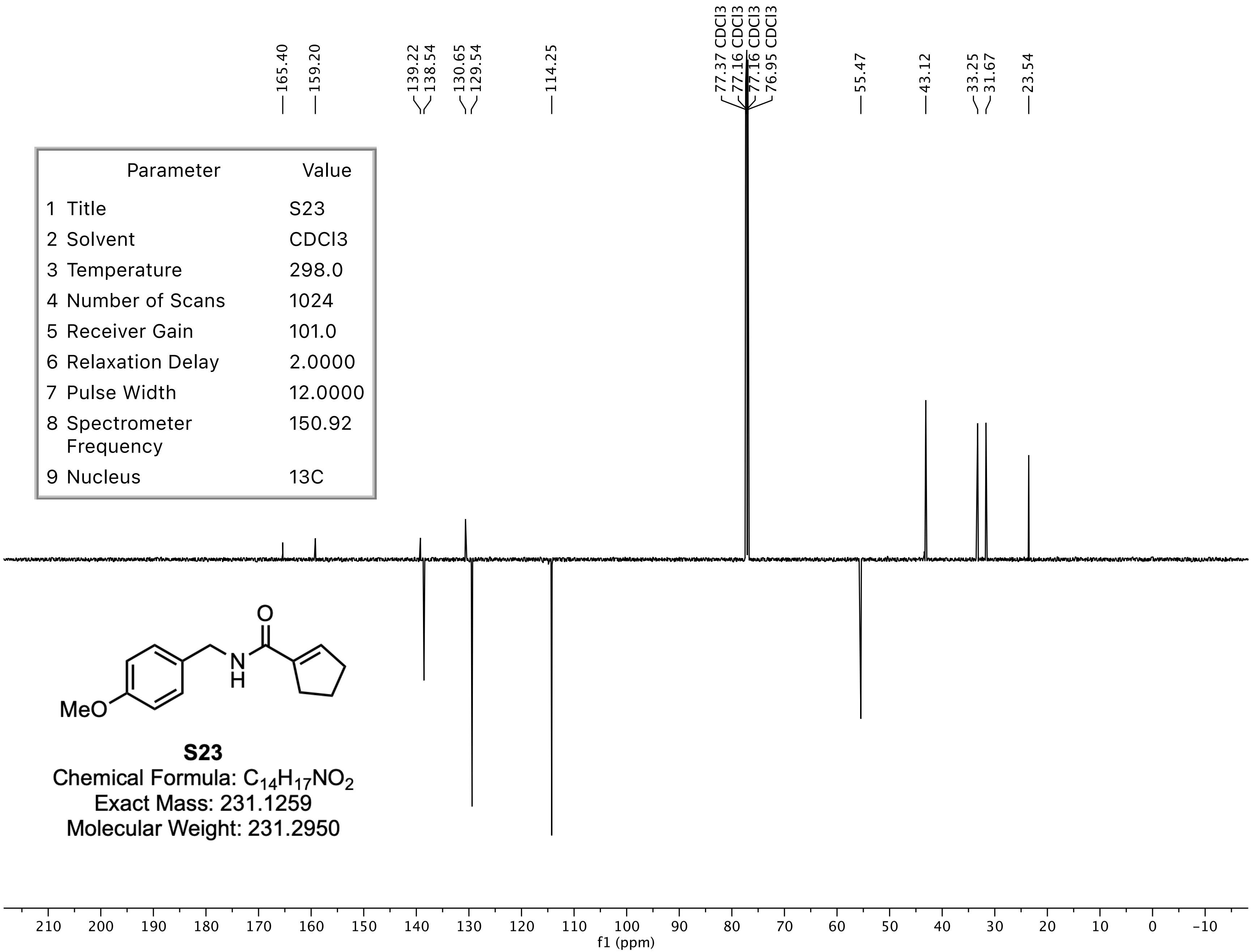


| | Parameter | Value |
|---|---|---|
| 1 | Title | S23 |
| 2 | Solvent | CDCl3 |
| 3 | Temperature | 298.0 |
| 4 | Number of Scans | 1024 |
| 5 | Receiver Gain | 101.0 |
| 6 | Relaxation Delay | 2.0000 |
| 7 | Pulse Width | 12.0000 |
| 8 | Spectrometer Frequency | 150.92 |
| 9 | Nucleus | 13C |



**S23**

Chemical Formula: $C_{14}H_{17}NO_2$

Exact Mass: 231.1259

Molecular Weight: 231.2950

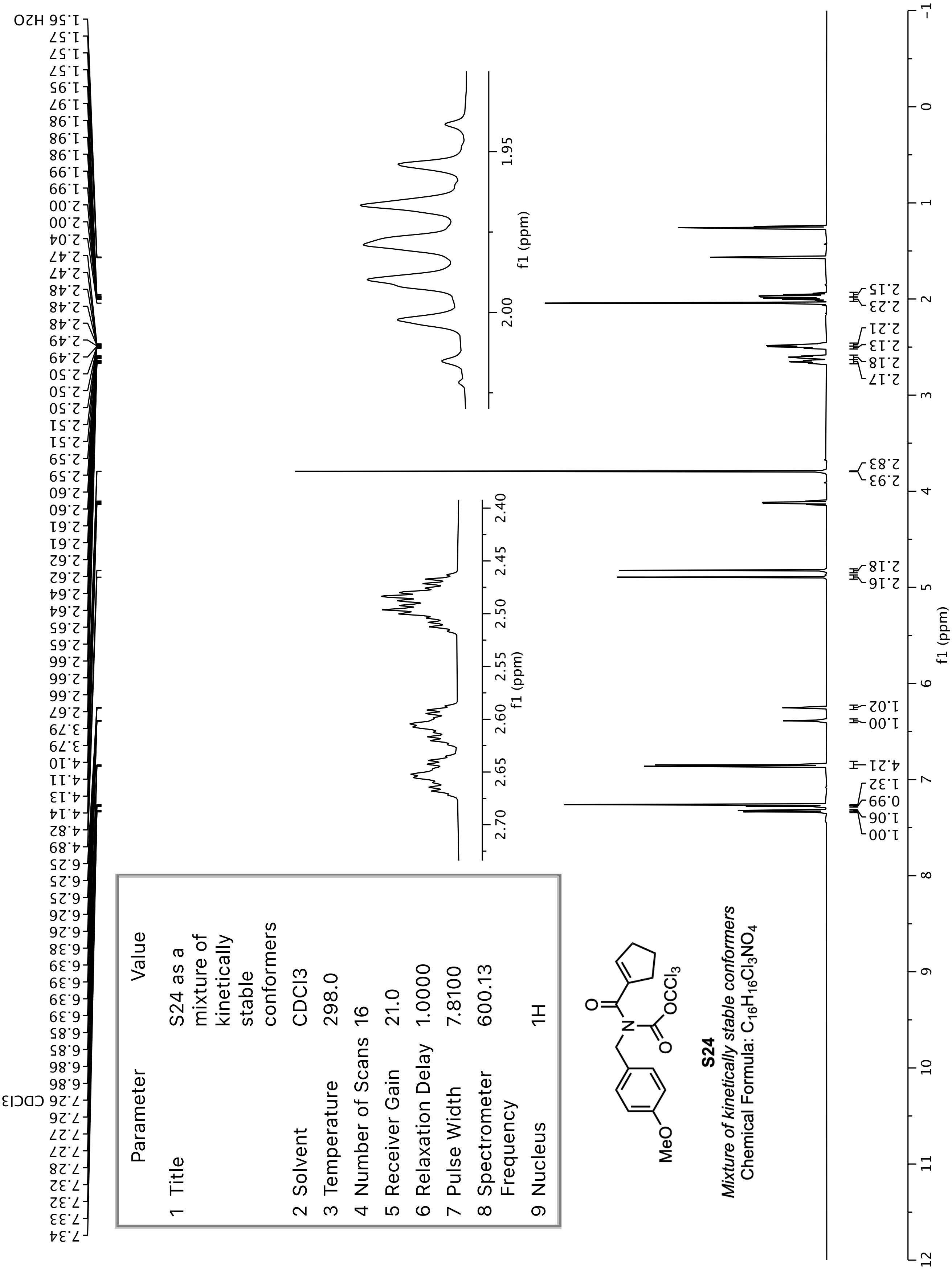


| Parameter | Value |
|---|---|
| 1 Title | S24 as a mixture of kinetically stable conformers |
| 2 Solvent | CDCl3 |
| 3 Temperature | 298.0 |
| 4 Number of Scans | 16 |
| 5 Receiver Gain | 21.0 |
| 6 Relaxation Delay | 1.0000 |
| 7 Pulse Width | 7.8100 |
| 8 Spectrometer Frequency | 600.13 |
| 9 Nucleus | 1H |

**S24**

*Mixture of kinetically stable conformers*

Chemical Formula: $C_{16}H_{16}Cl_3NO_4$

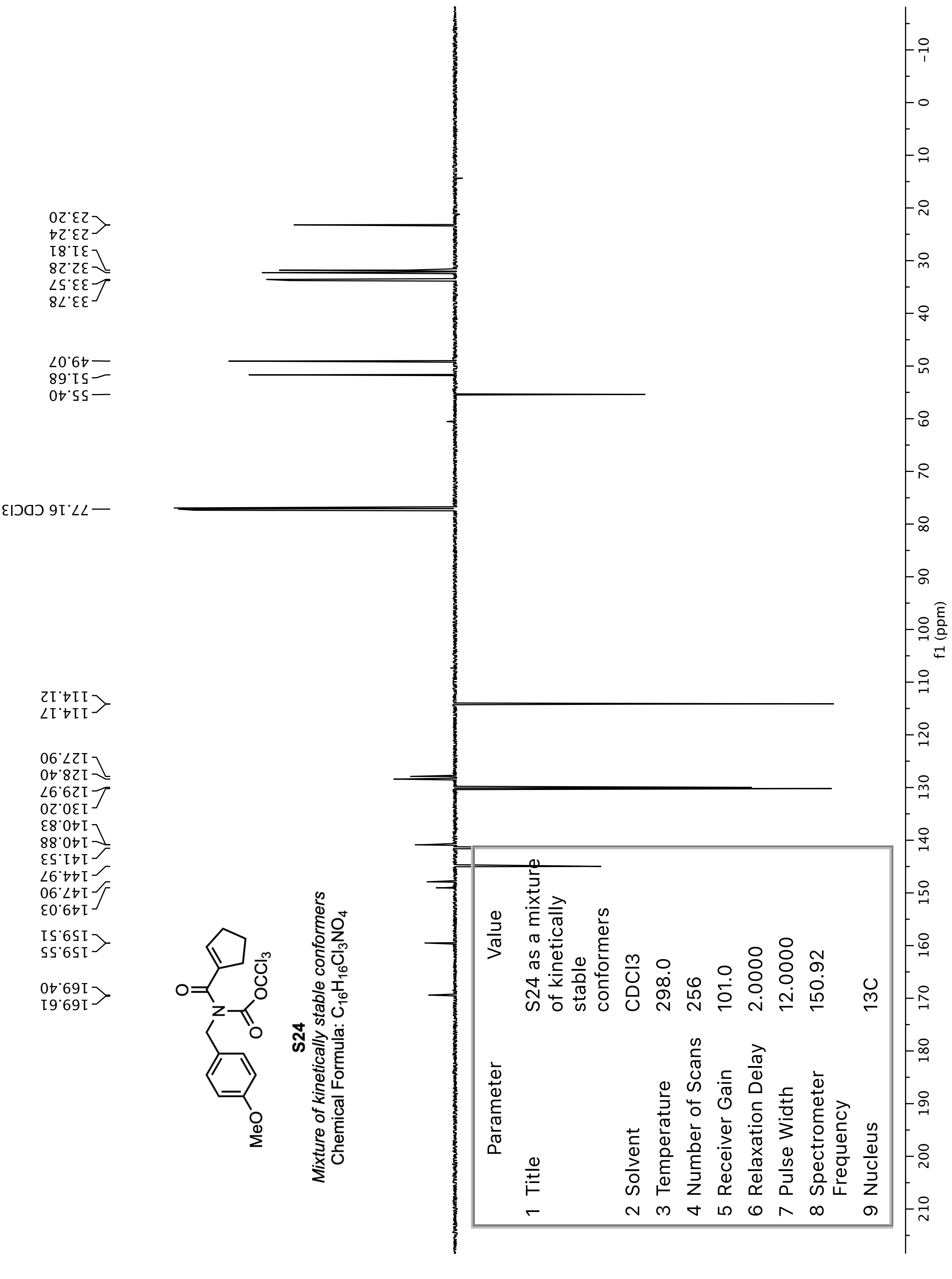


**S24**

*Mixture of kinetically stable conformers*

Chemical Formula: $C_{16}H_{16}Cl_3NO_4$

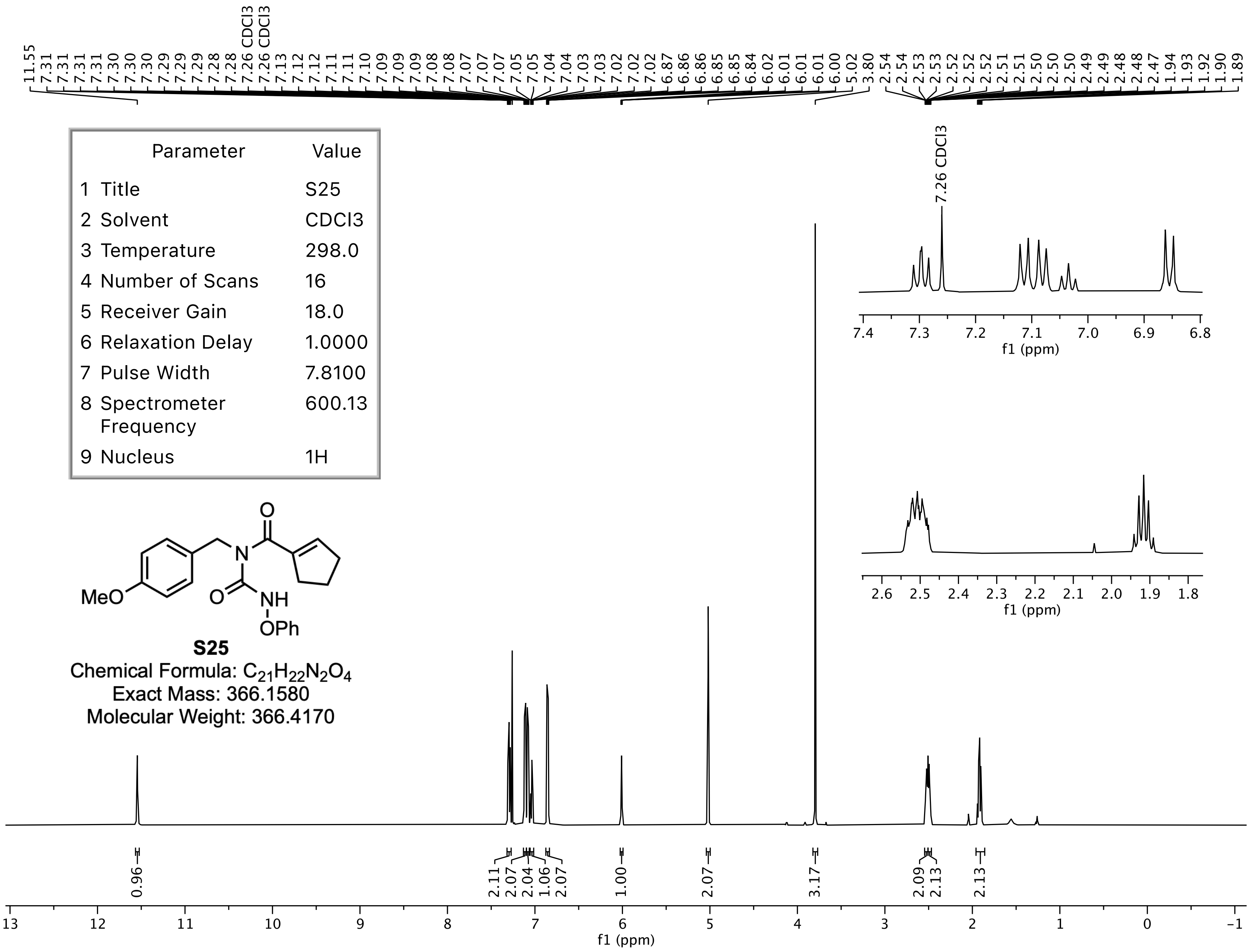


| | Parameter | Value |
|---|---|---|
| 1 | Title | S25 |
| 2 | Solvent | CDCl3 |
| 3 | Temperature | 298.0 |
| 4 | Number of Scans | 16 |
| 5 | Receiver Gain | 18.0 |
| 6 | Relaxation Delay | 1.0000 |
| 7 | Pulse Width | 7.8100 |
| 8 | Spectrometer Frequency | 600.13 |
| 9 | Nucleus | 1H |

**S25**

Chemical Formula: $C_{21}H_{22}N_2O_4$
Exact Mass: 366.1580
Molecular Weight: 366.4170

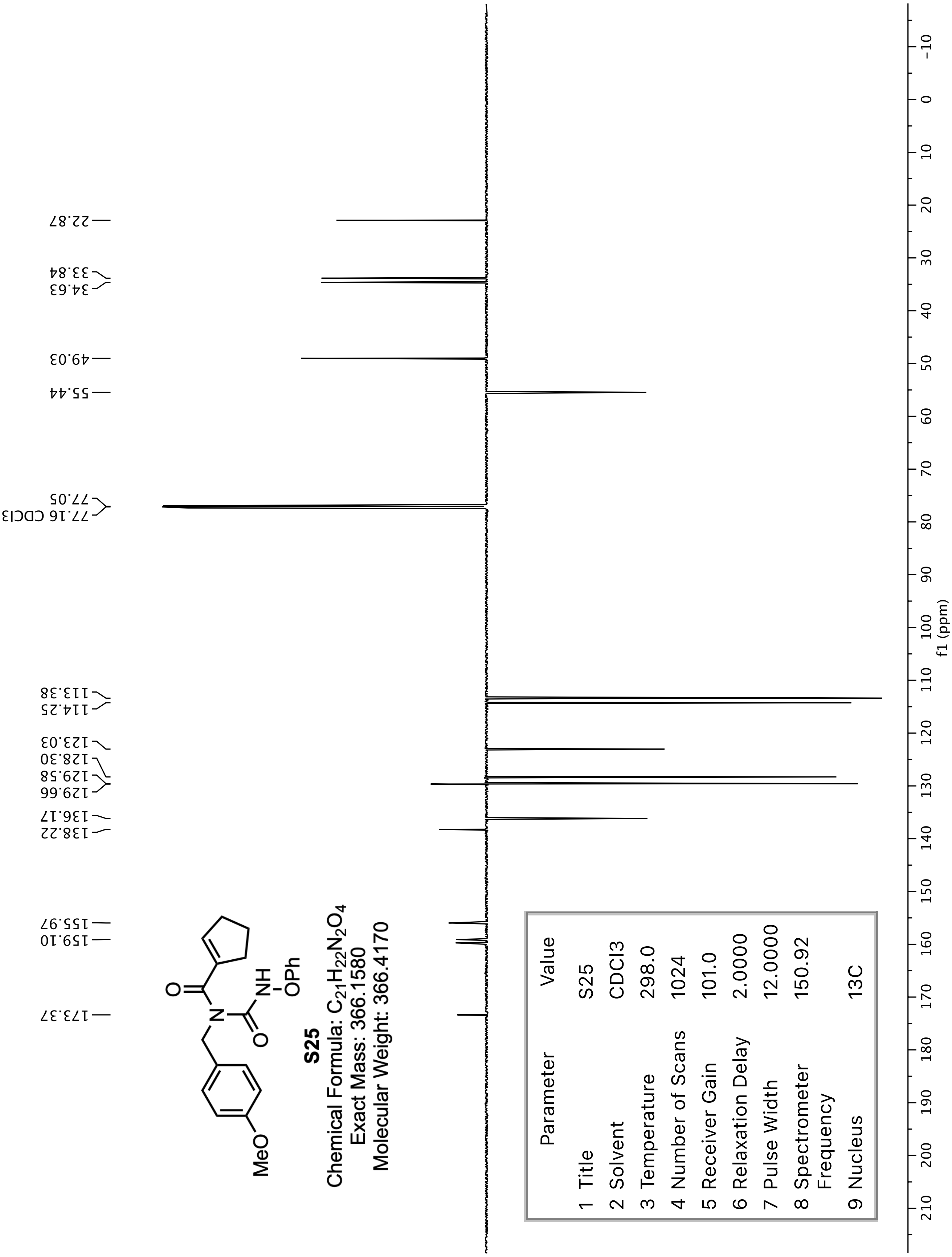

173.37
159.10
155.97
138.22
136.17
129.66
129.58
128.30
123.03
114.25
113.38
77.16 CDCl3
77.05
55.44
49.03
34.63
33.84
22.87
MeO
O
N
O
NH
OPh
S25
Chemical Formula: $C_{21}H_{22}N_2O_4$
Exact Mass: 366.1580
Molecular Weight: 366.4170
Parameter Value
1 Title S25
2 Solvent CDCl3
3 Temperature 298.0
4 Number of Scans 1024
5 Receiver Gain 101.0
6 Relaxation Delay 2.0000
7 Pulse Width 12.0000
8 Spectrometer Frequency 150.92
9 Nucleus 13C
f1 (ppm)

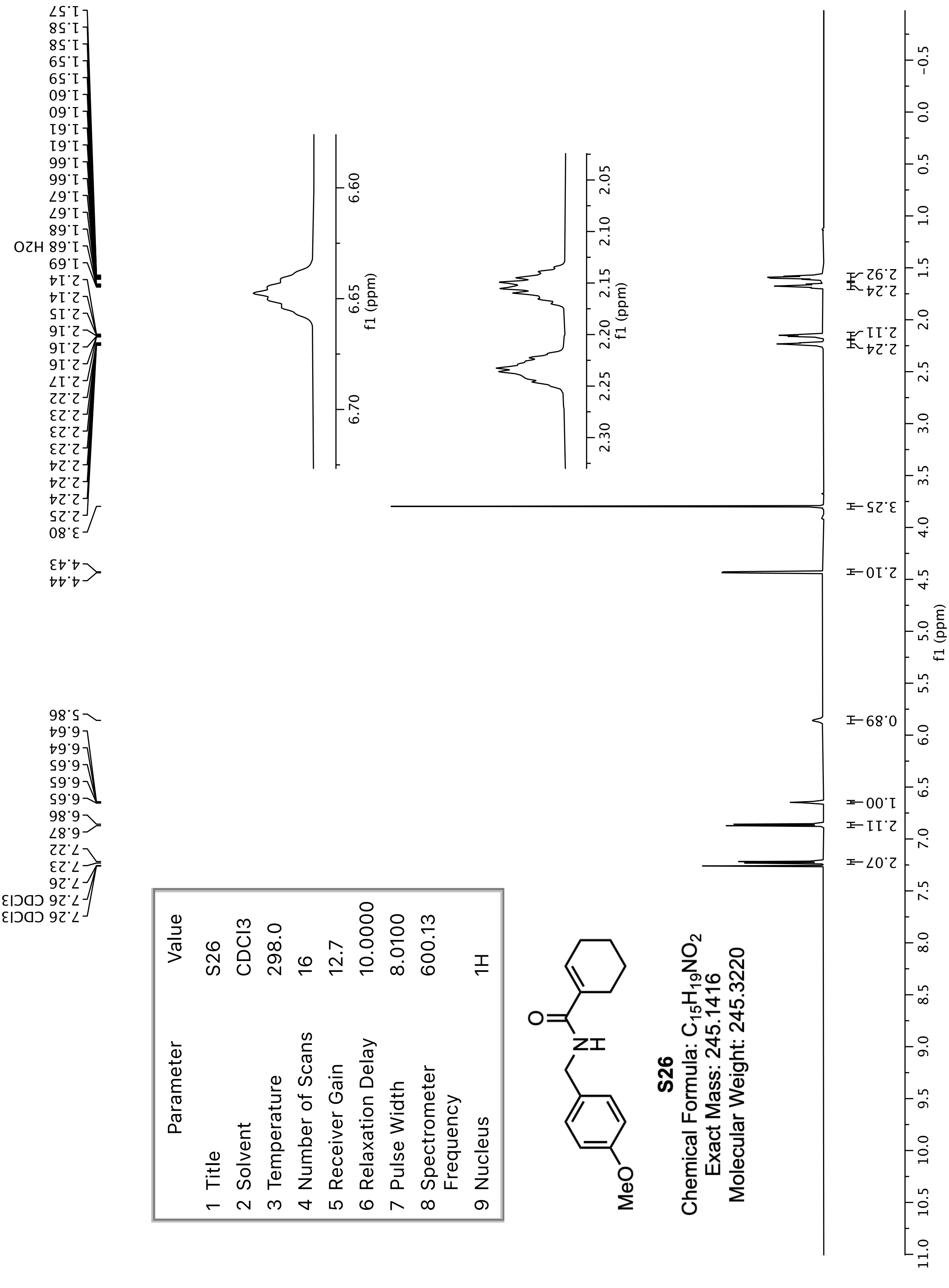


| Parameter | Value |
|---|---|
| 1 Title | S26 |
| 2 Solvent | CDCl3 |
| 3 Temperature | 298.0 |
| 4 Number of Scans | 16 |
| 5 Receiver Gain | 12.7 |
| 6 Relaxation Delay | 10.0000 |
| 7 Pulse Width | 8.0100 |
| 8 Spectrometer Frequency | 600.13 |
| 9 Nucleus | 1H |

**S26**

Chemical Formula: $C_{15}H_{19}NO_2$

Exact Mass: 245.1416

Molecular Weight: 245.3220

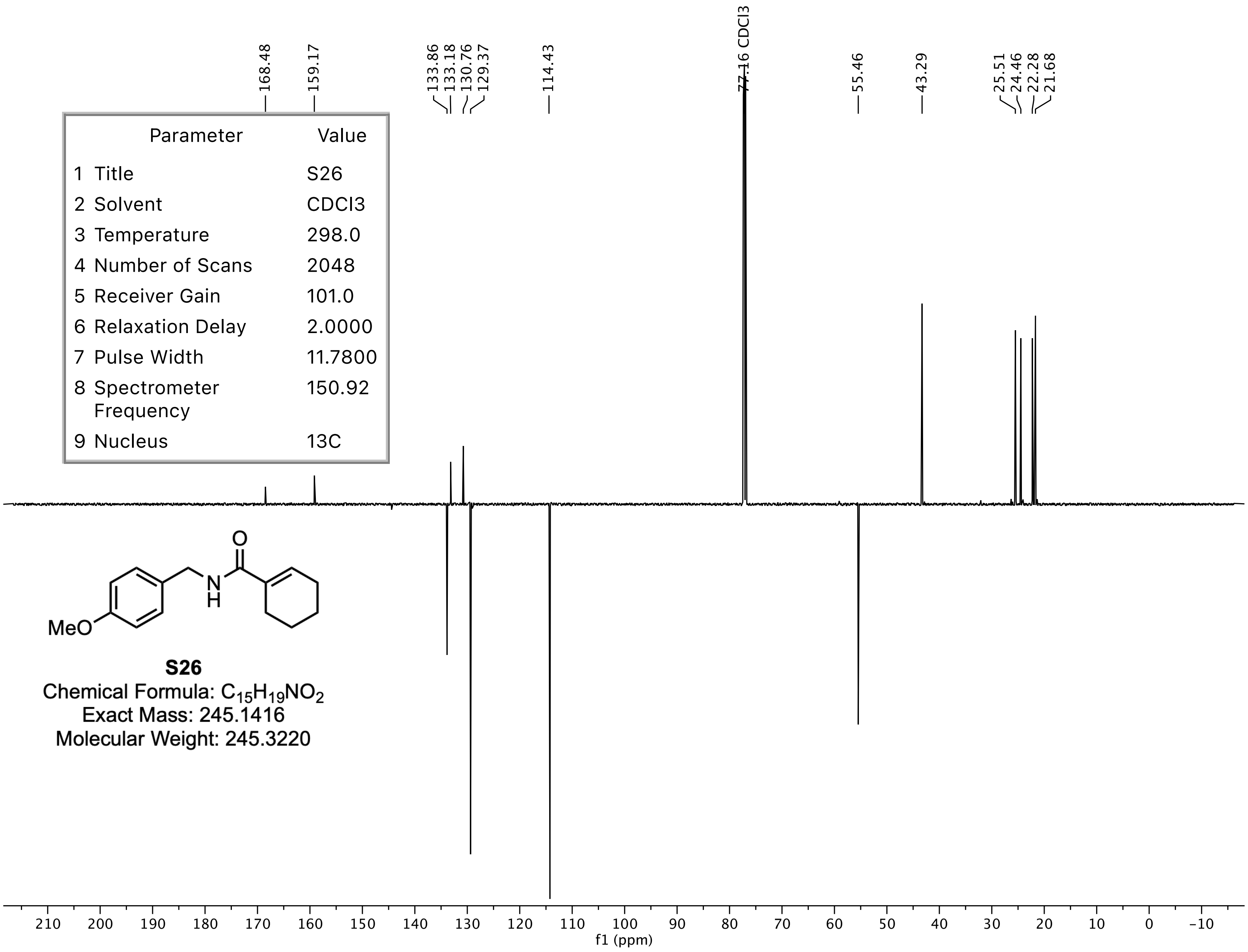


| | Parameter | Value |
|---|---|---|
| 1 | Title | S26 |
| 2 | Solvent | CDCl3 |
| 3 | Temperature | 298.0 |
| 4 | Number of Scans | 2048 |
| 5 | Receiver Gain | 101.0 |
| 6 | Relaxation Delay | 2.0000 |
| 7 | Pulse Width | 11.7800 |
| 8 | Spectrometer Frequency | 150.92 |
| 9 | Nucleus | 13C |

**S26**

Chemical Formula: $C_{15}H_{19}NO_2$

Exact Mass: 245.1416

Molecular Weight: 245.3220

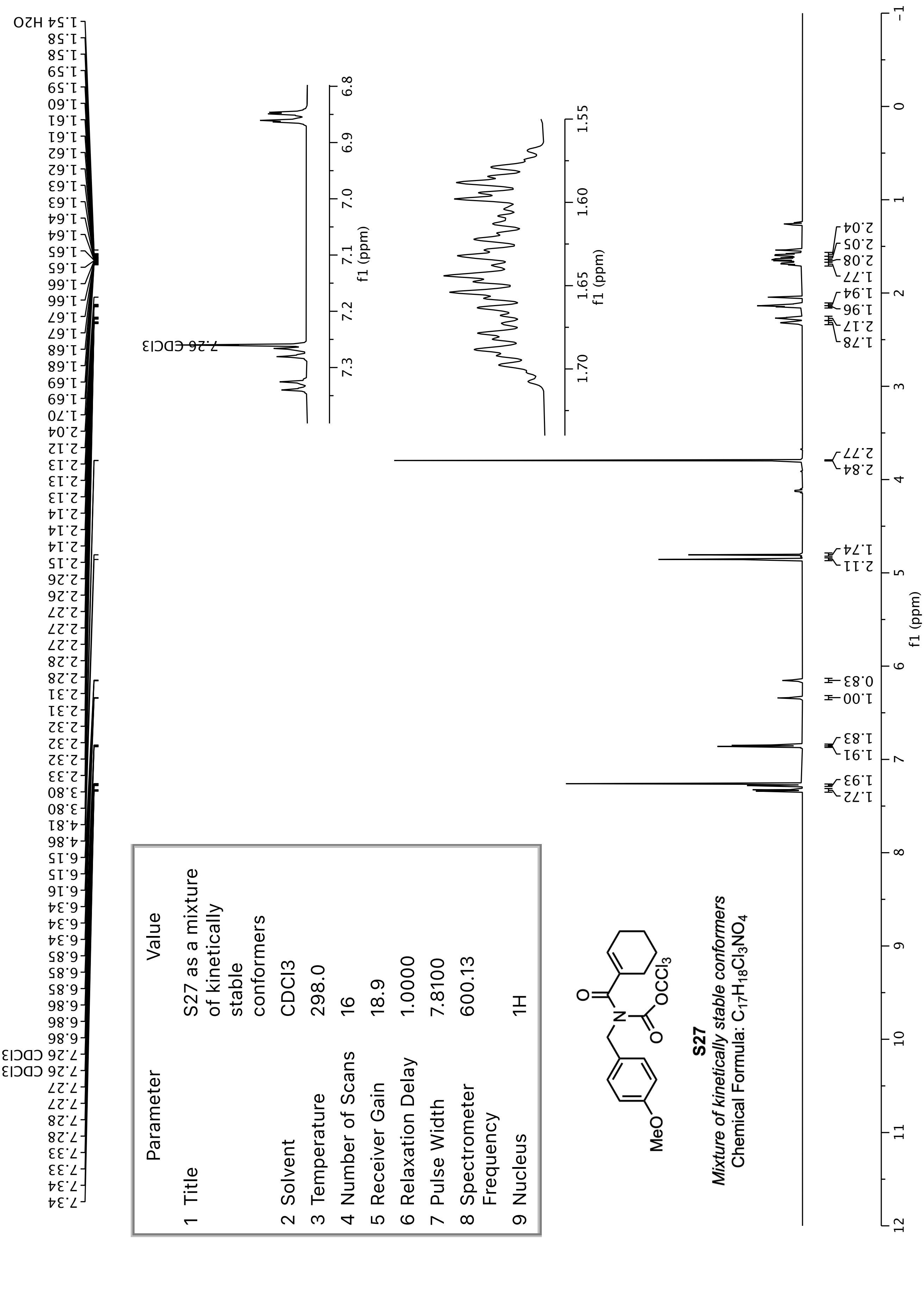


| Parameter | Value |
|---|---|
| 1 Title | S27 as a mixture of kinetically stable conformers |
| 2 Solvent | CDCl3 |
| 3 Temperature | 298.0 |
| 4 Number of Scans | 16 |
| 5 Receiver Gain | 18.9 |
| 6 Relaxation Delay | 1.0000 |
| 7 Pulse Width | 7.8100 |
| 8 Spectrometer Frequency | 600.13 |
| 9 Nucleus | 1H |

**S27**

*Mixture of kinetically stable conformers*

Chemical Formula: $C_{17}H_{18}Cl_3NO_4$

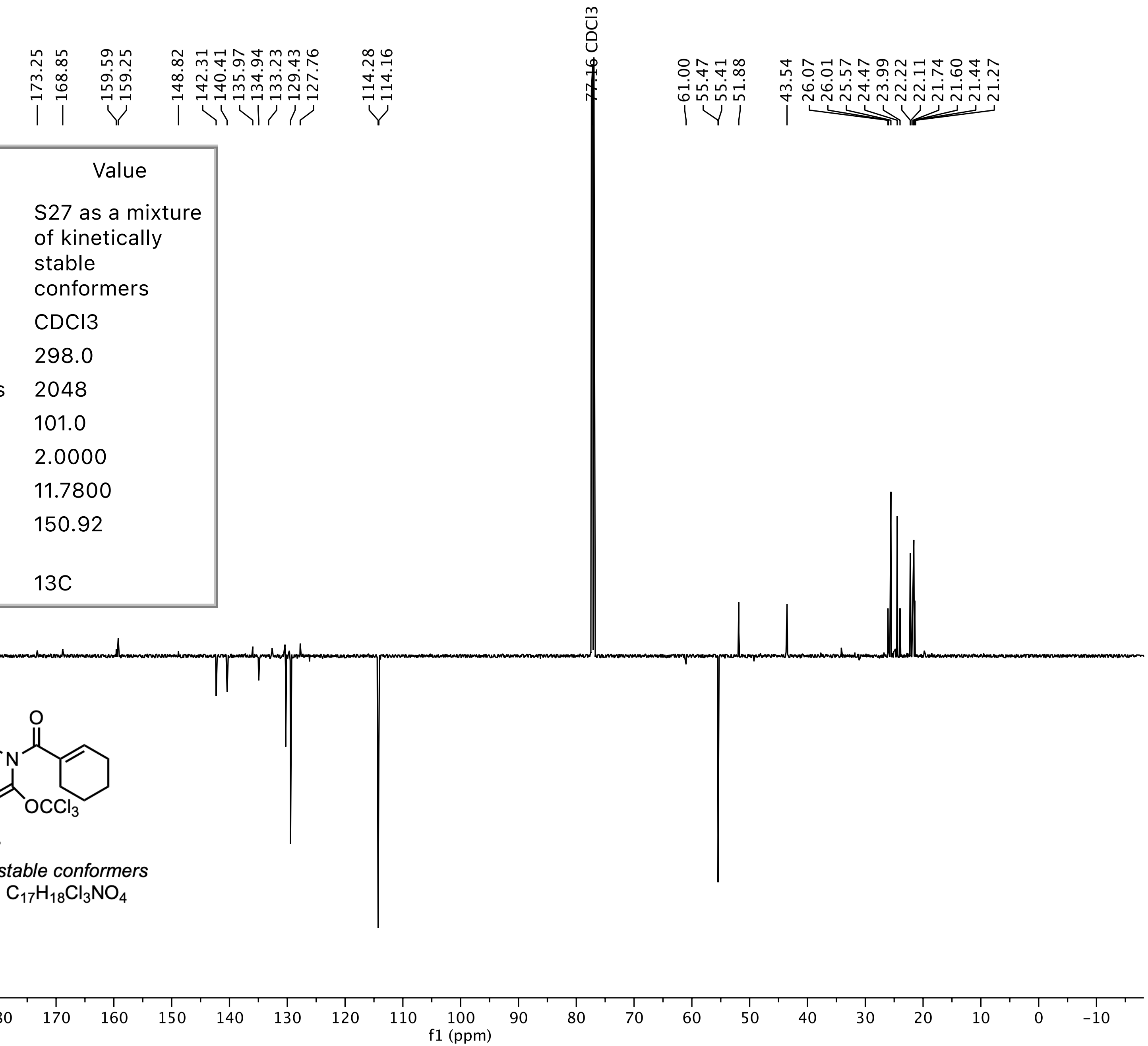


| Parameter | Value |
|---|---|
| 1 Title | S27 as a mixture of kinetically stable conformers |
| 2 Solvent | CDCl3 |
| 3 Temperature | 298.0 |
| 4 Number of Scans | 2048 |
| 5 Receiver Gain | 101.0 |
| 6 Relaxation Delay | 2.0000 |
| 7 Pulse Width | 11.7800 |
| 8 Spectrometer Frequency | 150.92 |
| 9 Nucleus | 13C |

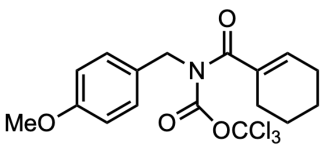


**S27**

*Mixture of kinetically stable conformers*

Chemical Formula: $C_{17}H_{18}Cl_3NO_4$

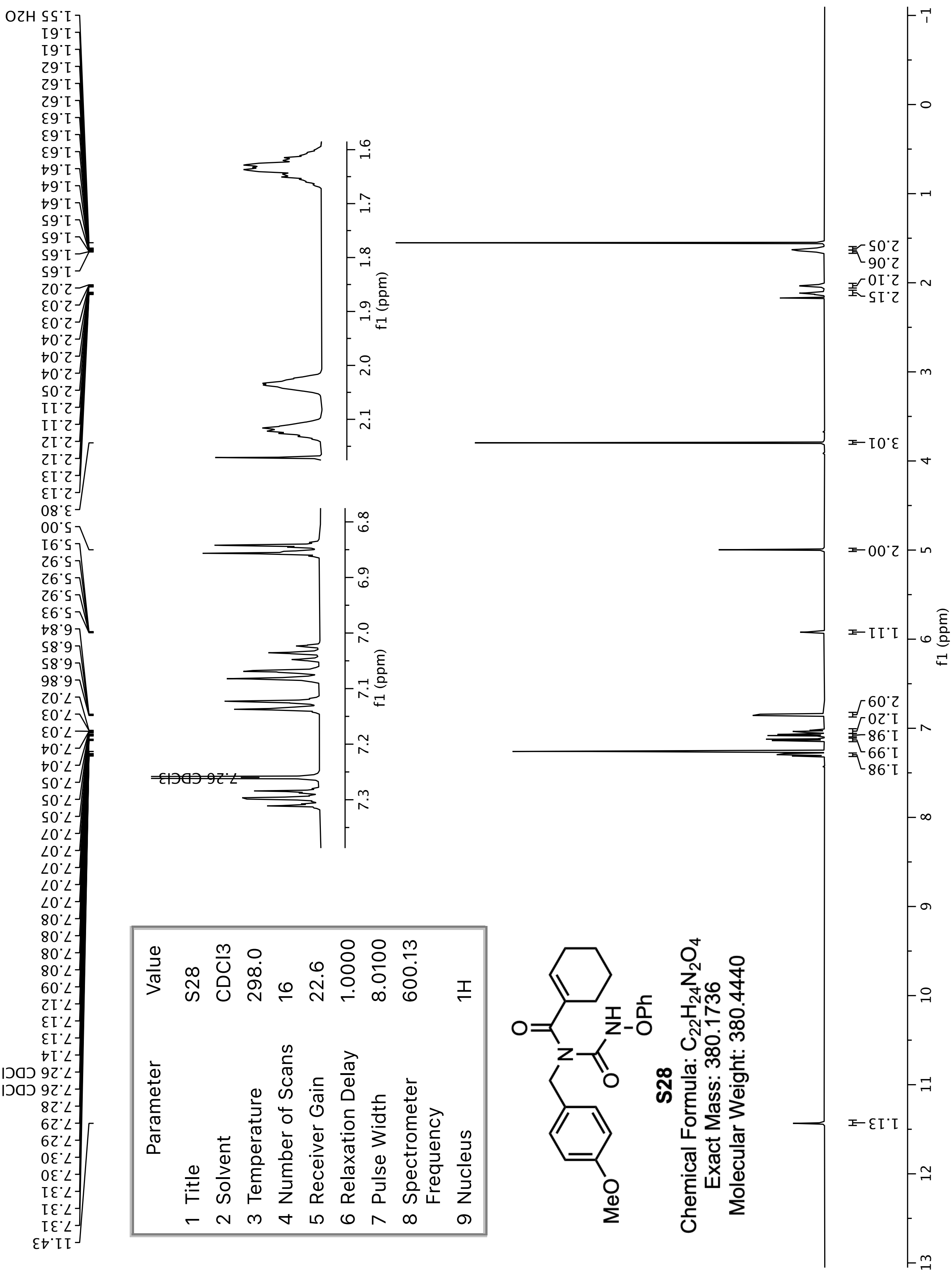


| Parameter | Value |
|---|---|
| 1 Title | S28 |
| 2 Solvent | CDCl3 |
| 3 Temperature | 298.0 |
| 4 Number of Scans | 16 |
| 5 Receiver Gain | 22.6 |
| 6 Relaxation Delay | 1.0000 |
| 7 Pulse Width | 8.0100 |
| 8 Spectrometer Frequency | 600.13 |
| 9 Nucleus | 1H |



**S28**

Chemical Formula: $C_{22}H_{24}N_2O_4$

Exact Mass: 380.1736

Molecular Weight: 380.4440

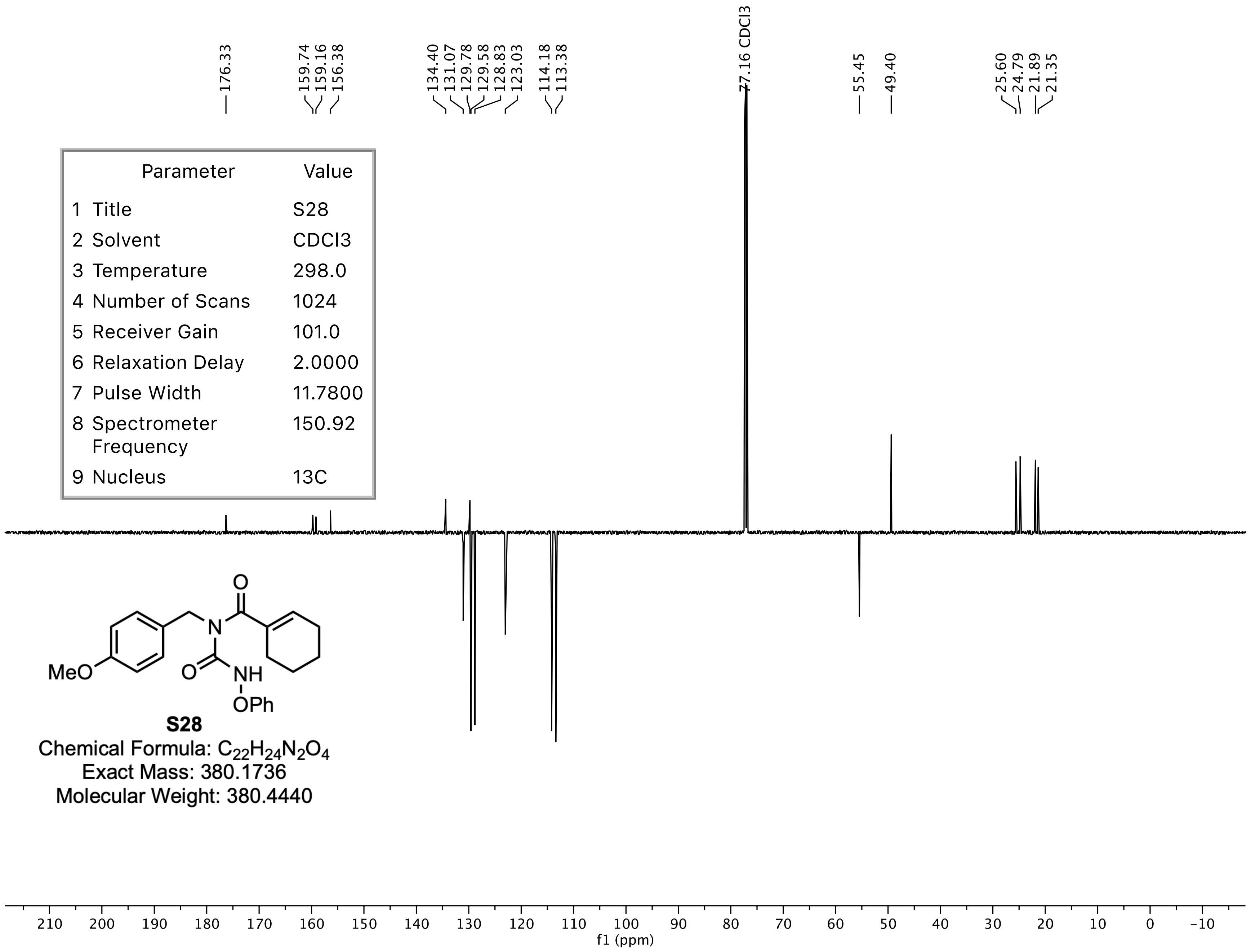


| | Parameter | Value |
|---|---|---|
| 1 | Title | S28 |
| 2 | Solvent | CDCl3 |
| 3 | Temperature | 298.0 |
| 4 | Number of Scans | 1024 |
| 5 | Receiver Gain | 101.0 |
| 6 | Relaxation Delay | 2.0000 |
| 7 | Pulse Width | 11.7800 |
| 8 | Spectrometer Frequency | 150.92 |
| 9 | Nucleus | 13C |

**S28**

Chemical Formula: $C_{22}H_{24}N_2O_4$

Exact Mass: 380.1736

Molecular Weight: 380.4440

| Parameter | Value |
|---|---|
| 1 Title | S29 |
| 2 Solvent | CDCl3 |
| 3 Temperature | 298.0 |
| 4 Number of Scans | 16 |
| 5 Receiver Gain | 11.3 |
| 6 Relaxation Delay | 10.0000 |
| 7 Pulse Width | 8.0100 |
| 8 Spectrometer Frequency | 600.13 |
| 9 Nucleus | 1H |

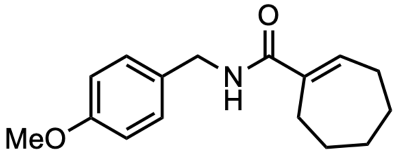


**S29**
Chemical Formula: $C_{16}H_{21}NO_2$
Exact Mass: 259.1572
Molecular Weight: 259.3490

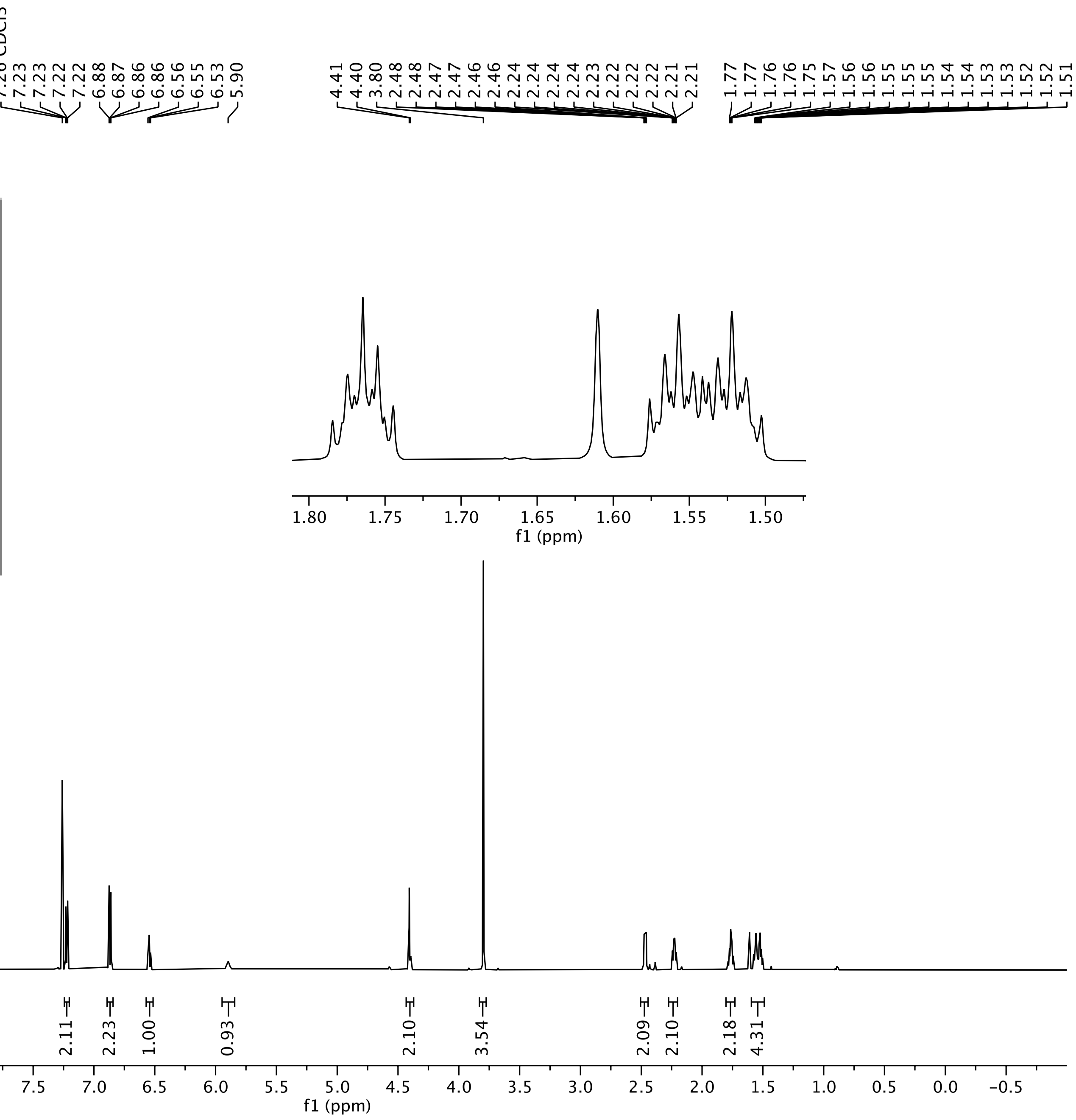

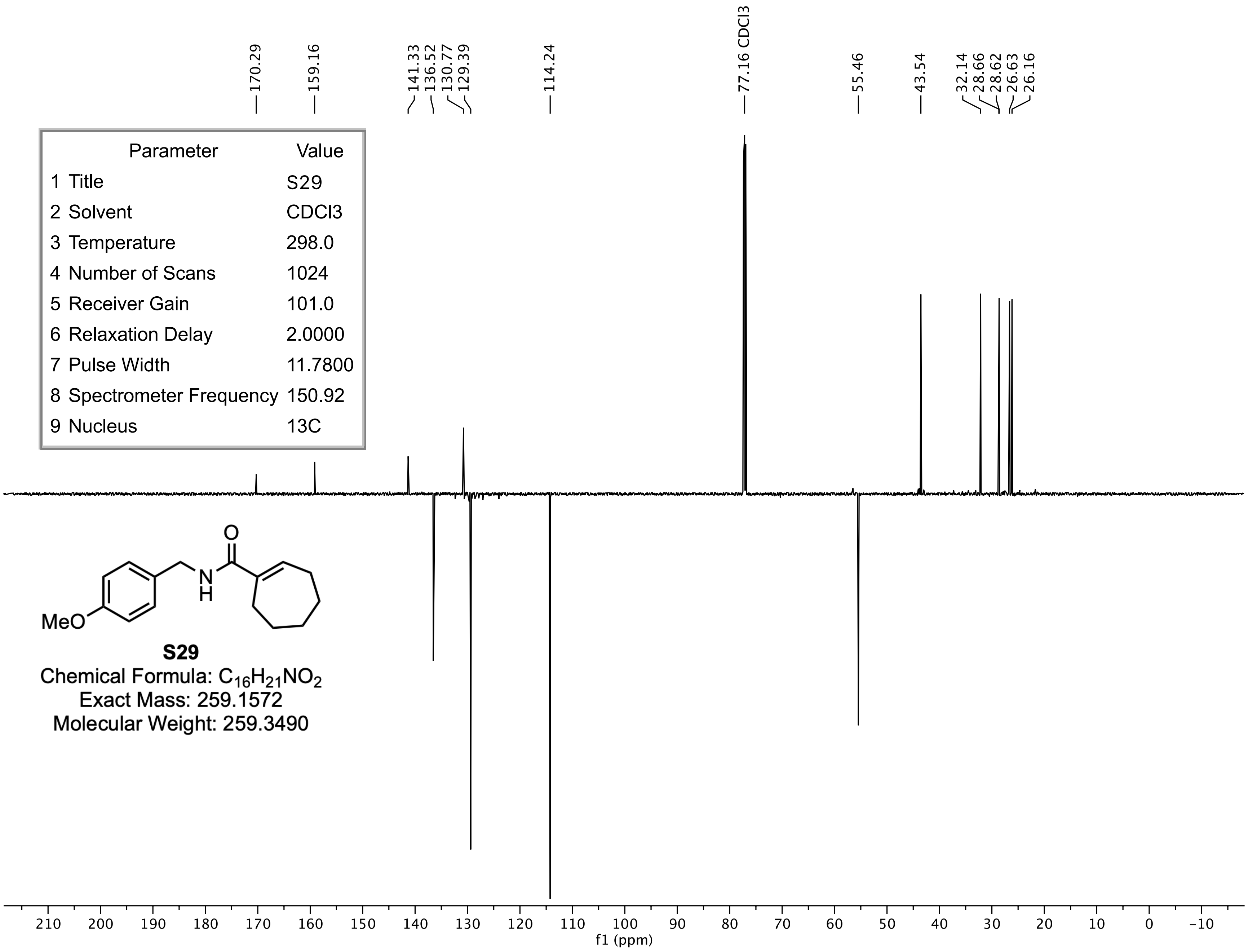


| Parameter | Value |
|---|---|
| 1 Title | S29 |
| 2 Solvent | CDCl3 |
| 3 Temperature | 298.0 |
| 4 Number of Scans | 1024 |
| 5 Receiver Gain | 101.0 |
| 6 Relaxation Delay | 2.0000 |
| 7 Pulse Width | 11.7800 |
| 8 Spectrometer Frequency | 150.92 |
| 9 Nucleus | 13C |

**S29**
Chemical Formula: $C_{16}H_{21}NO_2$
Exact Mass: 259.1572
Molecular Weight: 259.3490

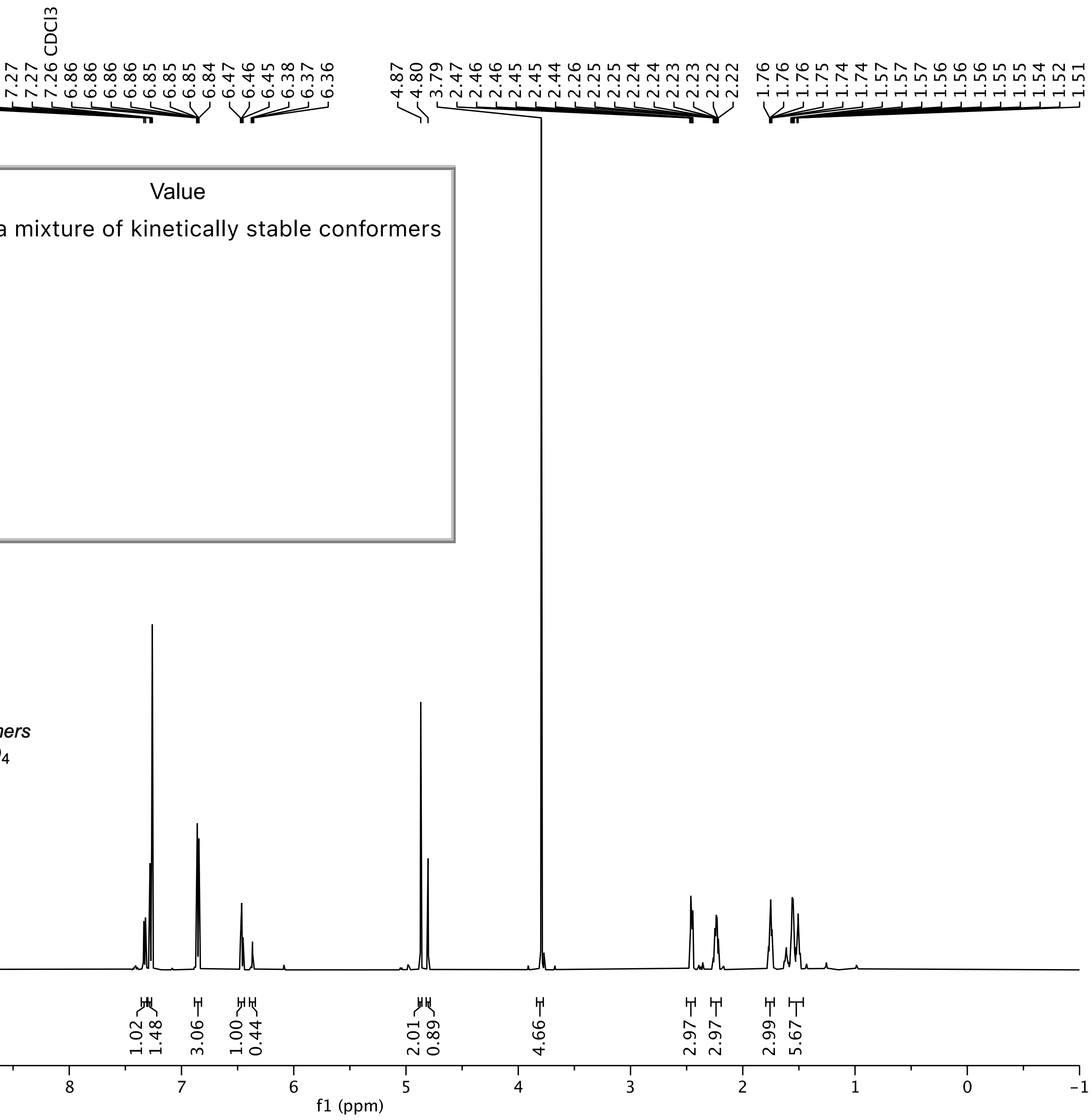


| Parameter | Value |
|---|---|
| 1 Title | S30 as a mixture of kinetically stable conformers |
| 2 Solvent | CDCl3 |
| 3 Temperature | 298.0 |
| 4 Number of Scans | 16 |
| 5 Receiver Gain | 11.3 |
| 6 Relaxation Delay | 10.0000 |
| 7 Pulse Width | 8.0100 |
| 8 Spectrometer Frequency | 600.13 |
| 9 Nucleus | 1H |

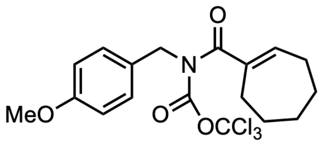


**S30**

*Mixture of kinetically stable conformers*

Chemical Formula: $C_{18}H_{20}Cl_3NO_4$

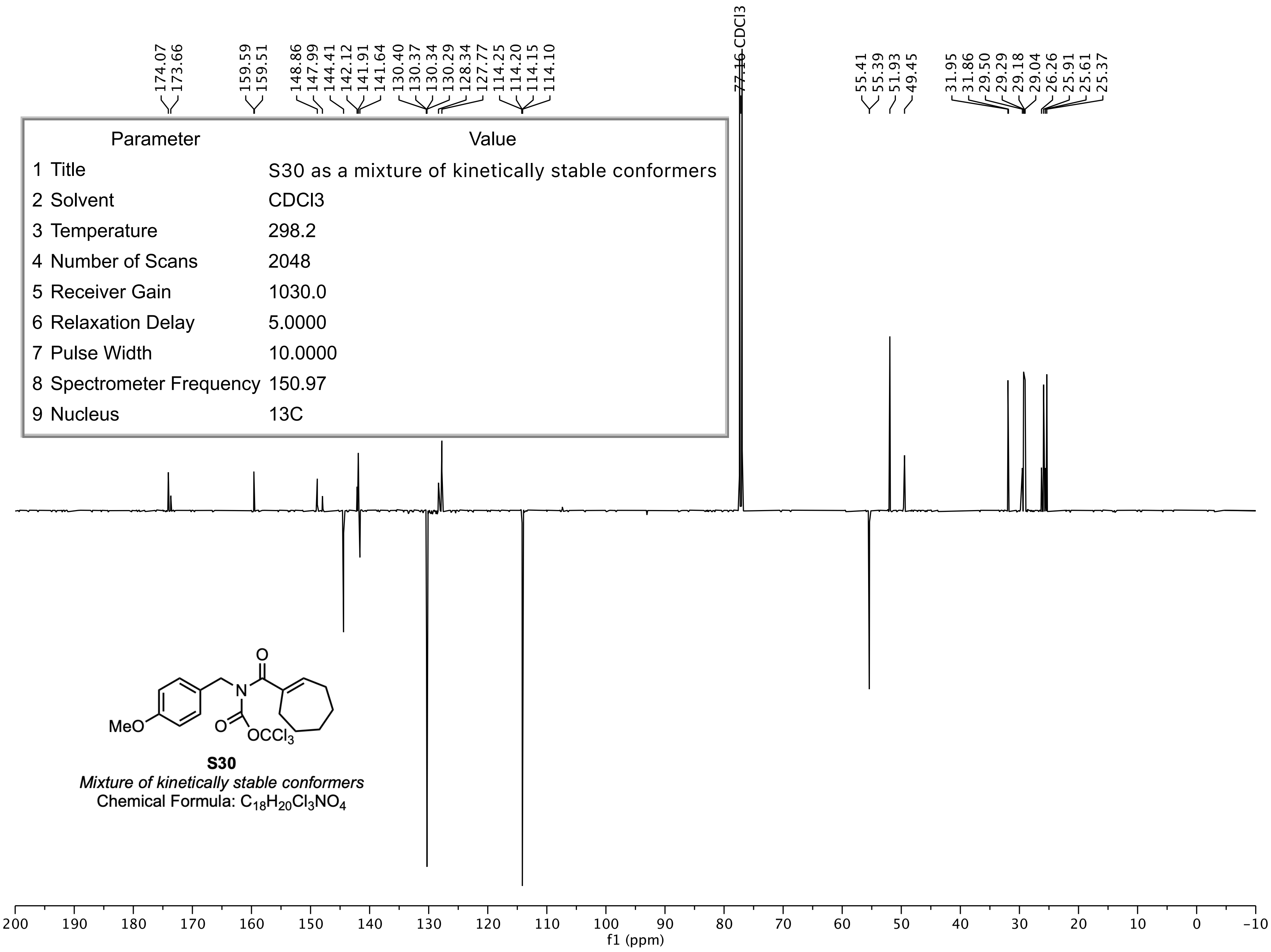


| Parameter | Value |
|---|---|
| 1 Title | S30 as a mixture of kinetically stable conformers |
| 2 Solvent | CDCl3 |
| 3 Temperature | 298.2 |
| 4 Number of Scans | 2048 |
| 5 Receiver Gain | 1030.0 |
| 6 Relaxation Delay | 5.0000 |
| 7 Pulse Width | 10.0000 |
| 8 Spectrometer Frequency | 150.97 |
| 9 Nucleus | 13C |



**S30**

*Mixture of kinetically stable conformers*

Chemical Formula: $C_{18}H_{20}Cl_3NO_4$

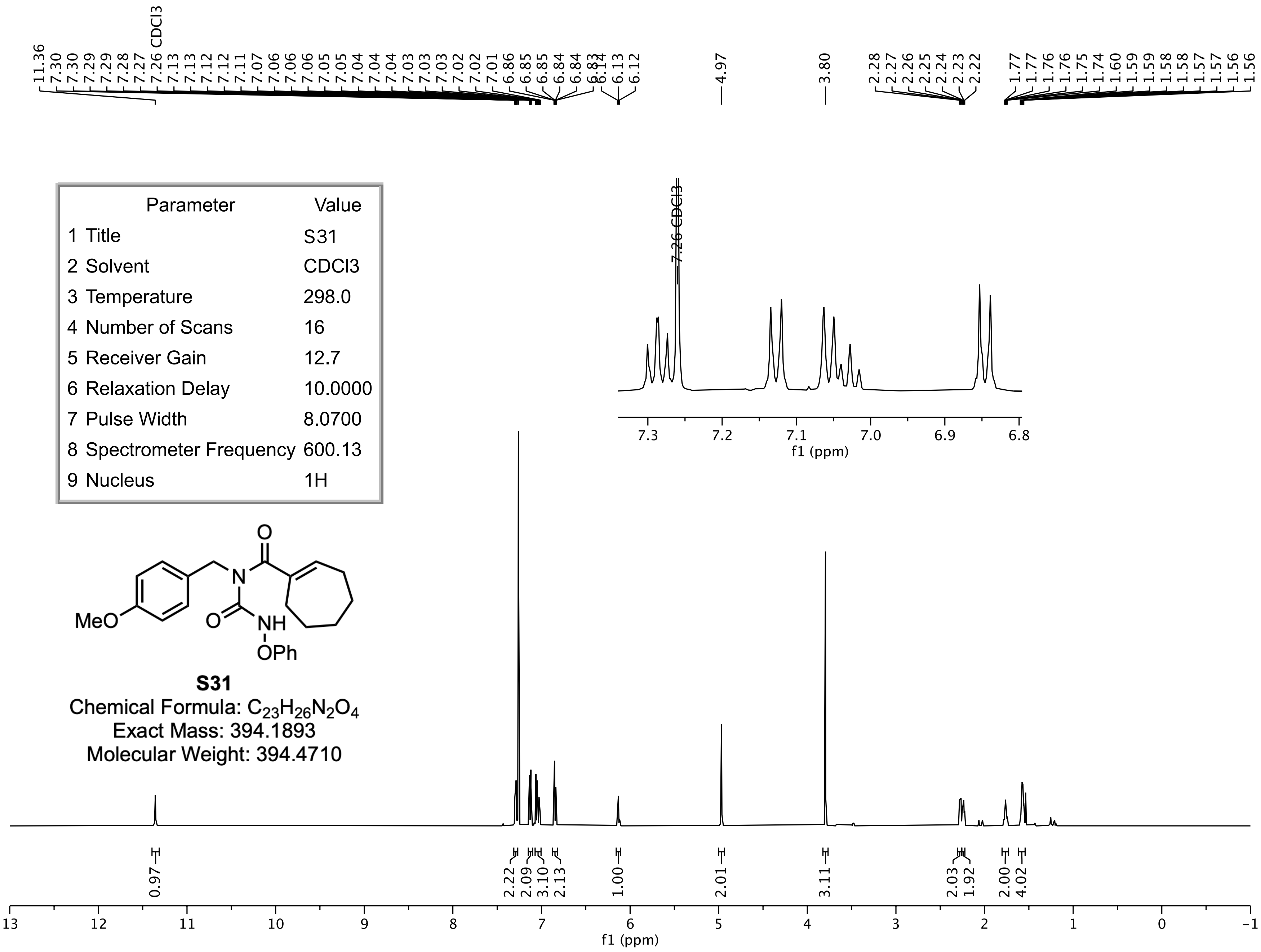


| Parameter | Value |
|---|---|
| 1 Title | S31 |
| 2 Solvent | CDCl3 |
| 3 Temperature | 298.0 |
| 4 Number of Scans | 16 |
| 5 Receiver Gain | 12.7 |
| 6 Relaxation Delay | 10.0000 |
| 7 Pulse Width | 8.0700 |
| 8 Spectrometer Frequency | 600.13 |
| 9 Nucleus | 1H |

**S31**
Chemical Formula: $C_{23}H_{26}N_2O_4$
Exact Mass: 394.1893
Molecular Weight: 394.4710

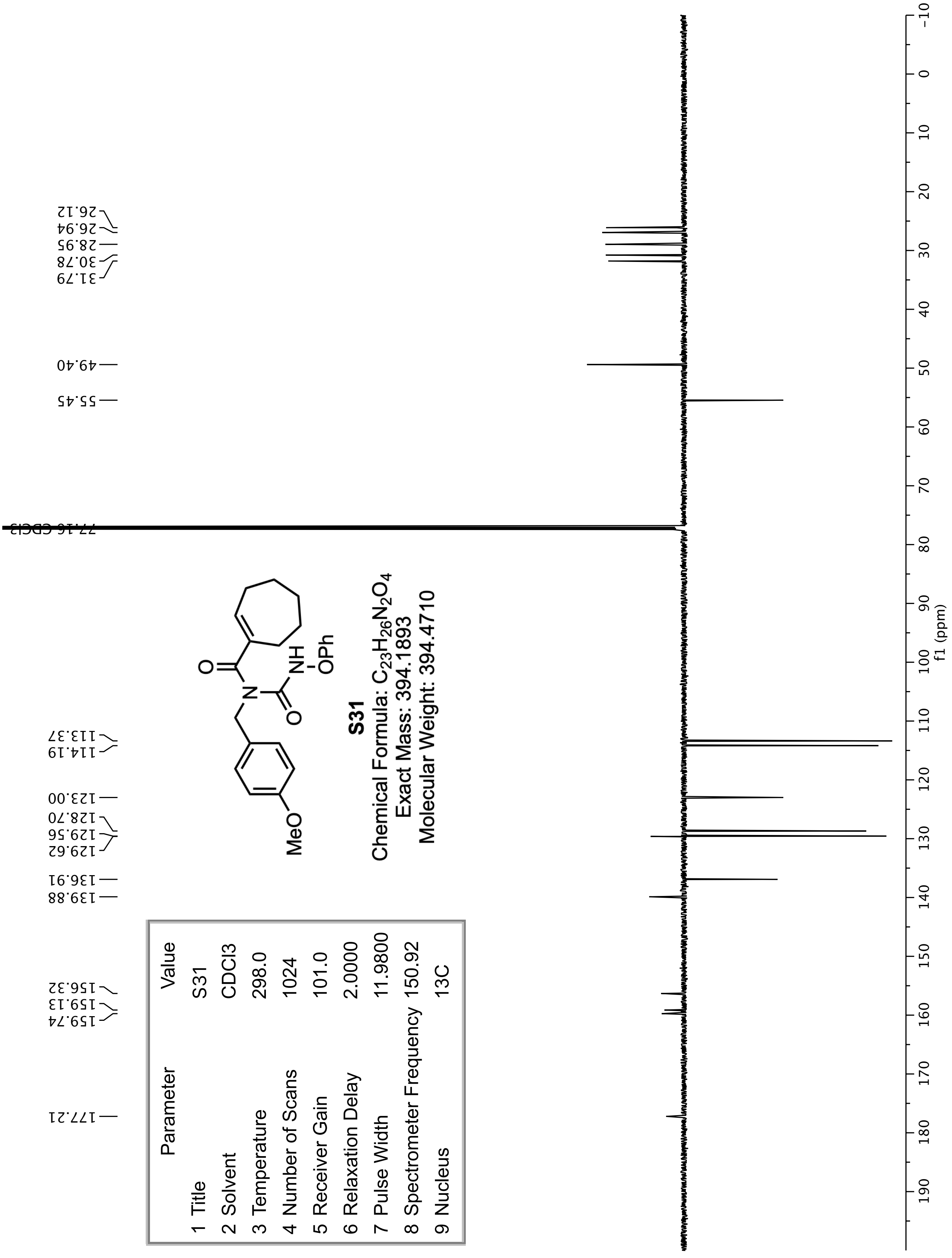


**S31**

Chemical Formula: $C_{23}H_{26}N_2O_4$

Exact Mass: 394.1893

Molecular Weight: 394.4710

| | Parameter | Value |
|---|---|---|
| 1 | Title | S31 |
| 2 | Solvent | CDCl3 |
| 3 | Temperature | 298.0 |
| 4 | Number of Scans | 1024 |
| 5 | Receiver Gain | 101.0 |
| 6 | Relaxation Delay | 2.0000 |
| 7 | Pulse Width | 11.9800 |
| 8 | Spectrometer Frequency | 150.92 |
| 9 | Nucleus | 13C |

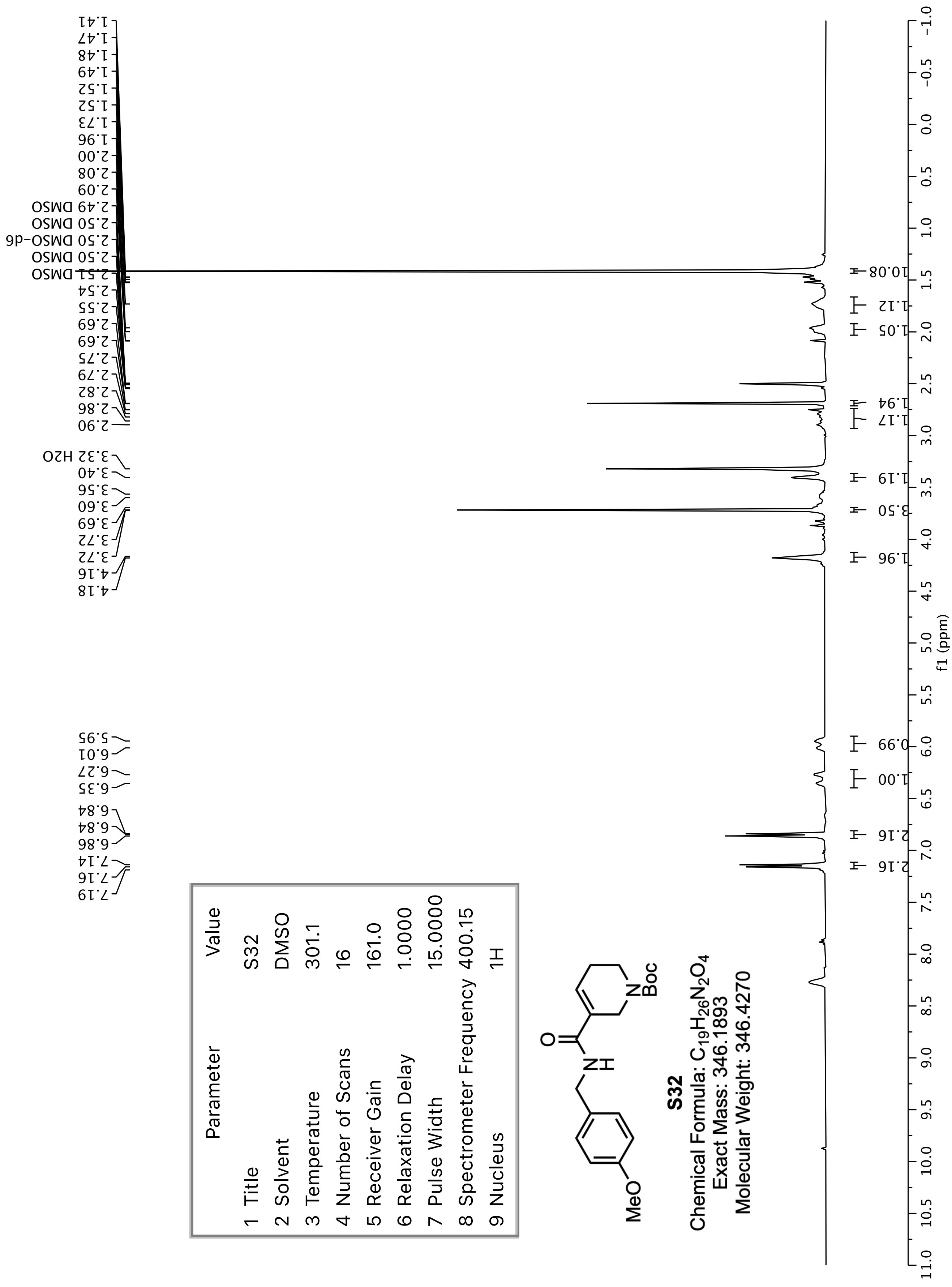


| Parameter | Value |
|---|---|
| 1 Title | S32 |
| 2 Solvent | DMSO |
| 3 Temperature | 301.1 |
| 4 Number of Scans | 16 |
| 5 Receiver Gain | 161.0 |
| 6 Relaxation Delay | 1.0000 |
| 7 Pulse Width | 15.0000 |
| 8 Spectrometer Frequency | 400.15 |
| 9 Nucleus | 1H |

**S32**
Chemical Formula: $C_{19}H_{26}N_2O_4$
Exact Mass: 346.1893
Molecular Weight: 346.4270

| Parameter | Value |
|---|---|
| 1 Title | S32 |
| 2 Solvent | DMSO |
| 3 Temperature | 333.2 |
| 4 Number of Scans | 16 |
| 5 Receiver Gain | 101.0 |
| 6 Relaxation Delay | 1.0000 |
| 7 Pulse Width | 14.8000 |
| 8 Spectrometer Frequency | 400.15 |
| 9 Nucleus | 1H |

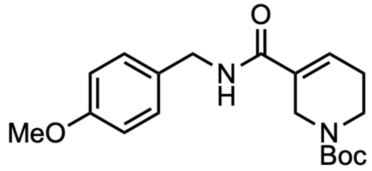


**S32**
Chemical Formula: $C_{19}H_{26}N_2O_4$
Exact Mass: 346.1893
Molecular Weight: 346.4270

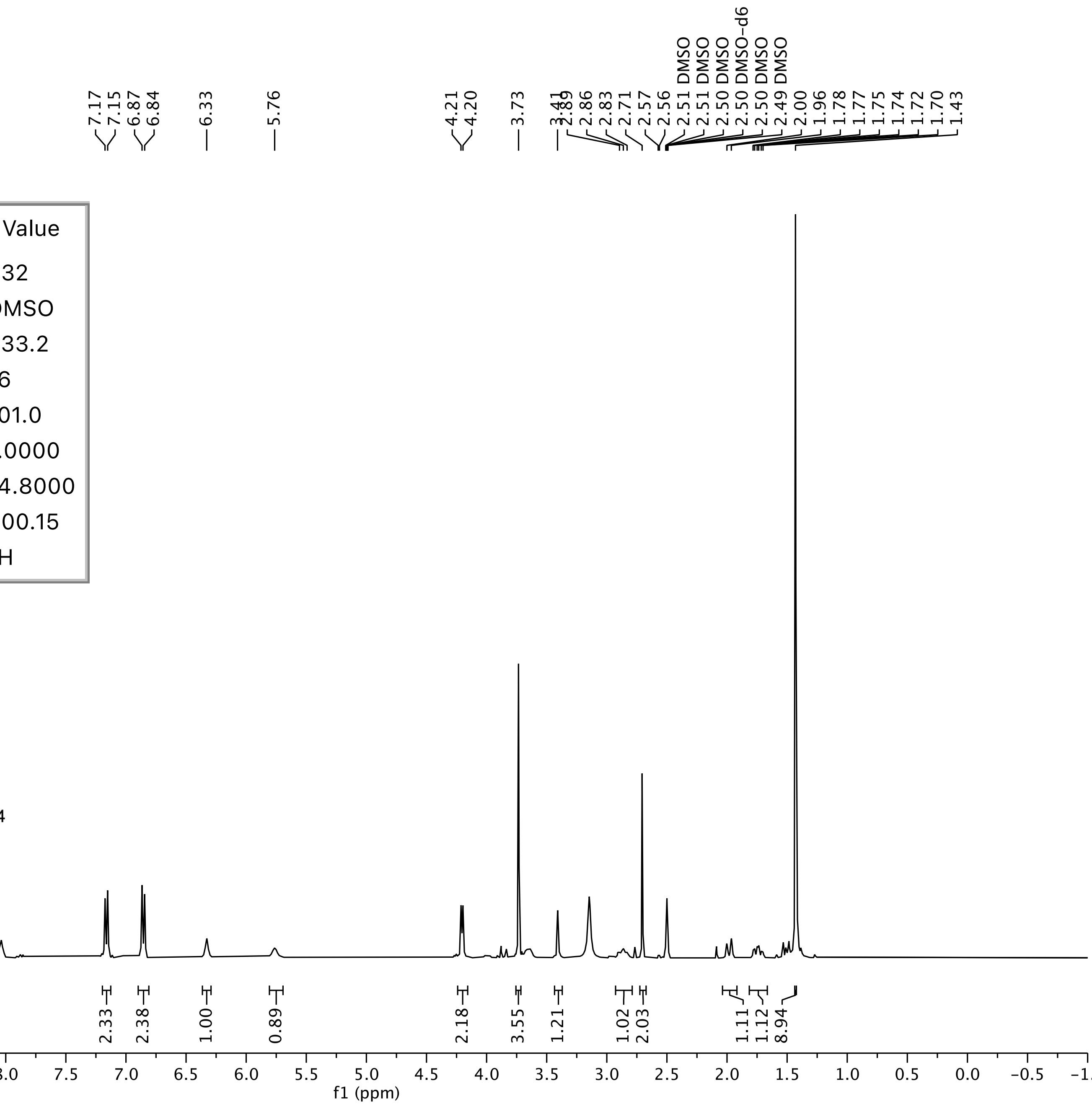

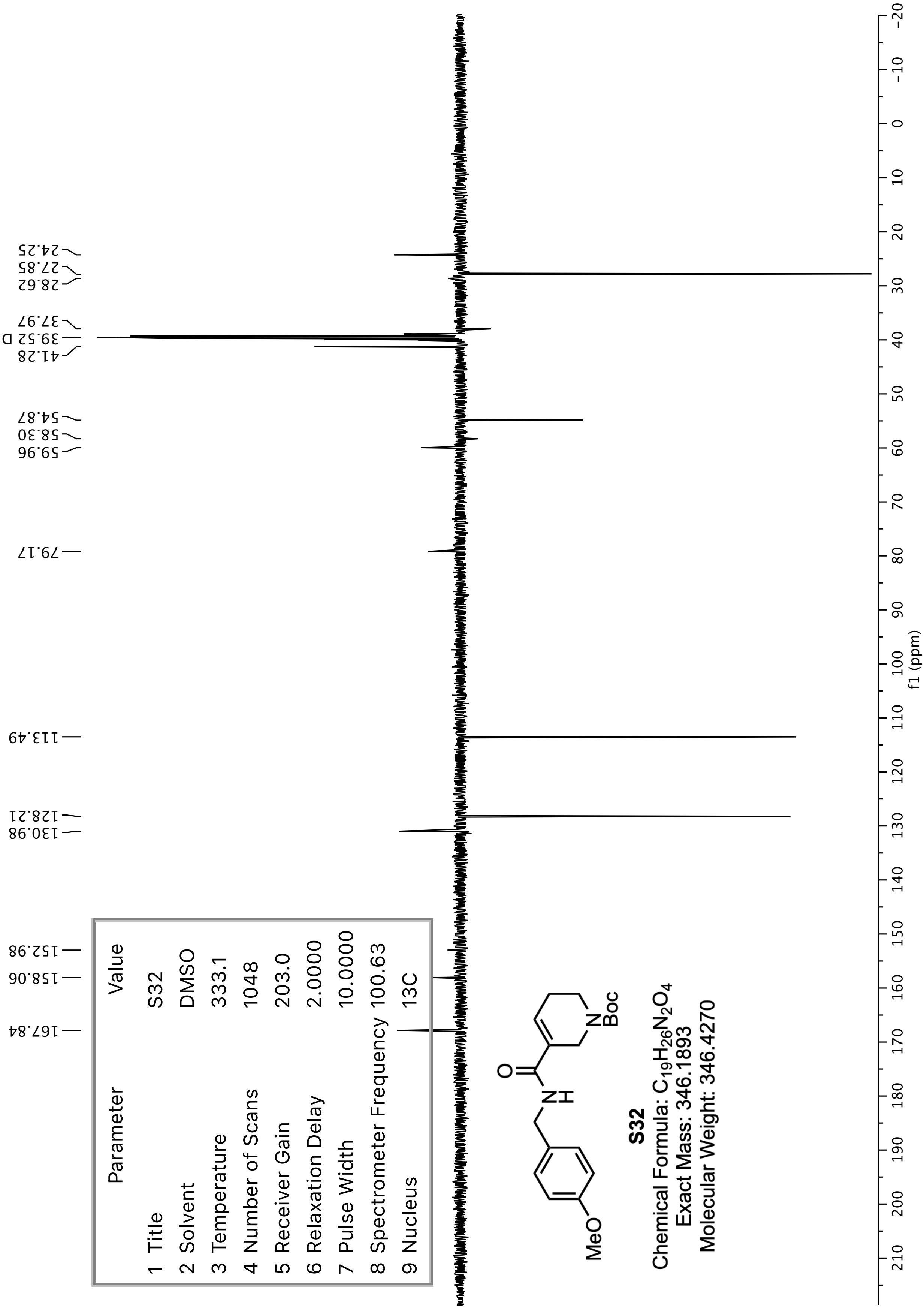


| | Parameter | Value |
|---|---|---|
| 1 | Title | S32 |
| 2 | Solvent | DMSO |
| 3 | Temperature | 333.1 |
| 4 | Number of Scans | 1048 |
| 5 | Receiver Gain | 203.0 |
| 6 | Relaxation Delay | 2.0000 |
| 7 | Pulse Width | 10.0000 |
| 8 | Spectrometer Frequency | 100.63 |
| 9 | Nucleus | 13C |

**S32**

Chemical Formula: $C_{19}H_{26}N_2O_4$

Exact Mass: 346.1893

Molecular Weight: 346.4270

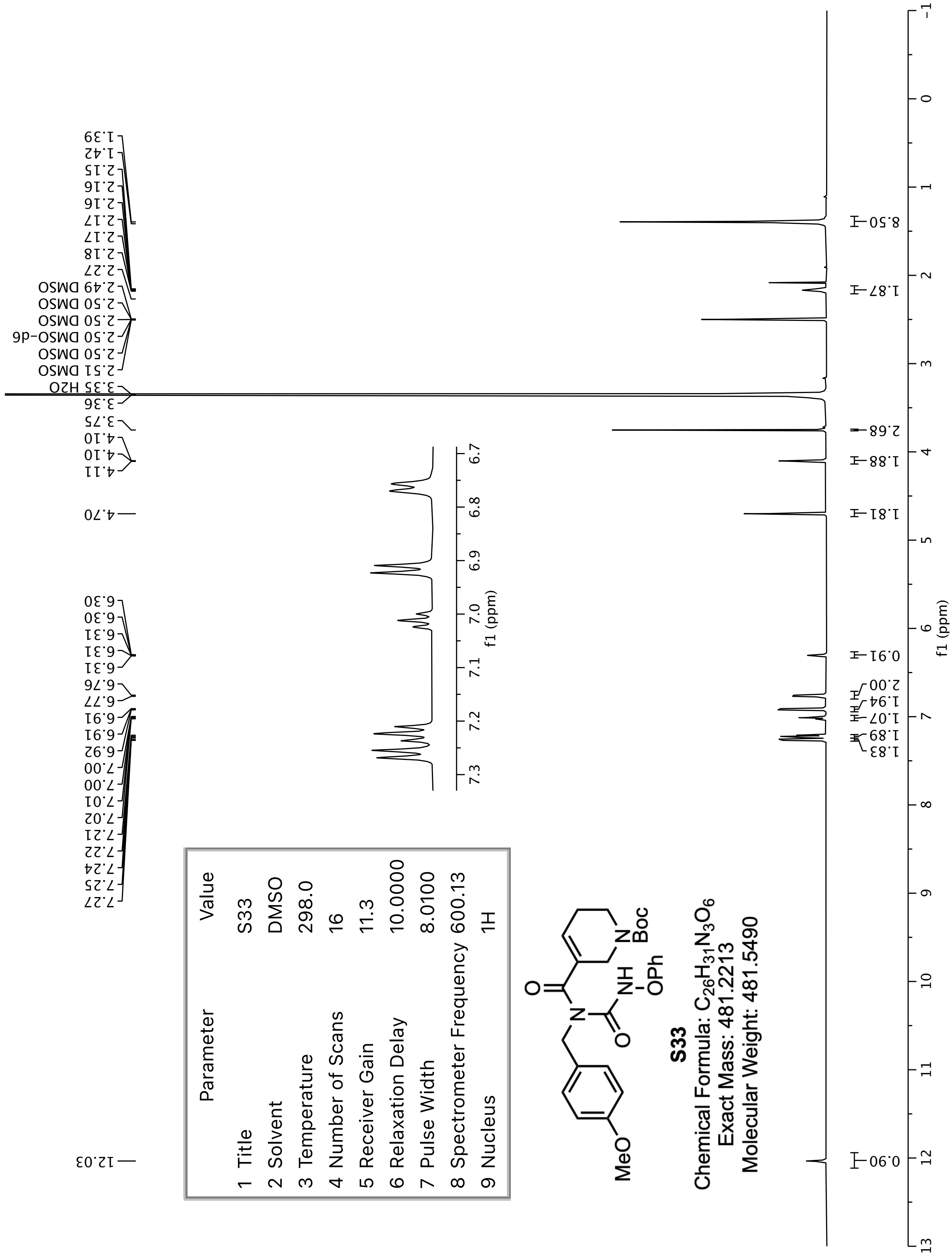


| | Parameter | Value |
|---|---|---|
| 1 | Title | S33 |
| 2 | Solvent | DMSO |
| 3 | Temperature | 298.0 |
| 4 | Number of Scans | 16 |
| 5 | Receiver Gain | 11.3 |
| 6 | Relaxation Delay | 10.0000 |
| 7 | Pulse Width | 8.0100 |
| 8 | Spectrometer Frequency | 600.13 |
| 9 | Nucleus | 1H |

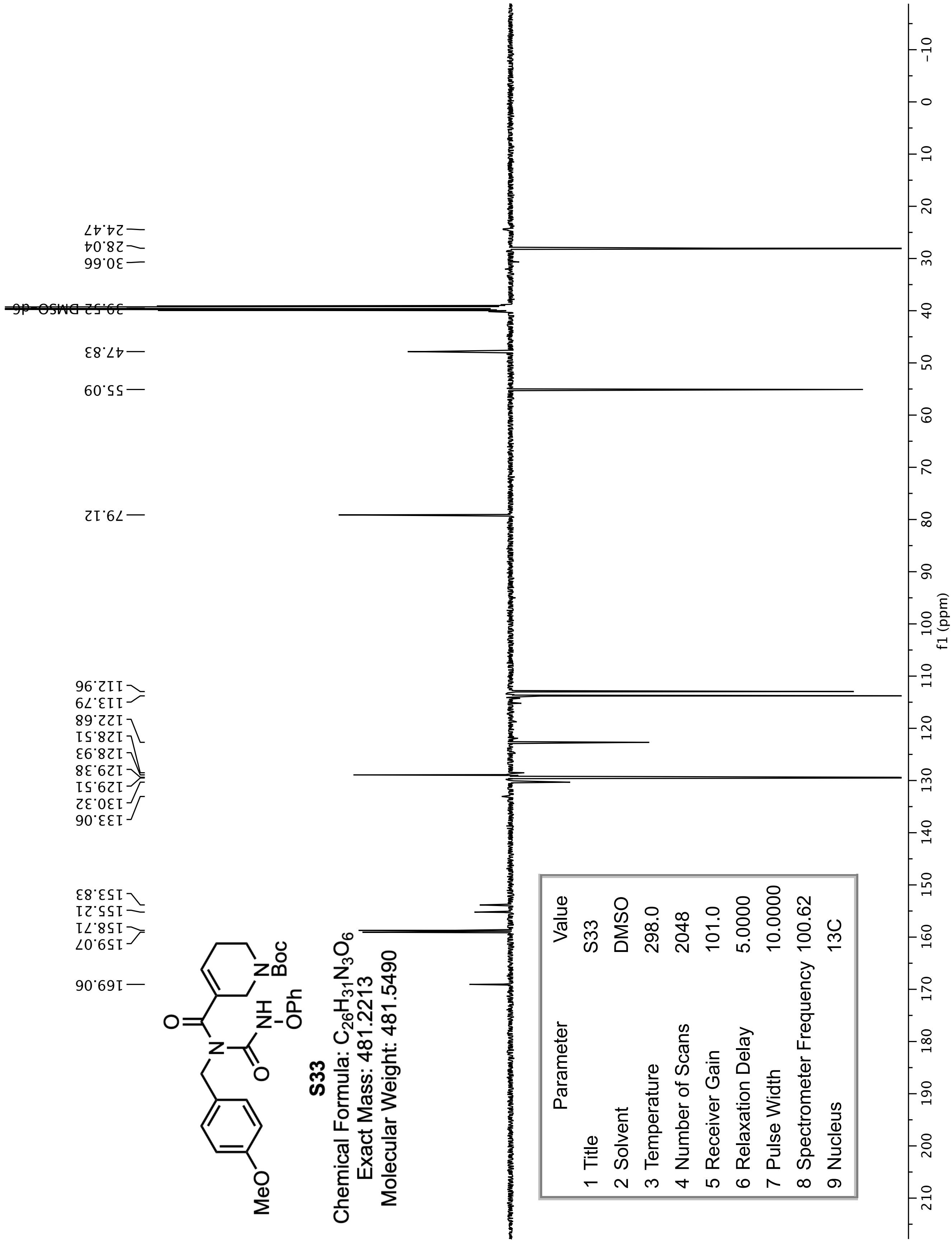


**S33**
Chemical Formula: $C_{26}H_{31}N_3O_6$
Exact Mass: 481.2213
Molecular Weight: 481.5490

| | Parameter | Value |
|---|---|---|
| 1 | Title | S33 |
| 2 | Solvent | DMSO |
| 3 | Temperature | 298.0 |
| 4 | Number of Scans | 2048 |
| 5 | Receiver Gain | 101.0 |
| 6 | Relaxation Delay | 5.0000 |
| 7 | Pulse Width | 10.0000 |
| 8 | Spectrometer Frequency | 100.62 |
| 9 | Nucleus | 13C |

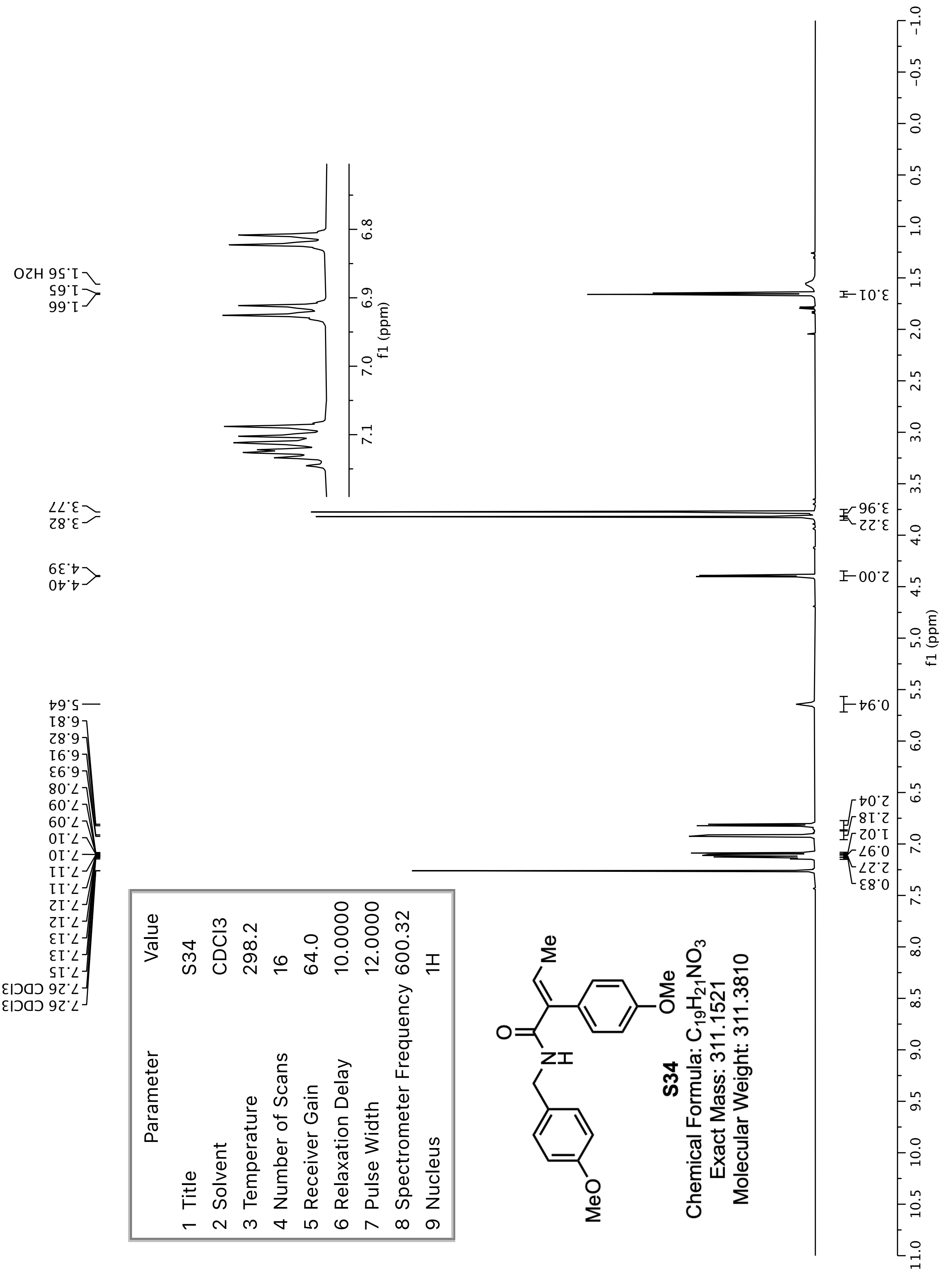


| | Parameter | Value |
|---|---|---|
| 1 | Title | S34 |
| 2 | Solvent | CDCl3 |
| 3 | Temperature | 298.2 |
| 4 | Number of Scans | 16 |
| 5 | Receiver Gain | 64.0 |
| 6 | Relaxation Delay | 10.0000 |
| 7 | Pulse Width | 12.0000 |
| 8 | Spectrometer Frequency | 600.32 |
| 9 | Nucleus | 1H |

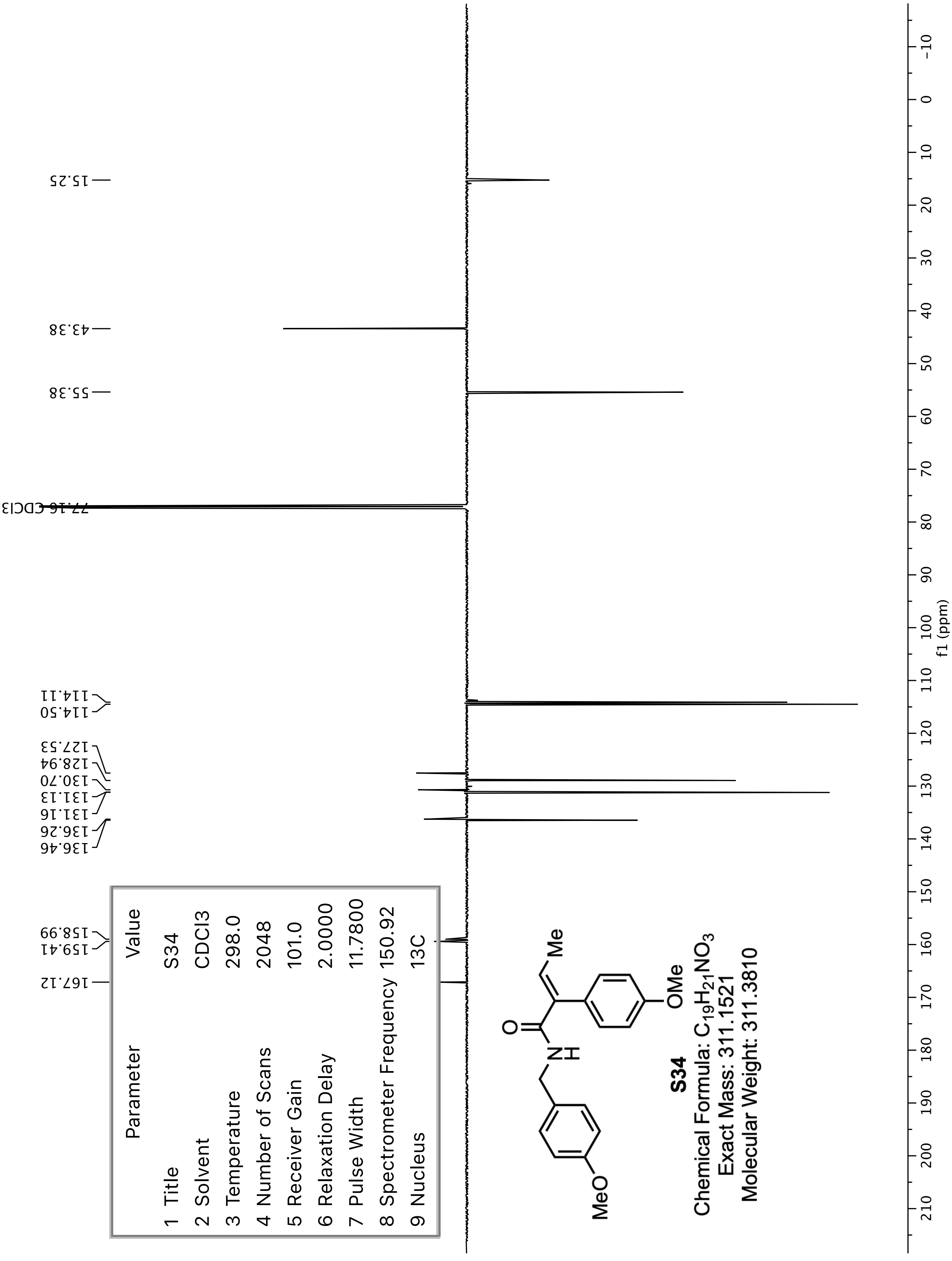
15.25
43.38
55.38
77.16 CDCl3
114.11
114.50
127.53
128.94
130.70
131.13
131.16
136.26
136.46
158.99
159.41
167.12
Parameter Value
1 Title S34
2 Solvent CDCl3
3 Temperature 298.0
4 Number of Scans 2048
5 Receiver Gain 101.0
6 Relaxation Delay 2.0000
7 Pulse Width 11.7800
8 Spectrometer Frequency 150.92
9 Nucleus 13C
Me
O
N
H
MeO
OMe
S34
Chemical Formula: $C_{19}H_{21}NO_3$
Exact Mass: 311.1521
Molecular Weight: 311.3810
f1 (ppm)

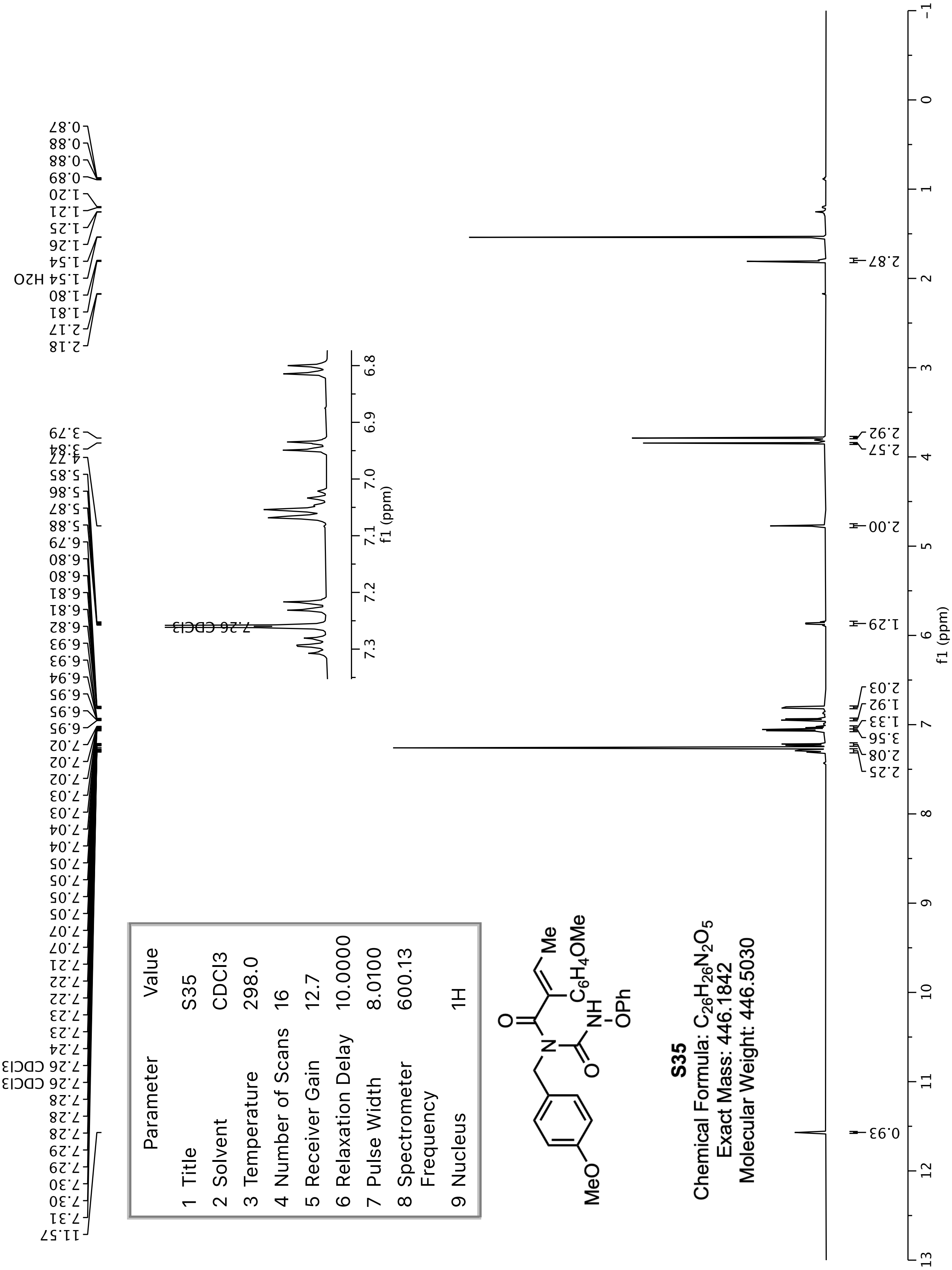


| Parameter | Value |
|---|---|
| 1 Title | S35 |
| 2 Solvent | CDCl3 |
| 3 Temperature | 298.0 |
| 4 Number of Scans | 16 |
| 5 Receiver Gain | 12.7 |
| 6 Relaxation Delay | 10.0000 |
| 7 Pulse Width | 8.0100 |
| 8 Spectrometer Frequency | 600.13 |
| 9 Nucleus | 1H |

**S35**

Chemical Formula: $C_{26}H_{26}N_2O_5$

Exact Mass: 446.1842

Molecular Weight: 446.5030

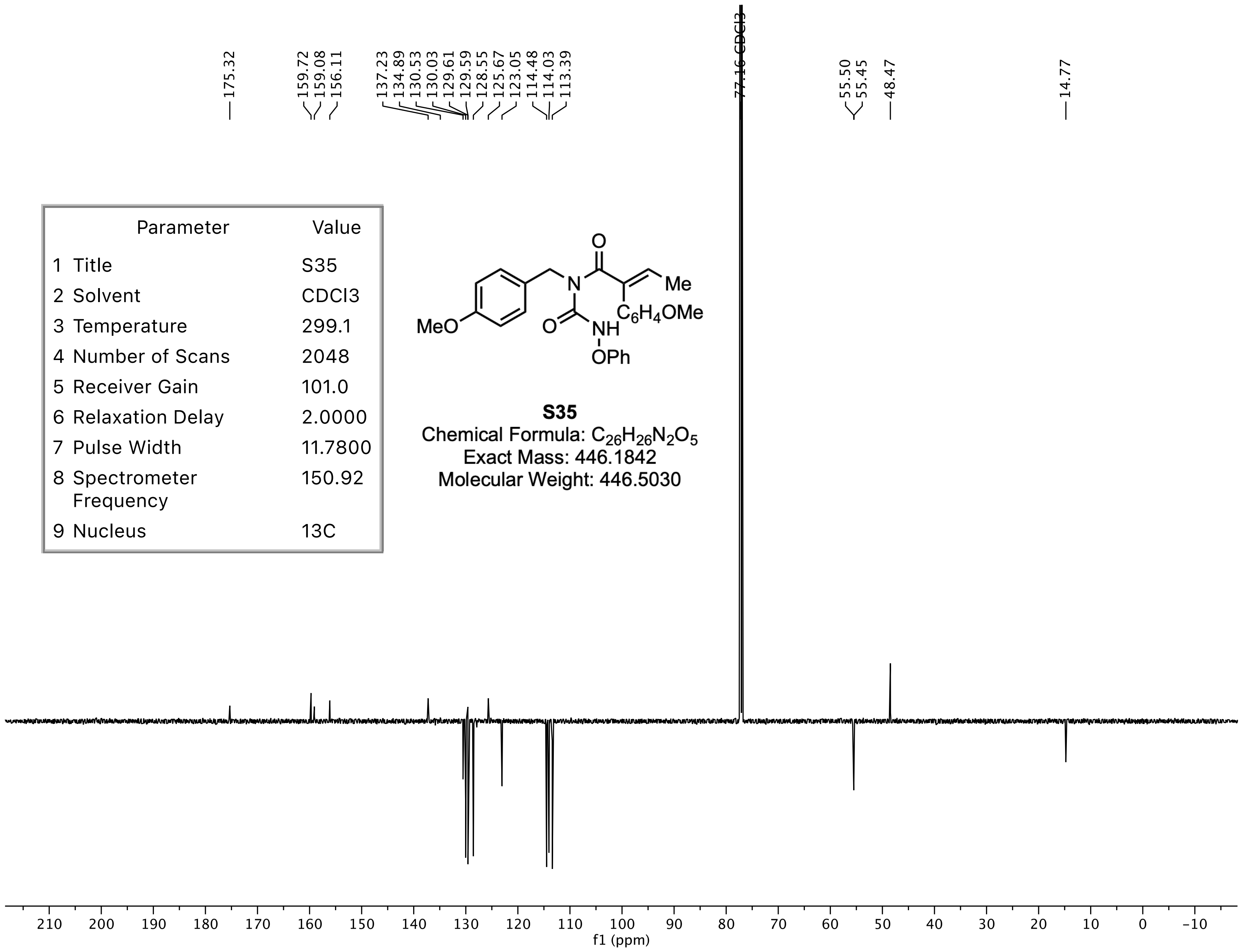


| | Parameter | Value |
|---|---|---|
| 1 | Title | S35 |
| 2 | Solvent | CDCl3 |
| 3 | Temperature | 299.1 |
| 4 | Number of Scans | 2048 |
| 5 | Receiver Gain | 101.0 |
| 6 | Relaxation Delay | 2.0000 |
| 7 | Pulse Width | 11.7800 |
| 8 | Spectrometer Frequency | 150.92 |
| 9 | Nucleus | 13C |

**S35**

Chemical Formula: $C_{26}H_{26}N_2O_5$

Exact Mass: 446.1842

Molecular Weight: 446.5030

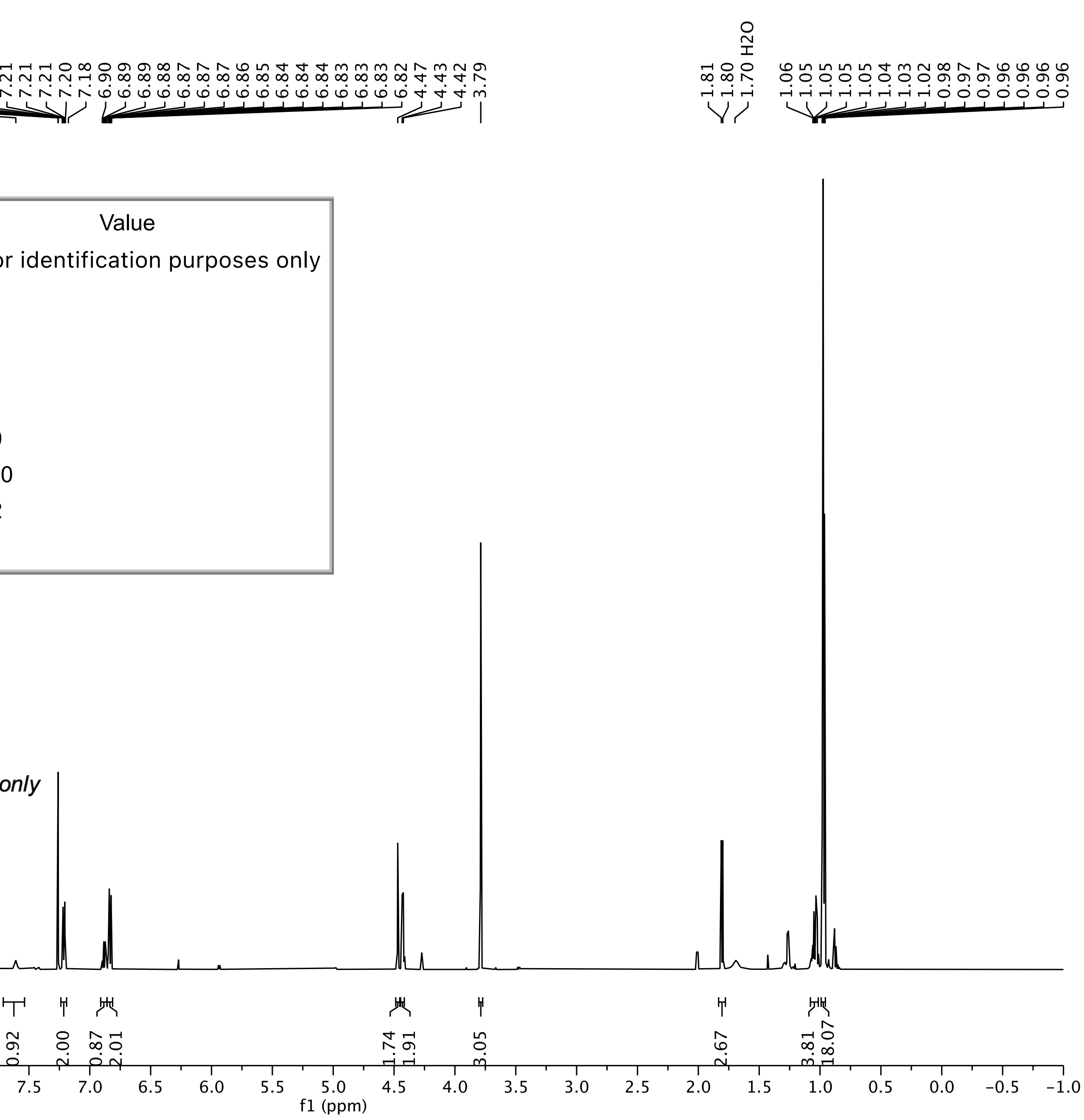


| Parameter | Value |
|---|---|
| 1 Title | S36 for identification purposes only |
| 2 Solvent | CDCl3 |
| 3 Temperature | 298.0 |
| 4 Number of Scans | 16 |
| 5 Receiver Gain | 64.0 |
| 6 Relaxation Delay | 1.0000 |
| 7 Pulse Width | 11.2250 |
| 8 Spectrometer Frequency | 600.32 |
| 9 Nucleus | 1H |

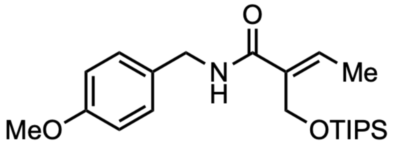


**S36**

*85% purity; for identification purposes only*

Chemical Formula: $C_{22}H_{37}NO_3Si$

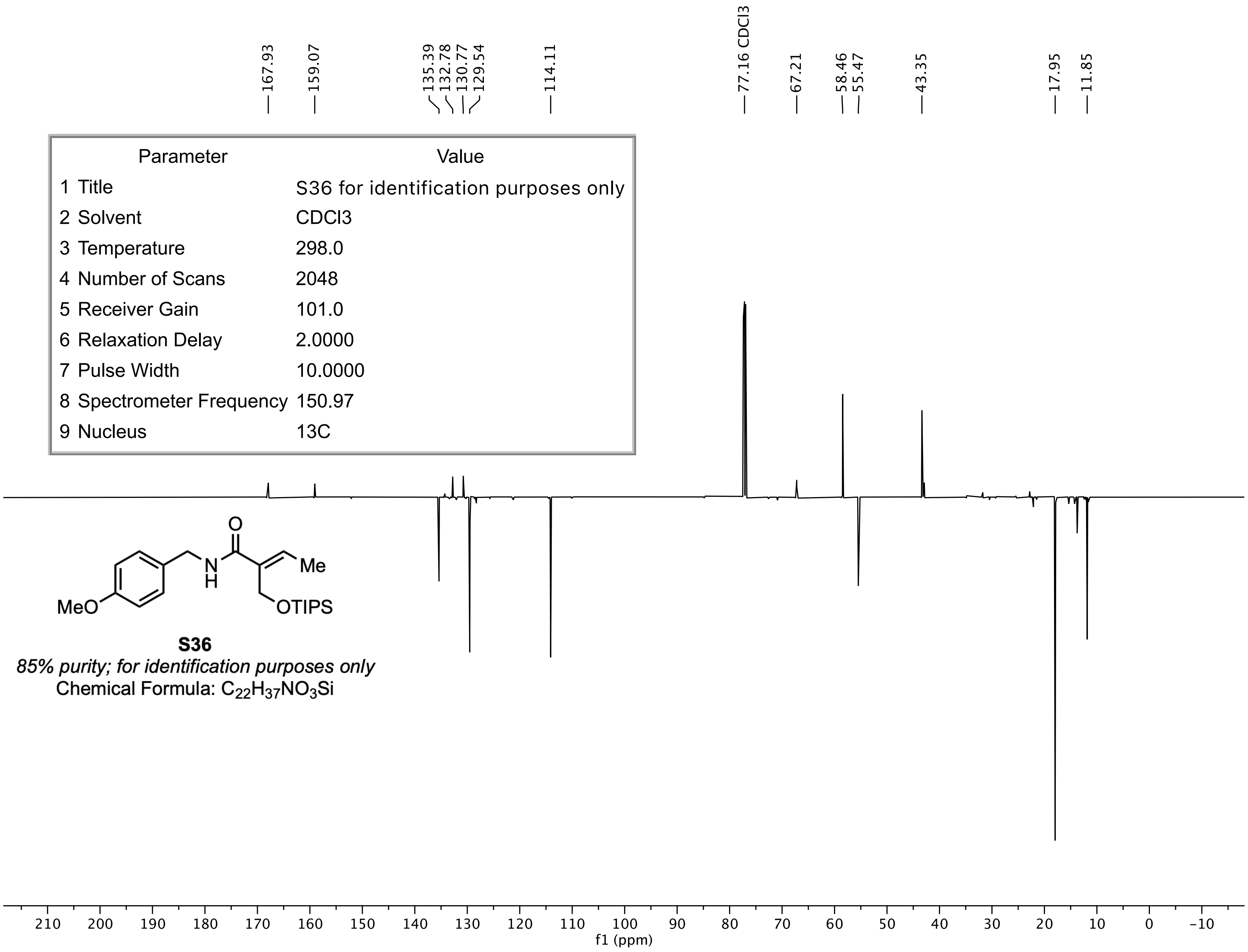


| Parameter | Value |
|---|---|
| 1 Title | S36 for identification purposes only |
| 2 Solvent | CDCl3 |
| 3 Temperature | 298.0 |
| 4 Number of Scans | 2048 |
| 5 Receiver Gain | 101.0 |
| 6 Relaxation Delay | 2.0000 |
| 7 Pulse Width | 10.0000 |
| 8 Spectrometer Frequency | 150.97 |
| 9 Nucleus | 13C |



**S36**

*85% purity; for identification purposes only*

Chemical Formula: $C_{22}H_{37}NO_3Si$

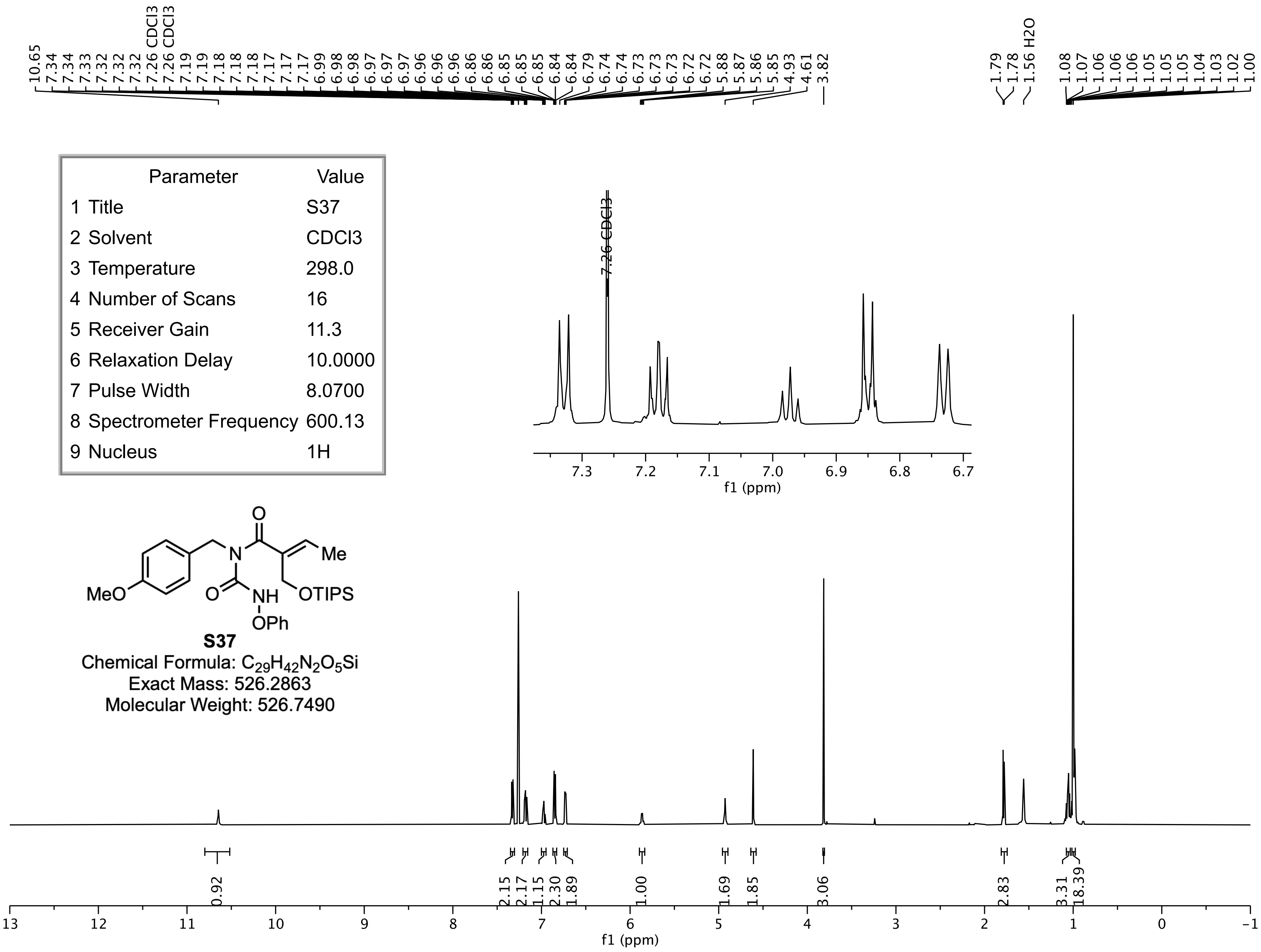


| Parameter | Value |
|---|---|
| 1 Title | S37 |
| 2 Solvent | CDCl3 |
| 3 Temperature | 298.0 |
| 4 Number of Scans | 16 |
| 5 Receiver Gain | 11.3 |
| 6 Relaxation Delay | 10.0000 |
| 7 Pulse Width | 8.0700 |
| 8 Spectrometer Frequency | 600.13 |
| 9 Nucleus | 1H |

**S37**

Chemical Formula: $C_{29}H_{42}N_2O_5Si$

Exact Mass: 526.2863

Molecular Weight: 526.7490

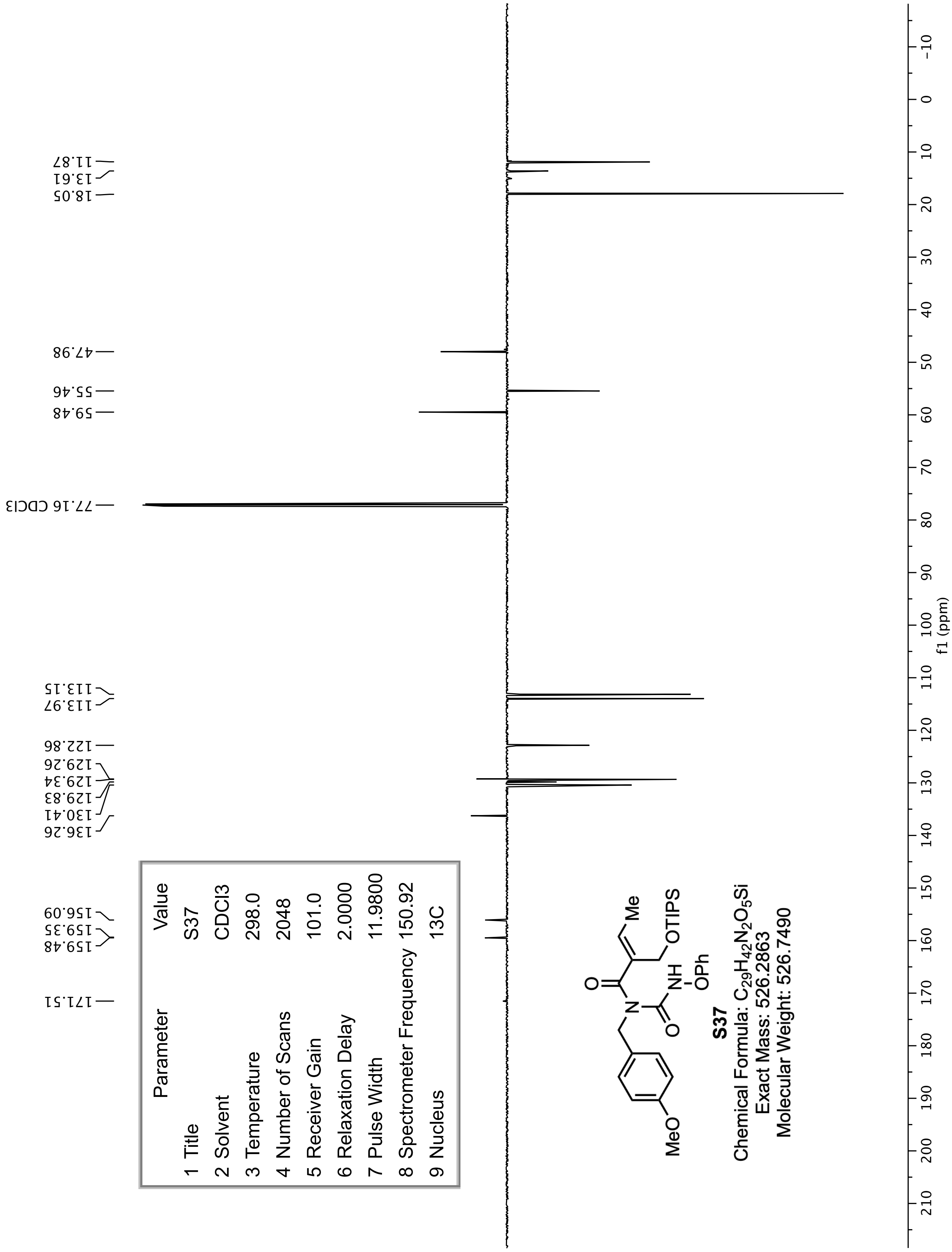


| | Parameter | Value |
|---|---|---|
| 1 | Title | S37 |
| 2 | Solvent | CDCl3 |
| 3 | Temperature | 298.0 |
| 4 | Number of Scans | 2048 |
| 5 | Receiver Gain | 101.0 |
| 6 | Relaxation Delay | 2.0000 |
| 7 | Pulse Width | 11.9800 |
| 8 | Spectrometer Frequency | 150.92 |
| 9 | Nucleus | 13C |

**S37**

Chemical Formula: $C_{29}H_{42}N_2O_5Si$
Exact Mass: 526.2863
Molecular Weight: 526.7490

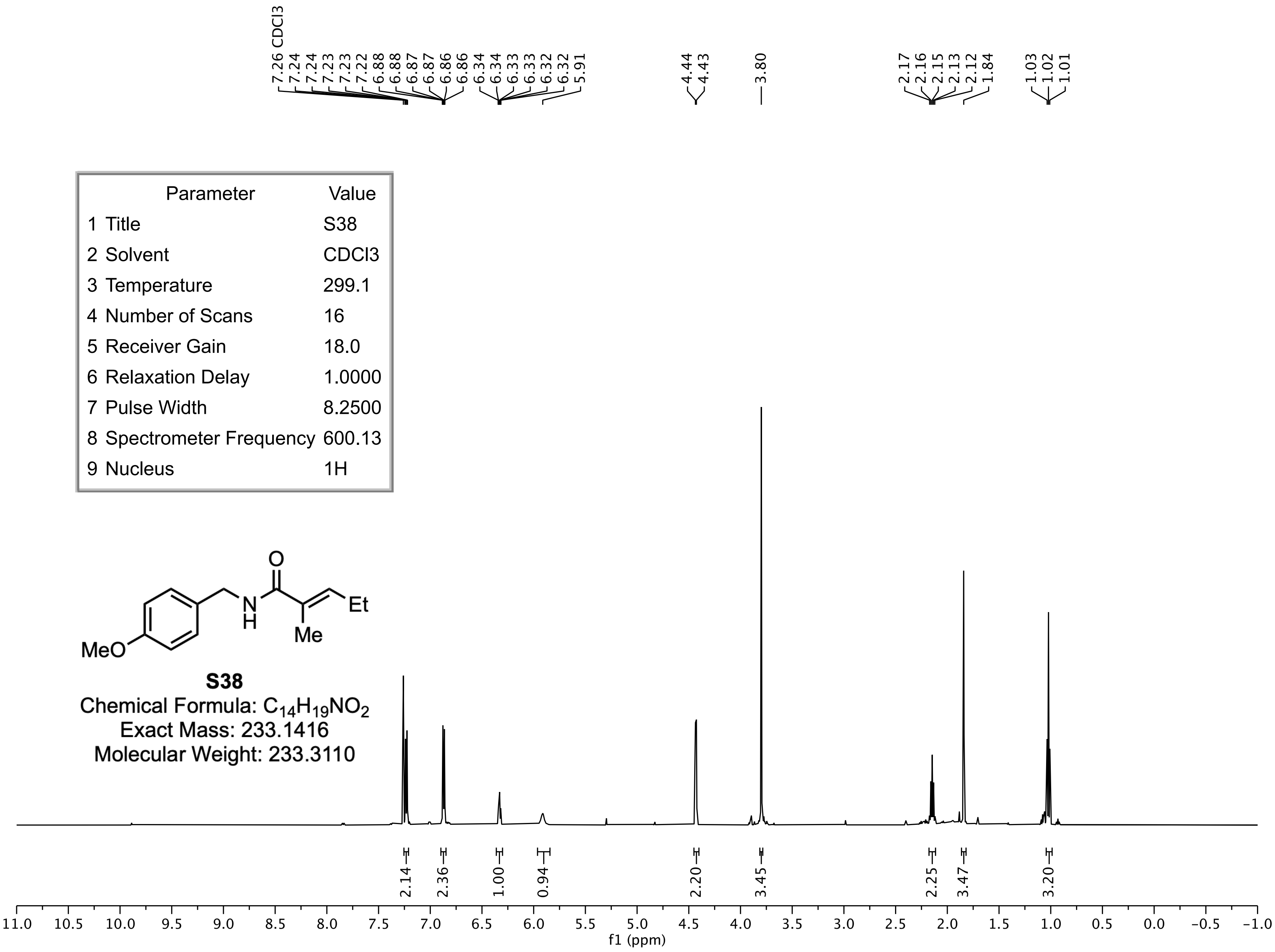


| | Parameter | Value |
|---|---|---|
| 1 | Title | S38 |
| 2 | Solvent | CDCl3 |
| 3 | Temperature | 299.1 |
| 4 | Number of Scans | 16 |
| 5 | Receiver Gain | 18.0 |
| 6 | Relaxation Delay | 1.0000 |
| 7 | Pulse Width | 8.2500 |
| 8 | Spectrometer Frequency | 600.13 |
| 9 | Nucleus | 1H |



**S38**
Chemical Formula: $C_{14}H_{19}NO_2$
Exact Mass: 233.1416
Molecular Weight: 233.3110

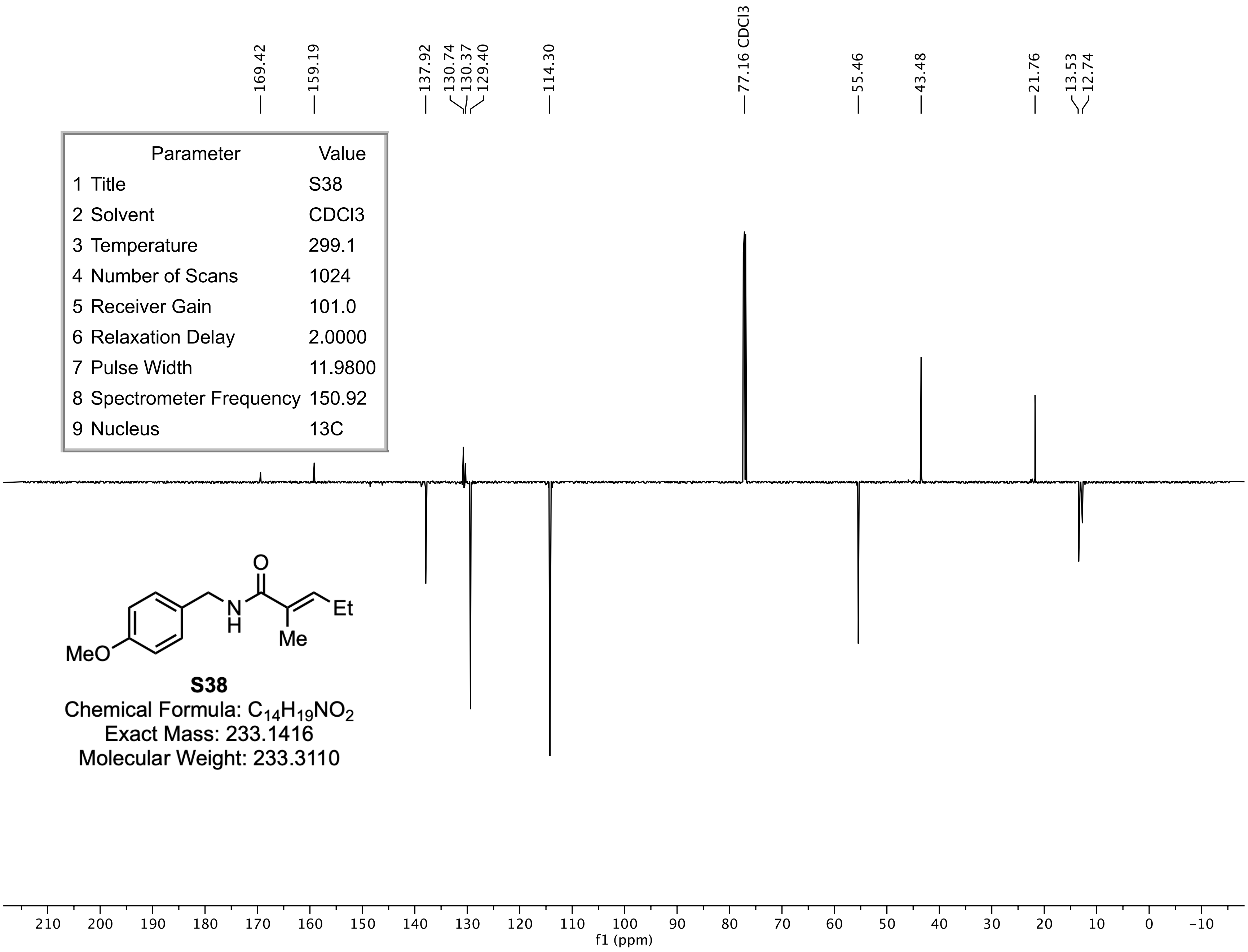


| Parameter | Value |
|---|---|
| 1 Title | S38 |
| 2 Solvent | CDCl3 |
| 3 Temperature | 299.1 |
| 4 Number of Scans | 1024 |
| 5 Receiver Gain | 101.0 |
| 6 Relaxation Delay | 2.0000 |
| 7 Pulse Width | 11.9800 |
| 8 Spectrometer Frequency | 150.92 |
| 9 Nucleus | 13C |

**S38**
Chemical Formula: $C_{14}H_{19}NO_2$
Exact Mass: 233.1416
Molecular Weight: 233.3110

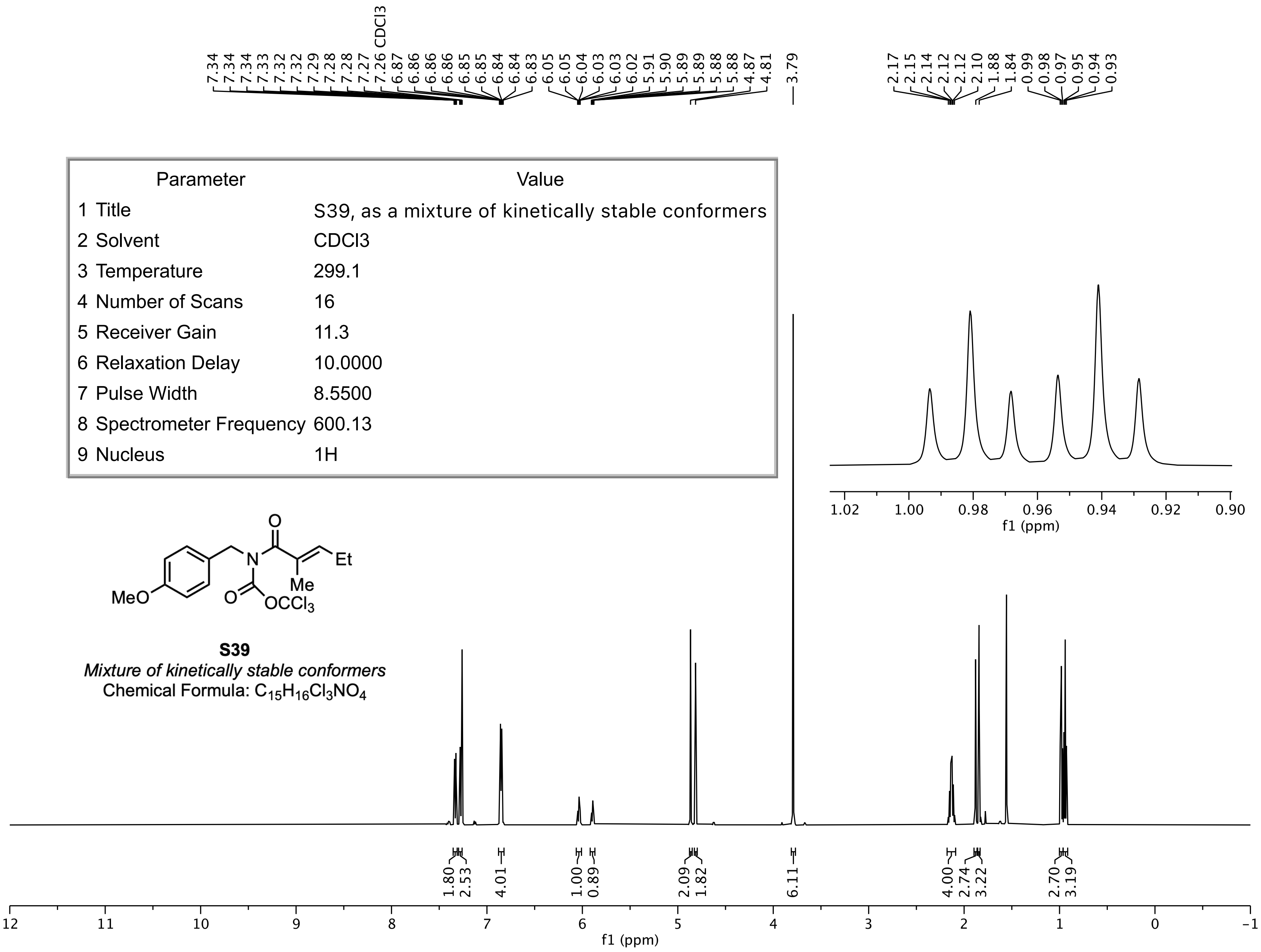


| Parameter | Value |
| --- | --- |
| 1 Title | S39, as a mixture of kinetically stable conformers |
| 2 Solvent | CDCl3 |
| 3 Temperature | 299.1 |
| 4 Number of Scans | 16 |
| 5 Receiver Gain | 11.3 |
| 6 Relaxation Delay | 10.0000 |
| 7 Pulse Width | 8.5500 |
| 8 Spectrometer Frequency | 600.13 |
| 9 Nucleus | 1H |



**S39**

*Mixture of kinetically stable conformers*

Chemical Formula: $C_{15}H_{16}Cl_3NO_4$

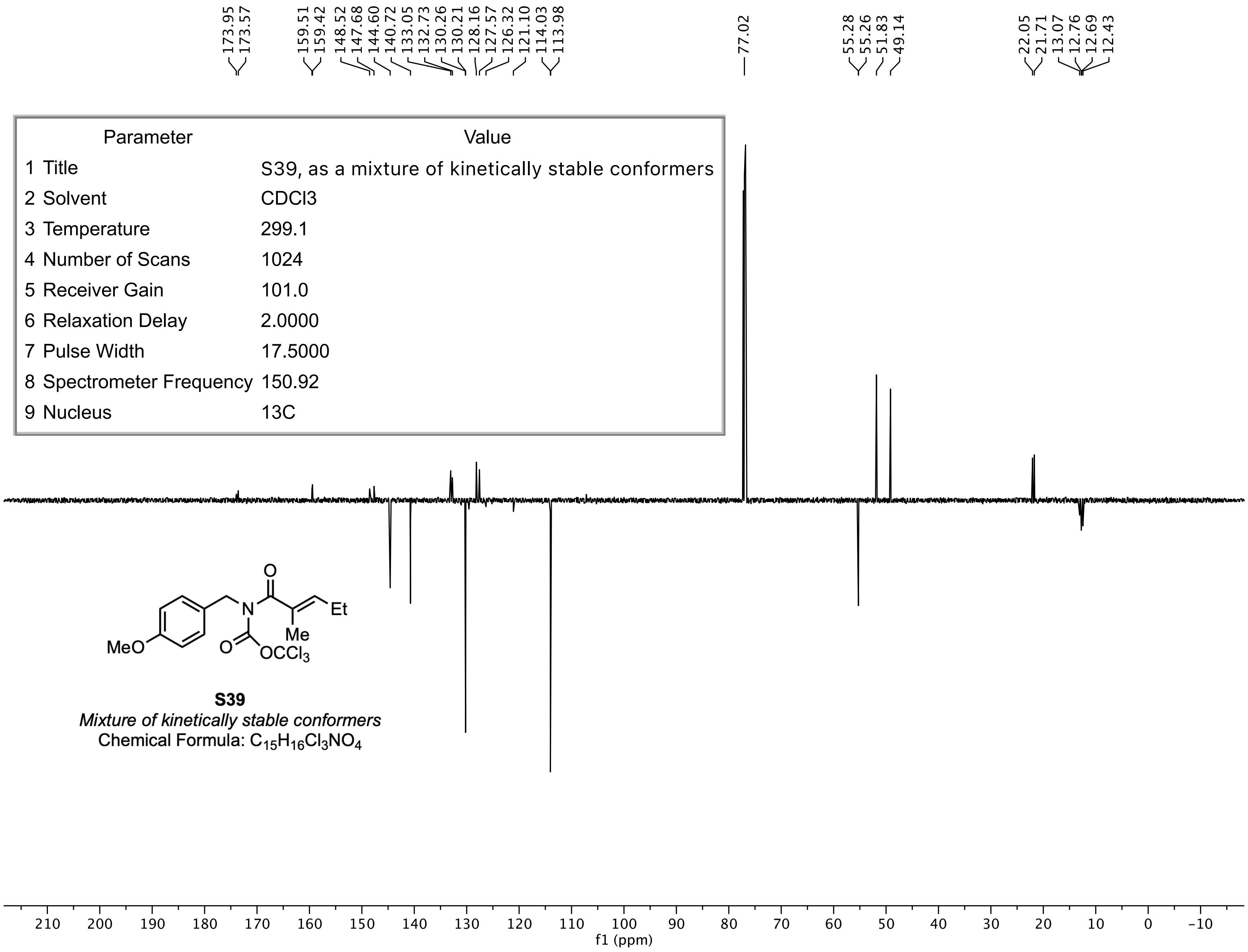


| Parameter | Value |
|---|---|
| 1 Title | S39, as a mixture of kinetically stable conformers |
| 2 Solvent | CDCl3 |
| 3 Temperature | 299.1 |
| 4 Number of Scans | 1024 |
| 5 Receiver Gain | 101.0 |
| 6 Relaxation Delay | 2.0000 |
| 7 Pulse Width | 17.5000 |
| 8 Spectrometer Frequency | 150.92 |
| 9 Nucleus | 13C |



**S39**

*Mixture of kinetically stable conformers*

Chemical Formula: $C_{15}H_{16}Cl_3NO_4$

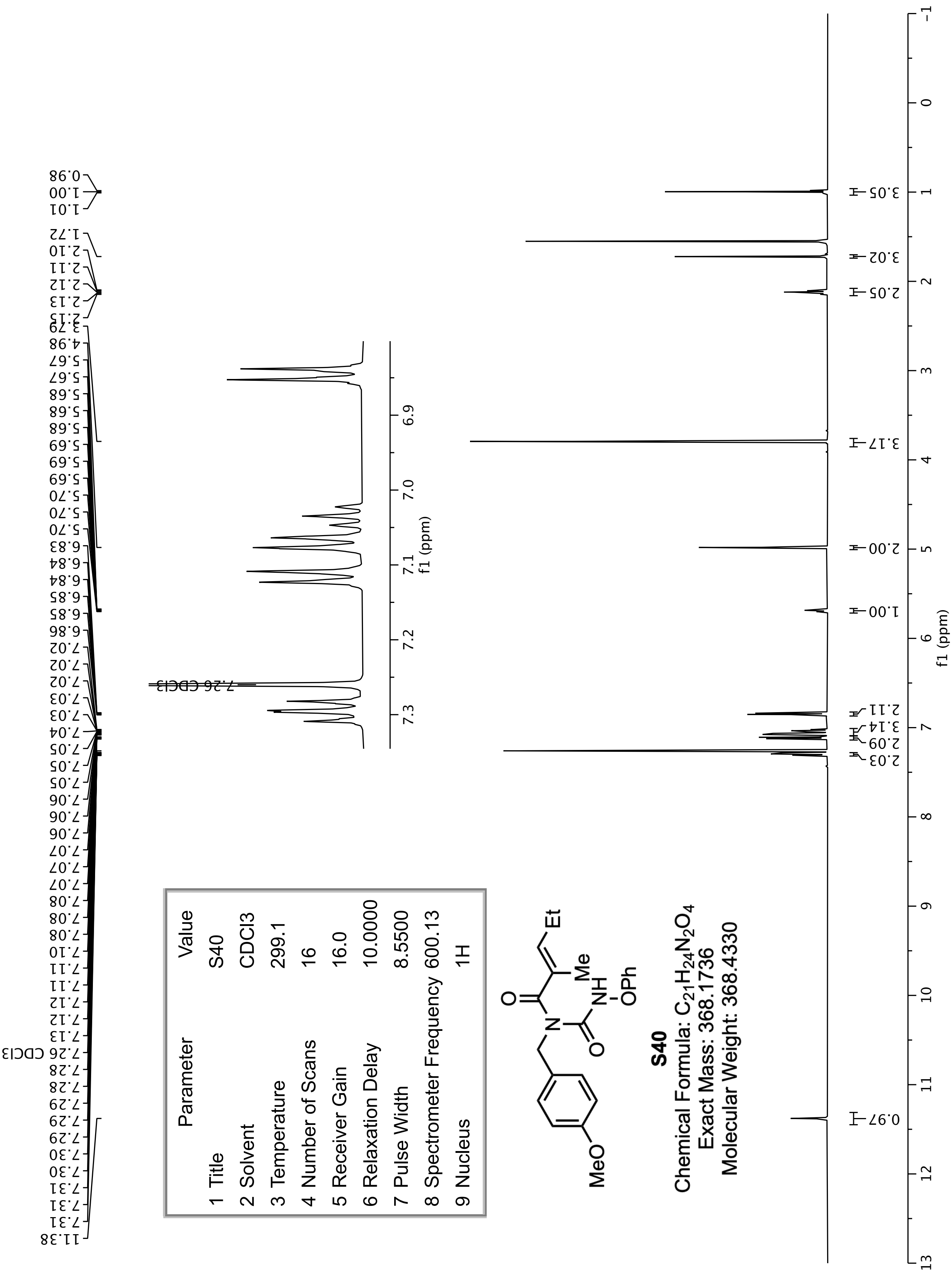


| Parameter | Value |
|---|---|
| 1 Title | S40 |
| 2 Solvent | CDCl3 |
| 3 Temperature | 299.1 |
| 4 Number of Scans | 16 |
| 5 Receiver Gain | 16.0 |
| 6 Relaxation Delay | 10.0000 |
| 7 Pulse Width | 8.5500 |
| 8 Spectrometer Frequency | 600.13 |
| 9 Nucleus | 1H |

**S40**

Chemical Formula: $C_{21}H_{24}N_2O_4$

Exact Mass: 368.1736

Molecular Weight: 368.4330

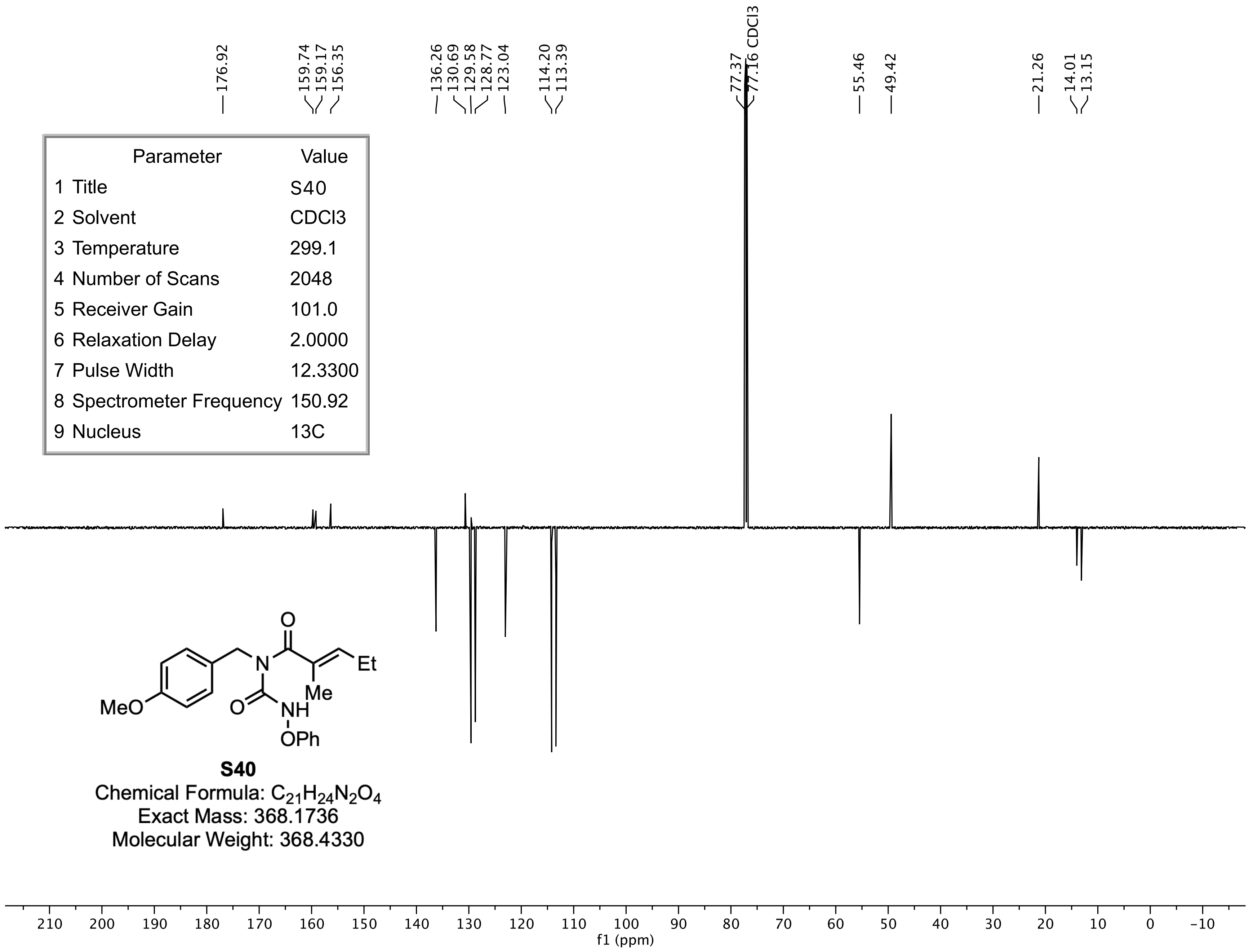


| | Parameter | Value |
|---|---|---|
| 1 | Title | S40 |
| 2 | Solvent | CDCl3 |
| 3 | Temperature | 299.1 |
| 4 | Number of Scans | 2048 |
| 5 | Receiver Gain | 101.0 |
| 6 | Relaxation Delay | 2.0000 |
| 7 | Pulse Width | 12.3300 |
| 8 | Spectrometer Frequency | 150.92 |
| 9 | Nucleus | 13C |


**S40**
Chemical Formula: $C_{21}H_{24}N_2O_4$
Exact Mass: 368.1736
Molecular Weight: 368.4330

| Parameter | Value |
|---|---|
| 1 Title | S41 |
| 2 Solvent | CDCl3 |
| 3 Temperature | 298.0 |
| 4 Number of Scans | 16 |
| 5 Receiver Gain | 12.7 |
| 6 Relaxation Delay | 10.0000 |
| 7 Pulse Width | 8.0100 |
| 8 Spectrometer Frequency | 600.13 |
| 9 Nucleus | 1H |

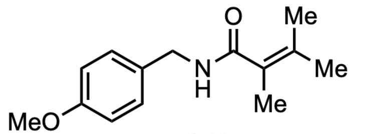


**S41**
Chemical Formula: $C_{14}H_{19}NO_2$
Exact Mass: 233.1416
Molecular Weight: 233.3110

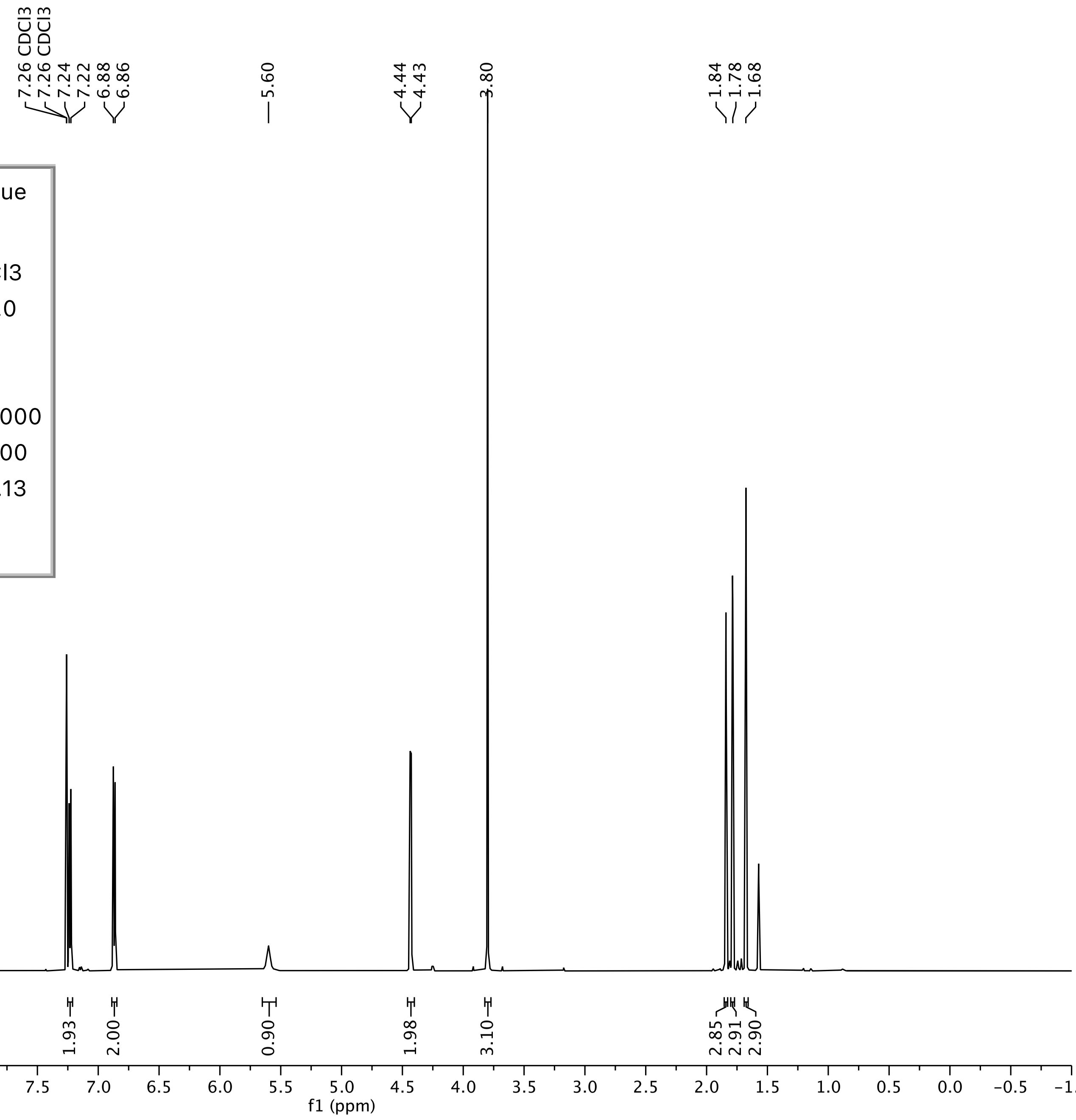

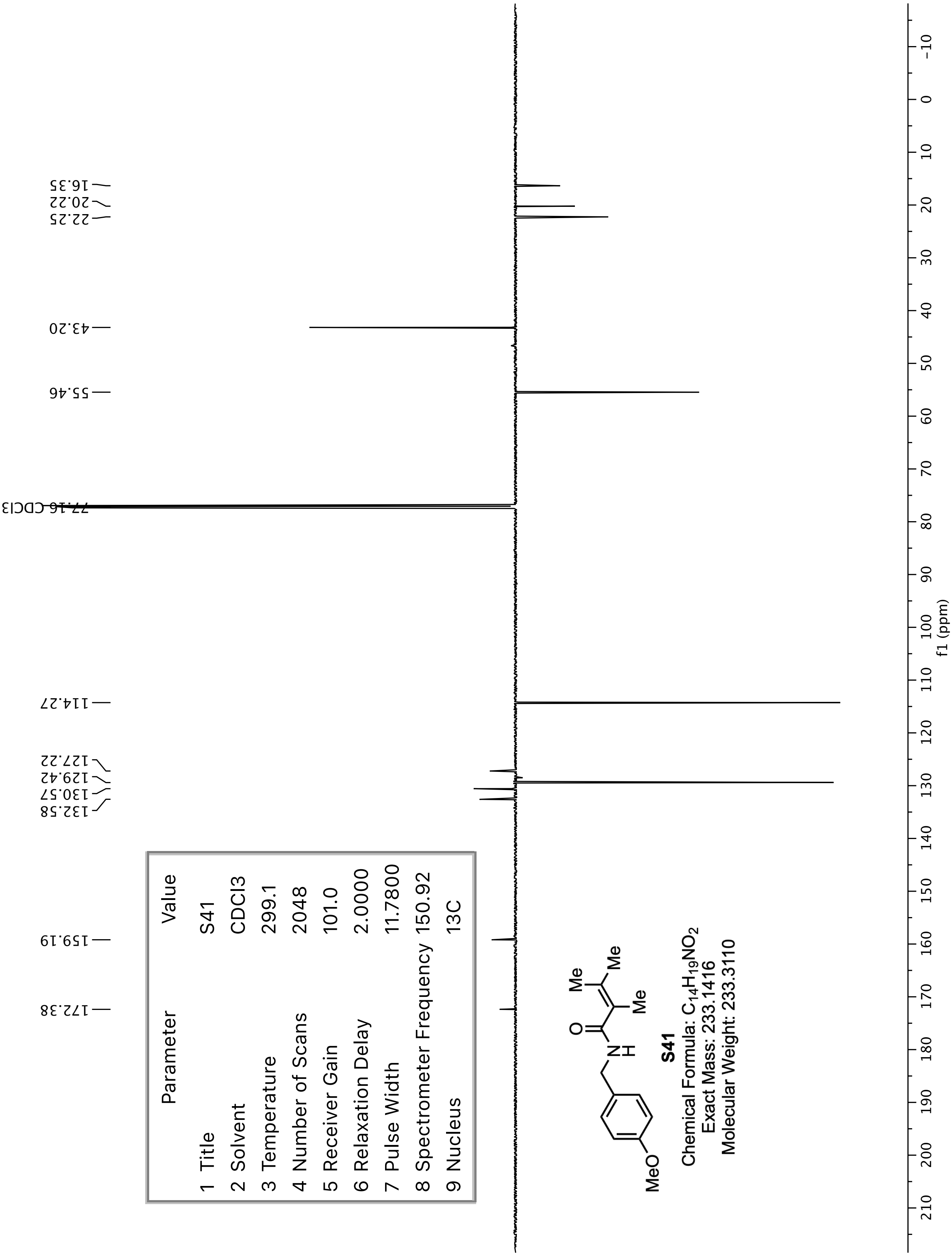


| | Parameter | Value |
|---|---|---|
| 1 | Title | S41 |
| 2 | Solvent | CDCl3 |
| 3 | Temperature | 299.1 |
| 4 | Number of Scans | 2048 |
| 5 | Receiver Gain | 101.0 |
| 6 | Relaxation Delay | 2.0000 |
| 7 | Pulse Width | 11.7800 |
| 8 | Spectrometer Frequency | 150.92 |
| 9 | Nucleus | 13C |

**S41**
Chemical Formula: $C_{14}H_{19}NO_2$
Exact Mass: 233.1416
Molecular Weight: 233.3110

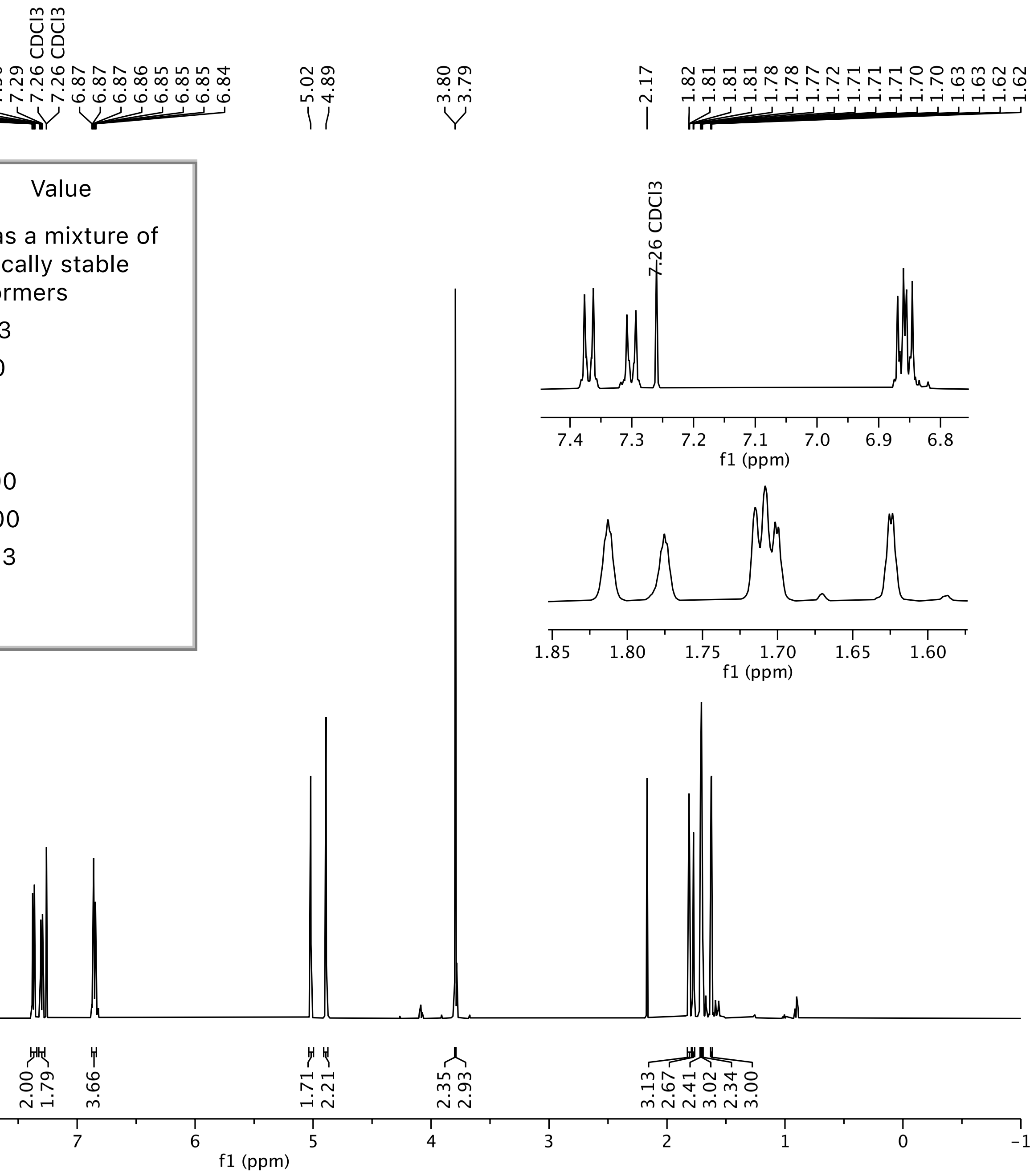


| Parameter | Value |
|---|---|
| 1 Title | S42 as a mixture of kinetically stable conformers |
| 2 Solvent | CDCl3 |
| 3 Temperature | 298.0 |
| 4 Number of Scans | 16 |
| 5 Receiver Gain | 12.7 |
| 6 Relaxation Delay | 1.0000 |
| 7 Pulse Width | 8.2200 |
| 8 Spectrometer Frequency | 600.13 |
| 9 Nucleus | 1H |

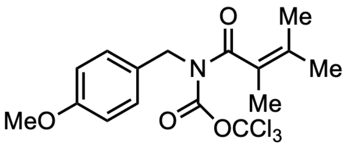


**S42**

*Mixture of kinetically stable conformers*

Chemical Formula: $C_{16}H_{18}Cl_3NO_4$

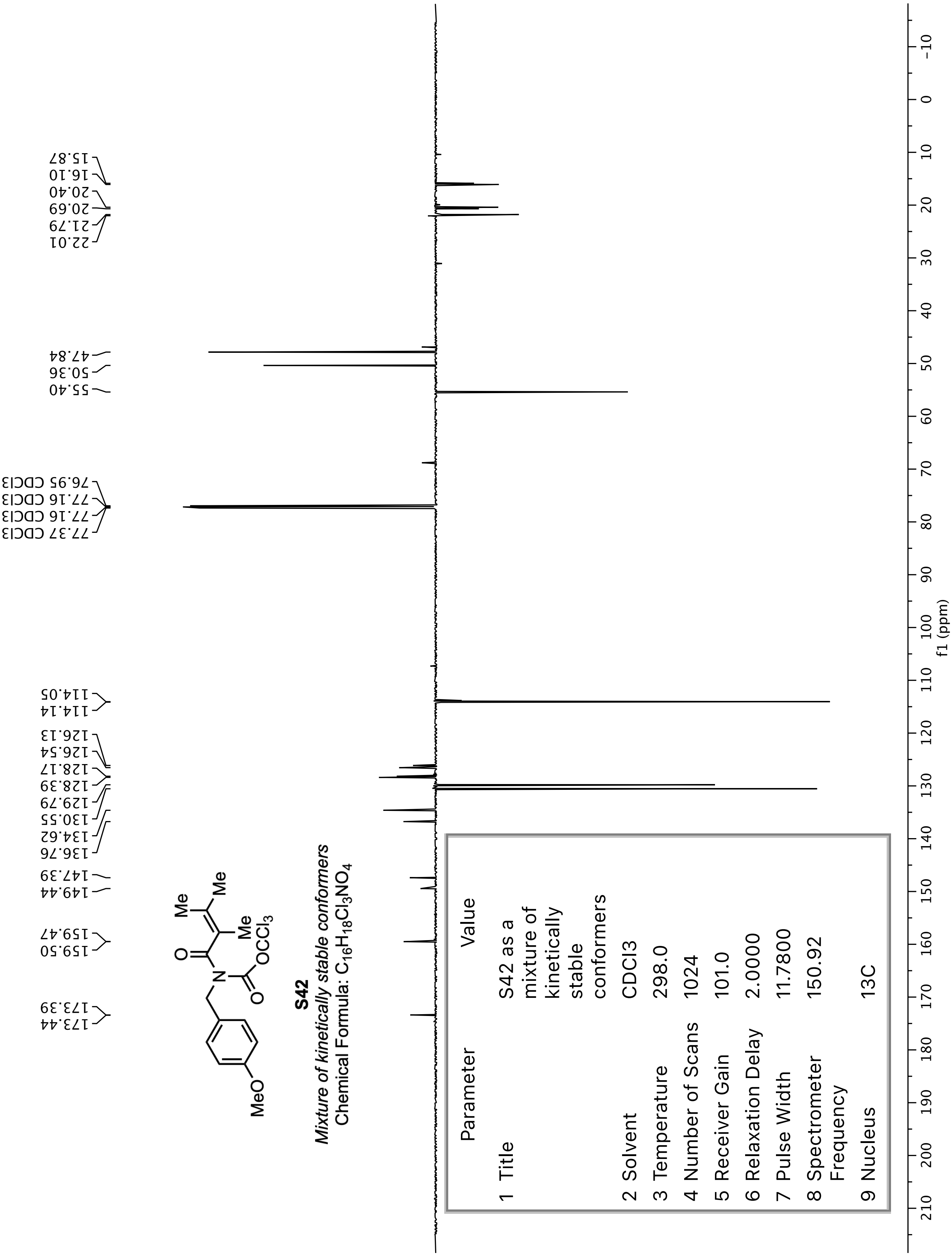
15.87
16.10
20.40
20.69
21.79
22.01
47.84
50.36
55.40
76.95 CDCl3
77.16 CDCl3
77.16 CDCl3
77.37 CDCl3
114.05
114.14
126.13
126.54
128.17
128.39
129.79
130.55
134.62
136.76
147.39
149.44
159.47
159.50
173.39
173.44
MeO
O
N
O
OCCl3
Me
Me
Me
S42
Mixture of kinetically stable conformers
Chemical Formula: C16H18Cl3NO4
Parameter | Value
1 Title | S42 as a mixture of kinetically stable conformers
2 Solvent | CDCl3
3 Temperature | 298.0
4 Number of Scans | 1024
5 Receiver Gain | 101.0
6 Relaxation Delay | 2.0000
7 Pulse Width | 11.7800
8 Spectrometer Frequency | 150.92
9 Nucleus | 13C
f1 (ppm)

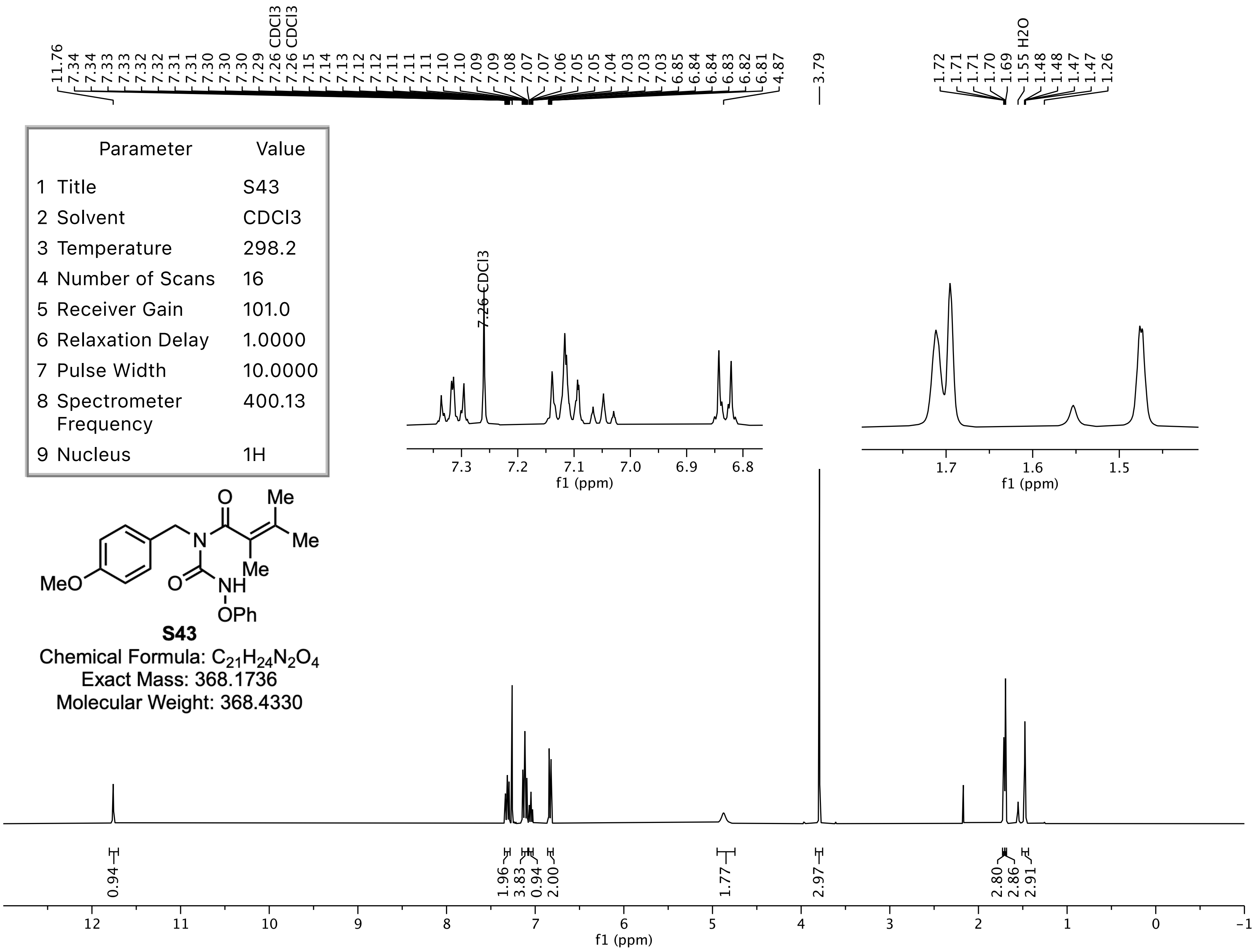


| | Parameter | Value |
|---|---|---|
| 1 | Title | S43 |
| 2 | Solvent | CDCl3 |
| 3 | Temperature | 298.2 |
| 4 | Number of Scans | 16 |
| 5 | Receiver Gain | 101.0 |
| 6 | Relaxation Delay | 1.0000 |
| 7 | Pulse Width | 10.0000 |
| 8 | Spectrometer Frequency | 400.13 |
| 9 | Nucleus | 1H |

**S43**

Chemical Formula: $C_{21}H_{24}N_2O_4$

Exact Mass: 368.1736

Molecular Weight: 368.4330

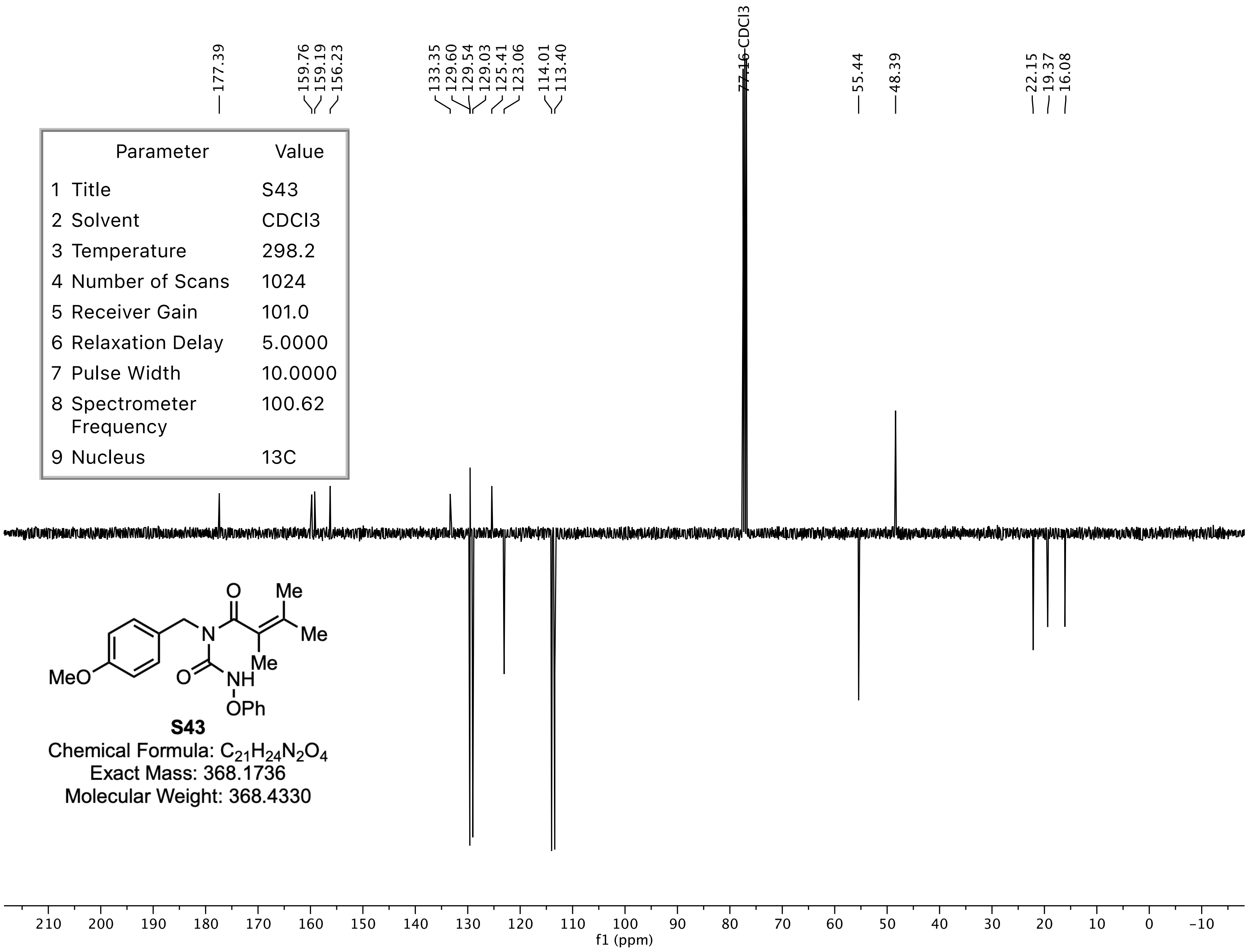


| Parameter | Value |
|---|---|
| 1 Title | S43 |
| 2 Solvent | CDCl3 |
| 3 Temperature | 298.2 |
| 4 Number of Scans | 1024 |
| 5 Receiver Gain | 101.0 |
| 6 Relaxation Delay | 5.0000 |
| 7 Pulse Width | 10.0000 |
| 8 Spectrometer Frequency | 100.62 |
| 9 Nucleus | 13C |

**S43**

Chemical Formula: $C_{21}H_{24}N_2O_4$

Exact Mass: 368.1736

Molecular Weight: 368.4330

| Parameter | Value |
|---|---|
| 1 Title | S44 |
| 2 Solvent | CDCl3 |
| 3 Temperature | 298.0 |
| 4 Number of Scans | 16 |
| 5 Receiver Gain | 101.0 |
| 6 Relaxation Delay | 10.0000 |
| 7 Pulse Width | 15.0000 |
| 8 Spectrometer Frequency | 400.13 |
| 9 Nucleus | 1H |

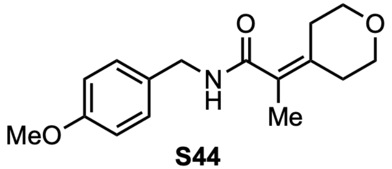


**S44**
Chemical Formula: $C_{16}H_{21}NO_3$
Exact Mass: 275.1521
Molecular Weight: 275.3480

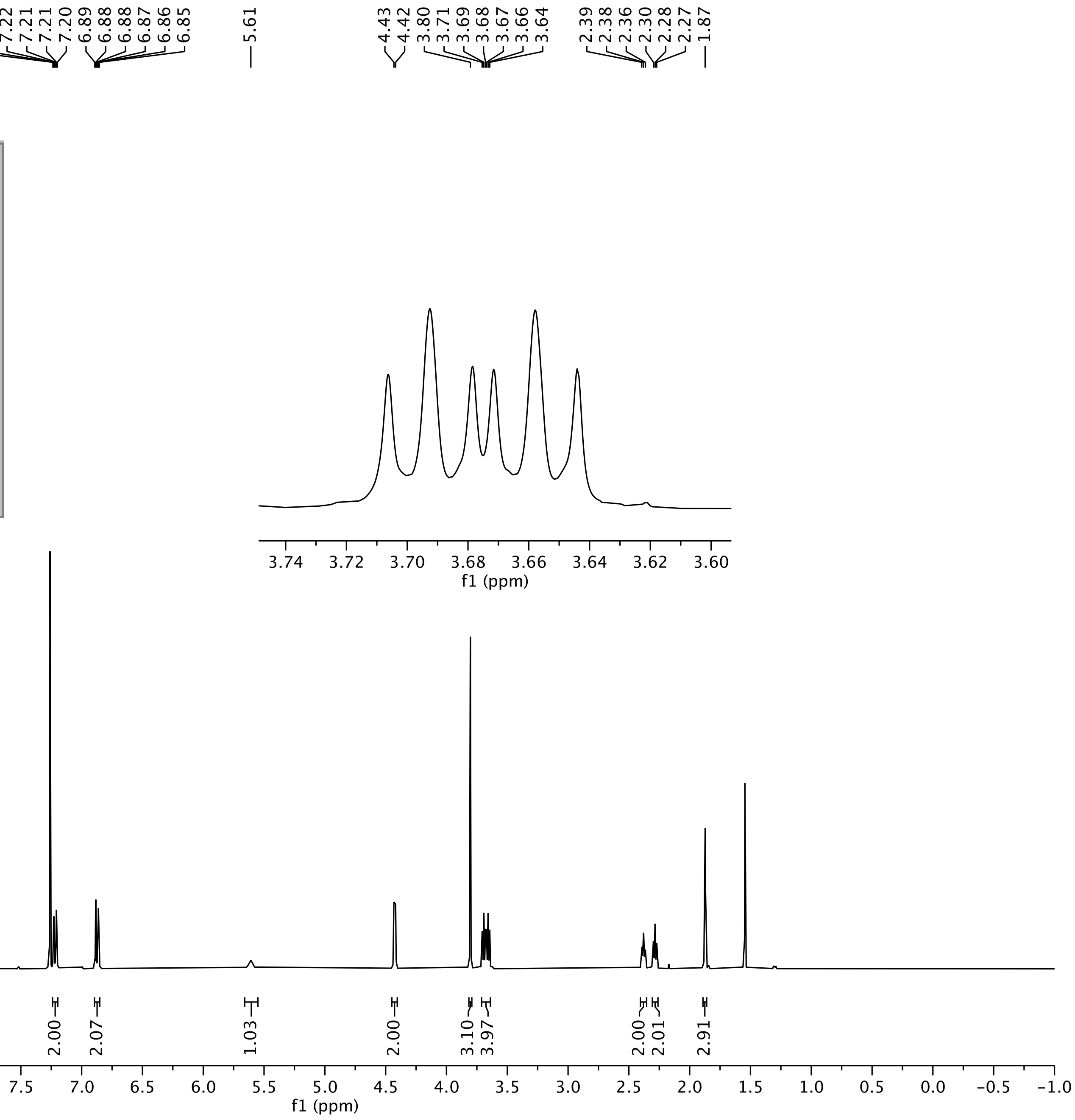

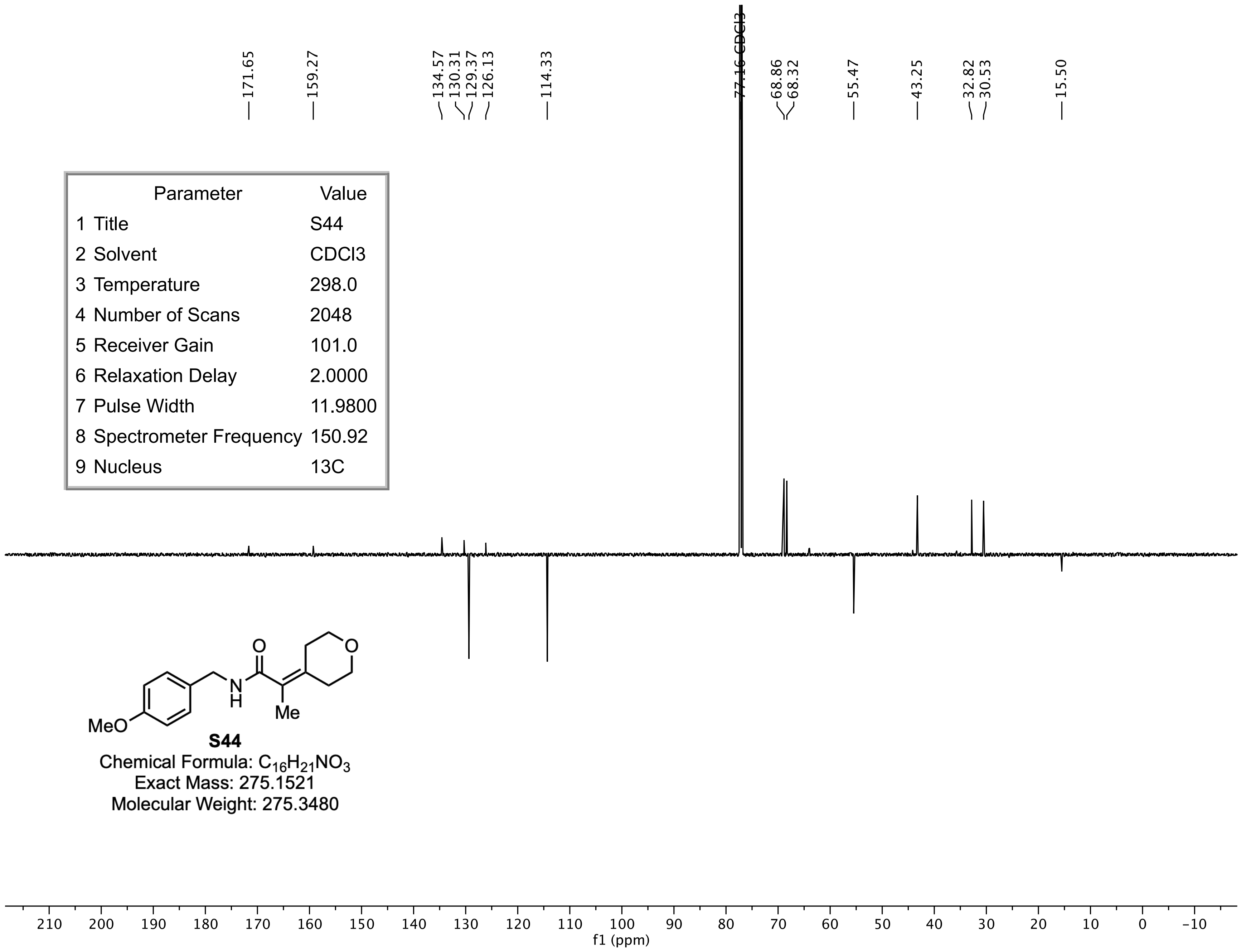


| | Parameter | Value |
|---|---|---|
| 1 | Title | S44 |
| 2 | Solvent | CDCl3 |
| 3 | Temperature | 298.0 |
| 4 | Number of Scans | 2048 |
| 5 | Receiver Gain | 101.0 |
| 6 | Relaxation Delay | 2.0000 |
| 7 | Pulse Width | 11.9800 |
| 8 | Spectrometer Frequency | 150.92 |
| 9 | Nucleus | 13C |

**S44**

Chemical Formula: $C_{16}H_{21}NO_3$

Exact Mass: 275.1521

Molecular Weight: 275.3480

| Parameter | Value |
|---|---|
| 1 Title | S45 as a mixture of kinetically stable conformers |
| 2 Solvent | CDCl3 |
| 3 Temperature | 300.0 |
| 4 Number of Scans | 16 |
| 5 Receiver Gain | 64.0 |
| 6 Relaxation Delay | 10.0000 |
| 7 Pulse Width | 12.0000 |
| 8 Spectrometer Frequency | 600.32 |
| 9 Nucleus | 1H |

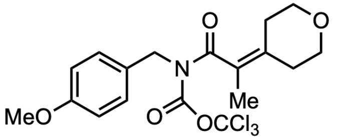


**S45**

*Mixture of kinetically stable conformers*

Chemical Formula: $C_{18}H_{20}Cl_3NO_5$

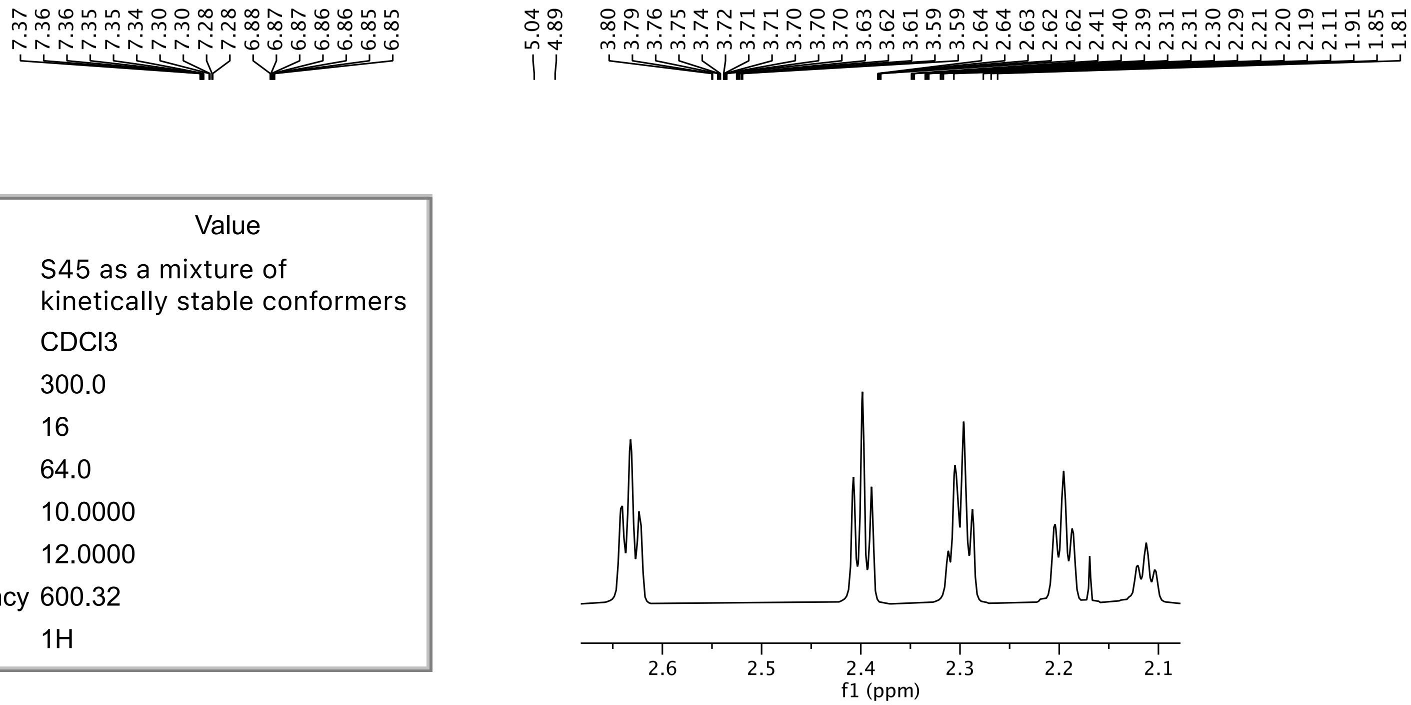
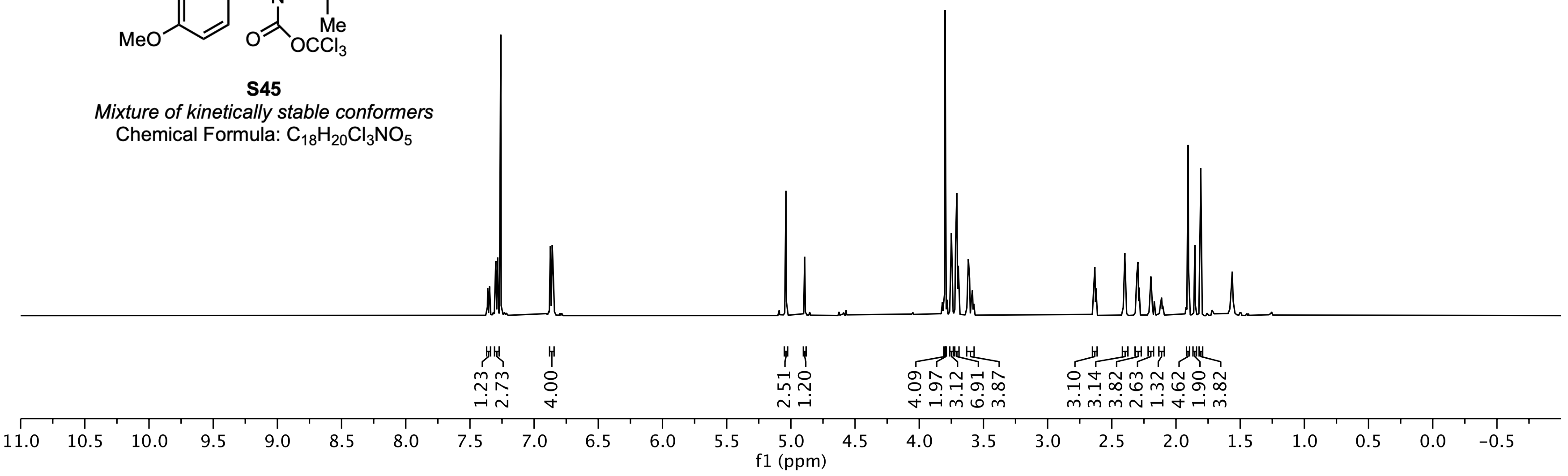

| Parameter | Value |
|---|---|
| 1 Title | S45 as a mixture of kinetically stable conformers |
| 2 Solvent | CDCl3 |
| 3 Temperature | 300.0 |
| 4 Number of Scans | 1024 |
| 5 Receiver Gain | 2050.0 |
| 6 Relaxation Delay | 5.0000 |
| 7 Pulse Width | 10.0000 |
| 8 Spectrometer Frequency | 150.97 |
| 9 Nucleus | 13C |

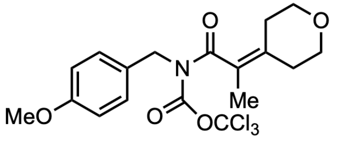


**S45**

*Mixture of kinetically stable conformers*

Chemical Formula: $C_{18}H_{20}Cl_3NO_5$

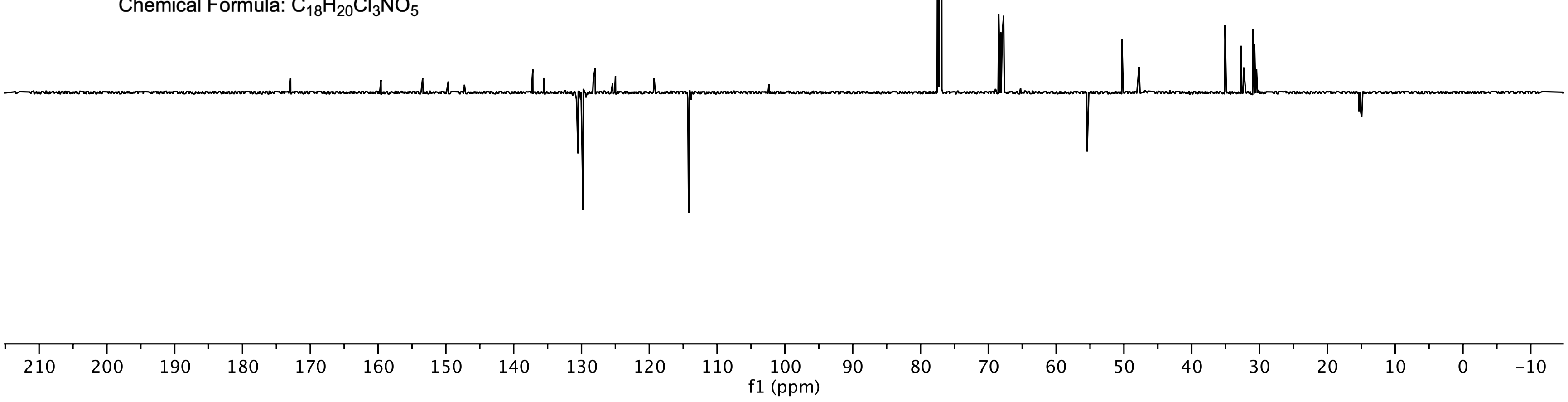

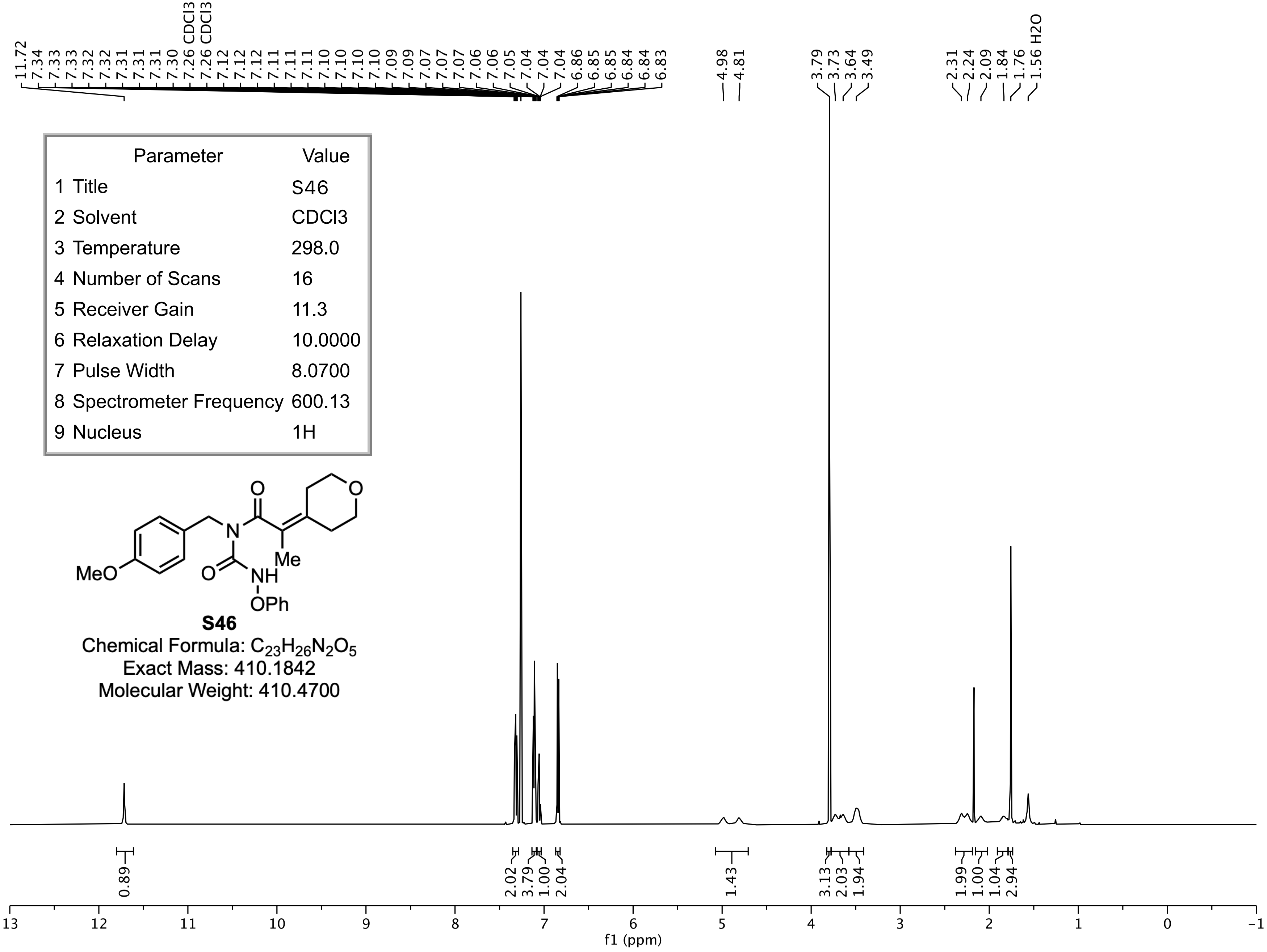


| Parameter | Value |
|---|---|
| 1 Title | S46 |
| 2 Solvent | CDCl3 |
| 3 Temperature | 298.0 |
| 4 Number of Scans | 16 |
| 5 Receiver Gain | 11.3 |
| 6 Relaxation Delay | 10.0000 |
| 7 Pulse Width | 8.0700 |
| 8 Spectrometer Frequency | 600.13 |
| 9 Nucleus | 1H |

**S46**

Chemical Formula: $C_{23}H_{26}N_2O_5$

Exact Mass: 410.1842

Molecular Weight: 410.4700

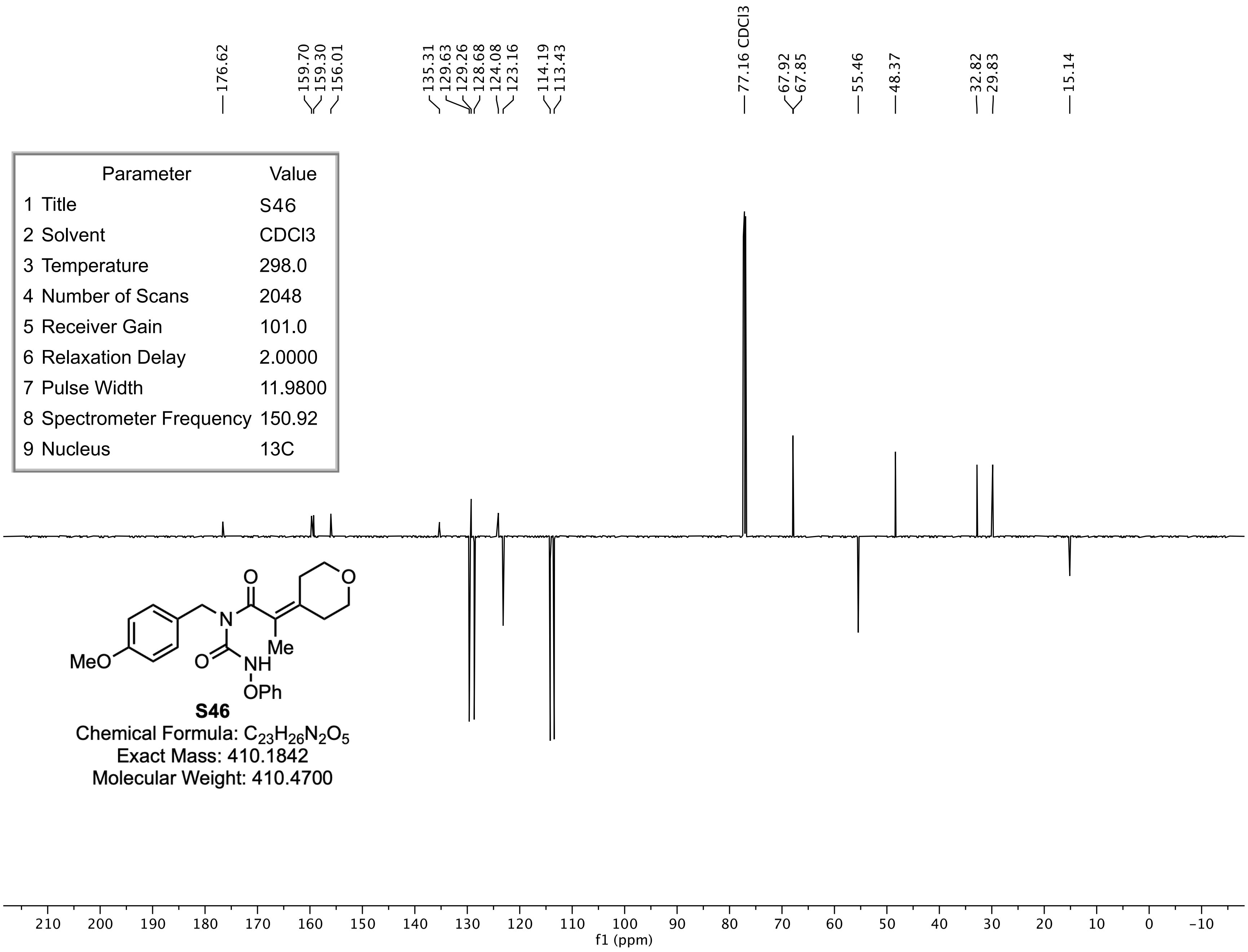


| Parameter | Value |
|---|---|
| 1 Title | S46 |
| 2 Solvent | CDCl3 |
| 3 Temperature | 298.0 |
| 4 Number of Scans | 2048 |
| 5 Receiver Gain | 101.0 |
| 6 Relaxation Delay | 2.0000 |
| 7 Pulse Width | 11.9800 |
| 8 Spectrometer Frequency | 150.92 |
| 9 Nucleus | 13C |



**S46**

Chemical Formula: $C_{23}H_{26}N_2O_5$

Exact Mass: 410.1842

Molecular Weight: 410.4700

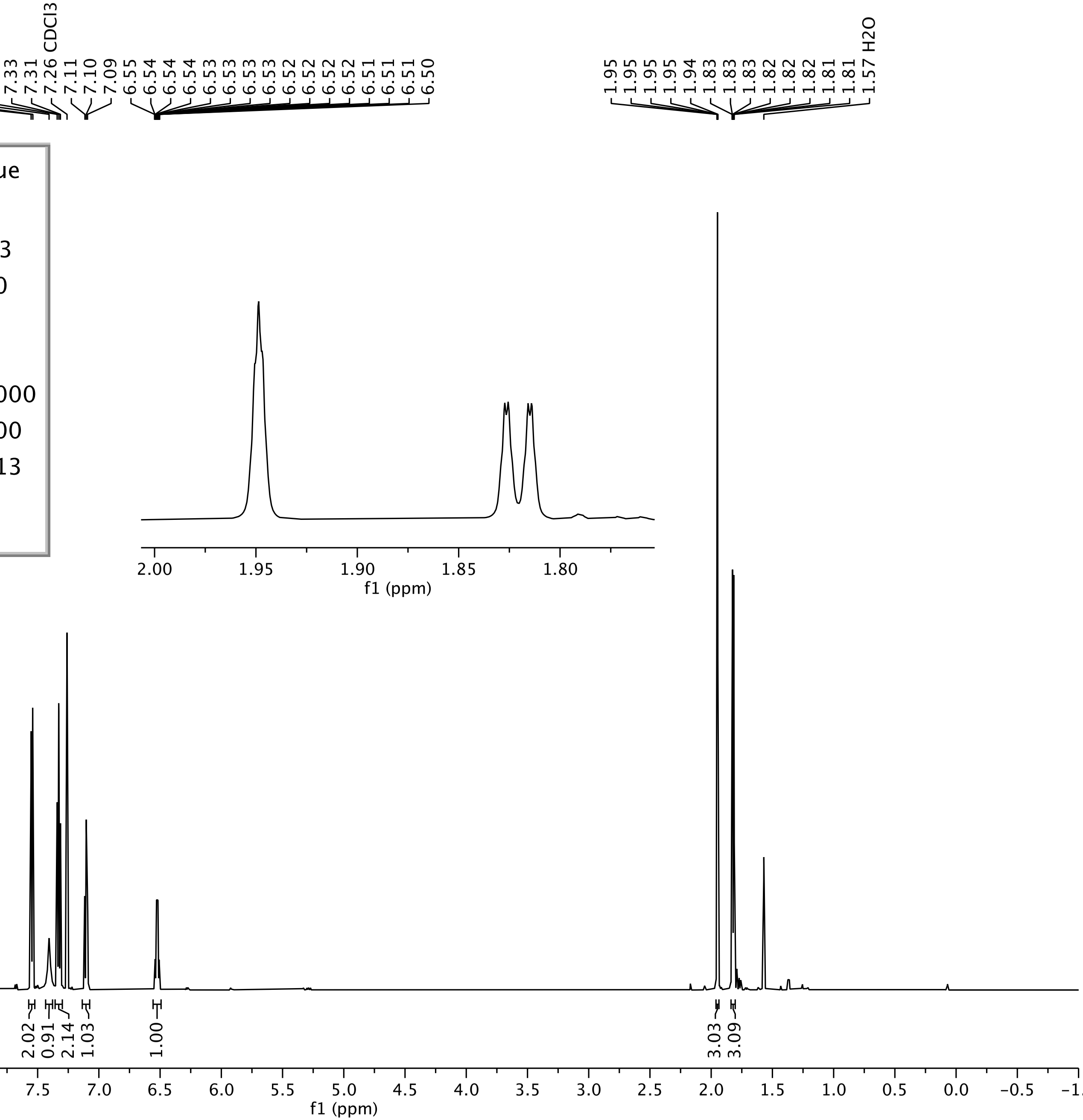


| Parameter | Value |
|---|---|
| 1 Title | S47 |
| 2 Solvent | CDCl3 |
| 3 Temperature | 298.0 |
| 4 Number of Scans | 16 |
| 5 Receiver Gain | 11.3 |
| 6 Relaxation Delay | 10.0000 |
| 7 Pulse Width | 8.0100 |
| 8 Spectrometer Frequency | 600.13 |
| 9 Nucleus | 1H |

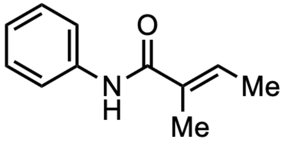


**S47**

Chemical Formula: $C_{11}H_{13}NO$

Exact Mass: 175.0997

Molecular Weight: 175.2310

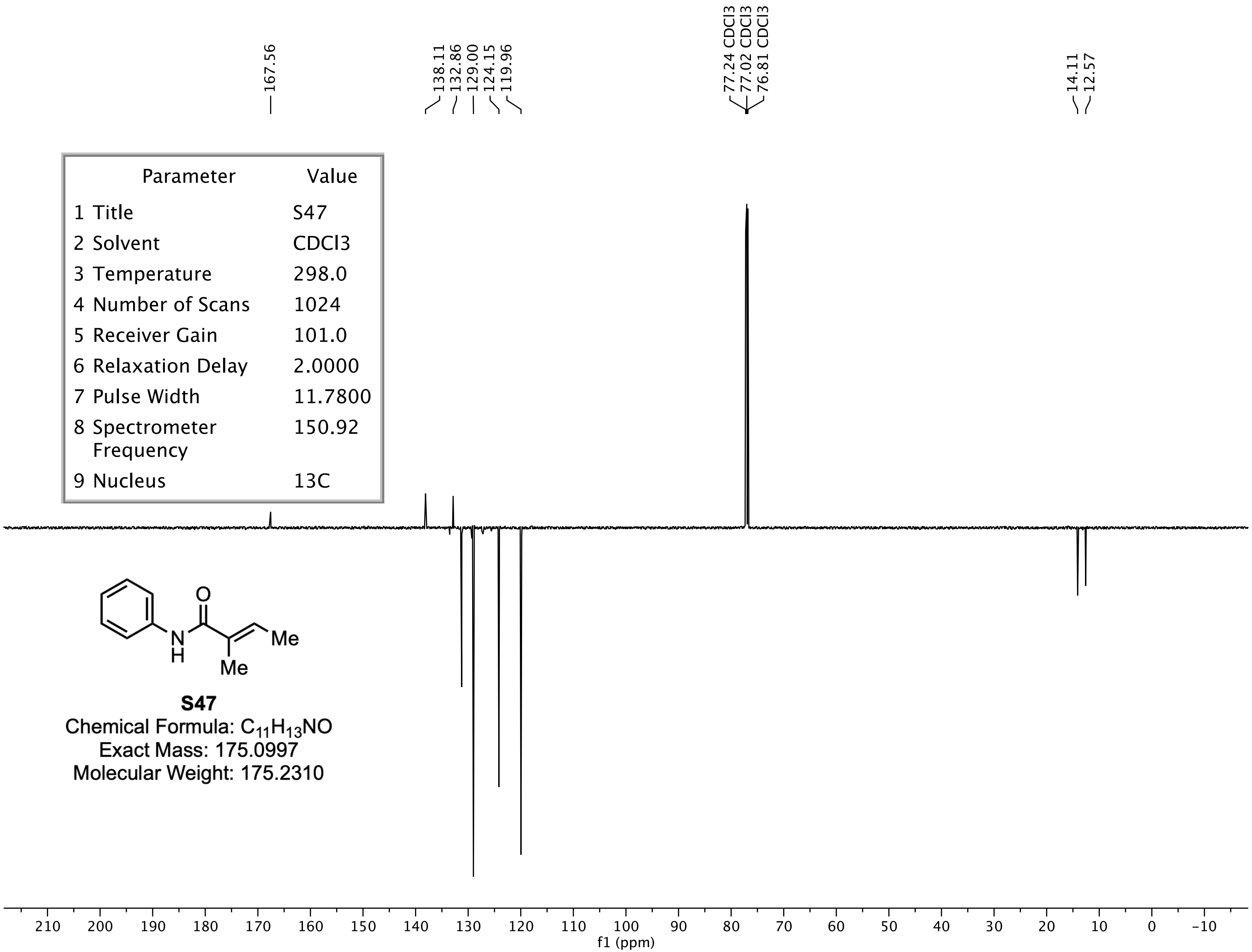


| | Parameter | Value |
|---|---|---|
| 1 | Title | S47 |
| 2 | Solvent | CDCl3 |
| 3 | Temperature | 298.0 |
| 4 | Number of Scans | 1024 |
| 5 | Receiver Gain | 101.0 |
| 6 | Relaxation Delay | 2.0000 |
| 7 | Pulse Width | 11.7800 |
| 8 | Spectrometer Frequency | 150.92 |
| 9 | Nucleus | 13C |

**S47**

Chemical Formula: $C_{11}H_{13}NO$

Exact Mass: 175.0997

Molecular Weight: 175.2310

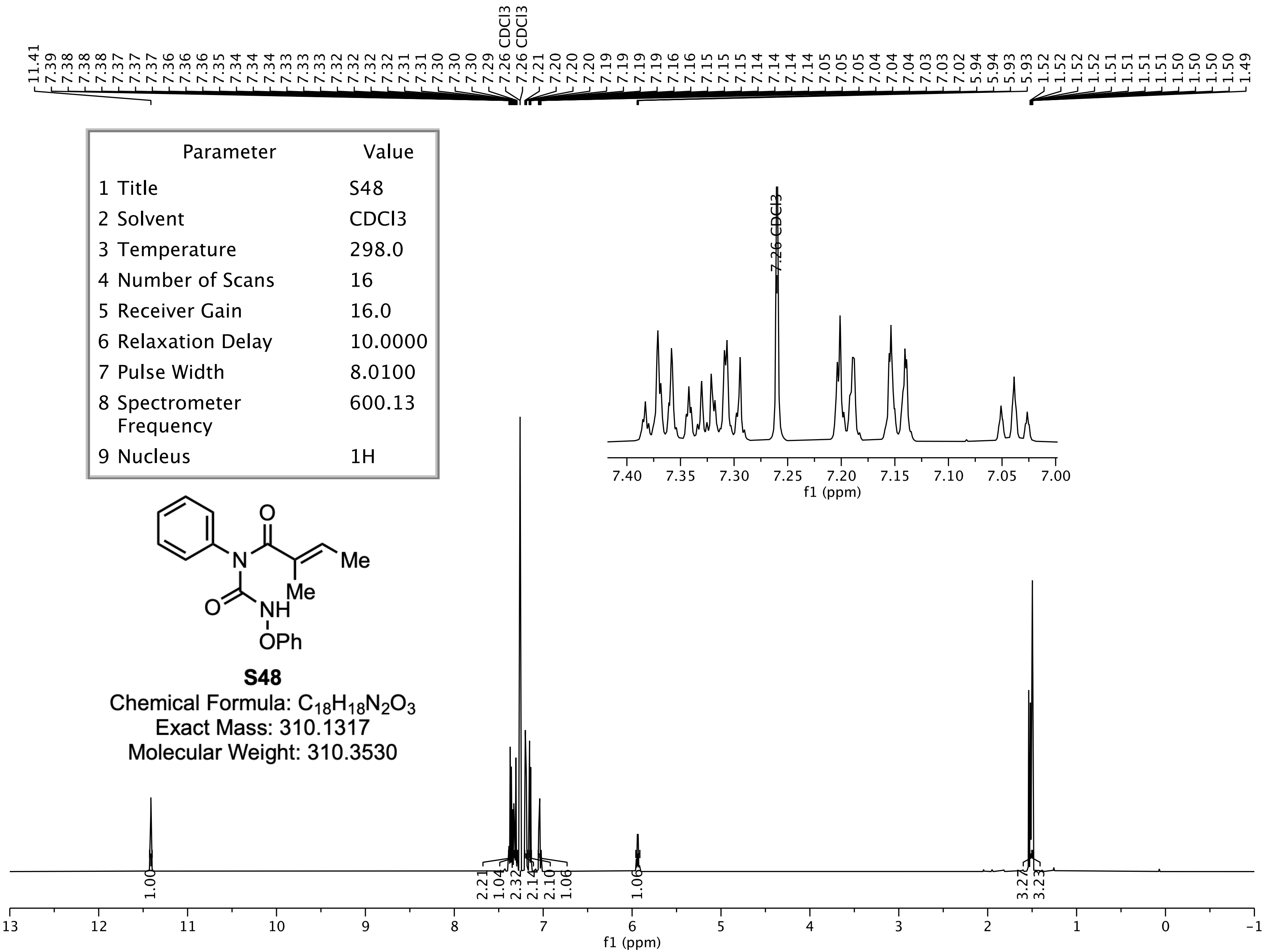


| Parameter | Value |
|---|---|
| 1 Title | S48 |
| 2 Solvent | CDCl3 |
| 3 Temperature | 298.0 |
| 4 Number of Scans | 16 |
| 5 Receiver Gain | 16.0 |
| 6 Relaxation Delay | 10.0000 |
| 7 Pulse Width | 8.0100 |
| 8 Spectrometer Frequency | 600.13 |
| 9 Nucleus | 1H |

**S48**

Chemical Formula: $C_{18}H_{18}N_2O_3$

Exact Mass: 310.1317

Molecular Weight: 310.3530

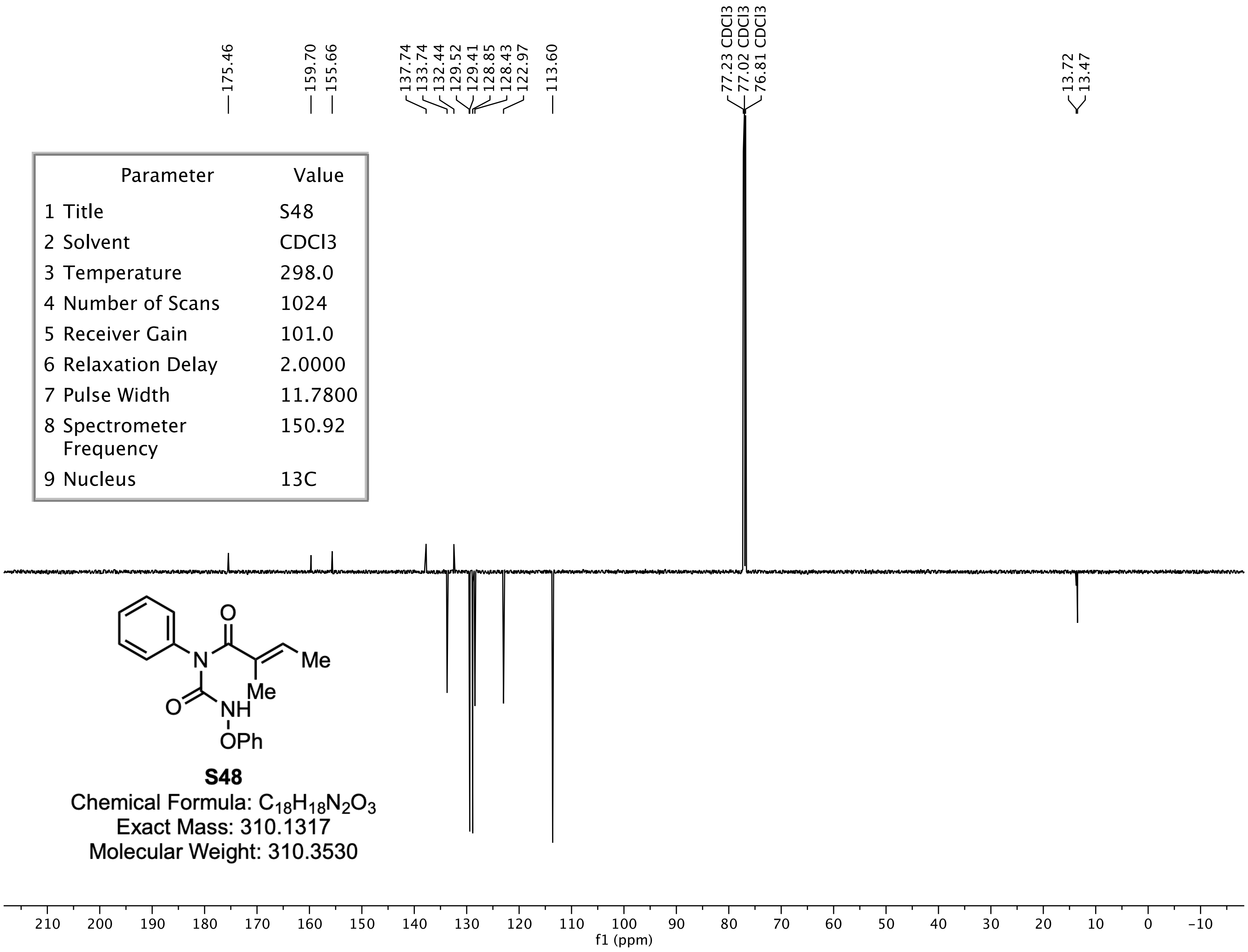


| Parameter | Value |
|---|---|
| 1 Title | S48 |
| 2 Solvent | CDCl3 |
| 3 Temperature | 298.0 |
| 4 Number of Scans | 1024 |
| 5 Receiver Gain | 101.0 |
| 6 Relaxation Delay | 2.0000 |
| 7 Pulse Width | 11.7800 |
| 8 Spectrometer Frequency | 150.92 |
| 9 Nucleus | 13C |

**S48**
Chemical Formula: $C_{18}H_{18}N_2O_3$
Exact Mass: 310.1317
Molecular Weight: 310.3530

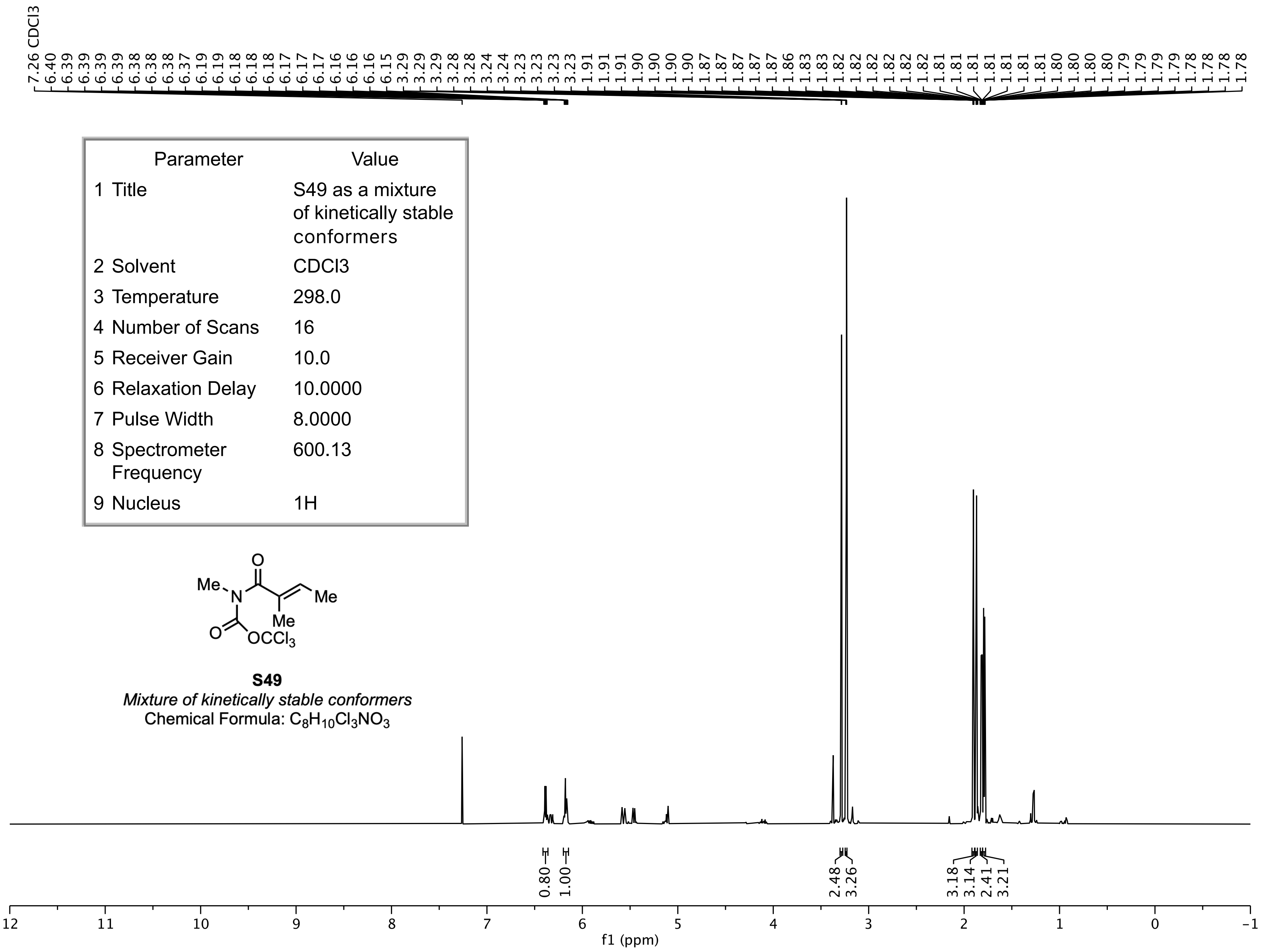


| Parameter | Value |
|---|---|
| 1 Title | S49 as a mixture of kinetically stable conformers |
| 2 Solvent | CDCl3 |
| 3 Temperature | 298.0 |
| 4 Number of Scans | 16 |
| 5 Receiver Gain | 10.0 |
| 6 Relaxation Delay | 10.0000 |
| 7 Pulse Width | 8.0000 |
| 8 Spectrometer Frequency | 600.13 |
| 9 Nucleus | 1H |

**S49**

*Mixture of kinetically stable conformers*

Chemical Formula: $C_8H_{10}Cl_3NO_3$

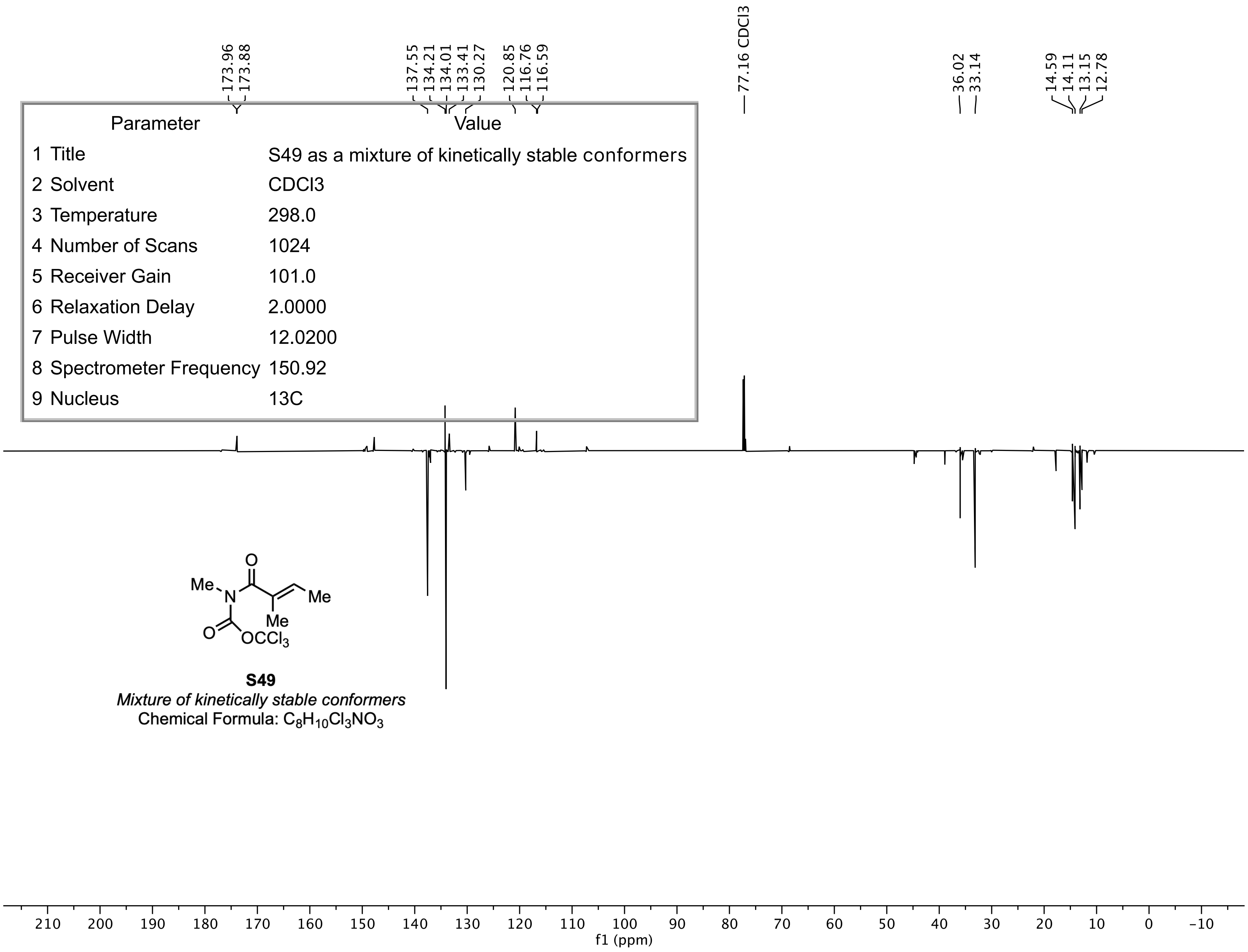


| | Parameter | Value |
|---|---|---|
| 1 | Title | S49 as a mixture of kinetically stable conformers |
| 2 | Solvent | CDCl3 |
| 3 | Temperature | 298.0 |
| 4 | Number of Scans | 1024 |
| 5 | Receiver Gain | 101.0 |
| 6 | Relaxation Delay | 2.0000 |
| 7 | Pulse Width | 12.0200 |
| 8 | Spectrometer Frequency | 150.92 |
| 9 | Nucleus | 13C |

**S49**

*Mixture of kinetically stable conformers*

Chemical Formula: $C_8H_{10}Cl_3NO_3$

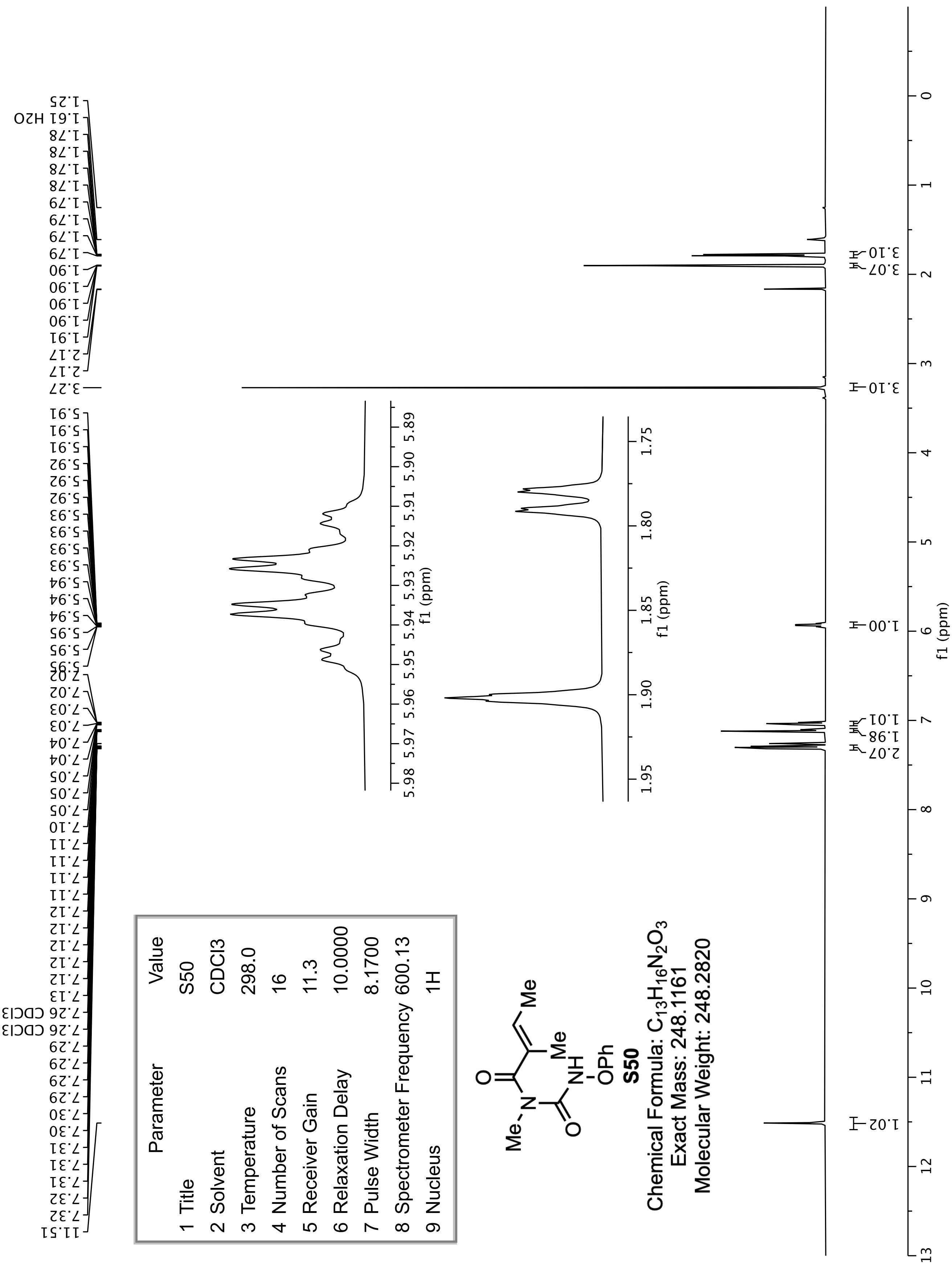
Parameter Value
1 Title S50
2 Solvent CDCl3
3 Temperature 298.0
4 Number of Scans 16
5 Receiver Gain 11.3
6 Relaxation Delay 10.0000
7 Pulse Width 8.1700
8 Spectrometer Frequency 600.13
9 Nucleus 1H
S50
Chemical Formula: C13H16N2O3
Exact Mass: 248.1161
Molecular Weight: 248.2820
f1 (ppm)

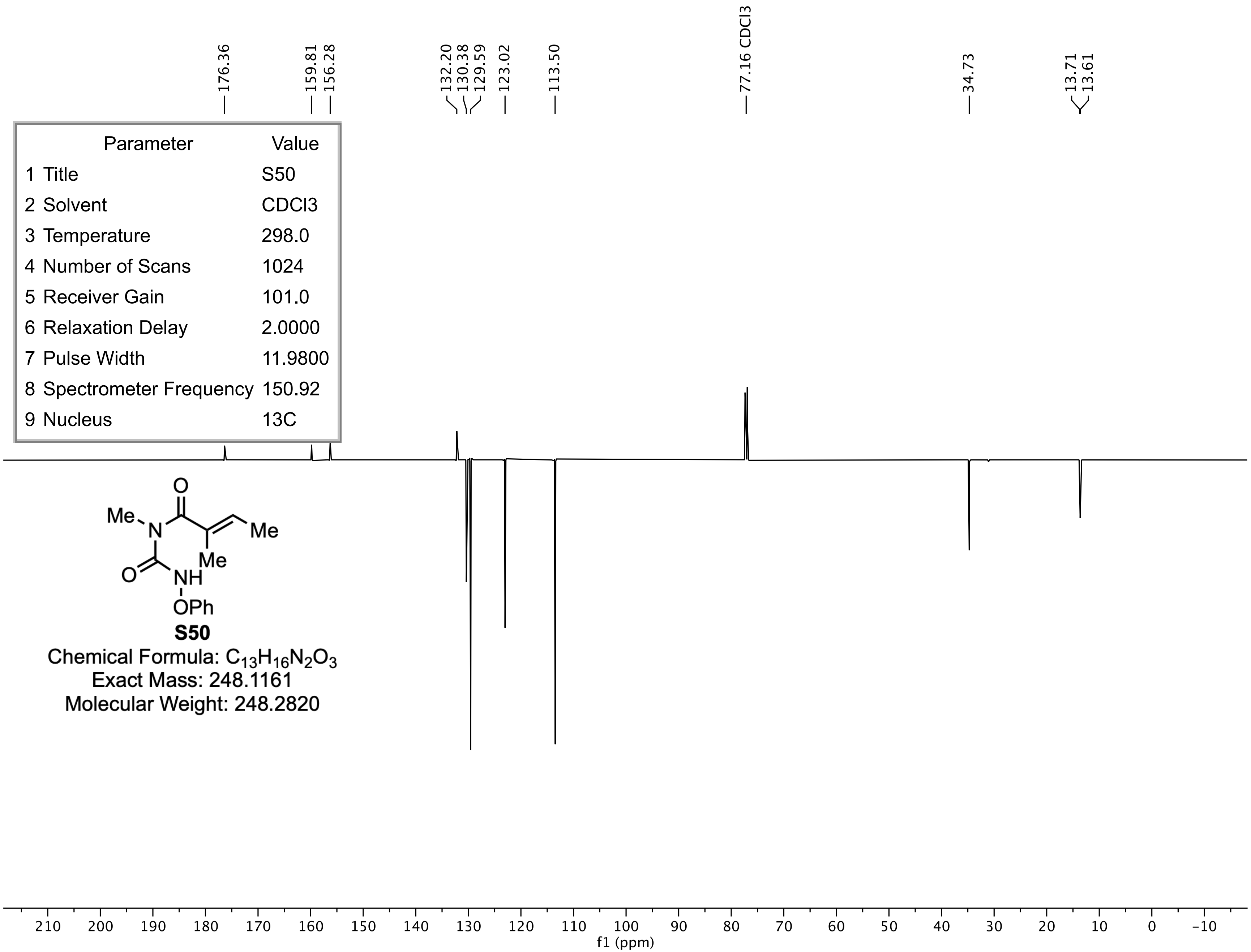


| Parameter | Value |
|---|---|
| 1 Title | S50 |
| 2 Solvent | CDCl3 |
| 3 Temperature | 298.0 |
| 4 Number of Scans | 1024 |
| 5 Receiver Gain | 101.0 |
| 6 Relaxation Delay | 2.0000 |
| 7 Pulse Width | 11.9800 |
| 8 Spectrometer Frequency | 150.92 |
| 9 Nucleus | 13C |

**S50**

Chemical Formula: $C_{13}H_{16}N_2O_3$

Exact Mass: 248.1161

Molecular Weight: 248.2820

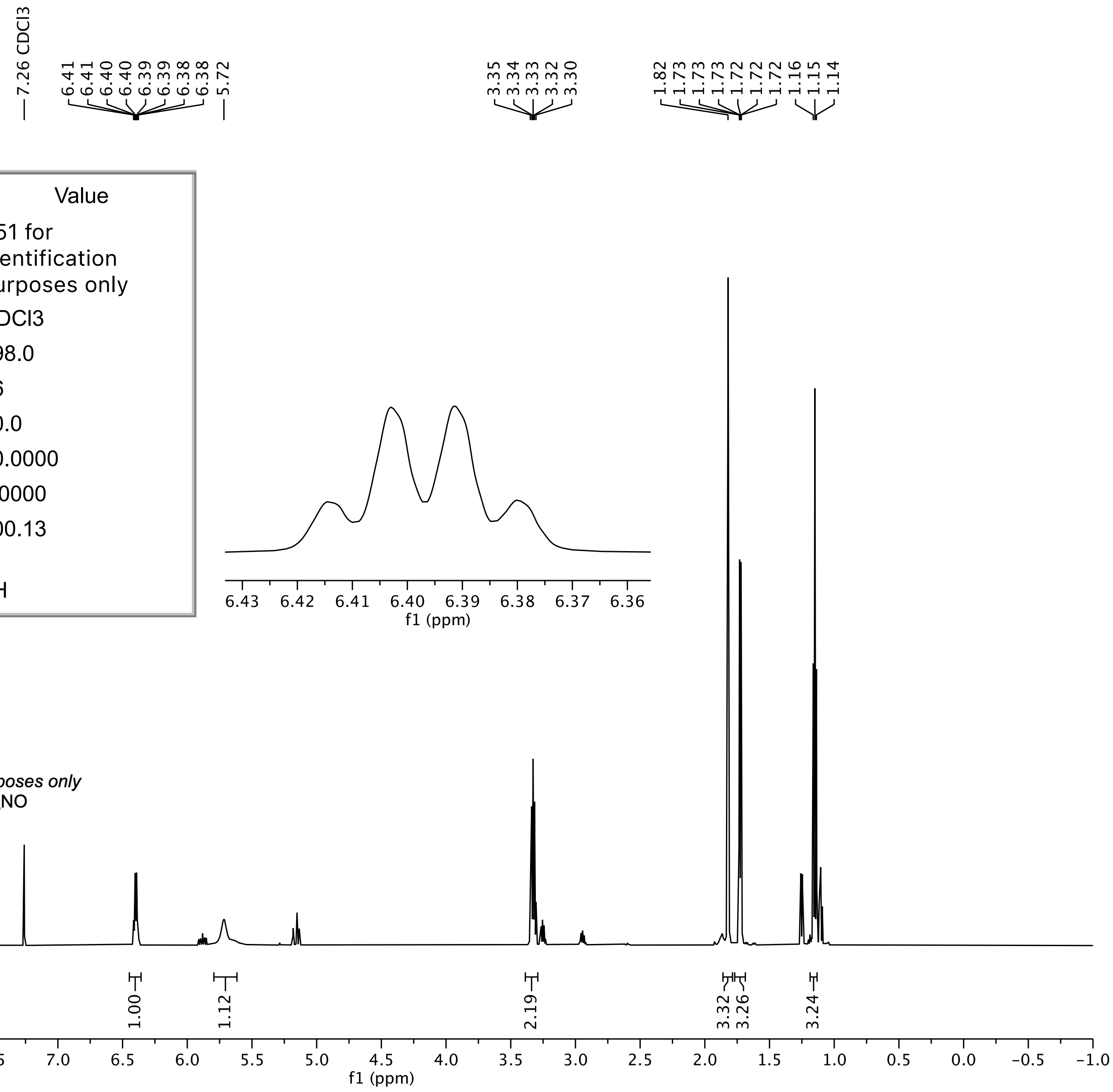


| Parameter | Value |
|---|---|
| 1 Title | S51 for identification purposes only |
| 2 Solvent | CDCl3 |
| 3 Temperature | 298.0 |
| 4 Number of Scans | 16 |
| 5 Receiver Gain | 10.0 |
| 6 Relaxation Delay | 10.0000 |
| 7 Pulse Width | 8.0000 |
| 8 Spectrometer Frequency | 600.13 |
| 9 Nucleus | 1H |

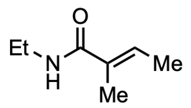


**S51**
*85% purity; for identification purposes only*
Chemical Formula: $C_7H_{13}NO$

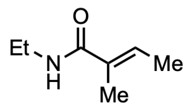

**S51**
*85% purity; for identification purposes only*
Chemical Formula: $C_7H_{13}NO$

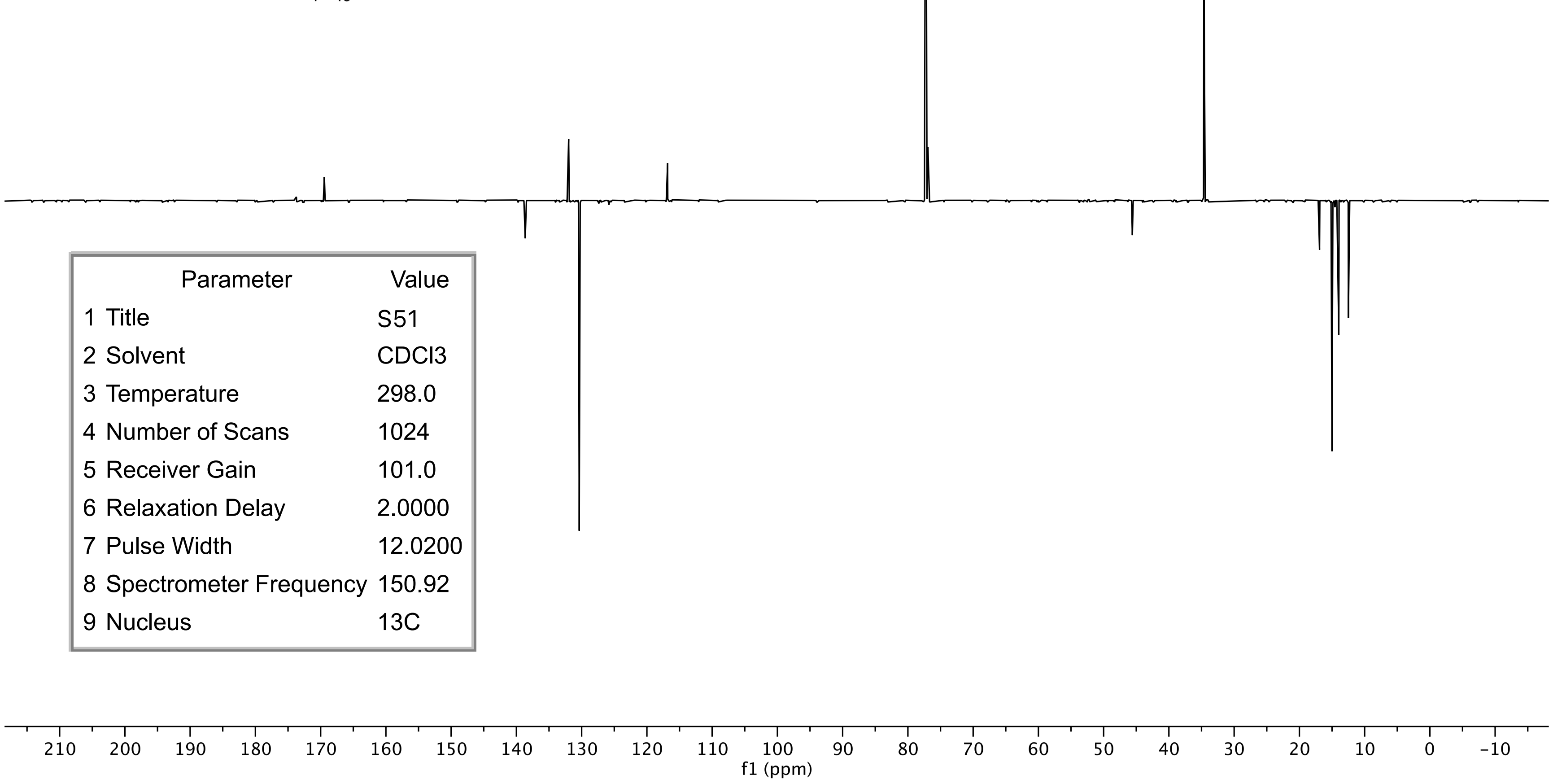


| | Parameter | Value |
|---|---|---|
| 1 | Title | S51 |
| 2 | Solvent | CDCl3 |
| 3 | Temperature | 298.0 |
| 4 | Number of Scans | 1024 |
| 5 | Receiver Gain | 101.0 |
| 6 | Relaxation Delay | 2.0000 |
| 7 | Pulse Width | 12.0200 |
| 8 | Spectrometer Frequency | 150.92 |
| 9 | Nucleus | 13C |

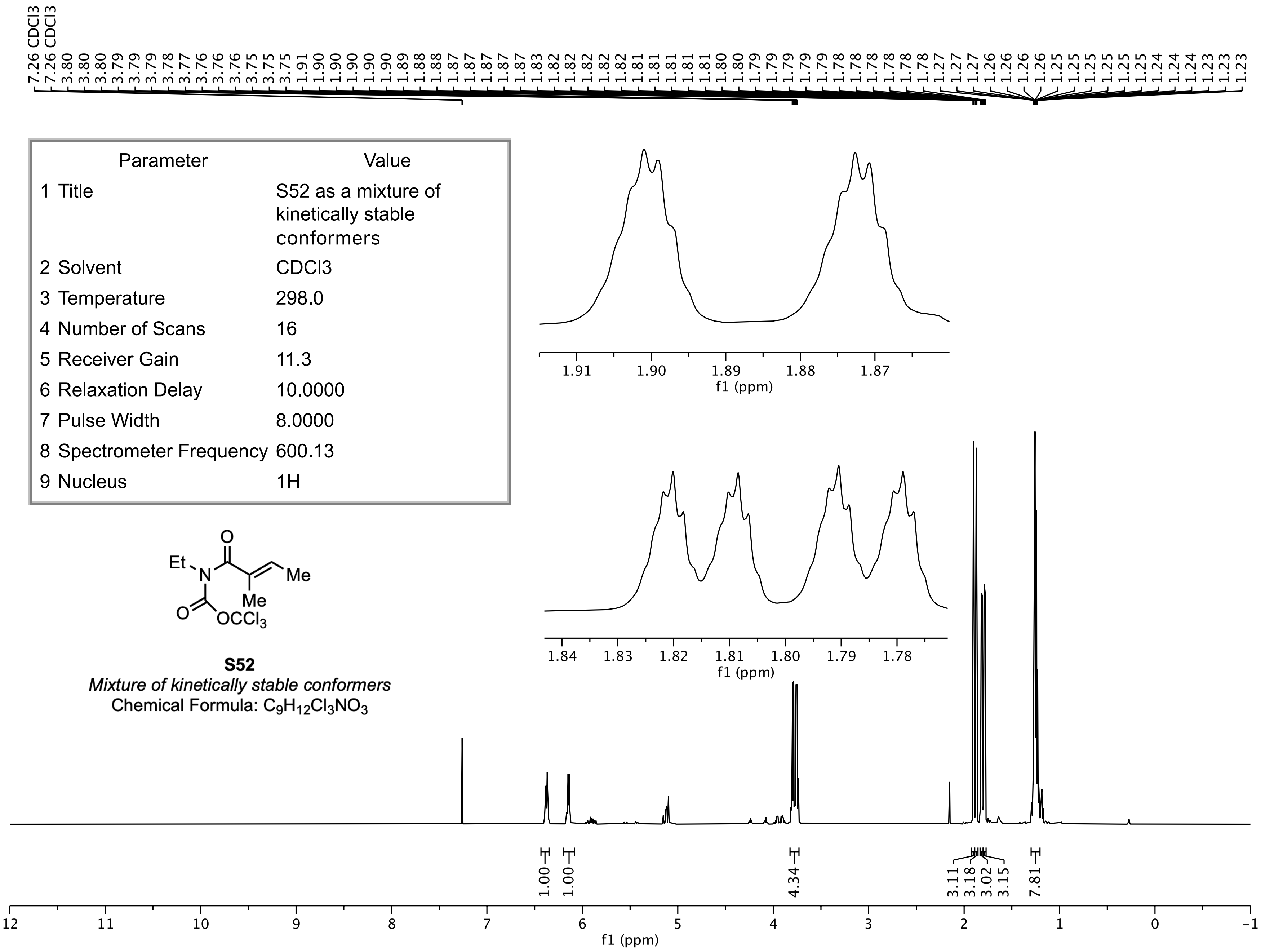


| Parameter | Value |
| --- | --- |
| 1 Title | S52 as a mixture of kinetically stable conformers |
| 2 Solvent | CDCl3 |
| 3 Temperature | 298.0 |
| 4 Number of Scans | 16 |
| 5 Receiver Gain | 11.3 |
| 6 Relaxation Delay | 10.0000 |
| 7 Pulse Width | 8.0000 |
| 8 Spectrometer Frequency | 600.13 |
| 9 Nucleus | 1H |

**S52**

*Mixture of kinetically stable conformers*

Chemical Formula: $C_9H_{12}Cl_3NO_3$

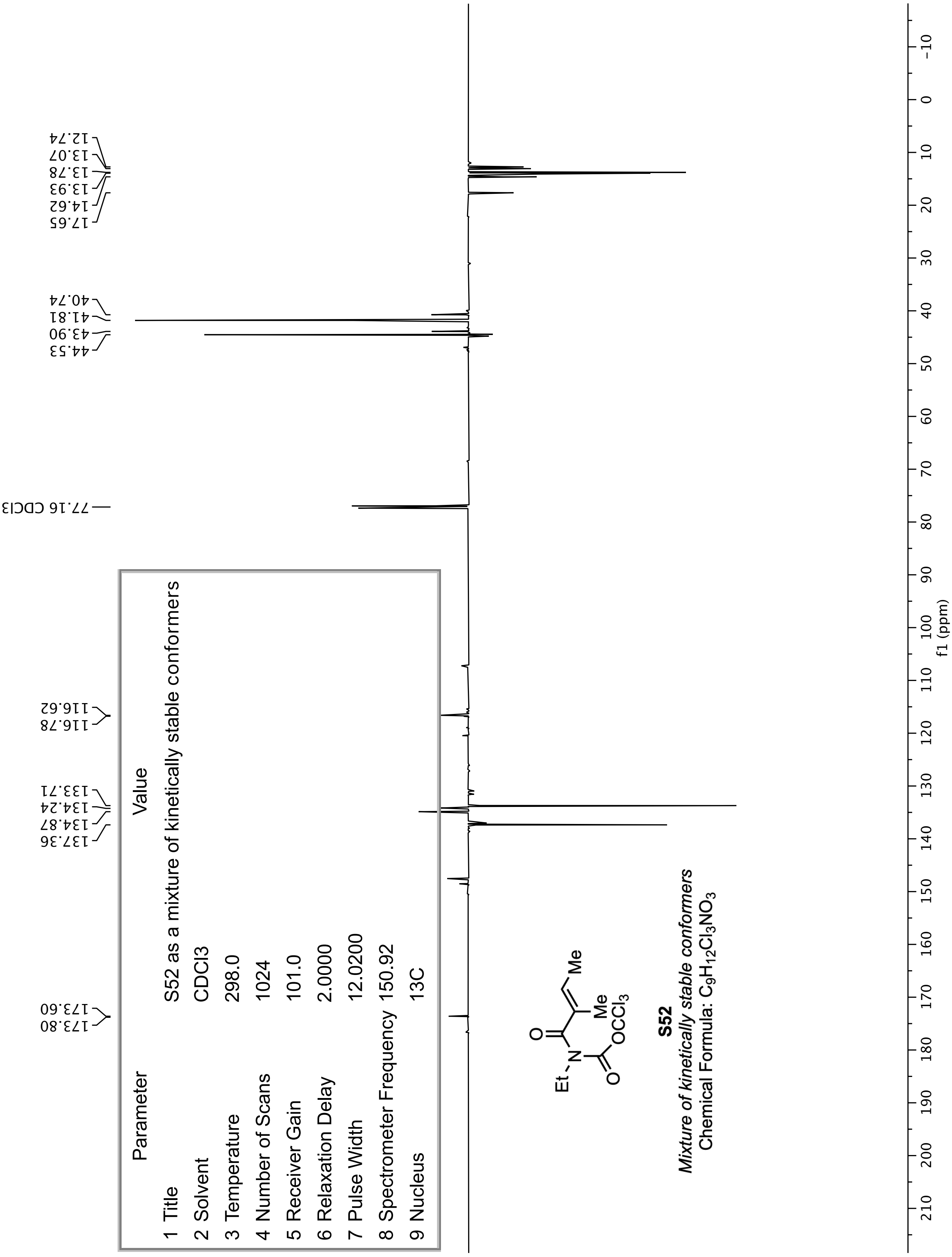
173.80
173.60
137.36
134.87
134.24
133.71
116.78
116.62
77.16 CDCl3
44.53
43.90
41.81
40.74
17.65
14.62
13.93
13.78
13.07
12.74
Parameter | Value
1 Title | S52 as a mixture of kinetically stable conformers
2 Solvent | CDCl3
3 Temperature | 298.0
4 Number of Scans | 1024
5 Receiver Gain | 101.0
6 Relaxation Delay | 2.0000
7 Pulse Width | 12.0200
8 Spectrometer Frequency | 150.92
9 Nucleus | 13C
Et
N
O
O
OCCl3
Me
Me
S52
Mixture of kinetically stable conformers
Chemical Formula: C9H12Cl3NO3
f1 (ppm)

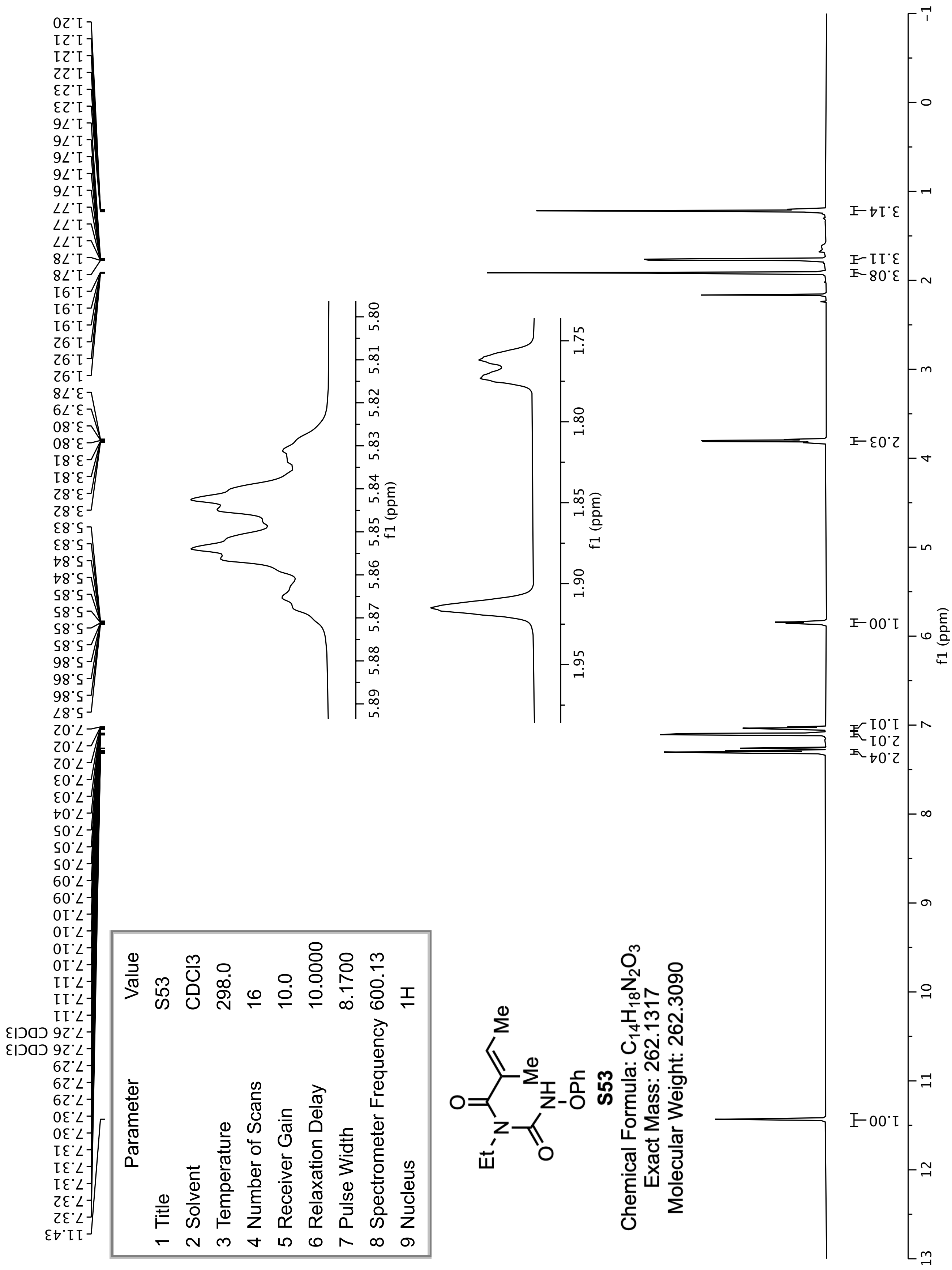
Parameter Value
1 Title S53
2 Solvent CDCl3
3 Temperature 298.0
4 Number of Scans 16
5 Receiver Gain 10.0
6 Relaxation Delay 10.0000
7 Pulse Width 8.1700
8 Spectrometer Frequency 600.13
9 Nucleus 1H
S53
Chemical Formula: C14H18N2O3
Exact Mass: 262.1317
Molecular Weight: 262.3090
f1 (ppm)

**S53**

Chemical Formula: $C_{14}H_{18}N_2O_3$
Exact Mass: 262.1317
Molecular Weight: 262.3090

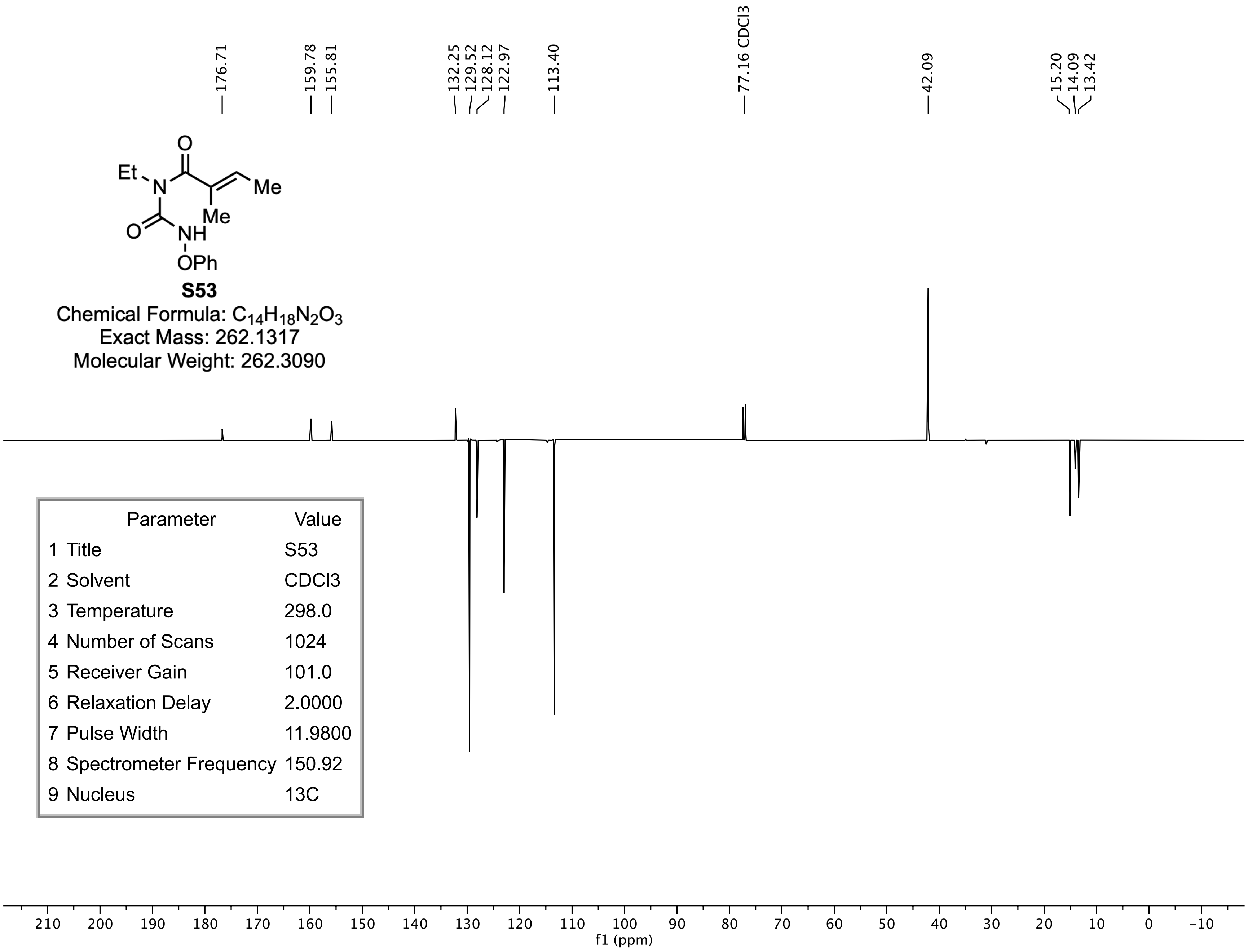


| | Parameter | Value |
|---|---|---|
| 1 | Title | S53 |
| 2 | Solvent | CDCl3 |
| 3 | Temperature | 298.0 |
| 4 | Number of Scans | 1024 |
| 5 | Receiver Gain | 101.0 |
| 6 | Relaxation Delay | 2.0000 |
| 7 | Pulse Width | 11.9800 |
| 8 | Spectrometer Frequency | 150.92 |
| 9 | Nucleus | 13C |

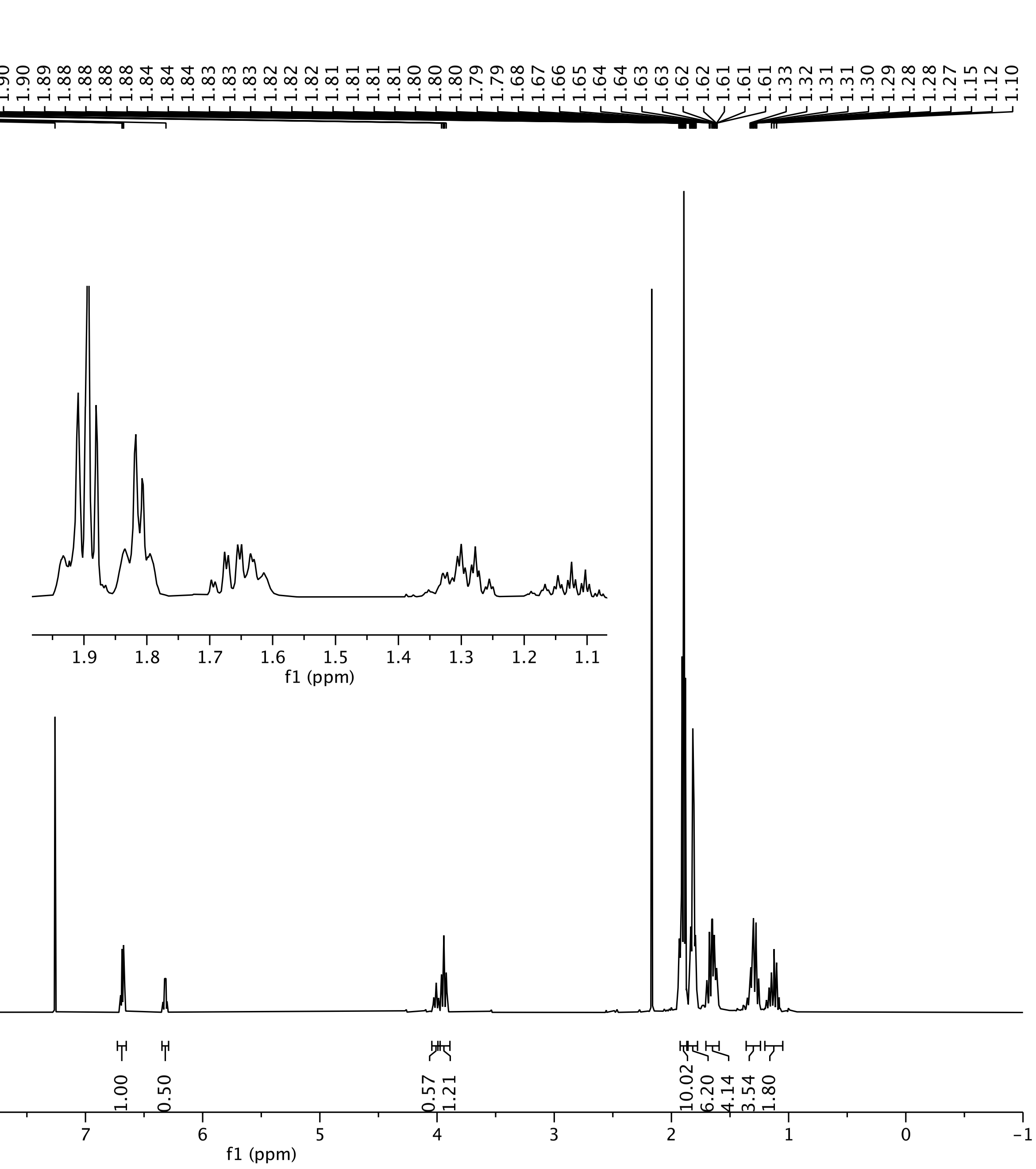


| Parameter | Value |
|---|---|
| 1 Title | S54 as a mixture of kinetically stable conformers |
| 2 Solvent | CDCl3 |
| 3 Temperature | 298.0 |
| 4 Number of Scans | 16 |
| 5 Receiver Gain | 11.3 |
| 6 Relaxation Delay | 10.0000 |
| 7 Pulse Width | 8.0000 |
| 8 Spectrometer Frequency | 600.13 |
| 9 Nucleus | 1H |

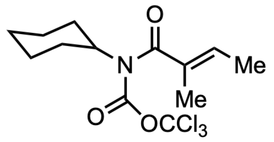


**S54**

*Mixture of kinetically stable conformers*

Chemical Formula: $C_{13}H_{18}Cl_3NO_3$

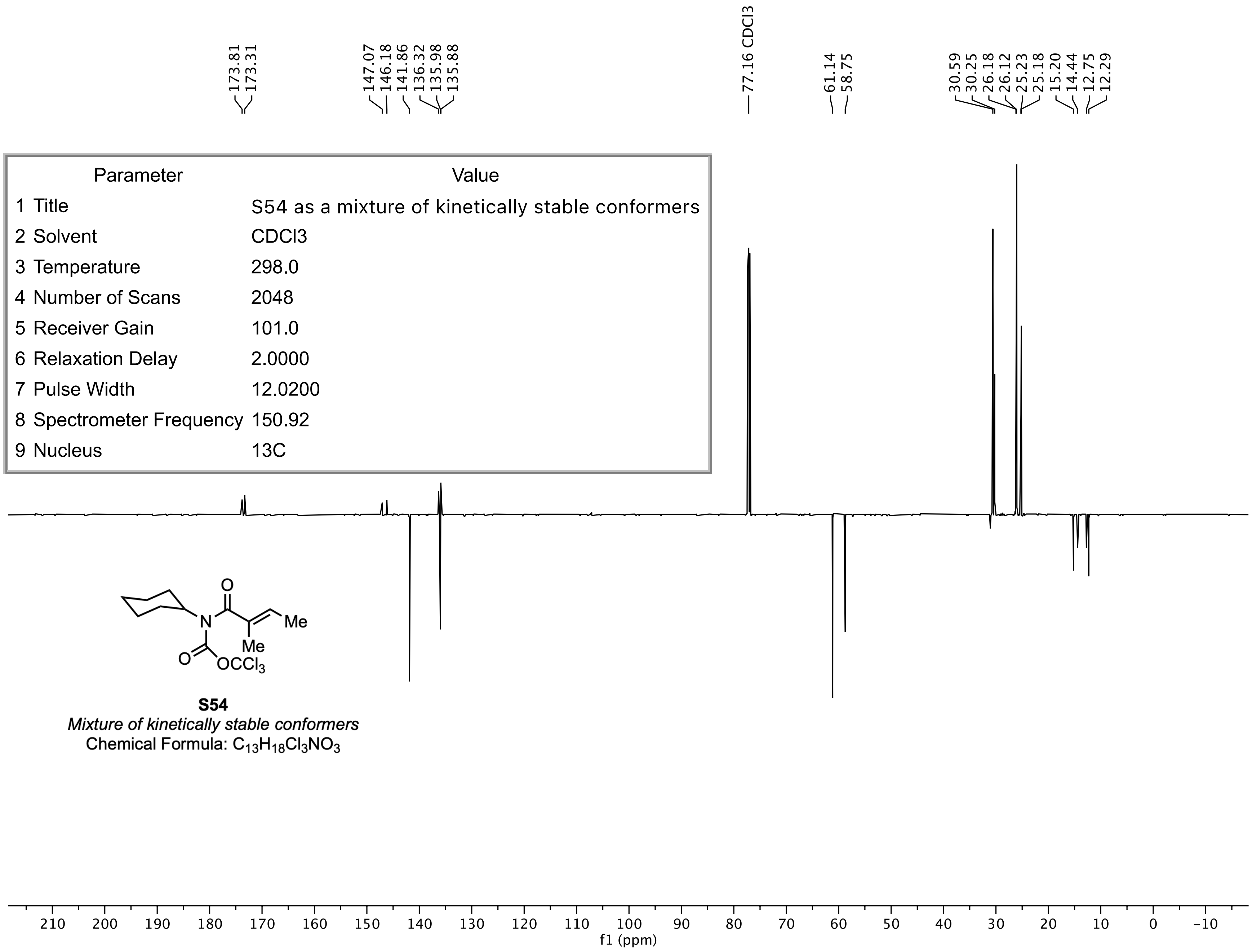


| Parameter | Value |
|---|---|
| 1 Title | S54 as a mixture of kinetically stable conformers |
| 2 Solvent | CDCl3 |
| 3 Temperature | 298.0 |
| 4 Number of Scans | 2048 |
| 5 Receiver Gain | 101.0 |
| 6 Relaxation Delay | 2.0000 |
| 7 Pulse Width | 12.0200 |
| 8 Spectrometer Frequency | 150.92 |
| 9 Nucleus | 13C |

**S54**

*Mixture of kinetically stable conformers*

Chemical Formula: $C_{13}H_{18}Cl_3NO_3$

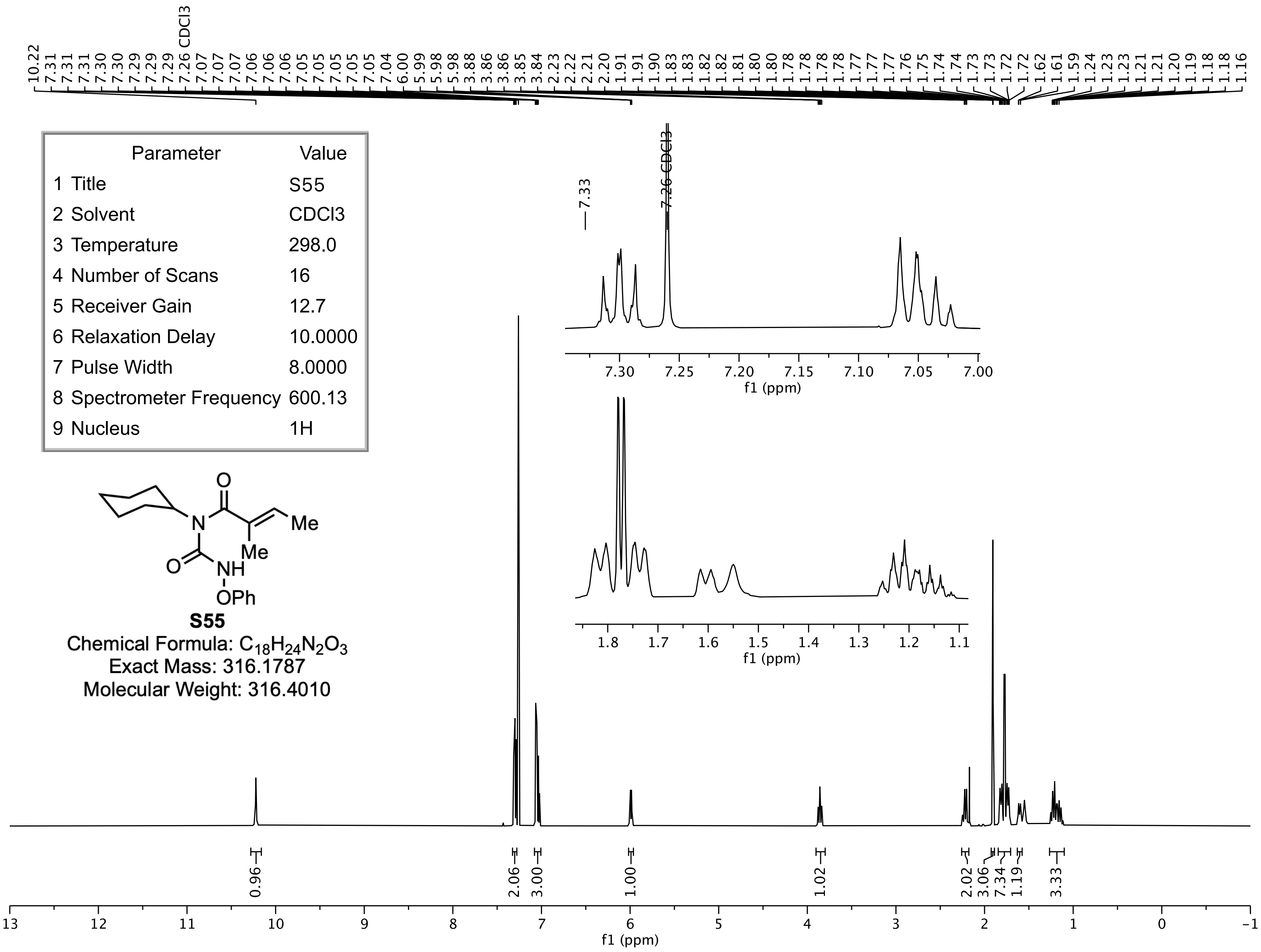


| | Parameter | Value |
|---|---|---|
| 1 | Title | S55 |
| 2 | Solvent | CDCl3 |
| 3 | Temperature | 298.0 |
| 4 | Number of Scans | 16 |
| 5 | Receiver Gain | 12.7 |
| 6 | Relaxation Delay | 10.0000 |
| 7 | Pulse Width | 8.0000 |
| 8 | Spectrometer Frequency | 600.13 |
| 9 | Nucleus | 1H |

**S55**

Chemical Formula: $C_{18}H_{24}N_2O_3$
Exact Mass: 316.1787
Molecular Weight: 316.4010

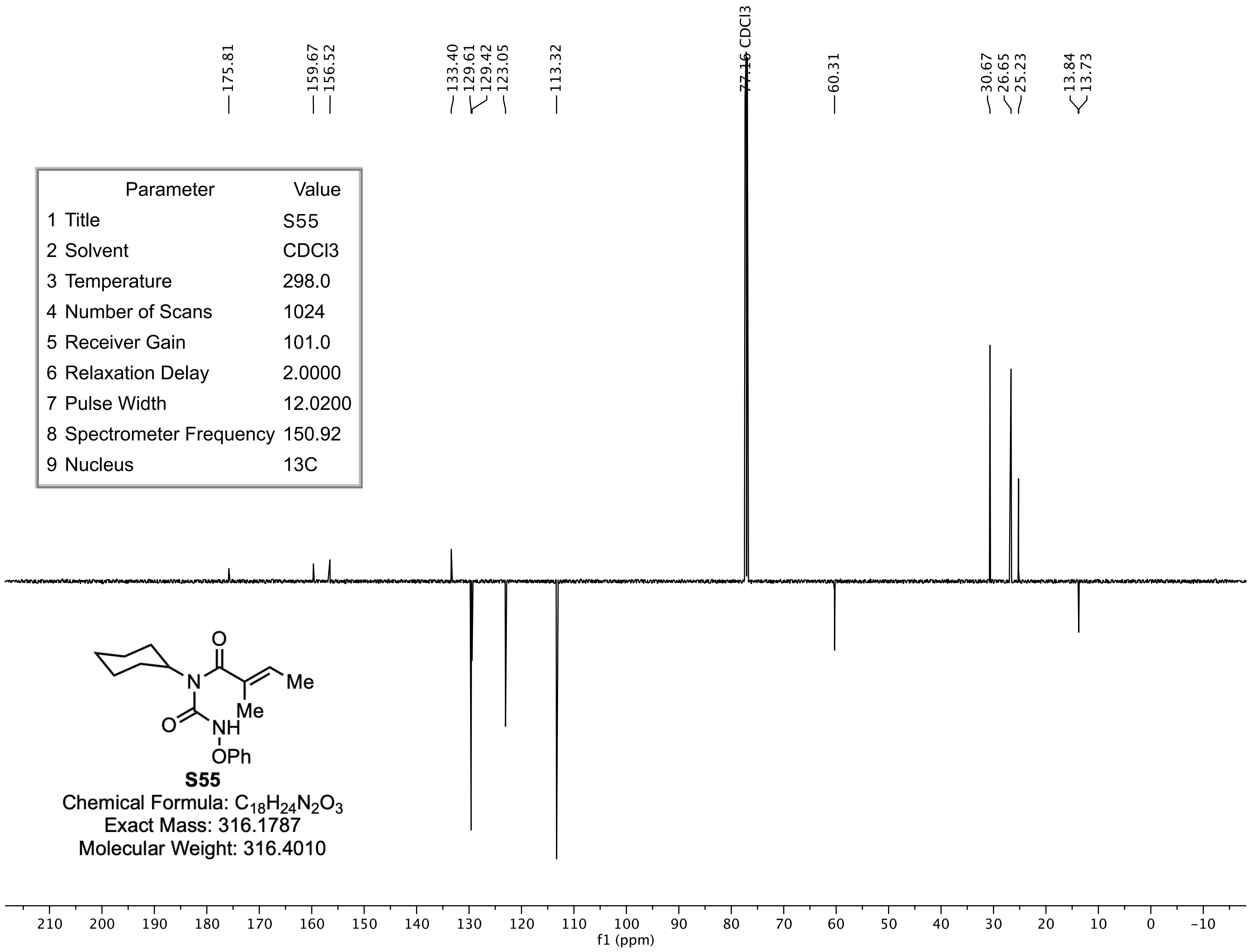


| Parameter | Value |
| --- | --- |
| 1 Title | S55 |
| 2 Solvent | CDCl3 |
| 3 Temperature | 298.0 |
| 4 Number of Scans | 1024 |
| 5 Receiver Gain | 101.0 |
| 6 Relaxation Delay | 2.0000 |
| 7 Pulse Width | 12.0200 |
| 8 Spectrometer Frequency | 150.92 |
| 9 Nucleus | 13C |



**S55**

Chemical Formula: $C_{18}H_{24}N_2O_3$
Exact Mass: 316.1787
Molecular Weight: 316.4010

| Parameter | Value |
|---|---|
| 1 Title | S56 |
| 2 Solvent | CDCl3 |
| 3 Temperature | 298.0 |
| 4 Number of Scans | 16 |
| 5 Receiver Gain | 64.0 |
| 6 Relaxation Delay | 10.0000 |
| 7 Pulse Width | 12.0000 |
| 8 Spectrometer Frequency | 600.32 |
| 9 Nucleus | 1H |

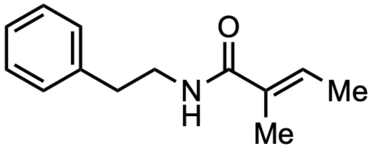


**S56**

Chemical Formula: $C_{13}H_{17}NO$
Exact Mass: 203.1310
Molecular Weight: 203.2850

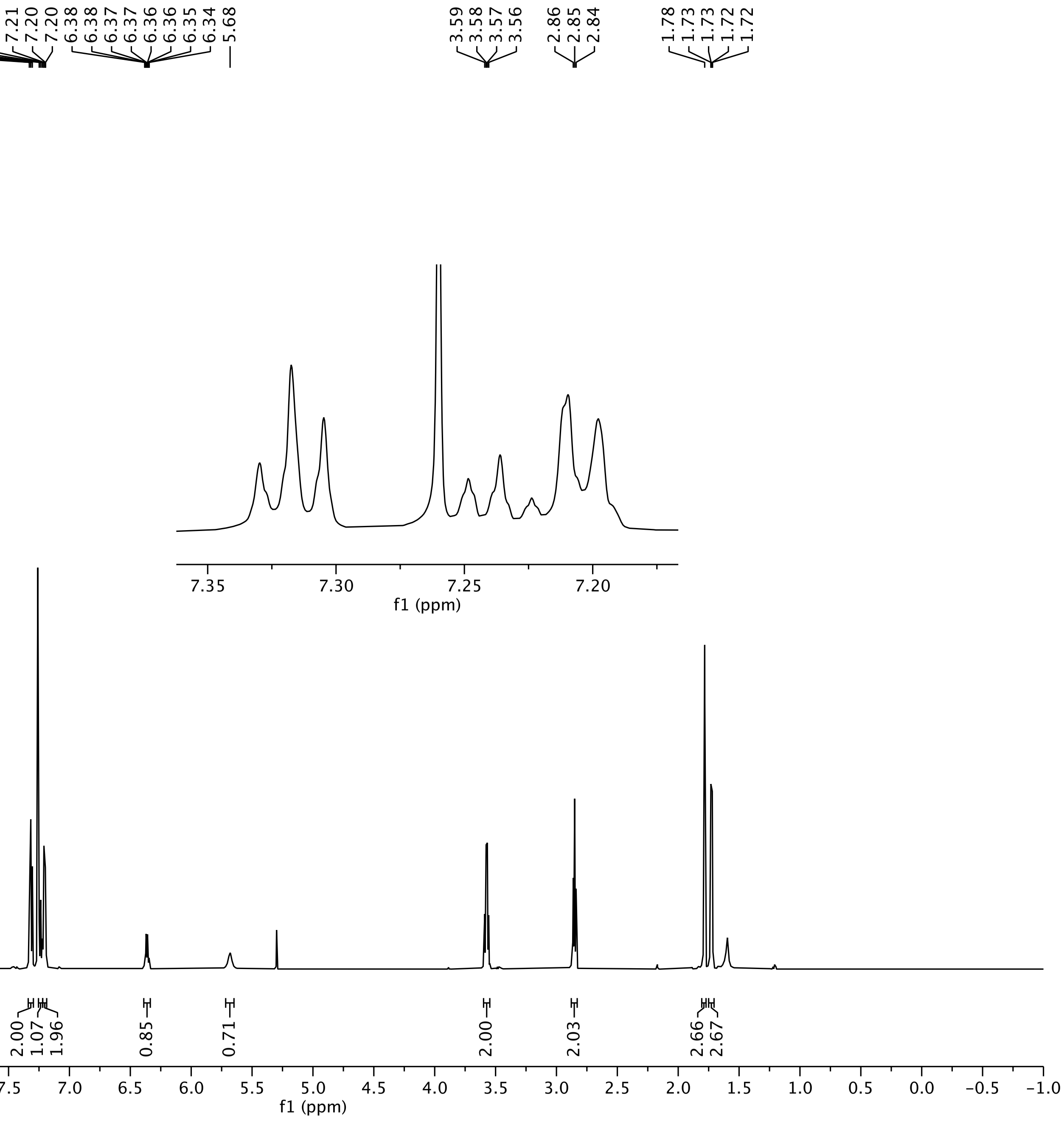

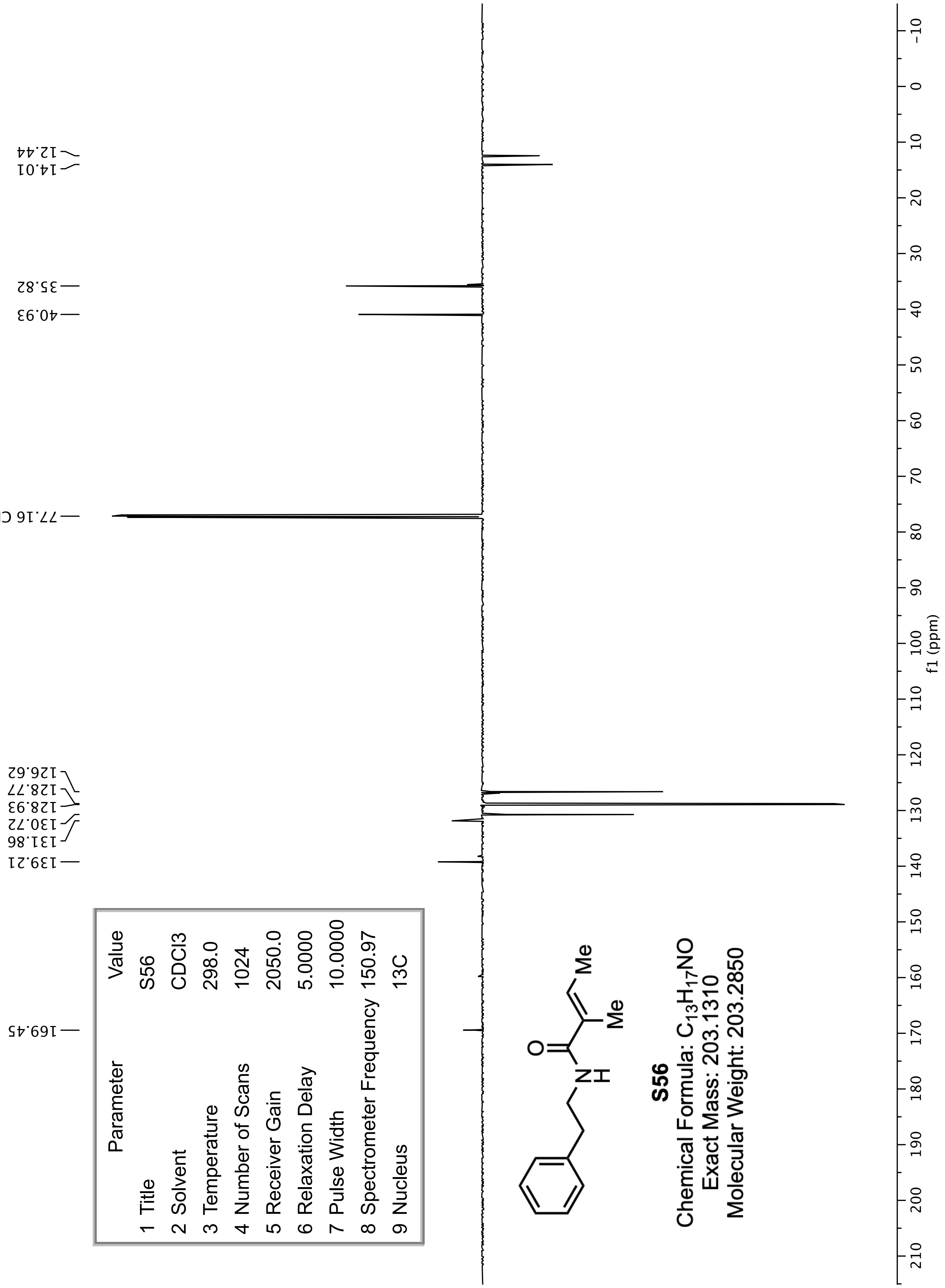


| | Parameter | Value |
|---|---|---|
| 1 | Title | S56 |
| 2 | Solvent | CDCl3 |
| 3 | Temperature | 298.0 |
| 4 | Number of Scans | 1024 |
| 5 | Receiver Gain | 2050.0 |
| 6 | Relaxation Delay | 5.0000 |
| 7 | Pulse Width | 10.0000 |
| 8 | Spectrometer Frequency | 150.97 |
| 9 | Nucleus | 13C |

**S56**

Chemical Formula: $C_{13}H_{17}NO$

Exact Mass: 203.1310

Molecular Weight: 203.2850

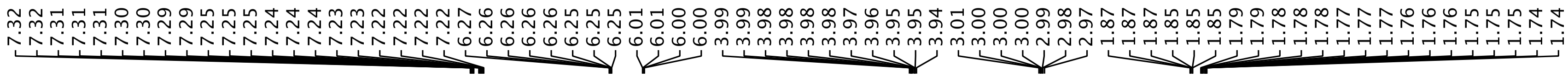


| Parameter | Value |
|---|---|
| 1 Title | S57 as a mixture of kinetically stable conformers |
| 2 Solvent | CDCl3 |
| 3 Temperature | 298.0 |
| 4 Number of Scans | 16 |
| 5 Receiver Gain | 32.0 |
| 6 Relaxation Delay | 10.0000 |
| 7 Pulse Width | 12.0000 |
| 8 Spectrometer Frequency | 600.32 |
| 9 Nucleus | 1H |

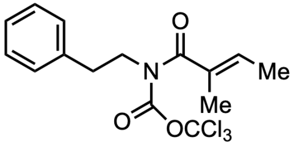


**S57**
*Mixture of kinetically stable conformers*
Chemical Formula: $C_{15}H_{16}Cl_3NO_3$

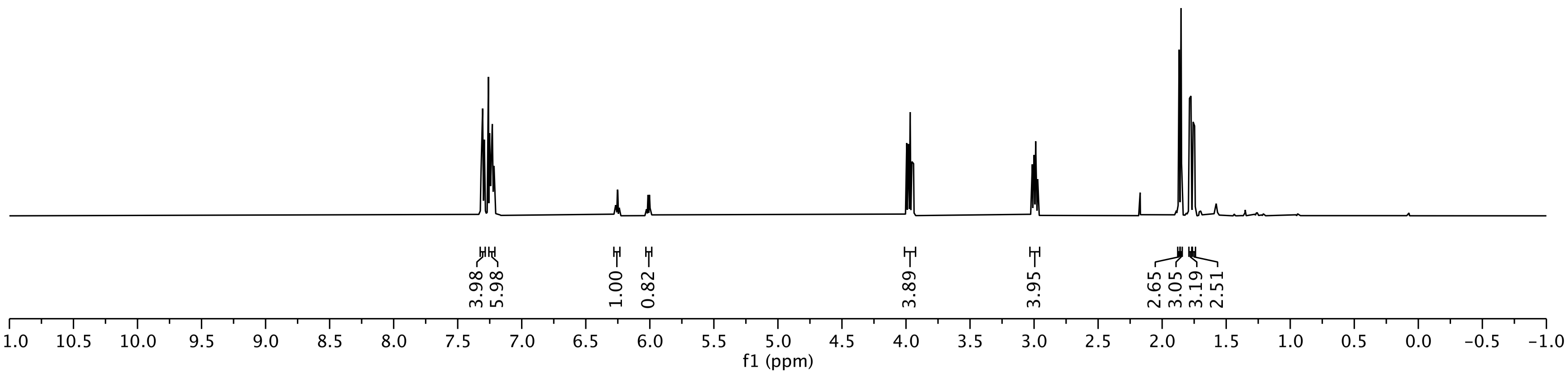

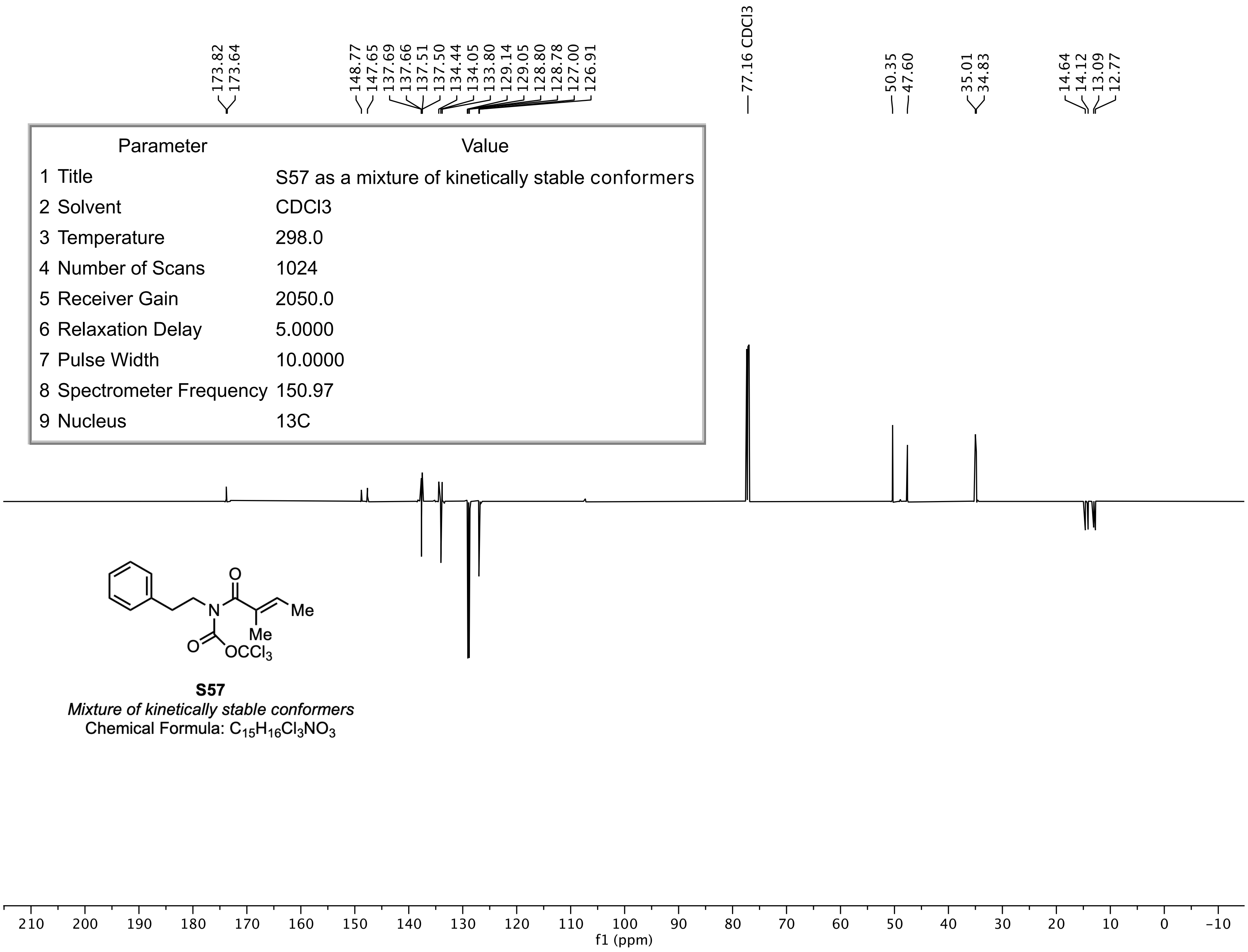


| Parameter | Value |
|---|---|
| 1 Title | S57 as a mixture of kinetically stable conformers |
| 2 Solvent | CDCl3 |
| 3 Temperature | 298.0 |
| 4 Number of Scans | 1024 |
| 5 Receiver Gain | 2050.0 |
| 6 Relaxation Delay | 5.0000 |
| 7 Pulse Width | 10.0000 |
| 8 Spectrometer Frequency | 150.97 |
| 9 Nucleus | 13C |

**S57**

*Mixture of kinetically stable conformers*

Chemical Formula: $C_{15}H_{16}Cl_3NO_3$

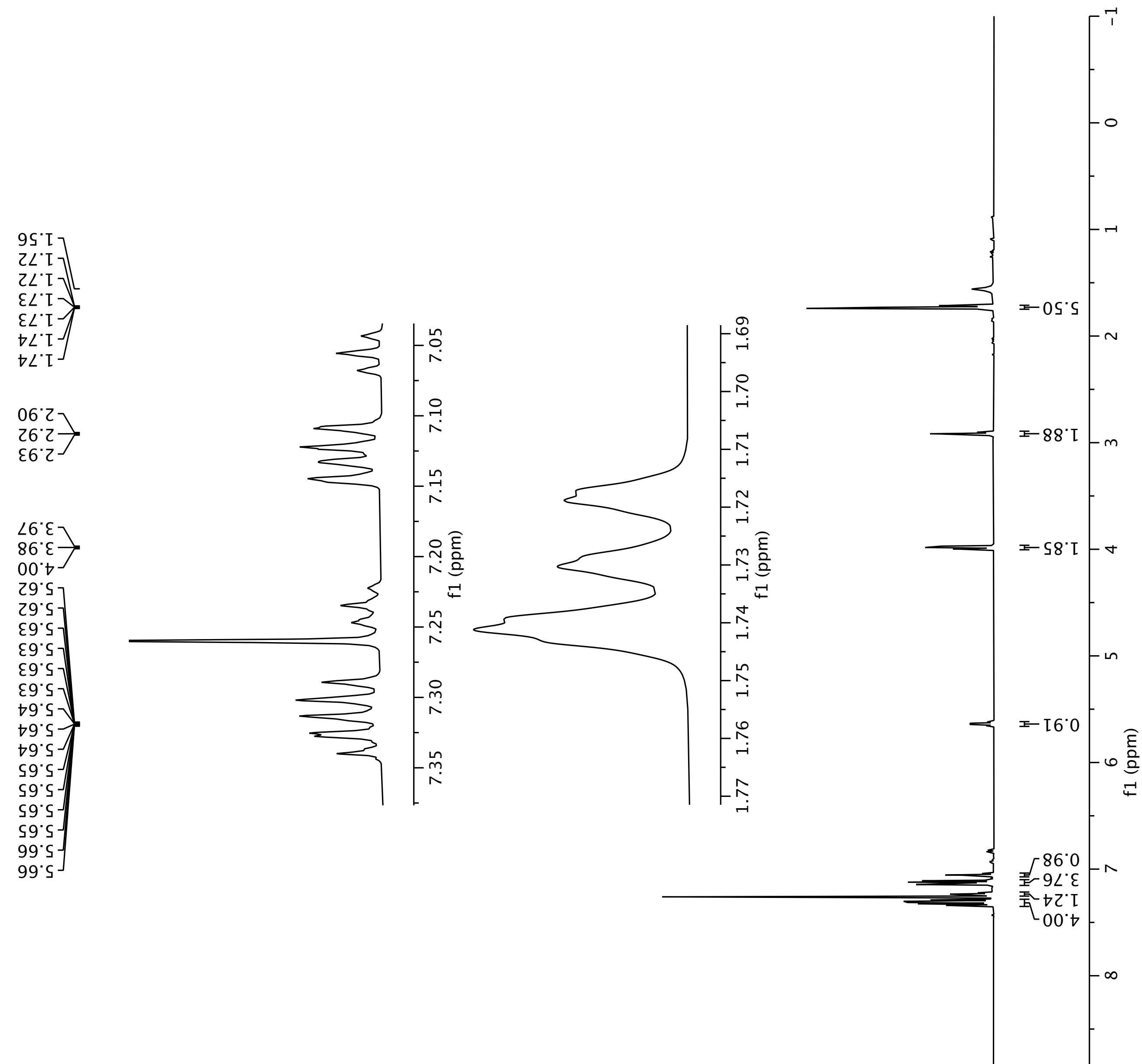


| Parameter | Value |
|---|---|
| 1 Title | S58 |
| 2 Solvent | CDCl3 |
| 3 Temperature | 298.0 |
| 4 Number of Scans | 16 |
| 5 Receiver Gain | 12.7 |
| 6 Relaxation Delay | 10.0000 |
| 7 Pulse Width | 8.0000 |
| 8 Spectrometer Frequency | 600.13 |
| 9 Nucleus | 1H |

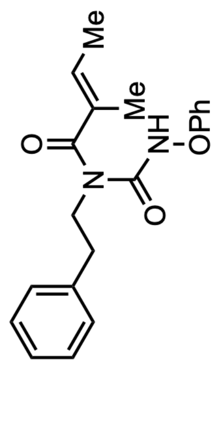

**S58**

Chemical Formula: $C_{20}H_{22}N_2O_3$

Exact Mass: 338.1630

Molecular Weight: 338.4070

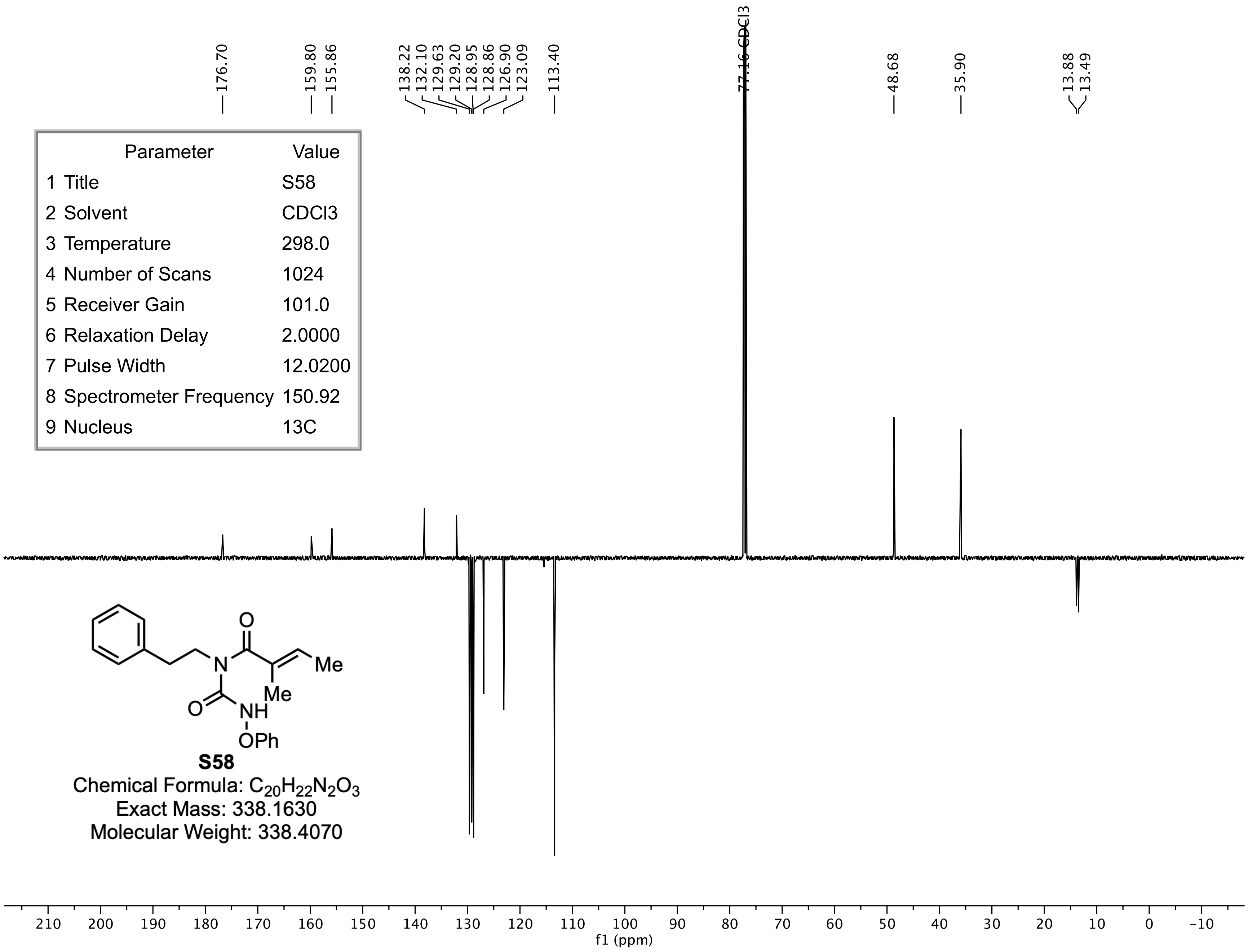


| Parameter | Value |
|---|---|
| 1 Title | S58 |
| 2 Solvent | CDCl3 |
| 3 Temperature | 298.0 |
| 4 Number of Scans | 1024 |
| 5 Receiver Gain | 101.0 |
| 6 Relaxation Delay | 2.0000 |
| 7 Pulse Width | 12.0200 |
| 8 Spectrometer Frequency | 150.92 |
| 9 Nucleus | 13C |

**S58**

Chemical Formula: $C_{20}H_{22}N_2O_3$

Exact Mass: 338.1630

Molecular Weight: 338.4070

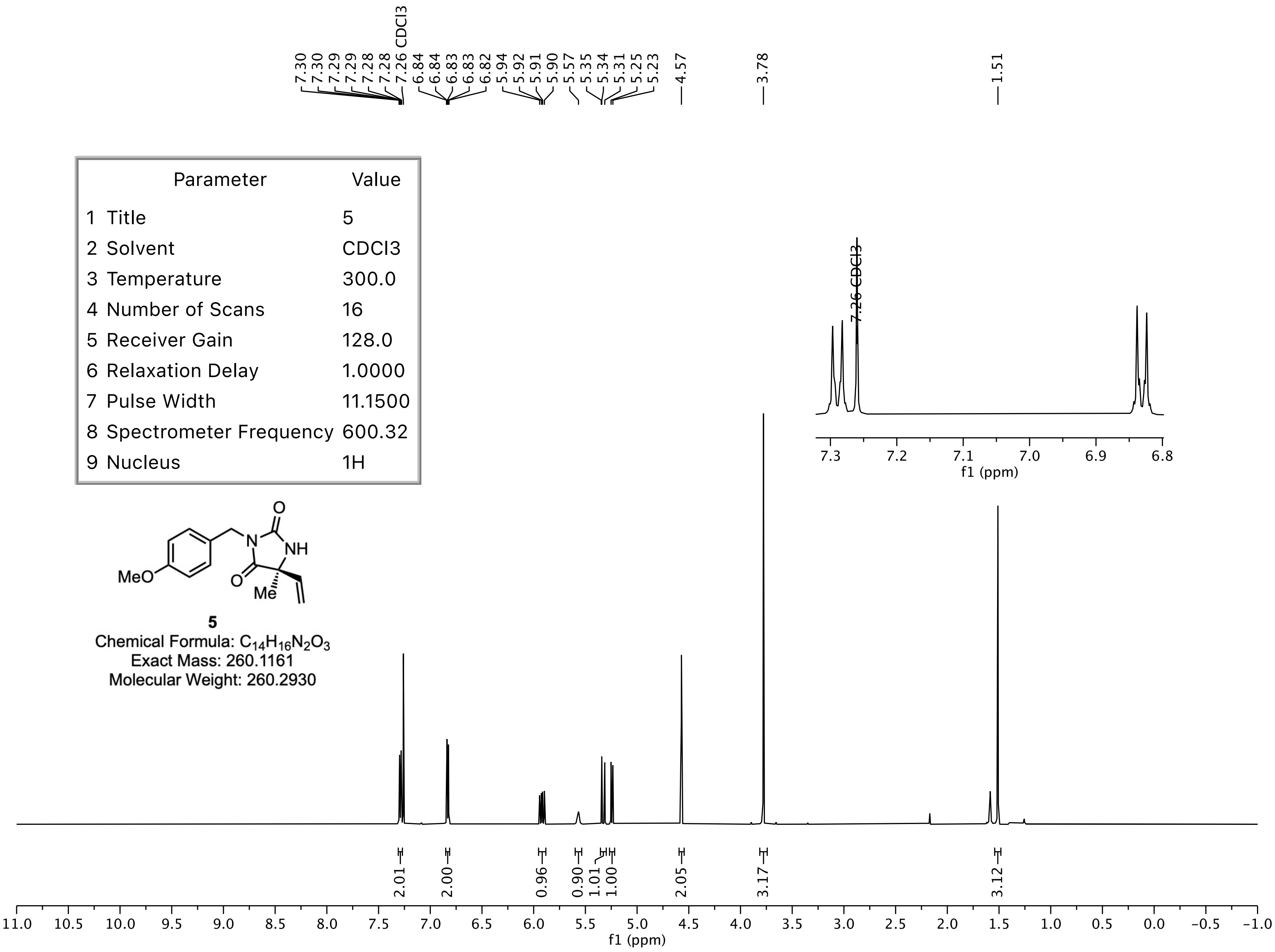


| Parameter | Value |
|---|---|
| 1 Title | 5 |
| 2 Solvent | CDCl3 |
| 3 Temperature | 300.0 |
| 4 Number of Scans | 16 |
| 5 Receiver Gain | 128.0 |
| 6 Relaxation Delay | 1.0000 |
| 7 Pulse Width | 11.1500 |
| 8 Spectrometer Frequency | 600.32 |
| 9 Nucleus | 1H |

**5**

Chemical Formula: $C_{14}H_{16}N_2O_3$
Exact Mass: 260.1161
Molecular Weight: 260.2930

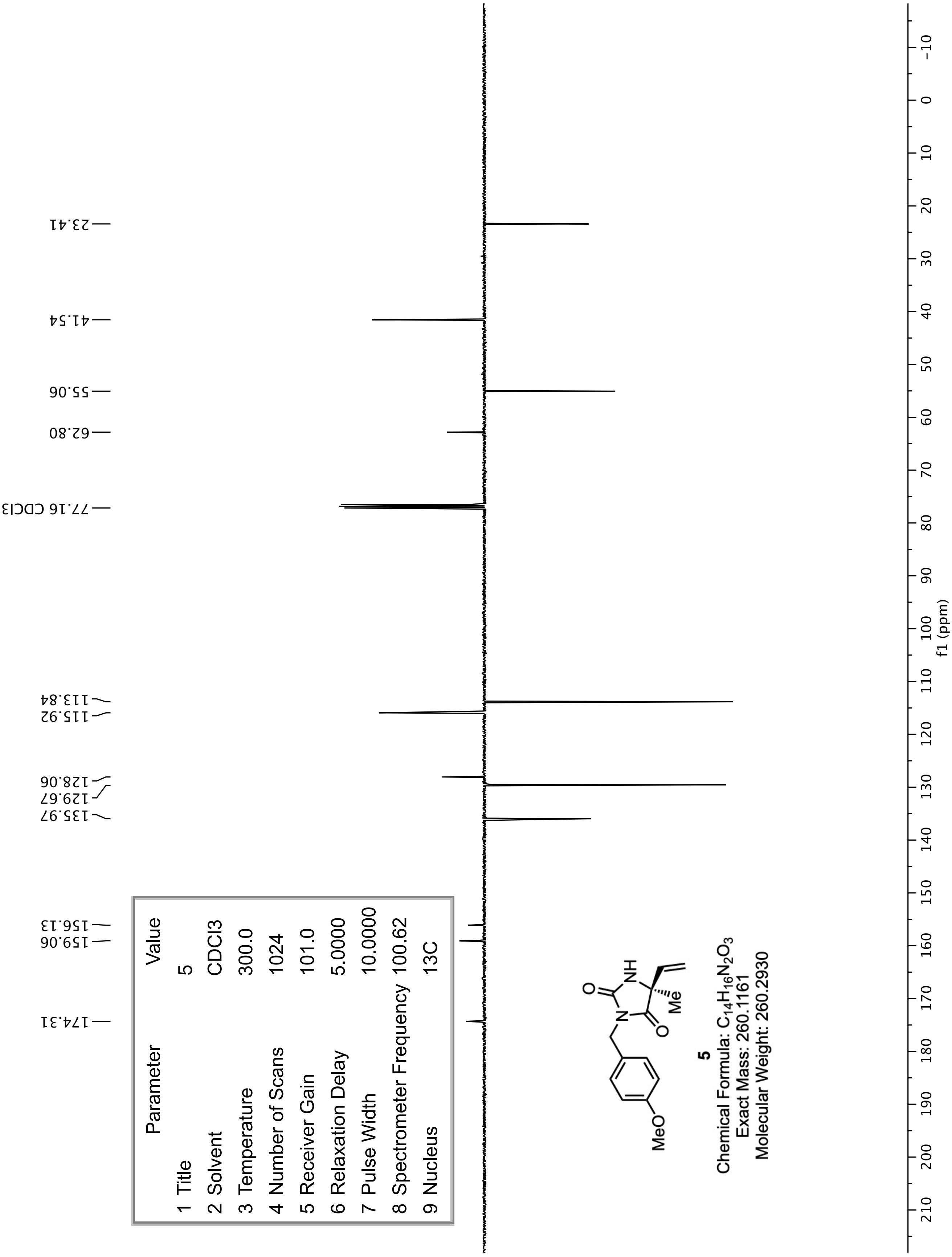


| | Parameter | Value |
|---|---|---|
| 1 | Title | 5 |
| 2 | Solvent | CDCl3 |
| 3 | Temperature | 300.0 |
| 4 | Number of Scans | 1024 |
| 5 | Receiver Gain | 101.0 |
| 6 | Relaxation Delay | 5.0000 |
| 7 | Pulse Width | 10.0000 |
| 8 | Spectrometer Frequency | 100.62 |
| 9 | Nucleus | 13C |

**5**
Chemical Formula: $C_{14}H_{16}N_2O_3$
Exact Mass: 260.1161
Molecular Weight: 260.2930

| Parameter | Value |
|---|---|
| 1 Title | 6 |
| 2 Solvent | CDCl3 |
| 3 Temperature | 298.0 |
| 4 Number of Scans | 16 |
| 5 Receiver Gain | 101.0 |
| 6 Relaxation Delay | 10.0000 |
| 7 Pulse Width | 15.0000 |
| 8 Spectrometer Frequency | 400.13 |
| 9 Nucleus | 1H |

**6**
Chemical Formula: $C_{15}H_{16}N_2O_4$
Exact Mass: 288.1110
Molecular Weight: 288.3030

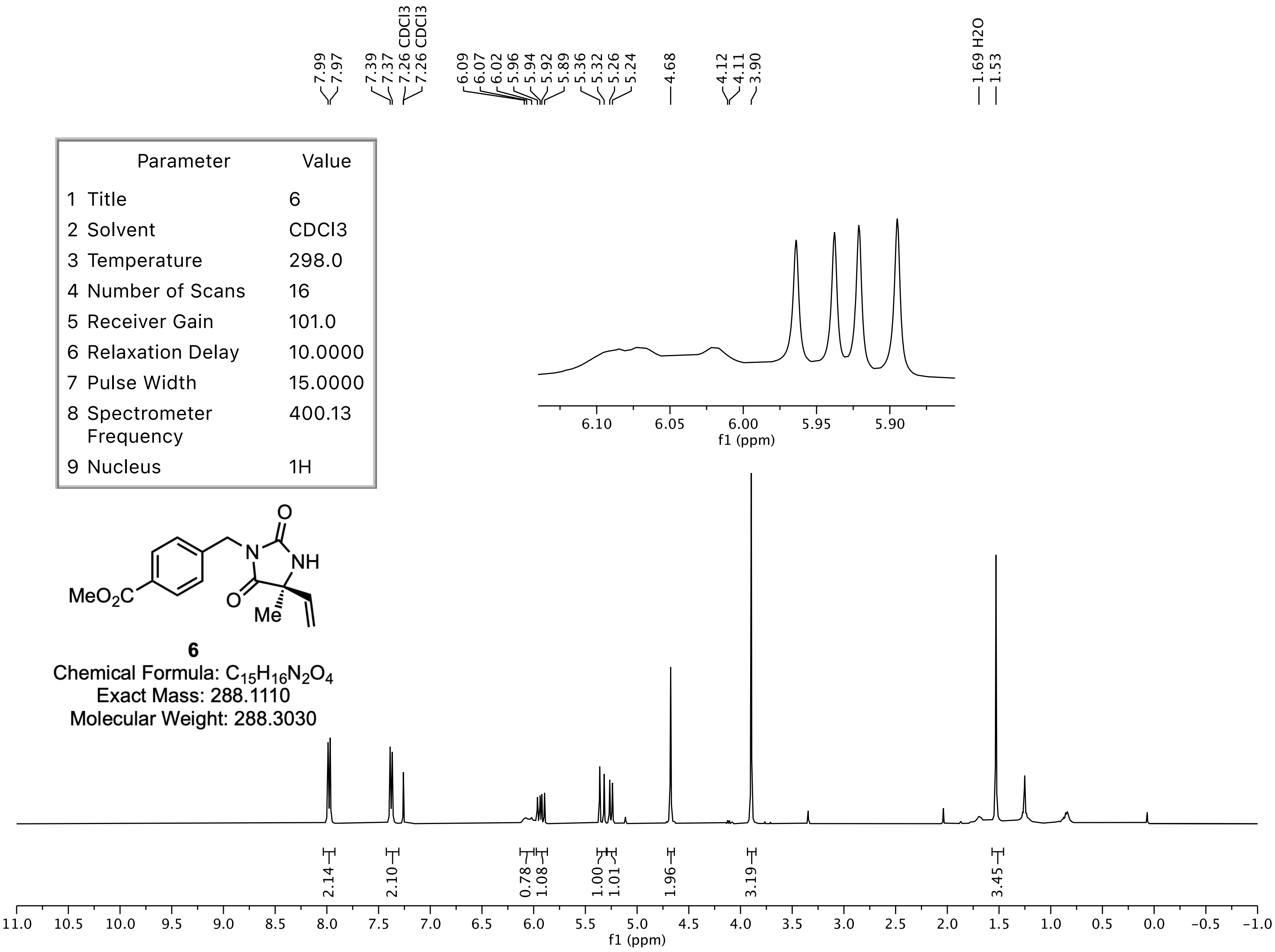

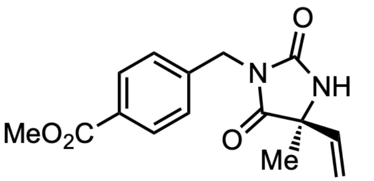

**6**

Chemical Formula: $C_{15}H_{16}N_2O_4$

Exact Mass: 288.1110

Molecular Weight: 288.3030

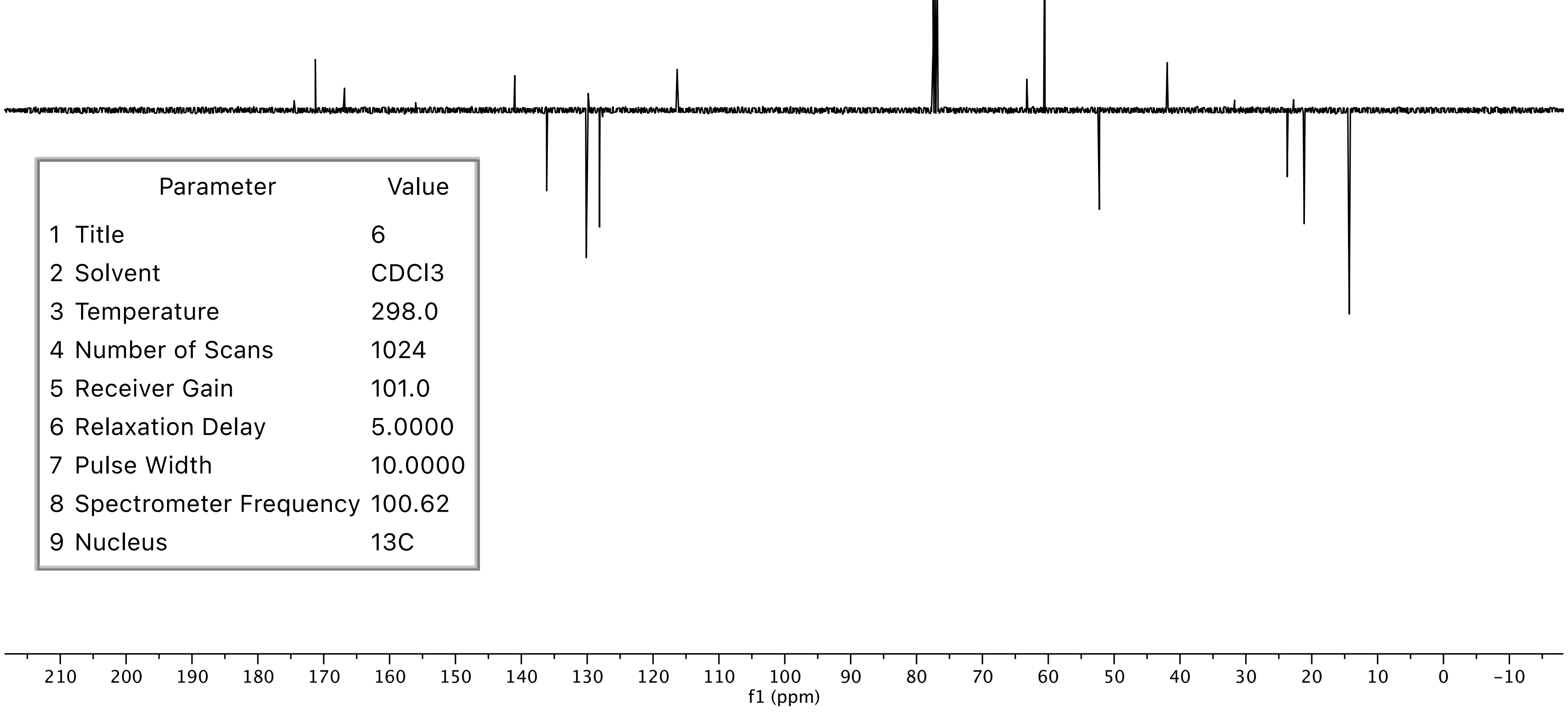


| | Parameter | Value |
|---|---|---|
| 1 | Title | 6 |
| 2 | Solvent | CDCl3 |
| 3 | Temperature | 298.0 |
| 4 | Number of Scans | 1024 |
| 5 | Receiver Gain | 101.0 |
| 6 | Relaxation Delay | 5.0000 |
| 7 | Pulse Width | 10.0000 |
| 8 | Spectrometer Frequency | 100.62 |
| 9 | Nucleus | 13C |

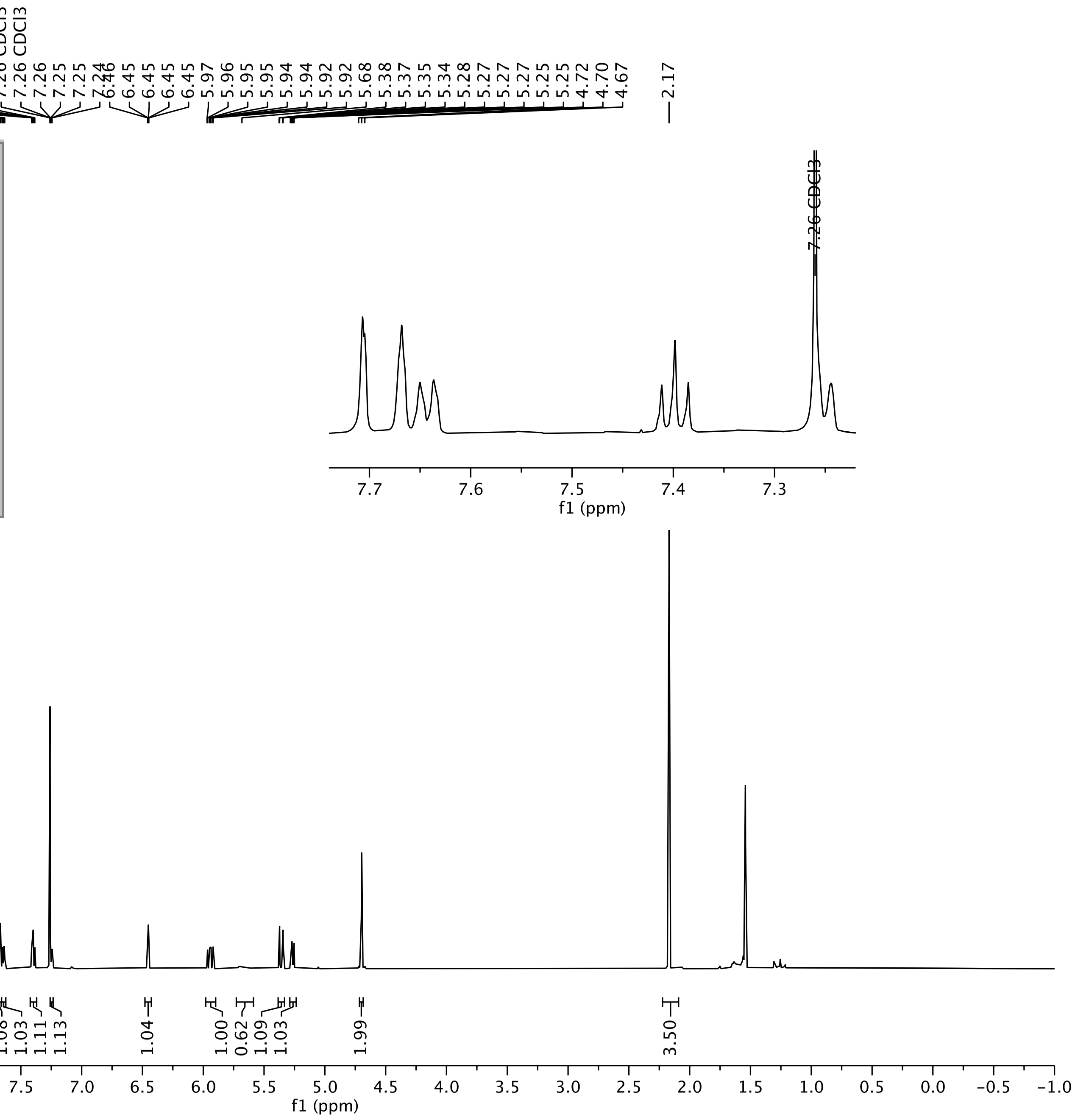


| Parameter | Value |
|---|---|
| 1 Title | 7 |
| 2 Solvent | CDCl3 |
| 3 Temperature | 298.0 |
| 4 Number of Scans | 16 |
| 5 Receiver Gain | 64.0 |
| 6 Relaxation Delay | 10.0000 |
| 7 Pulse Width | 12.0000 |
| 8 Spectrometer Frequency | 600.32 |
| 9 Nucleus | 1H |

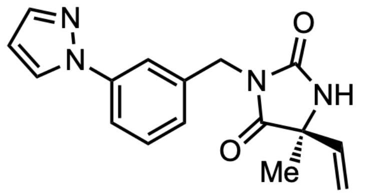


**7**

Chemical Formula: $C_{16}H_{16}N_4O_2$

Exact Mass: 296.1273

Molecular Weight: 296.3300

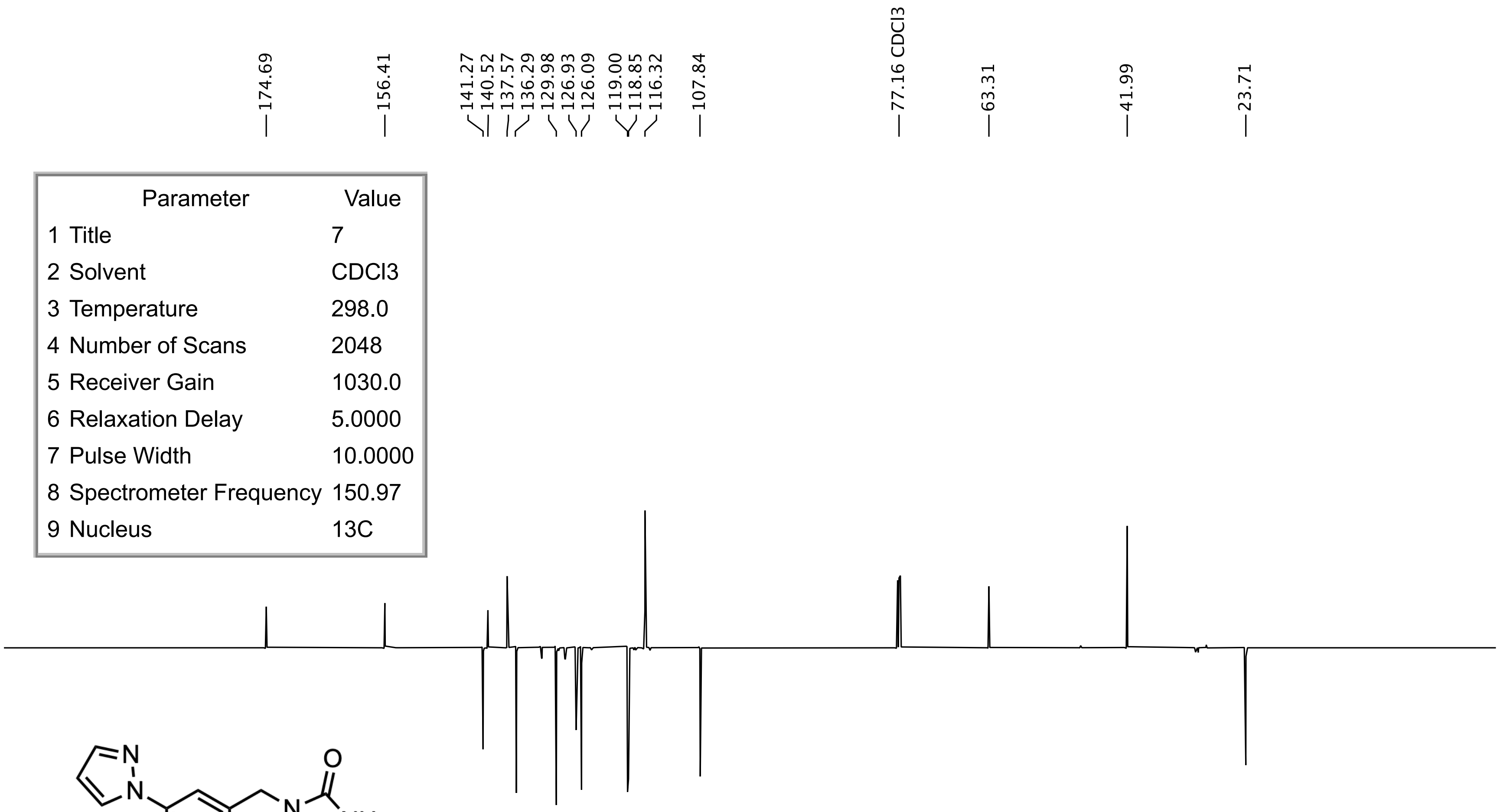


| Parameter | Value |
|---|---|
| 1 Title | 7 |
| 2 Solvent | CDCl3 |
| 3 Temperature | 298.0 |
| 4 Number of Scans | 2048 |
| 5 Receiver Gain | 1030.0 |
| 6 Relaxation Delay | 5.0000 |
| 7 Pulse Width | 10.0000 |
| 8 Spectrometer Frequency | 150.97 |
| 9 Nucleus | 13C |

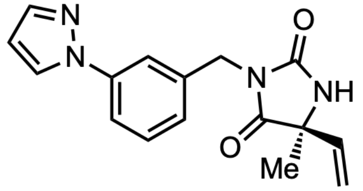


**7**

Chemical Formula: $C_{16}H_{16}N_4O_2$

Exact Mass: 296.1273

Molecular Weight: 296.3300

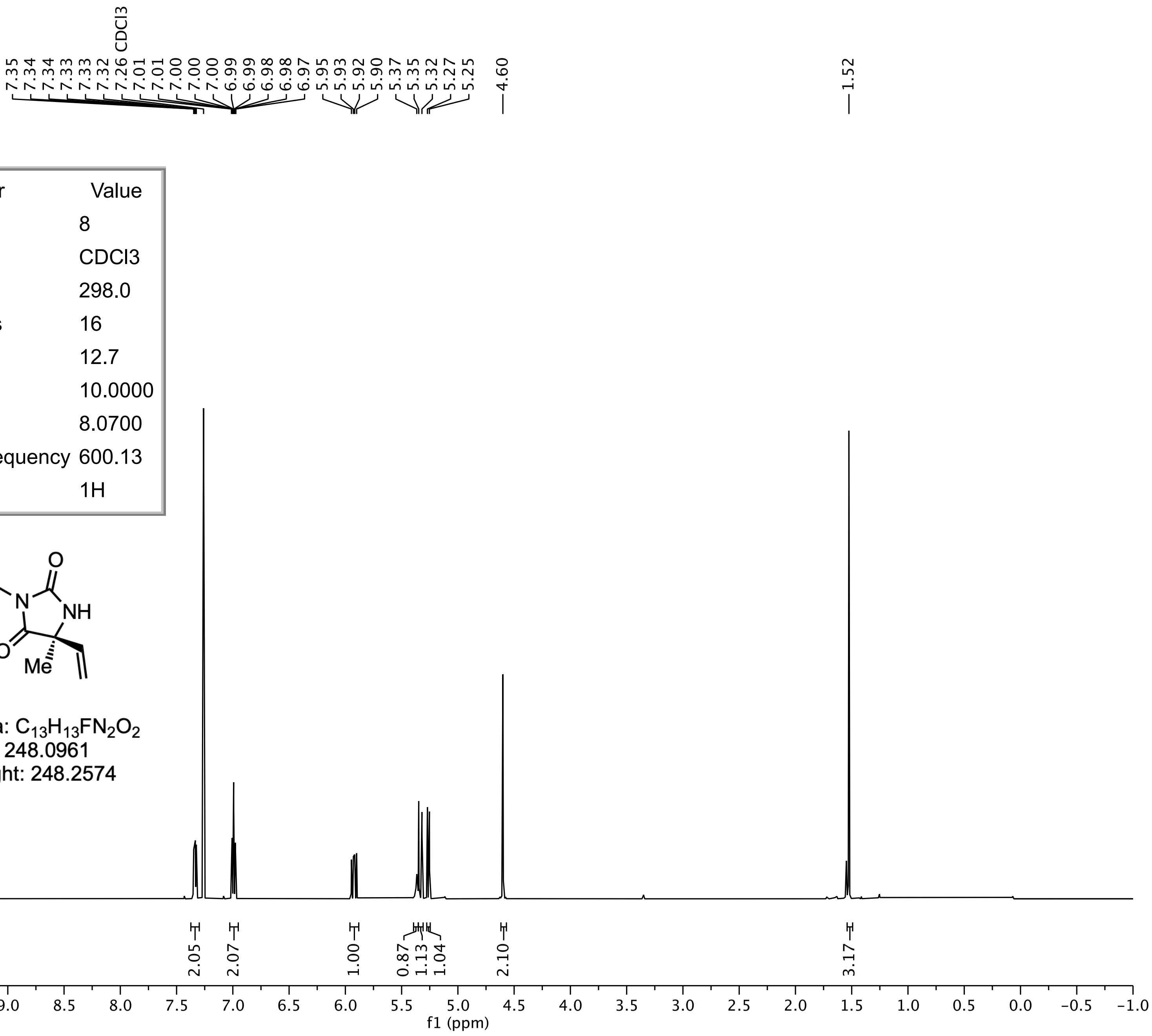


| Parameter | Value |
|---|---|
| 1 Title | 8 |
| 2 Solvent | CDCl3 |
| 3 Temperature | 298.0 |
| 4 Number of Scans | 16 |
| 5 Receiver Gain | 12.7 |
| 6 Relaxation Delay | 10.0000 |
| 7 Pulse Width | 8.0700 |
| 8 Spectrometer Frequency | 600.13 |
| 9 Nucleus | 1H |

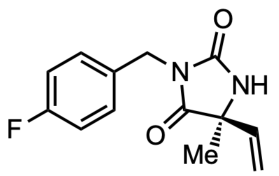


**8**

Chemical Formula: $C_{13}H_{13}FN_2O_2$

Exact Mass: 248.0961

Molecular Weight: 248.2574

**8**
Chemical Formula: $C_{13}H_{13}FN_2O_2$
Exact Mass: 248.0961
Molecular Weight: 248.2574

| Parameter | Value |
| --- | --- |
| 1 Title | 8 |
| 2 Solvent | CDCl3 |
| 3 Temperature | 298.0 |
| 4 Number of Scans | 2048 |
| 5 Receiver Gain | 101.0 |
| 6 Relaxation Delay | 2.0000 |
| 7 Pulse Width | 11.9800 |
| 8 Spectrometer Frequency | 150.92 |
| 9 Nucleus | 13C |

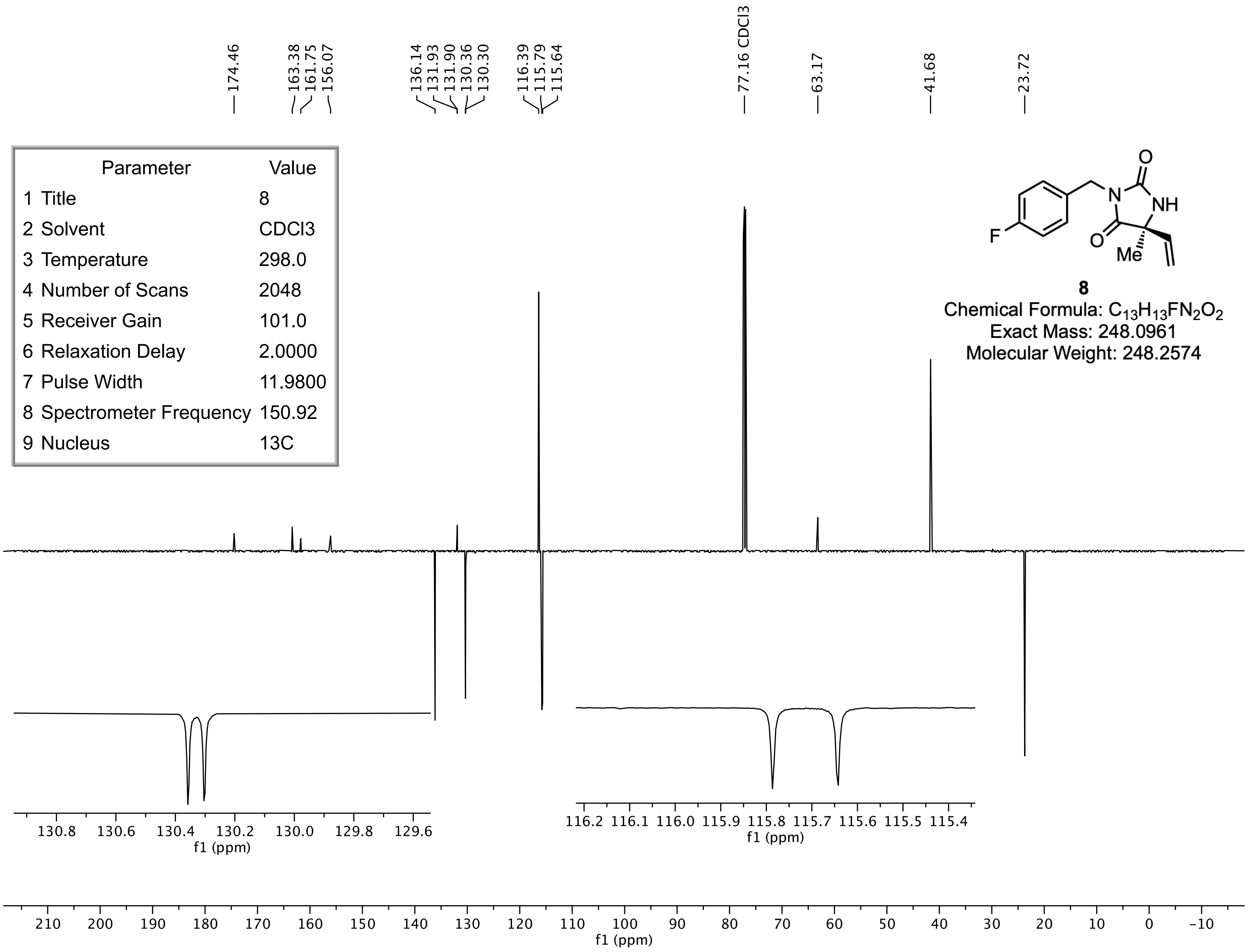

| Parameter | Value |
|---|---|
| 1 Title | 8 |
| 2 Solvent | CDCl3 |
| 3 Temperature | 298.1 |
| 4 Number of Scans | 16 |
| 5 Receiver Gain | 101.0 |
| 6 Relaxation Delay | 1.0000 |
| 7 Pulse Width | 15.0000 |
| 8 Spectrometer Frequency | 376.46 |
| 9 Nucleus | 19F |

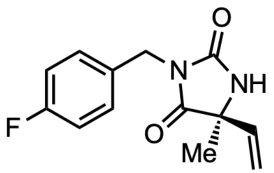


**8**

Chemical Formula: $C_{13}H_{13}FN_2O_2$

Exact Mass: 248.0961

Molecular Weight: 248.2574

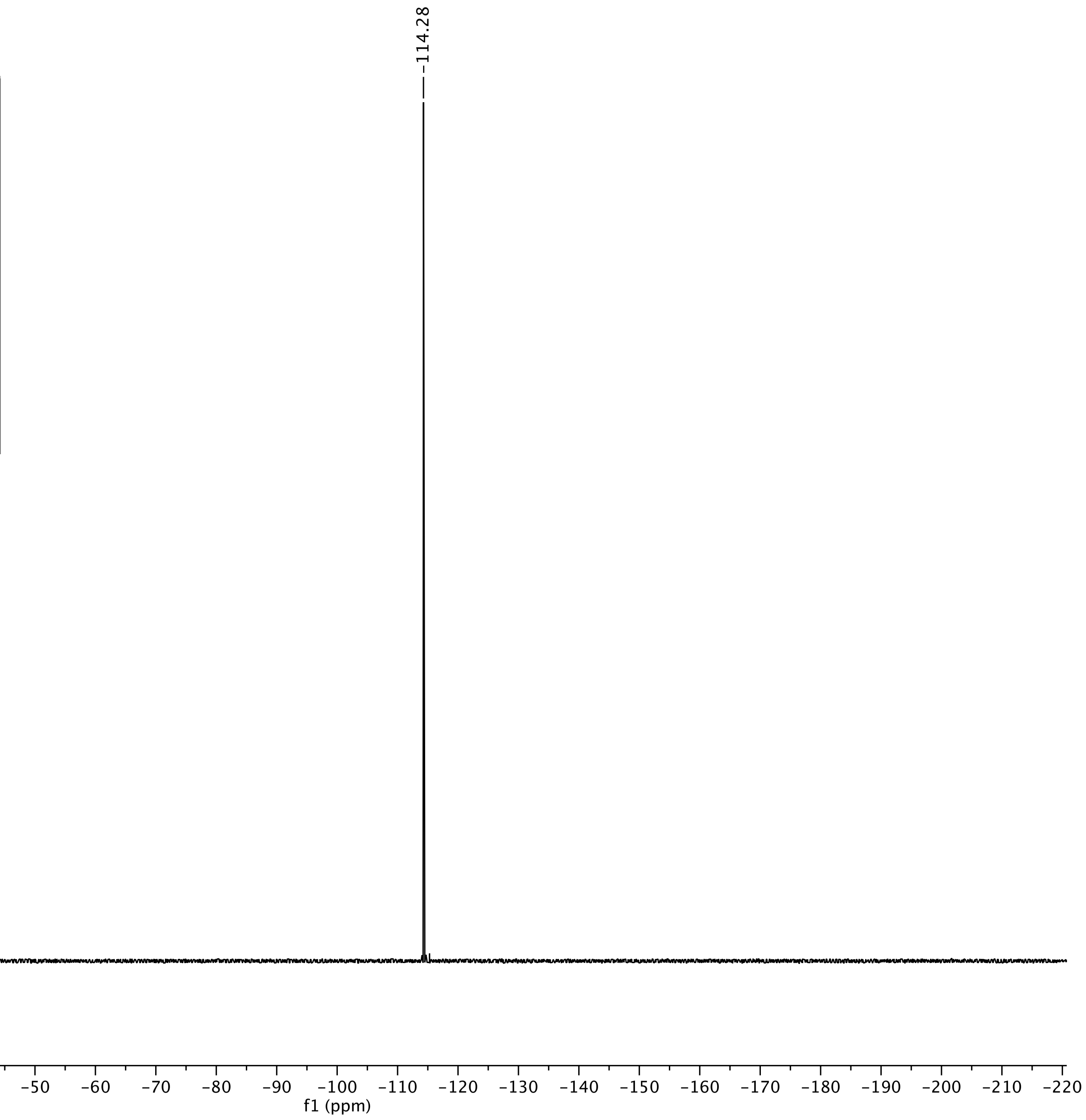

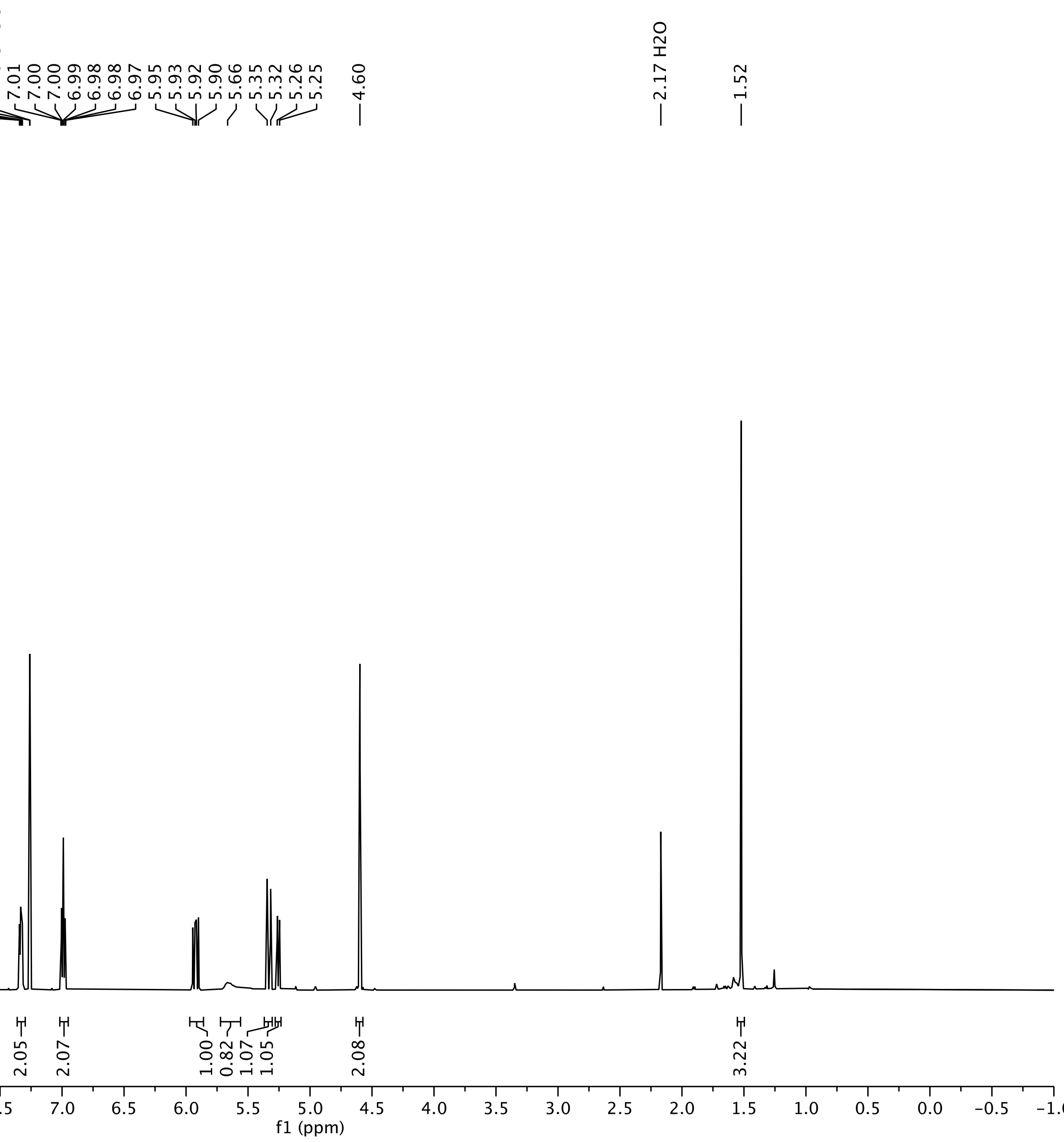


| Parameter | Value |
|---|---|
| 1 Title | 9 |
| 2 Solvent | CDCl3 |
| 3 Temperature | 298.0 |
| 4 Number of Scans | 16 |
| 5 Receiver Gain | 12.7 |
| 6 Relaxation Delay | 10.0000 |
| 7 Pulse Width | 8.0700 |
| 8 Spectrometer Frequency | 600.13 |
| 9 Nucleus | 1H |

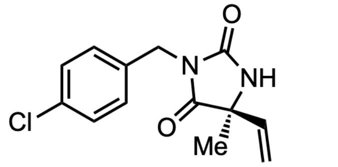


**9**
Chemical Formula: $C_{13}H_{13}ClN_2O_2$
Exact Mass: 264.0666
Molecular Weight: 264.7090

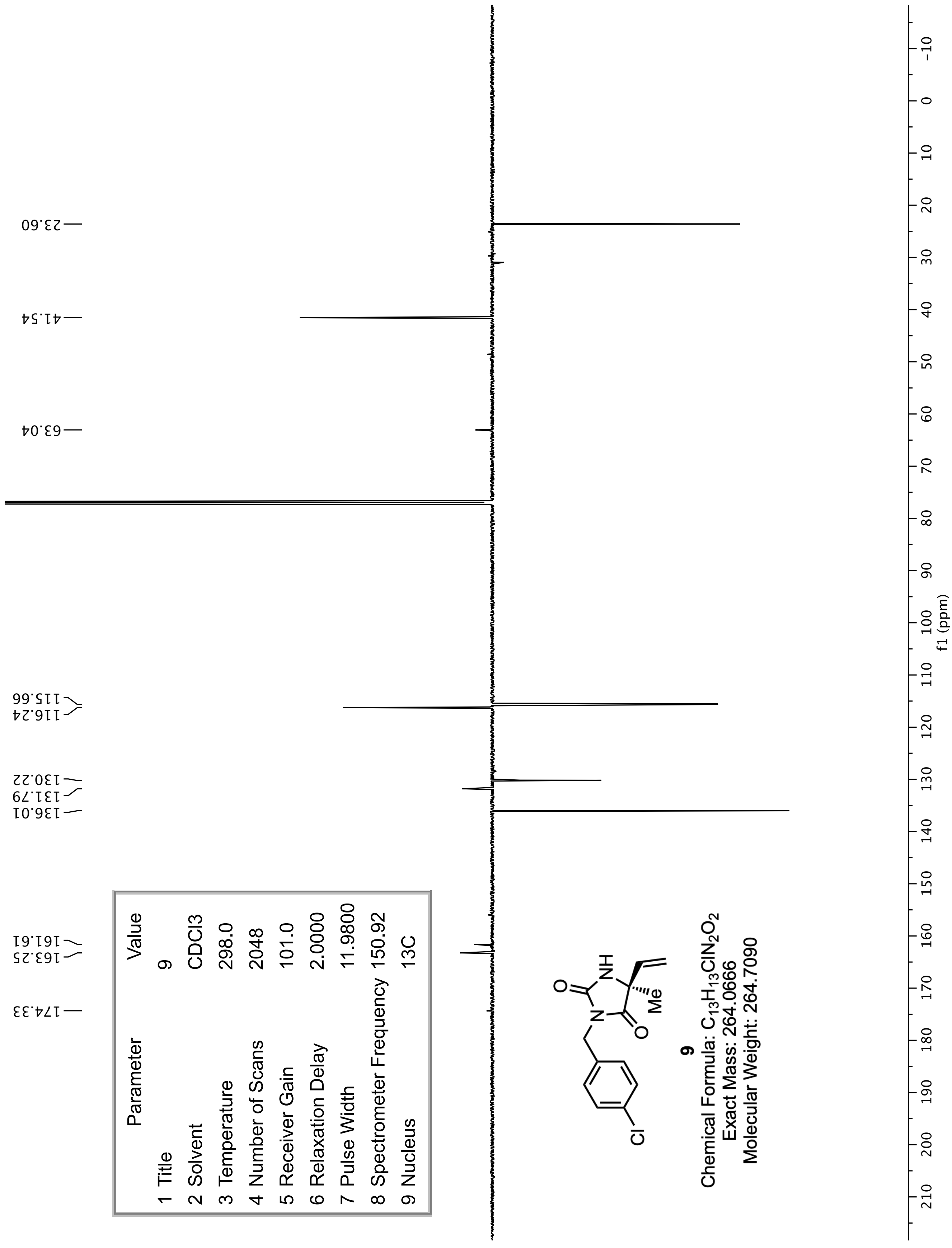
174.33
163.25
161.61
136.01
131.79
130.22
116.24
115.66
63.04
41.54
23.60
Parameter | Value
1 Title | 9
2 Solvent | CDCl3
3 Temperature | 298.0
4 Number of Scans | 2048
5 Receiver Gain | 101.0
6 Relaxation Delay | 2.0000
7 Pulse Width | 11.9800
8 Spectrometer Frequency | 150.92
9 Nucleus | 13C
Cl
O
N
NH
Me
9
Chemical Formula: C13H13ClN2O2
Exact Mass: 264.0666
Molecular Weight: 264.7090
f1 (ppm)
210 200 190 180 170 160 150 140 130 120 110 100 90 80 70 60 50 40 30 20 10 0 -10

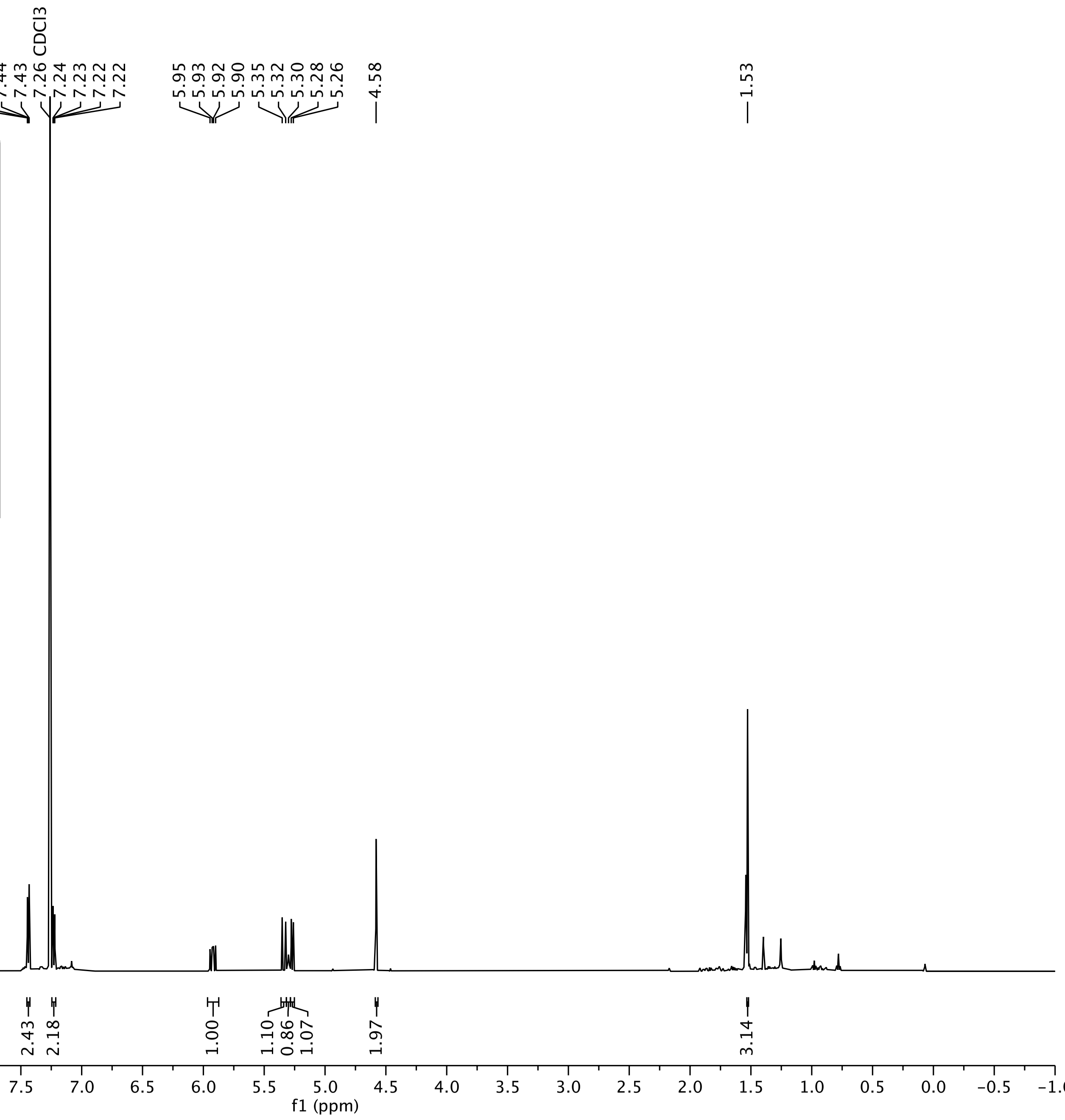


| Parameter | Value |
|---|---|
| 1 Title | 10 |
| 2 Solvent | CDCl3 |
| 3 Temperature | 298.0 |
| 4 Number of Scans | 64 |
| 5 Receiver Gain | 11.3 |
| 6 Relaxation Delay | 10.0000 |
| 7 Pulse Width | 8.0700 |
| 8 Spectrometer Frequency | 600.13 |
| 9 Nucleus | 1H |

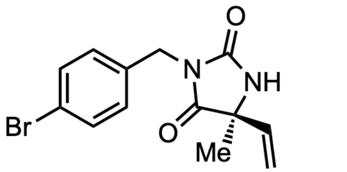


**10**
Chemical Formula: $C_{13}H_{13}BrN_2O_2$
Exact Mass: 308.0160
Molecular Weight: 309.1630

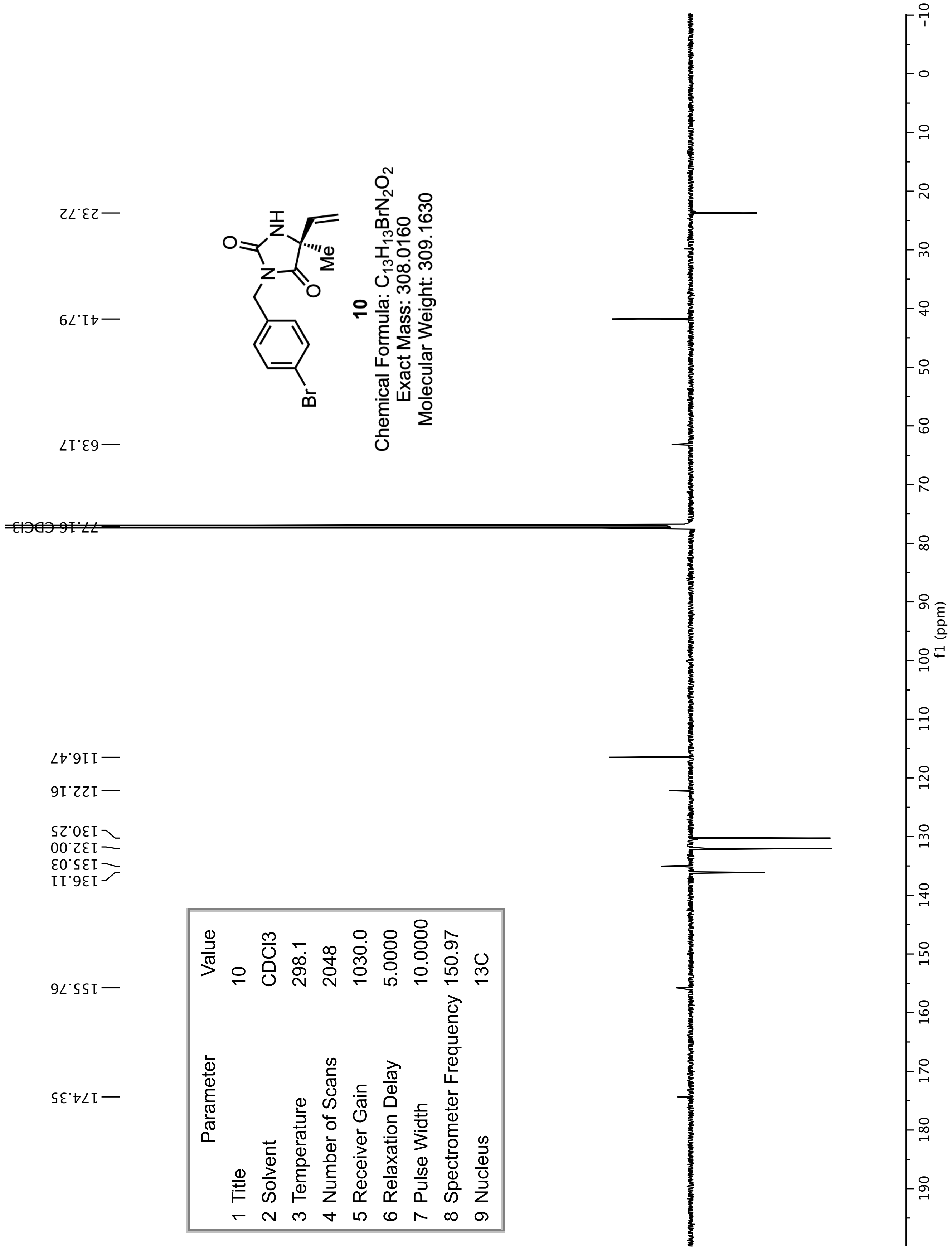


| Parameter | Value |
|---|---|
| 1 Title | 10 |
| 2 Solvent | CDCl3 |
| 3 Temperature | 298.1 |
| 4 Number of Scans | 2048 |
| 5 Receiver Gain | 1030.0 |
| 6 Relaxation Delay | 5.0000 |
| 7 Pulse Width | 10.0000 |
| 8 Spectrometer Frequency | 150.97 |
| 9 Nucleus | 13C |

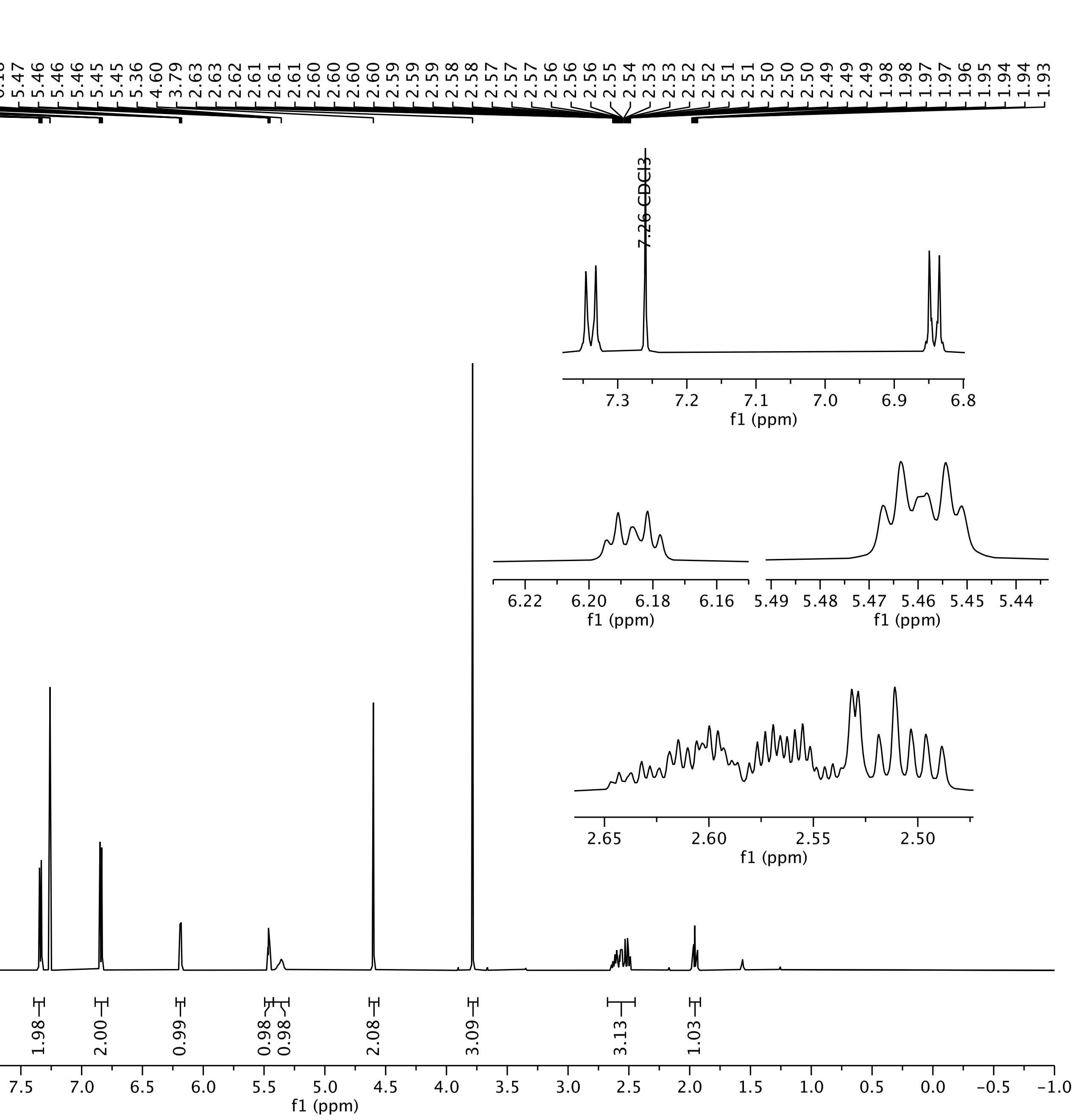


| Parameter | Value |
|---|---|
| 1 Title | 11 |
| 2 Solvent | CDCl3 |
| 3 Temperature | 298.0 |
| 4 Number of Scans | 16 |
| 5 Receiver Gain | 20.2 |
| 6 Relaxation Delay | 1.0000 |
| 7 Pulse Width | 8.2200 |
| 8 Spectrometer Frequency | 600.13 |
| 9 Nucleus | 1H |

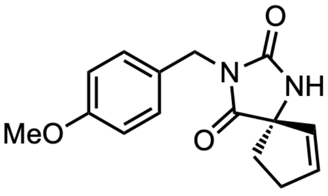


**11**

Chemical Formula: $C_{15}H_{16}N_2O_3$
Exact Mass: 272.1161
Molecular Weight: 272.3040

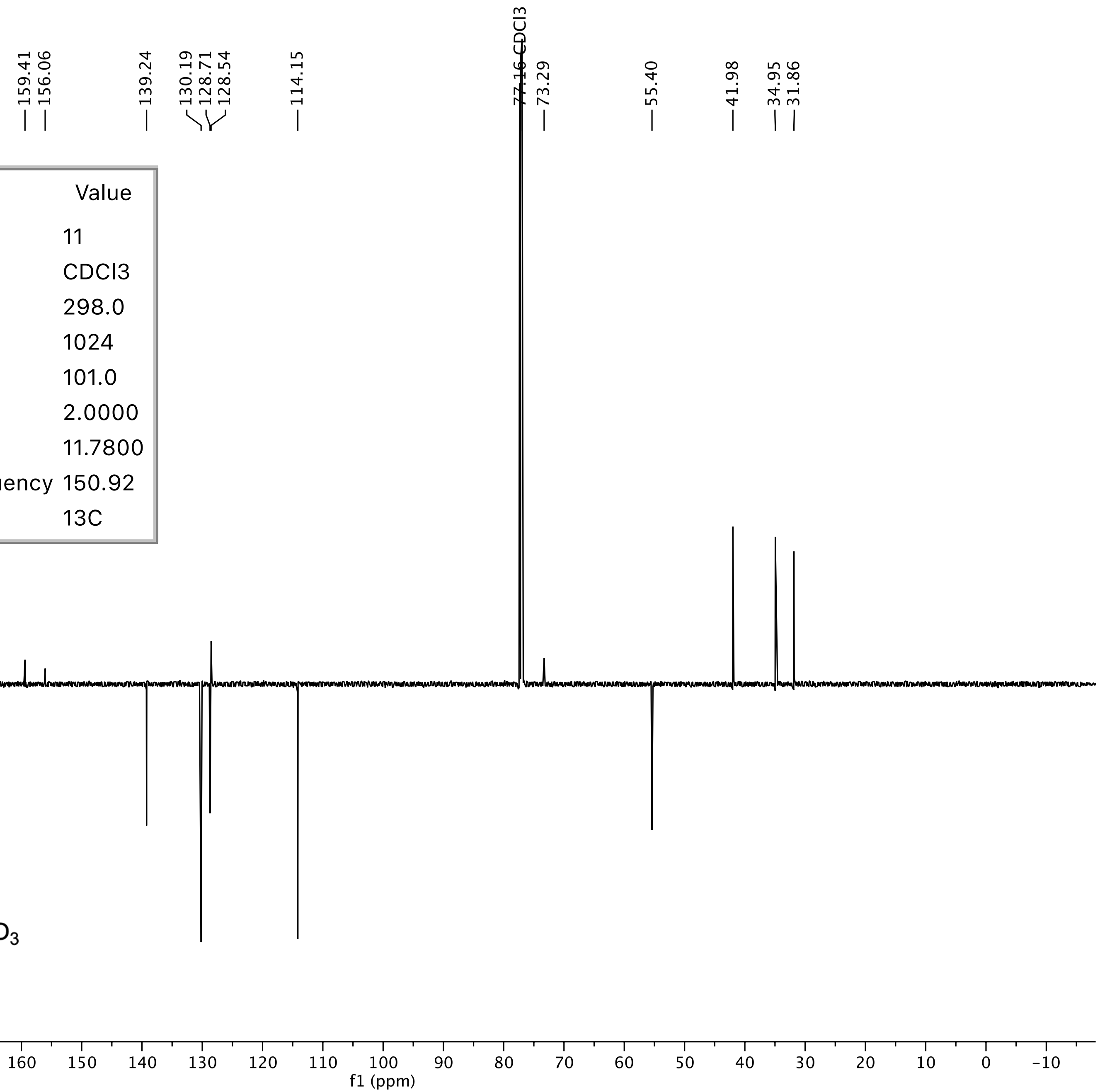


| | Parameter | Value |
|---|---|---|
| 1 | Title | 11 |
| 2 | Solvent | CDCl3 |
| 3 | Temperature | 298.0 |
| 4 | Number of Scans | 1024 |
| 5 | Receiver Gain | 101.0 |
| 6 | Relaxation Delay | 2.0000 |
| 7 | Pulse Width | 11.7800 |
| 8 | Spectrometer Frequency | 150.92 |
| 9 | Nucleus | 13C |

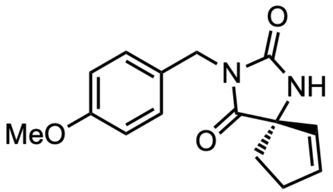


**11**

Chemical Formula: $C_{15}H_{16}N_2O_3$

Exact Mass: 272.1161

Molecular Weight: 272.3040

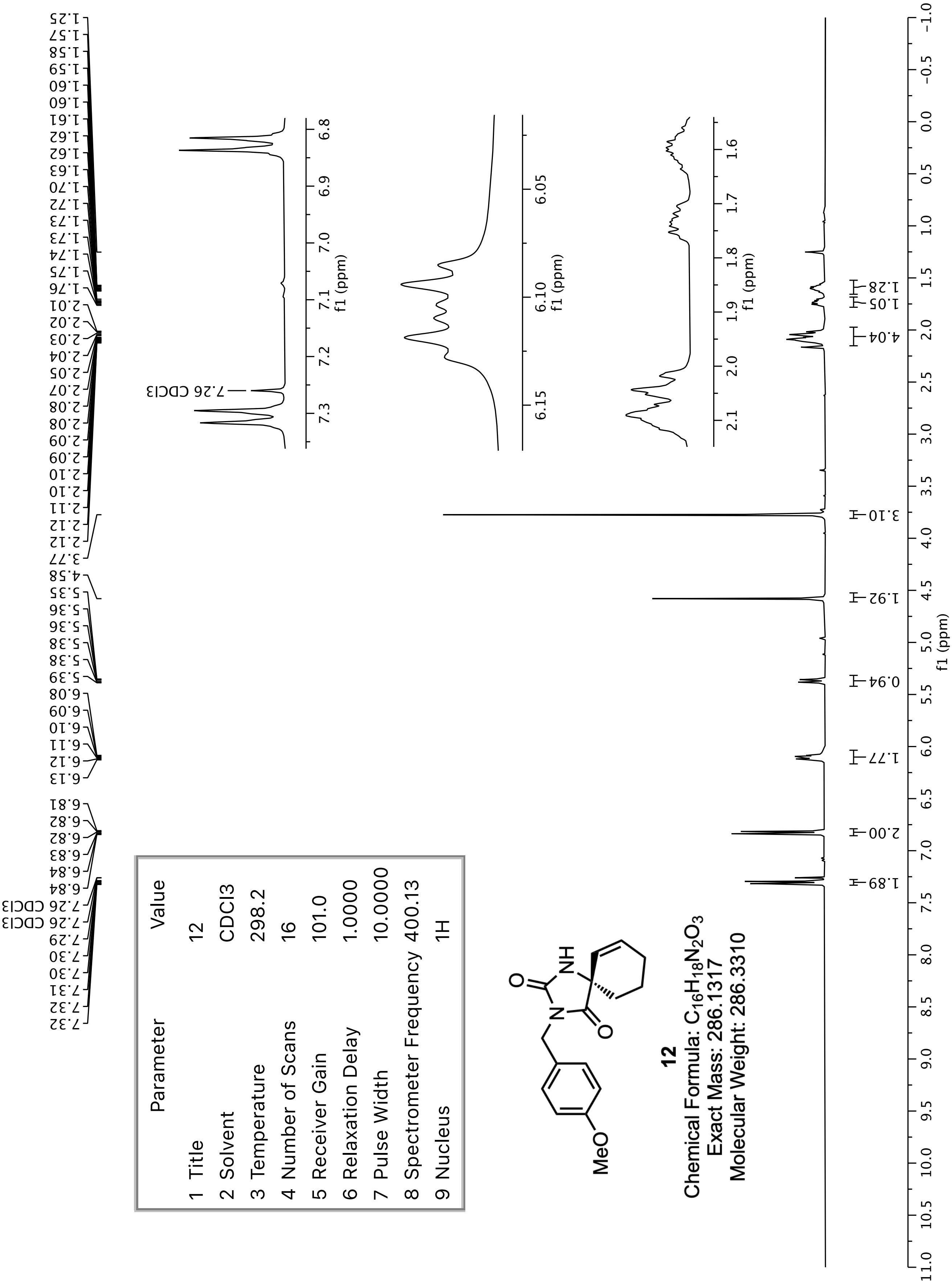


| Parameter | Value |
|---|---|
| 1 Title | 12 |
| 2 Solvent | CDCl3 |
| 3 Temperature | 298.2 |
| 4 Number of Scans | 16 |
| 5 Receiver Gain | 101.0 |
| 6 Relaxation Delay | 1.0000 |
| 7 Pulse Width | 10.0000 |
| 8 Spectrometer Frequency | 400.13 |
| 9 Nucleus | 1H |

**12**

Chemical Formula: $C_{16}H_{18}N_2O_3$
Exact Mass: 286.1317
Molecular Weight: 286.3310

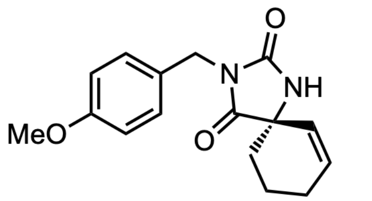


**12**
Chemical Formula: $C_{16}H_{18}N_2O_3$
Exact Mass: 286.1317
Molecular Weight: 286.3310

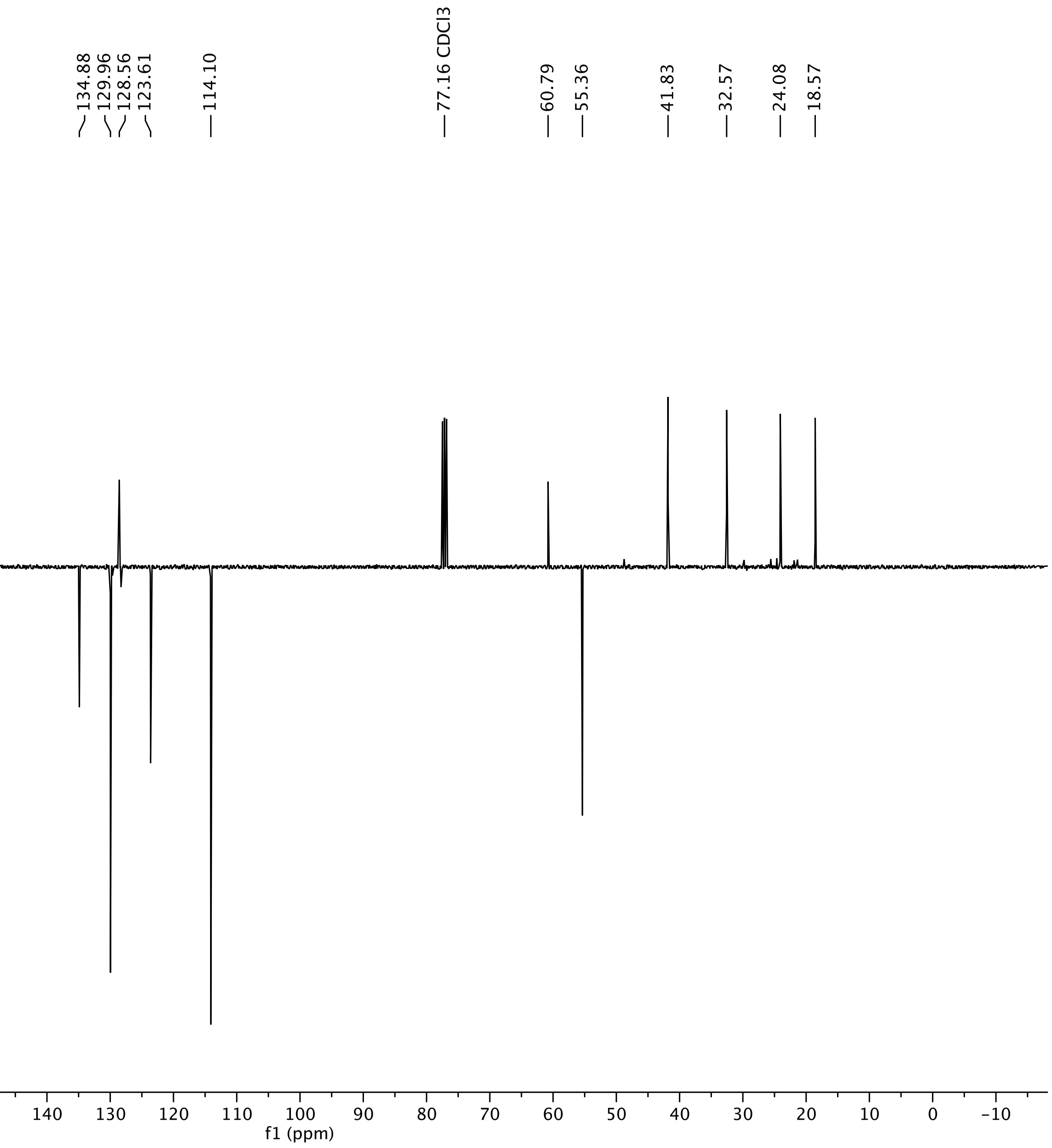


| | Parameter | Value |
|---|---|---|
| 1 | Title | 12 |
| 2 | Solvent | CDCl3 |
| 3 | Temperature | 298.2 |
| 4 | Number of Scans | 1024 |
| 5 | Receiver Gain | 101.0 |
| 6 | Relaxation Delay | 5.0000 |
| 7 | Pulse Width | 10.0000 |
| 8 | Spectrometer Frequency | 100.62 |
| 9 | Nucleus | 13C |

| Parameter | Value |
|---|---|
| 1 Title | 13 |
| 2 Solvent | CDCl3 |
| 3 Temperature | 298.0 |
| 4 Number of Scans | 64 |
| 5 Receiver Gain | 12.7 |
| 6 Relaxation Delay | 10.0000 |
| 7 Pulse Width | 8.0700 |
| 8 Spectrometer Frequency | 600.13 |
| 9 Nucleus | 1H |

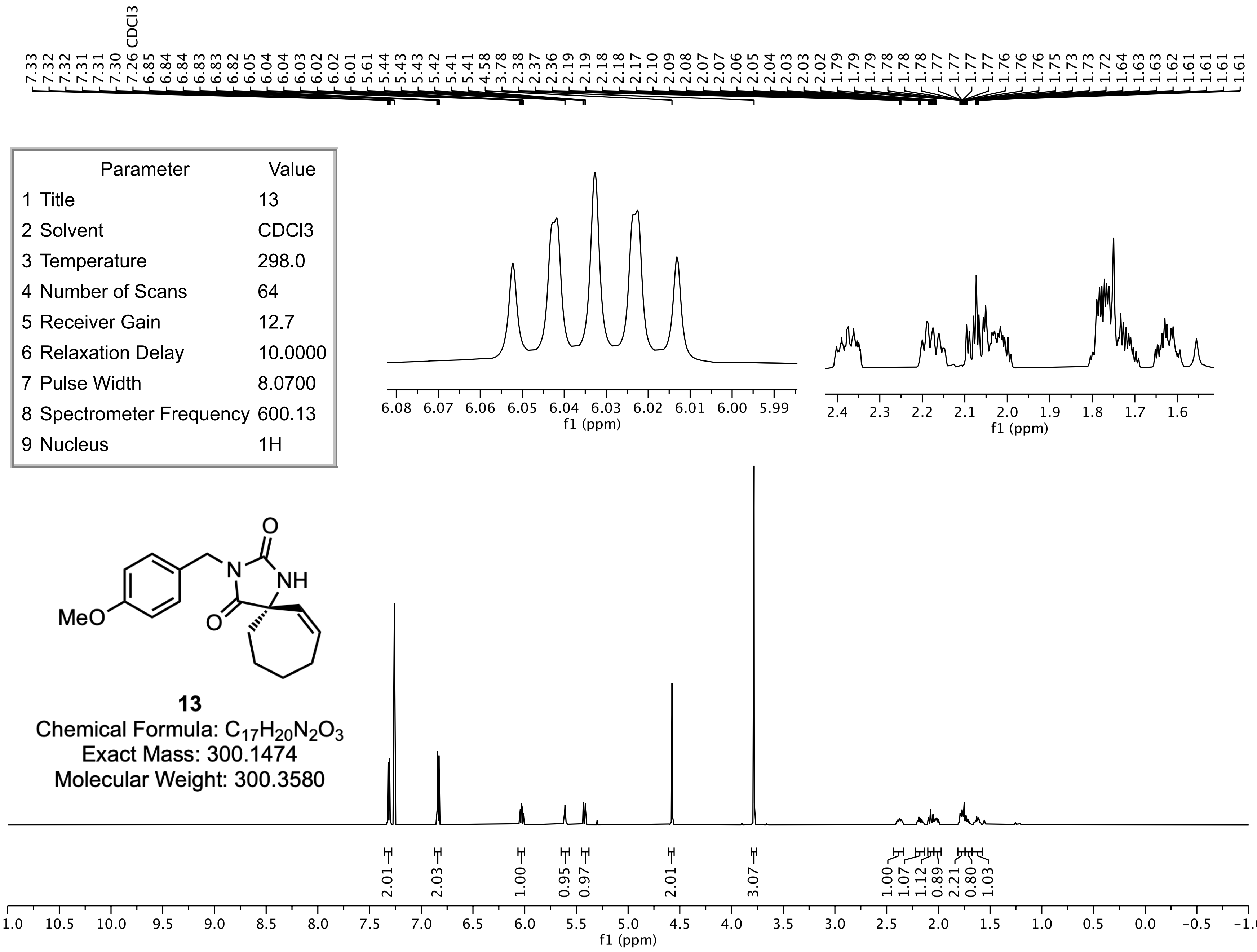


**13**
Chemical Formula: $C_{17}H_{20}N_2O_3$
Exact Mass: 300.1474
Molecular Weight: 300.3580

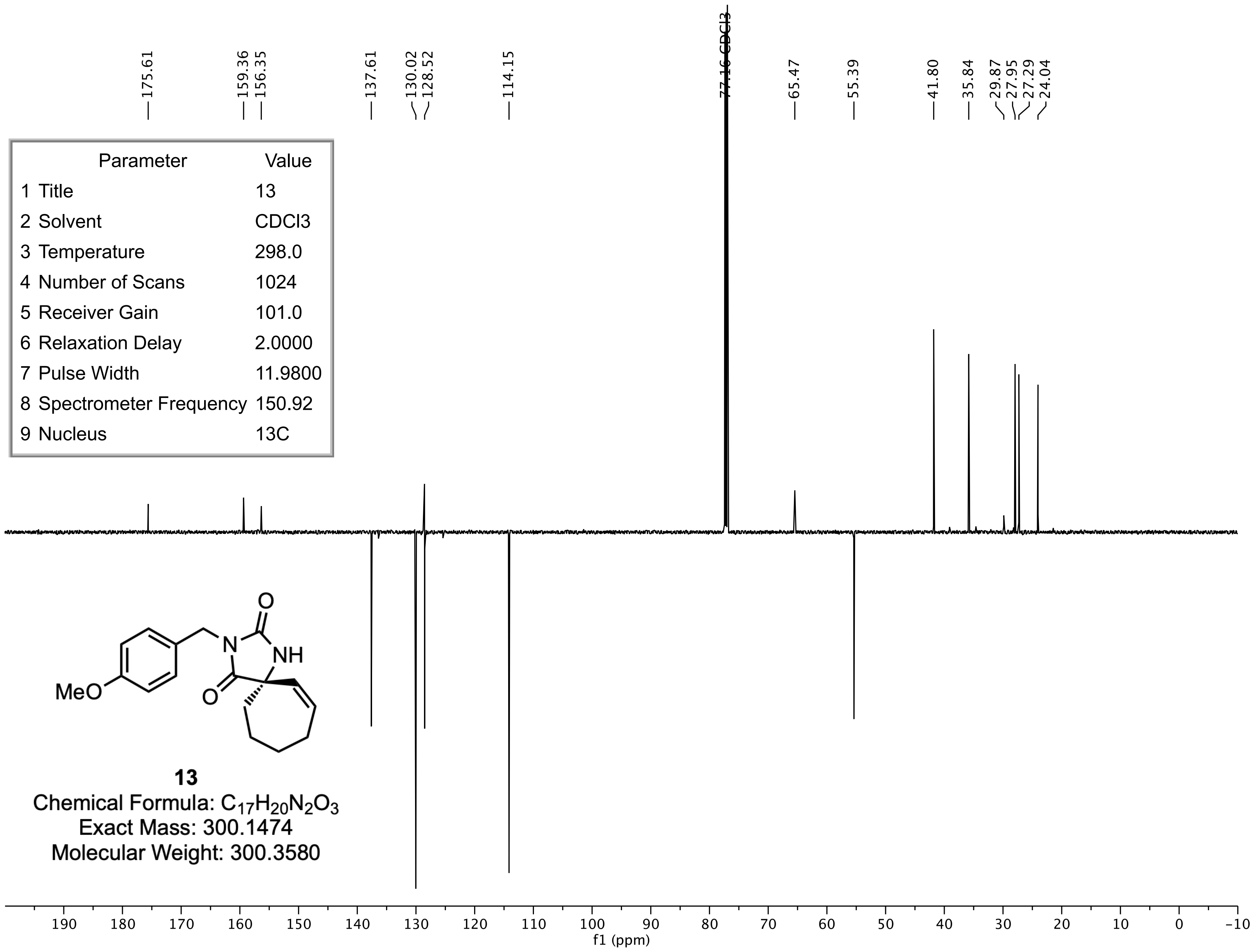


| Parameter | Value |
|---|---|
| 1 Title | 13 |
| 2 Solvent | CDCl3 |
| 3 Temperature | 298.0 |
| 4 Number of Scans | 1024 |
| 5 Receiver Gain | 101.0 |
| 6 Relaxation Delay | 2.0000 |
| 7 Pulse Width | 11.9800 |
| 8 Spectrometer Frequency | 150.92 |
| 9 Nucleus | 13C |

| Parameter | Value |
|---|---|
| 1 Title | 14 |
| 2 Solvent | CD3CN |
| 3 Temperature | 298.0 |
| 4 Number of Scans | 16 |
| 5 Receiver Gain | 12.7 |
| 6 Relaxation Delay | 10.0000 |
| 7 Pulse Width | 8.0000 |
| 8 Spectrometer Frequency | 600.13 |
| 9 Nucleus | 1H |

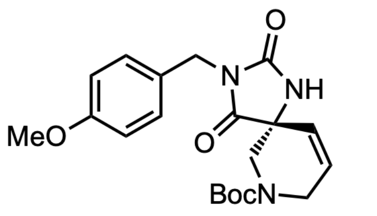


**14**

Chemical Formula: $C_{20}H_{25}N_3O_5$
Exact Mass: 387.1794
Molecular Weight: 387.4360

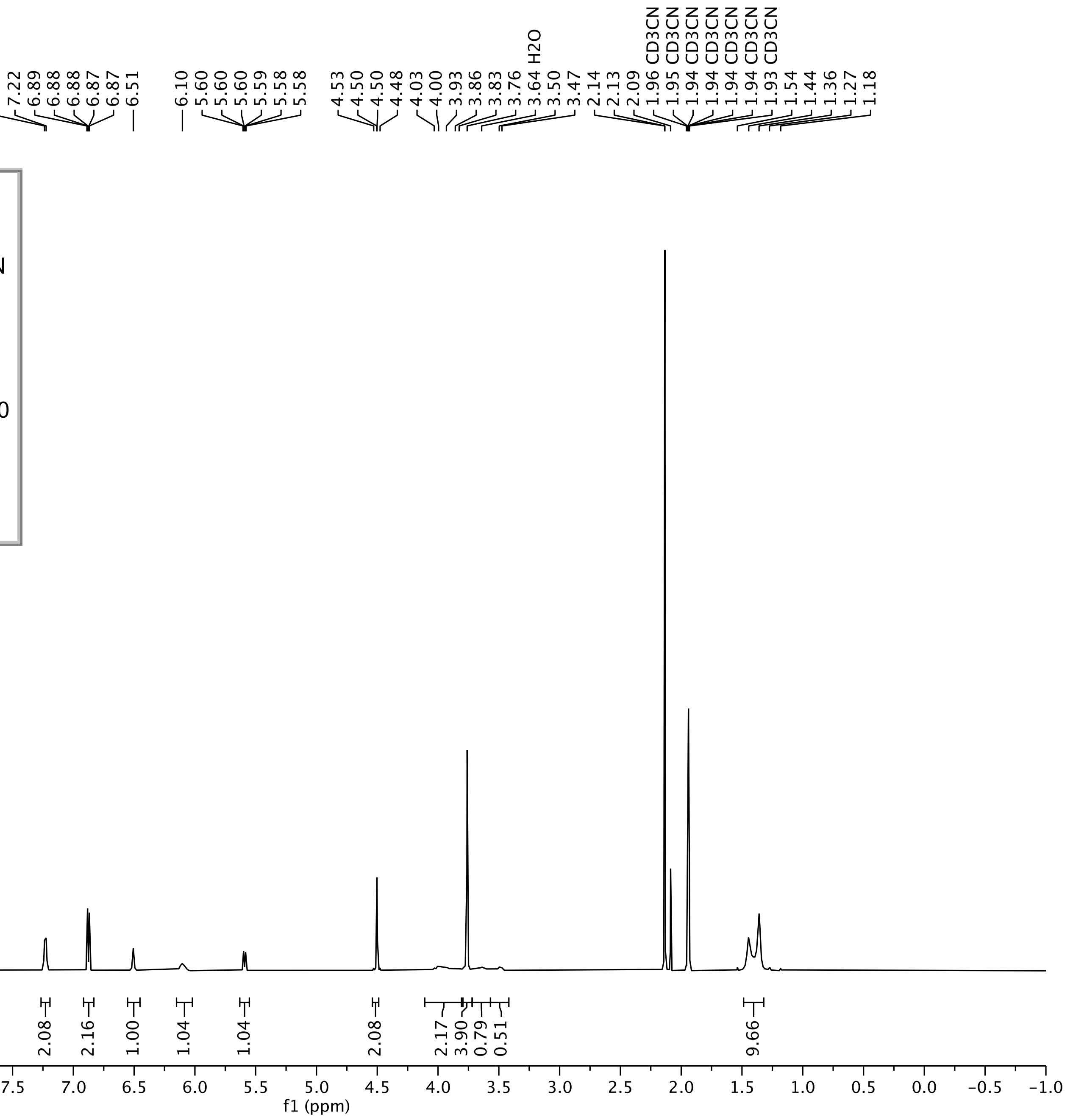

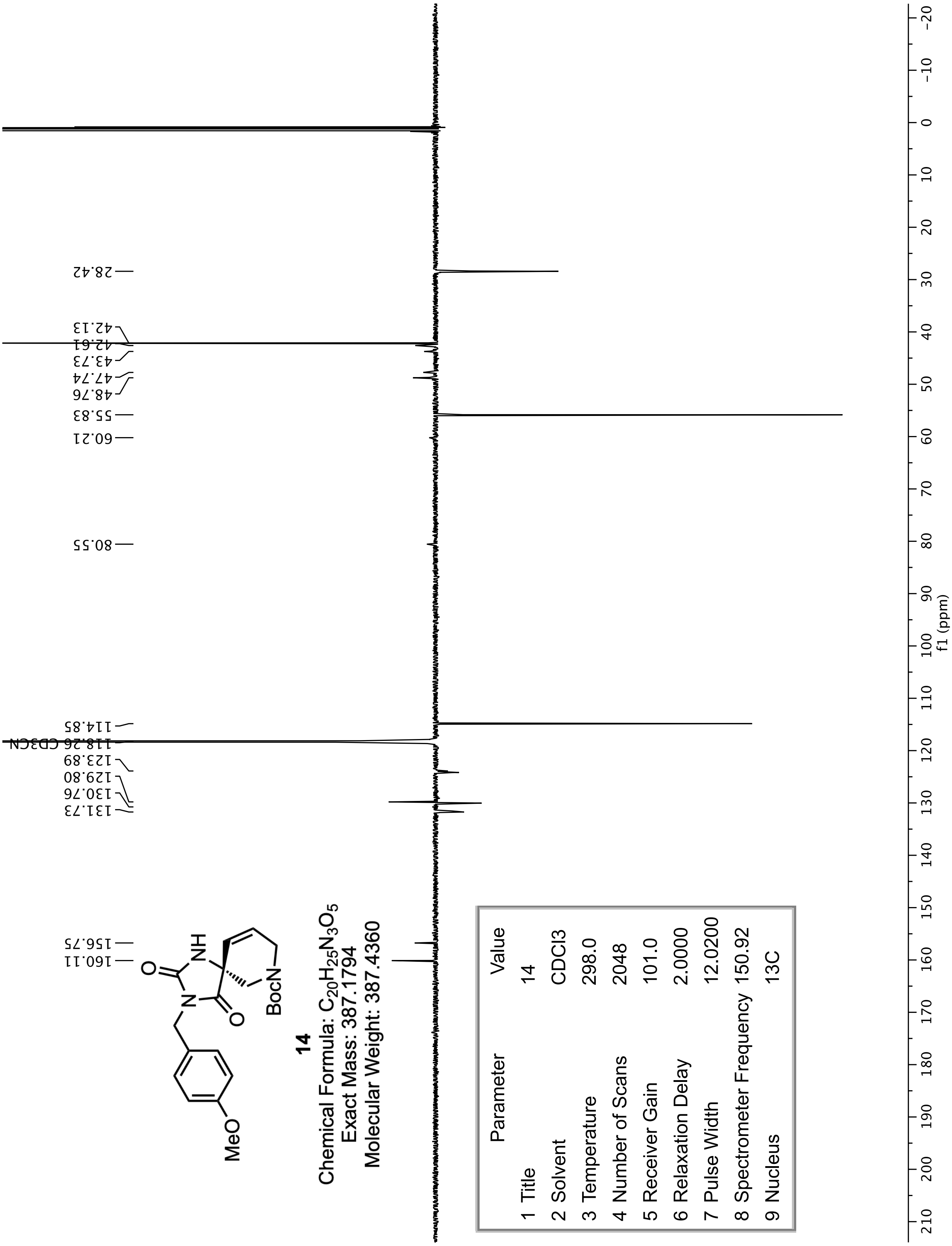

28.42
42.13
42.61
43.73
47.74
48.76
55.83
60.21
80.55
114.85
118.26 CD3CN
123.89
129.80
130.76
131.73
156.75
160.11
MeO
O
N
NH
O
BocN
14
Chemical Formula: $C_{20}H_{25}N_3O_5$
Exact Mass: 387.1794
Molecular Weight: 387.4360
Parameter | Value
1 Title | 14
2 Solvent | CDCl3
3 Temperature | 298.0
4 Number of Scans | 2048
5 Receiver Gain | 101.0
6 Relaxation Delay | 2.0000
7 Pulse Width | 12.0200
8 Spectrometer Frequency | 150.92
9 Nucleus | 13C
f1 (ppm)

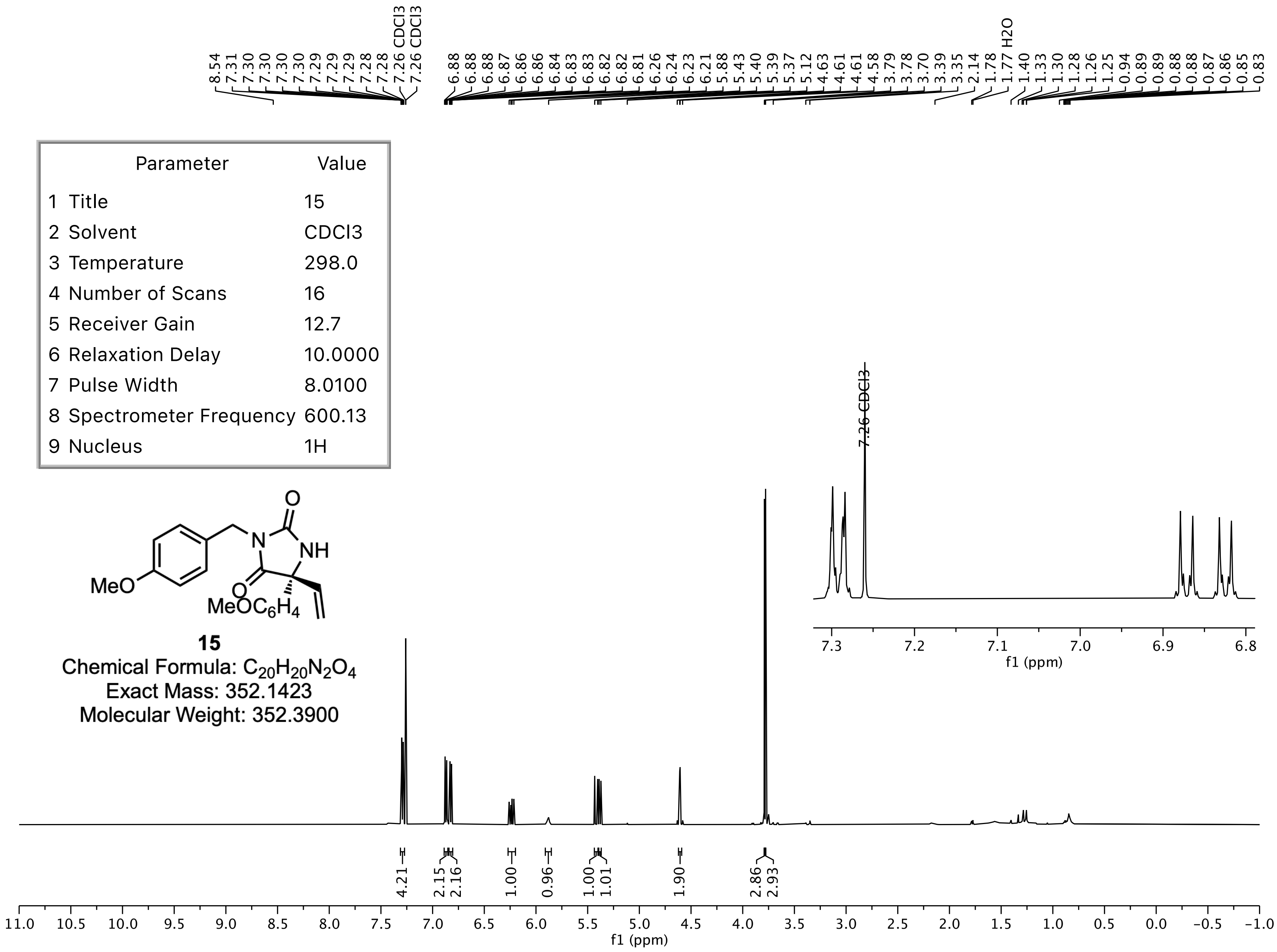


| | Parameter | Value |
|---|---|---|
| 1 | Title | 15 |
| 2 | Solvent | CDCl3 |
| 3 | Temperature | 298.0 |
| 4 | Number of Scans | 16 |
| 5 | Receiver Gain | 12.7 |
| 6 | Relaxation Delay | 10.0000 |
| 7 | Pulse Width | 8.0100 |
| 8 | Spectrometer Frequency | 600.13 |
| 9 | Nucleus | 1H |

**15**

Chemical Formula: $C_{20}H_{20}N_2O_4$

Exact Mass: 352.1423

Molecular Weight: 352.3900

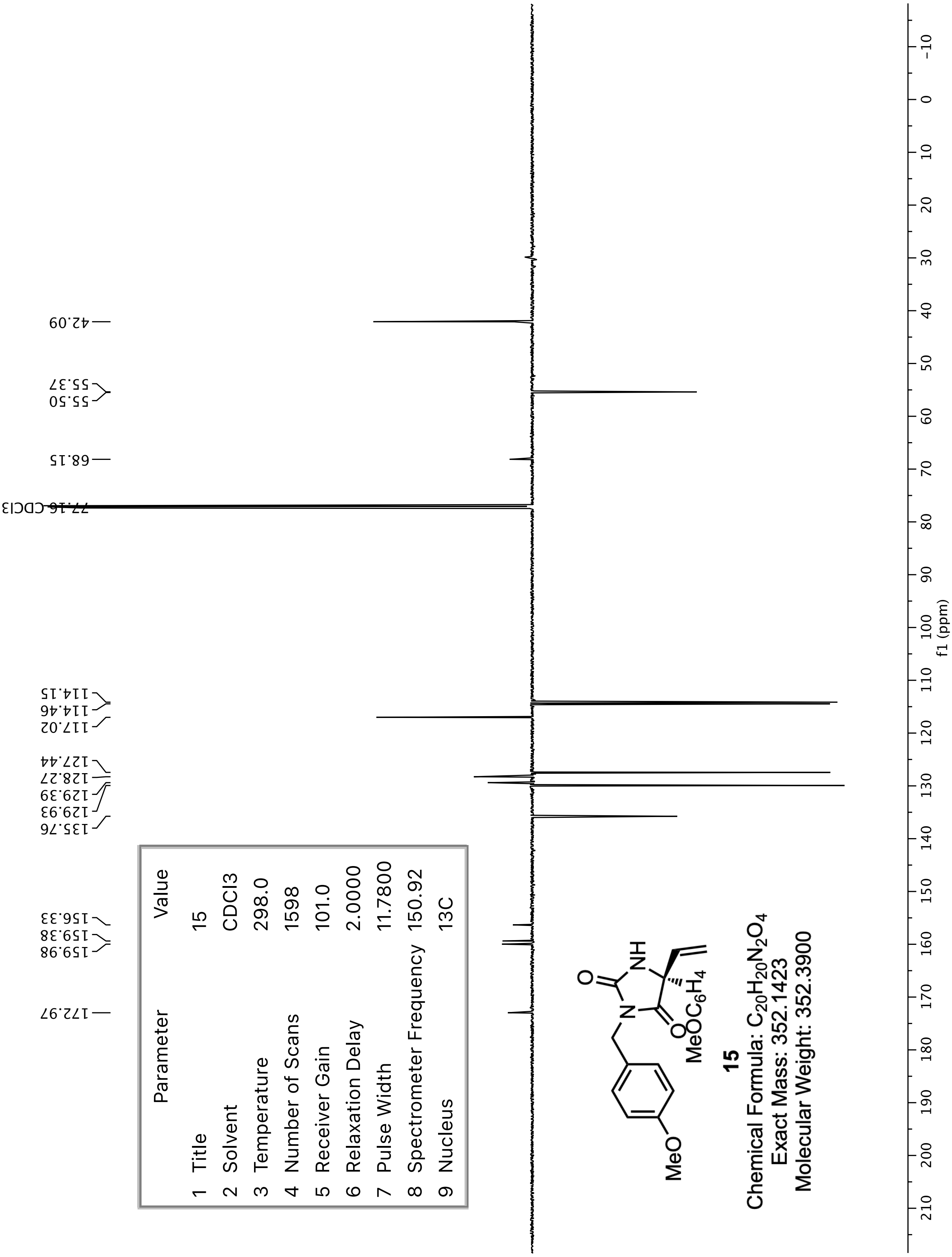


| | Parameter | Value |
|---|---|---|
| 1 | Title | 15 |
| 2 | Solvent | CDCl3 |
| 3 | Temperature | 298.0 |
| 4 | Number of Scans | 1598 |
| 5 | Receiver Gain | 101.0 |
| 6 | Relaxation Delay | 2.0000 |
| 7 | Pulse Width | 11.7800 |
| 8 | Spectrometer Frequency | 150.92 |
| 9 | Nucleus | 13C |

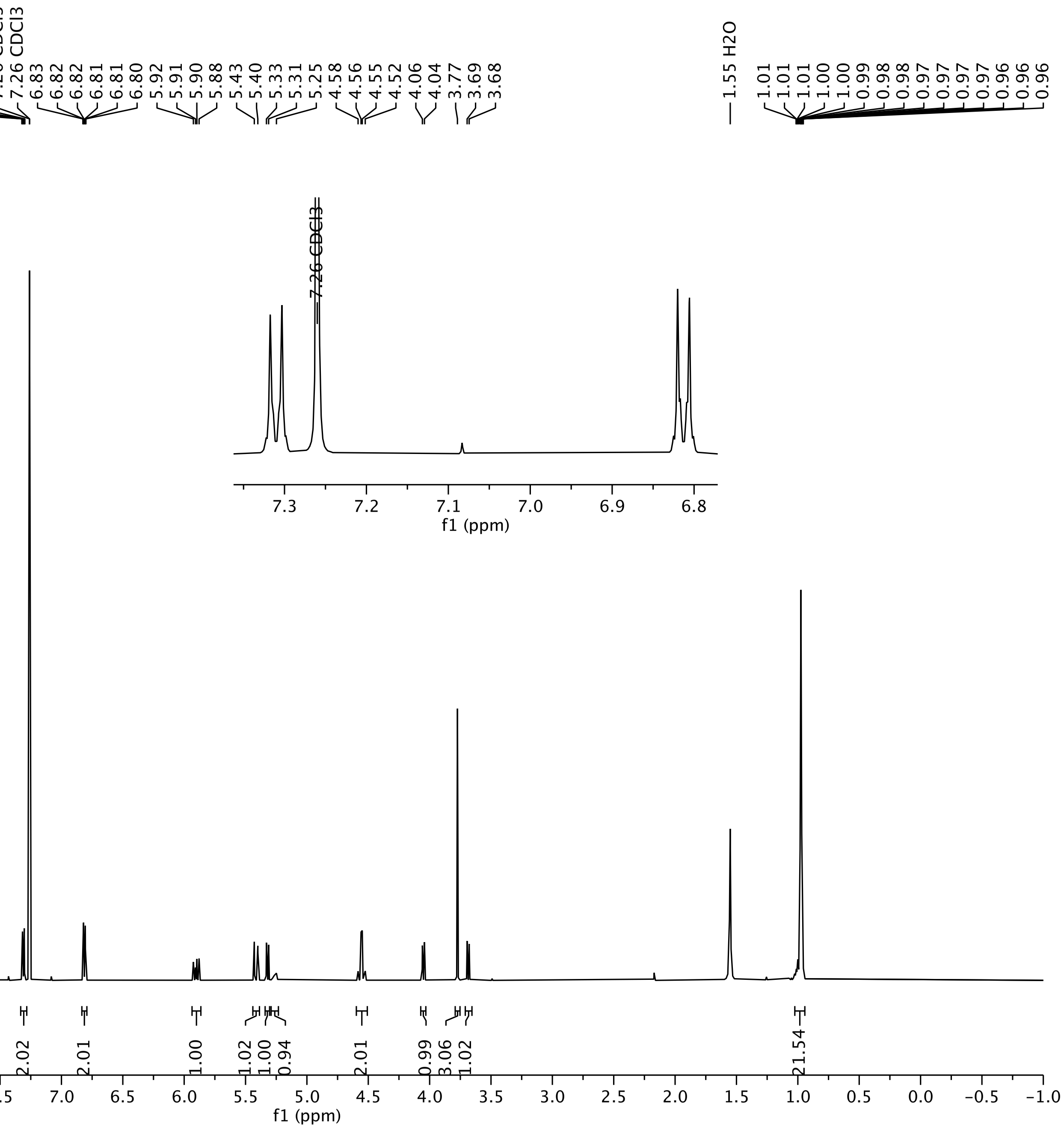


| Parameter | Value |
|---|---|
| 1 Title | 16 |
| 2 Solvent | CDCl3 |
| 3 Temperature | 298.0 |
| 4 Number of Scans | 16 |
| 5 Receiver Gain | 12.7 |
| 6 Relaxation Delay | 10.0000 |
| 7 Pulse Width | 8.0700 |
| 8 Spectrometer Frequency | 600.13 |
| 9 Nucleus | 1H |

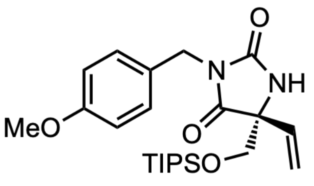


**16**

Chemical Formula: $C_{23}H_{36}N_2O_4Si$
Exact Mass: 432.2444
Molecular Weight: 432.6360

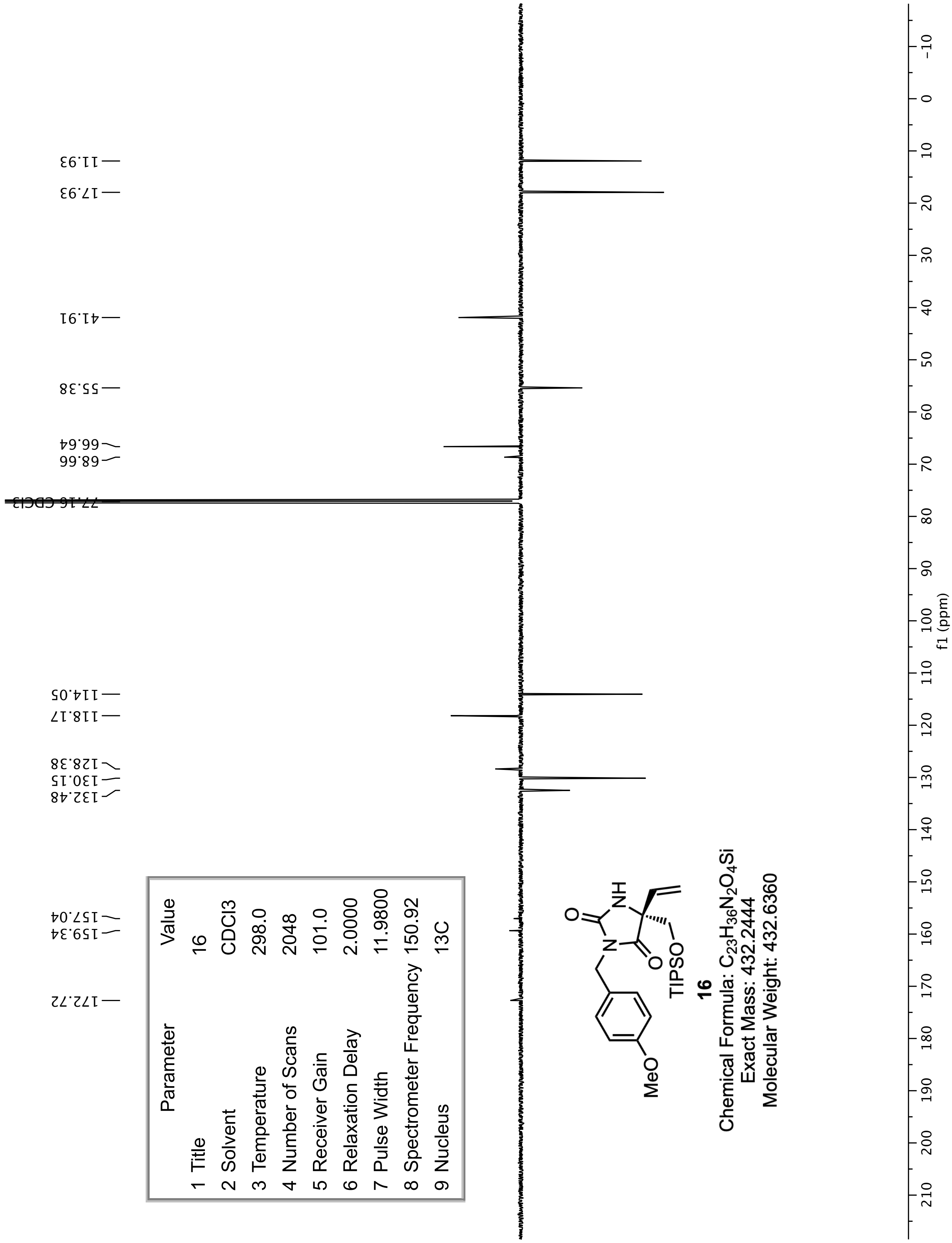


| | Parameter | Value |
|---|---|---|
| 1 | Title | 16 |
| 2 | Solvent | CDCl3 |
| 3 | Temperature | 298.0 |
| 4 | Number of Scans | 2048 |
| 5 | Receiver Gain | 101.0 |
| 6 | Relaxation Delay | 2.0000 |
| 7 | Pulse Width | 11.9800 |
| 8 | Spectrometer Frequency | 150.92 |
| 9 | Nucleus | 13C |

**16**

Chemical Formula: $C_{23}H_{36}N_2O_4Si$

Exact Mass: 432.2444

Molecular Weight: 432.6360

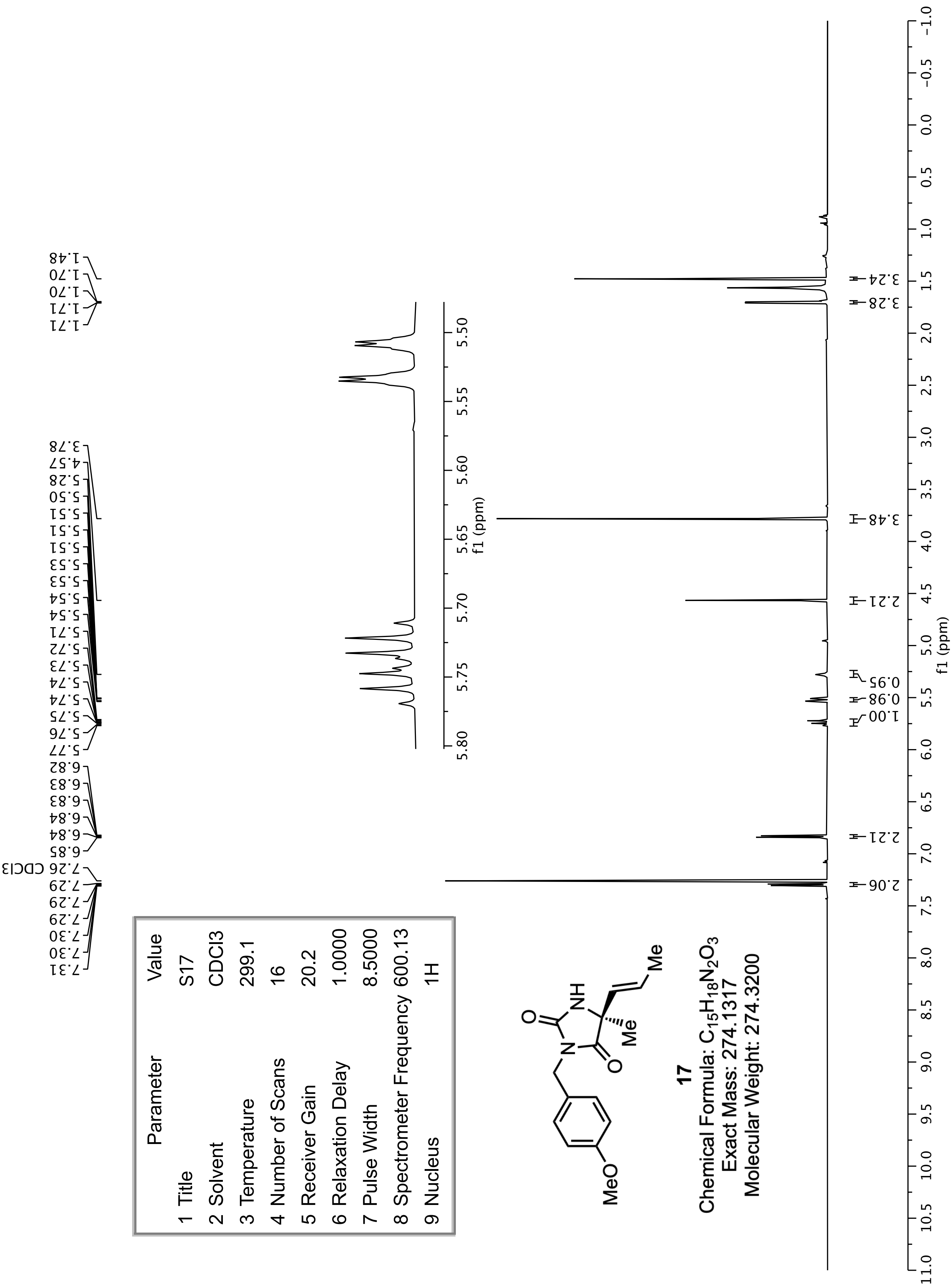


| Parameter | Value |
|---|---|
| 1 Title | S17 |
| 2 Solvent | CDCl3 |
| 3 Temperature | 299.1 |
| 4 Number of Scans | 16 |
| 5 Receiver Gain | 20.2 |
| 6 Relaxation Delay | 1.0000 |
| 7 Pulse Width | 8.5000 |
| 8 Spectrometer Frequency | 600.13 |
| 9 Nucleus | 1H |

**17**

Chemical Formula: $C_{15}H_{18}N_2O_3$
Exact Mass: 274.1317
Molecular Weight: 274.3200

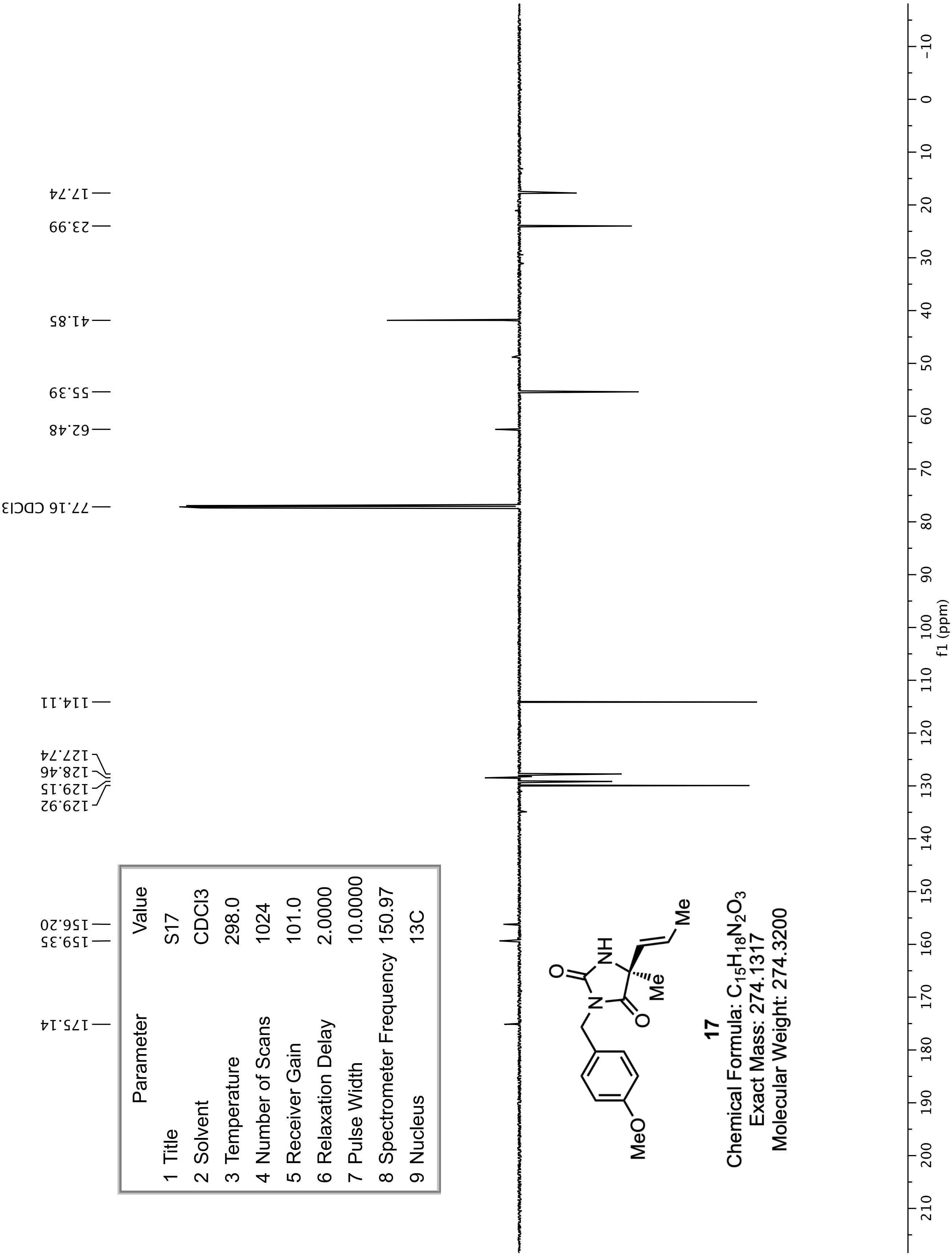


| Parameter | Value |
|---|---|
| 1 Title | S17 |
| 2 Solvent | CDCl3 |
| 3 Temperature | 298.0 |
| 4 Number of Scans | 1024 |
| 5 Receiver Gain | 101.0 |
| 6 Relaxation Delay | 2.0000 |
| 7 Pulse Width | 10.0000 |
| 8 Spectrometer Frequency | 150.97 |
| 9 Nucleus | 13C |

**17**
Chemical Formula: $C_{15}H_{18}N_2O_3$
Exact Mass: 274.1317
Molecular Weight: 274.3200

| Parameter | Value |
|---|---|
| 1 Title | 18 |
| 2 Solvent | CDCl3 |
| 3 Temperature | 298.1 |
| 4 Number of Scans | 16 |
| 5 Receiver Gain | 32.0 |
| 6 Relaxation Delay | 10.0000 |
| 7 Pulse Width | 12.0000 |
| 8 Spectrometer Frequency | 600.32 |
| 9 Nucleus | 1H |

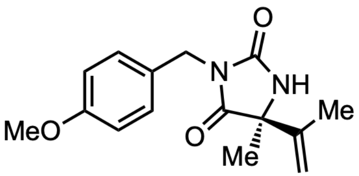


**18**
Chemical Formula: $C_{15}H_{18}N_2O_3$
Exact Mass: 274.1317
Molecular Weight: 274.3200

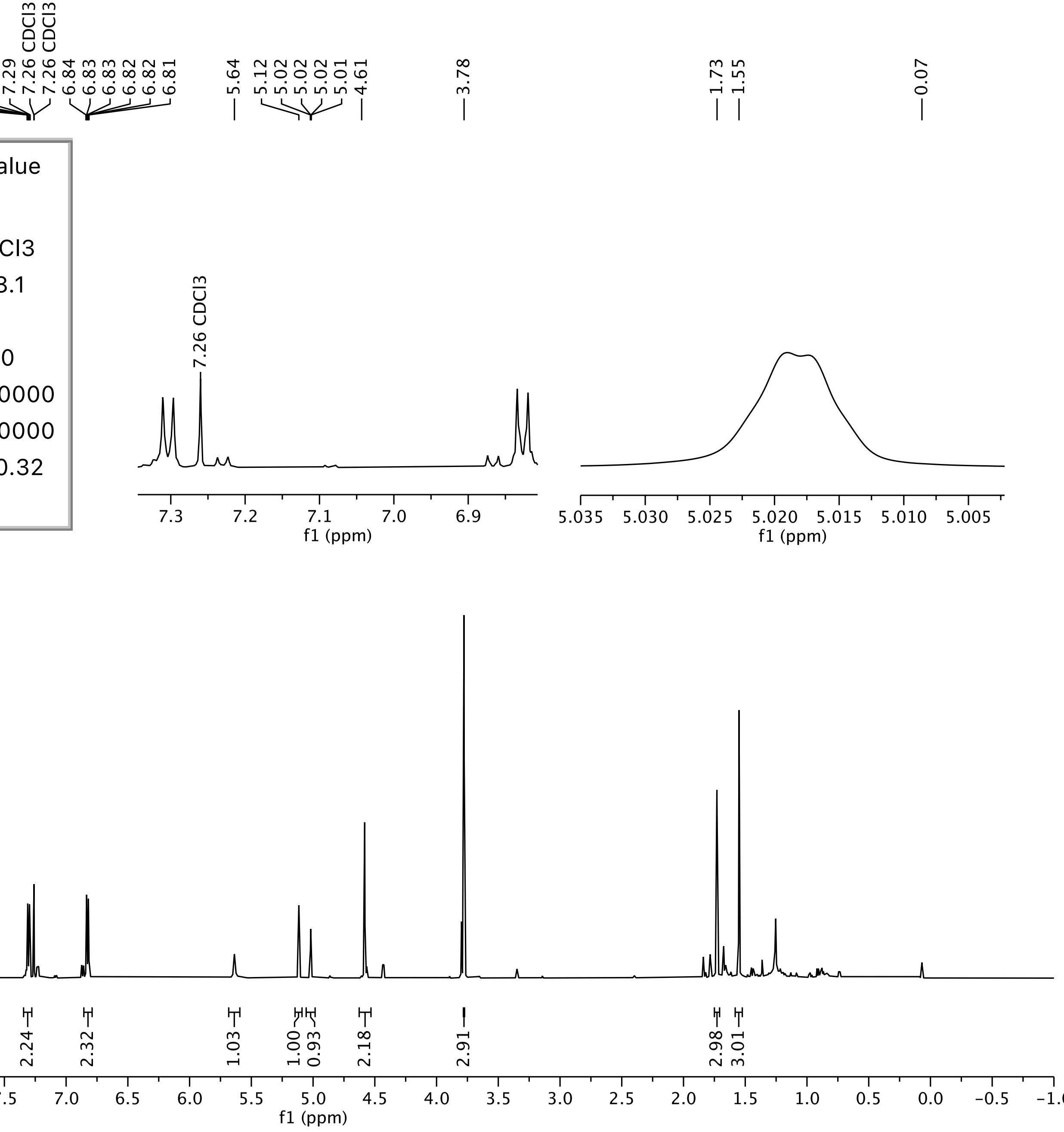

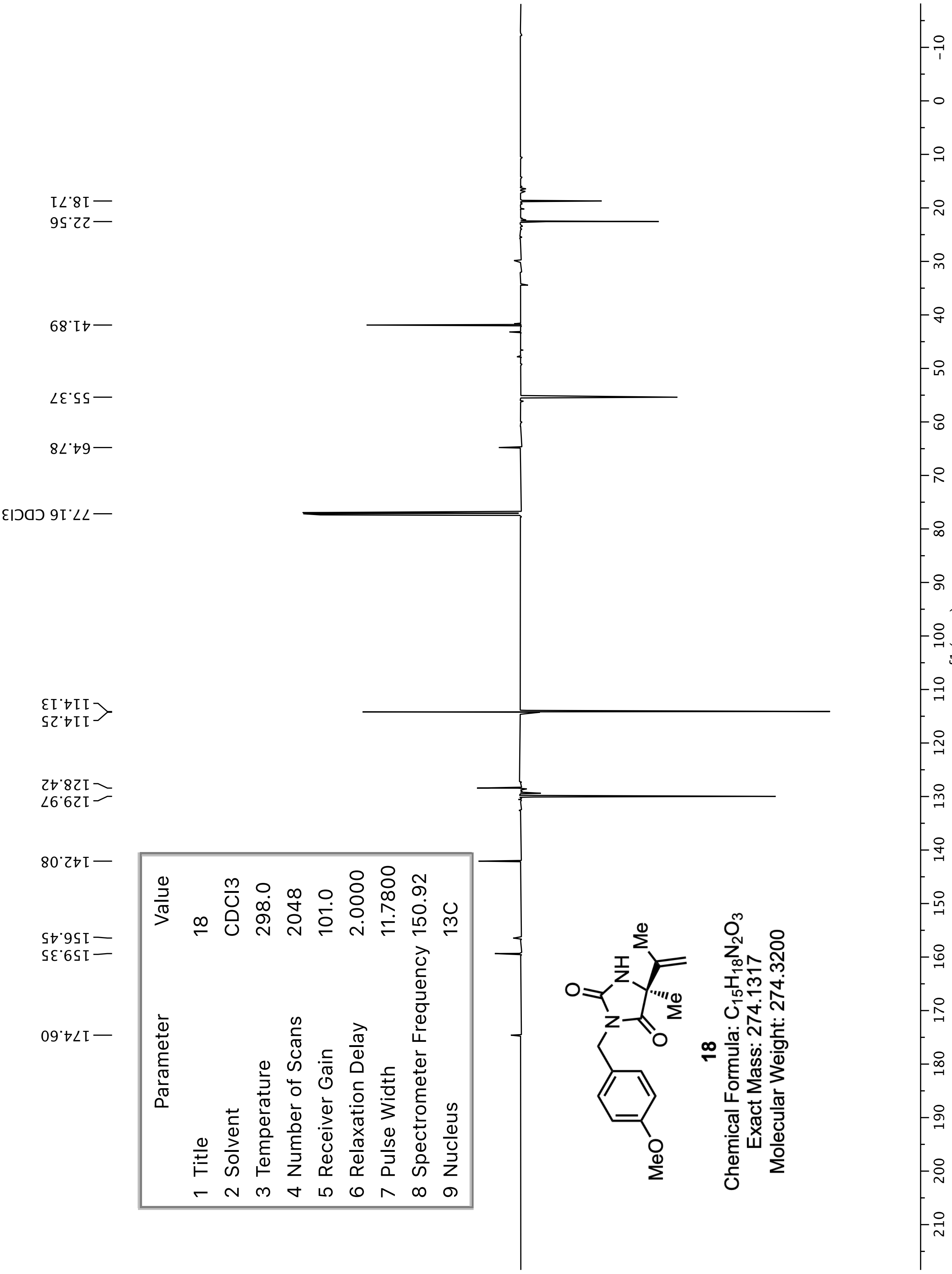


| | Parameter | Value |
|---|---|---|
| 1 | Title | 18 |
| 2 | Solvent | CDCl3 |
| 3 | Temperature | 298.0 |
| 4 | Number of Scans | 2048 |
| 5 | Receiver Gain | 101.0 |
| 6 | Relaxation Delay | 2.0000 |
| 7 | Pulse Width | 11.7800 |
| 8 | Spectrometer Frequency | 150.92 |
| 9 | Nucleus | 13C |

**18**

Chemical Formula: $C_{15}H_{18}N_2O_3$

Exact Mass: 274.1317

Molecular Weight: 274.3200

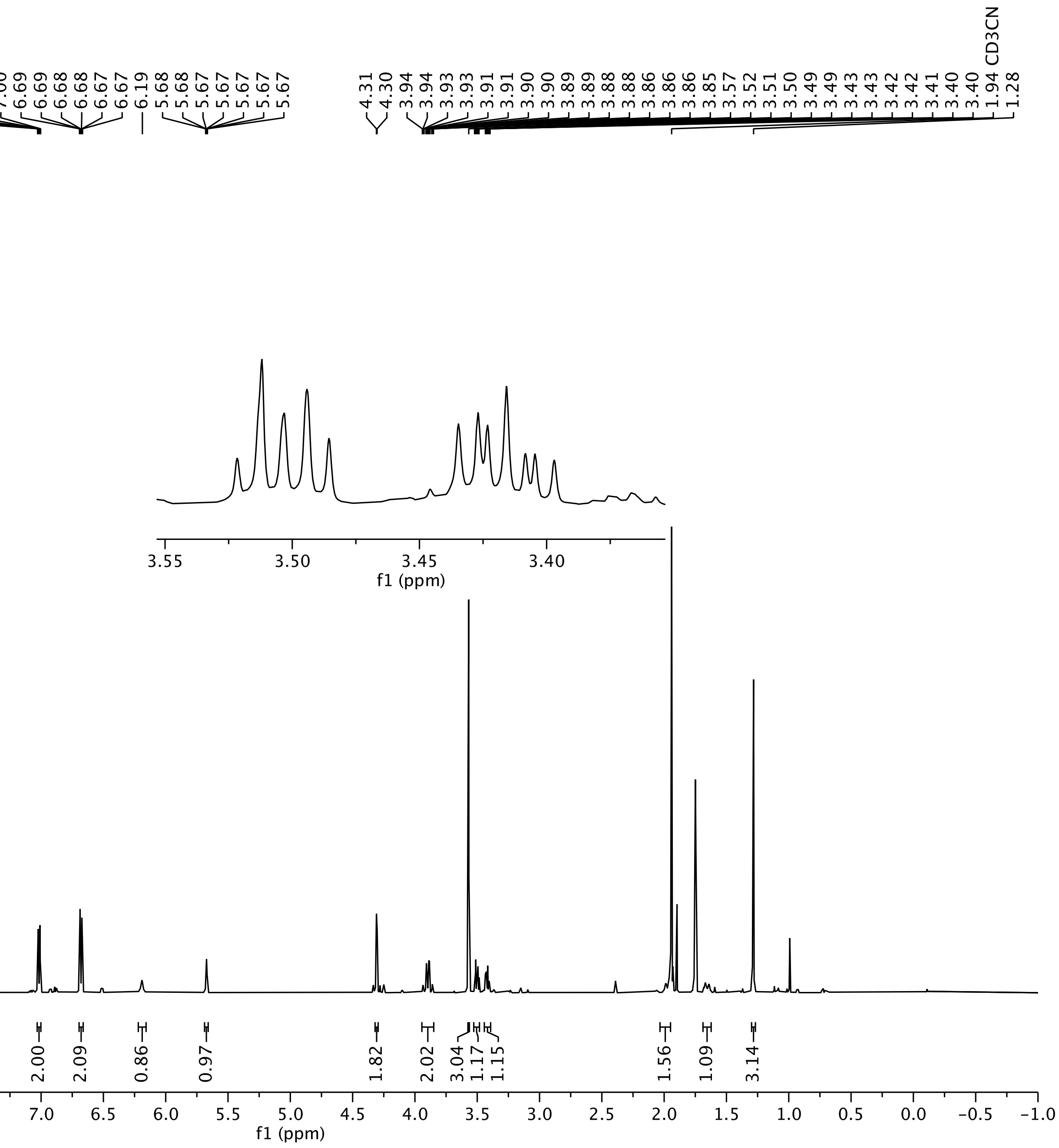


| | Parameter | Value |
|---|---|---|
| 1 | Title | 19 |
| 2 | Solvent | CD3CN |
| 3 | Temperature | 300.0 |
| 4 | Number of Scans | 16 |
| 5 | Receiver Gain | 64.0 |
| 6 | Relaxation Delay | 10.0000 |
| 7 | Pulse Width | 12.0000 |
| 8 | Spectrometer Frequency | 600.32 |
| 9 | Nucleus | 1H |

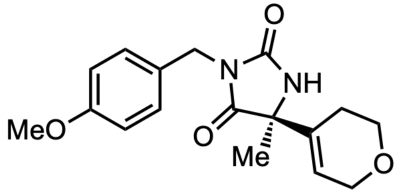


**19**
Chemical Formula: $C_{17}H_{20}N_2O_4$
Exact Mass: 316.1423
Molecular Weight: 316.3570

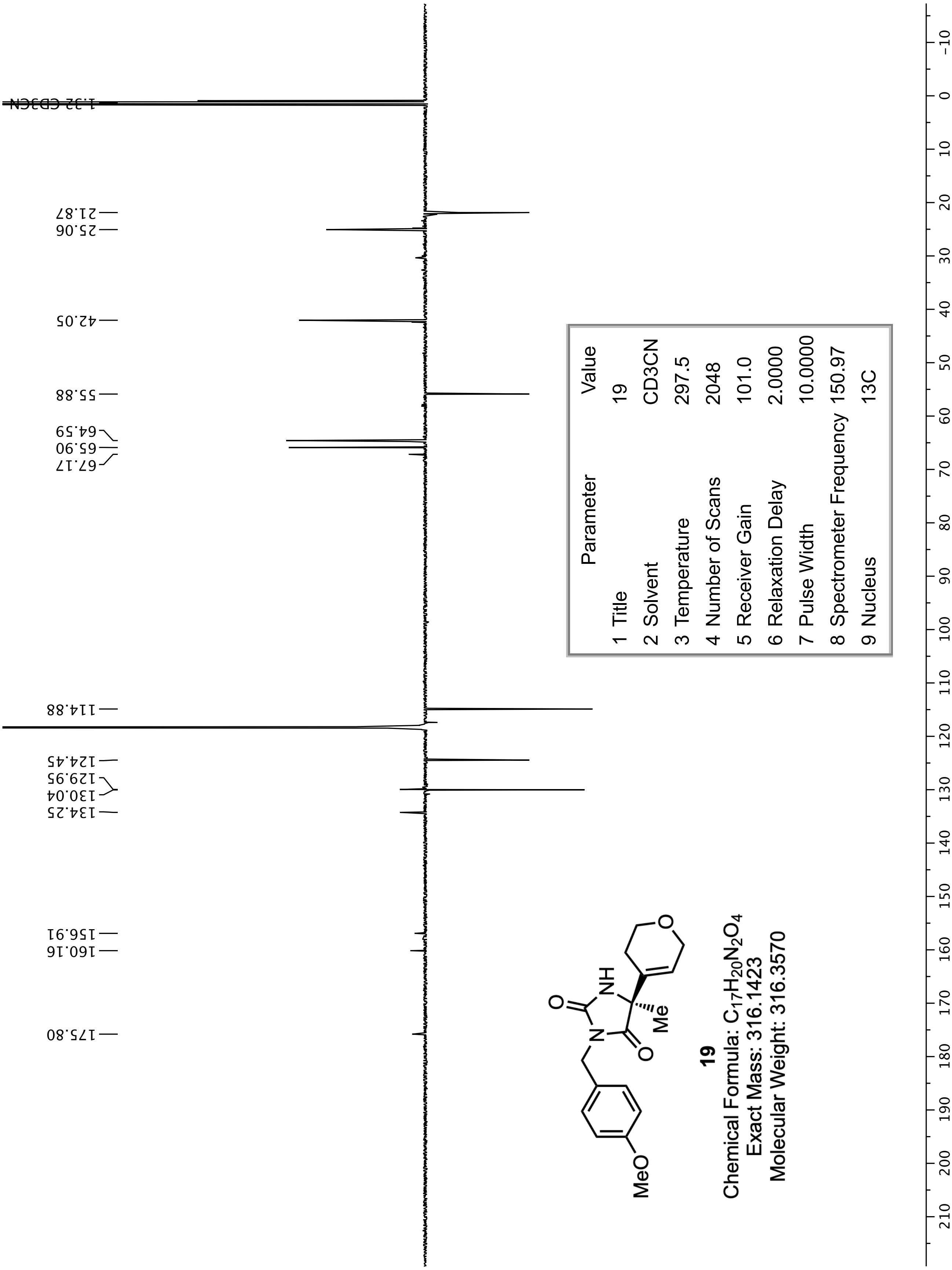


| | Parameter | Value |
|---|---|---|
| 1 | Title | 19 |
| 2 | Solvent | CD3CN |
| 3 | Temperature | 297.5 |
| 4 | Number of Scans | 2048 |
| 5 | Receiver Gain | 101.0 |
| 6 | Relaxation Delay | 2.0000 |
| 7 | Pulse Width | 10.0000 |
| 8 | Spectrometer Frequency | 150.97 |
| 9 | Nucleus | 13C |

**19**

Chemical Formula: $C_{17}H_{20}N_2O_4$

Exact Mass: 316.1423

Molecular Weight: 316.3570

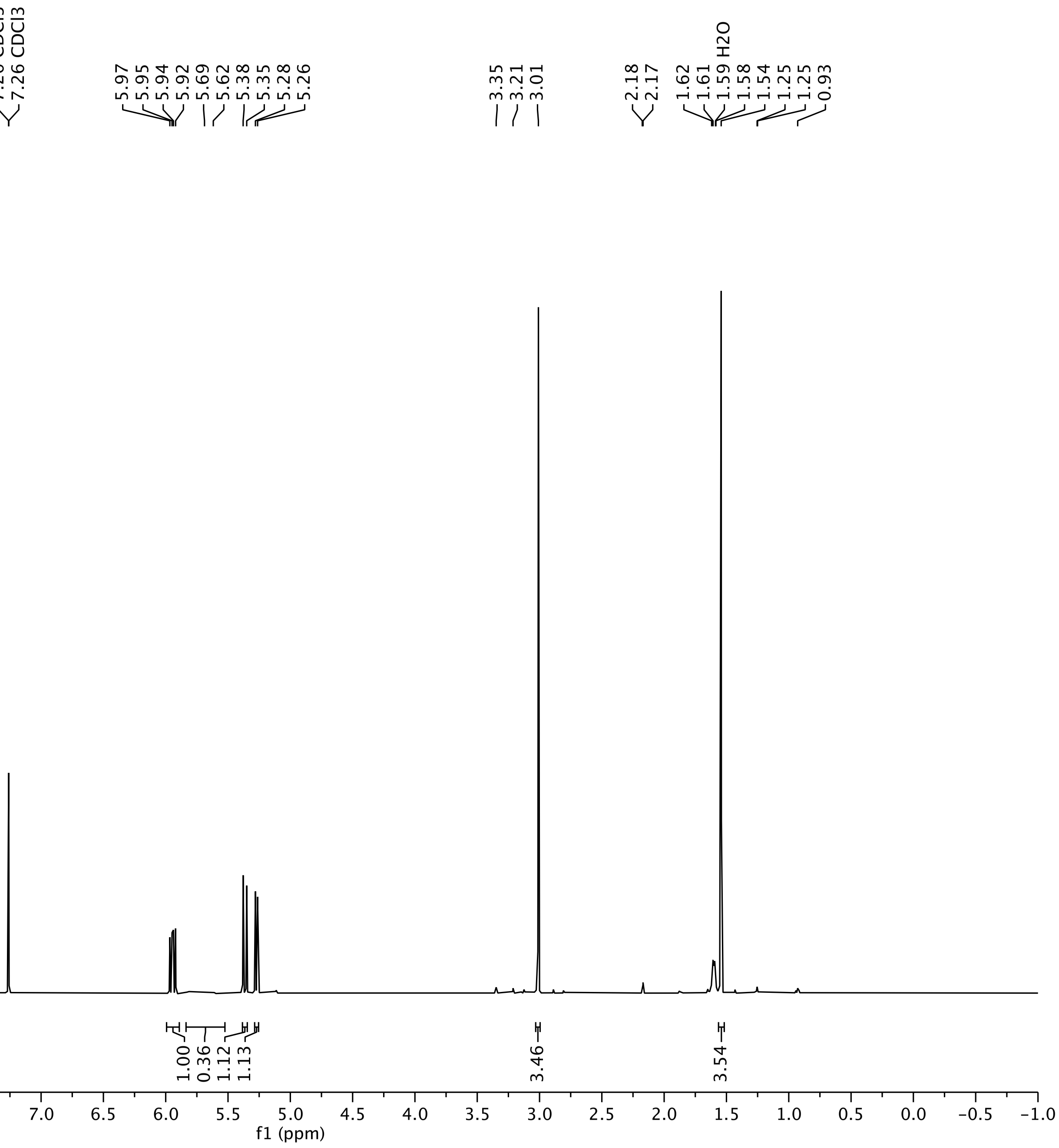


| | Parameter | Value |
|---|---|---|
| 1 | Title | 21 |
| 2 | Solvent | CDCl3 |
| 3 | Temperature | 298.0 |
| 4 | Number of Scans | 16 |
| 5 | Receiver Gain | 64.0 |
| 6 | Relaxation Delay | 10.0000 |
| 7 | Pulse Width | 12.0000 |
| 8 | Spectrometer Frequency | 600.32 |
| 9 | Nucleus | 1H |

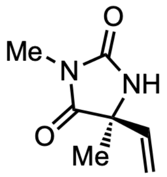


**21**

Chemical Formula: $C_7H_{10}N_2O_2$
Exact Mass: 154.0742
Molecular Weight: 154.1690

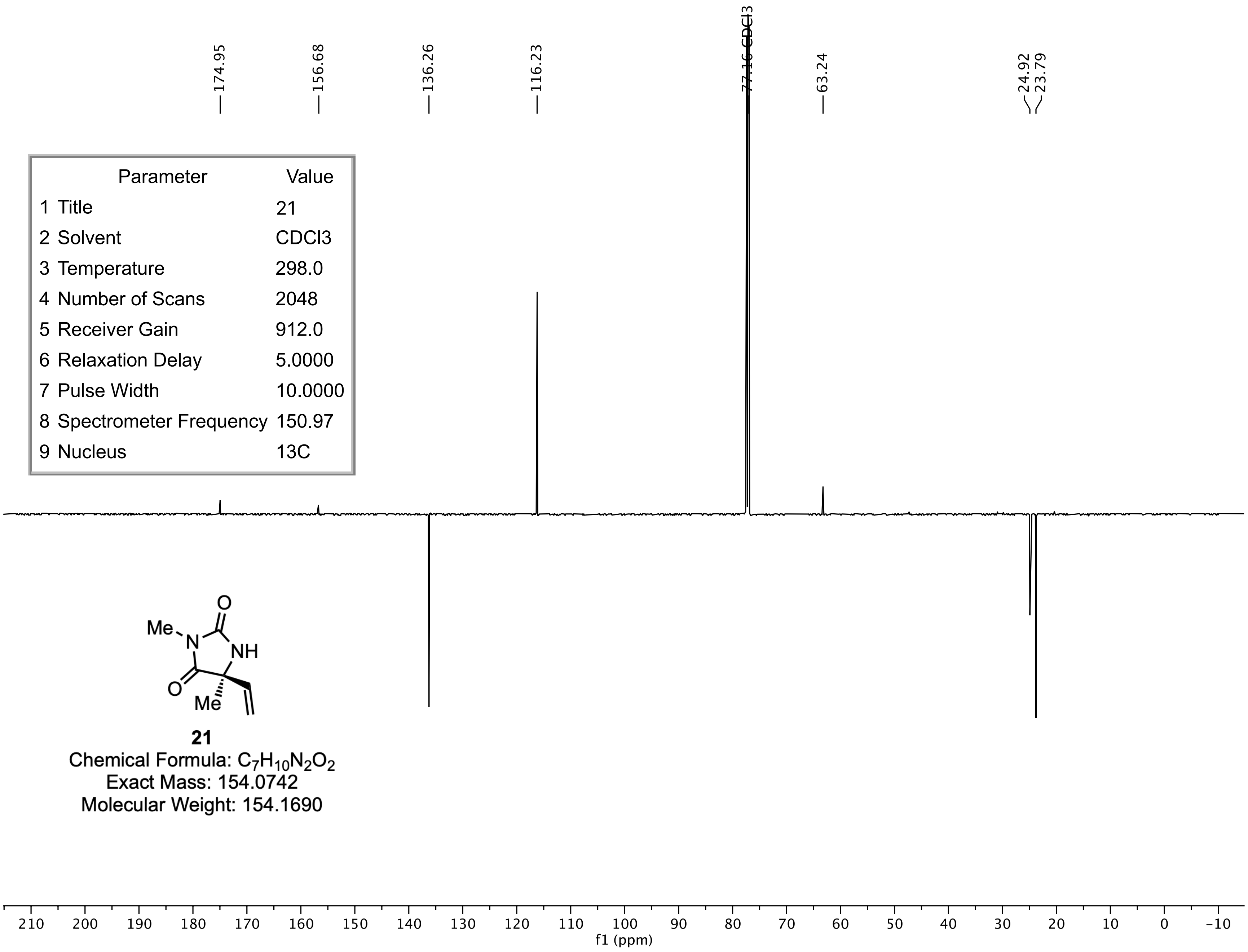


| | Parameter | Value |
|---|---|---|
| 1 | Title | 21 |
| 2 | Solvent | CDCl3 |
| 3 | Temperature | 298.0 |
| 4 | Number of Scans | 2048 |
| 5 | Receiver Gain | 912.0 |
| 6 | Relaxation Delay | 5.0000 |
| 7 | Pulse Width | 10.0000 |
| 8 | Spectrometer Frequency | 150.97 |
| 9 | Nucleus | 13C |



**21**
Chemical Formula: $C_7H_{10}N_2O_2$
Exact Mass: 154.0742
Molecular Weight: 154.1690

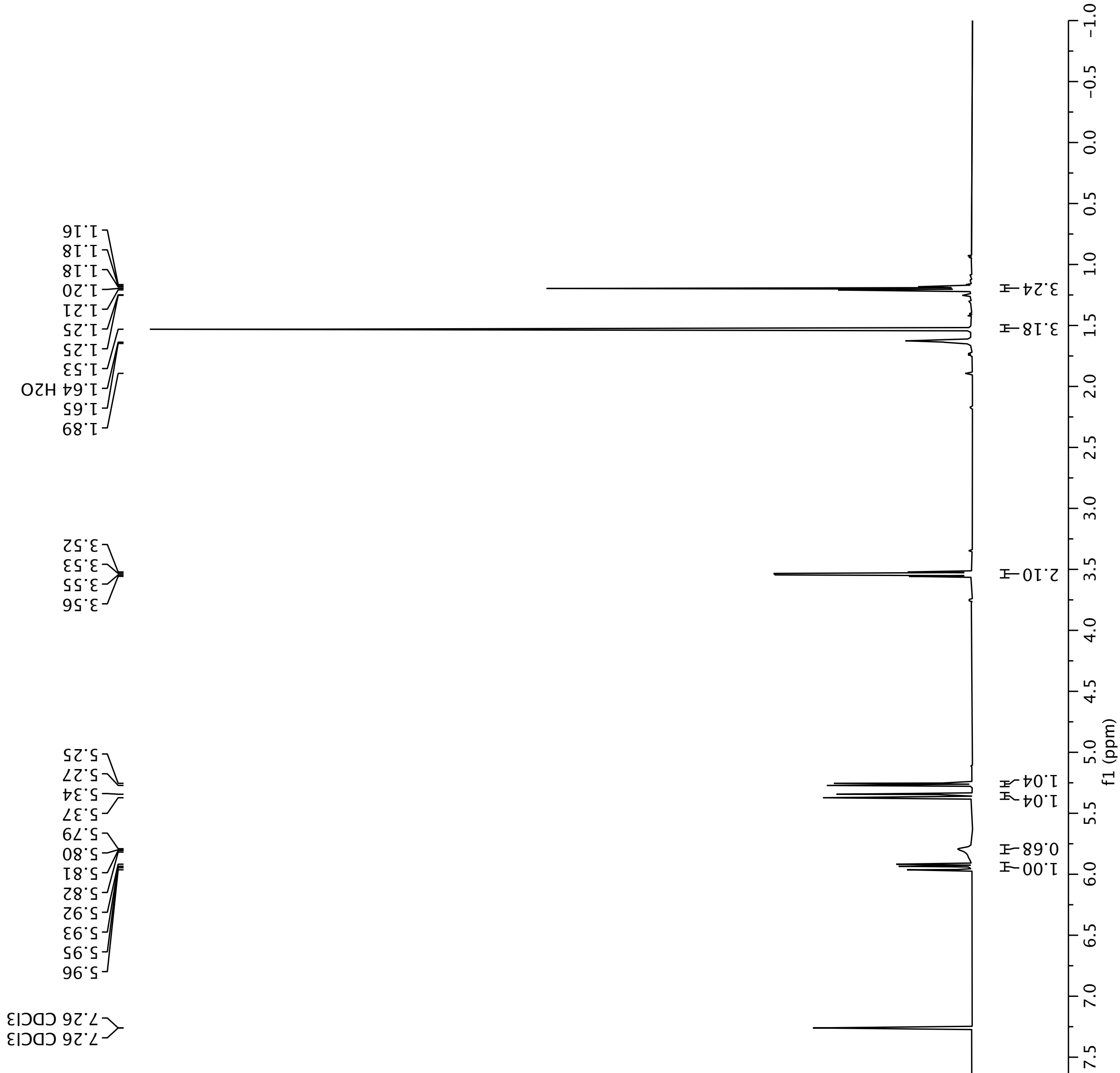


| | Parameter | Value |
|---|---|---|
| 1 | Title | 22 |
| 2 | Solvent | CDCl3 |
| 3 | Temperature | 298.1 |
| 4 | Number of Scans | 16 |
| 5 | Receiver Gain | 12.7 |
| 6 | Relaxation Delay | 10.0000 |
| 7 | Pulse Width | 8.0700 |
| 8 | Spectrometer Frequency | 600.13 |
| 9 | Nucleus | 1H |

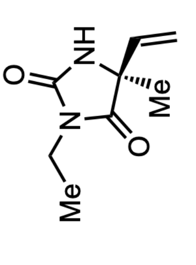


**22**

Chemical Formula: $C_8H_{12}N_2O_2$
Exact Mass: 168.0899
Molecular Weight: 168.1960

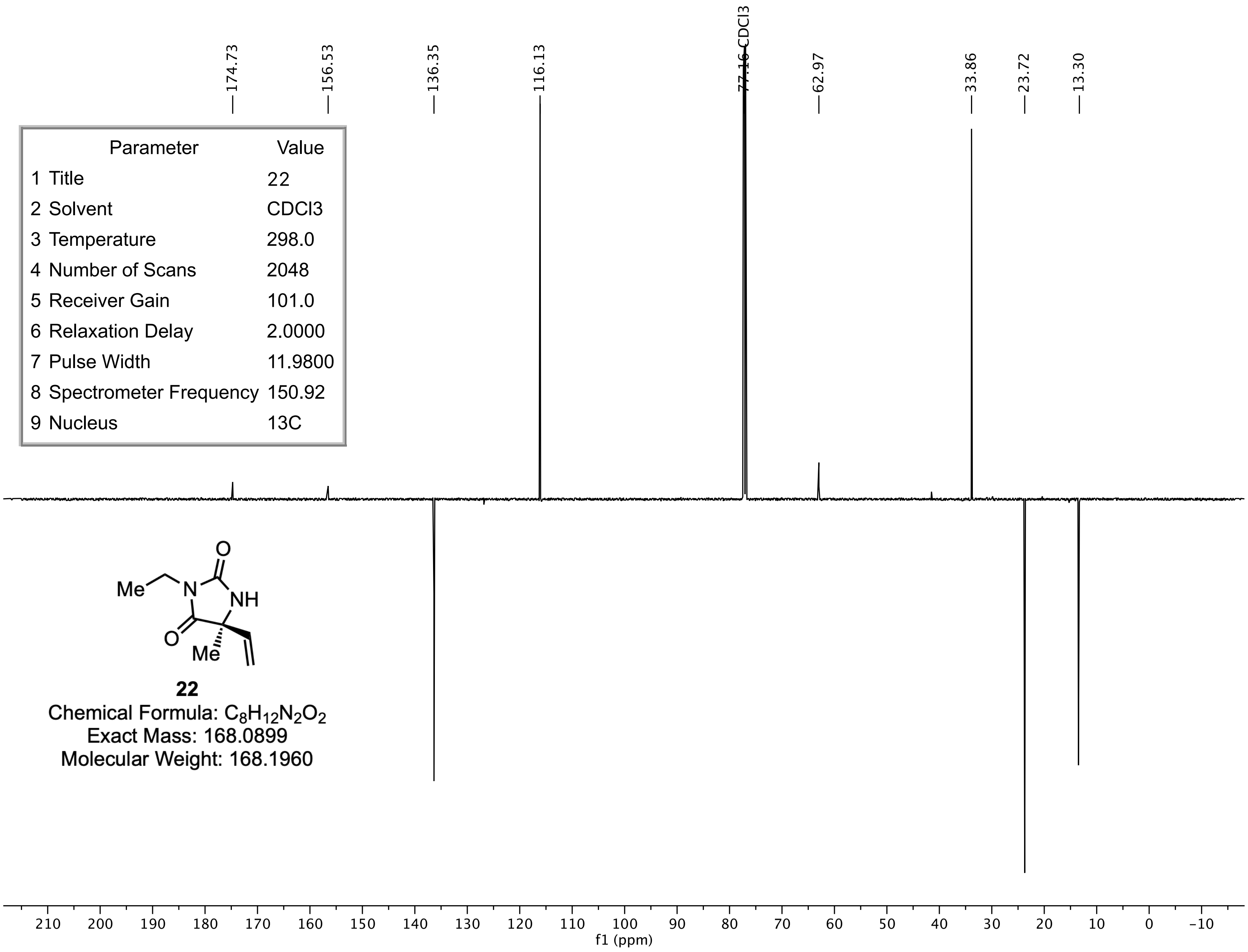


| Parameter | Value |
|---|---|
| 1 Title | 22 |
| 2 Solvent | CDCl3 |
| 3 Temperature | 298.0 |
| 4 Number of Scans | 2048 |
| 5 Receiver Gain | 101.0 |
| 6 Relaxation Delay | 2.0000 |
| 7 Pulse Width | 11.9800 |
| 8 Spectrometer Frequency | 150.92 |
| 9 Nucleus | 13C |

**22**

Chemical Formula: $C_8H_{12}N_2O_2$

Exact Mass: 168.0899

Molecular Weight: 168.1960

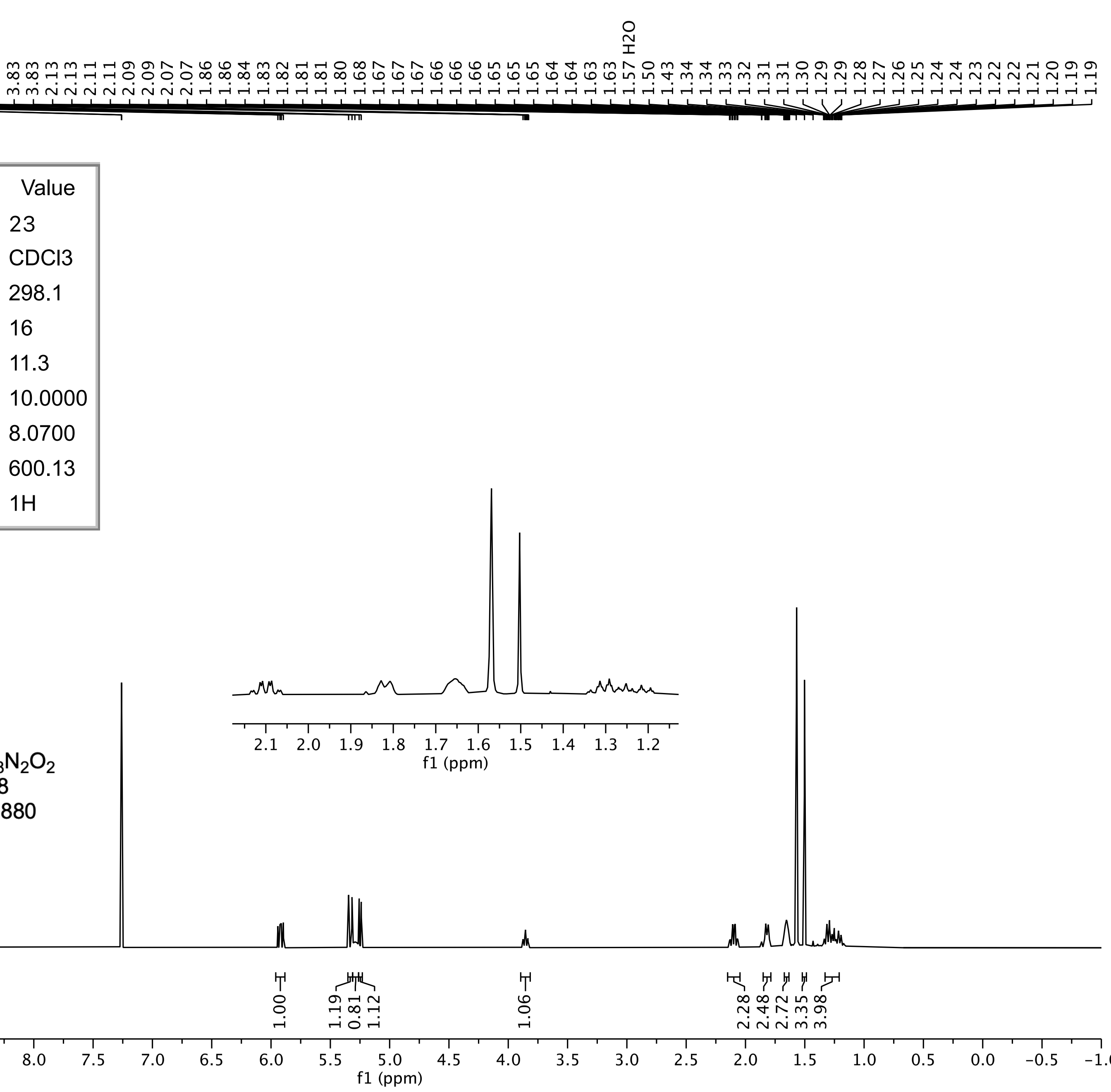


| | Parameter | Value |
|---|---|---|
| 1 | Title | 23 |
| 2 | Solvent | CDCl3 |
| 3 | Temperature | 298.1 |
| 4 | Number of Scans | 16 |
| 5 | Receiver Gain | 11.3 |
| 6 | Relaxation Delay | 10.0000 |
| 7 | Pulse Width | 8.0700 |
| 8 | Spectrometer Frequency | 600.13 |
| 9 | Nucleus | 1H |

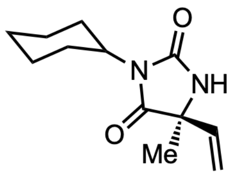


**23**

Chemical Formula: $C_{12}H_{18}N_2O_2$

Exact Mass: 222.1368

Molecular Weight: 222.2880

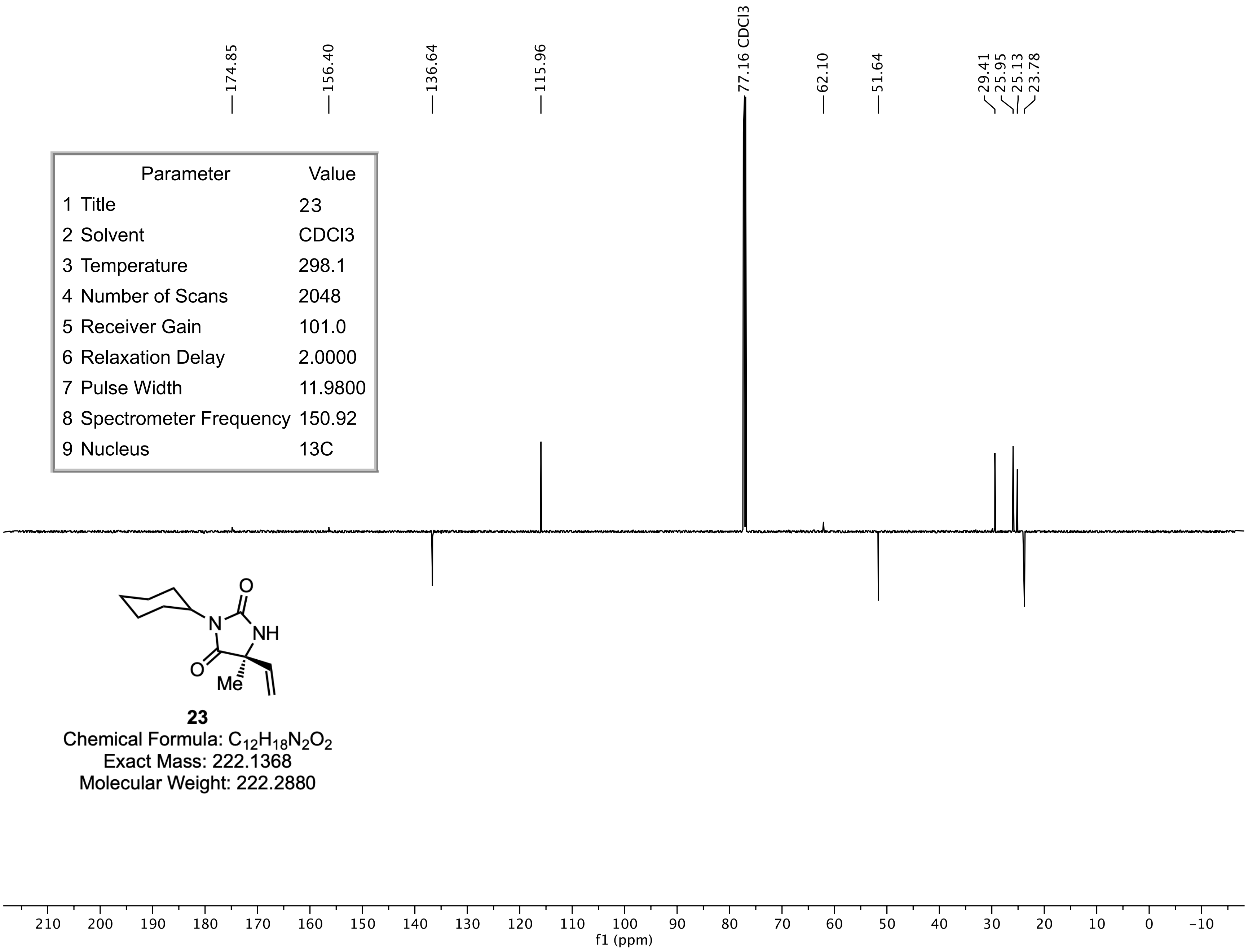


| Parameter | Value |
|---|---|
| 1 Title | 23 |
| 2 Solvent | CDCl3 |
| 3 Temperature | 298.1 |
| 4 Number of Scans | 2048 |
| 5 Receiver Gain | 101.0 |
| 6 Relaxation Delay | 2.0000 |
| 7 Pulse Width | 11.9800 |
| 8 Spectrometer Frequency | 150.92 |
| 9 Nucleus | 13C |



**23**

Chemical Formula: $C_{12}H_{18}N_2O_2$

Exact Mass: 222.1368

Molecular Weight: 222.2880

| | Parameter | Value |
|---|---|---|
| 1 | Title | 24 |
| 2 | Solvent | CDCl3 |
| 3 | Temperature | 298.0 |
| 4 | Number of Scans | 16 |
| 5 | Receiver Gain | 71.8 |
| 6 | Relaxation Delay | 10.0000 |
| 7 | Pulse Width | 12.0000 |
| 8 | Spectrometer Frequency | 600.32 |
| 9 | Nucleus | 1H |

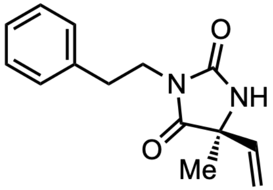

**24**

Chemical Formula: $C_{14}H_{16}N_2O_2$
Exact Mass: 244.1212
Molecular Weight: 244.2940

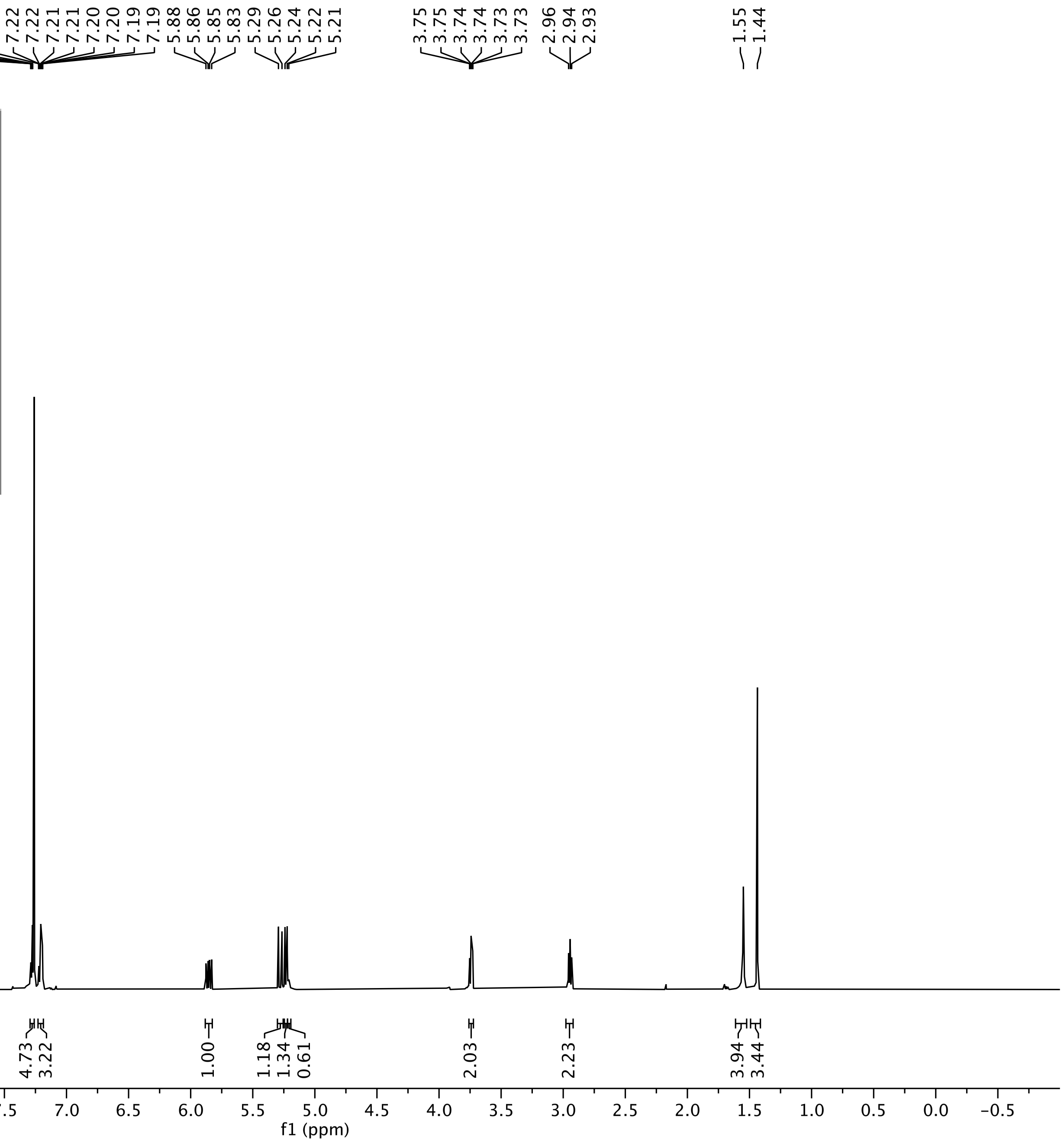

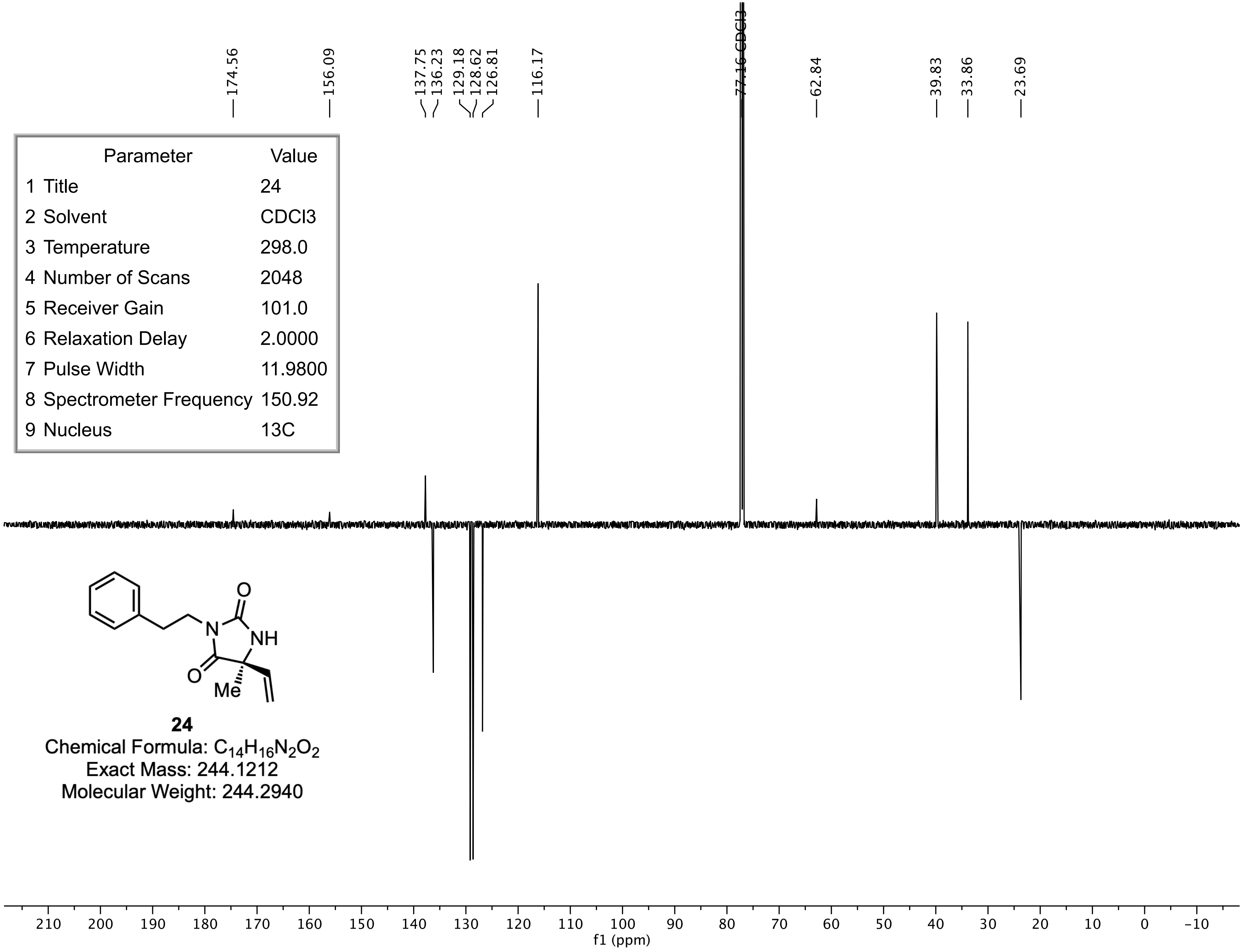


| Parameter | Value |
|---|---|
| 1 Title | 24 |
| 2 Solvent | CDCl3 |
| 3 Temperature | 298.0 |
| 4 Number of Scans | 2048 |
| 5 Receiver Gain | 101.0 |
| 6 Relaxation Delay | 2.0000 |
| 7 Pulse Width | 11.9800 |
| 8 Spectrometer Frequency | 150.92 |
| 9 Nucleus | 13C |

**24**
Chemical Formula: $C_{14}H_{16}N_2O_2$
Exact Mass: 244.1212
Molecular Weight: 244.2940

| Parameter | Value |
|---|---|
| 1 Title | 25 |
| 2 Solvent | CDCl3 |
| 3 Temperature | 298.0 |
| 4 Number of Scans | 16 |
| 5 Receiver Gain | 32.0 |
| 6 Relaxation Delay | 10.0000 |
| 7 Pulse Width | 12.0000 |
| 8 Spectrometer Frequency | 600.32 |
| 9 Nucleus | 1H |

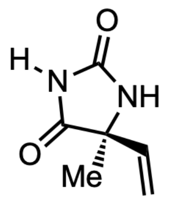


**(*S*)-25**
Chemical Formula: $C_6H_8N_2O_2$
Exact Mass: 140.0586
Molecular Weight: 140.1420

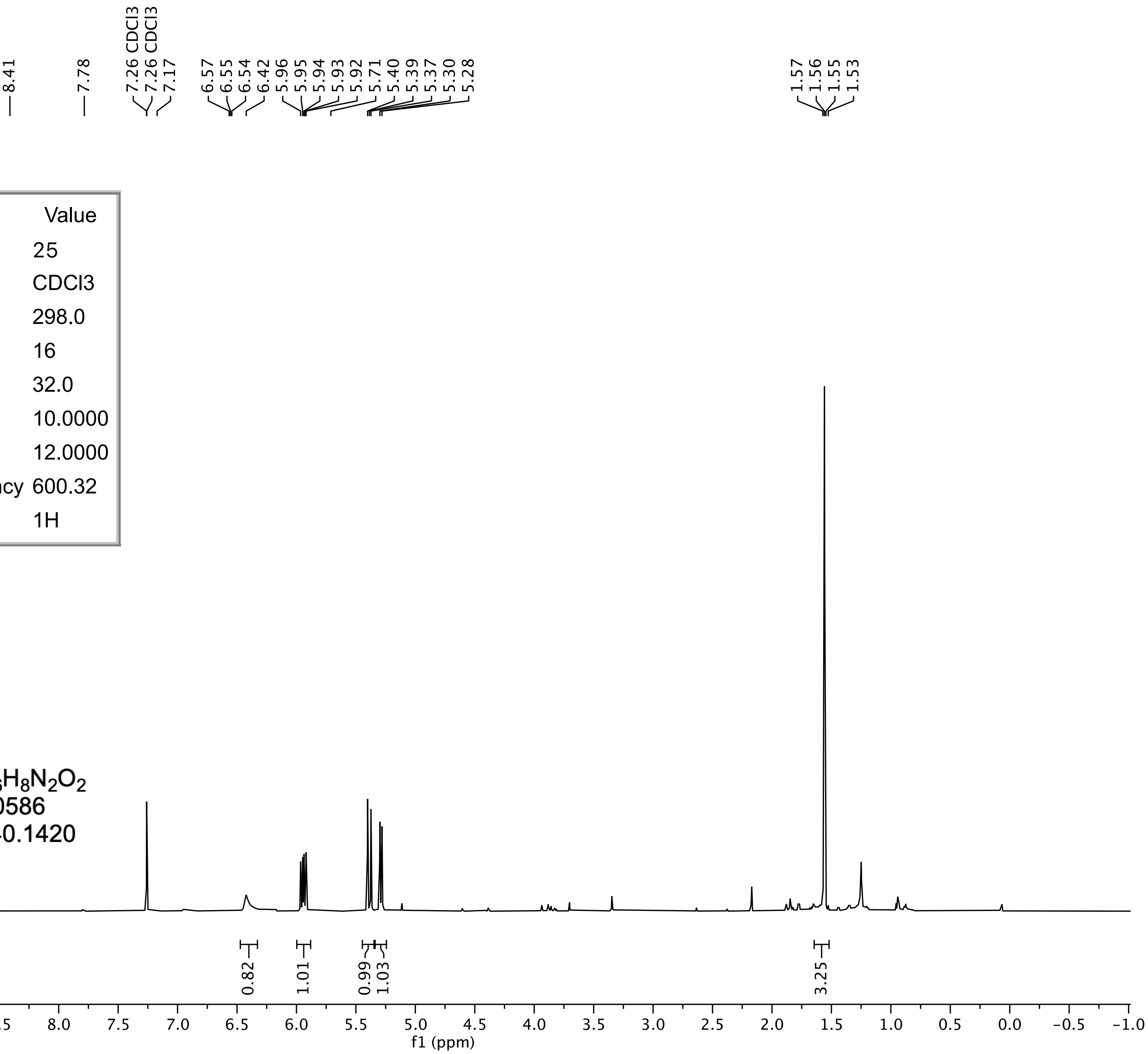

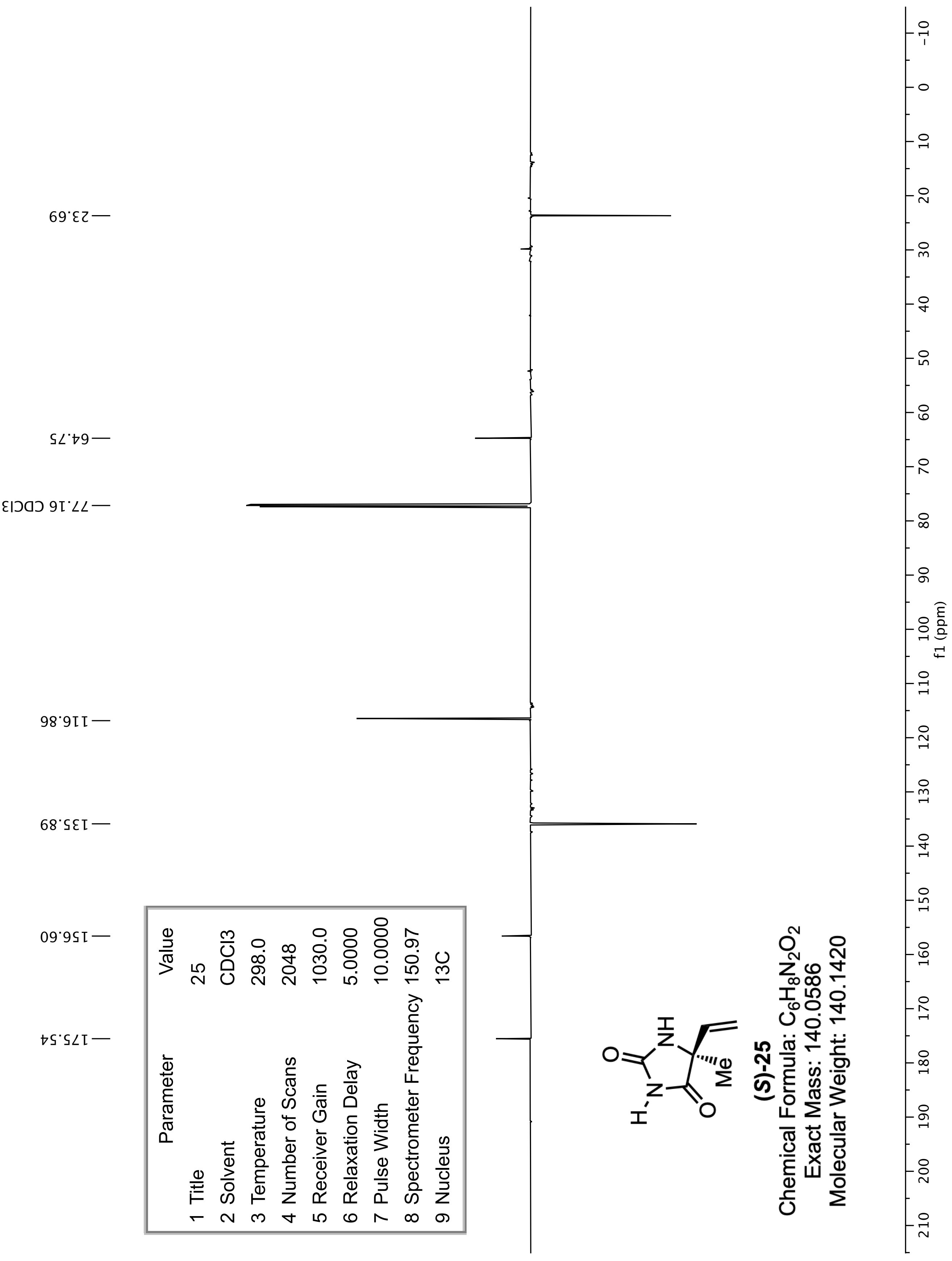

175.54
156.60
135.89
116.86
77.16 CDCl3
64.75
23.69
Parameter Value
1 Title 25
2 Solvent CDCl3
3 Temperature 298.0
4 Number of Scans 2048
5 Receiver Gain 1030.0
6 Relaxation Delay 5.0000
7 Pulse Width 10.0000
8 Spectrometer Frequency 150.97
9 Nucleus 13C
(S)-25
Chemical Formula: C6H8N2O2
Exact Mass: 140.0586
Molecular Weight: 140.1420
f1 (ppm)

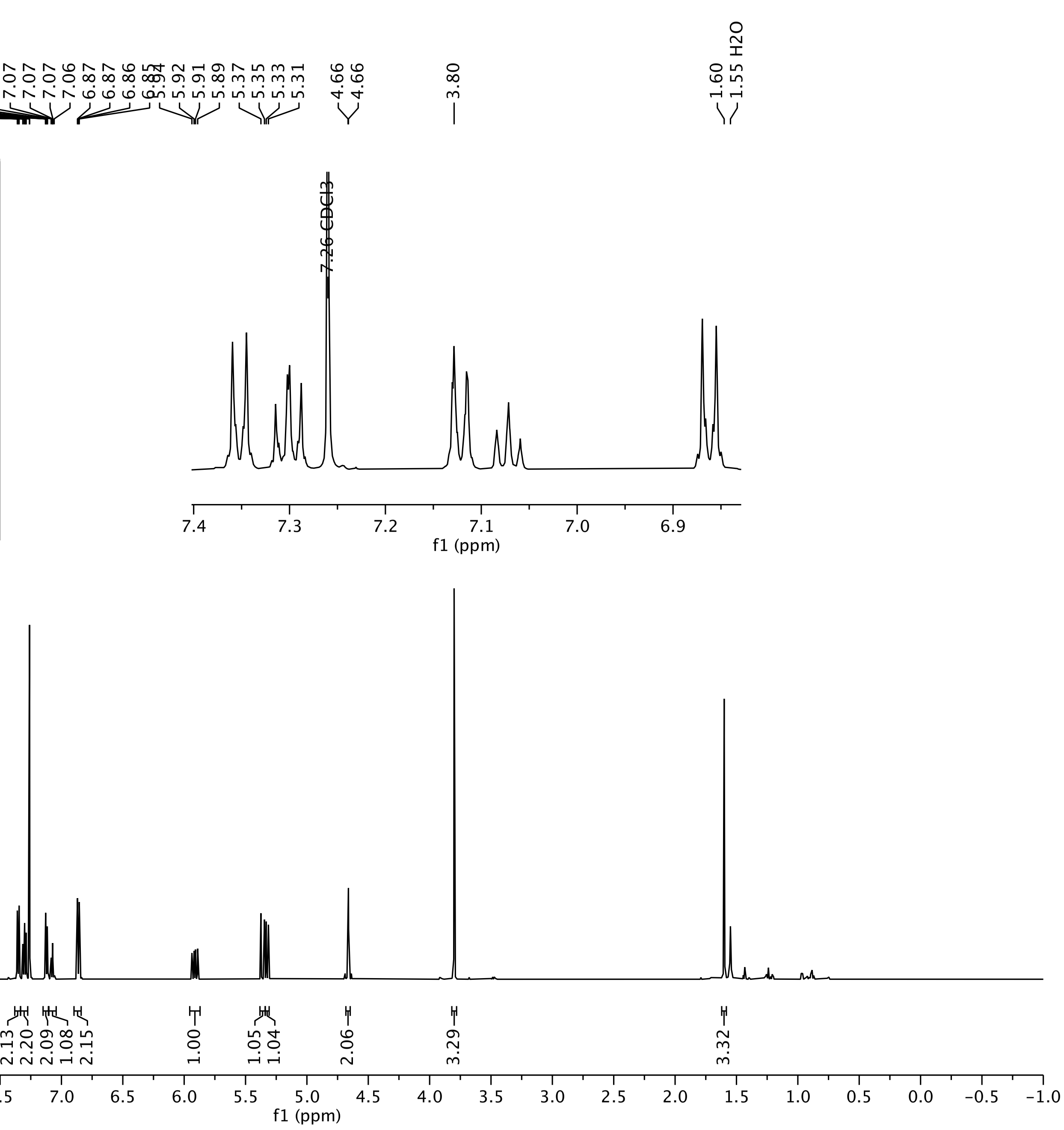


| Parameter | Value |
|---|---|
| 1 Title | 26 |
| 2 Solvent | CDCl3 |
| 3 Temperature | 298.0 |
| 4 Number of Scans | 16 |
| 5 Receiver Gain | 90.5 |
| 6 Relaxation Delay | 1.0000 |
| 7 Pulse Width | 11.2250 |
| 8 Spectrometer Frequency | 600.32 |
| 9 Nucleus | 1H |

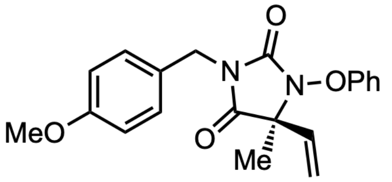


**26**

Chemical Formula: $C_{20}H_{20}N_2O_4$

Exact Mass: 352.1423

Molecular Weight: 352.3900

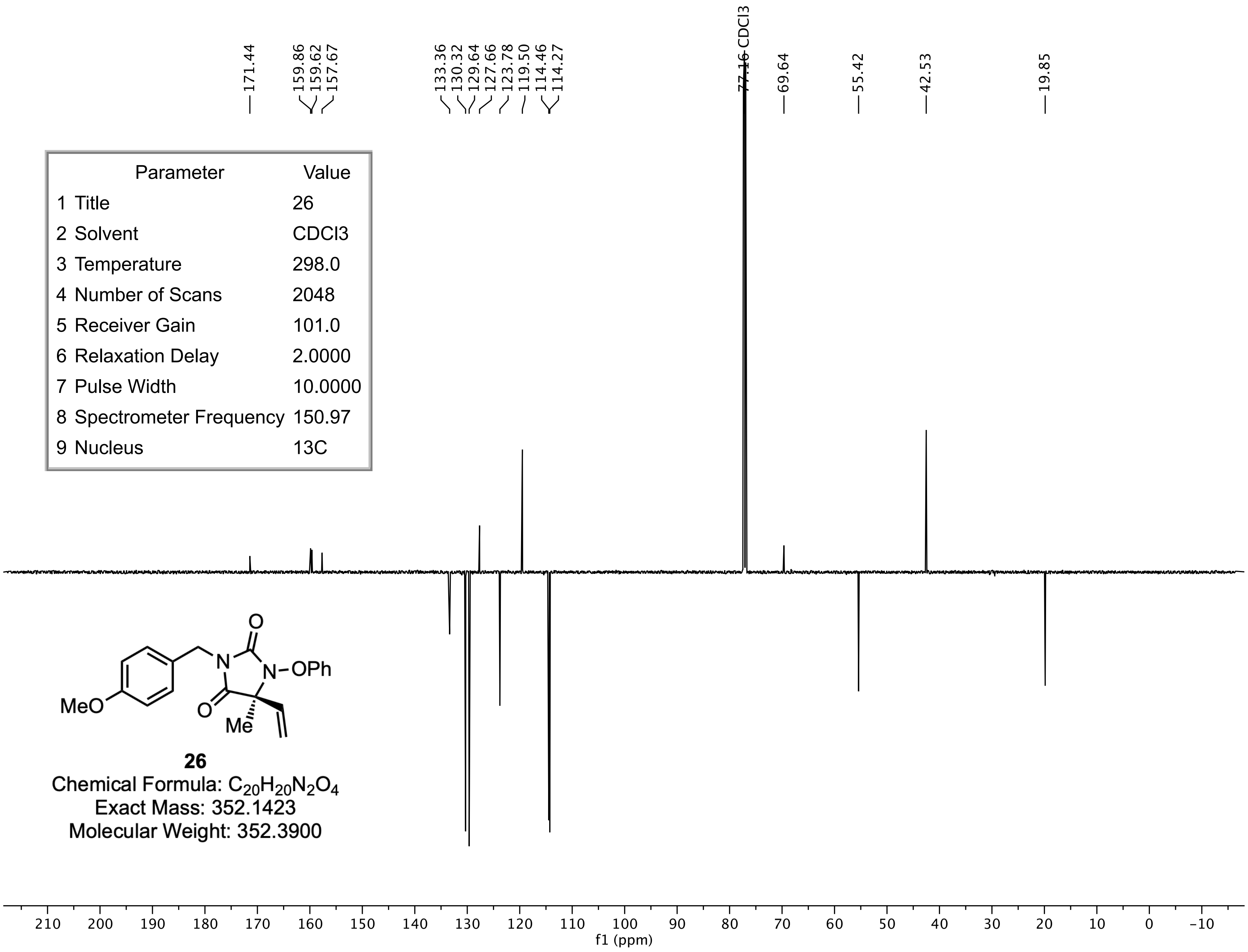


| Parameter | Value |
|---|---|
| 1 Title | 26 |
| 2 Solvent | CDCl3 |
| 3 Temperature | 298.0 |
| 4 Number of Scans | 2048 |
| 5 Receiver Gain | 101.0 |
| 6 Relaxation Delay | 2.0000 |
| 7 Pulse Width | 10.0000 |
| 8 Spectrometer Frequency | 150.97 |
| 9 Nucleus | 13C |

**26**

Chemical Formula: $C_{20}H_{20}N_2O_4$

Exact Mass: 352.1423

Molecular Weight: 352.3900

| | Parameter | Value |
|---|---|---|
| 1 | Title | 27 |
| 2 | Solvent | CDCl3 |
| 3 | Temperature | 298.0 |
| 4 | Number of Scans | 16 |
| 5 | Receiver Gain | 101.0 |
| 6 | Relaxation Delay | 1.0000 |
| 7 | Pulse Width | 15.0000 |
| 8 | Spectrometer Frequency | 400.13 |
| 9 | Nucleus | 1H |

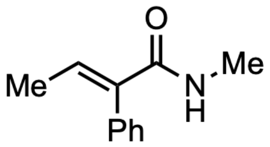

**27**
Chemical Formula: $C_{11}H_{13}NO$
Exact Mass: 175.0997
Molecular Weight: 175.2310

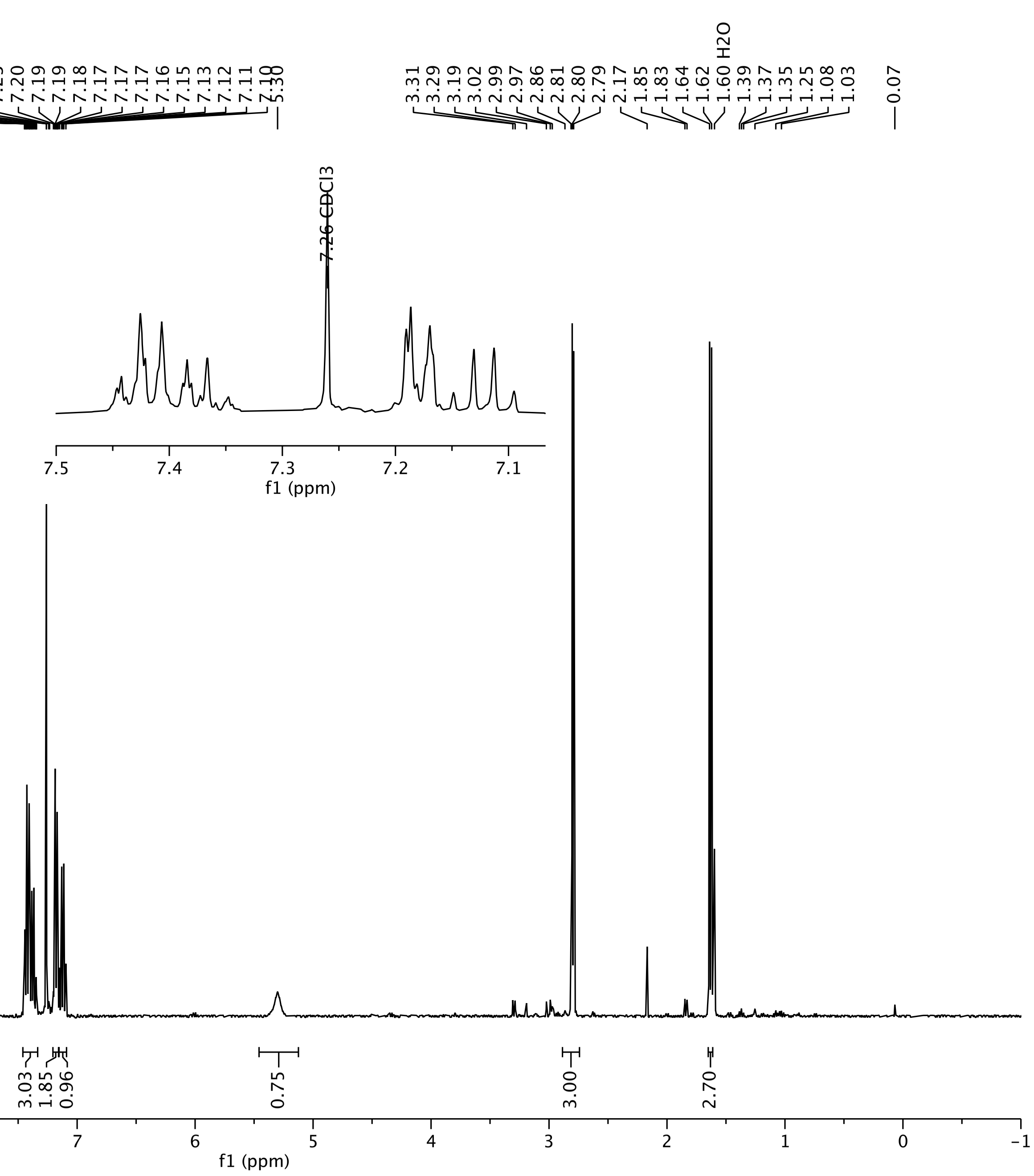

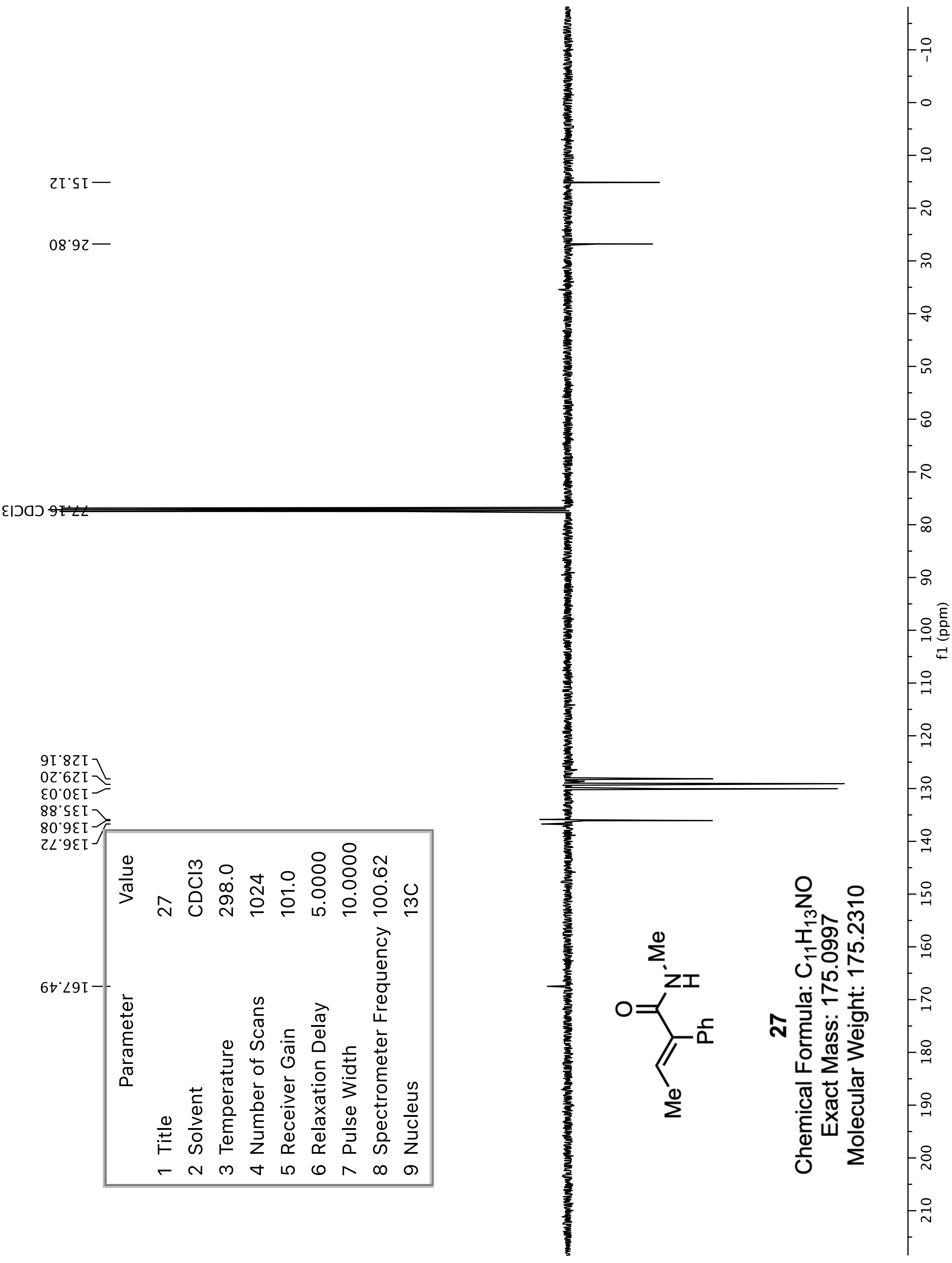


| | Parameter | Value |
|---|---|---|
| 1 | Title | 27 |
| 2 | Solvent | CDCl3 |
| 3 | Temperature | 298.0 |
| 4 | Number of Scans | 1024 |
| 5 | Receiver Gain | 101.0 |
| 6 | Relaxation Delay | 5.0000 |
| 7 | Pulse Width | 10.0000 |
| 8 | Spectrometer Frequency | 100.62 |
| 9 | Nucleus | 13C |

**27**

Chemical Formula: $C_{11}H_{13}NO$

Exact Mass: 175.0997

Molecular Weight: 175.2310

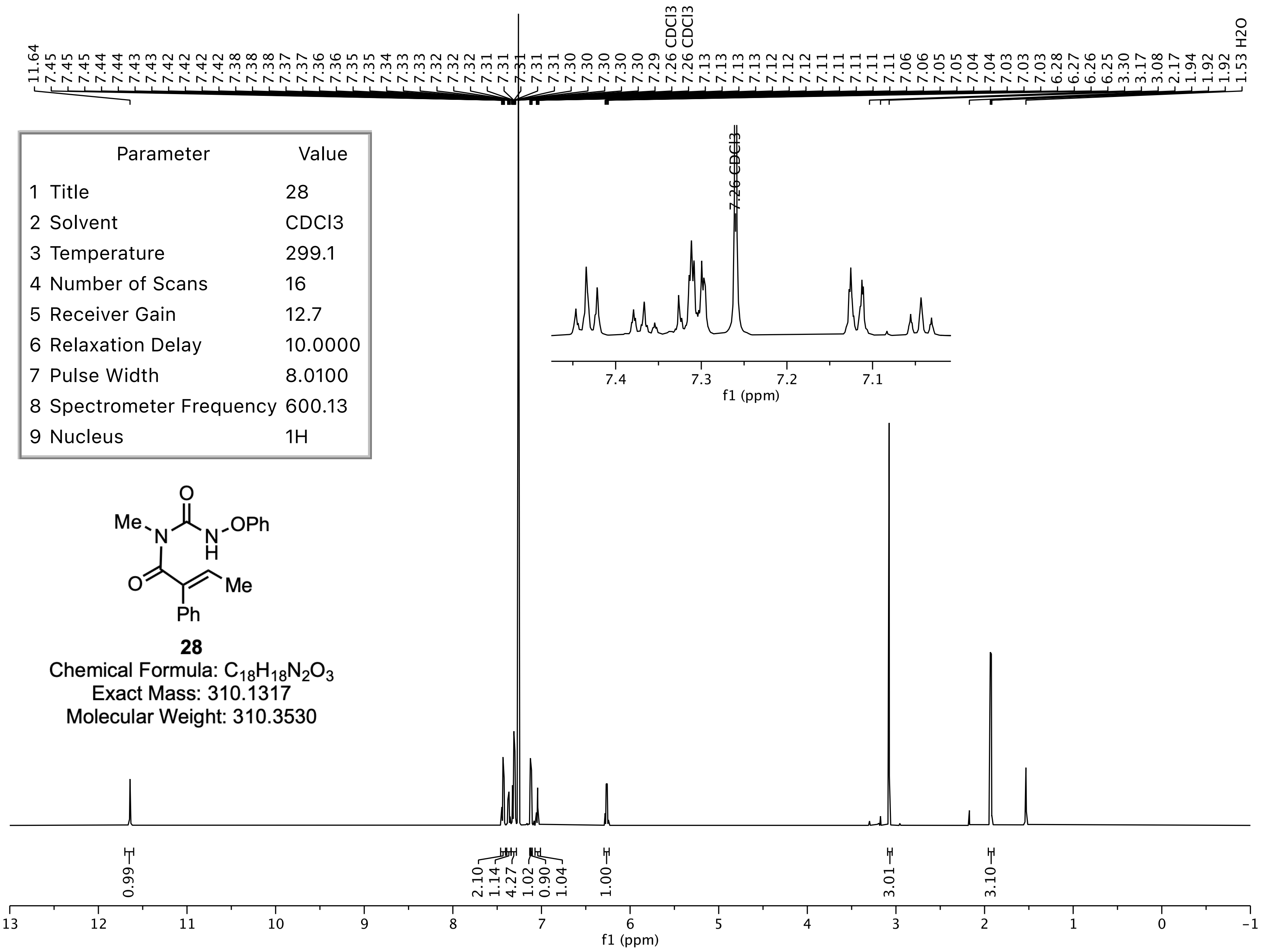


| | Parameter | Value |
|---|---|---|
| 1 | Title | 28 |
| 2 | Solvent | CDCl3 |
| 3 | Temperature | 299.1 |
| 4 | Number of Scans | 16 |
| 5 | Receiver Gain | 12.7 |
| 6 | Relaxation Delay | 10.0000 |
| 7 | Pulse Width | 8.0100 |
| 8 | Spectrometer Frequency | 600.13 |
| 9 | Nucleus | 1H |

**28**

Chemical Formula: $C_{18}H_{18}N_2O_3$
Exact Mass: 310.1317
Molecular Weight: 310.3530

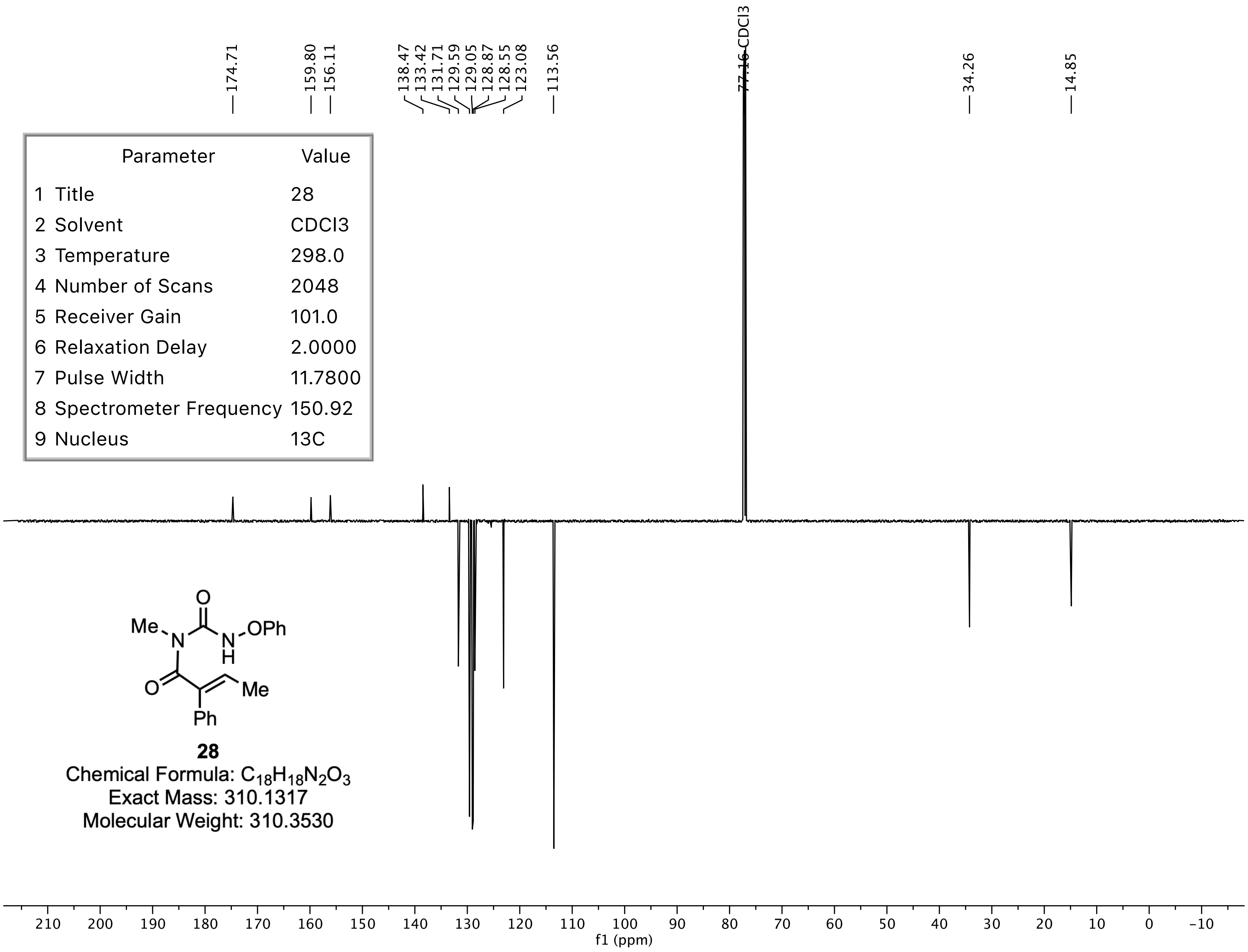


| Parameter | Value |
| --- | --- |
| 1 Title | 28 |
| 2 Solvent | CDCl3 |
| 3 Temperature | 298.0 |
| 4 Number of Scans | 2048 |
| 5 Receiver Gain | 101.0 |
| 6 Relaxation Delay | 2.0000 |
| 7 Pulse Width | 11.7800 |
| 8 Spectrometer Frequency | 150.92 |
| 9 Nucleus | 13C |



**28**

Chemical Formula: $C_{18}H_{18}N_2O_3$

Exact Mass: 310.1317

Molecular Weight: 310.3530

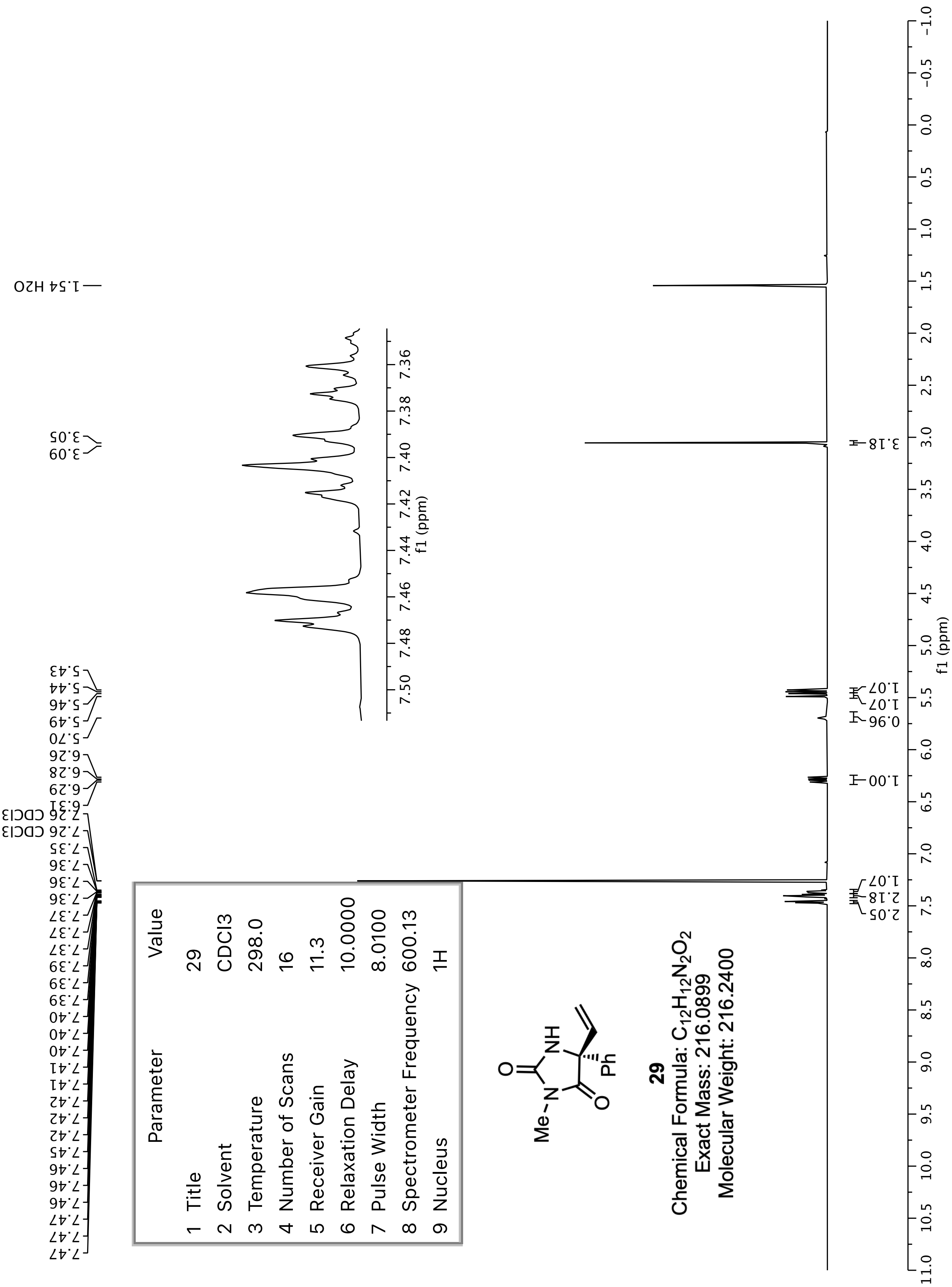


| | Parameter | Value |
|---|---|---|
| 1 | Title | 29 |
| 2 | Solvent | CDCl3 |
| 3 | Temperature | 298.0 |
| 4 | Number of Scans | 16 |
| 5 | Receiver Gain | 11.3 |
| 6 | Relaxation Delay | 10.0000 |
| 7 | Pulse Width | 8.0100 |
| 8 | Spectrometer Frequency | 600.13 |
| 9 | Nucleus | 1H |

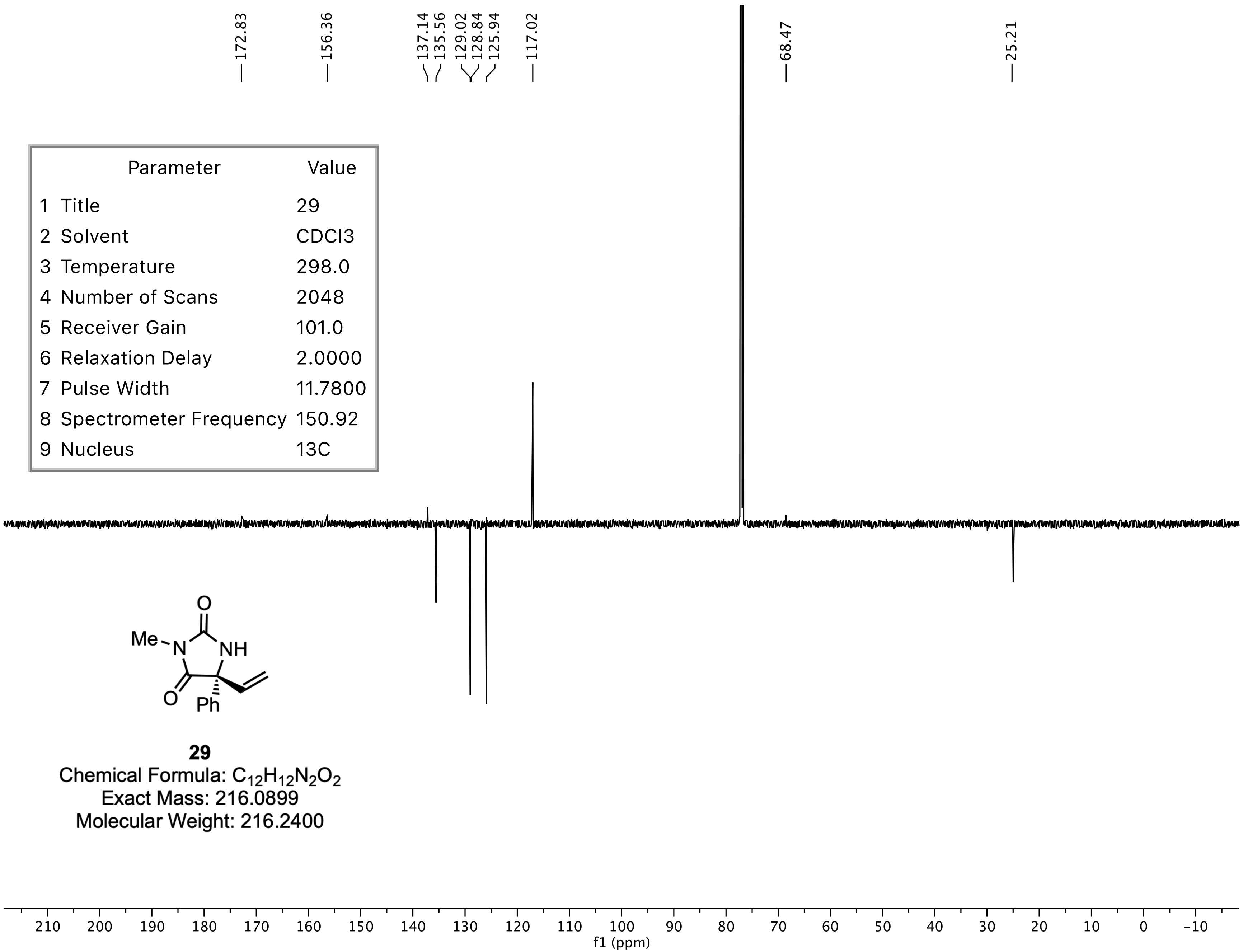


| | Parameter | Value |
|---|---|---|
| 1 | Title | 29 |
| 2 | Solvent | CDCl3 |
| 3 | Temperature | 298.0 |
| 4 | Number of Scans | 2048 |
| 5 | Receiver Gain | 101.0 |
| 6 | Relaxation Delay | 2.0000 |
| 7 | Pulse Width | 11.7800 |
| 8 | Spectrometer Frequency | 150.92 |
| 9 | Nucleus | 13C |

**29**
Chemical Formula: $C_{12}H_{12}N_2O_2$
Exact Mass: 216.0899
Molecular Weight: 216.2400

| | Parameter | Value |
|---|---|---|
| 1 | Title | 30 |
| 2 | Solvent | CDCl3 |
| 3 | Temperature | 298.0 |
| 4 | Number of Scans | 16 |
| 5 | Receiver Gain | 11.3 |
| 6 | Relaxation Delay | 10.0000 |
| 7 | Pulse Width | 8.0100 |
| 8 | Spectrometer Frequency | 600.13 |
| 9 | Nucleus | 1H |

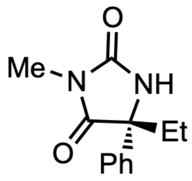


**30**
Chemical Formula: $C_{12}H_{14}N_2O_2$
Exact Mass: 218.1055
Molecular Weight: 218.2560

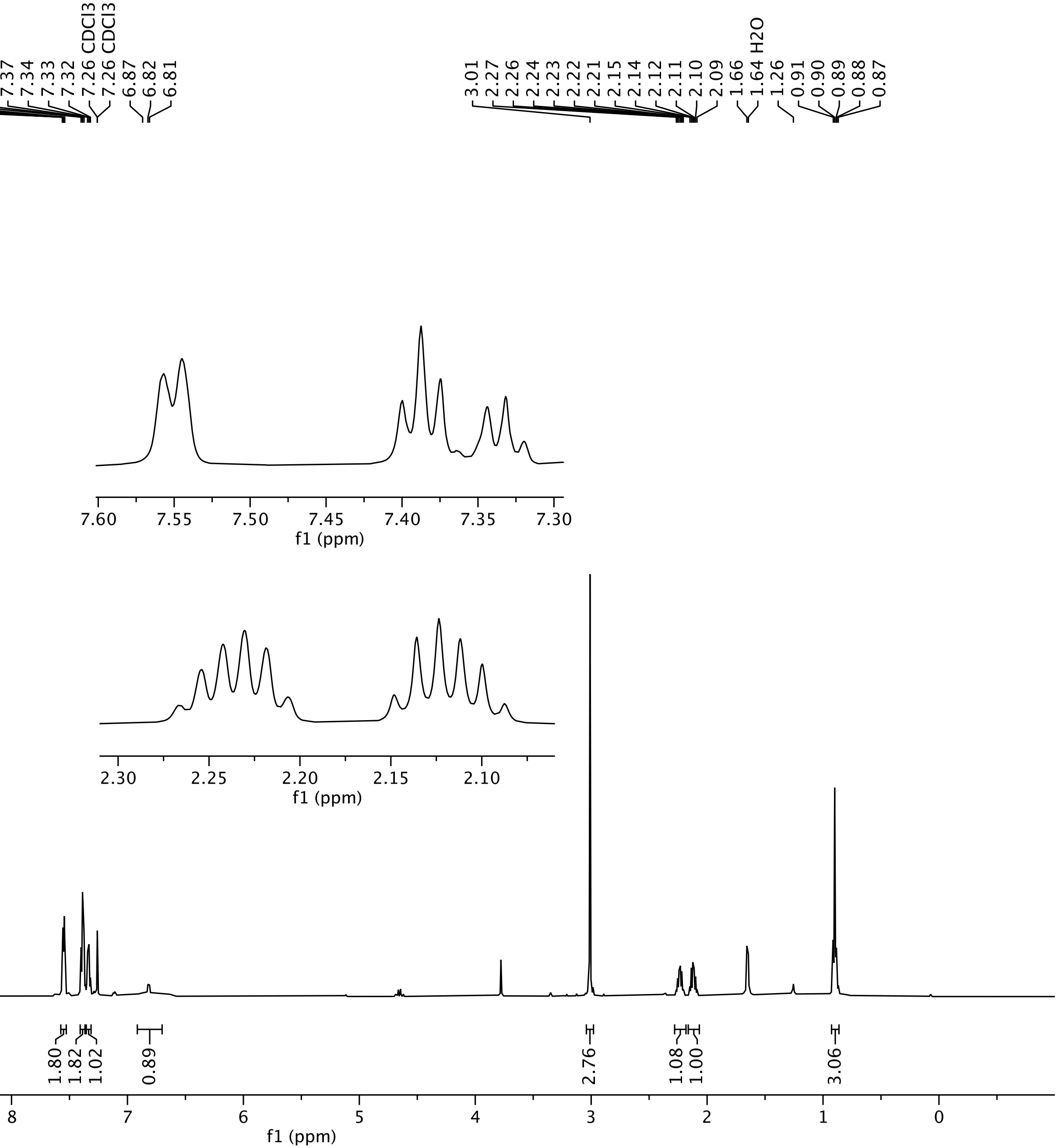

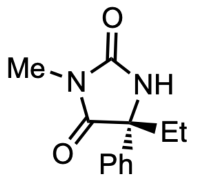

**30**

Chemical Formula: $C_{12}H_{14}N_2O_2$
Exact Mass: 218.1055
Molecular Weight: 218.2560

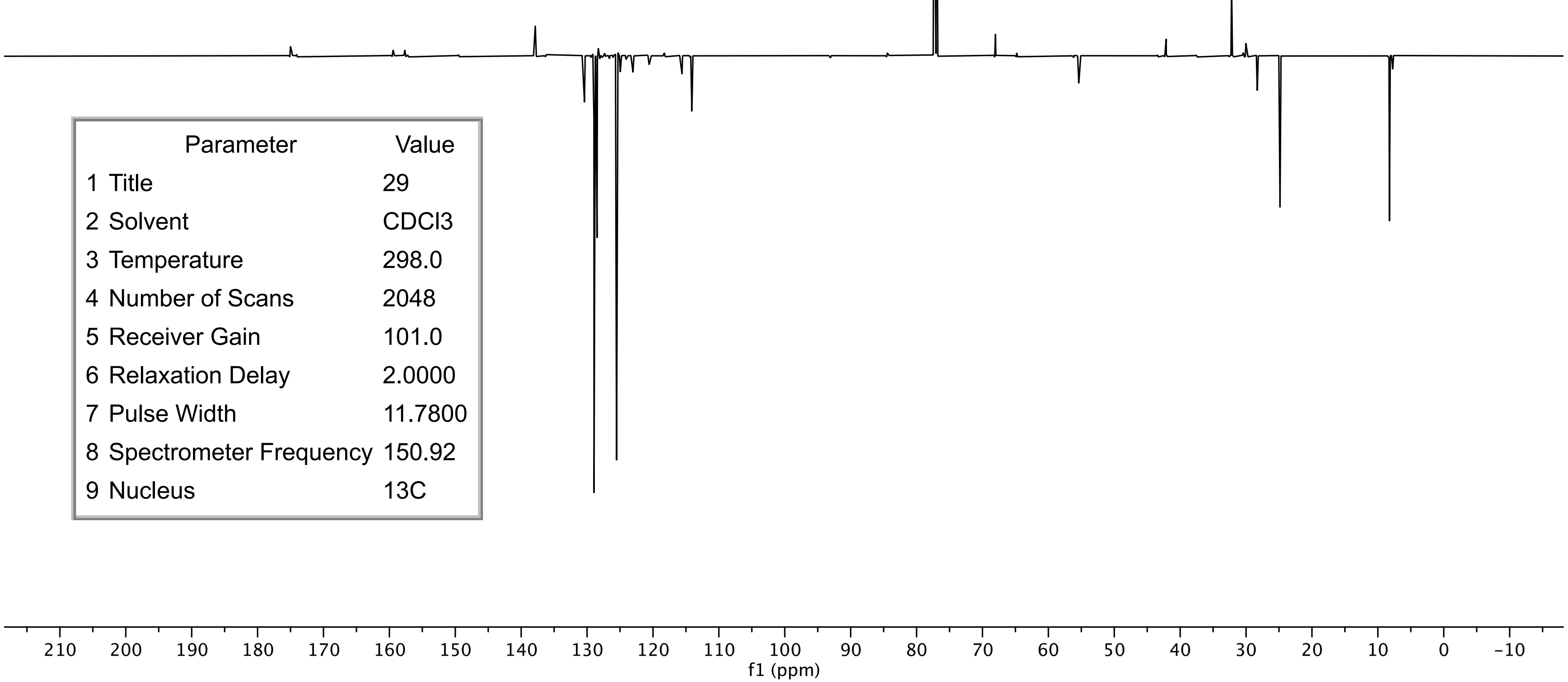


| | Parameter | Value |
|---|---|---|
| 1 | Title | 29 |
| 2 | Solvent | CDCl3 |
| 3 | Temperature | 298.0 |
| 4 | Number of Scans | 2048 |
| 5 | Receiver Gain | 101.0 |
| 6 | Relaxation Delay | 2.0000 |
| 7 | Pulse Width | 11.7800 |
| 8 | Spectrometer Frequency | 150.92 |
| 9 | Nucleus | 13C |

| Parameter | Value |
|---|---|
| 1 Title | S59, crude mixture, for identification purposes only |
| 2 Solvent | CDCl3 |
| 3 Temperature | 300.0 |
| 4 Number of Scans | 16 |
| 5 Receiver Gain | 32.0 |
| 6 Relaxation Delay | 1.0000 |
| 7 Pulse Width | 11.1000 |
| 8 Spectrometer Frequency | 600.13 |
| 9 Nucleus | 1H |

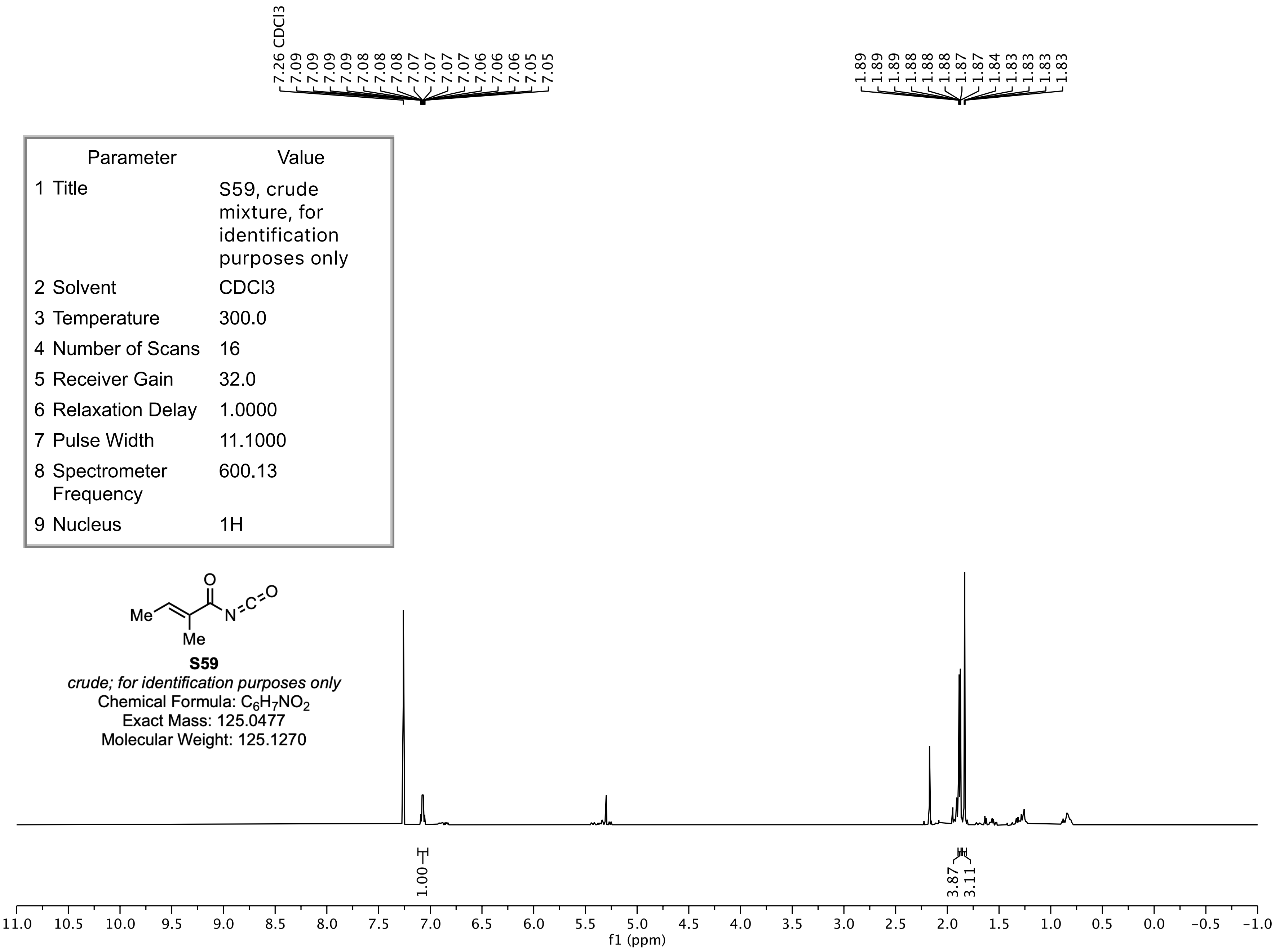


**S59**

*crude; for identification purposes only*

Chemical Formula: $C_6H_7NO_2$

Exact Mass: 125.0477

Molecular Weight: 125.1270

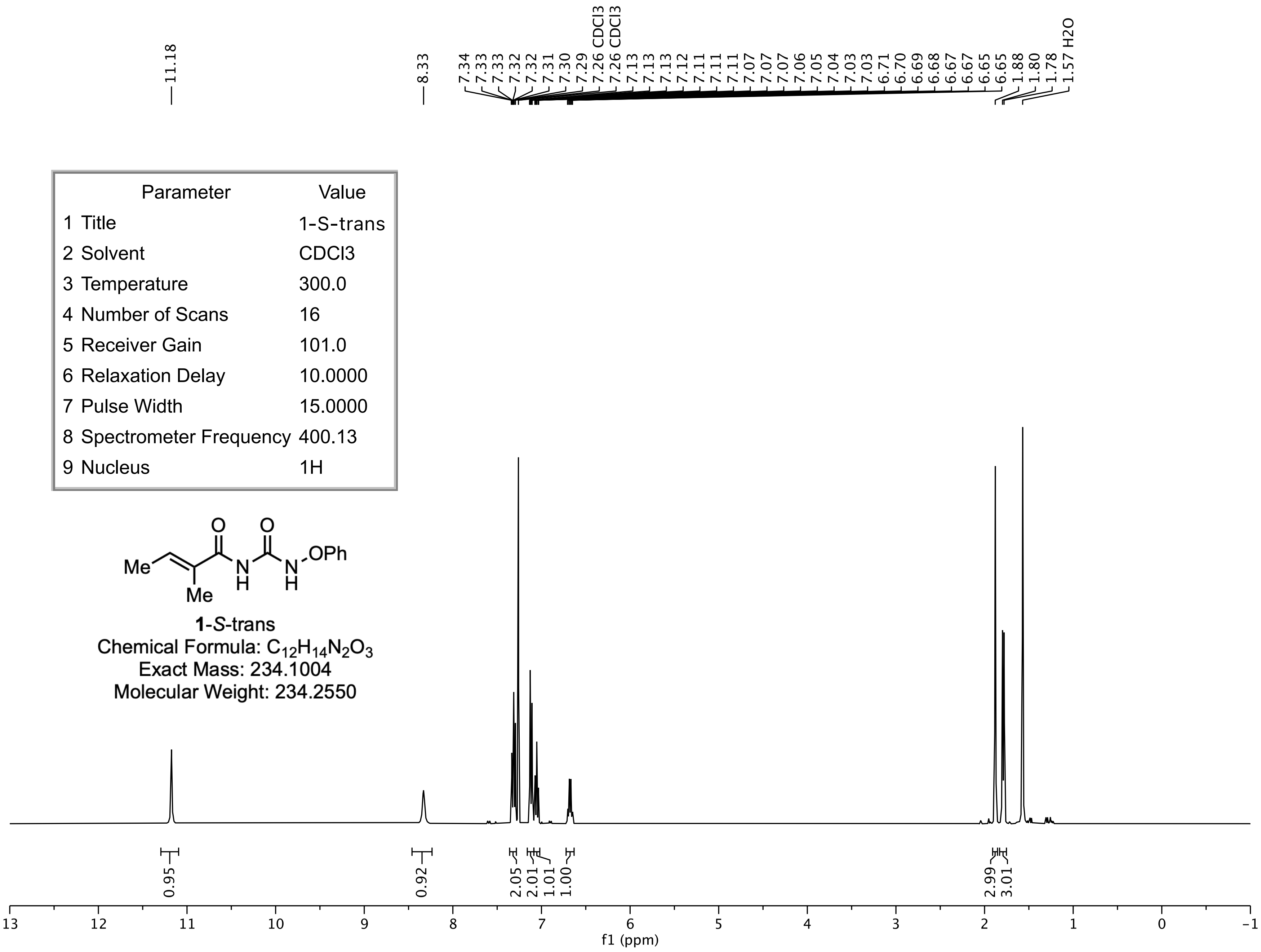


| | Parameter | Value |
|---|---|---|
| 1 | Title | 1-S-trans |
| 2 | Solvent | CDCl3 |
| 3 | Temperature | 300.0 |
| 4 | Number of Scans | 16 |
| 5 | Receiver Gain | 101.0 |
| 6 | Relaxation Delay | 10.0000 |
| 7 | Pulse Width | 15.0000 |
| 8 | Spectrometer Frequency | 400.13 |
| 9 | Nucleus | 1H |

**1**-*S*-trans
Chemical Formula: $C_{12}H_{14}N_2O_3$
Exact Mass: 234.1004
Molecular Weight: 234.2550

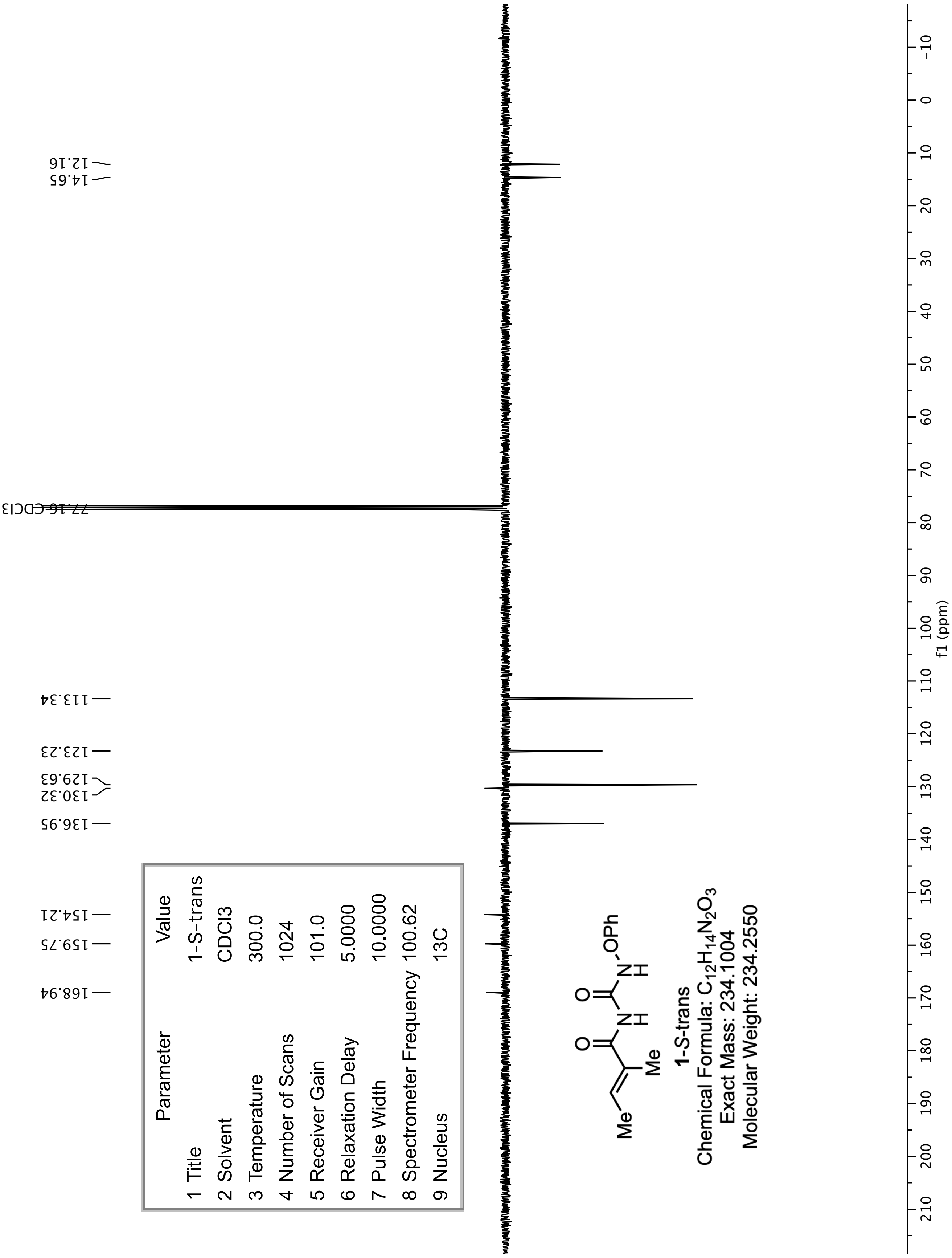
12.16
14.65
77.16 CDCl3
113.34
123.23
129.63
130.32
136.95
154.21
159.75
168.94
Parameter Value
1 Title 1-S-trans
2 Solvent CDCl3
3 Temperature 300.0
4 Number of Scans 1024
5 Receiver Gain 101.0
6 Relaxation Delay 5.0000
7 Pulse Width 10.0000
8 Spectrometer Frequency 100.62
9 Nucleus 13C
Me
Me
O
O
N
H
N
H
OPh
1-S-trans
Chemical Formula: $C_{12}H_{14}N_2O_3$
Exact Mass: 234.1004
Molecular Weight: 234.2550
f1 (ppm)
210 200 190 180 170 160 150 140 130 120 110 100 90 80 70 60 50 40 30 20 10 0 -10

| Parameter | Value |
|---|---|
| 1 Title | S60 |
| 2 Solvent | CDCl3 |
| 3 Temperature | 300.0 |
| 4 Number of Scans | 16 |
| 5 Receiver Gain | 90.5 |
| 6 Relaxation Delay | 1.0000 |
| 7 Pulse Width | 11.1500 |
| 8 Spectrometer Frequency | 600.32 |
| 9 Nucleus | 1H |

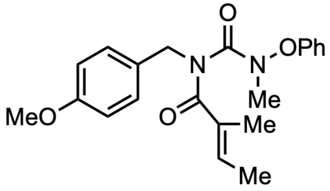


**S60**
Chemical Formula: $C_{21}H_{24}N_2O_4$
Exact Mass: 368.1736
Molecular Weight: 368.4330

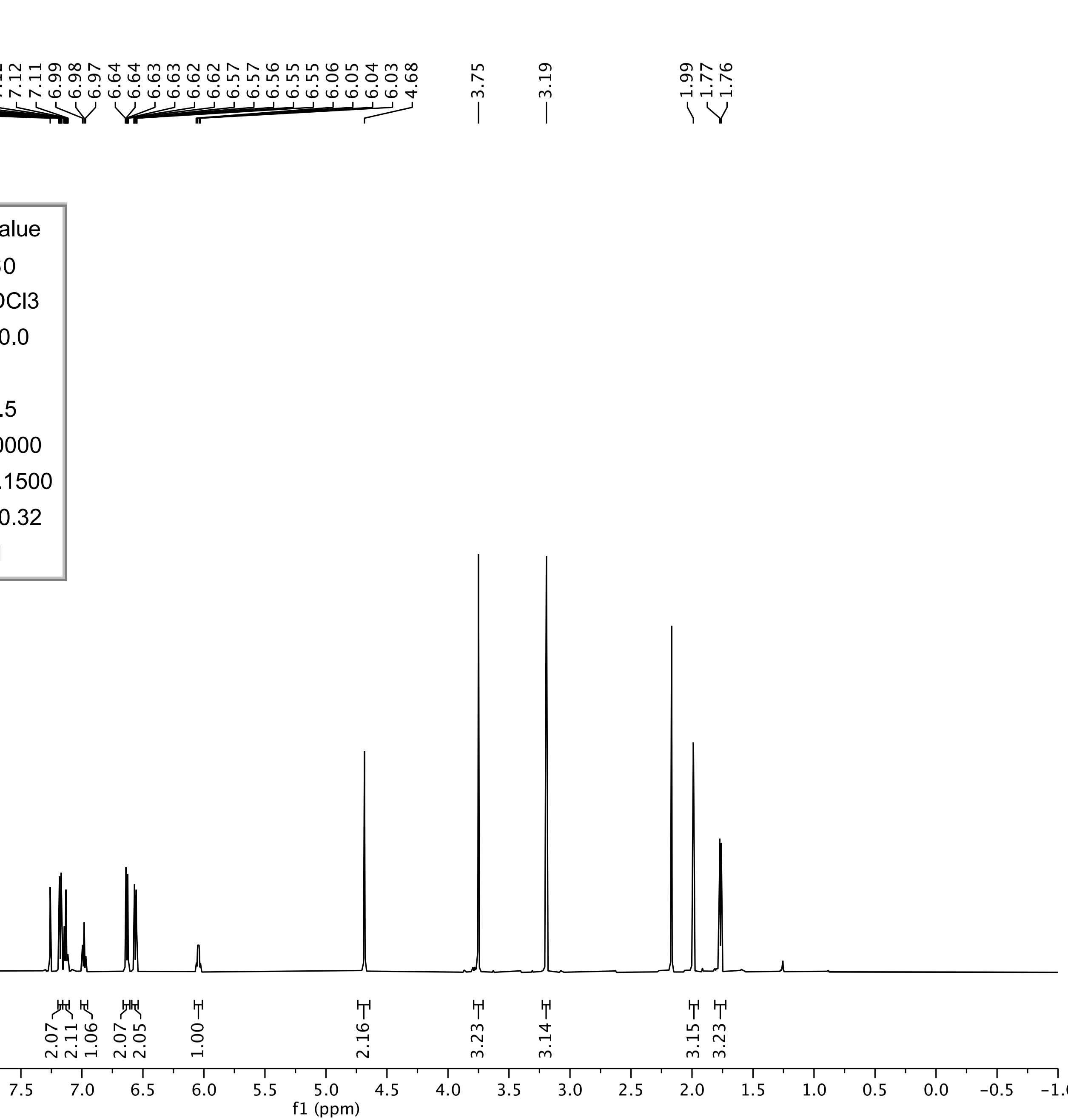

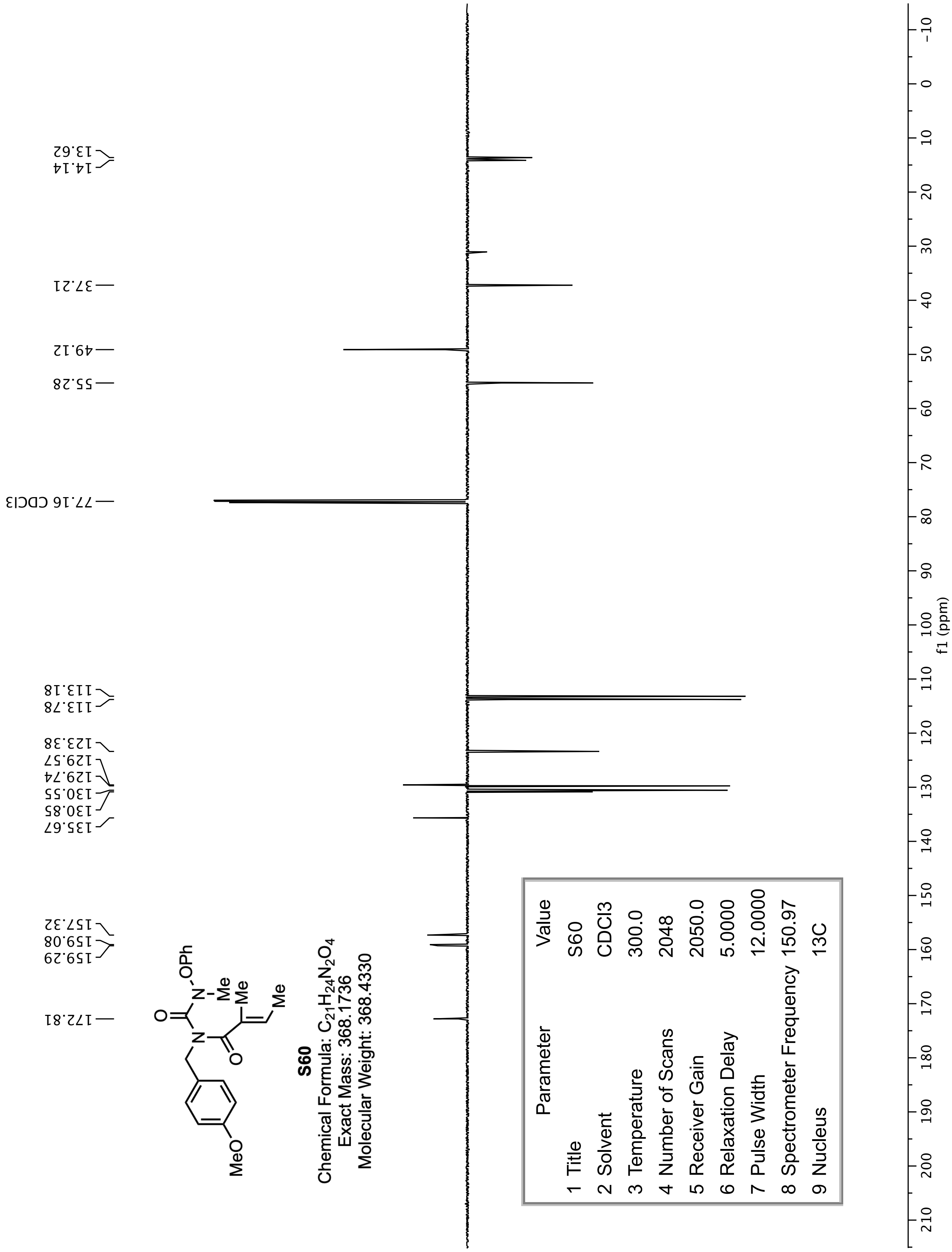
13.62
14.14
37.21
49.12
55.28
77.16 CDCl3
113.18
113.78
123.38
129.57
129.74
130.55
130.85
135.67
157.32
159.08
159.29
172.81
MeO
OPh
Me
Me
Me
S60
Chemical Formula: $C_{21}H_{24}N_2O_4$
Exact Mass: 368.1736
Molecular Weight: 368.4330
Parameter
Value
1 Title S60
2 Solvent CDCl3
3 Temperature 300.0
4 Number of Scans 2048
5 Receiver Gain 2050.0
6 Relaxation Delay 5.0000
7 Pulse Width 12.0000
8 Spectrometer Frequency 150.97
9 Nucleus 13C
f1 (ppm)

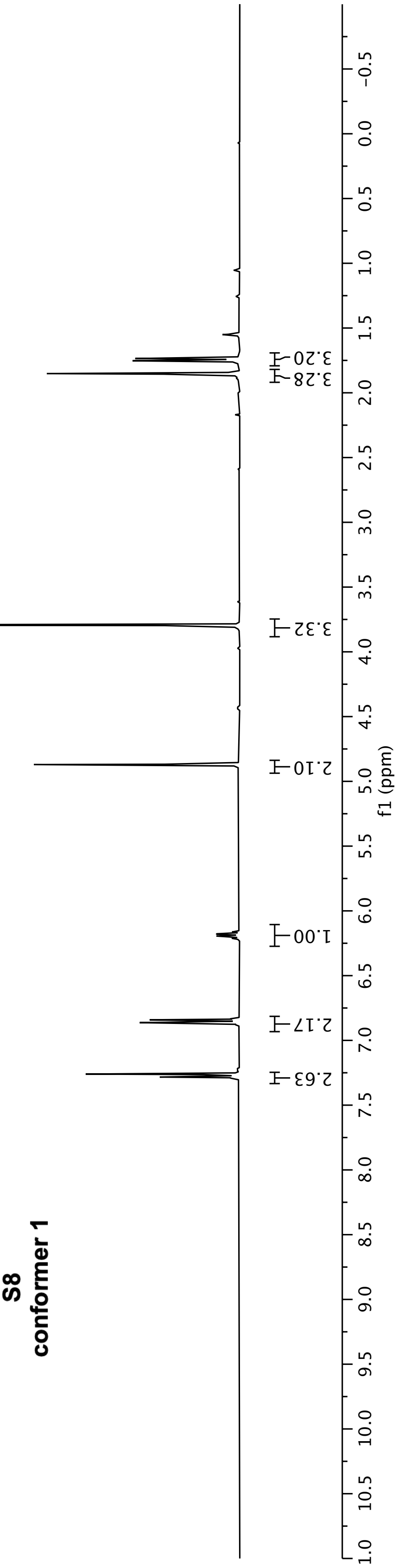


| Parameter | Value |
|---|---|
| 1 Title | S8 conformer 1 |
| 2 Solvent | CDCl3 |
| 3 Temperature | 298.0 |
| 4 Number of Scans | 32 |
| 5 Receiver Gain | 101.0 |
| 6 Relaxation Delay | 10.0000 |
| 7 Pulse Width | 15.0000 |
| 8 Spectrometer Frequency | 400.13 |
| 9 Nucleus | 1H |

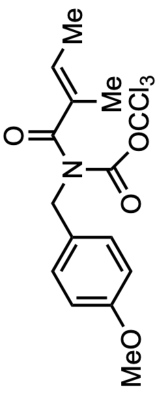


**S8**
**conformer 1**

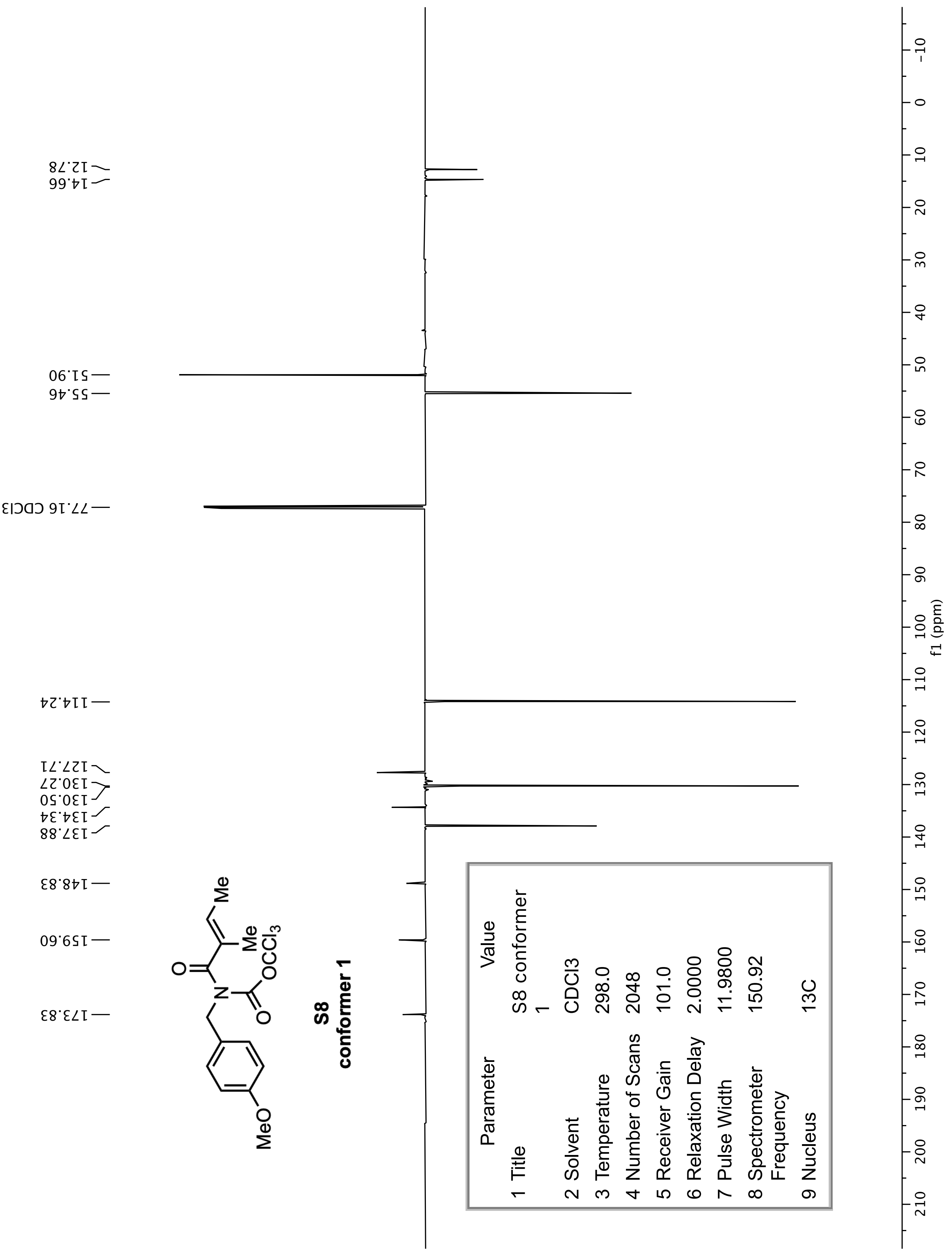


| Parameter | Value |
|---|---|
| 1 Title | S8 conformer 1 |
| 2 Solvent | CDCl3 |
| 3 Temperature | 298.0 |
| 4 Number of Scans | 2048 |
| 5 Receiver Gain | 101.0 |
| 6 Relaxation Delay | 2.0000 |
| 7 Pulse Width | 11.9800 |
| 8 Spectrometer Frequency | 150.92 |
| 9 Nucleus | 13C |

| | Parameter | Value |
|---|---|---|
| 1 | Title | S8 Conformer 1 |
| 2 | Solvent | CDCl3 |
| 3 | Temperature | 298.0 |
| 4 | Number of Scans | 4 |
| 5 | Receiver Gain | 101.0 |
| 6 | Relaxation Delay | 1.9140 |
| 7 | Pulse Width | 8.0700 |
| 8 | Spectrometer Frequency | (600.13, 600.13) |
| 9 | Spectral Width | (4717.0, 4717.0) |
| 10 | Lowest Frequency | (141.2, 152.6) |
| 11 | Nucleus | (1H, 1H) |
| 12 | Acquired Size | (1024, 256) |
| 13 | Spectral Size | (1024, 1024) |
| 14 | Digital Resolution | (4.61, 4.61) |

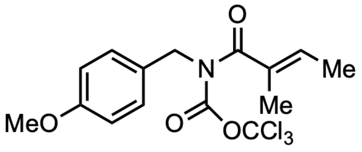


**S8**
**conformer 1**

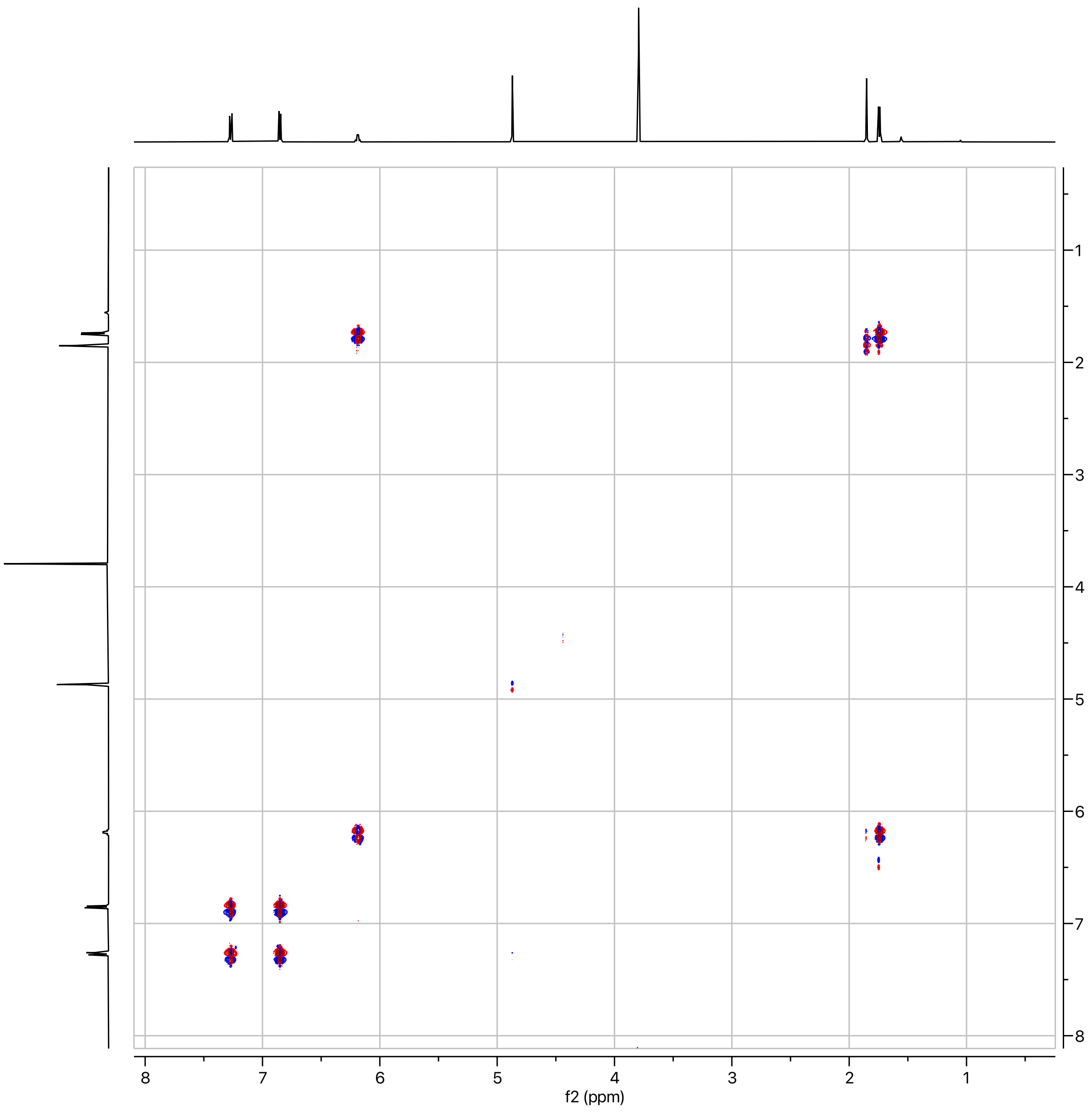

| | Parameter | Value |
|---|---|---|
| 1 | Title | S8 conformer 1 |
| 2 | Solvent | CDCl3 |
| 3 | Temperature | 298.0 |
| 4 | Number of Scans | 2 |
| 5 | Receiver Gain | 101.0 |
| 6 | Relaxation Delay | 1.5000 |
| 7 | Pulse Width | 8.0700 |
| 8 | Spectrometer Frequency | (600.13, 150.91) |
| 9 | Spectral Width | (4717.0, 24900.8) |
| 10 | Lowest Frequency | (141.0, -1076.5) |
| 11 | Nucleus | (1H, 13C) |
| 12 | Acquired Size | (512, 256) |
| 13 | Spectral Size | (512, 512) |
| 14 | Digital Resolution | (9.21, 48.63) |

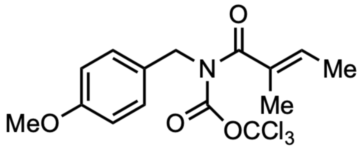


**S8**
**conformer 1**

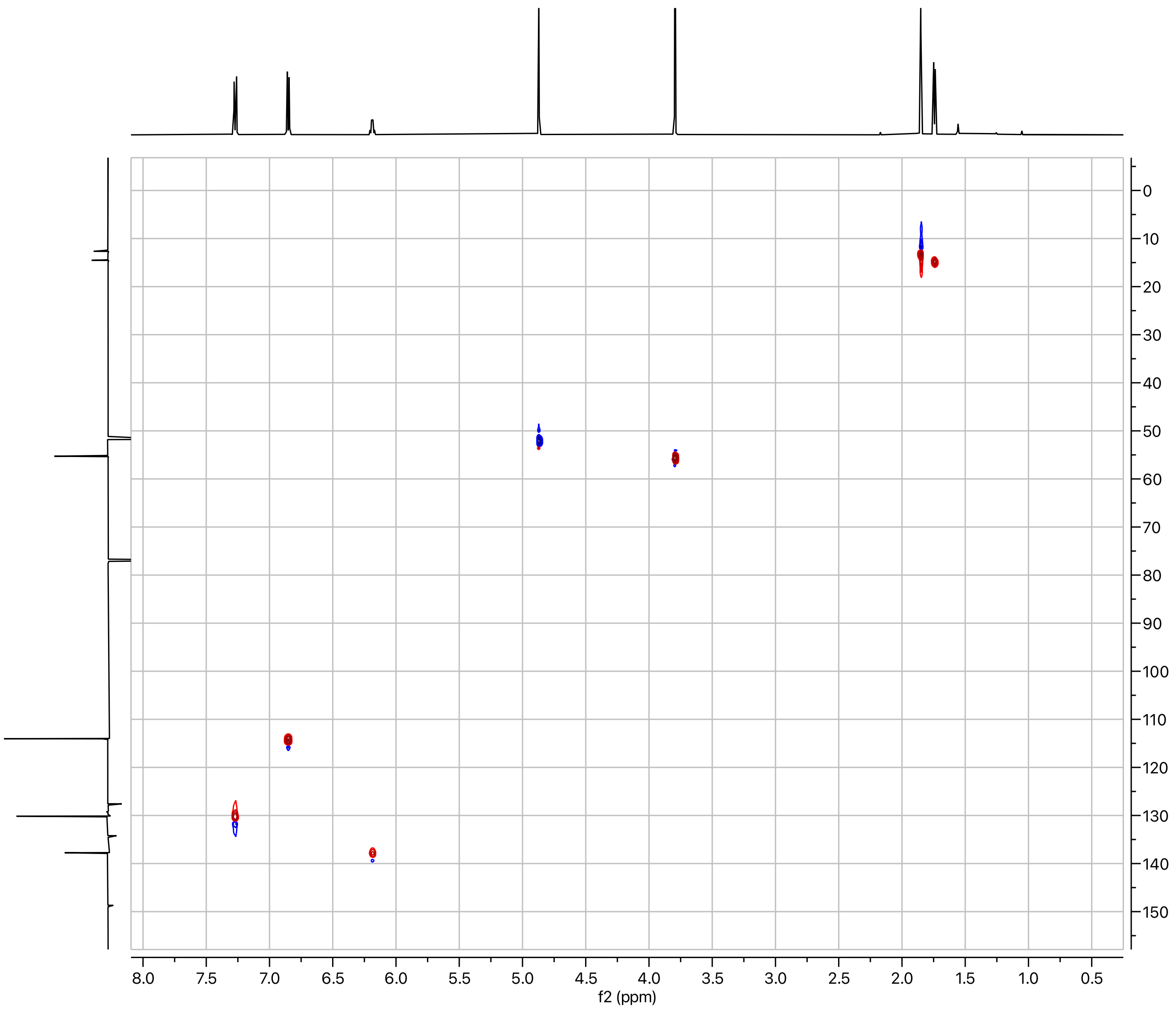

| | Parameter | Value |
|---|---|---|
| 1 | Title | S8 conformer 1 |
| 2 | Solvent | CDCl3 |
| 3 | Temperature | 298.0 |
| 4 | Number of Scans | 32 |
| 5 | Receiver Gain | 101.0 |
| 6 | Relaxation Delay | 1.5000 |
| 7 | Pulse Width | 8.0700 |
| 8 | Spectrometer Frequency | (600.13, 150.92) |
| 9 | Spectral Width | (4717.0, 33201.9) |
| 10 | Lowest Frequency | (142.2, -1478.1) |
| 11 | Nucleus | (1H, 13C) |
| 12 | Acquired Size | (1024, 128) |
| 13 | Spectral Size | (1024, 512) |
| 14 | Digital Resolution | (4.61, 64.85) |

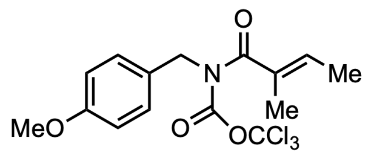


**S8**
**conformer 1**

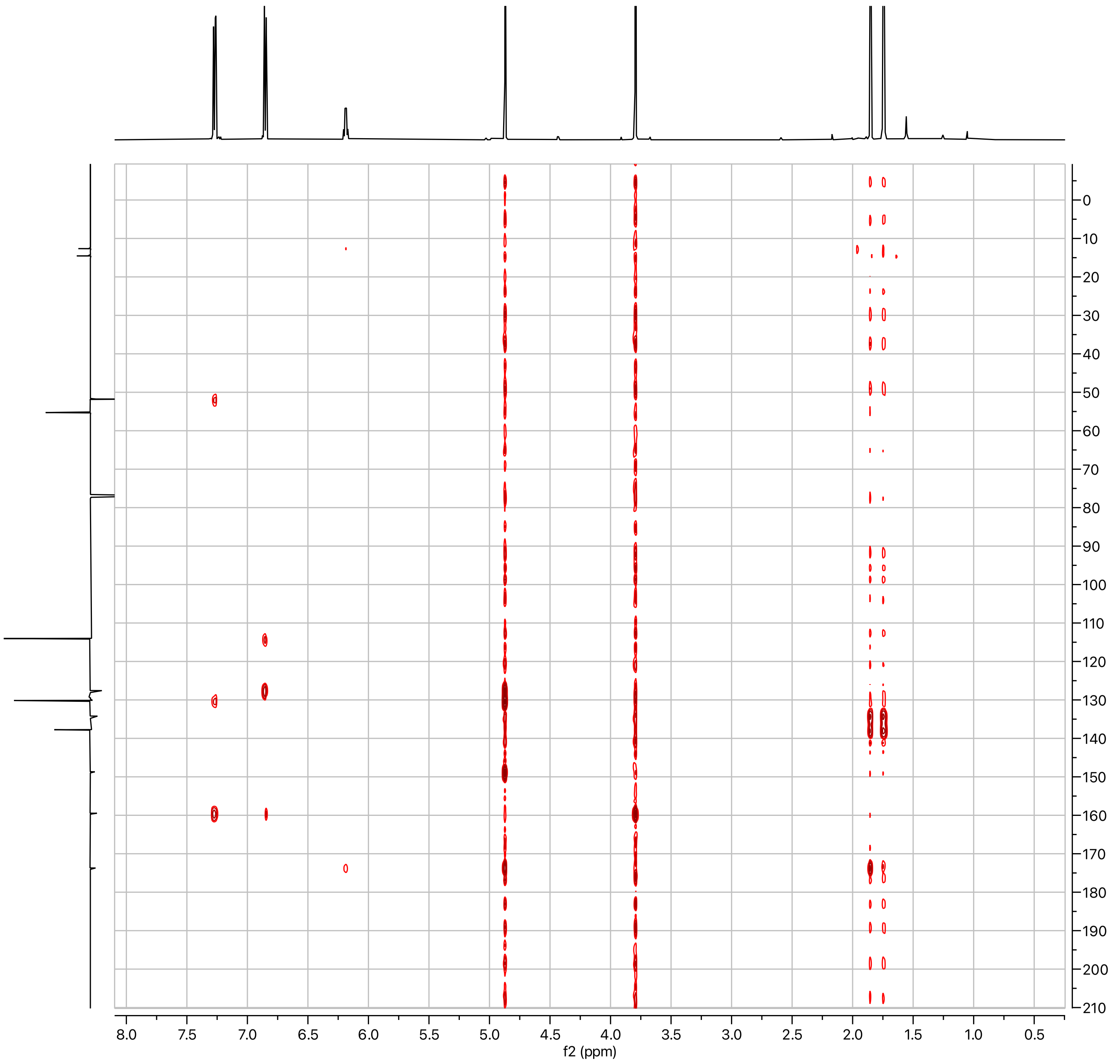

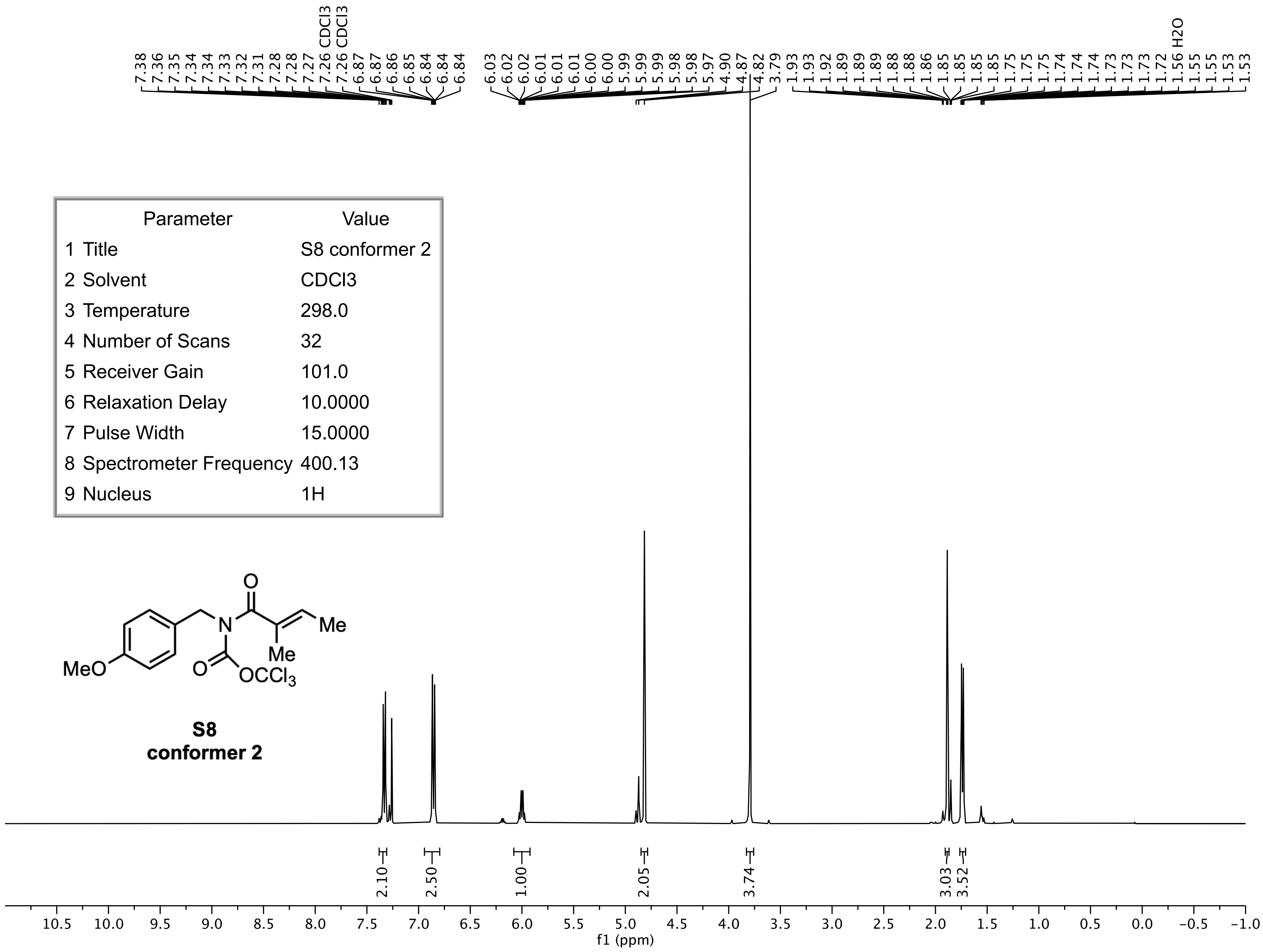


| Parameter | Value |
|---|---|
| 1 Title | S8 conformer 2 |
| 2 Solvent | CDCl3 |
| 3 Temperature | 298.0 |
| 4 Number of Scans | 32 |
| 5 Receiver Gain | 101.0 |
| 6 Relaxation Delay | 10.0000 |
| 7 Pulse Width | 15.0000 |
| 8 Spectrometer Frequency | 400.13 |
| 9 Nucleus | 1H |

**S8**
**conformer 2**

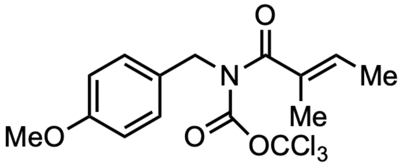


**S8**
**conformer 2**

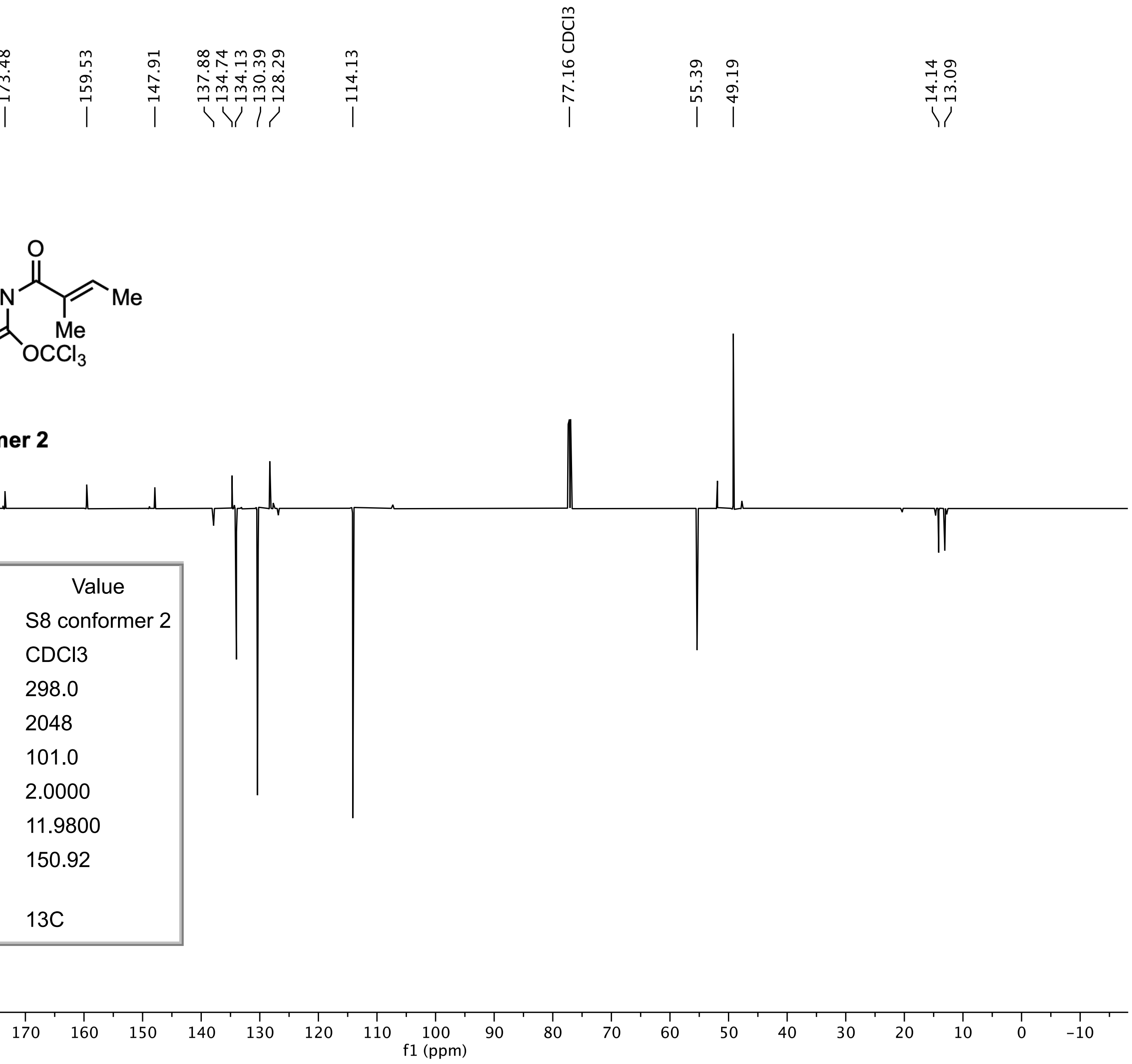


| Parameter | Value |
|---|---|
| 1 Title | S8 conformer 2 |
| 2 Solvent | CDCl3 |
| 3 Temperature | 298.0 |
| 4 Number of Scans | 2048 |
| 5 Receiver Gain | 101.0 |
| 6 Relaxation Delay | 2.0000 |
| 7 Pulse Width | 11.9800 |
| 8 Spectrometer Frequency | 150.92 |
| 9 Nucleus | 13C |

| | Parameter | Value |
|---|---|---|
| 1 | Title | S8 conformer 2 |
| 2 | Solvent | CDCl3 |
| 3 | Temperature | 298.0 |
| 4 | Number of Scans | 4 |
| 5 | Receiver Gain | 101.0 |
| 6 | Relaxation Delay | 1.9119 |
| 7 | Pulse Width | 8.0700 |
| 8 | Spectrometer Frequency | (600.13, 600.13) |
| 9 | Spectral Width | (4717.0, 4717.0) |
| 10 | Lowest Frequency | (157.9, 171.8) |
| 11 | Nucleus | (1H, 1H) |
| 12 | Acquired Size | (1024, 256) |
| 13 | Spectral Size | (1024, 1024) |
| 14 | Digital Resolution | (4.61, 4.61) |

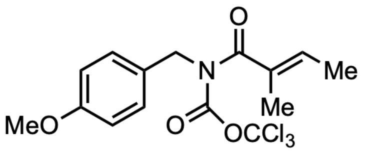


**S8**
**conformer 2**

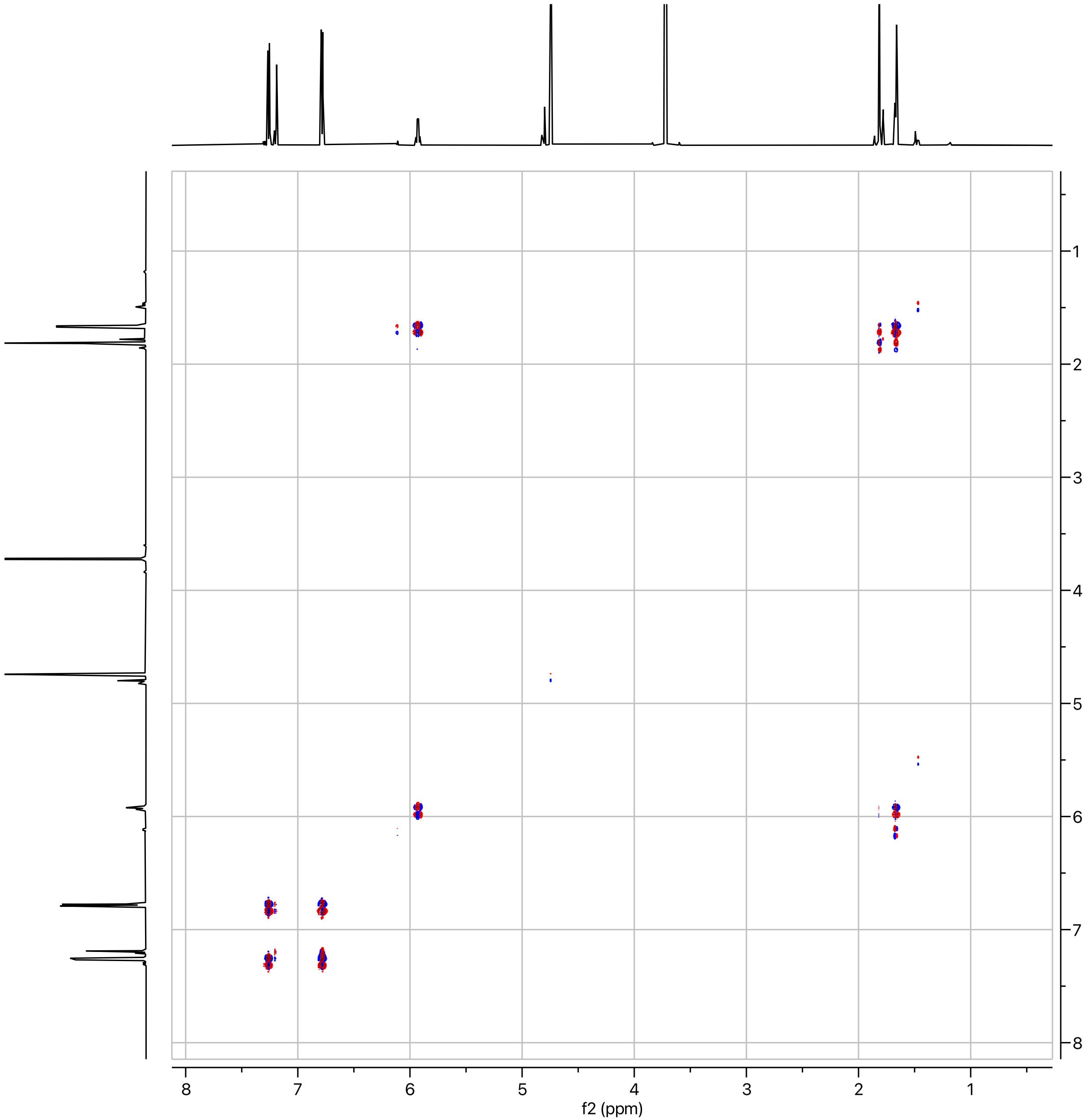

| | Parameter | Value |
|---|---|---|
| 1 | Title | S8 conformer 2 |
| 2 | Solvent | CDCl3 |
| 3 | Temperature | 298.0 |
| 4 | Number of Scans | 2 |
| 5 | Receiver Gain | 101.0 |
| 6 | Relaxation Delay | 1.5000 |
| 7 | Pulse Width | 8.0700 |
| 8 | Spectrometer Frequency | (600.13, 150.91) |
| 9 | Spectral Width | (4717.0, 24900.8) |
| 10 | Lowest Frequency | (157.9, -1132.7) |
| 11 | Nucleus | (1H, 13C) |
| 12 | Acquired Size | (512, 256) |
| 13 | Spectral Size | (512, 512) |
| 14 | Digital Resolution | (9.21, 48.63) |

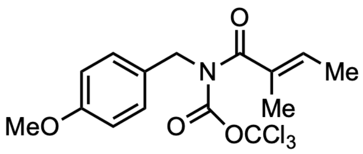


**S8**
**conformer 2**

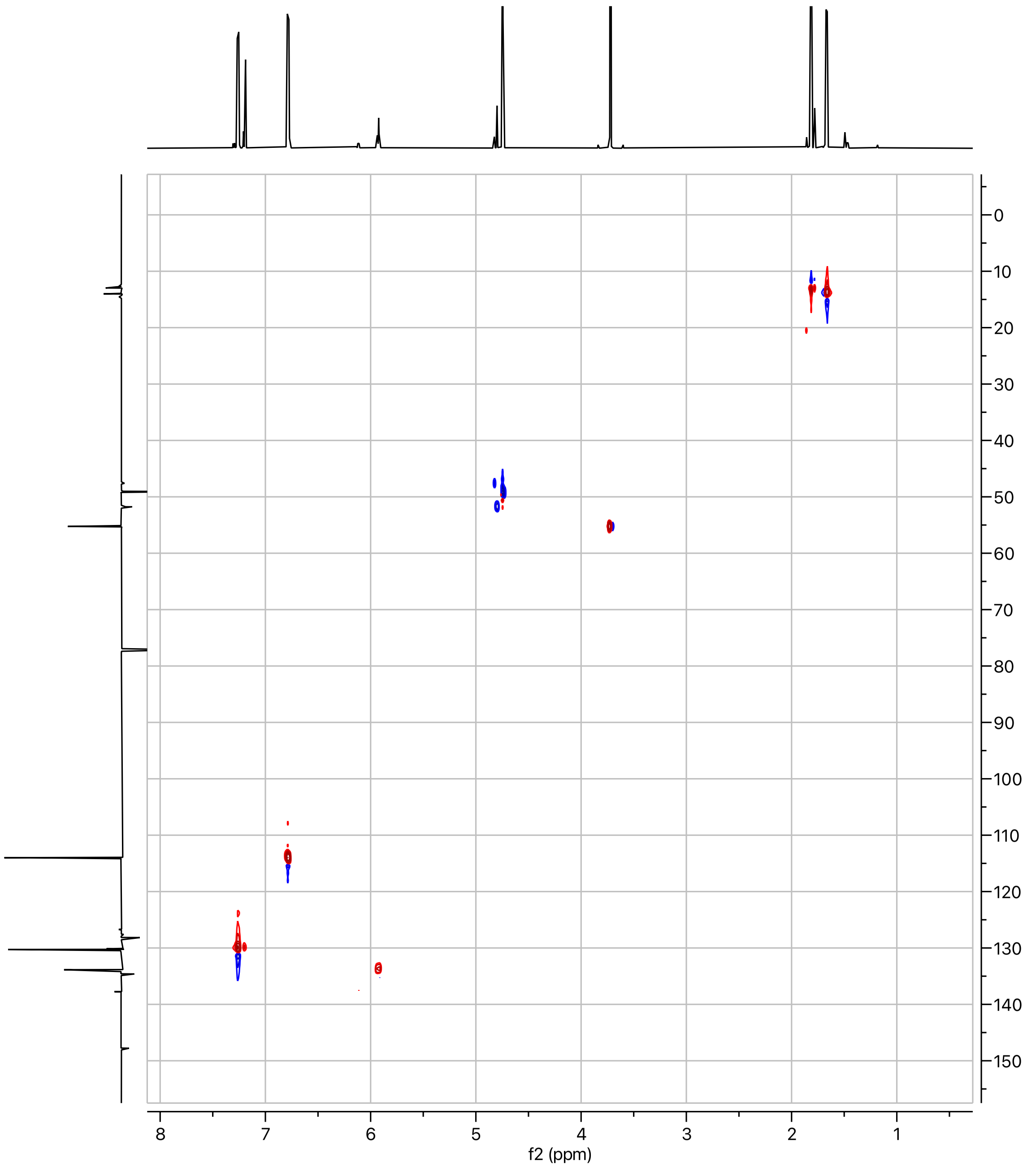

| | Parameter | Value |
|---|---|---|
| 1 | Title | S8 conformer 2 |
| 2 | Solvent | CDCl3 |
| 3 | Temperature | 298.0 |
| 4 | Number of Scans | 32 |
| 5 | Receiver Gain | 101.0 |
| 6 | Relaxation Delay | 1.5000 |
| 7 | Pulse Width | 8.0700 |
| 8 | Spectrometer Frequency | (600.13, 150.92) |
| 9 | Spectral Width | (4717.0, 33201.9) |
| 10 | Lowest Frequency | (157.9, -1510.7) |
| 11 | Nucleus | (1H, 13C) |
| 12 | Acquired Size | (1024, 128) |
| 13 | Spectral Size | (1024, 512) |
| 14 | Digital Resolution | (4.61, 64.85) |

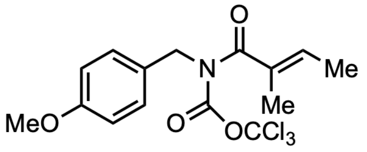


**S8**
**conformer 2**

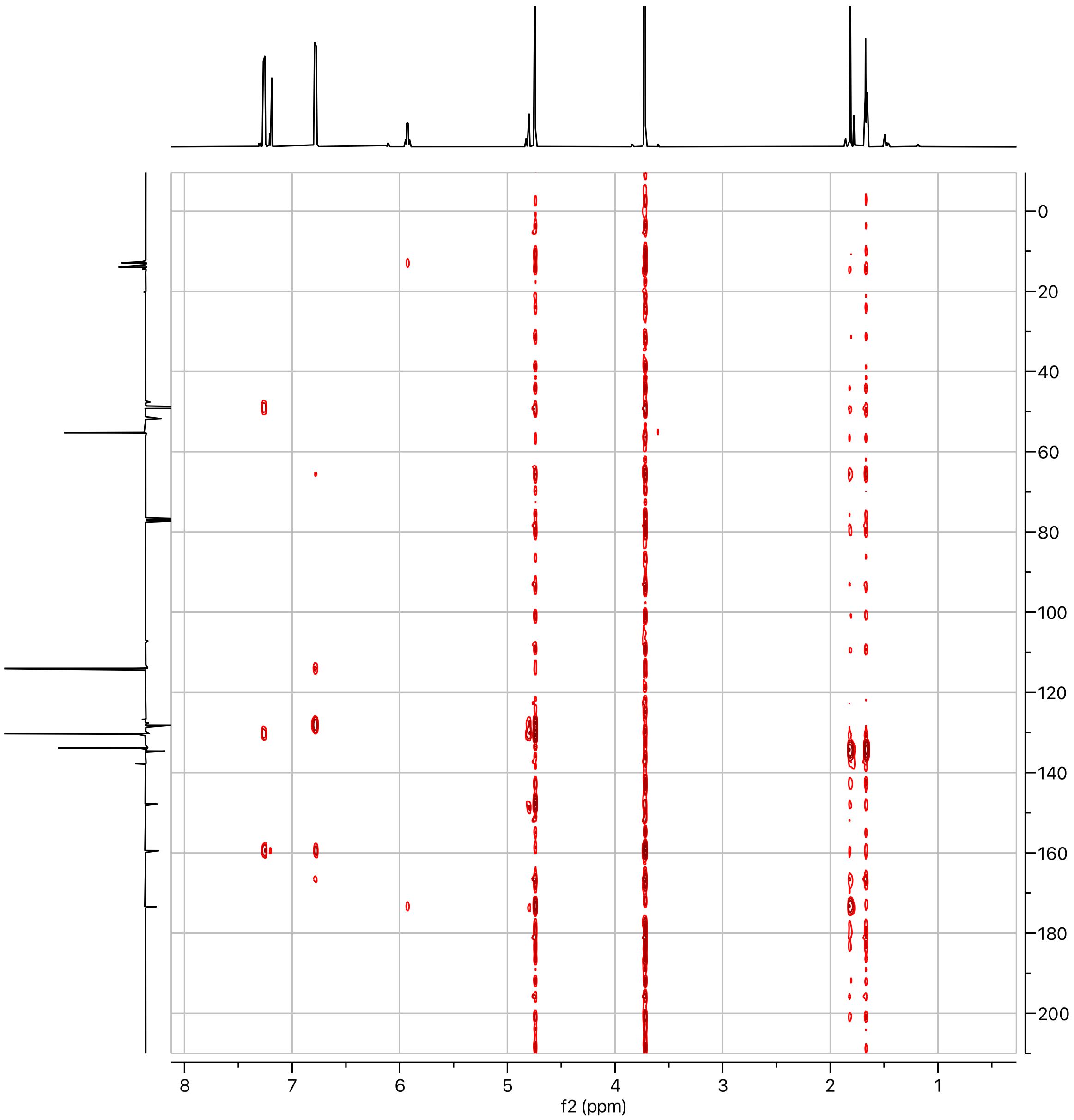

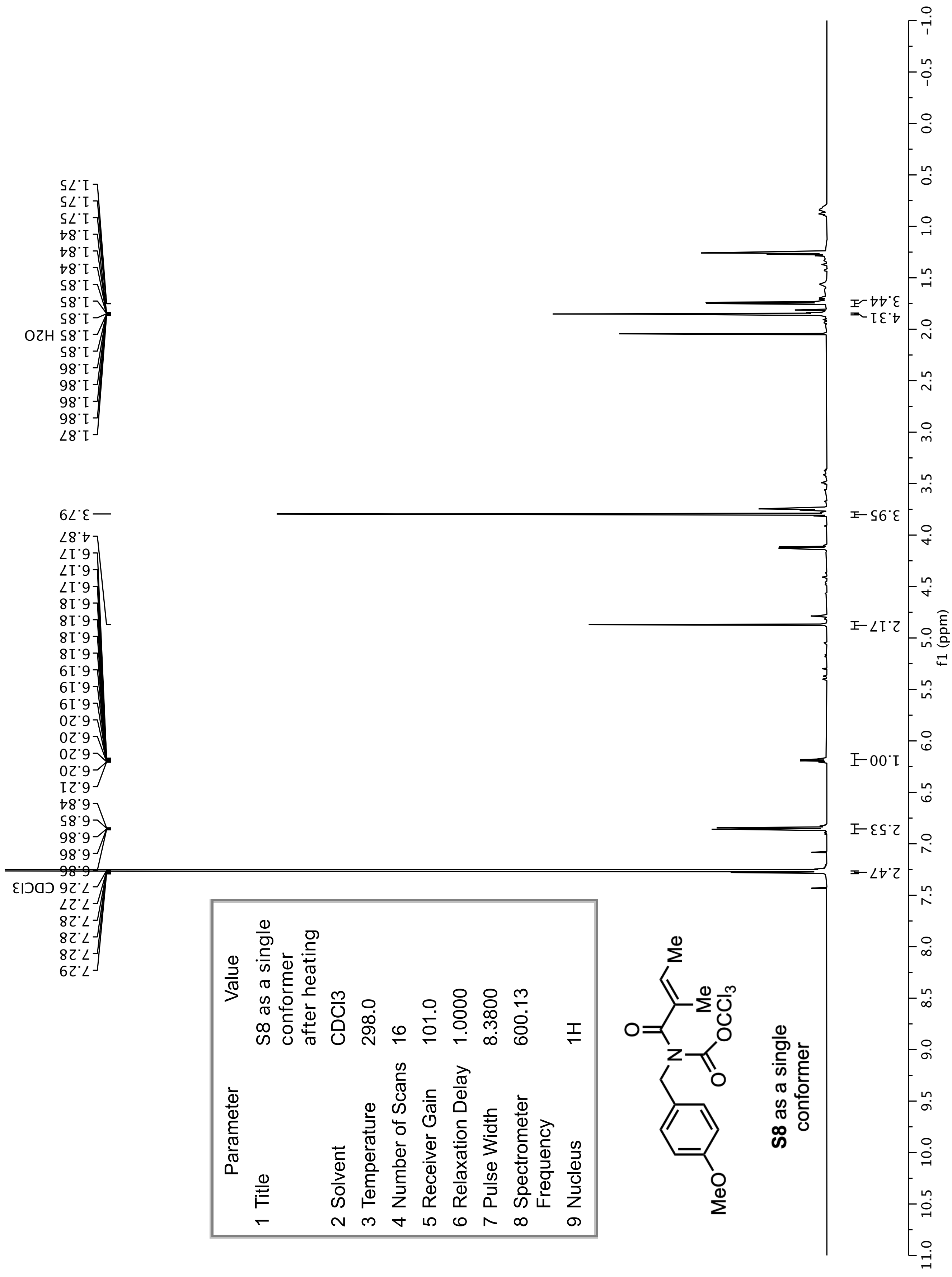


| Parameter | Value |
|---|---|
| 1 Title | S8 as a single conformer after heating |
| 2 Solvent | CDCl3 |
| 3 Temperature | 298.0 |
| 4 Number of Scans | 16 |
| 5 Receiver Gain | 101.0 |
| 6 Relaxation Delay | 1.0000 |
| 7 Pulse Width | 8.3800 |
| 8 Spectrometer Frequency | 600.13 |
| 9 Nucleus | 1H |

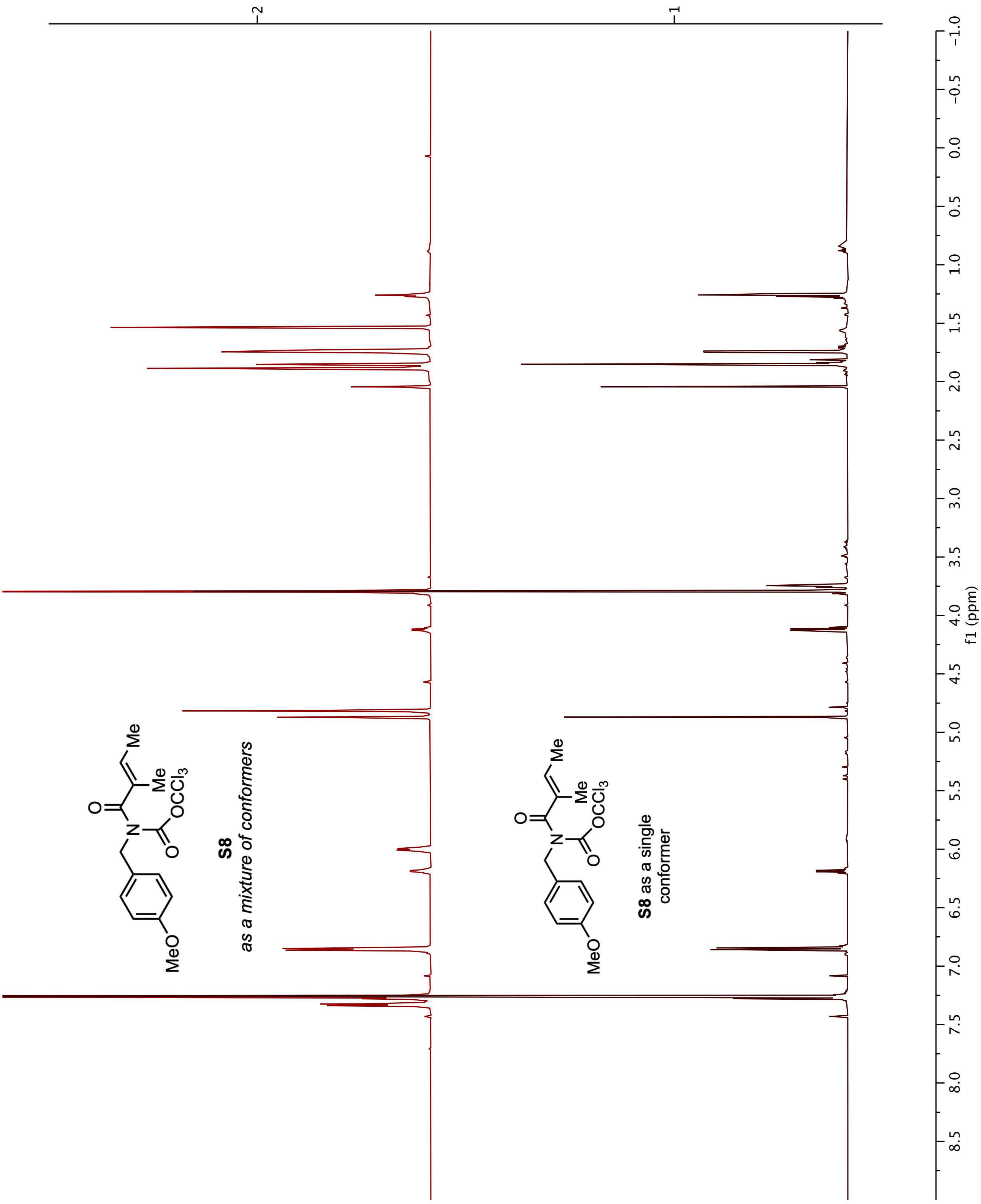
S8
as a mixture of conformers
S8 as a single
conformer
f1 (ppm)

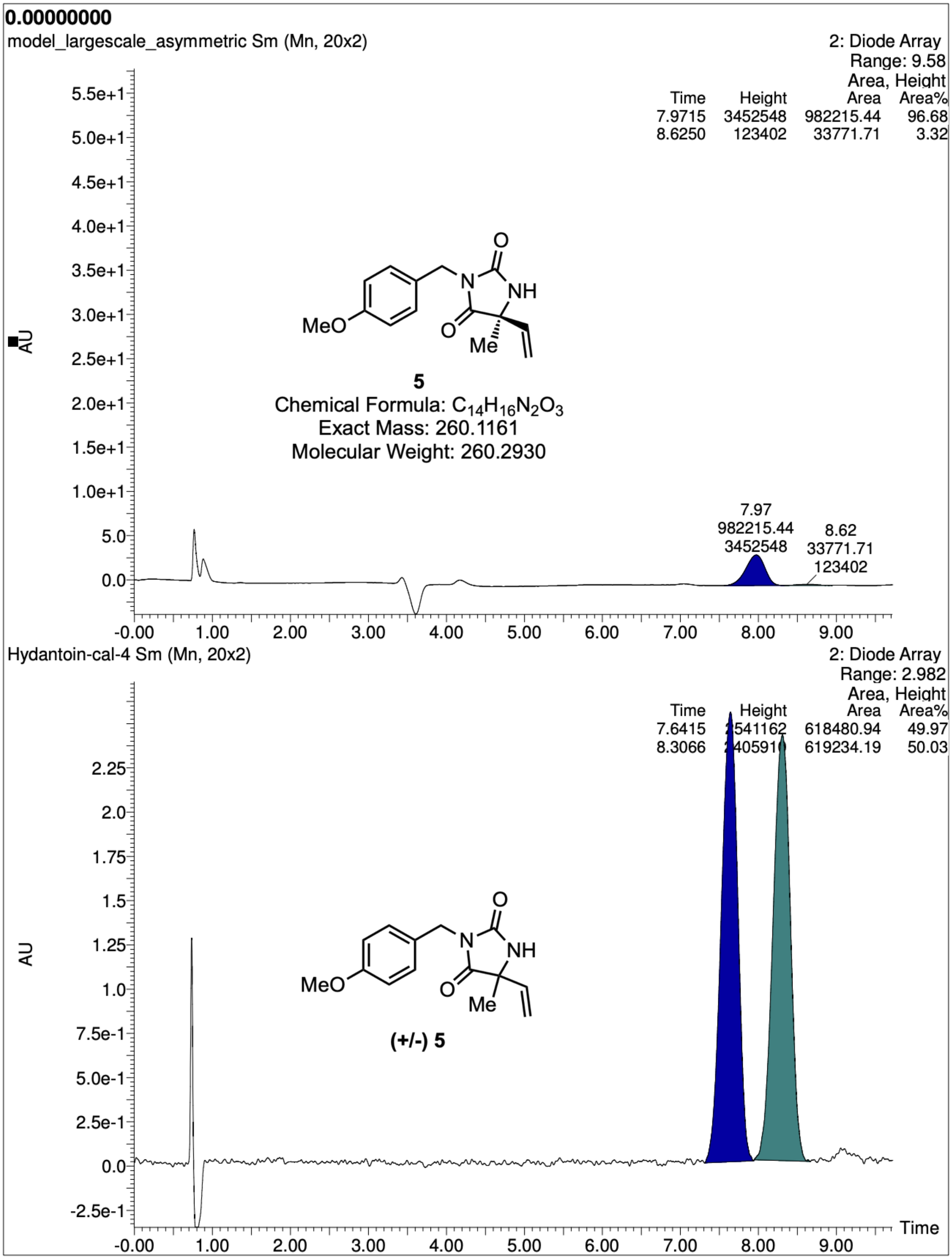

0.00000000
model_largescale_asymmetric Sm (Mn, 20x2)
2: Diode Array
Range: 9.58
Area, Height
Time Height Area Area%
7.9715 3452548 982215.44 96.68
8.6250 123402 33771.71 3.32
AU
MeO
N
O
NH
O
Me
5
Chemical Formula: $C_{14}H_{16}N_2O_3$
Exact Mass: 260.1161
Molecular Weight: 260.2930
7.97
982215.44
3452548
8.62
33771.71
123402
Hydantoin-cal-4 Sm (Mn, 20x2)
2: Diode Array
Range: 2.982
Area, Height
Time Height Area Area%
7.6415 618480.94 49.97
8.3066 619234.19 50.03
AU
(+/-) 5
Time

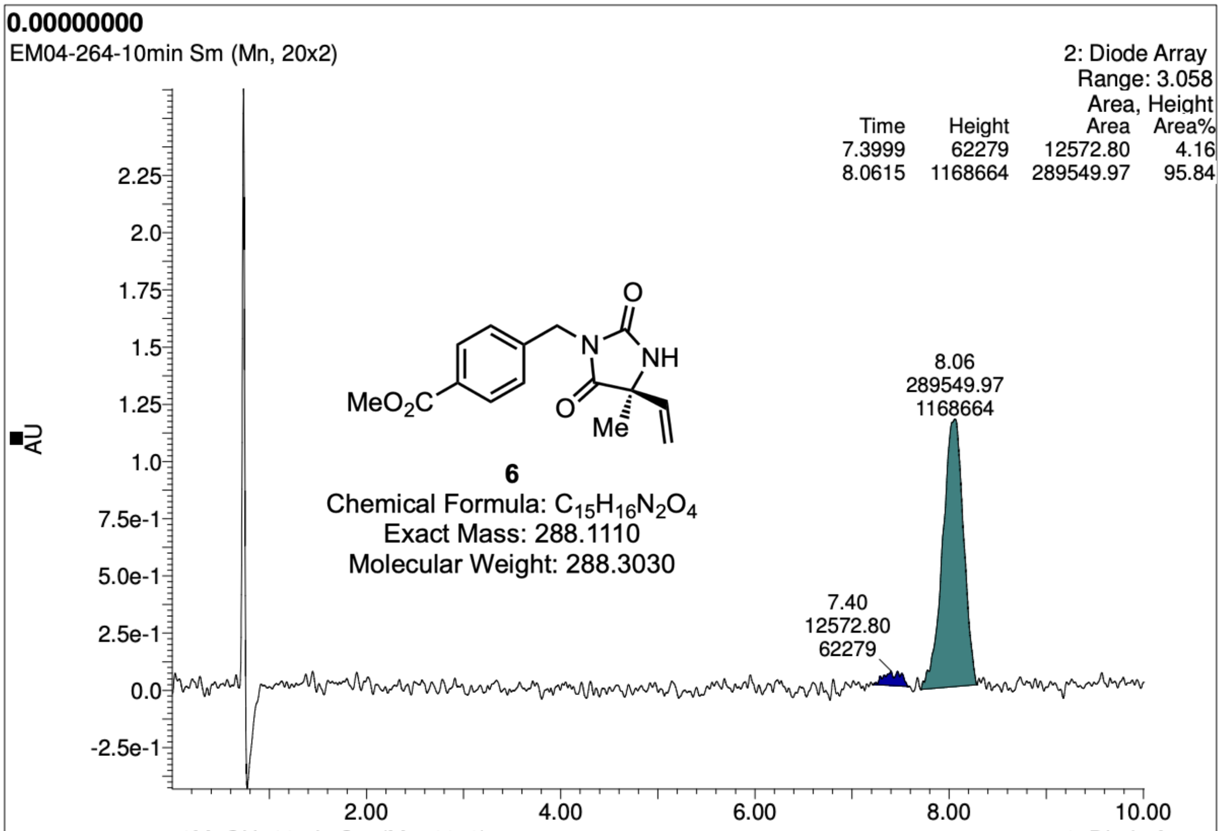
0.00000000
EM04-264-10min Sm (Mn, 20x2)
2: Diode Array
Range: 3.058
Area, Height
Time Height Area Area%
7.3999 62279 12572.80 4.16
8.0615 1168664 289549.97 95.84
MeO2C
N
NH
O
O
Me
6
Chemical Formula: C15H16N2O4
Exact Mass: 288.1110
Molecular Weight: 288.3030
7.40
12572.80
62279
8.06
289549.97
1168664
AU
2.25
2.0
1.75
1.5
1.25
1.0
7.5e-1
5.0e-1
2.5e-1
0.0
-2.5e-1
2.00
4.00
6.00
8.00
10.00

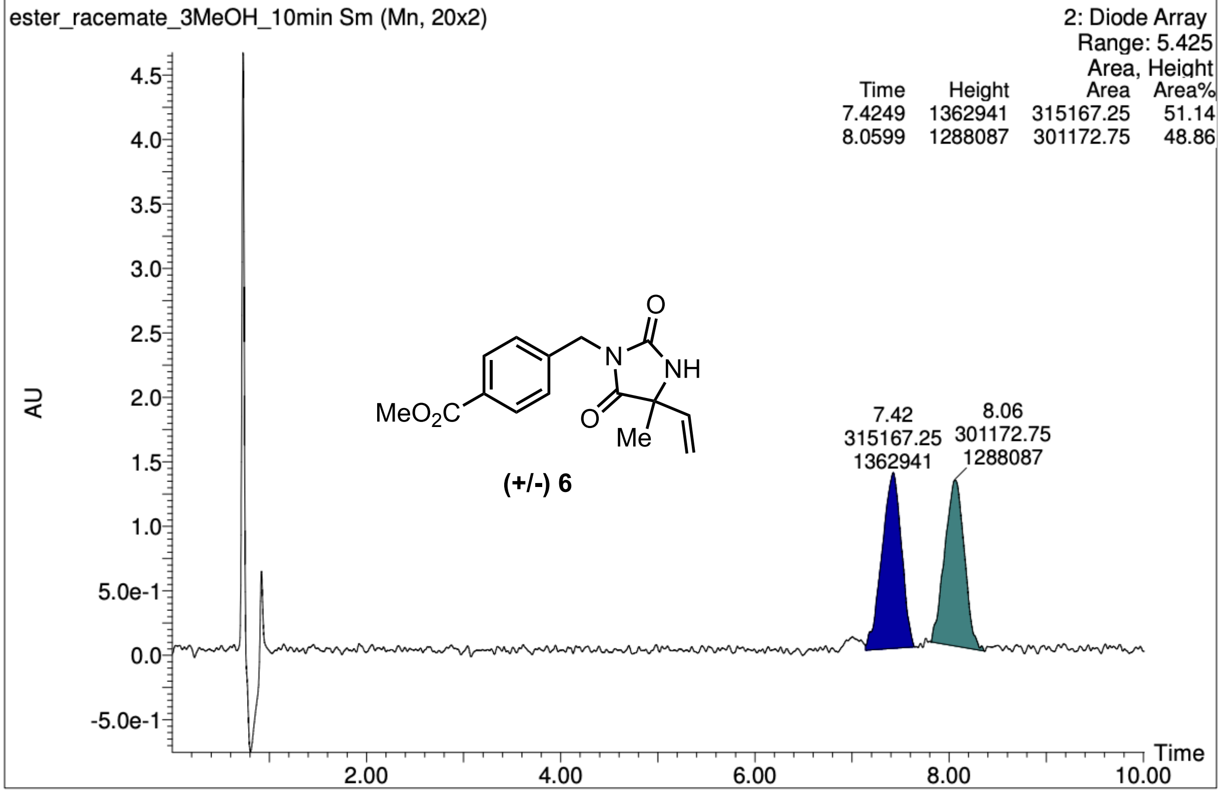
ester_racemate_3MeOH_10min Sm (Mn, 20x2)
2: Diode Array
Range: 5.425
Area, Height
Time Height Area Area%
7.4249 1362941 315167.25 51.14
8.0599 1288087 301172.75 48.86
MeO2C
N
NH
O
O
Me
(+/-) 6
7.42
315167.25
1362941
8.06
301172.75
1288087
AU
4.5
4.0
3.5
3.0
2.5
2.0
1.5
1.0
5.0e-1
0.0
-5.0e-1
Time
2.00
4.00
6.00
8.00
10.00

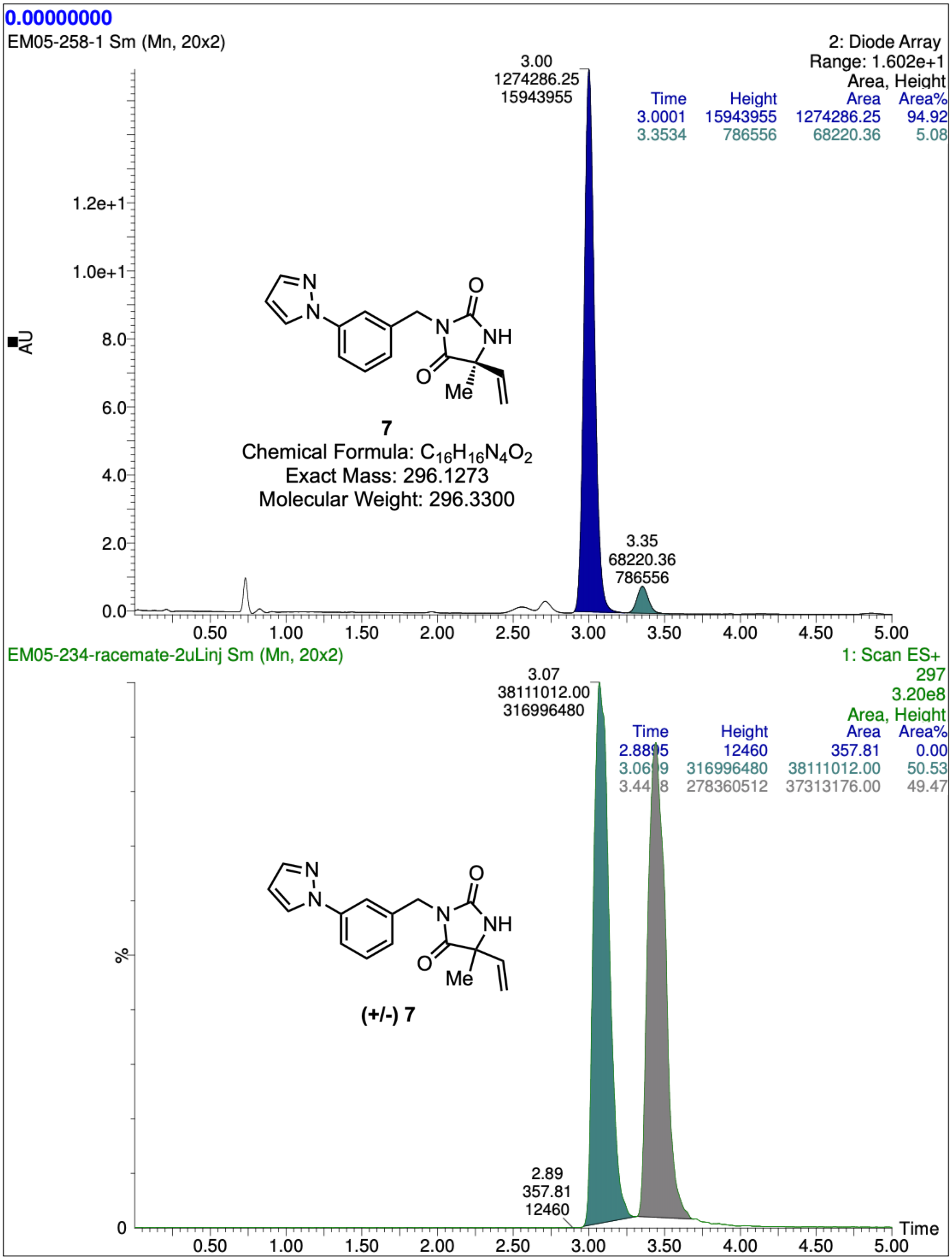
0.00000000
EM05-258-1 Sm (Mn, 20x2)
2: Diode Array
Range: 1.602e+1
Area, Height
Time Height Area Area%
3.0001 15943955 1274286.25 94.92
3.3534 786556 68220.36 5.08
3.00
1274286.25
15943955
3.35
68220.36
786556
AU
7
Chemical Formula: C16H16N4O2
Exact Mass: 296.1273
Molecular Weight: 296.3300
EM05-234-racemate-2uLinj Sm (Mn, 20x2)
1: Scan ES+
297
3.20e8
Area, Height
Time Height Area Area%
2.8895 12460 357.81 0.00
3.0699 316996480 38111012.00 50.53
3.4418 278360512 37313176.00 49.47
3.07
38111012.00
316996480
2.89
357.81
12460
%
(+/-) 7
Time

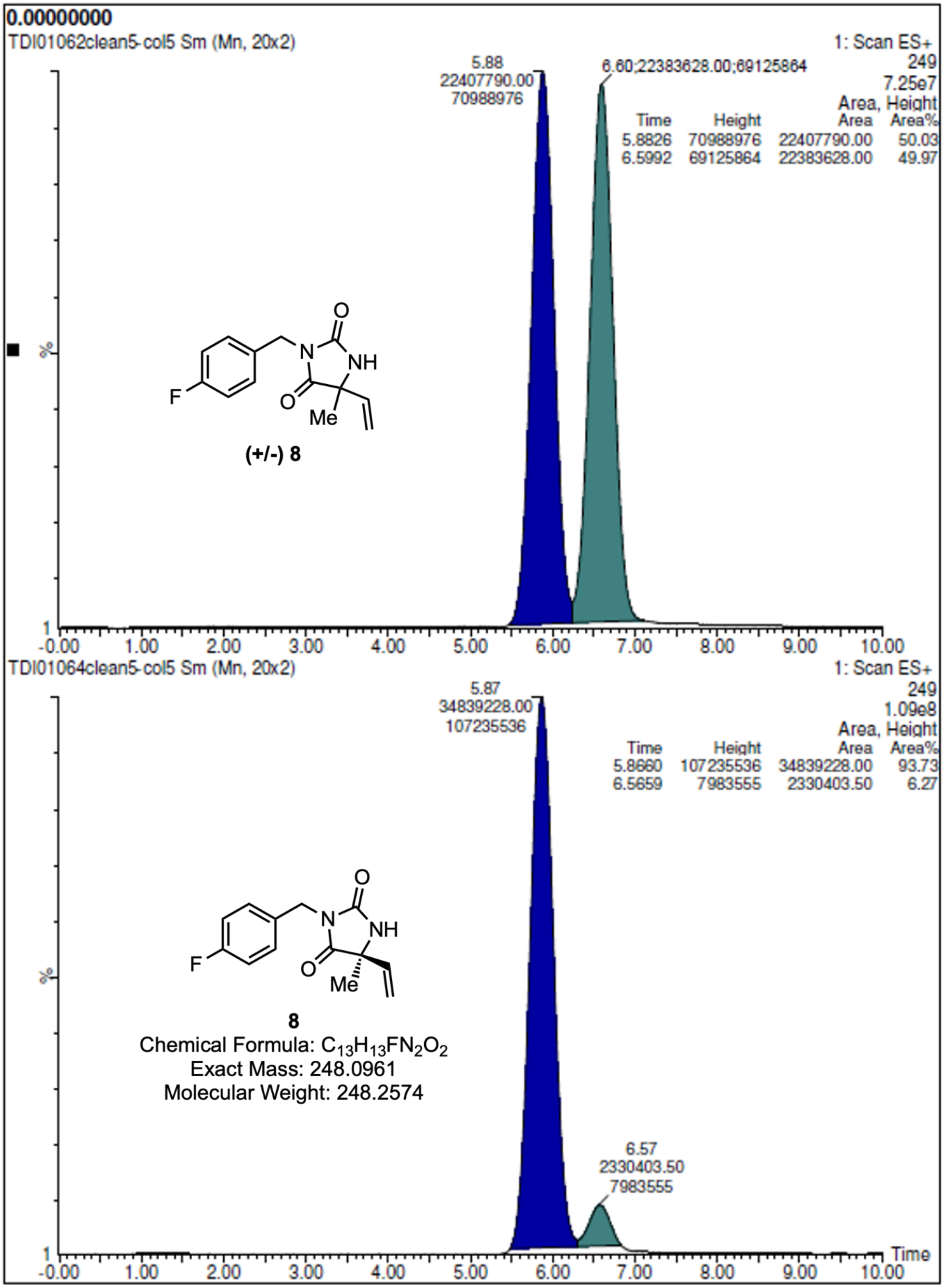
0.00000000
TDI01062clean5-col5 Sm (Mn, 20x2)
1: Scan ES+
249
7.25e7
Area, Height
5.88
22407790.00
70988976
6.60;22383628.00;69125864
Time Height Area Area%
5.8826 70988976 22407790.00 50.03
6.5992 69125864 22383628.00 49.97
%
(+/-) 8
TDI01064clean5-col5 Sm (Mn, 20x2)
1: Scan ES+
249
1.09e8
Area, Height
5.87
34839228.00
107235536
Time Height Area Area%
5.8660 107235536 34839228.00 93.73
6.5659 7983555 2330403.50 6.27
6.57
2330403.50
7983555
8
Chemical Formula: C13H13FN2O2
Exact Mass: 248.0961
Molecular Weight: 248.2574
Time
-0.00 1.00 2.00 3.00 4.00 5.00 6.00 7.00 8.00 9.00 10.00

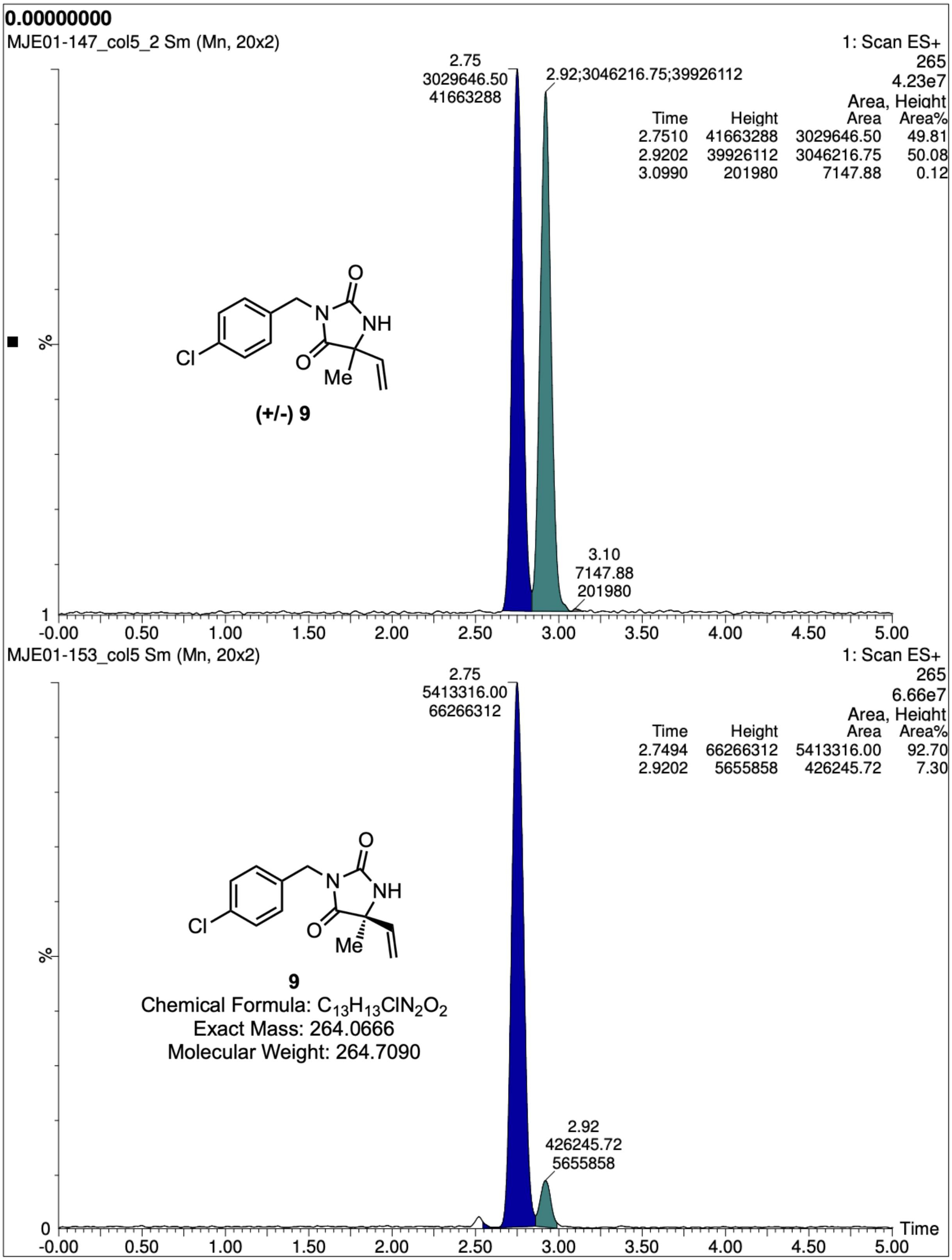
0.00000000
MJE01-147_col5_2 Sm (Mn, 20x2)
1: Scan ES+
265
4.23e7
Area, Height
Time Height Area Area%
2.7510 41663288 3029646.50 49.81
2.9202 39926112 3046216.75 50.08
3.0990 201980 7147.88 0.12
2.75
3029646.50
41663288
2.92;3046216.75;39926112
3.10
7147.88
201980
(+/-) 9
MJE01-153_col5 Sm (Mn, 20x2)
1: Scan ES+
265
6.66e7
Area, Height
Time Height Area Area%
2.7494 66266312 5413316.00 92.70
2.9202 5655858 426245.72 7.30
2.75
5413316.00
66266312
2.92
426245.72
5655858
9
Chemical Formula: $C_{13}H_{13}ClN_2O_2$
Exact Mass: 264.0666
Molecular Weight: 264.7090
Time

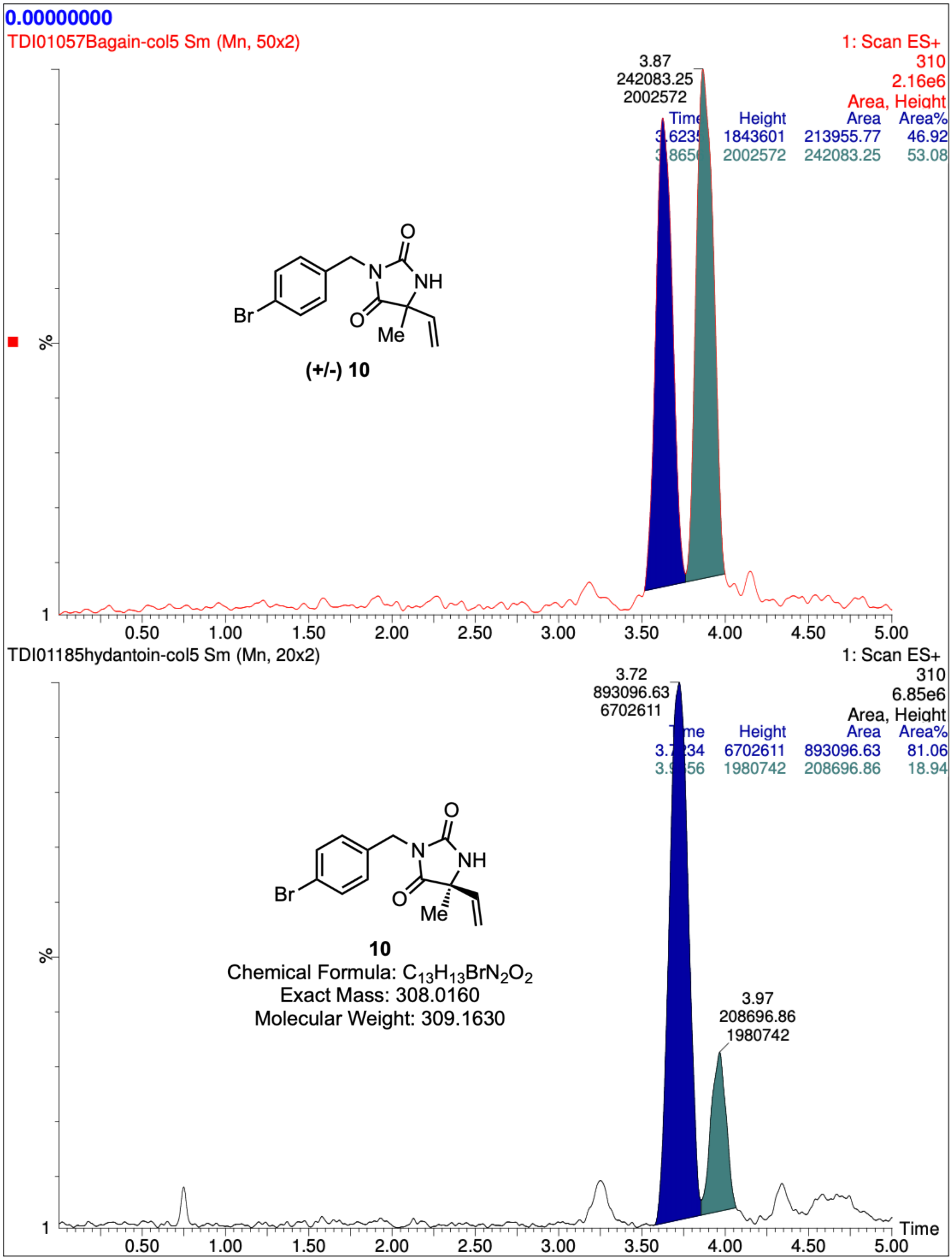
0.00000000
TDI01057Bagain-col5 Sm (Mn, 50x2)
1: Scan ES+
310
2.16e6
Area, Height
3.87
242083.25
2002572
Time Height Area Area%
1843601 213955.77 46.92
2002572 242083.25 53.08
%
Br
N
NH
O
O
Me
(+/-) 10
1
0.50 1.00 1.50 2.00 2.50 3.00 3.50 4.00 4.50 5.00
TDI01185hydantoin-col5 Sm (Mn, 20x2)
1: Scan ES+
310
6.85e6
Area, Height
3.72
893096.63
6702611
Height Area Area%
6702611 893096.63 81.06
1980742 208696.86 18.94
%
Br
N
NH
O
O
Me
10
Chemical Formula: C13H13BrN2O2
Exact Mass: 308.0160
Molecular Weight: 309.1630
3.97
208696.86
1980742
1
Time
0.50 1.00 1.50 2.00 2.50 3.00 3.50 4.00 4.50 5.00

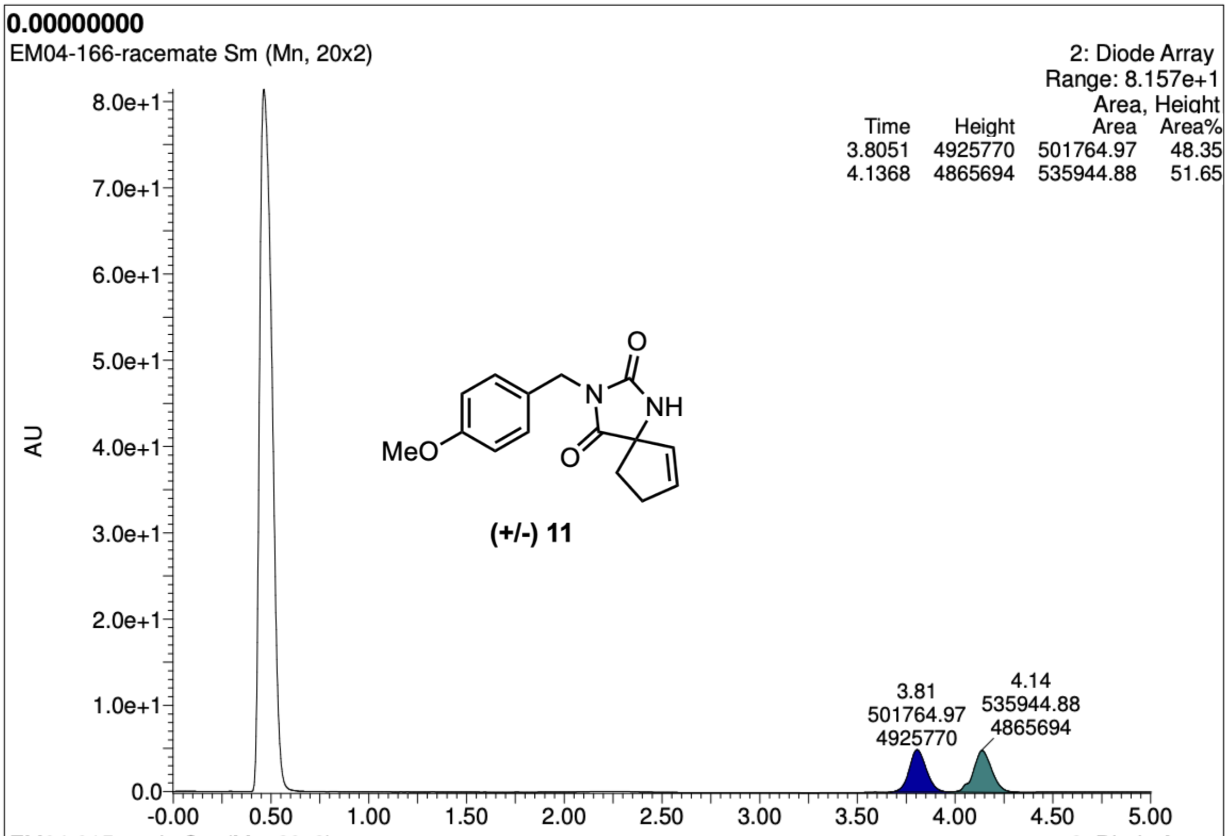
0.00000000
EM04-166-racemate Sm (Mn, 20x2)
2: Diode Array
Range: 8.157e+1
Area, Height
Time Height Area Area%
3.8051 4925770 501764.97 48.35
4.1368 4865694 535944.88 51.65
AU
8.0e+1
7.0e+1
6.0e+1
5.0e+1
4.0e+1
3.0e+1
2.0e+1
1.0e+1
0.0
MeO
N
NH
O
O
(+/-) 11
3.81
501764.97
4925770
4.14
535944.88
4865694
-0.00 0.50 1.00 1.50 2.00 2.50 3.00 3.50 4.00 4.50 5.00

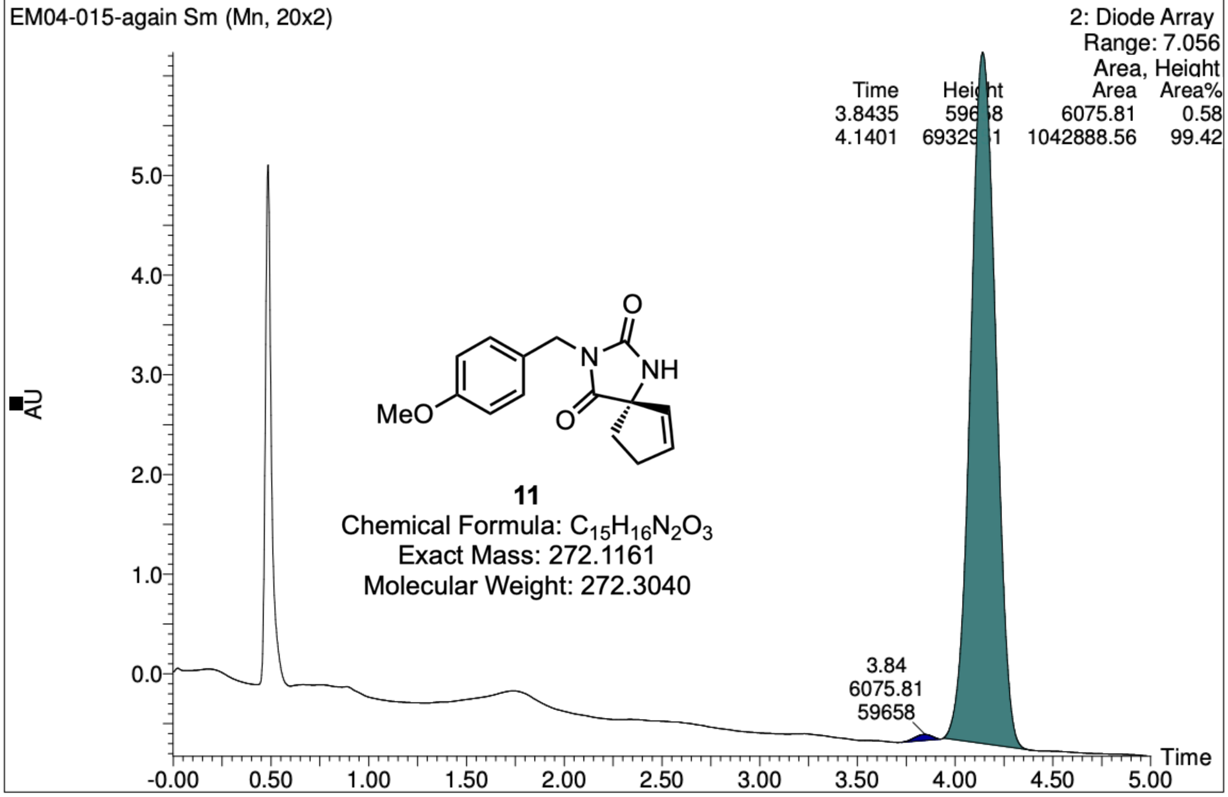
EM04-015-again Sm (Mn, 20x2)
2: Diode Array
Range: 7.056
Area, Height
Time
Hei
ht
Area Area%
3.8435
596
8
6075.81 0.58
4.1401
69329
1
1042888.56 99.42
AU
5.0
4.0
3.0
2.0
1.0
0.0
MeO
N
NH
O
O
11
Chemical Formula: C15H16N2O3
Exact Mass: 272.1161
Molecular Weight: 272.3040
3.84
6075.81
59658
Time
-0.00 0.50 1.00 1.50 2.00 2.50 3.00 3.50 4.00 4.50 5.00

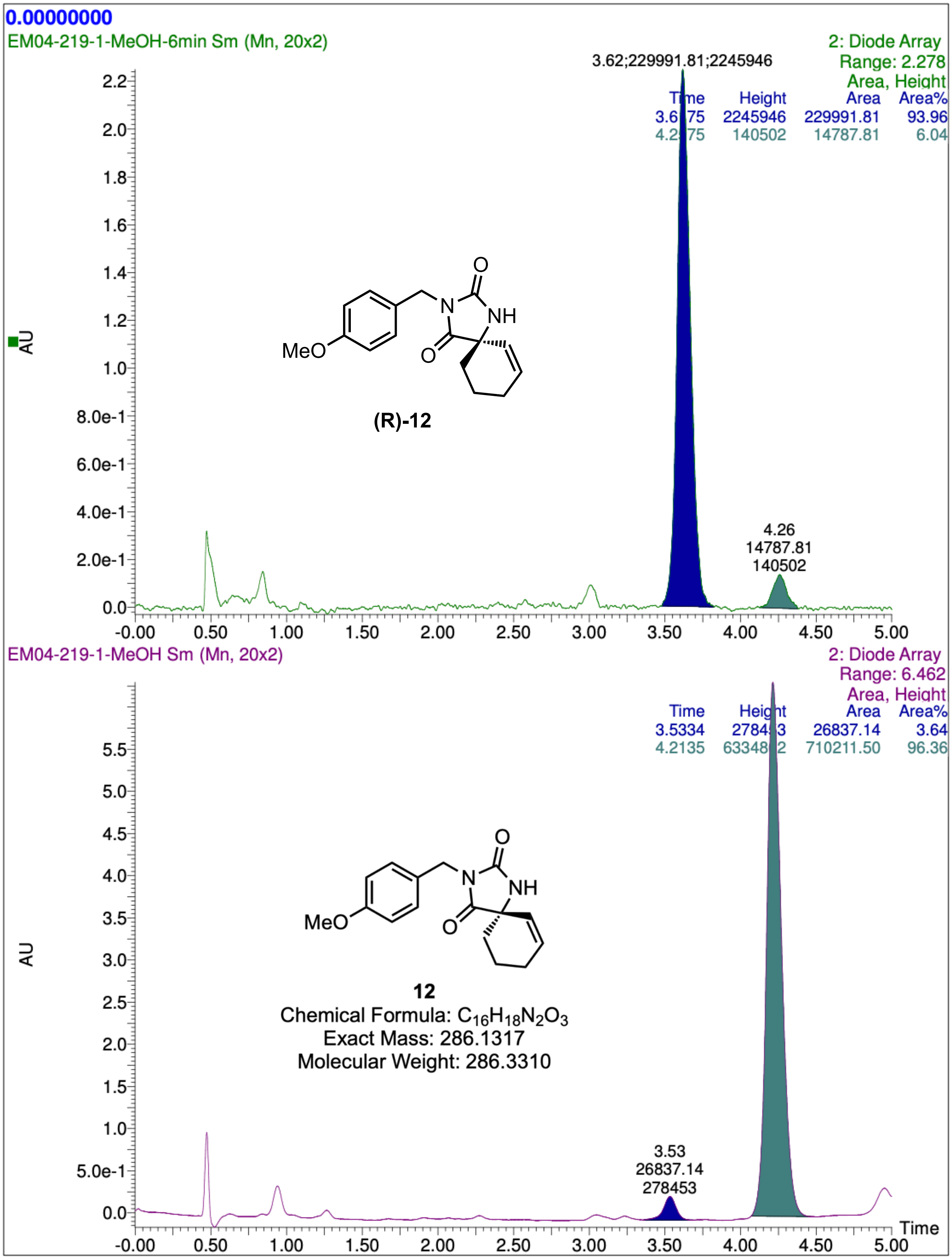
0.00000000
EM04-219-1-MeOH-6min Sm (Mn, 20x2)
2: Diode Array
Range: 2.278
Area, Height
3.62;229991.81;2245946
Height
Area
Area%
2245946
229991.81
93.96
140502
14787.81
6.04
4.26
14787.81
140502
MeO
N
NH
O
(R)-12
AU
EM04-219-1-MeOH Sm (Mn, 20x2)
2: Diode Array
Range: 6.462
Area, Height
Time
Area
Area%
3.5334
26837.14
3.64
4.2135
710211.50
96.36
3.53
26837.14
278453
12
Chemical Formula: C16H18N2O3
Exact Mass: 286.1317
Molecular Weight: 286.3310
Time

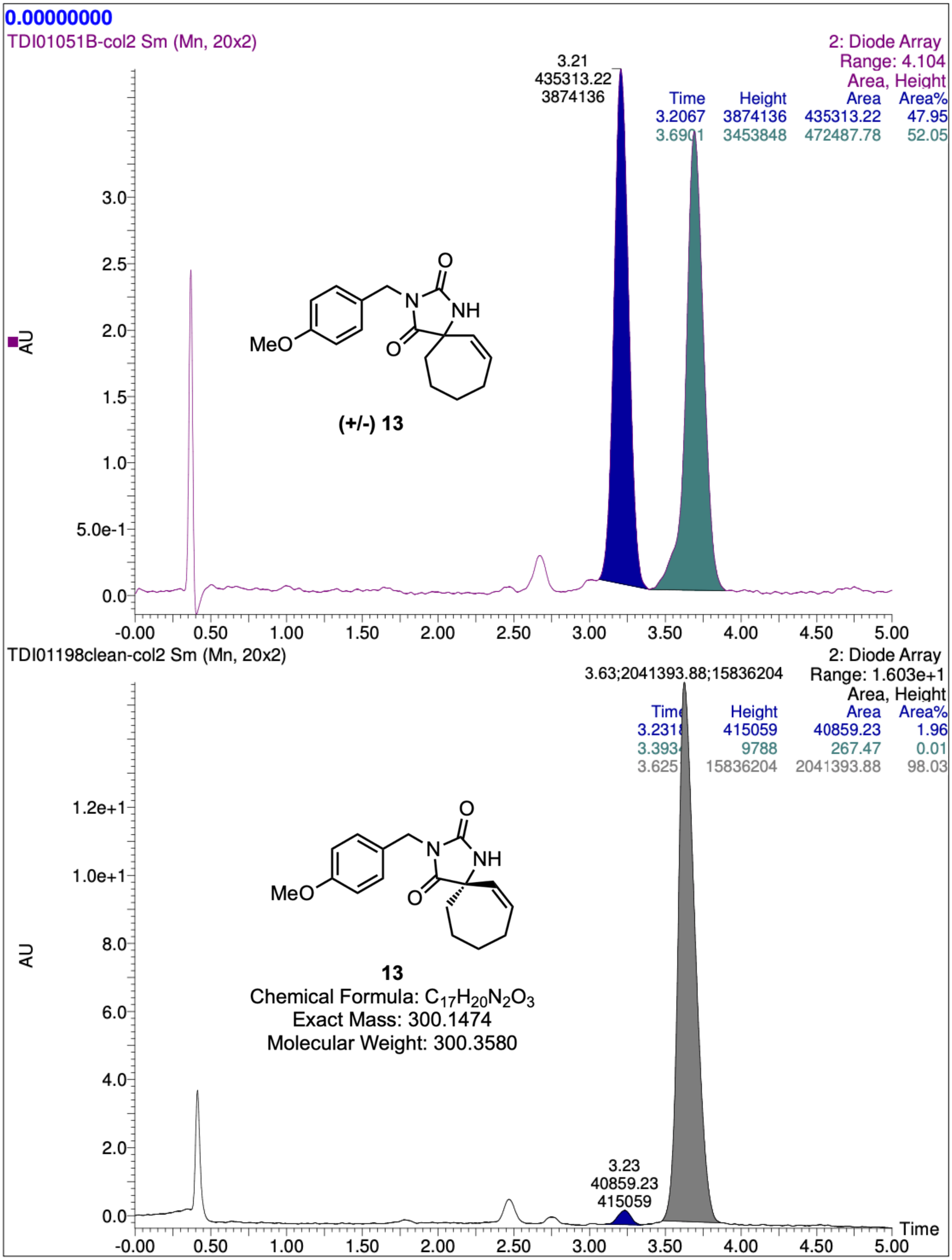
0.00000000
TDI01051B-col2 Sm (Mn, 20x2)
2: Diode Array
Range: 4.104
Area, Height
3.21
435313.22
3874136
Time Height Area Area%
3.2067 3874136 435313.22 47.95
3.6901 3453848 472487.78 52.05
AU
MeO
N
NH
O
(+/-) 13
TDI01198clean-col2 Sm (Mn, 20x2)
2: Diode Array
Range: 1.603e+1
Area, Height
3.63;2041393.88;15836204
Time Height Area Area%
3.2318 415059 40859.23 1.96
3.3934 9788 267.47 0.01
3.6251 15836204 2041393.88 98.03
13
Chemical Formula: C17H20N2O3
Exact Mass: 300.1474
Molecular Weight: 300.3580
3.23
40859.23
415059
Time

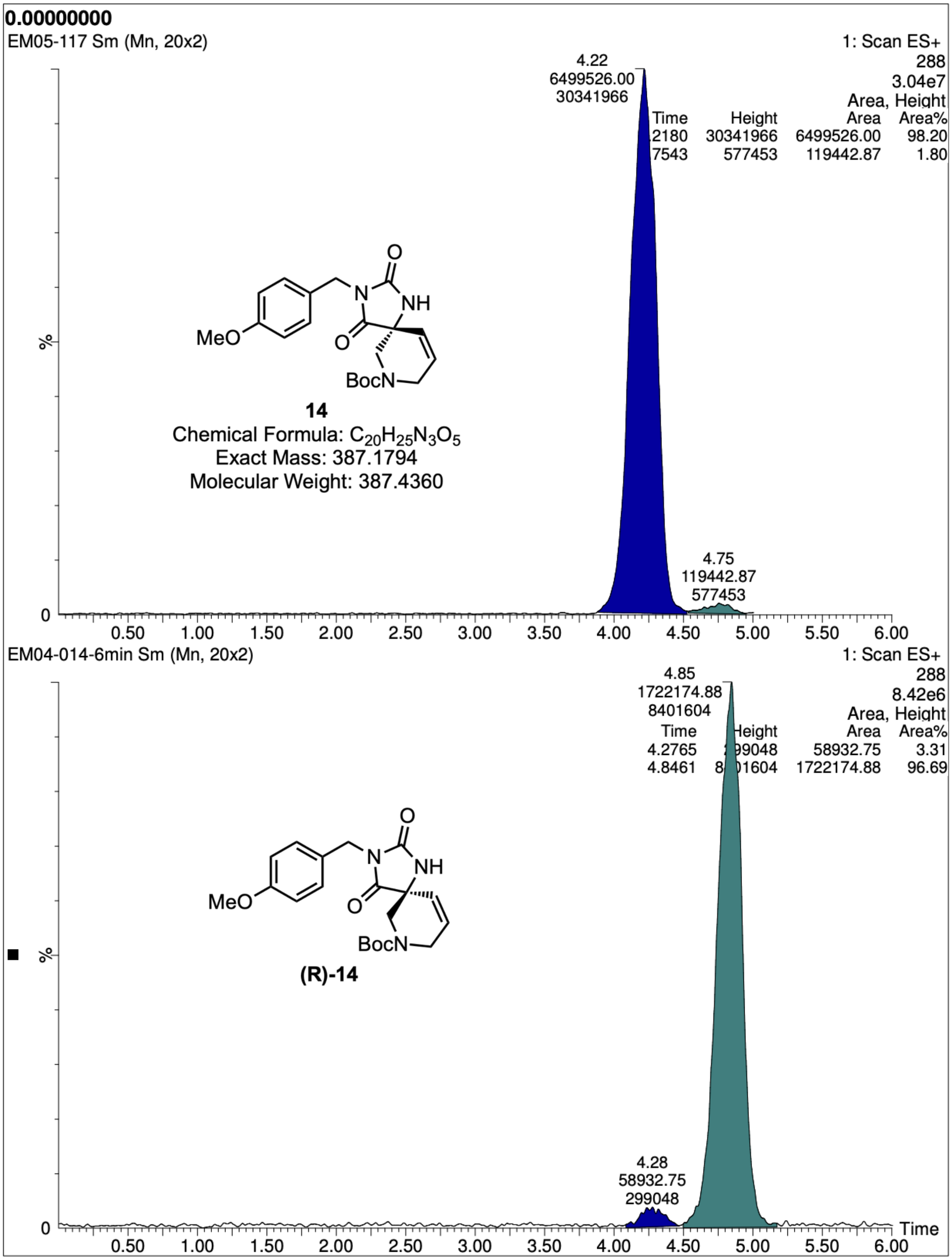
0.00000000
EM05-117 Sm (Mn, 20x2)
1: Scan ES+
288
3.04e7
Area, Height
4.22
6499526.00
30341966
Time Height Area Area%
.2180 30341966 6499526.00 98.20
.7543 577453 119442.87 1.80
MeO
N
O
NH
O
BocN
14
Chemical Formula: $C_{20}H_{25}N_3O_5$
Exact Mass: 387.1794
Molecular Weight: 387.4360
%
4.75
119442.87
577453
0
0.50 1.00 1.50 2.00 2.50 3.00 3.50 4.00 4.50 5.00 5.50 6.00
EM04-014-6min Sm (Mn, 20x2)
1: Scan ES+
288
8.42e6
Area, Height
4.85
1722174.88
8401604
Time Height Area Area%
4.2765 299048 58932.75 3.31
4.8461 8401604 1722174.88 96.69
MeO
N
O
NH
O
BocN
(R)-14
%
4.28
58932.75
299048
0
Time
0.50 1.00 1.50 2.00 2.50 3.00 3.50 4.00 4.50 5.00 5.50 6.00

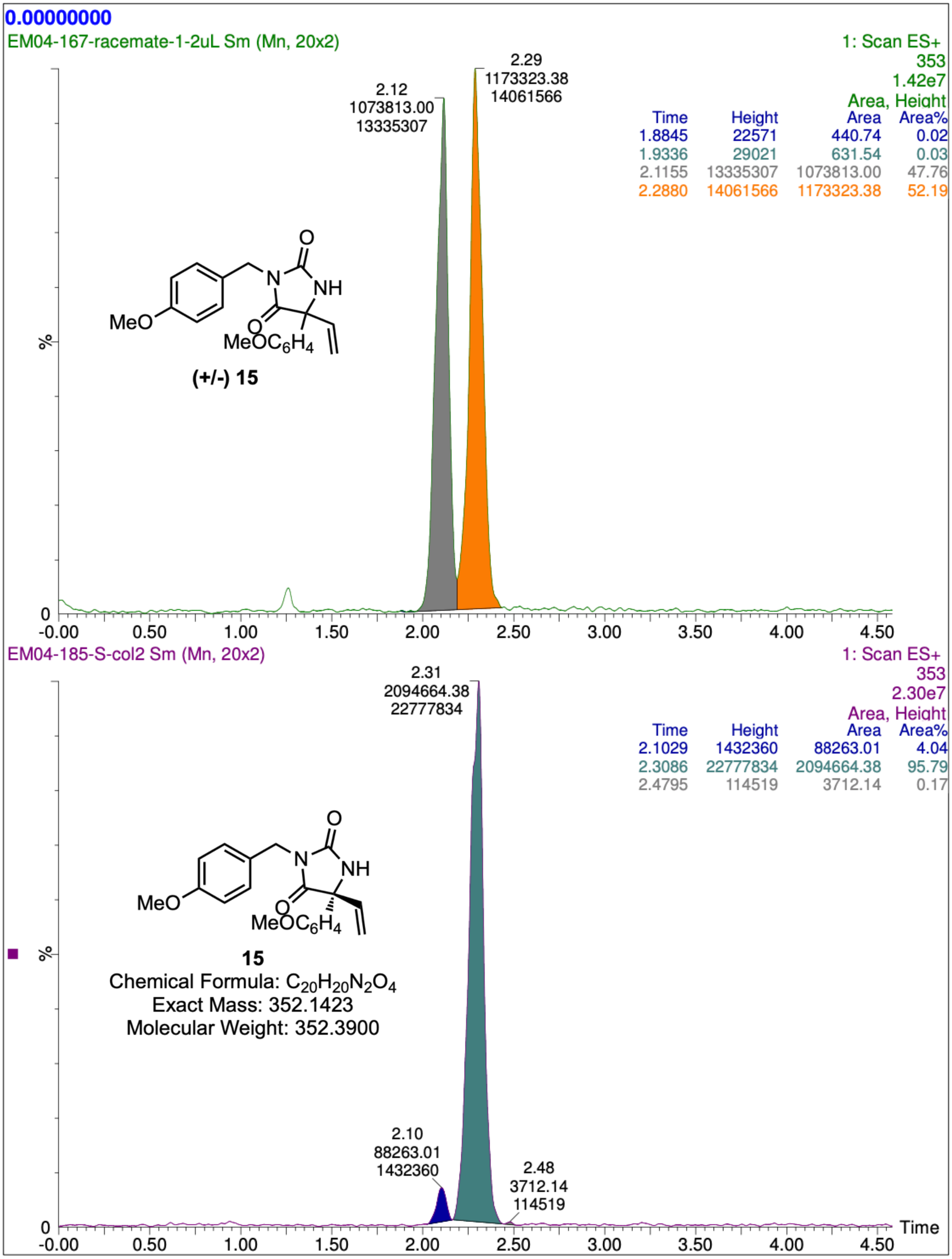
0.00000000
EM04-167-racemate-1-2uL Sm (Mn, 20x2)
1: Scan ES+
353
1.42e7
Area, Height
2.12
1073813.00
13335307
2.29
1173323.38
14061566
Time Height Area Area%
1.8845 22571 440.74 0.02
1.9336 29021 631.54 0.03
2.1155 13335307 1073813.00 47.76
2.2880 14061566 1173323.38 52.19
MeO
O
N
NH
O
MeOC6H4
(+/-) 15
%
0
-0.00 0.50 1.00 1.50 2.00 2.50 3.00 3.50 4.00 4.50
EM04-185-S-col2 Sm (Mn, 20x2)
1: Scan ES+
353
2.30e7
Area, Height
2.31
2094664.38
22777834
Time Height Area Area%
2.1029 1432360 88263.01 4.04
2.3086 22777834 2094664.38 95.79
2.4795 114519 3712.14 0.17
15
Chemical Formula: C20H20N2O4
Exact Mass: 352.1423
Molecular Weight: 352.3900
2.10
88263.01
1432360
2.48
3712.14
114519
Time

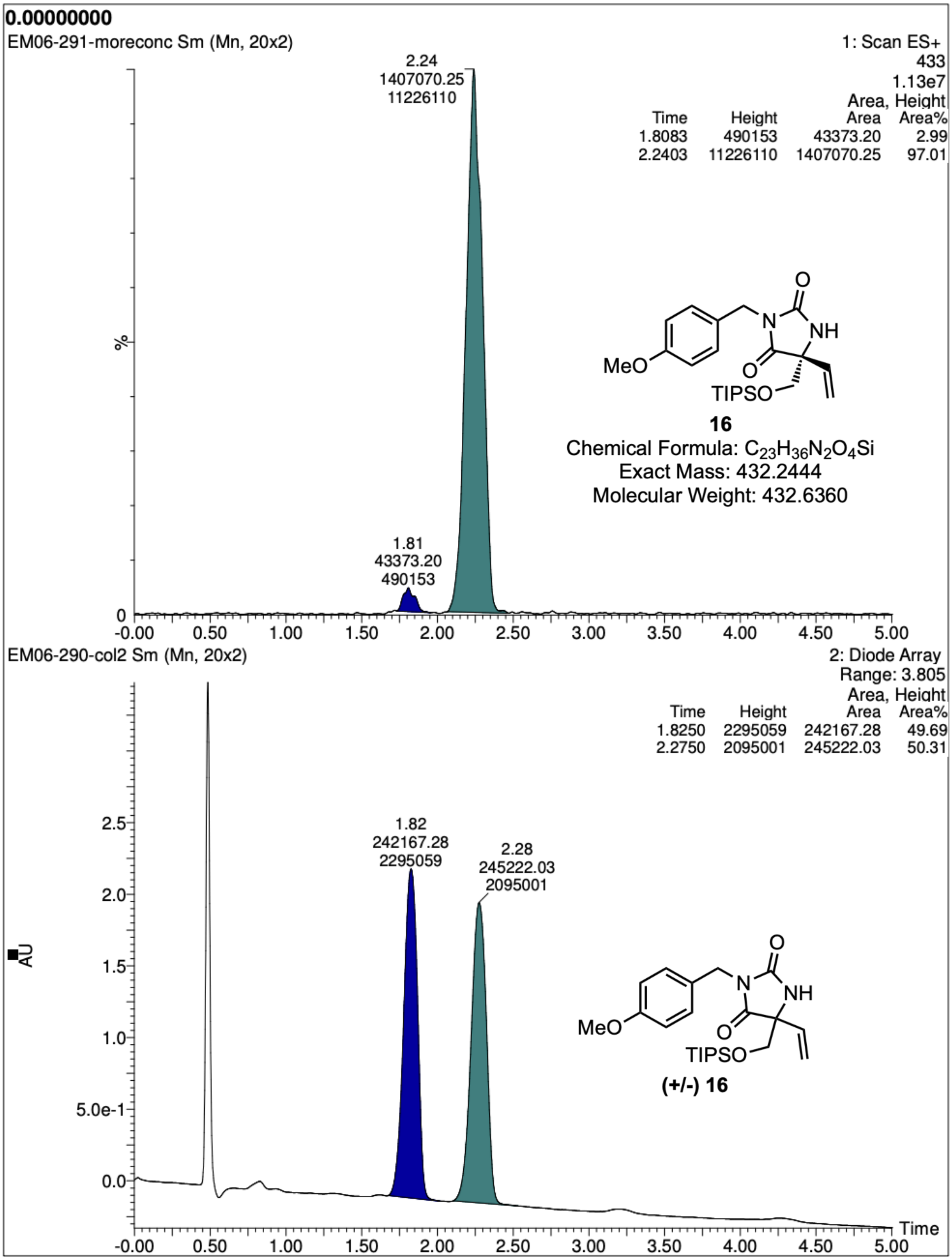
0.00000000
EM06-291-moreconc Sm (Mn, 20x2)
1: Scan ES+
433
1.13e7
Area, Height
Time Height Area Area%
1.8083 490153 43373.20 2.99
2.2403 11226110 1407070.25 97.01
2.24
1407070.25
11226110
1.81
43373.20
490153
%
16
Chemical Formula: $C_{23}H_{36}N_2O_4Si$
Exact Mass: 432.2444
Molecular Weight: 432.6360
EM06-290-col2 Sm (Mn, 20x2)
2: Diode Array
Range: 3.805
Area, Height
Time Height Area Area%
1.8250 2295059 242167.28 49.69
2.2750 2095001 245222.03 50.31
1.82
242167.28
2295059
2.28
245222.03
2095001
AU
(+/-) 16
Time

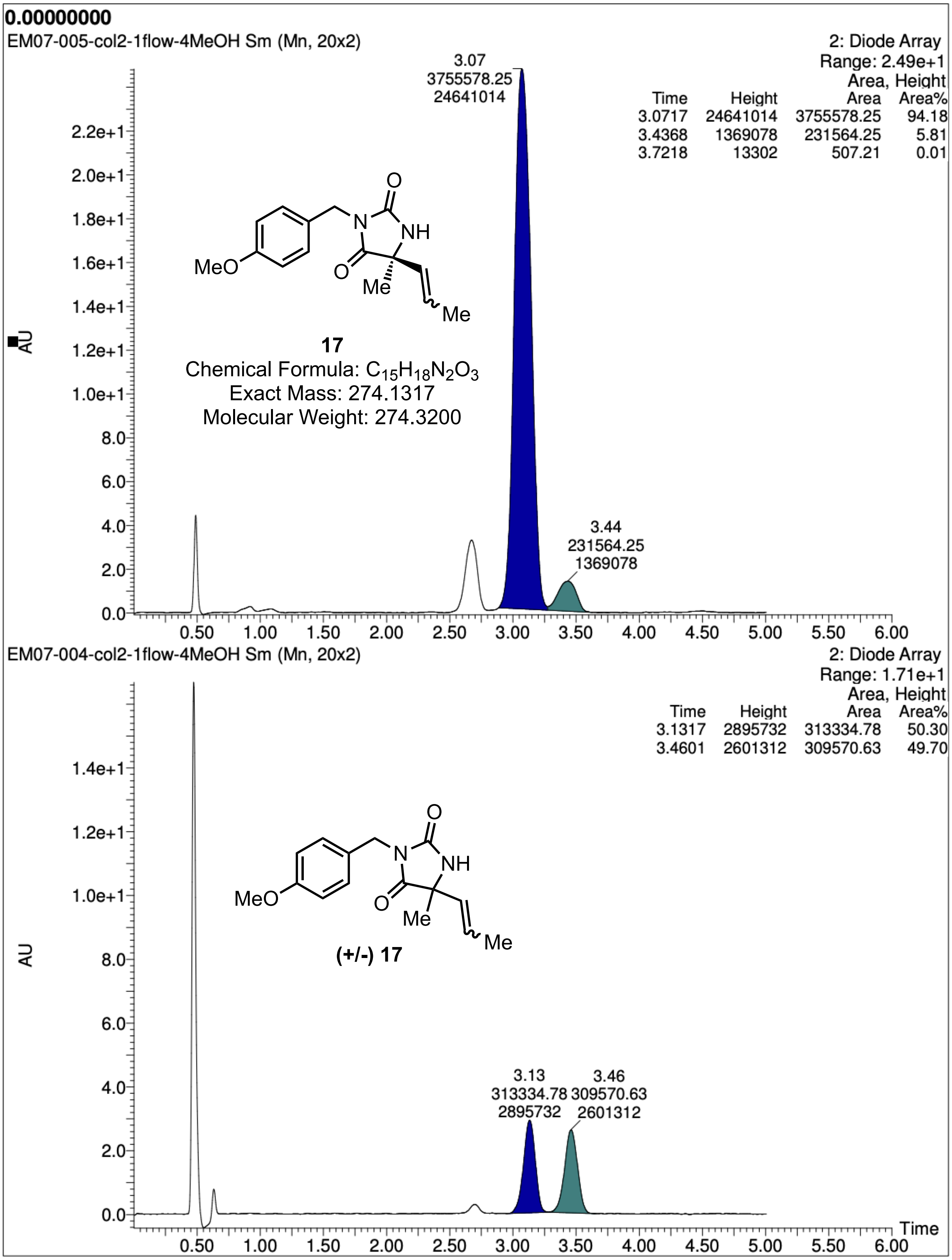
0.00000000
EM07-005-col2-1flow-4MeOH Sm (Mn, 20x2)
2: Diode Array
Range: 2.49e+1
Area, Height
Time Height Area Area%
3.0717 24641014 3755578.25 94.18
3.4368 1369078 231564.25 5.81
3.7218 13302 507.21 0.01
3.07
3755578.25
24641014
3.44
231564.25
1369078
MeO
N
NH
O
O
Me
Me
17
Chemical Formula: C15H18N2O3
Exact Mass: 274.1317
Molecular Weight: 274.3200
AU
EM07-004-col2-1flow-4MeOH Sm (Mn, 20x2)
2: Diode Array
Range: 1.71e+1
Area, Height
Time Height Area Area%
3.1317 2895732 313334.78 50.30
3.4601 2601312 309570.63 49.70
3.13
313334.78
2895732
3.46
309570.63
2601312
(+/-) 17
AU
Time

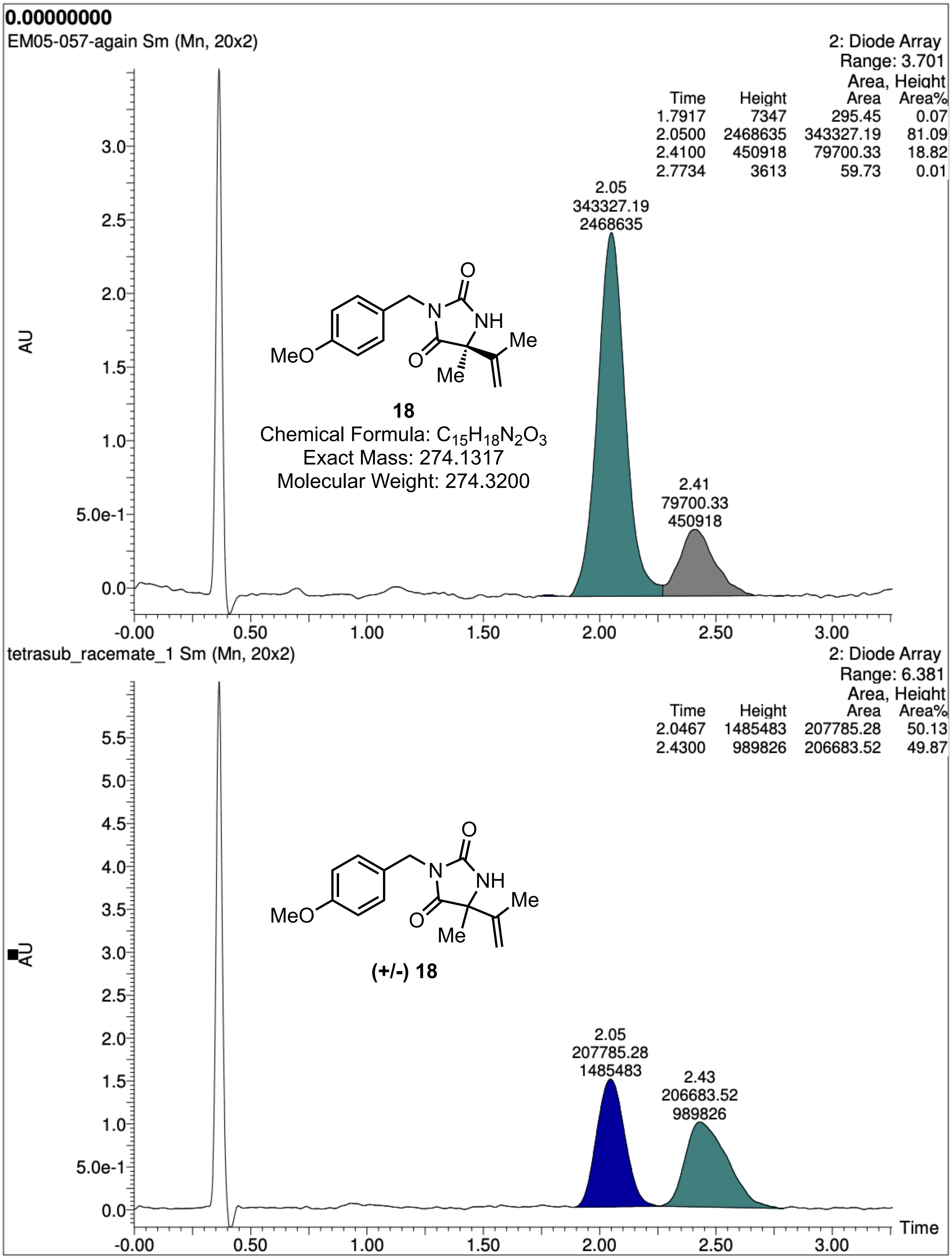
0.00000000
EM05-057-again Sm (Mn, 20x2)
2: Diode Array
Range: 3.701
Area, Height
Time Height Area Area%
1.7917 7347 295.45 0.07
2.0500 2468635 343327.19 81.09
2.4100 450918 79700.33 18.82
2.7734 3613 59.73 0.01
2.05
343327.19
2468635
2.41
79700.33
450918
AU
MeO
N
NH
O
O
Me
Me
18
Chemical Formula: C15H18N2O3
Exact Mass: 274.1317
Molecular Weight: 274.3200
tetrasub_racemate_1 Sm (Mn, 20x2)
2: Diode Array
Range: 6.381
Area, Height
Time Height Area Area%
2.0467 1485483 207785.28 50.13
2.4300 989826 206683.52 49.87
(+/-) 18
2.05
207785.28
1485483
2.43
206683.52
989826
Time

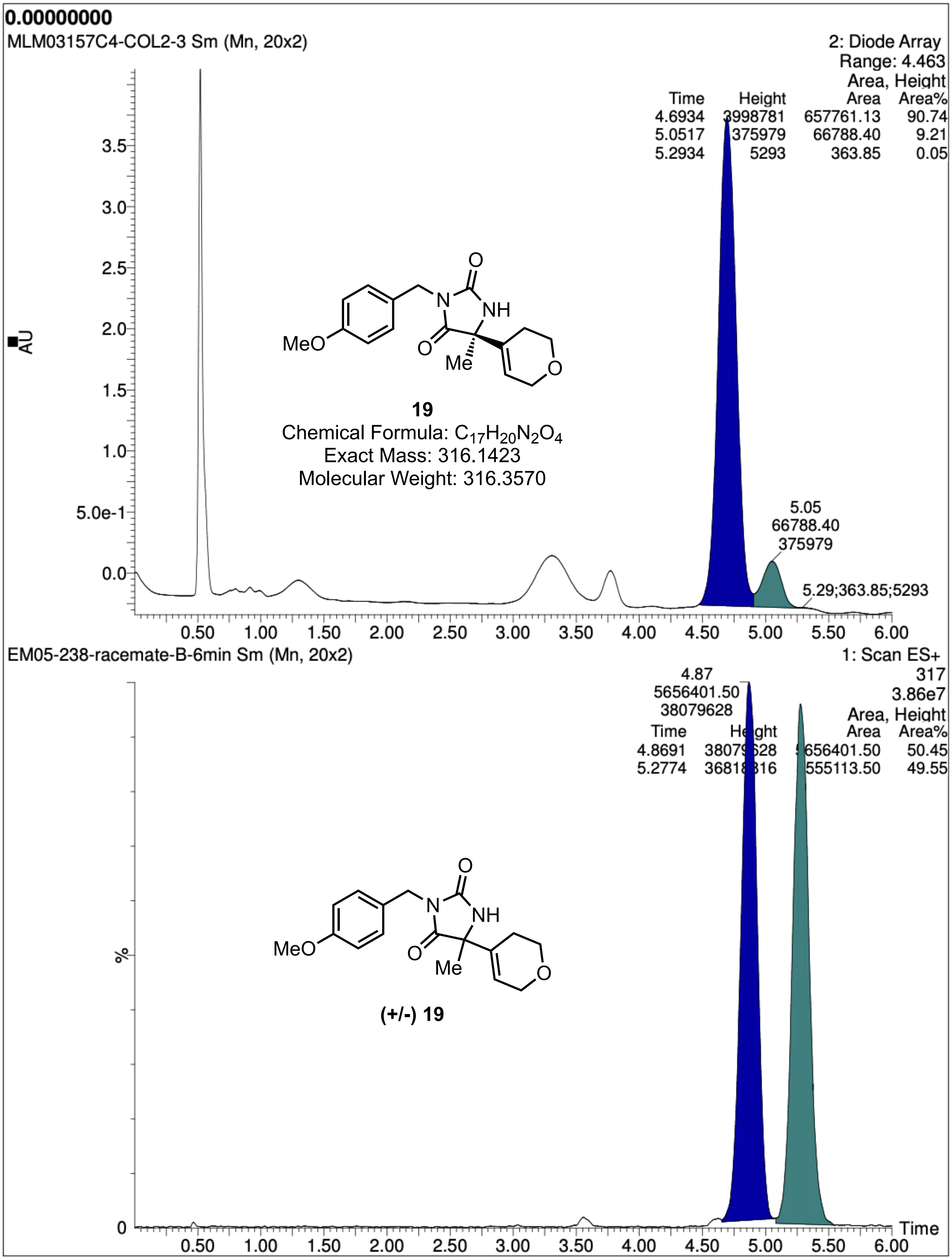
0.00000000
MLM03157C4-COL2-3 Sm (Mn, 20x2)
2: Diode Array
Range: 4.463
Area, Height
Time Height Area Area%
4.6934 3998781 657761.13 90.74
5.0517 375979 66788.40 9.21
5.2934 5293 363.85 0.05
AU
19
Chemical Formula: C17H20N2O4
Exact Mass: 316.1423
Molecular Weight: 316.3570
5.05
66788.40
375979
5.29;363.85;5293
EM05-238-racemate-B-6min Sm (Mn, 20x2)
1: Scan ES+
317
3.86e7
4.87
5656401.50
38079628
Area, Height
Time Height Area Area%
4.8691 5656401.50 50.45
5.2774 555113.50 49.55
%
(+/-) 19
Time

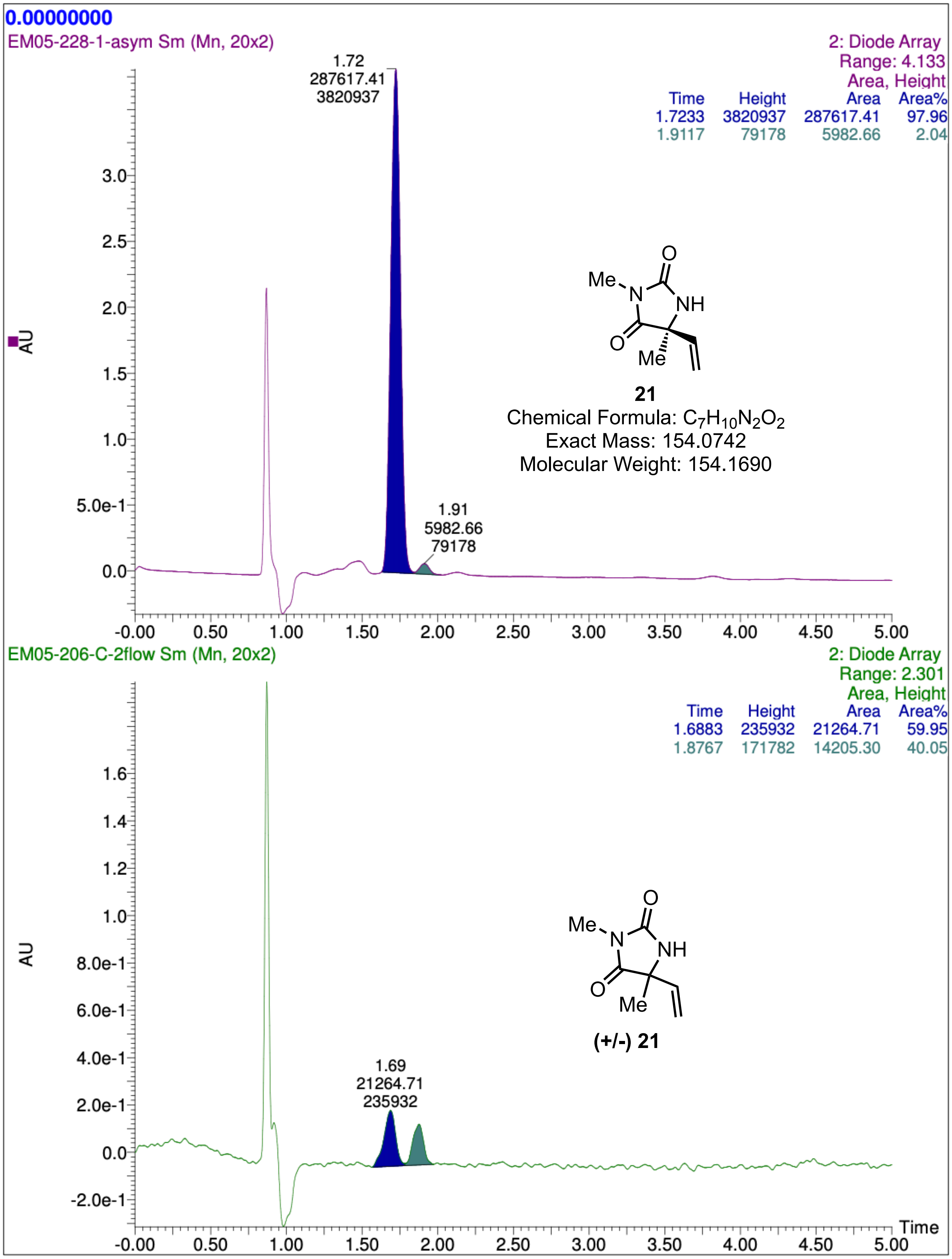
0.00000000
EM05-228-1-asym Sm (Mn, 20x2)
2: Diode Array
Range: 4.133
Area, Height
Time Height Area Area%
1.7233 3820937 287617.41 97.96
1.9117 79178 5982.66 2.04
1.72
287617.41
3820937
1.91
5982.66
79178
AU
21
Chemical Formula: $C_7H_{10}N_2O_2$
Exact Mass: 154.0742
Molecular Weight: 154.1690
EM05-206-C-2flow Sm (Mn, 20x2)
2: Diode Array
Range: 2.301
Area, Height
Time Height Area Area%
1.6883 235932 21264.71 59.95
1.8767 171782 14205.30 40.05
1.69
21264.71
235932
AU
(+/-) 21
Time

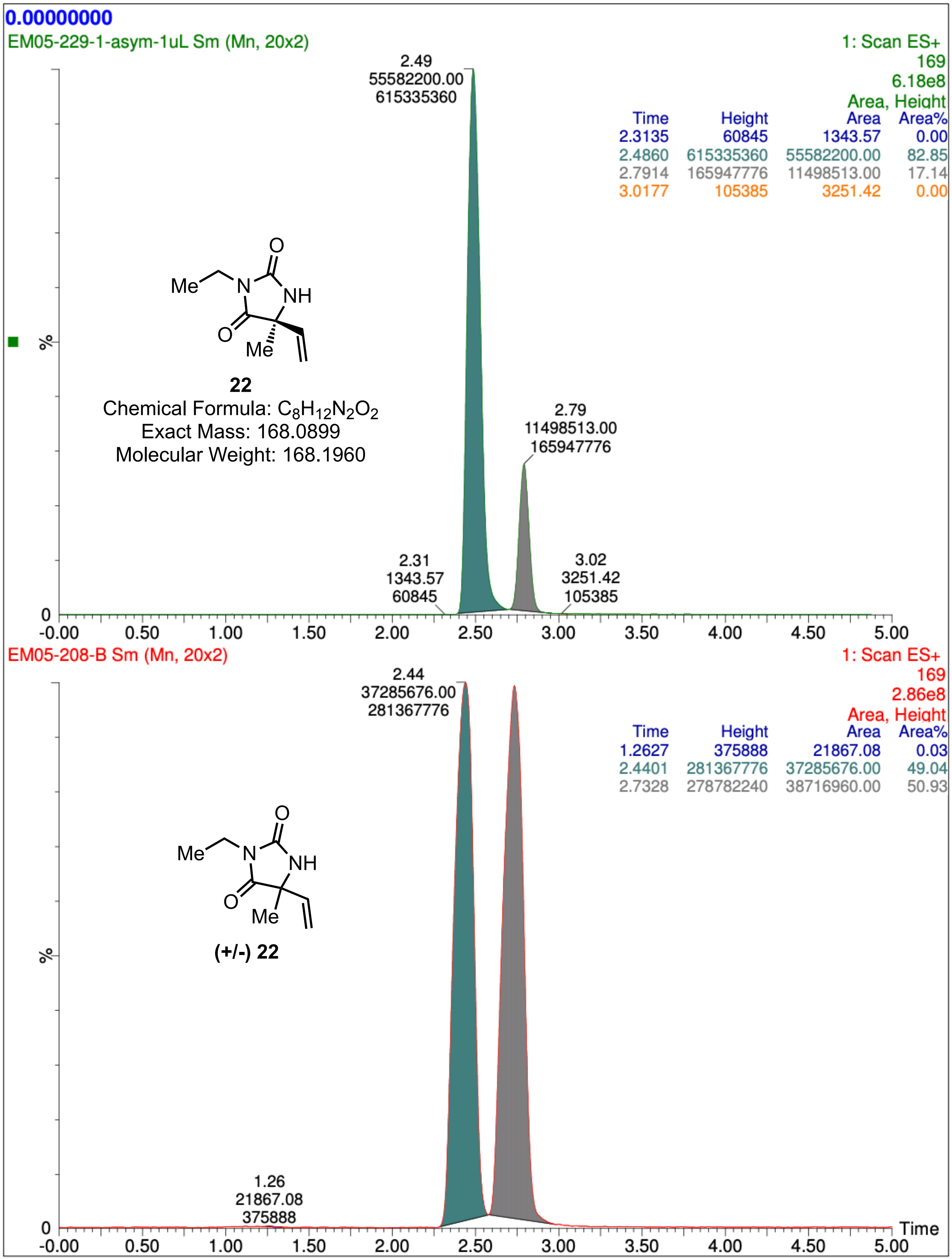


| Time | Height | Area | Area% |
|---|---|---|---|
| 2.3135 | 60845 | 1343.57 | 0.00 |
| 2.4860 | 615335360 | 55582200.00 | 82.85 |
| 2.7914 | 165947776 | 11498513.00 | 17.14 |
| 3.0177 | 105385 | 3251.42 | 0.00 |

| Time | Height | Area | Area% |
|---|---|---|---|
| 1.2627 | 375888 | 21867.08 | 0.03 |
| 2.4401 | 281367776 | 37285676.00 | 49.04 |
| 2.7328 | 278782240 | 38716960.00 | 50.93 |

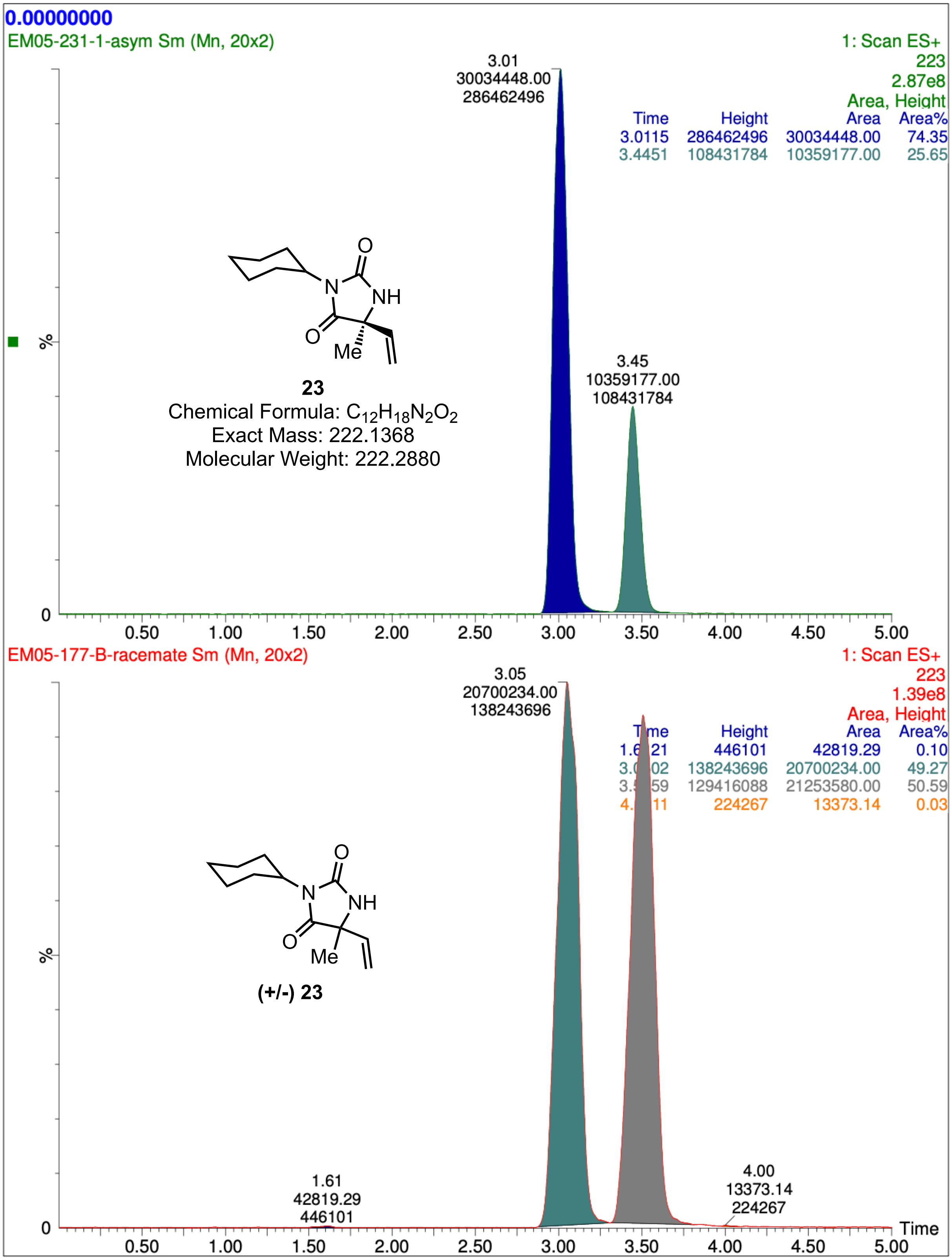
0.00000000
EM05-231-1-asym Sm (Mn, 20x2)
1: Scan ES+
223
2.87e8
Area, Height
Time Height Area Area%
3.0115 286462496 30034448.00 74.35
3.4451 108431784 10359177.00 25.65
3.01
30034448.00
286462496
3.45
10359177.00
108431784
O
N
NH
O
Me
23
Chemical Formula: C12H18N2O2
Exact Mass: 222.1368
Molecular Weight: 222.2880
%
0
0.50 1.00 1.50 2.00 2.50 3.00 3.50 4.00 4.50 5.00
EM05-177-B-racemate Sm (Mn, 20x2)
1: Scan ES+
223
1.39e8
Area, Height
Time Height Area Area%
446101 42819.29 0.10
138243696 20700234.00 49.27
129416088 21253580.00 50.59
224267 13373.14 0.03
3.05
20700234.00
138243696
1.61
42819.29
446101
4.00
13373.14
224267
(+/-) 23
%
0
Time
0.50 1.00 1.50 2.00 2.50 3.00 3.50 4.00 4.50 5.00

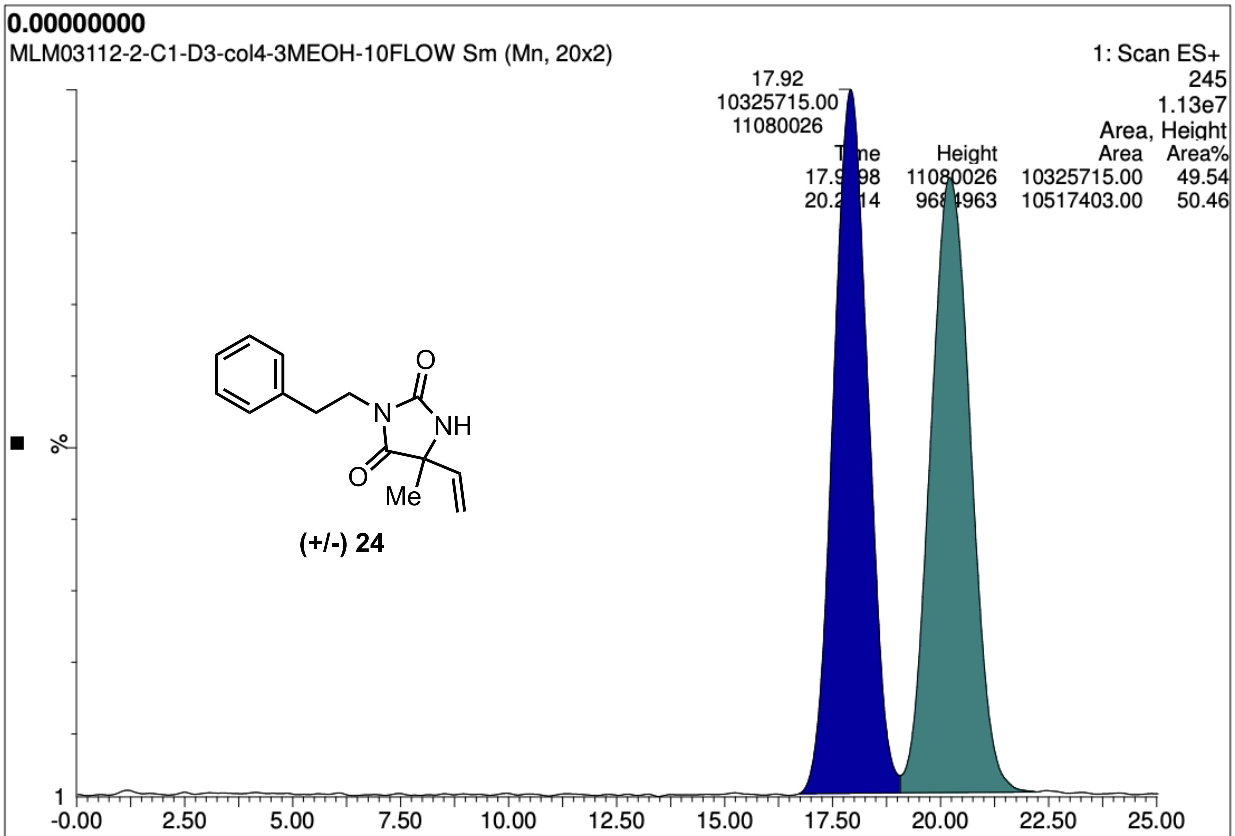
0.00000000
MLM03112-2-C1-D3-col4-3MEOH-10FLOW Sm (Mn, 20x2)
1: Scan ES+
245
1.13e7
Area, Height
17.92
10325715.00
11080026
Height
Area
Area%
10325715.00
49.54
10517403.00
50.46
%
NH
O
N
Me
(+/-) 24
-0.00
2.50
5.00
7.50
10.00
12.50
15.00
17.50
20.00
22.50
25.00

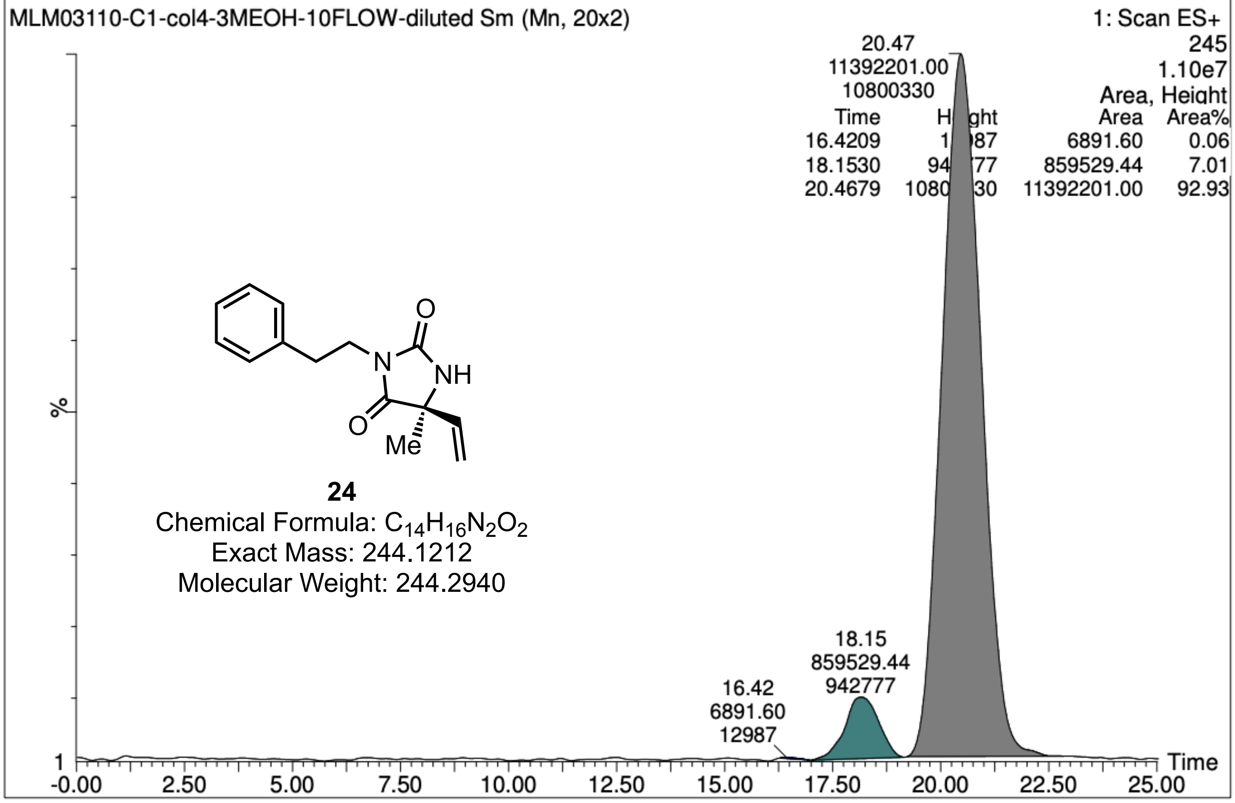
MLM03110-C1-col4-3MEOH-10FLOW-diluted Sm (Mn, 20x2)
1: Scan ES+
245
1.10e7
Area, Height
20.47
11392201.00
10800330
Time
Area
Area%
16.4209
6891.60
0.06
18.1530
859529.44
7.01
20.4679
11392201.00
92.93
%
NH
O
N
Me
24
Chemical Formula: $C_{14}H_{16}N_2O_2$
Exact Mass: 244.1212
Molecular Weight: 244.2940
18.15
859529.44
942777
16.42
6891.60
12987
Time
-0.00
2.50
5.00
7.50
10.00
12.50
15.00
17.50
20.00
22.50
25.00

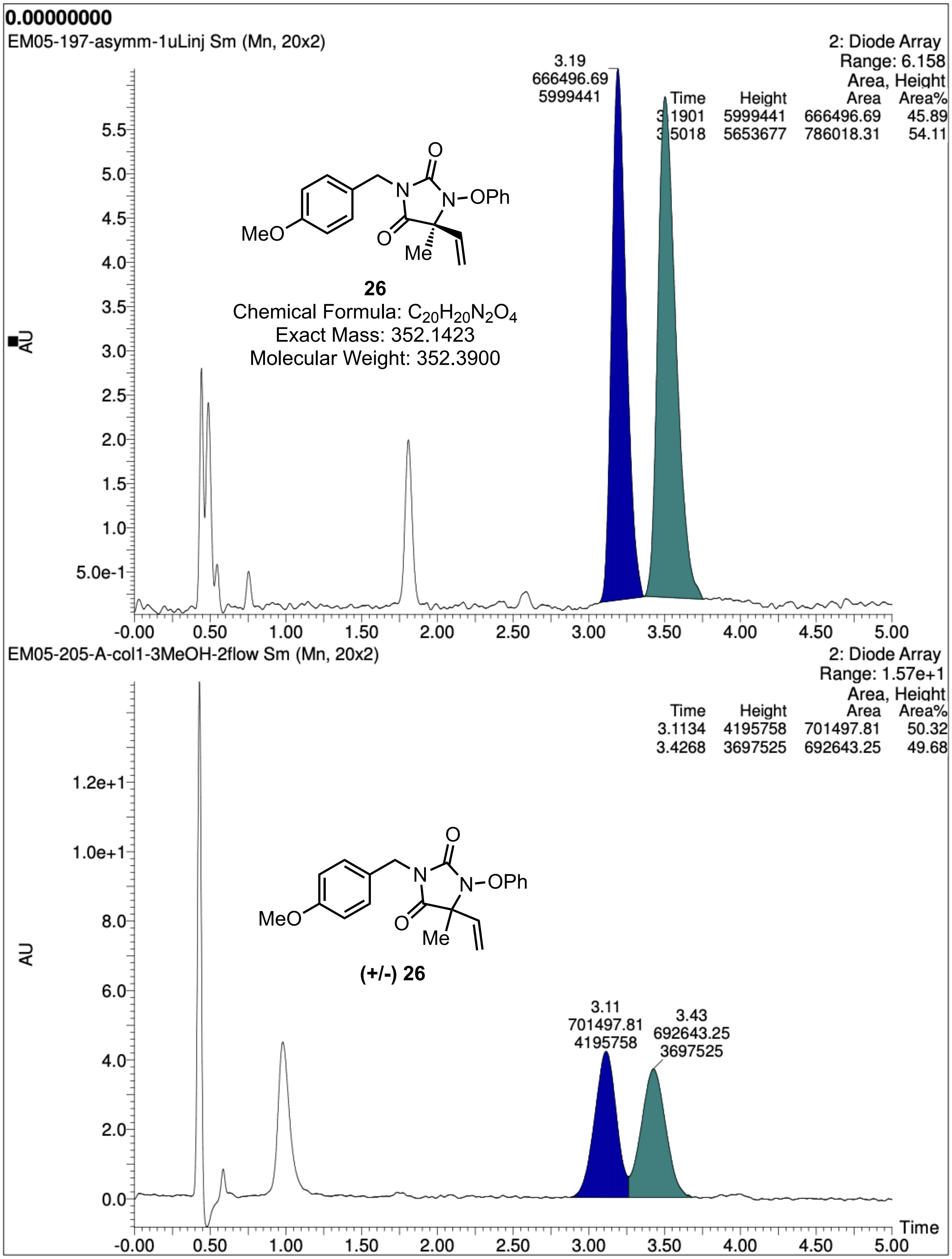
0.00000000
EM05-197-asymm-1uLinj Sm (Mn, 20x2)
2: Diode Array
Range: 6.158
Area, Height
Time Height Area Area%
3 1901 5999441 666496.69 45.89
3 5018 5653677 786018.31 54.11
3.19
666496.69
5999441
MeO
N
O
N-OPh
O
Me
26
Chemical Formula: $C_{20}H_{20}N_2O_4$
Exact Mass: 352.1423
Molecular Weight: 352.3900
AU
EM05-205-A-col1-3MeOH-2flow Sm (Mn, 20x2)
2: Diode Array
Range: 1.57e+1
Area, Height
Time Height Area Area%
3.1134 4195758 701497.81 50.32
3.4268 3697525 692643.25 49.68
(+/-) 26
3.11
701497.81
4195758
3.43
692643.25
3697525
AU
Time

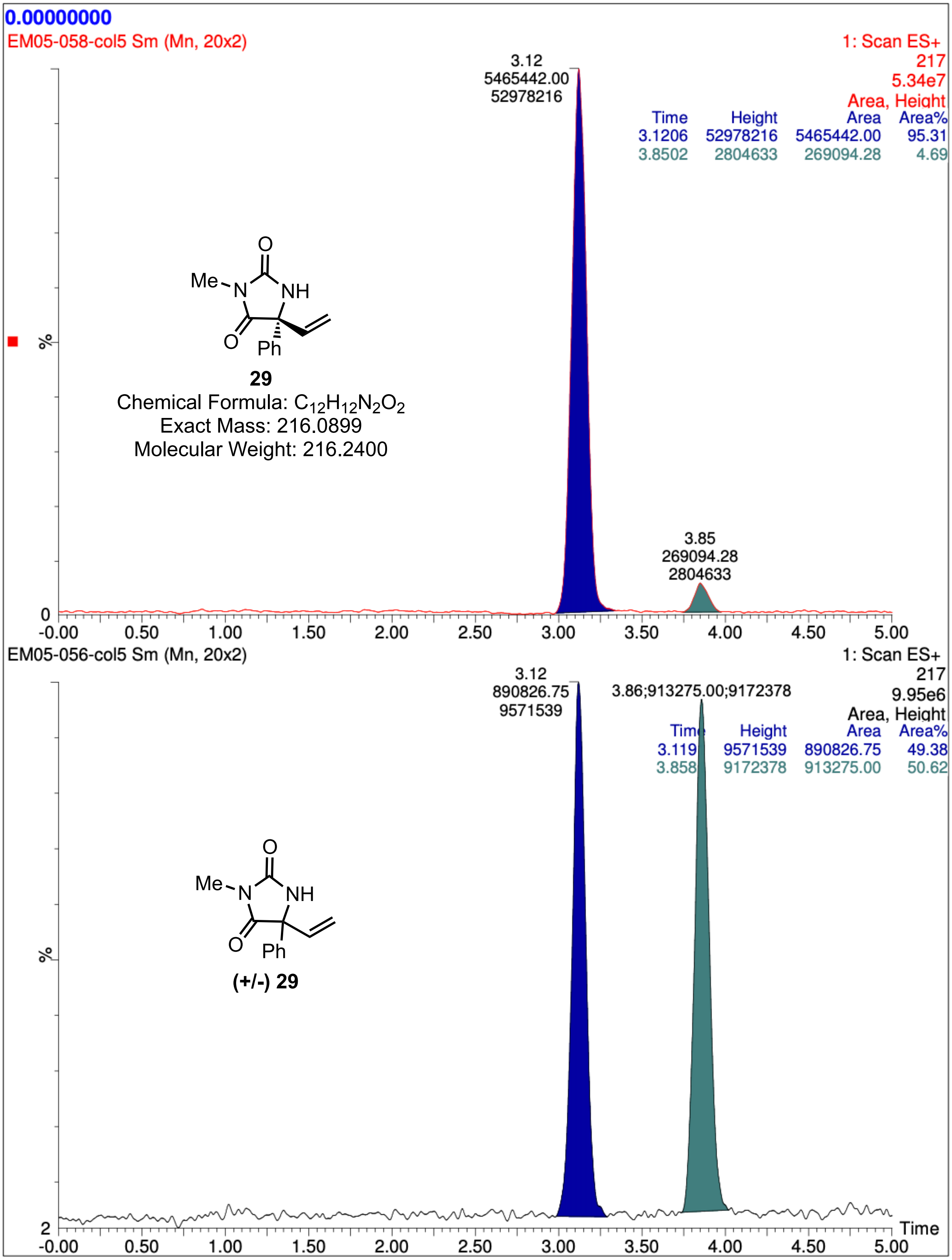
0.00000000
EM05-058-col5 Sm (Mn, 20x2)
1: Scan ES+
217
5.34e7
Area, Height
3.12
5465442.00
52978216
Time Height Area Area%
3.1206 52978216 5465442.00 95.31
3.8502 2804633 269094.28 4.69
Me
N
NH
O
O
Ph
29
Chemical Formula: $C_{12}H_{12}N_2O_2$
Exact Mass: 216.0899
Molecular Weight: 216.2400
%
3.85
269094.28
2804633
0
-0.00 0.50 1.00 1.50 2.00 2.50 3.00 3.50 4.00 4.50 5.00
EM05-056-col5 Sm (Mn, 20x2)
1: Scan ES+
217
9.95e6
Area, Height
3.12
890826.75
9571539
3.86;913275.00;9172378
Time Height Area Area%
3.119 9571539 890826.75 49.38
3.858 9172378 913275.00 50.62
Me
N
NH
O
O
Ph
(+/-) 29
%
2
-0.00 0.50 1.00 1.50 2.00 2.50 3.00 3.50 4.00 4.50 5.00
Time
