# Supplementary material for: Asymmetric Synthesis of Quaternary Hydantoins via a Palladium-Catalyzed Aza-Heck Cyclization
Source: J Am Chem Soc. 2025 Nov 14;147(49):44692–8. doi: 10.1021/jacs.5c16022 (PMC12703750; doi:10.1021/jacs.5c16022)
Supplement: Supplementary file 2 [file ja5c16022_si_002.zip › All NMR FID Files/S21/S21_AllNMR/TDI01-053.pdf]

TITLE PROJECT

Continued from page

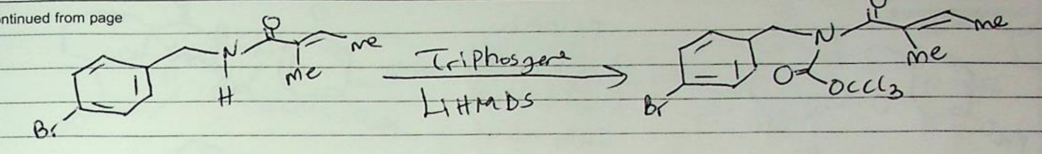

Reagents	MW	density	equiv	mmol	Amount
TD101052	268.15		1.0	10.0	2.7g
LiHMDS	167.33	0.86	1.1 (M=1.0)	11.0	11ml
Triphosgene	296.75		1.0	10.0	3.0g
THF			0.2M		50ml

Yield ⇒ 2.86g (6.65mmol) ⇒ 66% yield.

Procedure:

To an oven dried RBF (100ml) equipped with a magnetic stir bar, Crude (TD101052) was added and cycled 4x under N_2. Then anhy. THF was added, cooled to -78°C and LiHMDS was added dropwise and stirred for 5 min @ -78°C.

In a separate oven dried 50mL RBF, triphosgene and anhy. THF was added. This was added dropwise @ -78°C, warmed to rt and stirred over night.

Work up: Directly Concentrated

Purification: 100% hexanes → 10% EtOAc in hexanes.

Continued to page

SIGNATURE Temidayo

DATE

DISCLOSED TO AND UNDERSTOOD BY

DATE

PROPRIETARY INFORMATION
