# Supplementary material for: Asymmetric Synthesis of Quaternary Hydantoins via a Palladium-Catalyzed Aza-Heck Cyclization
Source: J Am Chem Soc. 2025 Nov 14;147(49):44692–8. doi: 10.1021/jacs.5c16022 (PMC12703750; doi:10.1021/jacs.5c16022)
Supplement: Supplementary file 2 [file ja5c16022_si_002.zip › All NMR FID Files/S17/S17_AllNMR/TDI01-061.pdf]

m.p. → 89 - 93°C

TITLE **PROJECT**

Continued from page

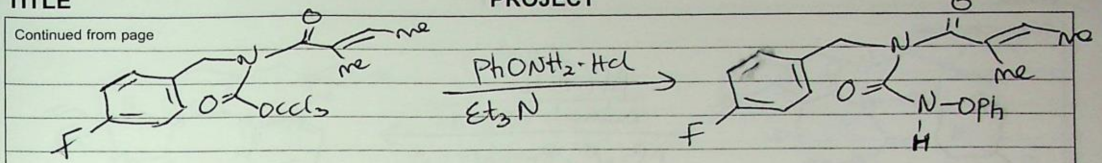

Reagents	MW	density	equiv	mmol	amount.
TD101060	368.61		1.0	5.0	1.84g
$PhONH_2 \cdot HCl$	145.51		2.0	10	1.5g
NEt_3	101.19	0.726	3.0	15	2.1ml
THF			0.2M		25ml

4.0 mmol → 1.47g

Procedure; Same as TD101056

Work up; Directly Concentrated.

0.451g (1.22 mmol).   m.p. 89 - 93°C

Continued to page

SIGNATURE Temi   DATE

DISCLOSED TO AND UNDERSTOOD BY   DATE

PROPRIETARY INFORMATION
