# Supplementary material for: Asymmetric Synthesis of Quaternary Hydantoins via a Palladium-Catalyzed Aza-Heck Cyclization
Source: J Am Chem Soc. 2025 Nov 14;147(49):44692–8. doi: 10.1021/jacs.5c16022 (PMC12703750; doi:10.1021/jacs.5c16022)
Supplement: Supplementary file 2 [file ja5c16022_si_002.zip › All NMR FID Files/S29/S29_AllNMR/Doc1.pdf]

TITLE Amide Coupling PROJECT

Continued from page

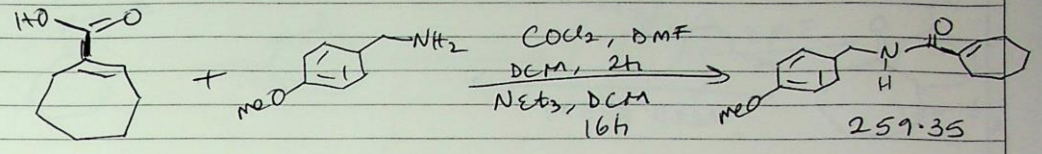


| Reagents | MW | density | equiv | mmol | Amount |
|---|---|---|---|---|---|
| TD101042 | 140.18 | | 1.0 | 8.0 | 1.12g |
| $COCl_2$ | 126.93 | 1.48 | 1.2 | 9.6 | 0.82ml |
| DMF | 73.09 | 0.948 | 0.05 | 0.4 | 0.03ml |
| DCM | 84.93 | 1.33 | 0.5M | — | 16ml |
| $NEt_3$ | 101.19 | 0.726 | 1.5 | 12 | 1.67ml |
| Amine | 137.18 | 1.06 | 1.1 | 8.8 | 1.14ml |

Procedure: Same as TD101016

Yield: mass = 2.06g ⇒ 7.94mmol (99% yield).

Continued to page

SIGNATURE [signature] DATE 07/24/23

DISCLOSED TO AND UNDERSTOOD BY DATE

PROPRIETARY INFORMATION
