# Supplementary material for: Asymmetric Synthesis of Quaternary Hydantoins via a Palladium-Catalyzed Aza-Heck Cyclization
Source: J Am Chem Soc. 2025 Nov 14;147(49):44692–8. doi: 10.1021/jacs.5c16022 (PMC12703750; doi:10.1021/jacs.5c16022)
Supplement: Supplementary file 2 [file ja5c16022_si_002.zip › All NMR FID Files/S20/S20_AllNMR/TDI01-052.pdf]

TITLE PROJECT m.P. ~74 – 80°C

Continued from page

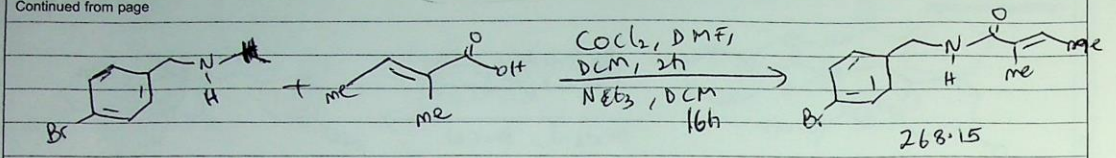

Reagents	MW	density	equiv	mmol	Amount
Tiglic acid	100.12		1.0	40	4g
$COCl_2$	126.93	1.48	1.2	48	4.12ml
DMF	73.09	0.948	0.05	2	0.15ml
DCM	84.93	1.33	0.5M	[illegible]	80ml
NEt_3	101.19	0.726	1.5	60	8.36ml
Br-Amine	186.06	1.473	1.1	44	8.2g → 5.6ml.

Yield → 10.4g (38.75mmol) → 97%.

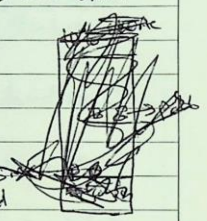

Procedure:

To an ovendried 250ml RBF equipped with magnetic stir bar and rubber septum, was attached to a double manifold and cooled under vacuum. Then Carboxylic acid was added, flask evacuated and backfilled with N_2 4x.

Anhydrous DCM was added, followed by DMF (anhydrous) and the reaction was cooled to 0°C in ice bath, then oxalyl chloride was added dropwise via syringe. The rxn mixture stirred for 15 mins @ 0°C and then warmed to rt over 2hrs. Excess oxalyl chloride + DCM was then removed in vacuo to give a white ppt. This was re-dissolved in DCM.

A separate ovendried 250ml RBF was cooled under vacuum and evacuated with N_2 3x. Anhydrous DCM, anhydrous NEt_3 and bromo amine were added to the RBF and cooled to 0°C in ice bath. Once cooled, the newly formed acyl chloride was added via cannula (extreme bubbling and exothermic!). The reaction was warmed to rt over 16h.

The reaction was quenched with NH_4Cl (Sat.) and organic layer washed with NH_4Cl 3x. Org. layer was then washed with brine, dried over $MgSO_4$ and concentrated. No further purification.

A yellowish red solid.

Continued to page

SIGNATURE [signature] DATE

DISCLOSED TO AND UNDERSTOOD BY DATE

PROPRIETARY INFORMATION
