# Supplementary material for: Asymmetric Synthesis of Quaternary Hydantoins via a Palladium-Catalyzed Aza-Heck Cyclization
Source: J Am Chem Soc. 2025 Nov 14;147(49):44692–8. doi: 10.1021/jacs.5c16022 (PMC12703750; doi:10.1021/jacs.5c16022)
Supplement: Supplementary file 2 [file ja5c16022_si_002.zip › All NMR FID Files/S30/S30_AllNMR/TDI01-050.pdf]

01 - 50
BOOK PAGE

**TITLE** **PROJECT**

Continued from page

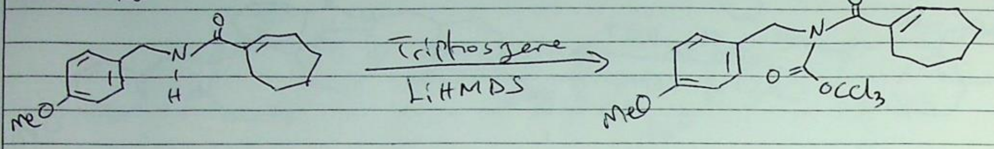


| Reagent | MW | density | equiv | mmol | Amount |
|---|---|---|---|---|---|
| TD101049 | 259.35 | | 1.0 | 3.5 | 0.91g |
| LiHMDS | 167.33 | | 1.1 (1.0M) | 3.85 | 3.85ml |
| Triphosgene | 296.75 | | 1.0 | 3.5 | 1.0g |
| THF | | | 0.2 | | 17.5 |

Procedure: Same as TD101017 64% yield 0.9446g.

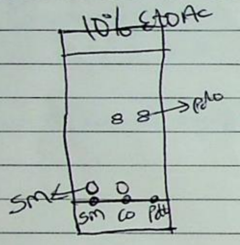


Continued to page

SIGNATURE [signature] DATE 08/30/23

DISCLOSED TO AND UNDERSTOOD BY DATE

PROPRIETARY INFORMATION
