# Supplementary material for: Asymmetric Synthesis of Quaternary Hydantoins via a Palladium-Catalyzed Aza-Heck Cyclization
Source: J Am Chem Soc. 2025 Nov 14;147(49):44692–8. doi: 10.1021/jacs.5c16022 (PMC12703750; doi:10.1021/jacs.5c16022)
Supplement: Supplementary file 2 [file ja5c16022_si_002.zip › All NMR FID Files/S31/S31_AllNMR/TDI01-197.pdf]

TITLE

PROJECT

Continued from page

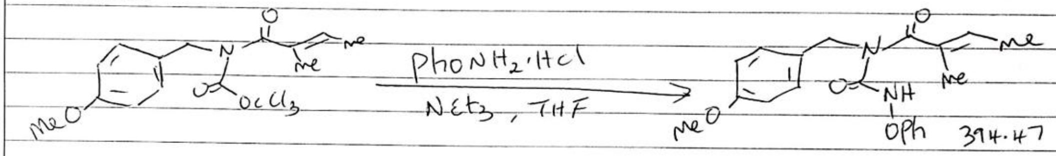

Reagents	MW	density	equiv	mmol	Amount.
TD101-196	420.71		1.0	2.0	0.85g
PhONH$_2$·HCl	145.59		2.0	4.0	0.6g
NEt$_3$	101.19	0.726	3.0	6.0	0.8ml
THF			0.2M		10ml

5% → 10% EtOAc in hexanes,

0.2973 + 0.1647 = 0.462 (1.17 mmol, (59%).

Continued to page

SIGNATURE

DATE
