# Supplementary material for: Asymmetric Synthesis of Quaternary Hydantoins via a Palladium-Catalyzed Aza-Heck Cyclization
Source: J Am Chem Soc. 2025 Nov 14;147(49):44692–8. doi: 10.1021/jacs.5c16022 (PMC12703750; doi:10.1021/jacs.5c16022)
Supplement: Supplementary file 2 [file ja5c16022_si_002.zip › All NMR FID Files/S22/S22_AllNMR/TDI01-178.pdf]

TITLE PROJECT

Continued from page

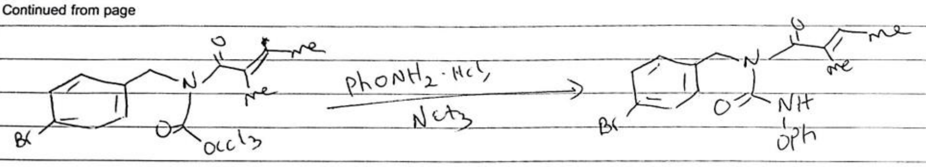

Reagents	MW	density	equiv	mmol	Amount
TD101-175	429.52		1.0	9mmol (6.6)	2.83g
$PhONH_2 \cdot HCl$	145.51		2.0	18.0	2.6g
NEt_3	101.19	0.726	3.0	27.0	3.8ml
THF			0.2M		45ml

Procedure: Same as TD101-~~[illegible]~~ 056

Yield ⇒ 1.52g ⇒ 4.6 mmol ⇒ 71%

Purification ⇒ Biotage: 100ml Column ⇒ 0% EtOAc → 10% EtOAc in hexanes.

Continued to page

SIGNATURE DATE
