# Supplementary material for: Asymmetric Synthesis of Quaternary Hydantoins via a Palladium-Catalyzed Aza-Heck Cyclization
Source: J Am Chem Soc. 2025 Nov 14;147(49):44692–8. doi: 10.1021/jacs.5c16022 (PMC12703750; doi:10.1021/jacs.5c16022)
Supplement: Supplementary file 2 [file ja5c16022_si_002.zip › All NMR FID Files/S15/S15_AllNMR/TDI01-073.pdf]

TITLE | PROJECT

Continued from page

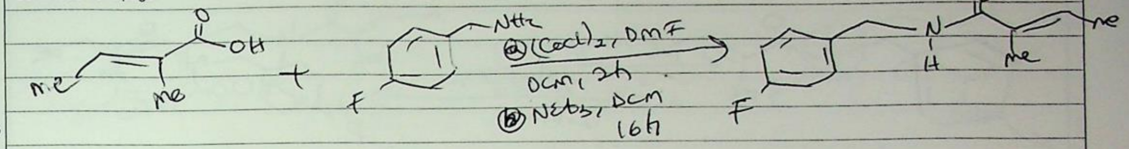


| Reagents | MW | density | mmol | equiv | Amount |
|---|---|---|---|---|---|
| Tiglic Acid | 100.12 | | 20 | 1 | 2g |
| $(COCl)_2$ | 126.93 | 1.48 | 24 | 1.2 | 2.05mL |
| DMF | 73.09 | 0.948 | 1 | 0.05 | 77 μL |
| DCM | 84.93 | 1.33 | [illegible] | 0.5M | 40mL |
| $NEt_3$ | 101.19 | 0.726 | 30 | 1.5 | 4.18mL |
| Amine | 125.14 | 1.095 | 22 | 1.1 | 2.51mL |

Procedures Same as TD 101016

7:15pm

Yield ⇒ 4.48g > 99% yield.

m.p → 66–71°C

Continued to page

SIGNATURE Temi DATE

DISCLOSED TO AND UNDERSTOOD BY DATE

PROPRIETARY INFORMATION
