# Supplementary material for: Asymmetric Synthesis of Quaternary Hydantoins via a Palladium-Catalyzed Aza-Heck Cyclization
Source: J Am Chem Soc. 2025 Nov 14;147(49):44692–8. doi: 10.1021/jacs.5c16022 (PMC12703750; doi:10.1021/jacs.5c16022)
Supplement: Supplementary file 2 [file TDI01-060.pdf › All NMR FID Files/S16/S16_AllNMR/TDI01-060.pdf]

01 - 60 Fluoro amide

BOOK PAGE

TITLE **PROJECT**

Continued from page

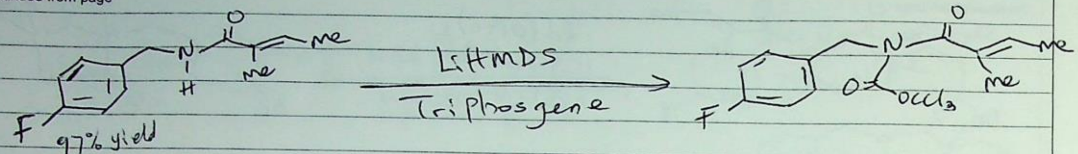

97% yield from MJE

Reagent	MW	density	equiv	mmol	Amount
MJE01-177	207.25		1.0	8.0	1.66g
LiHMDS	167.33	0.86	1.1(1.0M)	8.8	8.8ml
Triphosgene	296.75		1.0	8.0	2.4g
THF			0.2M		40ml

Procedure: Same as TDI 01 053.

Work up: Directly Concentrated

TLC: 10% EtOAc in hexanes

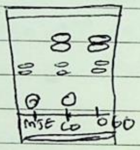

Column → 0% → 10% EtOAc in hexanes.

Yield ⇒ 1.71g → 4.6mmol (58%)

Continued to page

SIGNATURE Teri DATE

DISCLOSED TO AND UNDERSTOOD BY DATE

PROPRIETARY INFORMATION
